# Improving the accuracy of ^31^P NMR chemical shift calculations by use of scaling methods

**DOI:** 10.3762/bjoc.19.4

**Published:** 2023-01-10

**Authors:** William H Hersh, Tsz-Yeung Chan

**Affiliations:** 1 Department of Chemistry and Biochemistry, Queens College, Queens, NY 11367-1597, USA; 2 Ph.D. Program in Chemistry, The Graduate Center of the City University of New York, New York, NY 10016, USA

**Keywords:** calculations, DFT, phosphorus NMR, scaling methods, stereoisomers

## Abstract

Calculation of ^31^P NMR chemical shifts for a series of tri- and tetracoordinate phosphorus compounds using several basis sets and density functional theory (DFT) functionals gave a modest fit to experimental chemical shifts, but an excellent linear fit when plotted against the experimental values. The resultant scaling methods were then applied to a variety of “large” compounds previously selected by Latypov et al. and a set of stereoisomeric and unusual compounds selected here. No one method was best for all structural types. For compounds that contain P–P bonds and P–C multiple bonds, the Latypov et al. method using the PBE0 functional was best (mean absolute deviation/root mean square deviation (MAD/RMSD) = 6.9/8.5 ppm and 6.6/8.2 ppm, respectively), but for the full set of compounds gave higher deviations (MAD/RMSD = 8.2/12.3 ppm), and failed by over 60 ppm for a three-membered phosphorus heterocycle. Use of the M06-2X functional for both the structural optimization and NMR chemical shift calculation was best overall for the compounds without P–C multiple bonds (MAD/RMSD = 5.4/7.1 ppm), but failed by 30–49 ppm for compounds having any P–C multiple-bond character. Failures of these magnitudes have not been reported previously for these widely used functionals. These failures were then used to screen a variety of recommended functionals, leading to better overall methods for calculation of these chemical shifts: optimization with the M06-2X functional and NMR calculation with the PBE0 or ωB97x-D functionals gave values for MAD/RMSD = 6.9/8.5 ppm and 6.8/9.1 ppm, respectively, over an experimental chemical shift range of −181 to 356 ppm. Due to the unexplained failures observed, we recommend use of more than one method when looking at novel structures.

## Introduction

Calculation of ^1^H and ^13^C NMR chemical shifts and coupling constants using density functional theory (DFT) has increasingly become an adjunct to structure determination [1–8]. In particular for complex organic compounds, determination of relative stereochemistry using such calculations is a powerful technique [9–13]. A set of recommendations for best practices has been proposed [1–3,14] and made available online [15] describing basis set choices, geometry optimization, incorporation of solvation, and use of scaling factors derived from linear regressions between computed and experimental chemical shifts. In contrast, reports of calculation of ^31^P NMR chemical shifts – which span a range of roughly 500 ppm – have, even recently, used empirical methods [16], and theoretical methods have focused more on the choice of basis set to better match experimental chemical shifts of relatively small molecules [17–24]. For instance, while calculations for ^1^H NMR are considered to be sufficiently reliable using DFT methods with the 6-311+G(2d,p) basis set [15], work on ^31^P NMR chemical shifts has favored [18,21,24] the use of larger basis sets such as the IGLO-III [25–26] and pcS-n [27–28] basis sets with a focus on improved calculation of NMR shielding constants. Some theoretical work has been carried out on specialized phosphorus compounds that cover only a small range of chemical shifts, including nucleic acids [29–32] and polyoxometalates [33]. More recently, DFT methods have been applied to transition metal phosphorus complexes that cover a wide range of chemical shifts [34–35] using methods related to what we will describe here, but such studies are beyond the scope of this paper and typically focus on one metal at a time.

In the same way that improved results are obtainable for both ^1^H and ^13^C NMR chemical shifts using scaling methods, calculation of ^31^P NMR chemical shifts benefit as well from scaling. Chesnut first showed that scaling of the paramagnetic term of chemical shieldings calculated using the B3LYP functional and the 6-311+G(2d,p) basis set gave improved fits to experimental results, and further noted that while scaling is empirical, DFT methods themselves have an empirical component [36]. More recent studies have included a variety of functionals and basis sets in order to determine if there is a best combination in terms of accuracy and speed [22–23,37]. Even within the specialized studies of specific types of phosphorus compounds, scaling was found to give significant improvement [33]. A number of reports have speculated that scaling is needed to correct for rovibrational effects [21,24,36], and recent reports show that molecular dynamics methods can eliminate the need for empirical corrections [29–30,38]. However, these calculation-intensive methods are not likely to become routine soon, potentially leaving room for empirical scaling to be useful for some time.

The calculation-intensive and scaling approaches recently have been characterized by Jensen as the “purist” and “application” approaches [8], and the leading work on calculations of ^31^P NMR chemical shifts reflect one of each, namely, the “purist” approach reported by Krivdin [24] and the “application” approach due to Latypov [37]. The Krivdin group reported a group of 53 phosphorus compounds, which were first optimized at the MP2/6-311G(d,p) level with IEF-PCM solvation; ^31^P NMR chemical shifts were then calculated using the DFT KT2 functional [39] and a dual pcS-3/pcS-2 basis set [27–28]. In this case the use of the locally dense basis set approach, in which the larger pcS-3 basis set was used on phosphorus and the smaller pcS-2 basis set was then used on all the other atoms, allowed the computation of chemical shifts for a variety of benchmark compounds up to 35-atom triphenylphosphine oxide. Comparison of the calculated chemical shifts with the experimental values gave a mean absolute deviation and root mean square deviation (MAD/RMSD) [3,40] of 9.4/12.0 ppm over a roughly 550 ppm range. The Latypov group reported a training set of 22 phosphorus compounds, which were first optimized at a significantly lower but more easily implemented level of theory (PBE0 functional [41], 6-31+G(d) basis set, gas phase), followed by calculation of the ^31^P NMR chemical shifts with the same PBE0 functional with the still-modest 6-311G(2d,2p) basis set. While the calculated fit to experimental values was significantly worse than the higher-level Krivdin method (MAD/RMSD = 18.7/21.9 ppm), the linear fit of the *calculated* chemical shifts of the 22 training set compounds to their *experimental* values gave a scaled set of corrected calculated values with a much-improved MAD/RMSD = 9.3/10.9 ppm over a roughly 500 ppm range. That is, scaling of the theoretical results to experimental values gave values with deviations comparable to the unscaled results obtained at much higher levels of theory. In addition, the Latypov results provide a prescription that could be applied readily to larger and more interesting novel compounds. A collection of 10 such compounds actually gave a somewhat better scaled MAD/RMSD of 6.9/9.0 ppm, using the scaling factors derived from the benchmark set of 22 compounds.

In addition to the above review of ^31^P chemical shift calculations, a recent review by Krivdin covered an additional range of factors for higher-level calculations, scaling, and a variety of specialized compounds [42]. In this paper we describe work that had been in progress when the Latypov group’s report was published. It is similar in style, in that we describe the use of significantly lower levels of DFT calculations than the Krivdin group, but the motivation was to develop a high-accuracy method that would allow identification of both unusual phosphorus compounds and of stereochemistry, and still be accessible to organic chemists without specialized software. In addition, we were concerned by some of the choices made by the Latypov group for their training set of compounds, and so sought to use a much simpler set of phosphorus compounds for scaling purposes. We report here (1) a comparison of basis sets for calculation of a range of ^31^P NMR chemical shifts of well-known tri- and tetracoordinate phosphorus compounds, (2) development of scaling factors for calculation of ^31^P NMR chemical shifts, and (3) application of this method to determination of stereochemistry at phosphorus in heterocycles and to corroboration of some unusual compounds that have been reported previously. At that point we were left with two or possibly three structural types for which some of these methods failed to provide accurate chemical shifts, and so we report here (4) a search of 23 more recent combinations of DFT functionals that have been recommended for theoretical reasons, expedited by focusing on these failures.

## Results and Discussion

**1. Experimental chemical shifts.** A number of compilations of ^31^P NMR chemical shifts were published early in the development of NMR [43–45], followed by book-length compilations [46–48]. These chemical shifts were referenced to external 85% H_3_PO_4_ at 0 ppm (with positive values reported here downfield of H_3_PO_4_). The early work of necessity included mostly pure liquid samples, that is, without any deuterated solvents, and one of the compilations noted that chemical shift changes upon dilution with CS_2_, CCl_4_, CHCl_3_, and ethanol were all small (<2 ppm) [43]. As much as possible, we have tried to use chemical shifts in CDCl_3_ solution, and for the cases where we have literature data or have measured the chemical shifts ourselves, have found the solution values are very close to the reported liquid values (liquid/CDCl_3_ in ppm: PCl_3_ 220 [44]/219.79; P(OMe)_3_ 141 [44]/141.41; (iPrO)_2_P(O)CH_3_ 27.4 [49]/28.61; (iPrO)_2_P(O)H 4.2 [43]/4.54; PMe_3_ −62 [44]/−61.58 [50]). We have also included chemical shifts in more polar solvents (i.e., DMSO and methanol) when those were the only reported values.

The chemical shifts of two commonly used reference standards for ^31^P NMR calculations require comment, since a reference is needed to convert the calculated absolute magnetic shielding (σ) to the chemical shift (δ). The calculated chemical shift δ(^31^P)_calcd_ is given by the difference between the absolute magnetic shielding values of the reference and the desired phosphorus compound calculated at the same level of theory, plus the experimental chemical shift of the reference compound (Equation 1) [18,21,26].


[1] δ(P31)calcd=σ(reference)calcd−σ(P31)calcd+  δ(reference)exp

Since the 85% H_3_PO_4_ reference standard is a roughly 1:1 molar solution of phosphoric acid in water [51], calculation of its absolute magnetic shielding might be expected to be complicated by water solvation, as well as ionization or aggregation of the phosphoric acid in water, and calculation as a gas-phase chemical shift is also unreasonable [51]. Because of these issues, other studies have used PH_3_ as an alternative theoretical reference standard [18–19], despite the fact that actual use of this compound requires a fairly extraordinary experimental setup [52–53]. An additional issue with PH_3_ involves the choice of using the gas-phase or liquid-phase experimental chemical shifts, which differ dramatically: the universally used value for the gas-phase chemical shift is −266.1 ppm (referenced to external 85% H_3_PO_4_) [54], while the liquid-phase chemical shift is −238 ppm at −90 °C [44] and is also −238 ppm at 23 °C in CCl_4_ [52]. In the two ^31^P NMR studies described in the Introduction, Latypov used the gas-phase value [37] and Krivdin the liquid-phase value [24]. As noted above, the CDCl_3_ solution values are close to the liquid values, and so we will use the −238 ppm chemical shift for PH_3_, and would argue this value is the correct one for comparisons to other solution spectra. We would further argue that the simplest solution to the reference problem when scaling is used would be the calculation of all chemical shifts referenced to H_3_PO_4_ with water solvation since this provides values that can be compared immediately to experimental chemical shifts; further, as will be explained below, scaling eliminates the need for the calculated absolute shielding of the reference.

**Figure 1:**
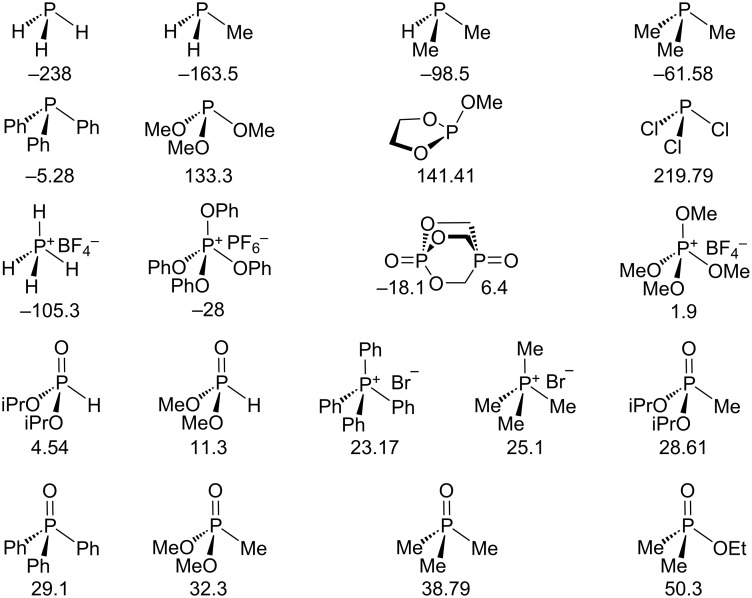
Training set of tri- and tetracoordinate phosphorus compounds; chemical shifts are in ppm, referenced to external 85% H_3_PO_4_ at 0 ppm (positive values downfield of H_3_PO_4_).

**2. Calculation of chemical shifts.** A small number of trivalent phosphorus compounds spanning a roughly 460 ppm chemical shift range was chosen initially to examine basis set effects, namely, PH_3_, PMeH_2_, PMe_2_H, PMe_3_, PPh_3_, P(OMe)_3_, methoxyphospholane (i.e., MeOP(OCH_2_CH_2_O)), and PCl_3_, each of which has been used in recent reports of phosphorus chemical shift calculations [18,20–21,24,37] except for the phospholane, and more surprisingly with one exception [24], PPh_3_ (Figure 1; see Computational and NMR Details section). For each of these, optimization (Gaussian 09 [55], DFT with 6-31+G(d,p) basis set) included solvation using the default polarizable continuum model (IEF-PCM using CHCl_3_), and except for trimethylphosphite resulted in one energy minimum. We found three local minima for trimethylphosphite, so NMR spectra were calculated for each and the energy-weighted average was used for the calculated chemical shift; methoxyphospholane was added as an alternative to trimethylphosphite simply because it seemed likely that it would exhibit only one local minimum, and so could provide a check on the calculated chemical shift. The choice of basis set for the optimizations was guided by recommendations by Tantillo and co-workers [3] for cases involving multiple conformations, and in addition here the presence of lone pairs on third-row atoms provides additional reason to use a higher level basis set than the usual 6-31G(d) [3]. As pointed out by van Wüllen [18], the energy-optimized structure for PCl_3_ is not a good fit to the experimental geometry [56], and the chemical shift changes significantly with geometry. Chemical shift values for both the optimized structure and the experimental geometry were therefore calculated, and the latter was much closer to the experimental chemical shift. For each compound, GIAO calculation of the chemical shift was carried out first using the widely used 6-311+G(2d,p) basis set with the B3LYP and PBE0 functionals, both used for ^1^H NMR calculations [3,15]. One lower-level basis set was used (6-311G(d,p)) [21], as well as two higher-level basis sets used by others [18,21,24] (IGLO-III [25–26] and pcS-2 [27–28]) specifically optimized for ^31^P, each with the B3LYP functional. Another widely used functional, M06-2X [57–58], was also used for the optimization (again using the 6-31+G(d,p) basis set) with the NMR chemical shifts calculated using both the B3LYP and M06-2X functionals with the 6-311+G(2d,p) basis set and IEF-PCM (CHCl_3_) solvation. Last, two versions of the Latypov method were calculated. In one, the structures were both optimized (PBE0/6-31+G(d,p), IEF-PCM using CHCl_3_) and the NMR calculated (PBE0/6-311+G(2d,p), IEF-PCM using CHCl_3_) in the same manner as the calculation methods described above. In the other, the same method as used by Latypov (PBE0 gas phase 6-31+G(d) optimization followed by PBE0 gas phase 6-311G(2d,2p) NMR) was carried out in order to allow a direct comparison [37]. Results may be found in Tables S1–S3 in Supporting Information File 1 for both the absolute chemical shifts and the chemical shifts referenced to that calculated for 85% H_3_PO_4_ at 0 ppm according to Equation 1. As described above, the same functionals and basis sets were used for H_3_PO_4_ but with water solvation, except when using the gas-phase Latypov method.

As expected, on the basis of the results reported by Krivdin and Latypov, the agreement between experimental and calculated chemical shifts, regardless of the basis set, for these simple compounds is poor. The MAD/RMSD values averaged 21/25 ppm and were remarkably similar for the nine combinations of DFT functionals, optimizations, and basis sets chosen. This was true both for the two larger basis sets optimized for phosphorus (IGLO-III and pcS-2) and the smaller basis set (due to the absence of diffuse orbitals) that gave significantly shortened computational times (6-311G(d,p)) [21].

A second set of tetracoordinate phosphorus compounds was then added to the trivalent set for scaling (Figure 1). In contrast to common trivalent phosphorus compounds, the chemical shifts of typical tetracoordinate analogues do not span such a large range, nor have calculations been routinely reported. A set of 13 compounds (with 14 different phosphorus atoms) was chosen, including PY_4_^+^ (Y = H, Me, Ph, OMe, OPh), Y_3_P=O (Y = Me, Ph), O=P(OCH_2_)_3_P=O, Y_2_P(O)H (Y = MeO, iOPr), Y_2_P(O)Me (Y = MeO, iOPr), and EtOP(O)Me_2_. Despite spanning a chemical shift range of only about 150 ppm, this set exhibited a wider range of structural types than the trivalent compounds. A number of the compounds required inclusion of several conformational isomers, and for the cationic compounds, the counterions were included, and the experimental chemical shifts included compounds as pure liquids and as CDCl_3_, CH_2_Cl_2_, CH_3_CN, DMSO, and CH_3_OH solutions (see Computational and NMR Details section). Minimizations and NMR chemical shift calculations were carried out as before, albeit all with CHCl_3_ solvation, and once again as expected, the average MAD/RMSD values of 9/12 ppm were high (albeit lower than the trivalent compounds) and together the MAD/RMSD values averaged 13.5/18.1 ppm.

**3. Scaling of chemical shifts.** Each of the sets of calculated chemical shifts was next plotted against the experimental chemical shifts according to Equation 2 to give an empirical scaling relationship [36] with a unique slope and intercept for both the training set compounds and the calculation methods used (Table S4 in Supporting Information File 1).


[2] δ(P31)experimental=m[δ(P31)calcd]+b

**Figure 2:**
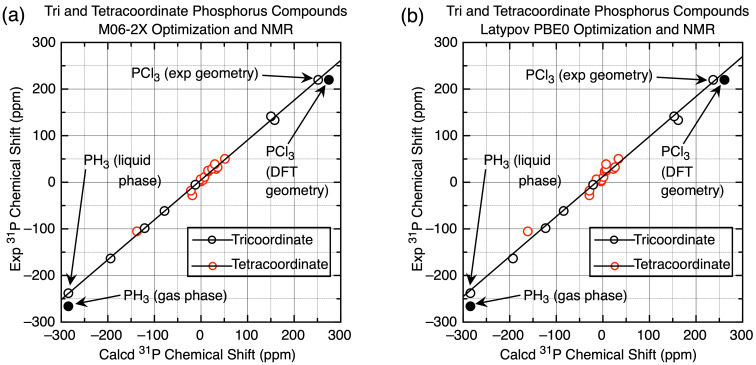
(a) Plot of experimental vs calculated chemical shifts of tri- and tetracoordinate phosphorus compounds (M06-2X optimization and NMR (6-311+G(2d,p) basis set) referenced to H_3_PO_4_, IEF-PCM CHCl_3_ solvation). Equation 2 *m* = 0.855 ± 0.011, *b* = 4.91 ± 1.27, giving scaled MAD/RMSD = 4.1/5.7 ppm (Equation 3). (b) Plot of experimental vs calculated chemical shifts of tri- and tetracoordinate phosphorus compounds (Latypov PBE0 optimization and NMR (6-311G(2d,2p) basis set) referenced to H_3_PO_4_, gas phase). Equtation 2: *m* = 0.858 ± 0.019, *b* = 12.36 ± 2.19, giving MAD/RMSD = 7.8/9.7 ppm. For both (a) and (b), see Table S3 in Supporting Information File 1; the points for PCl_3_ (DFT calculated geometry) and PH_3_ (gas phase) were not included in the linear fits.

This resulted in extraordinarily linear fits for the trivalent phosphorus compounds spanning the full chemical shift range, and a bit more scatter of the tetracoordinate compounds over their smaller range. The slope and intercept were then used to convert the DFT-calculated values to the empirically more accurate scaled values according to Equation 3, allowing the scaled MAD/RMSD values to be determined (Table 1 and Table S5 (Supporting Information File 1), and Figure 2).


[3] δ(P31)scaled=m[δ(P31)calcd]+b

**Table 1:** MAD^a^ and RMSD^a^ (ppm) for scaled ^31^P NMR chemical shifts.^b^

Optimization functional^c^	NMR functional^c^	NMR basis set^c^	MAD/RMSD all data^d^

B3LYP	B3LYP	6-311G(d,p)	8.9/10.6
B3LYP	B3LYP	6-311+G(2d,p)	7.0/8.4
M06-2X	B3LYP	6-311+G(2d,p)	6.5/8.0
M06-2X	M06-2X	6-311+G(2d,p)	**4.1/5.7**
B3LYP	B3LYP	IGLO-III	6.0/7.1
B3LYP	B3LYP	pcS-2	6.5/8.0
B3LYP	PBE0	6-311+G(2d,p)	6.2/7.6
PBE0	PBE0	6-311+G(2d,p)	5.9/7.1
PBE0^e^	PBE0^e^	6-311G(2d,2p)^e^	7.8/9.7

^a^MAD/RMSD: mean absolute deviation (MAD = Σ*_n_*|δ_calc_ − δ_exp_|/*n*) and root mean square deviation (RMSD = [Σ*_n_*(δ_calc_ − δ_exp_)^2^/*n*]^1/2^) [3,40]. ^b^In all tables, notable results (i.e., best and among the worst) are in bold, and the very best in bold italics. ^c^Optimization basis set/solvent and NMR solvent, except as noted: 6-31+G(d,p)/CHCl_3_. ^d^Deviations calculated for the training set of tri- and tetracoordinate phosphorus compounds (Supporting Information File 1, Table S5, 22 points). ^e^Optimization (6-31+G(d)/gas phase) and NMR (gas phase) for the same training set of tri- and tetracoordinate phosphorus compounds, but following Latypov’s method [37].

The average MAD/RMSD values dropped from 13.5/18.1 ppm to an average of 6.0/7.4 ppm for most of the optimization/NMR combinations; the B3LYP method using a smaller basis set and the Latypov method using the phosphorus compounds described above both exhibited higher deviations. The higher deviations for the Latypov method (7.8/9.7 ppm), using gas-phase calculations and smaller basis sets with this set of phosphorus compounds, compared to our method (5.9/7.1 ppm) with CHCl_3_ solvation and the larger basis sets, is notable. The greater scatter is clearly visible by comparing the scaling plot for the Latypov method in Figure 2b to the best fit method using the M06-2X functional for both optimization and the NMR calculation (Figure 2a, MAD/RMSD = 4.1/5.7 ppm). For both scaling plots, the fit shown occurs using the experimental rather than the calculated geometry for PCl_3_, consistent with van Wüllen’s observation [18] noted above. Also consistent with our observation on the use of the liquid chemical shift value for PH_3_, the fit for both scaling plots shown occurs with the liquid rather than the gas chemical shift. That is, as seen in Figure 2a, the values for the liquid PH_3_ and the experimental geometry for PCl_3_ are both clearly in line with the other chemical shifts, and while the fit is not quite as good in Figure 2b, it is close. Use of the −266.1 ppm gas-phase value for PH_3_ (as was done by Latypov [37]) for Figure 2b does not shift the scaling line significantly (giving a calculated scaled shift of −240.5 ppm) and a worse fit (MAD/RMSD = 8.5/11.6 ppm vs the 7.8/9.7 ppm shown for the −238 ppm liquid-phase value shown).

Since the absolute shielding of the reference (here σ(H_3_PO_4_)_calcd_) is a constant, one can equally well create a scaling equation using just the experimental vs the calculated absolute shieldings by rearrangement of Equation 1 and Equation 2 to give Equation 4, where the intercept *b*_1_ in Equation 4 simply incorporates the calculated shielding and chemical shift of the reference as shown.


[4]

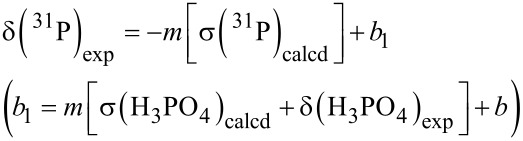



Calculation of the absolute shielding of the reference is therefore irrelevant if one is using a scaling method, and Equation 4 gives the identical scaled chemical shifts without the need to calculate the reference shielding. In the study reported by Latypov [37], gas-phase values for the H_3_PO_4_ calculation were used for this purpose, so only the scaled chemical shifts would be expected to be valid. In the study reported by Krivdin [24], a partially experimental absolute shielding for 85% H_3_PO_4_ in water was used (σ(85% H_3_PO_4_) = 351.6 ppm), derived from the experimental chemical shift of PH_3_ referenced to 85% H_3_PO_4_ [53]. The use of this value has the virtue of eliminating all theoretical complications of conformations and hydrogen bonding in phosphoric acid [51], since it is not based on a DFT-optimized structure. However, the goal of Krivdin’s work was the direct calculation of chemical shifts without scaling, so that a plot of experimental vs calculated chemical shifts as in Figure 2 would give a slope of 1 and (for the H_3_PO_4_ reference) an intercept of 0; actual values were 0.977(16) and 1.2(1.7) [24]. Nevertheless, for the present work we will use Equations 1–3 as they are intuitively straightforward and provide an approximate check via the unscaled chemical shifts, and will expect to derive a scaling relationship since the level of calculation is considerably lower than that of Krivdin’s work.

**4. Calculated ^31^P NMR chemical shifts for the Latypov test compounds.** The next step, then, requires application to a variety of compounds not yet included in the training set. One such set is the set of tri- and tetracoordinate phosphorus compounds that we have already calculated chemical shifts for using the Latypov method, so if our training set is comparable to the Latypov training set, then we should obtain a similar scaling equation and similar MAD/RMSD values. In fact we do not, even though both training sets cover a similar chemical shift range with a similar number of data points. The slope of the Latypov scaling equation is 0.925, which is significantly higher than the slopes for all of the scaling equations using the tri- and tetracoordinate phosphorus compounds (i.e. that for the Latypov PBE0/PBE0 method using our training set gave a slope of 0.858, Figure 2b). The Latypov scaling equation gave a MAD/RMSD = 9.3/12.3 ppm for the tri- and tetracoordinate phosphorus compounds, so the question arose as to why the two training sets would be so different.

**Figure 3:**
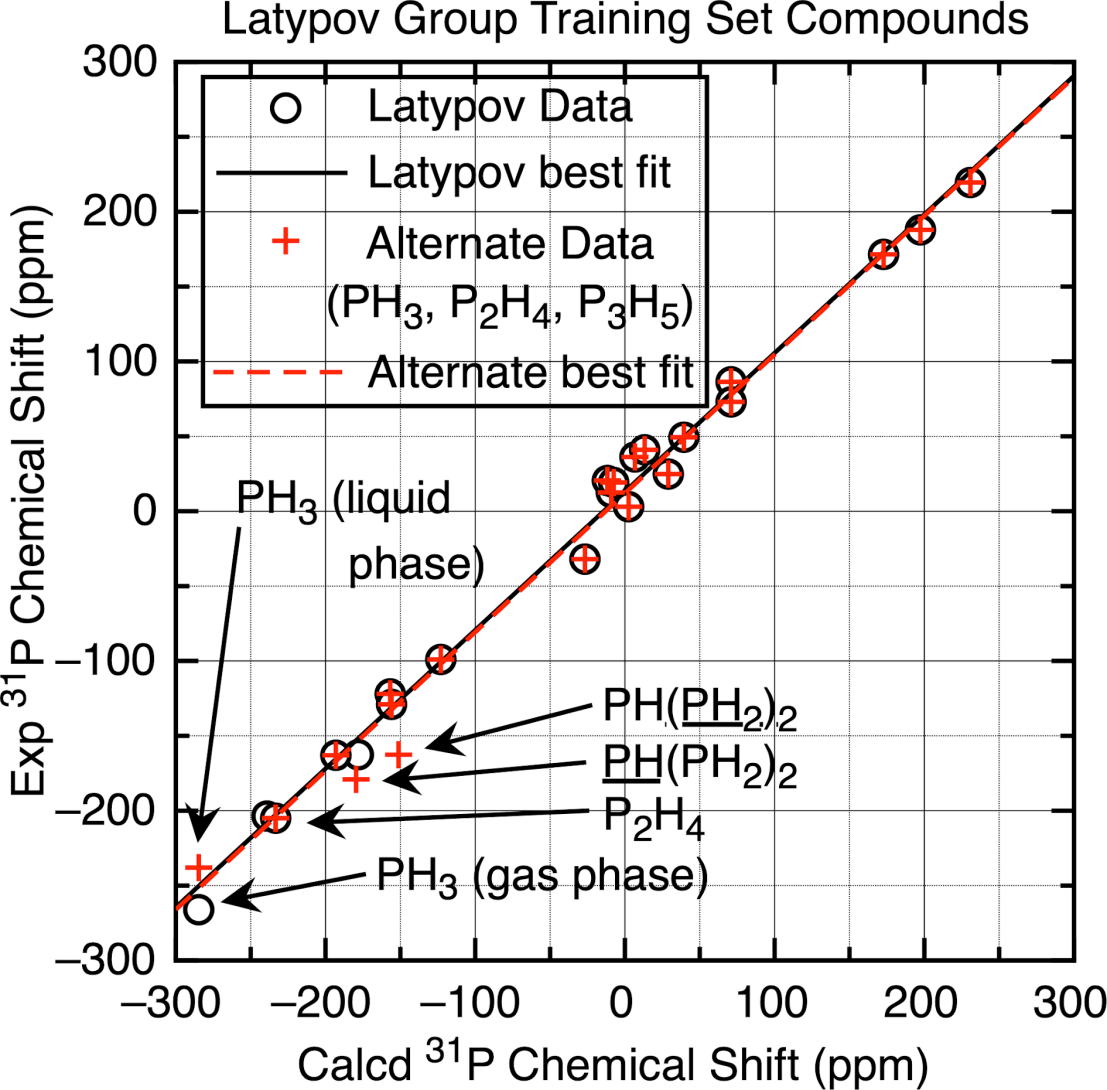
Plot of experimental vs calculated chemical shifts of training set compounds reported by Latypov et al. [37] (open circles, PBE0/6-31+G(d) gas-phase optimization, PBE0/6-311G(2d,2p) gas phase NMR referenced to gas phase H_3_PO_4_), and the best fit line to give scaling Equation 2 *m* = 0.925 ± 0.017, *b* = 13.2 ± 2.5, MAD/RMSD = 9.1/10.9 ppm. Alternate data includes PH_3_, (H_2_P)_2_ (virtually unchanged), and both P atoms in (H_2_P)_2_PH instead (red + symbols) and best fit line to give scaling Equation 2 *m* = 0.926 ± 0.021, *b* = 11.9 ± 3.1, MAD/RMSD = 10.7/13.7 ppm.

As described above, one difference is the choice of the PH_3_ chemical shift for the training set; by using the lower gas-phase value, the slope of the scaling equation will increase, as seen from 0.858 to 0.925, and the effect is amplified since this is the lowest chemical shift as seen in Figure 2. However, there are also other discrepancies. For instance, two of the highest field experimental chemical shifts are listed in the Latypov report [37] as −162.6 ppm for (H_2_P)_2_PH and −203.6 ppm for (H_2_P)_2_ [59]. Problems include (a) the Latypov group did not specify whether the PH or PH_2_ moiety is the one exhibiting the peak at −162.6 ppm in (H_2_P)_2_PH, (b) there is no reason both moieties should not be included, (c) the chemical shift values were determined using a complex INDOR spectrum of a mixture of compounds, exhibiting 47 lines, and so certainly subject to interpretation (and uncertainty), and (d) both (H_2_P)_2_PH and (H_2_P)_2_ exhibited multiple conformations in our hands that especially for (H_2_P)_2_PH affected the calculated chemical shifts, and (e) while we did not check all chemical shifts, the citation in reference [37] for CH_2_=C(H)PF_2_, the furthest downfield point (and hence a critical value) is wrong, although the value of 219.5 ppm is correct [60]. While these uncertainties in both the experimental and calculated chemical shifts suggest this training set may be unreliable, as seen in Figure 3 where we have plotted the Latypov data and our limited changes, these data points almost exactly cancel out in their effect on the best-fit scaling line. The MAD/RMSD of 9.5/12.4 ppm for our tri- and tetracoordinate phosphorus compounds was virtually the same as that using the original training set data reported by the Latypov group – that is, both gave an equally poor fit to the simplest phosphorus compounds.

**Figure 4:**
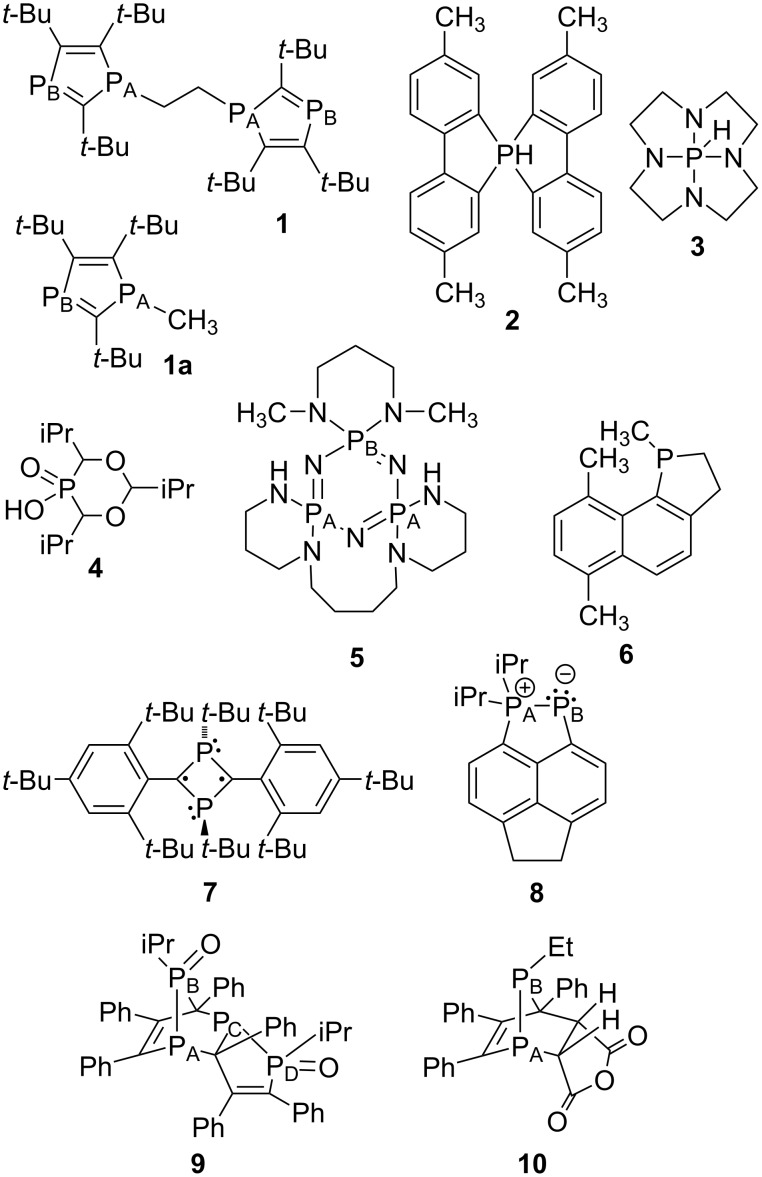
“Large” compounds selected for ^31^P NMR calculation by Latypov [37].

The next step requires use of the scaling equations to calculate chemical shifts for a set of 10 “large” compounds chosen by Latypov (Figure 4; there are a number of errors in the drawings and references in the Latypov paper so they are all redrawn and referenced here) [37]. Compound **1** chosen by Latypov had many different minima upon optimization in our hands, in some cases giving widely divergent chemical shifts for the two chemically equivalent phosphorus atoms, which could potentially exacerbate any deviations from the experimental chemical shifts. Since derivatives with methyl (**1a**) and butyl substitution on P_A_ were reported and had comparable chemical shifts [61], we substituted compound **1a** for **1**. Going forward, we used the optimization and NMR functional combinations from Table 1 that gave the lower set of MAD/RMSD values with some exceptions. First, the large IGLO-III and pcS-2 basis sets failed to overcome any deficiencies in the functional used, at least for the B3LYP case, and due to the significantly higher calculation time, these basis sets were not evaluated with other functionals. Since the IGLO-III basis set was better than the pcS-2 basis set, we kept this into the next round. In order to minimize the number of different optimization calculations, we dropped the PBE0 optimization with CHCl_3_ solvation and just included the Latypov gas-phase method, scaled both to our tri- and tetracoordinate phosphorus compound training set and to the original Latypov training set.

On the basis of the MAD/RMSD criterion (Table 2), the Latypov method using the Latypov training set is superior for this collection of compounds; the substitution of **1a** – which still exhibited two conformations – for **1** actually gave better results for the Latypov method than for the others, and gave a much better fit than had **1**. Inspection of the deviations showed that (1) use of the large IGLO-III basis set did not provide any advantage, and (2) the B3LYP and M06-2X methods using the M06-2X optimization were mostly comparable to the Latypov PBE0 method when scaled to our collection of compounds (i.e., MAD ranging from 9.1–11.4 ppm), but not to the Latypov training set. These B3LYP and M06-2X methods initially appeared to fare worst for compounds having P–P bonds (i.e., **8–10**), while the M06-2X NMR calculation for compound **1a** gave a large downfield chemical shift deviation for the phosphorus atom with a multiple bond to carbon. However, the largest deviation for the P–P-bonded compounds was that of P_B_ in **8** of up to roughly 40 ppm. An alternative hypothesis for this deviation might be that it could be due to multiple P–C bond character rather than the P–P bond itself, due to π overlap of dicoordinate P_B_ with the naphthalene ring. The MAD/RMSD values for the limited number of data points for compounds **9** and **10** support this in that the MAD values for the M06-2X and PBE0 NMR calculations (using the tri- and tetracoordinate phosphorus scaling equation) were similar (10.6–11.1 ppm). However, the MAD value for the M06-2X NMR calculation for P_B_ in **1a** and P_B_ in **8**, that is the atoms that we propose have multiple P–C bonding, were double (25.3 ppm) the P–P MAD value.

**Table 2:** Experimental^a^ and scaled^b 31^P NMR chemical shifts^c^ for Figure 4 compounds.

Optimization functional:		B3LYP	B3LYP	M06-2X	M06-2X	PBE0	PBE0
NMR functional:		B3LYP	B3LYP	B3LYP	M06-2X	PBE0^d^	PBE0^d,e^
Basis set for NMR:		6-311+G(2d,p)	IGLO-III	6-311+G(2d,p)	6-311+G(2d,p)	6-311G(2d,2p)	6-311G(2d,2p)

Compound	Exp.^f^						

**1a** P(A)	39.6 [61]	46.8	45.2	48.4	56.1	37.2	40.3
**1a** P(B)	297.6	286.3	280.0	288.1	**338.9**	279.1	303.0
**2**	−110 [62]	−109.1	−103.4	−108.8	−106.2	−98.6	−107.2
**3**	−54.5 [63]	−50.7	−47.8	−50.1	−47.1	−46.5	−50.5
**4**	24 [37]	23.8	25.4	22.4	26.1	31.7	34.3
**5** P(A)	18.3 [64]	10.2	11.4	13.0	20.3	17.3	18.8
**5** P(B)	27.6	17.9	19.2	22.2	28.2	25.2	27.4
**6**	−10.2 [65]	3.0	4.5	0.1	−5.7	−7.5	−8.1
**7**	38.7 [66]	45.0	42.7	39.3	44.8	43.9	47.7
**8** P(A)	76.7 [67]	74.9	77.7	74.9	75.3	67.2	72.9
**8** P(B)	−157.7	**−110.6**	**−112.4**	−121.8	**−115.5**	−135.6	−147.3
**9** P(A)	−29.7 [68]	−4.8	−3.9	−19.6	−24.4	−23.6	−25.7
**9** P(B)	100.5	90.7	91.7	85.5	94.5	84.5	91.7
**9** P(C)	−10.6	15.4	14.5	15.1	13.4	6.3	6.7
**9** P(D)	75.7	64.3	66.1	65.4	72.5	62.7	68.1
**10** P(A)	−22.6 [69]	1.0	2.6	−5.9	−9.8	−19.0	−20.6
**10** P(B)	84.1	105.8	104.5	93.3	99.1	76.1	82.6
MAD^g^		13.3	13.7	10.1	11.4	**9.1**	***5.3***
RMSD^g^		17.7	17.7	13.5	17.0	**11.0**	***7.0***
**9**, **10** (P*–*P)^h^							
MAD		19.6	19.2	14.5	11.1	10.6	6.9
RMSD		20.6	20.5	15.6	13.2	11.7	8.7
**1a**, **8** (P=C))^i^							
MAD		16.8	17.3	14.0	**25.3**	13.1	***5.1***
RMSD		24.5	24.4	19.1	**30.7**	15.2	***6.2***
**2**–**7**, **9**, **10**^j^							
MAD		12.3	12.6	**8.9**	**7.1**	**7.8**	***5.4***
RMSD		15.0	15.1	**11.2**	**9.5**	**9.3**	***7.2***

^a^Experimental chemical shifts referenced to external 85% H_3_PO_4_ at 0.00 ppm, positive values downfield. ^b^NMR calculations were carried out using GIAO with IEF-PCM solvation on optimized structures (DFT/6-31G+(d,p)/IEF-PCM) except as noted for the last two columns; the IGLO-III basis set was taken from the Basis Set Exchange [70], and all other basis sets were taken from Gaussian. Except as noted for the final column, all calculations scaled using the tri- and tetracoordinate training set. ^c^Chemical shifts calculated from the absolute isotropic chemical shieldings according to Equation 1 (Tables S6–S8 in Supporting Information File 1), where H_3_PO_4_ was optimized and its NMR was calculated using the same basis sets and functionals, and except for the final two columns, IEF-PCM using water, and scaled using parameters in S4 (Equation 2) giving scaled shifts (Supporting Information File 1, Table S9, Equation 3). ^d^Optimized (PBE0/6-31+G(d)/gas phase), NMR (PBE0/6-311G(2d,2p)/gas phase), and H_3_PO_4_ reference (gas phase) following Latypov [37]. ^e^Scaled using the Latypov training set, and values for **2**–**10** taken from Latypov [37]. ^f^Compounds **1a**–**4** were measured and calculated in benzene, and **5**–**10** in chloroform. ^g^See note a in Table 1. ^h^MAD/RMSD (ppm) for compounds that contain a P–P bond with no P–C multiple bonding. ^i^MAD/RMSD for compounds with P–C multiple bonding. ^j^MAD/RMSD (ppm) for compounds that contain no P–C multiple bonding.

At least part of the cause for the P–P chemical shift deviations might be that the Latypov method gives better agreement of the P–P bond lengths with those observed by X-ray, but simply using the X-ray structure geometries with the other functionals (or even with the PBE0 functional) does not give better agreement of the calculated to the experimental chemical shifts. That is, the P–P bond length in compound **8** is 2.147(6) Å by X-ray [67] and is 2.143 Å using the Latypov PBE0/6-31+G(d)/gas-phase optimization method. This optimized geometry gave a scaled NMR (PBE0/6-311G(2d,2p)/gas phase) of −147.3 ppm compared to the experimental chemical shift of −157.7 ppm. The P–P bond length was 2.175 Å and 2.156 Å using the B3LYP and M06-2X optimizations, respectively, giving scaled chemical shifts of −115 to −120 ppm for the former and −116 and −128 ppm for the latter optimization. Calculation of the scaled chemical shifts using the X-ray geometry gave −133.5 and −121.8 ppm for the B3LYP and M06-2X functionals, so clearly the more accurate M06-2X bond length did not give better agreement with the experimental chemical shift. A similar bond length comparison was seen for compounds **9** and **10**, where the PBE0 functional gave P–P bond lengths that were closest to the X-ray geometries, but using the X-ray geometry for the B3LYP and M06-2X NMR functionals gave some improved and some far worse calculated NMR chemical shifts. Clearly the problems with both the B3LYP and M06-2X NMR calculations are with the functionals themselves, not the geometries.

In order to test if the P–C multiple bond effect was reproducible, we optimized 3,4-dimethylphosphabenzene (a training set compound chosen by Latypov, with experimental and scaled chemical shifts of 187.9 [71] and 197.4 [37] ppm) and found the scaled chemical shift was 175.1 ppm for the M06-2X (optimization) and B3LYP (6-311+G(2d,p) NMR) combination, but was 225.2 ppm when the M06-2X functional was used for both the optimization and NMR. As will be seen below, this chemical shift calculation failure was seen in all the subsequent cases we examined that have P–C multiple bonding when the M06-2X functional was used for the NMR calculation.

The results in Table 2 show that the Latypov functionals, used without any solvation and with the Latypov training set for scaling, gave the best fit for these 10 compounds (MAD/RMSD = 5.3/7.0 ppm). Use only of our different training set gave a significantly worse fit (MAD/RMSD = 9.1/11.0 ppm), showing that Jensen’s point that choice of functional resembles data fitting [8] can also be applied to choice of training set. Following removal of the multiple bonded P–C chemical shifts, the Latypov scaling was still best (and essentially unchanged) but the M06-2X NMR method was closer (MAD/RMSD = 7.1/9.5 ppm) and was better than the Latypov method scaled with the alternate training set (MAD/RMSD = 7.8/9.3 ppm).

**Figure 5:**
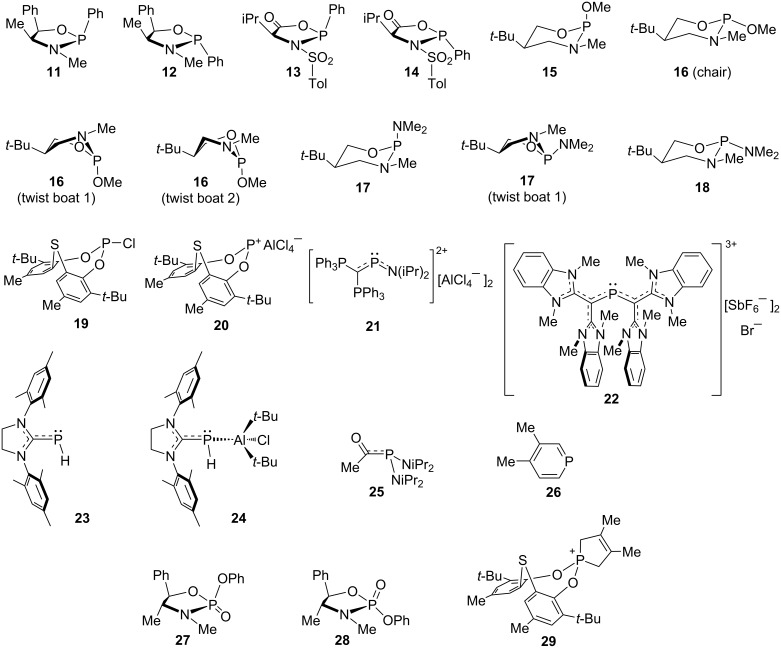
Stereoisomers and unusual phosphorus compounds used for chemical shift calculations.

**5. Calculated ^31^P NMR chemical shifts for stereoisomers and unusual structures.** We next chose our own set of phosphorus compounds (Figure 5, **11**–**29**; for simplicity compounds **30**–**34[O]** discussed later are included in the MAD/RMSD values here). This was done to to determine if the calculation and scaling would be accurate enough to distinguish stereoisomers via chemical shifts rather than coupling constants [72–73] and provide confirmation of unusual structures and chemical shifts, and with the further stipulation that multiple P–C bonding would likely give inaccurate M06-2X NMR calculations (Table 3; results with the IGLO-III basis set are included in Supporting Information File 1, Tables S6–S9, and as expected gave higher MAD/RMSD values). The M06-2X (optimization) and B3LYP (NMR) functional combination gave the best MAD/RMSD, and the relatively low RMSD is consistent with the fact that there were no glaring discrepancies in experimental and calculated chemical shifts, both for the initial group of **1a**–**10** (Table 2) and the new group of **11**–**34[O]**. As expected the M06-2X functional gave the highest values when it was used for the NMR calculation due to the presence of compounds with P–C multiple bonds. In all cases for the sets of stereoisomers (i.e., **11**/**12**, **13**/**14**, **15**/**16**, **17**/**18**, and **27**/**28**), the correct order of calculated upfield and downfield shifts was observed, although the calculated difference between the *cis* and *trans* isomers tended to be larger than the experimental difference for the trivalent compounds. Conformational differences play a role here, particularly for the six-membered rings in compounds **15**–**18**, where the twist boat conformations can be the major isomers in solution [74] (see Table 3 footnotes and Supporting Information File 1 for details), although care was taken to find all important conformations to be included in the NMR calculations.

**Table 3:** Experimental^a^ and scaled^b 31^P NMR chemical shifts^c^ for the compounds shown in Figure 5, Figure 6, and Figure 7.

Optimization functional:		B3LYP	M06-2X	M06-2X	PBE0
NMR functional:		B3LYP	B3LYP	M06-2X	PBE0^d^

Compound	Exp.^e^				

**11** ^f^	152.9 [75]	156.9	153.3	152.1	166.5
**12** ^ f^	139.3 [75]	142.0	138.5	139.3	150.6
**13**	133.0 [76]	133.8	125.4	125.0	128.8
**14** ^f^	136.7 [76]	144.8	128.8	132.0	138.8
**15** ^f^	132.2[74]	129.3	126.4	124.7	137.5
**16** ^f^	138.5 [74]	142.1	137.2	134.8	150.3
**17** ^f^	125.8 [74]	128.6	126.2	124.9	133.7
**18** ^f^	145.3 [74]	146.7	144.5	142.3	152.8
**19** ^g^	169 [77]	181.3	178.9	172.3	186.0
**20** ^g^	153 [77]	147.8	146.4	148.7	169.2
**21** ^h^	355.7 [78]	350.9	355.2	**398.1**	372.4
**21** (Ph_3_*P*)	26.2	20.7	19.2	23.8	20.9
**22** ^i^	302 [79]	310.4	299.7	**350.9**	306.4^j^
**23**	−127.2 [80]	−125.7	−124.8	**−107.0**	−130.0
**24**	−151.0 [80]	−129.0	−131.2	**−120.6**	−140.3
**25**	63.5 [81]	64.7	68.7	**73.6**	63.6
**26**	187.9 [71]	181.7	182.1	**224.9**	197.3
**27** ^f^	13.9 [82]	11.0	12.3	17.7	19.6
**28**	16.0 [82]	14.2	15.4	20.6	22.7
**29** ^g^	93 [77]	80.8	97.1	104.6	94.1
*anti*-**30**	24.2 [83]	34.8	25.2	17.9	18.8
*syn*-**30**^f^	11.3 [83]	23.5	15.6	9.1	7.8
*anti*-**30[O]**	54.1 [83]	44.8	42.1	45.4	44.7
*syn*-**30[O]**^f^	61.8 [83]	49.7	46.3	49.3	50.1
*anti*-**31**	−24.4	−12.1	−29.2	−27.9	−31.2
*syn*-**31**	−21.8	−0.9	−21.4	−19.6	−22.2
**32** ^f^	−181	**−59.2**	−169.3	−180.1	**−119.8**
**33**	−79	−78.8	−74.7	−72.1	−90.3
**34**	−14	−3.6	−11.2	−10.0	−20.4
**33[O]**	38	27.2	30.3	36.3	24.6
**34[O]**	26	12.6	13.7	20.8	11.0
**11**–**34[O]**					
MAD/RMSD^k^		11.1/23.7	***5.4/7.3***	9.7/15.8	9.8/14.4
**1a**–**34[O]**					
MAD/RMSD		11.9/21.8	***7.1/9.9***	10.3/16.3	8.2/12.3
**2–7**, **9–20**, **27–34[O]**^l^					
MAD/RMSD		12.0/22.8	6.4/8.5	***5.4/7.1***	8.7/13.1

^a,b,c,d^See notes a–d for Table 2; NMR basis sets and solvation were 6-311+G(2d,p) and CHCl_3_ except for PBE0 (6-311G(2d,2p) and gas phase). ^e^Compounds **11**, **12**, and **30**–**31** were optimized and the NMR spectra calculated in toluene, **13**, **14**, **19**, **20**, **25**, **26**, **29**, and **32**–**34[O]** in chloroform, **15**–**18**, **23**, **24**, **27**, and **28** in benzene, **21** in dichloromethane, and **22** in acetonitrile. ^f^Major conformations shown in Figures 5–7 but compounds **11**, **12**, **14**, **16**–**18**, **27**, *syn*-**30**, *syn*-**30[O]**, and **32** exhibit multiple conformations; for **16** twist-boat 1 and twist-boat 2 are significant, and for **17** the chair and twist boat 1 are significant; for **15** only the chair was significant (Table S7, Supporting Information File 1). ^g^Optimizations and NMR calculations were carried out on the compounds without the methyl groups *para* to the oxygen atoms due to problems with convergence because of methyl rotation; no significant chemical shift differences were seen. ^h^BF_4_¯ rather than the actual AlCl_4_¯ ions were used in the calculations to minimize the size of the calculation. ^i^PF_6_¯ and Cl¯ ions rather than the actual SbF_6_¯ and Br¯ ions were used in the calculations to minimize the size of the calculation. ^j^In the absence of solvation the trication structure could not be optimized in the presence of anions so this calculation is for the trication alone. ^k^See note a in Table 1. ^l^Combined MAD/RMSD for all compounds in Table 2 and Table 3 with no multiple P–C bonds.

Unusual structures such as phosphenium cation **20**, having a chemical shift upfield of the trivalent chloride **19**, contrary to expectation where the cation is typically 100 ppm downfield of the corresponding chloride [77,84–86], were confirmed by each of the calculation methods, as were the remarkably downfield shifts for the novel di- and trications **21** and **22**, for which even drawing suitable resonance structures is a challenge. Two phosphinidenes (**23**, **24)**, i.e., carbene analogues with potentially anionic phosphorus atoms, have remarkably upfield chemical shifts that are once again confirmed by the calculated values. Both of the P–C bond lengths are indicative of single bond character albeit relatively short (both the X-ray [80] and DFT structures), although the large downfield deviations for the M06-2X NMR calculation suggest multiple P–C bonding – perhaps a novel use of this DFT failure.

The acylphosphonodiamidite **25** is another novel structure confirmed by the chemical shift calculation, although the largest deviation was seen for the M06-2X NMR calculation, again perhaps suggesting multiple P–C bonding. The case for multiple P–C bonding in compound **25** is supported by the amide-like CO infrared stretching frequency of 1654 cm^−1^. This compound further warrants mention since one might suppose that the carbonyl carbon atom would be found near 170 ppm in the ^13^C NMR spectrum by analogy to amides, but was instead observed at 228 ppm and confirmed by a calculated (unscaled) value of 242 ppm [81]! Such a result demonstrates the value of DFT calculations for structures not having any experimental NMR precedent.

We include here phosphabenzene **26** [71], which as noted above was used as part of the Latypov training set, for these four methods. As expected the calculations confirm the failure of only the M06-2X NMR method for compounds with multiple P–C bond character.

**Figure 6:**
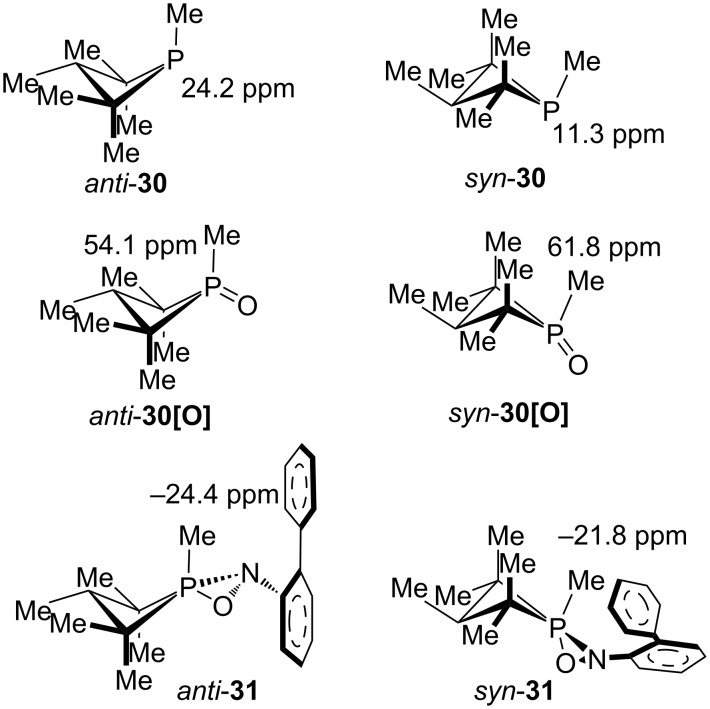
Phosphorus-catalyzed oxygen transfer reaction intermediates.

We finish with two challenging examples, one a relatively recent report by the Radosevich group of a novel catalytic oxygen transfer reaction involving four-membered ring phosphorus compounds [83], and one involving a 57-year old report by the Katz group of the first characterized three-membered ring phosphorus heterocycle [87]. The four-membered ring phosphetanes and proposed intermediate structures (**30**, **30[O]**, **31**, Figure 6) provide examples of novel structures [83] where stereochemistry is also confirmed by calculations, even for the challenging intermediates *anti* and *syn*-**31**. The authors of that study chose to optimize the structures using the M06-2X functional with the 6-311++G(d,p) basis set, but we found that the smaller 6-31+G(d,p) basis set that we had been using was adequate. Each of the methods allowed the stereoisomers to be distinguished, although the optimization and NMR with the B3LYP functional was much worse (MAD/RMSD = 12.9/13.4 ppm) while the M06-2X optimization and NMR was the best (MAD/RMSD = 5.9/7.0 ppm). We also note that the calculated chemical shifts reported in the phosphetane study [83] were referenced to *anti*-**30** rather than H_3_PO_4_, while here all the values were calculated with reference to H_3_PO_4_, before being scaled.

**Figure 7:**
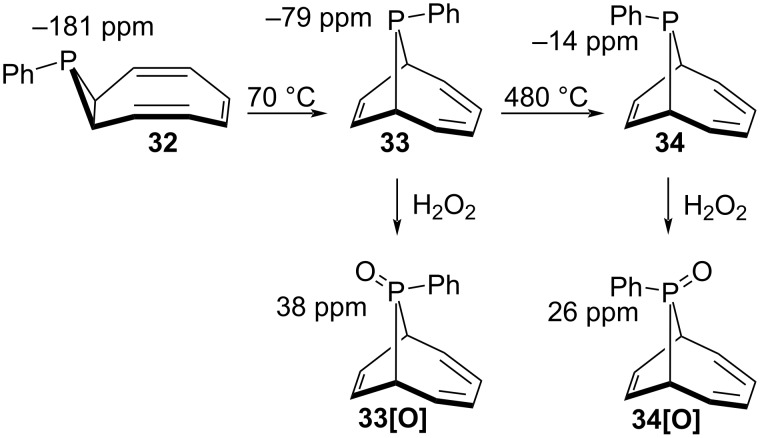
Phosphirane reactions.

As the final example, Katz reported in 1966 [87] the reaction of cyclooctatetraene dianion with PhPCl_2_ to give a single phosphine product **32** having an unusually high field ^31^P NMR chemical shift of −181 ppm. Compound **32** underwent a stereospecific thermal [1,5]-sigmatropic rearrangement to bicyclic **33** exhibiting a ^31^P NMR chemical shift of −79 ppm. Pyrolysis of **33** at 480 °C gave isomeric **34** having a ^31^P NMR chemical shift of −14 ppm, while H_2_O_2_ oxidation of compounds **33** and **34** gave the corresponding phosphine oxides **33[O]** and **34[O]** with chemical shifts of 38 and 26 ppm, respectively (Figure 7). Identification of the isomers by ^31^P NMR would represent a nice example of the utility of the calculations described here. Results for **33**, **34**, **33[O]**, and **34[O]** all confirmed the 1966 identifications (although the deviations are smallest for the two M06-2X optimization methods), but the ^31^P NMR of the compound of primary interest, **32**, differed by 61 ppm from the calculated value using the Latypov method and 122 ppm using the B3LYP optimization, but gave excellent agreement using the M06-2X optimization, especially with the M06-2X NMR calculation (Table 3)! The X-ray structure of **32** was reported in 2004 [88–89] so the identification is correct, and provides a surprisingly extreme example of how these DFT functionals can differ. Comparison of bond lengths showed that this might be due to sensitivity of the DFT chemical shift to bond lengths. The three-membered phosphorus ring in the X-ray structure exhibited C–C and average C–P bond lengths of 1.495(2) and 1.869(5) Å, while the values for the M06-2X, Latypov, and B3LYP optimizations were 1.495/1.869, 1.488/1.882, and 1.489/1.908 Å, respectively. This suggested that the virtually exact match of the M06-2X optimization with the X-ray structure contributed to the agreement of the NMR calculation with the experimental chemical shift. Consistent with this, calculation of the NMR chemical shifts using the X-ray structure geometry also gave near perfect fits to the experimental for the B3LYP and PBE0 functionals. However as noted above use of the X-ray geometries of **8**–**10** with the B3LYP and M06-2X NMR functionals showed that the poor agreement of the NMR chemical shift calculations was due to the NMR functionals, not the geometries used.

**6. The search for a failure-free functional.** Looking at the complete collection of compounds evaluated (Table 2 and Table 3), the best MAD/RMSD (7.1/9.9 ppm) for **1a**–**34[O]** was seen for the M06-2X optimization and B3LYP NMR. As seen in the last row of Table 3, elimination of the failures due to compounds with P–C multiple bonding character gave the best MAD/RMSD = 5.4/7.1 ppm for optimization and NMR calculations both with the M06-2X functional. Clearly we are looking at a better “fitting” functional as described by Jensen [8], but what truly distinguishes these ^31^P NMR results from the better-studied ^1^H and ^13^C results is the existence of the ^31^P NMR failures.

The failures that we encountered included (1) the P–C multiple bonds for the M06-2X NMR calculations (i.e., **1a**), (2) *possibly* P–P bonding for both the M06-2X and B3LYP NMR calculations (i.e., **8** and to a lesser extent **9** and **10**), and (3) the phosphirane **32** for the B3LYP/B3LYP method and the PBE0 method of Latypov. We therefore set out to look at these outliers with recently recommended functionals rather than the widely-used functionals [15,90] that we have already tried (i.e., B3LYP, PBE0, M06-2X). In this way we might hope to see if this could provide a short-cut to find the best NMR functional, that is, one that might give the best fit with the least scatter (i.e., the lowest MAD), but also with none of the failures.

A recent review assessed 200 density functionals [91] and assigned them to the five rungs of “Jacob’s ladder” [92–95]. In principle these 200 functionals could be evaluated for our ^31^P NMR chemical shift problem, but in that same review Mardirossian and Head-Gordon noted that while DFT methods have been successfully used for chemical shift calculations, magnetic properties are not included in conventional functional development nor in the energy benchmarks that are used. Instead, we looked at recent work, mostly on ^13^C NMR spectroscopy, for recommendations [7,90–91,96–98].

Results are collected in Table 4 for the chemical shifts for compounds **1a**, **8**, and **32**, scaled using only the first and last points for the trivalent series, PCl_3_ and PH_3_ (see Computational and NMR Details below). Comparison of the two-point scaling values for entries 1–4 in Table 4 with the full linear regression for scaling in Table 2 and Table 3 validates this short-cut. The GIAO NMR method was used for all of the calculations described to this point, as well as for the commonly recommended methods for ^1^H and ^13^C calculations [15]. However, the first method to be tried for this group of three compounds utilized the alternative CSGT NMR method recommended by Iron [7] with the TPSSTPSS [92,99–100] functional for the NMR calculation (albeit we used the computationally less intensive basis sets and the M06-2X optimization method already employed). As seen in entry 5 (Table 4) the GIAO method gave no improvement over entries 3 and 4, but use of the recommended CSGT method did give a reduction in the MAD (Table 4, entry 6). Optimization with the TPSSTPSS functional followed by the TPSSTPSS/CSGT NMR (Table 4, entry 7) failed, especially for compound **32**. Iron further found that long-range corrected (LC) functionals all out-performed the non-corrected functionals, so this was tested as seen by entries 8 and 9 (Table 4) for both the GIAO and CSGT calculation. The GIAO entry was only slightly better while the CSGT entry was worse, but more interestingly, both failed for the P–C multiple-bonded case, giving chemical shifts over 80 ppm downfield from the non-long-range corrected calculations for P_B_ in **1a** (Table 4, entries 5 and 6). The related PBETPSS functionals recommended by Modrzejewski et al. [98] exhibited the identical downfield failure for P–C multiple bonding (Table 4, entries 10–13) upon adding in the LC calculation. An obvious hypothesis for the M06-2X P-C multiple bonding NMR failure therefore was that this functional employs too much long-range correction, and Truhlar has described it as a medium-range method [57,101]. We therefore tried the local version of the M06-2X functional, namely the M06-L functional [102], for the NMR calculation (Table 4, entries 14–16), and were rewarded with the best MAD for these three compounds when using the M06-2X optimization and the M06-L/GIAO NMR calculation (Table 4, entry 14). Interestingly, this functional was *not* recommended in a recent study on calculation of solid-state chemical shifts for ^31^P NMR [103]. Use of the CSGT NMR method gave a higher MAD (Table 4, entry 15) and M06-L optimization gave a very poor result (entry 16). Use of the newer local M11-L functional [104] for the NMR however was much worse (Table 4, entries 17 and 18) due entirely to the poor fit of **1a** when using M06-2X optimization but all four chemical shifts were poor (even P_A_ in **1a**, used essentially as a control) using M11-L optimization. Two fourth rung [90] functionals, M11 and MN12-SX [105–106], also failed to give any improvement (Table 4, entries 19 and 20), and these were also worse than the older fourth rung [90] functional M06-2X. Grimme’s D3 dispersion correction has been recommended for general use as it increases the accuracy of many calculations [97–98], particularly with the BLYP functional (Table 4, entry 21) [97], but for the cases we tried no improvement was seen (Table 4, entries 21–24). Last, Modrzejewski et al. [98] noted that Head-Gordon’s ωB97X-D, a long-range corrected range-separated functional with dispersion correction [96], was also found to be highly accurate, and it was the most recently developed of the five representative functionals chosen by Jensen for evaluation [8]. Somewhat surprisingly, given the long-range correction that we previously found had resulted in the failure of P–C multiple bonding calculation, this functional (Table 4, entries 25 and 26) gave two of the best MAD values, with optimization either by the M06-2X or ωB97X-D functionals. Last, given the success of many of the M06-2X optimizations here and the Latypov PBE0 method, it seemed appropriate to test that (Table 4, entry 27). Here the PBE0 NMR calculation was carried out in the same way as entries 2–26, with CHCl_3_ solvation and the 6-311+G(2d,p) basis set, and as seen it gave one of the best results.

**Table 4:** Scaled (using PH_3_ and PCl_3_) chemical shifts for the “failures” **1a**, **8**, and **32** for various optimization and NMR functionals.

Entry	Optimization	NMR^a^	**1a** P_A_	**1a** P_B_	**8** P_B_	**32**	MAD^b^

	**experimental**		**39.6**	**297.6**	**−157.7**	**−181**	
1	PBE0 (Latypov)	PBE0 (Latypov)	37.2	282.5	−139.6	−112.7	33.9
2	B3LYP	B3LYP	42.1	278.4	−114.2	−59.2	61.5
3	M06-2X	B3LYP	40.3	272.2	−125.7	−167.9	23.5
4	M06-2X	M06-2X	56.2	336.0	−115.1	−175.2	28.9
5	M06-2X	TPSSTPSS	36.9	256.4	−135.8	−164.1	26.7
**6**	**M06-2X**	TPSSTPSS*	**34.3**	**260.3**	**−148.1**	**−173.3**	**18.2**
7	TPSSTPSS	TPSSTPSS*	31.8	259.0	−132.9	−20.9	74.5
8	M06-2X	LC-TPSSTPSS	38.1	339.4	−132.4	−184.8	23.6
9	M06-2X	LC-TPSSTPSS*	33.4	345.0	−149.8	−199.0	24.4
10	M06-2X	PBETPSS	44.1	260.1	−124.5	−152.2	33.1
11	M06-2X	PBETPSS*	41.4	264.4	−139.0	−162.3	23.5
12	M06-2X	LC-PBETPSS	40.9	342.1	−127.4	−178.4	25.8
13	M06-2X	LC-PBETPSS*	35.0	348.7	−147.5	−195.4	25.3
**14**	**M06-2X**	**M06-L**	**36.5**	**274.4**	**−156.7**	**−180.9**	**8.1**
15	M06-2X	M06-L*	32.1	278.1	−171.5	−190.8	14.4
16	M06-L	M06-L	27.4	257.4	−161.4	−61.5	54.5
17	M06-2X	M11-L	23.6	231.5	−156.5	−181.8	22.7
18	M11-L	M11-L	7.8	212.3	−172.6	−70.1	70.4
19	M11	M11	43.8	339.8	−124.9	−194.4	29.5
20	MN12-SX	MN12-SX	27.2	274.6	−154.9	−145.6	20.4
21	BLYP-D3	BLYP-D3	34.4	242.3	−100.1	−7.6	95.4
22	M06-2X-D3	M06-2X-D3	56.1	336.0	−115.0	−175.2	29.0
23	M06-L-D3	M06-L-D3	27.3	257.4	−161.3	−61.5	54.4
24	TPSSTPSS-D3	TPSSTPSS-D3*	31.8	259.6	−140.5	−23.1	71.0
**25**	**M06-2X**	**ωB97X-D**	**39.3**	**293.1**	**−132.8**	**−177.7**	**10.9**
**26**	**ωB97X-D**	**ωB97X-D**	**38.0**	**292.8**	**−136.9**	**−174.2**	**10.8**
**27**	**M06-2X**	**PBE0**	**43.3**	**285.1**	**−133.2**	**−169.0**	**16.3**

^a^CSGT method indicated by *; all others are GIAO. ^b^MAD for **1a** isomer A P_B_, **8** P_B_, and **32** isomer A; scaled chemical shifts for **1a** isomer A P_A_** (**which has a P–C single bond) are shown for comparison but are not included in the MAD calculation, and scaled chemical shifts for **8** P_A_ are not included because these are correctly calculated by all methods previously (Table 2).

**Figure 8:**
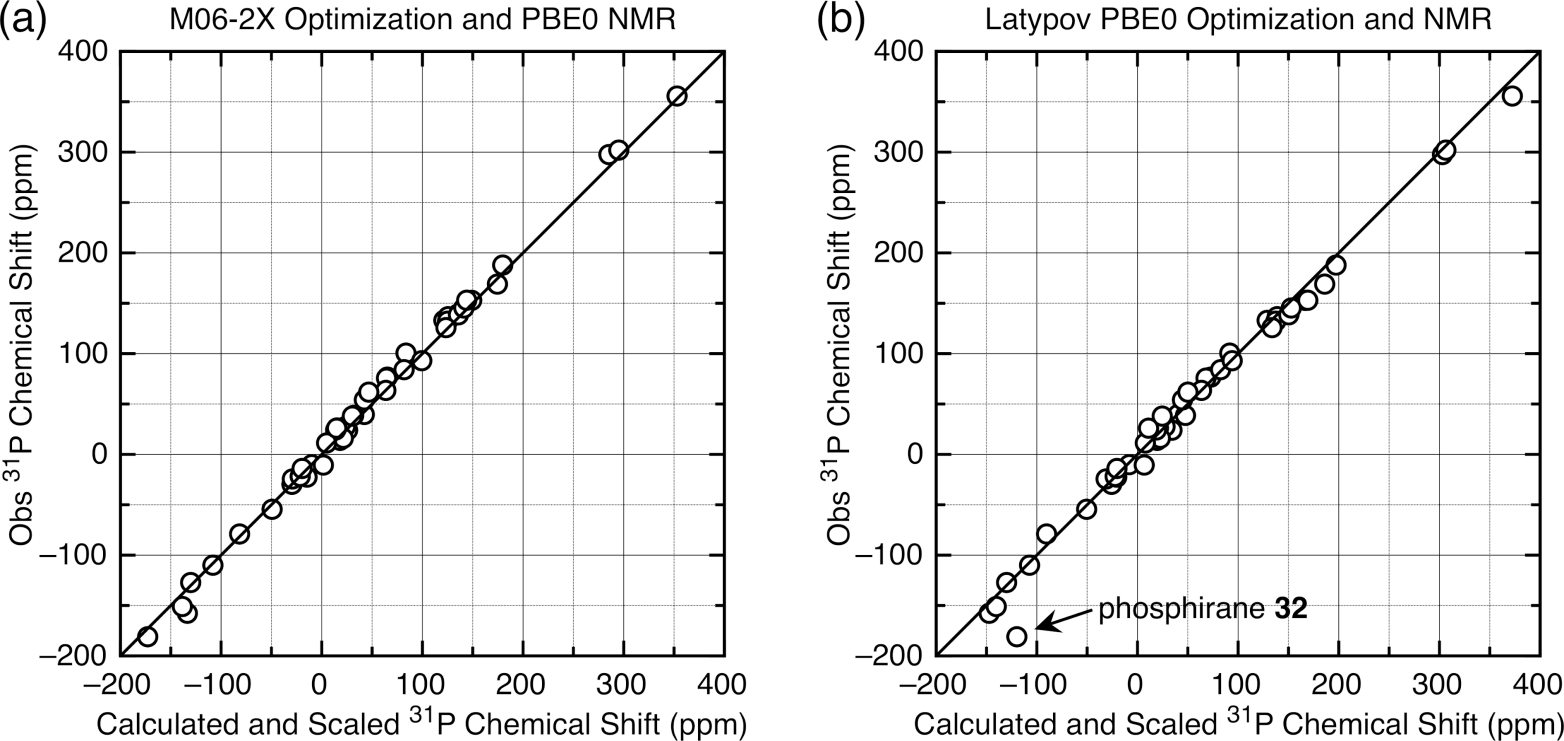
(a) Plot of experimental vs scaled chemical shifts derived from the tri- and tetracoordinate phosphorus training set compounds (M06-2X optimization and PBE0 NMR (6-311+G(2d,p) basis set) referenced to H_3_PO_4_, IEF-PCM CHCl_3_ solvation; see Supporting Information File 1, Table S17 for values). The line drawn (slope = 1, intercept = 0) is the perfect fit line. (b) Plot of experimental vs scaled chemical shifts derived from the Latypov training set compounds (PBE0 optimization and PBE0 NMR (6-311G(2d,2p) basis set) referenced to H_3_PO_4_, no solvation; see Supporting Information File 1, Table S9 for values). The line drawn is the perfect fit line.

The optimization/NMR methods that gave the lowest MAD values in Table 4 (entries 6, 14, 25–27, apart from entry 15 which was worse than the related entry 14) were then used for the full set of tri- and tetracoordinate compounds for scaling, and then the full set of test compounds **1a**–**34[O]**; MAD/RMSD results are listed in Table 5 (see Supporting Information File 1, Tables S7 and S10–S17 for all data) along with comparisons to the four best prior methods in Table 4 (entries 1, 3, and 4). As can be seen by examination of the results for the training set of tri- and tetracoordinate compounds in the first column, two of the new combinations, namely the M06-2X/PBE0 and the M06-2X/ωB97x-D functionals for optimization and NMR calculations, were among the best for the MAD/RMSD values in Table 1 and Table 5. These two also exhibited the lowest MAD/RMSD values of 6.9/8.5 and 6.8/9.1 ppm, respectively, for the full set of test compounds **1a**–**34[O]**. The higher RMSD for the M06-2X/ωB97x-D combination is due to the relatively large number of scaled chemical shifts that differ by 18–26 ppm from the experimental chemical shifts, while the M06-2X/PBE0 combination exhibits only one of those large chemical shift deviations. The next best M06-2X/M06-L combination (MSD/RMSD = 7.5/9.3 ppm) exhibits four such large deviations, although it was the best for the troublesome P_B_ of **8**. In fact, the only other combination that has only one large deviation is Latypov’s gas-phase PBE0/PBE0 calculation, and as described above that is the phosphirane **32**, with a scaled chemical shift calculation of −120 ppm compared to the experimental value of −181 ppm. Without that one data point, the MAD/RMSD drops from 8.2/12.3 ppm to 7.1/8.6 ppm, which is one of the best results. As seen in Figure 8, plotting the experimental chemical shifts against the scaled calculated values for **1a**–**34[O]** for the M06-2X/PBE0 and the Latypov PBE0/PBE0 combinations each gives a set of values very close to the desired straight line with a slope of 1 and an intercept of 0, except for the one failure for compound **32** as shown; by inspection it can be seen that the Latypov plot does exhibit more scatter about the perfect fit line, so the higher MAD/RMSD for the points other than that for **32** makes sense.

**Table 5:** Comparison of the best^a^ functionals for **1a**–**34[O]**.

	MAD/RMSD (ppm)					
	
Opt/NMR^b^	P3 and P4 training set	**1a**–**34[O]**	P–P: **8**–**10**	**1a–7**, **11**–**34[O]**	P=C: **1a**, **8**P_B_, **21–26**	**2**–**8**P_A_, **9–20**, **27**–**34[O]**

M06-2X/B3LYP	**6.5/8.0**	**7.1/9.9**	15.6/18.5	**5.4/7.1**	9.7/14.0	6.4/8.5
M06-2X/ M06-2X	**4.1/5.7**	10.3/16.3	13.7/18.8	9.7/15.7	29.1/32.8	**5.4/7.1**
PBE0/PBE0^c^	7.8/9.7	**8.2/12.3** ^d^	**6.9/8.5**	8.5/12.9	**6.6/8.2**	8.7/13.1
M06-2X/M06-L	7.2/8.8	7.5/9.3	9.3/10.7	7.2/9.0	8.2/9.6	7.4/9.2
M06-2X/TPSSTPSS*^e^	6.7/8.2	8.7/12.1	11.8/13.1	8.0/11.8	17.2/20.7	6.4/8.4
M06-2X/ωB97x-D	**5.9/7.3**	**6.8/9.1**	11.6/13.8	**5.8/7.9**	10.4/13.9	**5.9/7.4**
ωB97x-D/ωB97x-D	6.1/7.5	7.4/9.4	11.0/13.3	6.6/8.4	9.8/12.6	6.7/8.3
M06-2X/PBE0	**5.8/7.1**	**6.9/8.5**	10.8/13.0	**6.1/7.2**	8.4/10.7	6.5/7.7

^a^Best results are in bold. ^b^Functionals for optimization (6-31+G(d,p) basis set) and NMR (6-311+G(2d,p) basis set), both with CHCl_3_ solvation (IEF-PCM) except as noted. ^c^Optimization and NMR following Latypov, gas phase and 6-31+G(d) and 6-311G(2d,2p) basis sets, respectively. ^d^Without **32**, MAD/RMSD = **7.1/8.6** ppm. ^e^CSGT NMR method; all others are GIAO.

We conclude this section by examining separation of the P–P-bonded compounds or the P–C multiple-bonded compounds. Separating out first the P–P-bonded compounds **8**–**10** showed that the Latypov PBE0/PBE0 method was by far the best for those compounds, with the M06-2X/M06-L method that gave the best calculated chemical shift for P_B_ in **8** (−148.0 vs −157.7 ppm for the experimental value) the only other method that was close; the M06-2X/B3LYP method was the worst. When compiling the MAD/RMSD values for the remaining non-P–P-bonded compounds **1a**–**7** and **11**–**34[O]**, the overall results were somewhat better and the M06-2X/B3LYP method was the best. On the other hand, when the P–C multiple-bonded compounds **1a**, **8**(P_B_), and **21**–**26** were separated out, the failure of the M06-2X/M06-2X was confirmed (MAD/RMSD = 29.1/32.8 ppm), and surprisingly the M06-2X/TPSSTPSS* method also failed (MAD/RMSD = 17.2/20.7 ppm) – surprising since this had been selected in the screening method. The Latypov PBE0/PBE0 method was once again the best for this subset, with the M06-2X/M06-L and M06-2X/PBE0 methods the only others that were close. When these multiple-bonded compounds were eliminated from the test set and the MAD/RMSD values compiled for **2**–**8**(P_A_), **9**–**20**, and **27**–**34[O]**, however, the M06-2X/M06-2X was best (MAD/RMSD = 5.4/7.1 ppm).

## Conclusion

We have developed a method for accurate calculation of ^31^P NMR chemical shifts using a training set of well-known tri- and tetracoordinate phosphorus compounds that allows scaling of the readily accessible DFT chemical shifts. The present method follows established norms [15] of optimization of compound geometries in solution and weighting of the calculated chemical shifts on the basis of calculated equilibrium ratios in solutions of the different conformers. We compare this to the previously reported method described by Latypov [37], which uses a somewhat eclectic mix of unusual phosphorus compounds, some of which have questionable chemical shifts, and that are optimized using gas-phase calculations. The Latypov method, using the PBE0 functional for both optimization of compound geometry and chemical shift calculation, was found to be superior for compounds that contain P–P bonds and P–C multiple bonds. Optimization with the M06-2X functional was found to be superior for all compound types *except* the P–P-bonded compounds when the B3LYP functional was used for the chemical shift calculation, and the M06-2X functional was also found to be superior for all compound types *except* the P–C multiple-bonded compounds when the M06-2X functional was used for the chemical shift calculation.

One of the goals of this work was that the calculated chemical shifts should be sufficiently accurate to distinguish stereoisomers and confirm structures of unusual compounds. In fact, the methods were able to correctly reproduce the relative chemical shifts of stereoisomers that differed by as little as 3 ppm. However, for the unusual compounds, some combinations of functionals failed for confirmation of structures that contained multiple P–C bonding, P–P bonding, and for a 3-membered phosphirane ring. For instance, use of the M06-2X functional for both optimization and NMR calculations gave large downfield shifts of 30–49 ppm from the experimental values if there was any P–C multiple-bonding character, and B3LYP and PBE0 optimization led to downfield shifts of 61–124 ppm from the experimental value for the phosphirane ring. The P–P bonding failure is less clear, with downfield shift failures of 20–47 ppm for P_B_ in **8** for all pairs of functionals *except* the Latypov PBE0/PBE0 method and the M06-2X/M06-L combination, both of which gave downfield shifts of 10 ppm. A screening method was used to allow rapid calculations of scaled values to detect methods that would avoid these three failures, in hopes that this might lead to better overall methods. The results show that the strategy of screening methods using the “failures” did in fact lead to improvements in calculations, including a potential method using a localized version (M06-L) of the M06-2X functional for the NMR, and the two best methods for general use: the best combination involved optimization using the M06-2X functional with NMR calculations using the PBE0 functional, and use of the ωB97x-D functional for the NMR calculations was a close second. For compounds without P–P bonds, the M06-2X/B3LYP combination, on the basis of fairly limited data, can be used, and for compounds without P–C multiple bonds, the M06-2X/M06-2X combination is a good choice. For compounds with those functionalities, the Latypov method is best. Given the unexpected failures noted, for compounds with novel structures not covered here, more than one method should be used.

On the interesting question posed by Jensen [8] on whether the search for the “best” functionals and basis sets for chemical shift scaling is an exercise in data fitting, we note that unlike the case for ^1^H and ^13^C chemical shifts, ^31^P chemical shifts must depend on a far more variable collection of phosphorus bond lengths and geometries. The functionals that give the most accurate bond lengths to phosphorus might give the best calculated chemical shifts, but changing the bond lengths to the correct lengths using the X-ray geometries does not necessarily then yield the correct chemical shifts as described in detail for **8** and in brief for **9** and **10**, and of course this is not a viable strategy to confirm novel and unknown structures. In the failure of the M06-2X functional with multiple bonding, the cause is clearly not due to failure to give proper bond lengths, as chemical shifts using the B3LYP functional are reliable using the identical M06-2X optimized structures. The decrease in scatter using the M06-2X optimization, however, can be considered as improved data fitting as described by Jensen [8], but the failures, including P–P bonding, P–C multiple bonding, and, for the Latypov optimization, the three-membered phosphirane ring, are examples of functionals that clearly do not give a fit to experimental chemical shifts due to serendipitous cancelling of errors, but rather must have some fundamental flaw for those structural types. Reports of comparisons of functionals do not describe such failures [7–8,107], so the observation here of a closer fit of calculated to experimental chemical shifts for most compounds using the M06-2X functional for the NMR calculation, but with major significant failures for some structural types, is unique and so must be taken into account when looking at novel structures.

Overall these scaling methods were shown to provide excellent support for confirmation of stereochemistry and of solution structures of unusual phosphorus compounds, and should be considered part of standard practice for DFT calculation of ^31^P NMR chemical shifts of novel compounds and those with unknown stereochemistry. Future work, however, should focus on the outlier compounds described here, whose unusual bonding gives rise to increased sensitivity to chemical shift calculations, and may help to more rapidly uncover which functionals are best for both geometry optimization and NMR chemical shift calculation.

## Computational and NMR Details

For the trivalent phosphorus compounds, NMR chemical shifts (referenced to external 85% H_3_PO_4_ at 0.00 ppm, positive values downfield) were measured in CDCl_3_ on a 400 MHz Bruker spectrometer for PPh_3_ (−5.28 ppm), P(OMe)_3_ (141.41 ppm), and PCl_3_ (219.79 ppm) or were taken from the literature: PH_3_ (−238 ppm, liquid sample at −90 °C [44] and at room temperature in CCl_4_ [52]), PMeH_2_ (−163.5 ppm, liquid sample [44]), PMe_2_H (−98.5 ppm, liquid sample [44]), PMe_3_ (−61.58 ppm in CDCl_3_ [50]), MeOP(OCH_2_CH_2_O) (133.3 ppm, in CDCl_3_ [108]). For PCl_3_ the experimental geometry was used for the NMR calculations (P–Cl = 2.043 Å, 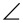
(ClPCl) = 100.1°) [56] rather than the DFT optimized geometries (B3LYP: P–Cl = 2.096 Å, 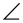
(ClPCl) = 100.9°; M06-2X: P–Cl = 2.069 Å, 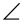
(ClPCl) = 100.0°; PBE0: P–Cl = 2.070 Å, 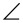
(ClPCl) = 100.7°; PBE0/6-31+G(d)/gas phase: P–Cl = 2.066 Å, 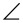
(ClPCl) = 101.0°).

For the tetracoordinate phosphorus compounds, NMR chemical shifts (referenced to external 85% H_3_PO_4_ at 0.00 ppm, positive values downfield) were measured in CDCl_3_ on a 400 MHz Bruker spectrometer for (iPrO)_2_P(O)H (4.54 ppm), Ph_4_P^+^ Br^−^ (23.17 ppm), and (iPrO)_2_P(O)Me (28.61 ppm) or were taken from the literature: PH_4_^+^ BF_4_^−^ (−105.3 ppm in CH_3_OH/CH_3_OD solution [109–110]), (PhO)_4_P^+^ PF_6_^−^ (−28.0 ppm in CH_3_CN solution [111]), O=***P*(**OCH_2_)_3_P=O (−18.1 ppm in DMSO solution [112]), O=P**(**OCH_2_)_3_***P***=O (6.4 ppm in DMSO solution [112]), (MeO)_4_P^+^ BF_4_^−^ (1.9 ppm in CH_2_Cl_2_ solution [113]), (MeO)_2_P(O)H (11.3 ppm, liquid sample [45]), Me_4_P^+^ Br^−^ (25.1 ppm in DMSO solution [114]), Ph_3_P=O (29.10 ppm in CDCl_3_ solution [115]), (MeO)_2_P(O)Me (32.3 ppm, liquid sample [116]), Me_3_P=O (38.79 ppm in CDCl_3_ solution [115]), EtOP(O)Me_2_ (50.3 ppm, liquid sample [43]).

Chemical shifts for the phosphonium salts R_4_P^+^ (R = MeO, Me, Ph) require comment. In the case of (MeO)_4_P^+^ BF_4_^−^, initial reports gave the ^31^P NMR chemical shift as 51.5 ppm [117–118], while all of our initial calculations placed it near −3 ppm. Subsequent work found the chemical shift to be 1.9 ppm [113], in agreement with our calculated shift, and no explanation for the original report has been offered [113]. For both of the R_4_P^+^ (R = Me, Ph) salts, chemical shift data were readily available for the Br^−^ but not the Cl^−^ salts. Bromine is not included in the IGLO-III and pcS-2 basis sets, however, so in those cases the calculated chloride salt chemical shifts were substituted. For Me_4_P^+^, the other basis sets gave identical chemical shifts for the Cl^−^ and Br^−^ salts, but for the Ph_4_P^+^ salts the Br^−^ salts were on average 2.7(0.2) ppm upfield of the Cl^−^ salts; if this correction were made, the deviations for the IGLO-III and pcS-2 basis sets would have been further reduced. Given the small difference and the absence of a strong justification for the correction, the chloride calculations were used without change.

Calculations were carried out using Gaussian 09, Revision D.01 [55]. The IGLO-III and pcS-2 basis sets were taken from the Basis Set Exchange [70], and all other basis sets were taken from Gaussian. X-ray structure coordinates were used as a starting point for optimizations when available. Energy optimizations were all accompanied by vibrational frequency calculations to ensure that stationary points were minima (all vibrations positive), and to ensure that true stationary points were confirmed in the vibrational frequency calculation. For the relatively large molecules, it was often found that the best results (in particular convergence to a minimum) were obtained using the “nosymm” instruction, and an initial calculation of all force constants (“calcfc”), rather than using the tight convergence criterion. All optimizations for the tri- and tetracoordinate phosphorus compounds utilized the 6-31G+(d,p) basis set with the polarizable continuum model, IEF-PCM/CHCl_3_, except for the Latypov calculations, which used the 6-31+G(d) basis set and no solvation [37]. NMR calculations (GIAO) were then carried out on these optimized structures using the same solvation method (IEF-PCM/CHCl_3_) or for the Latypov calculations, with no solvation. The reference calculations on H_3_PO_4_ were carried out the same way, using water solvation, again except for the Latypov calculations where no solvation was included. For compounds **1a**–**34[O]** the same solvent used for the experimental NMR spectrum was used for each optimization and NMR calculation, again except for the Latypov calculation where no solvent was used. Keywords to run M06-2X calculations were m062x and integral=ultrafine, and for PBE0 pbe1pbe. For compounds where multiple optimized minima were found, energies were taken from the vibrational calculation (sum of electronic and thermal free energies) and used to calculate the relative amount of each conformation present at 298.15 K or at the temperature of the literature NMR when available [59], and the energy-weighted NMR chemical shifts were then computed. Coordinates and GaussView 6 images for all optimized structures including conformational minima may be found in Tables S18–S26 in Supporting Information File 1.

For Table 4, isotropic absolute magnetic shielding values were calculated for isomer A of **1a** and **32** (both of which were about 90% of the total, and isomer B did not differ significantly for both) and for **8** for each method. In order to scale these isotropic values, the absolute shieldings were calculated in each case for the two most distant points in the trivalent scaling plots corresponding to Figure 2, namely, PH_3_ (liquid phase) and PCl_3_ (experimental geometry), and these two points were then used (Equation 4, which does not require the reference H_3_PO_4_) to scale the test compound values. Comparison of the values for the first four entries in Table 4 to those in Table 2 and Table 3 shows the agreement is acceptable. In order to compare each method, the MAD for each was determined using only the problematical chemical shifts for **1a** (that is, C=P_B_) and **8** (dicoordinate P_B_) as well as that for **32**, but P_A_ for **1a** was also listed for each.

## Supporting Information

File 1 Tables of calculated absolute isotropic chemical shifts, isomer ratios, unscaled chemical shifts, linear regressions, scaled chemical shifts and deviations, and coordinates of DFT optimized structures used for NMR calculations.

File 2 Tables S1–S17 in editable format.
