# Supplementary material for: Improving the accuracy of 31P NMR chemical shift calculations by use of scaling methods
Source: Beilstein J Org Chem. 2023 Jan 10;19:36–56. doi: 10.3762/bjoc.19.4 (PMC9843238; doi:10.3762/bjoc.19.4)
Supplement: File 1 — Tables of calculated absolute isotropic chemical shifts, isomer ratios, unscaled chemical shifts, linear regressions, scaled chemical shifts and deviations, and coordinates of DFT optimized structures used for NMR calculations. [file Beilstein_J_Org_Chem-19-36-s001.pdf]

BEILSTEIN JOURNAL OF ORGANIC CHEMISTRY

Supporting Information

for

Improving the accuracy of ^{31}P NMR chemical shift calculations by use of scaling methods

William H. Hersh and Tsz-Yeung Chan

Beilstein J. Org. Chem. **2023,** *19,* 36–56. doi:10.3762/bjoc.19.4

Tables of calculated absolute isotropic chemical shifts, isomer ratios, unscaled chemical shifts, linear regressions, scaled chemical shifts and deviations, and coordinates of DFT optimized structures used for NMR calculations

License and Terms: This is a supporting information file under the terms of the Creative Commons Attribution License (https://creativecommons.org/licenses/by/4.0). Please note that the reuse, redistribution and reproduction in particular requires that the author(s) and source are credited and that individual graphics may be subject to special legal provisions.
The license is subject to the *Beilstein Journal of Organic Chemistry* terms and conditions: (https://www.beilstein-journals.org/bjoc/terms)

SUPPORTING INFORMATION

Table of Contents **Page**

Table S1. Absolute chemical shifts for tri and tetracoordinate phosphorus compounds. S3

Table S2. Calculation of energy-weighted isomer ratios for Table S1 compounds. S4

Table S3. Calculated (unscaled) chemical shifts for tri and tetracoordinate phosphorus compounds referenced to H3PO4 at 0.00 ppm. S5

Table S4. Linear regressions, P(exp) vs P(calcd) chemical shifts, ppm. S6

Table S5. Scaled chemical shifts (eq 3 in text) from best fit parameters in Table S4 and unscaled calculations in Table S3; deviation from experimental. S7

Table S6. Absolute chemical shifts for Table 2 and 3 phosphorus compounds. S9

Table S7. Calculation of energy-weighted isomer ratios For Table S6 and S15 compounds.S10

Table S8. Unscaled chemical shifts for **1a-34[O]** referenced to H3PO4 at 0.00 ppm. S11

Table S9. Scaled chemical shifts (eq 3 in text) from best fit parameters in Table S4 and unscaled calculations in Table S8; deviation from experimental. S12

Table S10. Absolute chemical shifts for tri and tetracoordinate phosphorus compounds for screened functionals. S14

Table S11. Calculation of energy-weighted isomer ratios for Table S10 ωB97X-D optimization. S15

Table S12. Chemical shifts (1) for tri and tetracoordinate phosphorus compounds referenced to H3PO4 at 0.00 ppm for screened functionals. S15

Table S13. Linear regressions, P(Exp) vs P(calcd) chemical shifts, ppm, for screened functionals. S16

Table S14. Scaled chemical shifts (eq 3 in text) from best fit parameters in Table S13 and unscaled calculations in Table S12, deviation from experimental. S16

Table S15. Absolute chemical shifts for Table 2 and 3 phosphorus compounds for screened functionals. S17

Table S16. Unscaled chemical shifts for **1a-34[O]** referenced to H3PO4 at 0.00 ppm for screened functionals. S19

Table S17. Scaled chemical shifts (eq 3 in text) from best fit parameters in Table S13 and unscaled calculations in Table S16; deviation from experimental. S20

Table S18. Coordinates of optimized structures for tricoordinate and tetracoordinate phosphorus compounds, B3LYP/6-31+G(d,p). S22

Table S19. Coordinates of optimized structures for tricoordinate and tetracoordinate phosphorus compounds, M06-2X/6-31+G(d,p). S51

Table S20. Coordinates of optimized structures for tricoordinate and tetracoordinate phosphorus compounds, PBE0 (pbe1pbe)/6-31+G(d,p), CHCl3 solvation. S80

Table S21. Coordinates of optimized structures for tricoordinate and tetracoordinate phosphorus compounds, PBE0 (pbe1pbe)/6-31+G(d), no solvent. S104

Table S22. Coordinates of optimized structures for **1a**–**34[O]**, B3LYP/6-31+G(d,p), IEFPCM solvation. S127

Table S23. Coordinates of optimized structures for **1a**–**34[O]**, M06-2X/6-31+G(d,p), IEFPCM solvation. S205

Table S24. Coordinates of optimized structures for **1a, 11**–**34[O]**, PBE0 (pbe1pbe)/6-31+G(d), no solvent. S286

Table S25. Coordinates of optimized structures for tricoordinate and tetracoordinate phosphorus compounds, ωB97x-D /6-31+G(d,p). S350

Table S26. Coordinates of optimized structures for **1a**–**34[O]**, ωB97x-D/6-31+G(d,p), IEFPCM solvation. S377

	A	B	C	D	E	F	G	H	I	J	K	L	M	N
1	**Table S1. Absolute chemical shifts (1) for tri and tetracoordinate phosphorus compounds**													
2	**Functional for Optimization**		**B3LYP**	**B3LYP**	**M06-2X**	**M06-2X**	**B3LYP**	**B3LYP**	**B3LYP**	**PBE0**	**PBE0 (3)**			
3	**Functional for NMR**		**B3LYP**	**B3LYP**	**B3LYP**	**M06-2X**	**B3LYP**	**B3LYP**	**PBE0**	**PBE0**	**PBE0 (3)**			
4	**Compound/Basis set (2)**	**Exp vs H3PO4**	**6-311G(d,p)**	**6-311+G(2d,p)**	**6-311+G(2d,p)**	**6-311+G(2d,p)**	**IGLO-III**	**pcS-2**	**6-311+G(2d,p)**	**6-311+G(2d,p)**	**6-311G(2d,2p)**			
5	PH3	-238.00	561.4026	554.9949	559.1874	578.4758	563.9239	551.9404	576.8307	578.5599	583.2180			
6	PMeH2	-163.50	469.8641	459.2714	463.6054	487.801	467.0987	455.2914	487.2367	488.0473	491.8479			
7	PMe2H	-98.50	397.5399	383.6854	389.2509	414.7242	391.5472	379.1749	415.4666	418.1932	421.8841			
8	PMe3	-61.58	354.5327	340.0746	349.3526	372.0986	348.9827	335.6283	372.4788	379.4884	383.0150			
9	PPh3	-5.28	294.1567	281.6929	291.0941	305.7704	289.3203	275.3571	311.8327	318.3909	319.7585			
10	Methoxyphospholane	133.30	118.1997	111.2174	126.4015	135.678	124.0972	110.6025	131.2446	138.4847	137.1363			
11	P(OMe)3 isomer A		124.0839	119.4166	131.0375	139.4699	131.2297	117.9748	139.8238	146.1609	143.3519			
12	P(OMe)3 isomer B		159.3134	150.1548	163.665	170.6128	161.9956	148.9811	169.7553	178.0191	175.0565			
13	P(OMe)3 isomer C		93.7333	92.5935	104.5126	112.6385	104.6146	90.3623	113.3299	119.9306	116.1640			
14	P(OMe)3 weighted avg (4)	141.41	125.1684	120.3333	135.2602	143.4696	132.1673	118.8515	140.6841	147.2957	145.7607			
15	PCl3 (exp struc) (5)	219.79	26.2463	25.2261	25.2261	42.1440	33.0505	22.1519	62.6203	62.6203	62.0324			
16	PCl3(DFT struc) (6)	219.79	-32.6576	-31.4470	0.4022	19.2026	-22.4430	-34.6513	9.8567	35.7787	37.7006			
17	H3PO4 in water sym isomer	0	290.1035	282.0007	287.9119	293.7132	292.3520	281.4812	295.5786	298.5135	298.8299			
18	PH4+ BF4- in MeOH	-105.3	435.1999	417.5336	419.2853	431.2196	422.1986	413.7348	436.8781	437.6322	460.1675			
19	P(OPh)4+ PF6-	-28.0	320.3945	310.9396	308.7353	312.3168	322.7251	312.7309	323.8273	326.6892	327.7926			
20	O=**P(**OCH2)3P=O **PO**	-18.1	316.9879	306.6971	310.1613	315.4699	316.8853	305.5755	320.291	321.9122	327.7003			
21	P(OMe)4+ BF4- isomer A		293.7627	285.8902	289.5174	293.6709	295.9276	285.6737	298.5492	301.1908	302.5662			
22	P(OMe)4+ BF4- isomer B		286.5171	278.1574	280.7099	285.7322	288.7473	277.9179	291.0797	293.4636	301.8041			
23	P(OMe)4+ BF4- weighted avg	1.9	293.5189	285.6300	289.5118	293.6659	295.6860	285.4127	298.2978	301.1756	302.4626			
24	(iPrO)2P(O)H isomer A		292.7864	283.6516	286.5025	289.1512	293.1384	281.5378	296.5339	297.7825	302.5754			
25	(iPrO)2P(O)H isomer B		292.6928	283.4433	286.1845	289.6327	292.3632	280.6151	296.1933	298.9345	301.6789			
26	(iPrO)2P(O)H isomer C		292.7164	283.4842	286.2554	289.7312	292.4108	280.6704	296.2267	298.9497				
27	(iPrO)2P(O)H isomer D		292.0816	282.9603	286.4154	289.0709	292.4884	280.864	295.8692	297.7289				
28	(iPrO)2P(O)H weighted avg	4.54	292.6981	283.4730	286.2379	289.6510	292.4549	280.7262	296.2313	298.8387	301.7037			
29	O=P(OCH2)3**P**=O **PC**	6.4	306.3423	289.9311	288.9422	294.6675	293.8652	284.6886	310.1258	310.1593	312.7363			
30	(MeO)2P(O)H isomer A		288.3787	278.5239	282.7784	286.5795	287.4558	275.6176	291.2727	294.2874	297.8290			
31	(MeO)2P(O)H isomer B		288.2770	278.2475	281.7604	285.2467	287.3945	275.6371	291.1085	293.1451	299.0173			
32	(MeO)2P(O)H isomer C		286.1769	276.4226	280.7155	284.3628	285.4767	273.6202	289.1771	291.8267	295.9179			
33	(MeO)2P(O)H weighted avg	11.3	288.0043	278.1356	282.4081	286.1631	287.1245	275.2973	290.9047	293.3690	297.4802			
34	PPh4+ Cl-		275.8048	262.3407	268.6715	275.5308	266.0932	253.5022	286.9132	290.7902				
35	PPh4+ Br-	23.17	278.7647	265.1155	271.5229	278.0110			289.4805	293.4090	294.9089			
36	PMe4+ Cl-		282.1388	260.5895	264.1852	277.3351	262.6347	253.7384	286.5637	290.2242				
37	PMe4+ Br-	25.1	282.1989	260.6188	265.2594	278.5869			286.5953	290.1385	291.3493			
38	(iPrO)2P(O)Me isomer A		268.1059	258.4052	262.6947	265.0377	267.1816	256.6082	273.282	276.151	283.8260			
39	(iPrO)2P(O)Me isomer B		262.7929	253.6975	257.8942	260.9372	262.8723	251.5593	268.5934	271.7117	276.2328			
40	(iPrO)2P(O)Me isomer C		268.3932	258.7101	262.7299	265.0743	267.4587	256.8950	273.5958	276.2831	283.7007			
41	(iPrO)2P(O)Me weighted avg	28.61	262.8875	253.7818	258.1502	261.1560	262.9495	251.6493	268.6775	271.9179	276.2690			
42	Ph3PO	29.10	275.3585	261.7736	266.1358	269.9906	266.7677	254.8995	281.3444	284.1501	291.4911			
43	(MeO)2P(O)Me isomer A		259.3569	249.2994	254.1831	257.6326	258.1745	246.9502	264.2404	267.6174	272.7183			
44	(MeO)2P(O)Me isomer B		258.6019	248.0900	253.7706	257.0235	256.8728	245.5779	262.9209	266.7517	272.694			
45	(MeO)2P(O)Me isomer C		266.0172	255.1739	260.074	262.7857	263.8044	252.7744	269.9855	273.2287	279.2519			
46	(MeO)2P(O)Me weighted avg	32.3	259.4412	249.3580	254.5883	257.9719	258.2265	247.0032	264.2937	267.7321	272.7496			
47	Me3PO	38.79	272.4578	254.7079	259.2502	264.1381	259.2207	249.6721	275.3105	278.9888	292.0078			
48	EtOP(O)Me2 isomer A		248.2353	234.2842	239.8656	242.4268	241.2125	230.5865	251.6279	255.4460	265.4069			
49	EtOP(O)Me2 isomer B		252.8871	237.8724	242.4183	244.3041	244.9805	234.1468	255.2572	258.3252	273.0888			
50	EtOP(O)Me2 weighted avg	50.3	248.2898	234.3262	239.8908	242.4453	241.2566	230.6282	251.6704	255.4770	265.4141			

	A	B	C	D	E	F	G	H	I	J	K	L	M	N
51	H2P-P(H)-PH2 isomer A **P**H2										436.4497, 436.4497			
52	H2P-P(H)-PH2 isomer A **P**H										481.4502			
53	H2P-P(H)-PH2 isomer B **P**H2										508.7065, 508.7064			
54	H2P-P(H)-PH2 isomer B **P**H										464.9642			
55	H2P-P(H)-PH2 isomer C **P**H2										508.4149, 522.5669			
56	H2P-P(H)-PH2 isomer C **P**H										483.9058			
57	H2PP(H)PH2 weighted avg **P**H2	-162.6									457.6772			
58	H2PP(H)PH2 weighted avg **P**H	-179.1									478.5935			
59	H2P-PH2 isomer A										527.7870			
60	H2P-PH2 isomer B										537.7651			
61	H2P-PH2 weighted avg	-205.0									532.2788			
62	(1) Except as noted, all optimized with B3LYP/6-31+G(d,p), CHCl3 IEF-PCM solvation													
63	(2) For NMR calculation, GIAO													
64	(3) Latypov optimization (see text): optimized with 6-31+G(d) basis set, no solvation, and NMR with no solvation (both including H3PO4, which did not use water solvation).													
65	(4) See Table S2 for weighted average isomer ratio calculations													
66	(5) Experimental structure used for NMR calculation (see text)													
67	(6) DFT optimized structure used for NMR calculation													
68														
69														
70	**Table S2. Calculation of energy-weighted isomer ratios for Table S1 compounds**										**E (au) (1)**			
71		**E (au) (1)**	**Relative E**		**E (au) (1)**	**Relative E**		**E (au) (1)**	**Relative E**		**no solvent**	**Relative E**		
72	**Functional for Optimization**	**B3LYP**	**(kcal)**	**Ratio 298.15 K**	**M06-2X**	**(kcal)**	**Ratio 298.15 K**	**PBE0**	**(kcal)**	**Ratio 298.15 K**	**PBE0**	**(kcal)**	**Ratio 298.15 K**	
73	**Basis set**	**6-31+G(d,p)**			**6-31+G(d,p)**			**6-31+G(d,p)**			**6-31+G(d)**			
74	P(OMe)3 Isomer													
75	A	-686.722417	0	0.8083	-686.524666	0	0.8336	-686.181104	0	0.8132	-686.164323	0	0.8789	
76	B	-686.720492	1.2080	0.1052	-686.523021	1.0323	0.1460	-686.179161	1.2193	0.1039	-686.162240	1.3071	0.0968	
77	C	-686.720306	1.3247	0.0864	-686.521162	2.1988	0.0204	-686.178948	1.3529	0.0829	-686.160934	2.1266	0.0243	
78	P(OMe)4+ BF4- isomer													
79	A	-1226.318728	0	0.9663	-1225.935583	0	0.9994	-1225.235861	0	0.9980	-1225.192551	0	0.8640	
80	B	-1226.315558	1.9892	0.0337	-1225.928628	4.3643	0.0006	-1225.229980	3.6904	0.0020	-1225.190805	1.0956	0.1360	
81	(iPrO)2P(O)H isomer													
82	A	-804.640120	1.4019	0.0686	-804.37518	1.6165	0.0334	-803.955953	1.3811	0.0657	-803.926120	2.1091	0.0277	
83	B	-804.640982	0.8609	0.1708	-804.377586	0.1067	0.4270	-803.957148	0.6313	0.2330	-803.929481	0	0.9723	
84	C	-804.642354	0	0.7306	-804.377756	0	0.5112	-803.958154	0	0.6761				
85	D	-804.639339	1.8919	0.0300	-804.375028	1.7118	0.0284	-803.955049	1.9484	0.0252				
86	(MeO)2P(O)H isomer													
87	A	-647.455600	0.0000	0.6649	-647.278831	0	0.7814	-646.967743	0	0.5625	-646.950502	0	0.7784	
88	B	-647.454329	0.7976	0.1730	-647.276646	1.3711	0.0772	-646.966285	0.9149	0.1201	-646.947220	2.0595	0.0241	
89	C	-647.454267	0.8365	0.1620	-647.277217	1.0128	0.1414	-646.967203	0.3389	0.3175	-646.949207	0.8126	0.1975	
90	(iPrO)2P(O)Me isomer													
91	A	-843.946567	2.8853	0.0075	-843.663617	2.0356	0.0305	-843.214641	2.4749	0.0146	-843.181092	3.7331	0.0018	
92	B	-843.951165	0	0.9827	-843.666861	0	0.9468	-843.218585	0	0.9545	-843.187041	0	0.9952	
93	C	-843.946808	2.7341	0.0097	-843.663337	2.2113	0.0227	-843.215346	2.0325	0.0309	-843.181556	3.4419	0.0030	
94	(MeO)2P(O)Me isomer													
95	A	-686.765788	0	0.9549	-686.566746	0	0.8661	-686.229522	0	0.9353	-686.208822	0	0.5109	
96	B	-686.762492	2.0683	0.0291	-686.564238	1.5738	0.0608	-686.226505	1.8932	0.0383	-686.208768	0.0339	0.4825	
97	C	-686.761925	2.4241	0.0160	-686.564411	1.4652	0.0730	-686.226152	2.1147	0.0264	-686.204713	2.5784	0.0066	
98	EtOP(O)Me2 isomer													
99	A	-650.830403	0.0000	0.9883	-650.632536	0	0.9901	-650.322495	0	0.9892	-650.297318	0	0.9991	
100	B	-650.826215	2.6280	0.0117	-650.628185	2.7303	0.0099	-650.318228	2.6776	0.0108	-650.290734	4.1315	0.0009	

	A	B	C	D	E	F	G	H	I	J	K	L	M	N
101	H2P-P(H)-PH2 isomer											**at 213.15 K**	**Ratio 213.15 K**	
102	A										-1026.578464	0	0.7153	
103	B										-1026.577561	0.5666	0.1877	
104	C										-1026.577115	0.8465	0.0970	
105	H2P-PH2 isomer													
106	A										-684.773859	0	0.5498	
107	B										-684.773724	0.0847	0.4502	
108	(1) Sum of electronic and thermal Free Energies from *Gaussian* vibrational calculation; see Table S1 notes for solvation.													
109	Gas constant from IUPAC:		1.9872	cal mol-1 J-1										
110	E conversion:		627.5095	kcal/mol/au										
111														
112														
113	**Table S3. Calculated (unscaled) chemical shifts (1) for tri and tetracoordinate phosphorus compounds referenced to H3PO4 at 0.00 ppm**													
114	**Functional for Optimization**		**B3LYP**	**B3LYP**	**M06-2X**	**M06-2X**	**B3LYP**	**B3LYP**	**B3LYP**	**PBE0**	**Latypov PBE0**			
115	**Functional for NMR (2)**		**B3LYP**	**B3LYP**	**B3LYP**	**M06-2X**	**B3LYP**	**B3LYP**	**PBE0**	**PBE0**	**Latypov PBE0**			
116	**Compound/NMR basis set**	**Exp vs H3PO4**	**6-311G(d,p)**	**6-311+G(2d,p)**	**6-311+G(2d,p)**	**6-311+G(2d,p)**	**IGLO-III**	**pcS-2**	**6-311+G(2d,p)**	**6-311+G(2d,p)**	**6-311G(2d,2p)**			
117	PH3	-238	-271.2991	-272.9942	-271.2755	-284.7626	-271.5719	-270.4592	-281.2521	-280.0464	-284.3881			
118	PMeH2	-163.5	-179.7606	-177.2707	-175.6935	-194.0878	-174.7467	-173.8102	-191.6581	-189.5338	-193.0180			
119	PMe2H	-98.5	-107.4364	-101.6847	-101.3390	-121.0110	-99.1952	-97.6937	-119.8880	-119.6797	-123.0542			
120	PMe3	-61.58	-64.4292	-58.0739	-61.4407	-78.3854	-56.6307	-54.1471	-76.9002	-80.9749	-84.1851			
121	PPh3	-5.28	-4.0532	0.3078	-3.1822	-12.0572	3.0317	6.1241	-16.2541	-19.8774	-20.9286			
122	Methoxyphospholane	133.3	171.9038	170.7833	161.5104	158.0352	168.2548	170.8787	164.3340	160.0288	161.6936			
123	P(OMe)3	141.41	164.9351	161.6674	152.6517	150.2436	160.1847	162.6297	154.8945	151.2178	153.0692			
124	PCl3 (exp struc)	219.79	263.8572	256.7746	262.6858	251.5692	259.3015	259.3293	232.9583	235.8932	236.7975			
125	PH4+ in MeOH	-105.3	-145.0964	-135.5329	-131.3734	-137.5064	-129.8466	-132.2536	-141.2995	-139.1187	-161.3376			
126	PhO)4P+ PF6-	-28	-30.2910	-28.9389	-20.8234	-18.6036	-30.3731	-31.2497	-28.2487	-28.1757	-28.9627			
127	O=POCH2)3P=O PO	-18.1	-26.8844	-24.6964	-22.2494	-21.7567	-24.5333	-24.0943	-24.7124	-23.3987	-28.8704			
128	P(OMe)4+ BF4-	1.9	-3.4154	-3.6293	-1.5999	0.0473	-3.3340	-3.9315	-2.7192	-2.6621	-3.6327			
129	(iPrO)2P(O)H	4.54	-2.5946	-1.4723	1.6740	4.0622	-0.1029	0.7550	-0.6527	-0.3252	-2.8738			
130	O=POCH2)3P=O PC	6.4	-16.2388	-7.9304	-1.0303	-0.9543	-1.5132	-3.2074	-14.5472	-11.6458	-13.9064			
131	MeO)2P(O)H	11.3	2.0992	3.8651	5.5038	7.5501	5.2275	6.1839	4.6739	5.1445	1.3497			
132	Ph4P+Br-/Cl-	23.17	11.3388	16.8852	16.3890	15.7022	26.2588	27.9790	6.0981	5.1045	3.9210			
133	Me4P Br-/Cl-	25.1	7.9046	21.3819	22.6525	15.1263	29.7173	27.7428	8.9833	8.3750	7.4806			
134	(iPrO)2P(O)Me	28.61	27.2160	28.2189	29.7617	32.5572	29.4025	29.8319	26.9011	26.5956	22.5609			
135	Ph3PO	29.1	14.7450	20.2271	21.7761	23.7226	25.5843	26.5817	14.2342	14.3634	7.3388			
136	(MeO)2P(O)Me	32.3	30.6623	32.6427	33.3236	35.7413	34.1255	34.4780	31.2849	30.7814	26.0803			
137	Me3PO	38.79	17.6457	27.2928	28.6617	29.5751	33.1313	31.8091	20.2681	19.5247	6.8221			
138	EtOP(O)Me2	50.3	41.8137	47.6745	48.0211	51.2679	51.0954	50.8530	43.9082	43.0365	33.4158			
139	H2P-P(H)-PH2 **P**H2	-162.6									-158.8473			
140	H2P-P(H)-PH2 **P**H	-179.1									-179.7636			
141	H2P-PH2	-205.0									-233.4489			
142	See Table S1 Notes 1-3 (Note 3 is for Latypov PBE0)													
143	(1) Chemical shifts calc'd according to text eq 1: δ(31P)Calcd = σ(reference)Calcd – σ(31P)Calcd + δ(reference)Exp													
144														
145														
146														
147														
148														
149														
150														

	A	B	C	D	E	F	G	H	I	J	K	L	M	N
151	**Table S4. Linear regressions, P(Exp) vs P(calcd) chemical shifts, ppm**													
152	**Functional for Optimization**		**B3LYP**	**B3LYP**	**M06-2X**	**M06-2X**	**B3LYP**	**B3LYP**	**B3LYP**	**PBE0**	**Latypov PBE0**			
153	**Functional for NMR**		**B3LYP**	**B3LYP**	**B3LYP**	**M06-2X**	**B3LYP**	**B3LYP**	**PBE0**	**PBE0**	**Latypov PBE0**			
154	**Basis set for NMR**		**6-311G(d,p)**	**6-311+G(2d,p)**	**6-311+G(2d,p)**	**6-311+G(2d,p)**	**IGLO-III**	**pcS-2**	**6-311+G(2d,p)**	**6-311+G(2d,p)**	**6-311G(2d,2p)**			
155	Chi squared		2462.904228	1537.747034	1221.004026	703.477493	1121.685333	1404.335651	1276.355477	1114.839809	2073.054457			
156	slope		0.854923	0.869464	0.878928	0.855386	0.873516	0.871521	0.87136	0.876551	0.858159			
157	std dev		0.02117285	0.01697555	0.01527922	0.01127252	0.01455086	0.01625549	0.01548931	0.01455656	0.01947961			
158	intercept		5.045476	2.229725	1.476937	4.91327	-0.06747987	-0.494732	8.770647	9.045654	12.363916			
159	std dev		2.367761	1.869555	1.66584	1.265357	1.596801	1.786825	1.708399	1.597009	2.185178			
160	Correlation coefficient		0.993922	0.99621	0.996992	0.998268	0.997237	0.996539	0.996855	0.997254	0.994887			

	A	B	C	D	E	F	G	H	I	J	K	L	M	N
161	**Table S5. Scaled chemical shifts (eq 3 in text) from best fit parameters in Table S4 and unscaled calculations in Table S3; deviation from experimental**													
162	**Functional for Optimization**		**B3LYP**		**B3LYP**		**M06-2X**		**M06-2X**		**B3LYP**		**B3LYP**	
163	**Functional for NMR**		**B3LYP**		**B3LYP**		**B3LYP**		**M06-2X**		**B3LYP**		**B3LYP**	
164	**Compound/NMR basis set**	**Exp vs H3PO4**	**6-311G(d,p)**	**Deviation**	**6-311+G(2d,p)**	**Deviation**	**6-311+G(2d,p)**	**Deviation**	**6-311+G(2d,p)**	**Deviation**	**IGLO-III**	**Deviation**	**pcS-2**	**Deviation**
165	PH3	-238	-226.89	11.11	-235.13	2.87	-236.95	1.05	-238.67	0.67	-237.29	0.71	-236.21	1.79
166	PMeH2	-163.5	-148.64	14.86	-151.90	11.60	-152.94	10.56	-161.11	2.39	-152.71	10.79	-151.97	11.53
167	PMe2H	-98.5	-86.80	11.70	-86.18	12.32	-87.59	10.91	-98.60	0.10	-86.72	11.78	-85.64	12.86
168	PMe3	-61.58	-50.04	11.54	-48.26	13.32	-52.53	9.05	-62.14	0.56	-49.54	12.04	-47.69	13.89
169	PPh3	-5.28	1.58	6.86	2.50	7.78	-1.32	3.96	-5.40	0.12	2.58	7.86	4.84	10.12
170	Methoxyphospholane	133.3	152.01	18.71	150.72	17.42	143.43	10.13	140.09	6.79	146.91	13.61	148.43	15.13
171	P(OMe)3	141.41	146.05	4.64	142.79	1.38	135.65	5.76	133.43	7.98	139.86	1.55	141.24	0.17
172	PCl3 (exp struc)	219.79	230.62	10.83	225.49	5.70	232.36	12.57	220.10	0.31	226.44	6.65	225.52	5.73
173	PH4+ in MeOH	-105.3	-119.00	13.70	-115.61	10.31	-119.38	14.08	-112.71	7.41	-113.49	8.19	-115.76	10.46
174	PhO)4P+ PF6-	-28	-20.85	7.15	-22.93	5.07	-14.87	13.13	-11.00	17.00	-26.60	1.40	-27.73	0.27
175	O=POCH2)3P=O PO	-18.1	-17.94	0.16	-19.24	1.14	-17.65	0.45	-13.70	4.40	-21.50	3.40	-21.49	3.39
176	P(OMe)4+ BF4-	1.9	2.13	0.23	-0.93	2.83	1.52	0.38	4.95	3.05	-2.98	4.88	-3.92	5.82
177	(iPrO)2P(O)H	4.54	2.83	1.71	0.95	3.59	5.05	0.51	8.39	3.85	-0.16	4.70	0.16	4.38
178	O=POCH2)3P=O PC	6.4	-8.84	15.24	-4.67	11.07	0.64	5.76	4.10	2.30	-1.39	7.79	-3.29	9.69
179	MeO)2P(O)H	11.3	6.84	4.46	5.59	5.71	8.11	3.19	11.37	0.07	4.50	6.80	4.89	6.41
180	Ph4P+Br-/Cl-	23.17	14.74	8.43	16.91	6.26	15.28	7.89	18.34	4.83	22.87	0.30	23.89	0.72
181	Me4P Br-/Cl-	25.1	11.80	13.30	20.82	4.28	14.77	10.33	17.85	7.25	25.89	0.79	23.68	1.42
182	(iPrO)2P(O)Me	28.61	28.31	0.30	26.77	1.84	30.09	1.48	32.76	4.15	25.62	2.99	25.50	3.11
183	Ph3PO	29.1	17.65	11.45	19.82	9.28	22.33	6.77	25.21	3.89	22.28	6.82	22.67	6.43
184	(MeO)2P(O)Me	32.3	31.26	1.04	30.61	1.69	32.89	0.59	35.49	3.19	29.74	2.56	29.55	2.75
185	Me3PO	38.79	20.13	18.66	25.96	12.83	27.47	11.32	30.21	8.58	28.87	9.92	27.23	11.56
186	EtOP(O)Me2	50.3	40.79	9.51	43.68	6.62	46.54	3.76	48.77	1.53	44.57	5.73	43.82	6.48
187	**MAD**			**8.89**		**7.04**		**6.53**		**4.11**		**5.97**		**6.55**
188	**RMSD**			**10.58**		**8.36**		**7.97**		**5.65**		**7.14**		**7.99**
189														
190	**Table S5 Continued**													
191	**Functional for Optimization**		**B3LYP**		**PBE0**		**Latypov PBE0**							
192	**Functional for NMR**		**PBE0**		**PBE0**		**Latypov PBE0**							
193	**Compound/NMR basis set**	**Exp vs H3PO4**	**6-311+G(2d,p)**	**Deviation**	**6-311+G(2d,p)**	**Deviation**	**6-311G(2d,2p)**							
194	PH3	-238	-236.30	1.70	-236.43	1.57	-231.69	6.31						
195	PMeH2	-163.5	-158.23	5.27	-157.09	6.41	-153.28	10.22						
196	PMe2H	-98.5	-95.69	2.81	-95.86	2.64	-93.24	5.26						
197	PMe3	-61.58	-58.24	3.34	-61.93	0.35	-59.88	1.70						
198	PPh3	-5.28	-5.39	0.11	-8.38	3.10	-5.60	0.32						
199	Methoxyphospholane	133.3	151.96	18.66	149.32	16.02	151.12	17.82						
200	P(OMe)3	141.41	143.74	2.33	141.60	0.19	143.72	2.31						
201	PCl3 (exp struc)	219.79	211.76	8.03	215.82	3.97	215.57	4.22						
202	PH4+ in MeOH	-105.3	-114.35	9.05	-112.90	7.60	-126.09	20.79						
203	PhO)4P+ PF6-	-28	-15.84	12.16	-15.65	12.35	-12.49	15.51						
204	O=POCH2)3P=O PO	-18.1	-12.76	5.34	-11.46	6.64	-12.41	5.69						
205	P(OMe)4+ BF4-	1.9	6.40	4.50	6.71	4.81	9.25	7.35						
206	(iPrO)2P(O)H	4.54	8.20	3.66	8.76	4.22	9.90	5.36						
207	O=POCH2)3P=O PC	6.4	-3.91	10.31	-1.16	7.56	0.43	5.97						
208	MeO)2P(O)H	11.3	12.84	1.54	13.56	2.26	13.52	2.22						
209	Ph4P+Br-/Cl-	23.17	14.08	9.09	13.52	9.65	15.73	7.44						
210	Me4P Br-/Cl-	25.1	16.60	8.50	16.39	8.71	18.78	6.32						

	A	B	C	D	E	F	G	H	I	J	K	L	M	N
211	(iPrO)2P(O)Me	28.61	32.21	3.60	32.36	3.75	31.72	3.11						
212	Ph3PO	29.1	21.17	7.93	21.64	7.46	18.66	10.44						
213	(MeO)2P(O)Me	32.3	36.03	3.73	36.03	3.73	34.74	2.44						
214	Me3PO	38.79	26.43	12.36	26.16	12.63	18.22	20.57						
215	EtOP(O)Me2	50.3	47.03	3.27	46.77	3.53	41.04	9.26						
216	**MAD**			**6.24**		**5.87**		**7.76**						
217	**RMSD**			**7.62**		**7.12**		**9.71**						
218														
219														
220														
221														
222														

	A	B	C	D	E	F	G	H	I	J	K	L	M	N
223														
224	**Table S6. Absolute chemical shifts for Table 2 and 3 phosphorus compounds**													
225	**Functional for Optimization (1)**			**B3LYP**	**M06-2X**	**M06-2X**	**B3LYP**	**Latypov PBE0 (3)**						
226	**Functional for NMR**			**B3LYP**	**B3LYP**	**M06-2X**	**B3LYP**	**Latypov PBE0 (3)**						
227	**Compound/NMR Basis set (2)**			**6-311+G(2d,p)**	**6-311+G(2d,p)**	**6-311+G(2d,p)**	**IGLO-III**	**6-311G(2d,2p)**						
228	**1a P(A) isomer A**			230.8443	234.5915	233.7611	240.6495	269.9121						
229	**1a P(B) isomer A**			-42.5672	-35.8930	-93.9873	-26.0863	-9.3202						
230	**1a P(A) isomer B**			229.6119	233.3273	235.4204	239.6173	270.1485						
231	**1a P(B) isomer B**			-71.5659	-62.4552	-125.948	-55.7724	-40.7723						
232	**1a P(A) weighted avg (4)**			230.7520	234.4830	233.9035	240.5722	269.9319						
233	**1a P(B) weighted avg (4)**			-44.7401	-38.1720	-96.7294	-28.3107	-11.9541						
234	**2**			410.0211	413.3227	423.6051	410.6879							
235	**3**			342.9311	346.5690	354.4792	346.9897							
236	**4**			257.1773	264.1619	268.9631	263.2142							
237	**5 P(A)**			272.7993	274.8505	275.7315	279.1902							
238	**5 P(B)**			263.9253	264.3702	266.5416	270.3387							
239	**6**			281.1452	289.4736	306.1356	287.0774							
240	**7**			232.8052	244.9353	247.0436	243.3976							
241	**8 P(A)**			198.3979	204.3552	211.4237	203.3708							
242	**8 P(B)**			411.7729	428.1517	434.4661	421.0008							
243	**9 P(A)**			290.1006	311.8738	327.9363	296.7368							
244	**9 P(B)**			180.2356	192.3113	189.0313	187.3524							
245	**9 P(C)**			266.8119	272.4523	283.8192	275.6957							
246	**9 P(D)**			210.6058	215.1917	214.6840	216.6421							
247	**10 P(A)**			283.4696	296.3450	310.8896	289.3250							
248	**10 P(B)**			162.9194	183.4346	183.5823	172.6289							
249	**11 isomer A**			99.7594	111.7599	118.7464	112.0194	134.6205						
250	**11 isomer B**			129.4127	137.1948	140.8452	138.2311	124.6372						
251	**11 isomer C**			91.3904	102.2557	106.0365	101.7616							
252	**11 weighted avg**			104.0965	115.1673	121.6273	115.8135	134.5405						
253	**12 isomer A**			133.2982	134.9449	139.0794	142.7379	150.0595						
254	**12 isomer B**			109.8678	121.0574	127.0474	120.5527	141.9650						
255	**12 isomer C**							157.4160						
256	**12 weighted avg**			121.2853	132.0456	136.5675	131.3634	151.5631						
257	**13**			130.6901	146.9384	153.3203	139.8435	174.9370						
258	**14 isomer A**			118.9454	142.7093	145.7902	128.8150	164.9695						
259	**14 isomer B**			113.5567	143.5262	144.1594	124.1035	159.2454						
260	**14 weighted avg**			118.0089	143.0161	145.1777	127.9962	164.2200						
261	**15**			135.8183	145.8343	153.6870	145.8308	165.5785						
262	**16 isomer A**			80.3456	90.2505	98.7984	91.6320	107.1585						
263	**16 isomer B**			121.0841	136.0574	144.3690	132.1837	151.6626						
264	**16 isomer C**			121.4284	126.5800	135.1006	132.2766	152.3553						
265	**16 weighted avg**			121.0860	133.4884	141.8566	132.0928	151.8616						
266	**17 isomer A (cis, chair)**			136.3846	145.1146	153.2455	145.7294	168.5981						
267	**17 isomer B (cis, twist 1)**			136.2766	146.4685	153.3577	147.0282	170.3364						
268	**17 isomer C (cis, twist 2)**			144.6359	151.8747	158.6677	154.7464	176.2653						
269	**17 weighted avg**			136.6912	146.0171	153.4226	146.8042	169.7479						
270	**18 isomer A (trans, chair)**			115.7809	115.7809	123.9566	126.6326	148.4207						
271	**18 isomer B trans, twist 1)**			122.1044	130.5967	138.4116	131.9724	154.3104						
272	**18 isomer C (trans, twist 2)**			116.3942	147.2430	154.5105	147.3797	170.3855						

	A	B	C	D	E	F	G	H	I	J	K	L	M	N
273	**18 weighted avg**			115.8124	125.2201	133.1244	127.4822	149.1521						
274	**19**			76.0963	86.0344	98.0139	86.7760	113.5430						
275	**20**			114.5484	123.0137	125.5709	127.8598	131.5914						
276	**21 (dicoordinate P)**			-119.0410	-114.5126	-165.9889	-102.4641	-86.4483						
277	**21 (PPh3)**			260.8139	267.7679	271.6541	265.6456	290.7625						
278	**22**			-72.4444	-51.4050	-110.7357	-56.6501	-15.6428						
279	**23**			429.1942	431.5634	424.5137	436.2070	452.7203						
280	**24**			432.9381	438.8665	440.4980	439.1803	463.7260						
281	**25**			210.1853	211.4632	213.4065	217.8574	244.9526						
282	**26**			75.6259	82.3775	36.5498	88.2064	101.4026						
283	**27 isomer A**			271.9647	275.6425	278.7163	281.2612	292.0410						
284	**27 isomer B**			271.6359	274.6476	277.6802	281.3687	299.2730						
285	**27 isomer C**							289.6904						
286	**27 weighted avg**			271.9619	275.6376	278.7112	281.2621	292.1149						
287	**28**			268.2862	272.0144	275.3758	277.6893	288.8707						
288	**29**			191.6073	179.0976	177.1895	198.4762	212.2291						
289	*anti* -**30**			244.5968	260.8983	278.5421	253.3642	293.0210						
290	*syn* -**30 isomer A**			257.9899	272.4174	289.2968	265.4850	305.1780						
291	*syn* -**30 isomer B**			247.1842	262.1814	280.6598	256.0548	294.3197						
292	*syn* -**30 weighted average**			257.5751	271.8797	288.8431	265.1230	304.8621						
293	*anti* -**30[O] isomer A**			233.0412	241.6411	246.3594	237.8604	265.3531						
294	*anti* -**30[O] isomer B**							259.0811						
295	*anti* -**30[O] weighted average**							265.2442						
296	*syn* -**30[O] isomer A**			225.7662	233.3639	238.4576	230.9454	258.3357						
297	*syn* -**30[O] isomer B**			234.7199	244.0628	248.4401	240.0223	267.4185						
298	*syn* -**30[O] weighted average**			227.4588	236.9087	241.7651	232.6613	259.3712						
299	*anti* -**31**			298.4525	322.8111	332.0787	302.0069	346.6677						
300	*syn* -**31**			285.5813	313.9248	322.3614	289.2733	337.0564						
301	**32 isomer A**			348.1362	477.3828	504.8594	357.5957	440.5655						
302	**32 isomer B**			360.7959	497.2469	525.9140	369.7054	444.9776						
303	**32 weighted avg**			352.6421	482.2016	509.9670	361.9058	441.7717						
304	**33**			375.1876	374.5296	383.7343	382.0624	410.1208						
305	**34**			288.7498	302.3060	311.1242	295.7162	335.0661						
306	**33[O]**			253.2303	255.1156	256.9684	258.1458	286.7926						
307	**34[O]**			270.0398	274.0285	275.1703	274.8957	301.3912						
308	(1) Except as noted, all optimized with 6-31+G(d,p) basis set; both the optimization and NMR used the solvents listed in													
309	Tables 2 and 3 with IEF-PCM solvation													
310	(2) For NMR calculation													
311	(3) Latypov optimization (see text): optimized with 6-31+G(d) basis set and no solvation; NMR: no solvation.													
312	(4) See Table S7 for weighted average isomer ratio calculations; if only one value is reported in Table S6, only one isomer was found.													
313														
314														
315	**Table S7. Calculation of energy-weighted isomer ratios for Table S6 and S15 compounds**													
316		**E (au) (1)**		**Relative E**		**E (au) (1)**	**Relative E**		**E (au) (1)**	**Relative E**		**E (au) (1)**	**Relative E**	
317	**Functional for Optimization**	**B3LYP**		**(kcal)**	**Ratio 298.15 K**	**M06-2X**	**(kcal)**	**Ratio 298.15 K**	**PBE0**	**(kcal)**	**Ratio 298.15 K**	**ωB97X-D**	**(kcal)**	**Ratio 298.15 K**
318	**Basis set**	**6-31+G(d,p)**	**solvent**			**6-31+G(d,p)**			**6-31+G(d)**			**6-31+G(d,p)**		
319	**1a isomer A**	-1310.118082	benzene	0	0.9251	-1309.720519	0	0.9142	-1309.012606	0	0.9163	-1309.900807	0	0.9086
320	**1a isomer B**	-1310.115709	benzene	1.4891	0.0749	-1309.718285	1.4019	0.0858	-1309.010347	1.4175	0.0837	-1309.898638	1.3611	0.0914
321	**11 isomer A**	-1091.671654	toluene	0	0.8360	-1091.302278	0	0.8415	-1090.612361	0	0.9920	-1091.405142	0	0.5854
322	**11 isomer B**	-1091.670033	toluene	1.0172	0.1502	-1091.300589	1.0599	0.1407	-1090.607811	2.8552	0.0080	-1091.404765	0.2366	0.3927

	A	B	C	D	E	F	G	H	I	J	K	L	M	N
323	**11 isomer C**	-1091.667783	toluene	2.4291	0.0139	-1091.298642	2.2816	0.0179				-1091.402038	1.9478	0.0219
324	**12 isomer A**	-1091.671458	toluene	0.0301	0.4873	-1091.305029	0	0.7912	-1090.614472	0	0.6219	-1091.407394	0	0.6955
325	**12 isomer B**	-1091.671506	toluene	0	0.5127	-1091.303771	0.7894	0.2088	-1090.612567	1.1954	0.0827	-1091.406614	0.4895	0.3045
326	**12 isomer C**								-1090.613769	0.4411	0.2954			
327	**14 isomer A**	-1792.903243	chloroform	0	0.8262	-1792.387462	0	0.6244	-1791.371252	0	0.8691	-1792.534008	0	0.4130
328	**14 isomer B**	-1792.901771	chloroform	0.9237	0.1738	-1792.386982	0.3012	0.3756	-1791.369465	1.1214	0.1309	-1792.534340	-0.2083	0.5870
329	**15 isomer A (cis, chair)**	-901.378764	benzene	0	1	-901.076471	0	1.0000	-900.544416	0	1	-901.192958	0	0.9995
330	**15 isomer B (cis, twist 1)**	-901.370816	benzene	4.9874	0.0002	-901.069505	4.3712	0.0006	-900.537471	4.3581	0.0006	-901.185457	4.7069	0.0004
331	**15 isomer C (cis, twist 2)**	-901.370902	benzene	4.9335	0.0002	-901.069472	4.3919	0.0006	-900.537248	4.4980	0.0005	-901.184267	5.4537	0.0001
332	**16 isomer A (trans, chair)**	-901.369257	benzene	3.1457	0.0031	-901.064889	4.7490	0.0002	-900.533284	4.4484	0.0004	-901.180788	5.0376	0.0002
333	**16 isomer B (trans, twist 1)**	-901.374270	benzene	0	0.6255	-901.072457	0	0.7288	-900.540373	0	0.6880	-901.188816	0	0.7464
334	**16 isomer C (trans, twist 2)**	-901.373778	benzene	0.3087	0.3714	-901.071523	0.5861	0.2710	-900.539625	0.4694	0.3116	-901.187796	0.6401	0.2534
335	**17 isomer A (cis, chair)**	-920.765886	benzene	0.1098	0.4338	-920.449712	0.1757	0.4174	-919.896464	0	0.4886	-920.578892	0.0144	0.4822
336	**17 isomer B (cis, twist 1)**	-920.766061	benzene	0	0.5222	-920.449992	0	0.5615	-919.896422	0.0264	0.4674	-920.578915	0	0.4941
337	**17 isomer C (cis, twist 2)**	-920.763725	benzene	1.4659	0.0440	-920.446891	1.9459	0.0210	-919.894191	1.4263	0.0440	-920.576045	1.8010	0.0236
338	**18 isomer A (trans, chair)**	-920.768892	benzene	0	0.9583	-920.450264	0	0.6918	-919.899774	0	0.9659	-920.579964	0	0.8379
339	**18 isomer B trans, twist 1)**	-920.762439	benzene	4.0493	0.0010	-920.446674	2.2528	0.0154	-919.893379	4.0129	0.0011	-920.575476	2.8163	0.0072
340	**18 isomer C (trans, twist 2)**	-920.765909	benzene	1.8719	0.0407	-920.449452	0.5095	0.2927	-919.896586	2.0005	0.0330	-920.578370	1.0003	0.1549
341	**27 isomer A**	-1242.190806	benzene	0	0.9915	-1241.770093	0	0.9951	-1240.977494	0	0.9754	-1241.879394	0	0.9901
342	**27 isomer B**	-1242.186316	benzene	2.8175	0.0085	-1241.765078	3.1470	0.0049	-1240.973470	2.5251	0.0137	-1241.875048	2.7272	0.0099
343	**27 isomer C**								-1240.973246	2.6657	0.0108			
344	***syn*-30 isomer A**	-695.556246	toluene	0	0.9616	-695.323112	0	0.9475	-694.942244	0	0.9709	-695.437383	0	0.9573
345	***syn*-30 isomer B**	-695.553205	toluene	1.9083	0.0384	-695.320381	1.7137	0.0525	-694.938932	2.0783	0.0291	-695.434446	1.8430	0.0427
346	*anti*-**30[O] isomer A**	-770.817738	toluene			-770.557377			-770.124628	0	0.9826			
347	*anti*-**30[O] isomer B**								-770.120817	2.3914	0.0174			
348	*syn*-**30[O] isomer A**	-770.816337	toluene	0	0.8110	-770.555380	0	0.6687	-770.123142	0	0.8860	-770.677702	0	0.7247
349	*syn*-**30[O] isomer B**	-770.814962	toluene	0.862825562	0.1890	-770.554717	0.4160	0.3313	-770.121206	1.2149	0.1140	-770.676788	0.5735	0.2753
350	**32 isomer A**	-882.443537	chloroform	0	0.6441	-882.156376	0	0.7574	-881.622393	0	0.7266	-882.232307	0	0.7805
351	**32 isomer B**	-882.442977	chloroform	0.3514	0.3559	-882.155301	0.6746	0.2426	-881.621470	0.5792	0.2734	-882.231109	0.7518	0.2195
352	(1) See Notes in Table S2.													
353														
354														
355	**Table S8. Unscaled chemical shifts (1) for 1a-34[O] referenced to H3PO4 at 0.00 ppm**													
356	**Functional for Optimization**			**B3LYP**	**M06-2X**	**M06-2X**	**B3LYP**							
357	**Functional for NMR**			**B3LYP**	**B3LYP**	**M06-2X**	**B3LYP**	**Latypov PBE0 (3)**						
358	**Compound/NMR basis set (2)**	**Exp vs H3PO4**		**6-311+G(2d,p)**	**6-311+G(2d,p)**	**6-311+G(2d,p)**	**IGLO-III**	**6-311G(2d,2p)**						
359	**1a P(A) weighted avg (4)**	39.6		51.2487	53.4289	59.8097	51.7798	28.898						
360	**1a P(B) weighted avg (4)**	297.6		326.7408	326.0839	390.4426	320.6627	310.784						
361	**2**	-110		-128.0204	-125.4108	-129.8919	-118.3359	-129.3						
362	**3**	-54.5		-60.9304	-58.6571	-60.766	-54.6377	-68.6						
363	**4**	24		24.8234	23.75	24.7501	29.1378	22.5						
364	**5 P(A)**	18.3		9.2014	13.0614	17.9817	13.1618	5.8						
365	**5 P(B)**	27.6		18.0754	23.5417	27.1716	22.0133	15						
366	**6**	-10.2		0.8555	-1.5617	-12.4224	5.2746	-23.1						
367	**7**	38.7		49.1955	42.9766	46.6696	48.9544	36.8						
368	**8 P(A)**	76.7		83.6028	83.5567	82.2895	88.9812	63.9						
369	**8 P(B)**	-157.7		-129.7722	-140.2398	-140.7529	-128.6488	-172.4						
370	**9 P(A)**	-29.7		-8.0999	-23.9619	-34.2231	-4.3848	-41.9						
371	**9 P(B)**	100.5		101.7651	95.6006	104.6819	104.9996	84.1						
372	**9 P(C)**	-10.6		15.1888	15.4596	9.894	16.6563	-7.1						

	A	B	C	D	E	F	G	H	I	J	K	L	M	N
373	**9 P(D)**	75.7		71.3949	72.7202	79.0292	75.7099	58.7						
374	**10 P(A)**	-22.6		-1.4689	-8.4331	-17.1764	3.027	-36.5						
375	**10 P(B)**	84.1		119.0813	104.4773	110.1309	119.7231	74.3						
376	**11 weighted avg**	152.9		177.9042	172.7446	172.0859	176.5385	164.2894						
377	**12 weighted avg**	139.3		160.7154	155.8663	157.1457	160.9886	147.2668						
378	**13**	133.0		151.3106	140.9735	140.3929	152.5085	123.8929						
379	**14 weighted avg**	136.7		163.9918	144.8958	148.5355	164.3558	134.6099						
380	**15**	132.2		146.1824	142.0776	140.0262	146.5212	133.2514						
381	**16 weighted avg**	138.5		160.9147	154.4235	151.8566	160.2592	146.9683						
382	**17 weighted avg**	125.8		145.3095	141.8948	140.2906	145.5478	129.082						
383	**18 weighted avg**	145.3		166.1883	162.6918	160.5888	164.8698	149.6778						
384	**19**	169		205.9044	201.8775	195.6993	205.576	185.2869						
385	**20**	153		167.4523	164.8982	168.1423	164.4922	167.2385						
386	**21 (dicoordinate P)**	355.7		401.0417	402.4245	459.7021	394.8161	385.2782						
387	**21 (PPh3)**	26.2		21.1868	20.144	22.0591	26.7064	8.0674						
388	**22**	302		354.4451	339.3169	404.4489	349.0021	314.4727						
389	**23**	-127.2		-147.1935	-143.6515	-130.8005	-143.855	-153.8904						
390	**24**	-151.0		-150.9374	-150.9546	-146.7848	-146.8283	-164.8961						
391	**25**	63.5		71.8154	76.4487	80.3067	74.4946	53.8773						
392	**26**	187.9		206.3748	205.5344	257.1634	204.1456	197.4273						
393	**27 weighted avg**	13.9		10.0388	12.2743	15.002	11.0899	6.715						
394	**28**	16.0		13.7145	15.8975	18.3374	14.6627	9.9592						
395	**29**	93		90.3934	108.8143	116.5237	93.8758	86.6008						
396	*anti* -**30**	24.2		37.4039	27.0136	15.1711	38.9878	5.8089						
397	*syn* -**30 weighted average**	11.3		24.4256	16.0322	4.8701	27.229	-6.0322						
398	*anti* -**30[O] weighted average**	54.1		48.9595	46.2708	47.3538	54.4916	33.5857						
399	*syn* -**30[O] weighted average**	61.8		54.5419	51.0032	51.9481	59.6907	39.4587						
400	*anti* -**31**	-24.4		-16.4518	-34.8992	-38.3655	-9.6549	-47.8378						
401	*syn* -**31**	-21.8		-3.5806	-26.0129	-28.6482	3.0787	-38.2265						
402	**32 weighted avg**	-181		-70.6414	-194.2897	-216.2538	-69.5538	-142.9418						
403	**33**	-79		-93.1869	-86.6177	-90.0211	-89.7104	-111.2909						
404	**34**	-14		-6.7491	-14.3941	-17.411	-3.3642	-36.2362						
405	**33[O]**	38		28.7704	32.7963	36.7448	34.2062	12.0373						
406	**34[O]**	26		11.9609	13.8834	18.5429	17.4563	-2.5613						
407	**See Notes for Table 6; values in red taken from Latypov paper (see text).**													
408														
409														
410	**Table S9. Scaled chemical shifts (eq 3 in text) from best fit parameters in Table S4 and unscaled calculations in Table S8; deviation from experimental**													
411	**Functional for Optimization**			**B3LYP**		**M06-2X**		**M06-2X**		**B3LYP**		**Latypov PBE0 (1)**		
412	**Functional for NMR**			**B3LYP**		**B3LYP**		**M06-2X**		**B3LYP**		**Latypov PBE0 (1)**		
413	**Compound/NMR basis set**	**Exp vs H3PO4**		**6-311+G(2d,p)**	**Deviation**	**6-311+G(2d,p)**	**Deviation**	**6-311+G(2d,p)**	**Deviation**	**IGLO-III**	**Deviation**	**6-311G(2d,2p)**	**Deviation**	
414	**1a P(A) weighted avg (5)**	39.6		46.79	7.19	48.44	8.84	56.07	16.47	45.16	5.56	40.31	0.71	
415	**1a P(B) weighted avg (5)**	297.6		286.32	11.28	288.08	9.52	338.89	41.29	280.04	17.56	302.96	5.36	
416	**2**	-110		-109.08	0.92	-108.75	1.25	-106.19	3.81	-103.44	6.56	-107.20	2.80	
417	**3**	-54.5		-50.75	3.75	-50.08	4.42	-47.07	7.43	-47.79	6.71	-50.50	4.00	
418	**4**	24		23.81	0.19	22.35	1.65	26.08	2.08	25.38	1.38	34.30	10.30	
419	**5 P(A)**	18.3		10.23	8.07	12.96	5.34	20.29	1.99	11.43	6.87	18.80	0.50	
420	**5 P(B)**	27.6		17.95	9.65	22.17	5.43	28.16	0.56	19.16	8.44	27.40	0.20	
421	**6**	-10.2		2.97	13.17	0.10	10.30	-5.71	4.49	4.54	14.74	-8.10	2.10	
422	**7**	38.7		45.00	6.30	39.25	0.55	44.83	6.13	42.69	3.99	47.70	9.00	

	A	B	C	D	E	F	G	H	I	J	K	L	M	N
423	**8 P(A)**	76.7		74.92	1.78	74.92	1.78	75.30	1.40	77.66	0.96	72.90	3.80	
424	**8 P(B)**	-157.7		-110.60	47.10	-121.78	35.92	-115.48	42.22	-112.44	45.26	-147.30	10.40	
425	**9 P(A)**	-29.7		-4.81	24.89	-19.58	10.12	-24.36	5.34	-3.90	25.80	-25.70	4.00	
426	**9 P(B)**	100.5		90.71	9.79	85.50	15.00	94.46	6.04	91.65	8.85	91.70	8.80	
427	**9 P(C)**	-10.6		15.44	26.04	15.06	25.66	13.38	23.98	14.48	25.08	6.70	17.30	
428	**9 P(D)**	75.7		64.31	11.39	65.39	10.31	72.51	3.19	66.07	9.63	68.10	7.60	
429	**10 P(A)**	-22.6		0.95	23.55	-5.94	16.66	-9.78	12.82	2.58	25.18	-20.60	2.00	
430	**10 P(B)**	84.1		105.77	21.67	93.30	9.20	99.12	15.02	104.51	20.41	82.60	1.50	
431	**11 weighted avg**	152.9		156.91	4.01	153.31	0.41	152.11	0.79	154.14	1.24	166.46	13.56	
432	**12 weighted avg**	139.3		141.97	2.67	138.47	0.83	139.33	0.03	140.56	1.26	150.60	11.30	
433	**13**	133.0		133.79	0.79	125.38	7.62	125.00	8.00	133.15	0.15	128.82	4.18	
434	**14 weighted avg**	136.7		144.81	8.11	128.83	7.87	131.97	4.73	143.50	6.80	138.81	2.11	
435	**15**	132.2		129.33	2.87	126.35	5.85	124.69	7.51	127.92	4.28	137.54	5.34	
436	**16 weighted avg**	138.5		142.14	3.64	137.20	1.30	134.81	3.69	139.92	1.42	150.32	11.82	
437	**17 weighted avg**	125.8		128.57	2.77	126.19	0.39	124.92	0.88	127.07	1.27	133.66	7.86	
438	**18 weighted avg**	145.3		146.72	1.42	144.47	0.83	142.28	3.02	143.95	1.35	152.85	7.55	
439	**19**	169		181.26	12.26	178.91	9.91	172.31	3.31	179.51	10.51	186.03	17.03	
440	**20**	153		147.82	5.18	146.41	6.59	148.74	4.26	143.62	9.38	169.21	16.21	
441	**21 (dicoordinate P)**	355.7		350.92	4.78	355.18	0.52	398.14	42.44	344.81	10.89	372.37	16.67	
442	**21 (PPh3)**	26.2		20.65	5.55	19.18	7.02	23.78	2.42	23.26	2.94	20.90	5.30	
443	**22**	302		310.41	8.41	299.71	2.29	350.87	48.87	304.79	2.79	306.40	4.40	
444	**23**	-127.2		-125.75	1.45	-124.78	2.42	-106.97	20.23	-125.73	1.47	-130.01	2.81	
445	**24**	-151.0		-129.00	22.00	-131.20	19.80	-120.64	30.36	-128.32	22.68	-140.26	10.74	
446	**25**	63.5		64.67	1.17	68.67	5.17	73.61	10.11	65.00	1.50	63.58	0.08	
447	**26**	187.9		181.67	6.23	182.13	5.77	224.89	36.99	178.26	9.64	197.34	9.44	
448	**27 weighted avg**	13.9		10.96	2.94	12.27	1.63	17.75	3.85	9.62	4.28	19.64	5.74	
449	**28**	16.0		14.15	1.85	15.45	0.55	20.60	4.60	12.74	3.26	22.66	6.66	
450	**29**	93		80.82	12.18	97.12	4.12	104.59	11.59	81.93	11.07	94.07	1.07	
451	*anti* -**30**	24.2		34.75	10.55	25.22	1.02	17.89	6.31	33.99	9.79	18.79	5.41	
452	*syn* -**30 weighted average**	11.3		23.47	12.17	15.57	4.27	9.08	2.22	23.72	12.42	7.76	3.54	
453	*anti* -**30[O] weighted average**	54.1		44.80	9.30	42.15	11.95	45.42	8.68	47.53	6.57	44.68	9.42	
454	*syn* -**30[O] weighted average**	61.8		49.65	12.15	46.31	15.49	49.35	12.45	52.07	9.73	50.15	11.65	
455	*anti* -**31**	-24.4		-12.07	12.33	-29.20	4.80	-27.90	3.50	-8.50	15.90	-31.19	6.79	
456	*syn* -**31**	-21.8		-0.88	20.92	-21.39	0.41	-19.59	2.21	2.62	24.42	-22.24	0.44	
457	**32 weighted avg**	-181		-59.19	121.81	-169.29	11.71	-180.07	0.93	-60.82	120.18	-119.81	61.19	
458	**33**	-79		-78.79	0.21	-74.65	4.35	-72.09	6.91	-78.43	0.57	-90.31	11.31	
459	**34**	-14		-3.64	10.36	-11.17	2.83	-9.98	4.02	-3.01	10.99	-20.38	6.38	
460	**33[O]**	38		27.24	10.76	30.30	7.70	36.34	1.66	29.81	8.19	24.60	13.40	
461	**34[O]**	26		12.63	13.37	13.68	12.32	20.77	5.23	15.18	10.82	11.00	15.00	
462	**MAD**				11.89		7.08		10.33		11.89		8.22	
463	**RMSD**				21.78		9.93		16.26		21.65		12.28	
464	**See Notes for previous tables; values in red taken from Latypov paper (see text).**													
465	(1) In order to reproduce the reported scaled values from the Latypov group (where P(calcd) vs P(exp) was plotted),													
466	P(scaled) = (P(calcd)-b)/m where m = 1.073232 ± 0.020120, b = -14.362193 ± 2.652406, and P(calcd) = 298.8299-P(absolute)													
467														
468														
469														
470														
471														
472														

	A	B	C	D	E	F	G	H	I	J	K	L	M	N
473	**Table S10. Absolute chemical shifts for tri and tetracoordinate phosphorus compounds for screened functionals**													
474	**Functional for Optimization**		**M06-2X**	**M06-2X**	**M06-2X**	**M06-2X**	**ωB97X-D**							
475	**Functional for NMR (1)**		**M06-L**	**TPSSTPSS (2)**	**PBE0**	**wB97X-D**	**ωB97X-D**							
476	**Compound/Basis set**	**Exp vs H3PO4**	**6-311+G(2d,p)**	**6-311+G(2d,p)**	**6-311+G(2d,p)**	**6-311+G(2d,p)**	**6-311+G(2d,p)**							
477	PH3	-238.00	563.6064	556.6887	580.7466	572.4913	569.1797							
478	PMeH2	-163.50	487.8721	473.5627	491.1340	485.0275	482.005							
479	PMe2H	-98.50	426.0372	407.9983	420.8359	416.1485	414.187							
480	PMe3	-61.58	388.3121	370.9853	381.7086	376.6027	375.1427							
481	PPh3	-5.28	339.0342	314.0300	321.2732	317.1130	317.1543							
482	Methoxyphospholane	133.30	179.4959	147.9373	145.9416	140.0648	135.8291							
483	P(OMe)3 isomer A		185.5287	153.6291	151.0541	145.5038	143.2923							
484	P(OMe)3 isomer B		217.4167	186.5973	182.9062	177.1484	174.8157							
485	P(OMe)3 isomer C		160.1683	127.5436	124.7378	118.9926	116.2499							
486	P(OMe)3 weighted avg (4)	141.41	189.6671	157.9105	155.1678	149.5833	146.1451							
487	PCl3 (exp struc) (5)	219.79	99.3270	56.3322	62.6960	59.5935	59.5935							
488	H3PO4 in water sym isomer	0	333.1821	305.3026	300.9919	296.9293	295.4004							
489	PH4+ BF4- in MeOH	-105.3	428.4728	435.2271	438.4165	432.4315	431.9921							
490	P(OPh)4+ PF6-	-28.0	353.6712	330.8801	322.3659	315.8536	318.2495							
491	O=**P(**OCH2)3P=O **PO**	-18.1	353.8256	328.5296	323.3500	318.316	318.8837							
492	P(OMe)4+ BF4- isomer A		334.0748	308.8857	302.0414	297.2905	297.7621							
493	P(OMe)4+ BF4- isomer B		325.4027	299.8644	293.5573	289.1044	290.6714							
494	P(OMe)4+ BF4- weighted avg	1.9	334.0693	308.8800	302.0360	297.2853	297.7441							
495	(iPrO)2P(O)H isomer A		331.2273	307.4113	299.1369	295.1438	294.6777							
496	(iPrO)2P(O)H isomer B		329.4020	306.7765	298.8187	294.9932	294.5030							
497	(iPrO)2P(O)H isomer C		329.5500	306.9219	298.9244	295.0955	294.4129							
498	(iPrO)2P(O)H isomer D		331.1507	307.3355	299.0534	295.0618	294.7139							
499	(iPrO)2P(O)H weighted avg	4.54	329.5883	306.8879	298.8900	295.0525	294.4981							
500	O=P(OCH2)3**P**=O **PC**	6.4	338.2808	310.6235	308.9085	304.5078	307.5934							
501	(MeO)2P(O)H isomer A		325.7824	303.0401	295.2512	291.5960	291.4923							
502	(MeO)2P(O)H isomer B		326.6625	302.2138	294.3743	290.8523	290.3880							
503	(MeO)2P(O)H isomer C		324.8795	300.6961	293.1599	289.7158	289.3102							
504	(MeO)2P(O)H weighted avg	11.3	325.7227	302.6448	294.8878	291.2727	290.9257							
505	PPh4+ Br-	23.17	320.7062	296.7203	295.1872	289.4164	289.2103							
506	PMe4+ Br-	25.1	309.9795	289.5095	291.7736	287.2706	286.6317							
507	(iPrO)2P(O)Me isomer A		311.6846	283.3256	277.2204	272.4588	273.4050							
508	(iPrO)2P(O)Me isomer B		306.6100	278.3814	272.5344	267.7825	267.4472							
509	(iPrO)2P(O)Me isomer C		311.7389	283.3624	277.2758	272.5070	273.5226							
510	(iPrO)2P(O)Me weighted avg	28.61	306.8810	278.6450	272.7847	268.0322	268.4882							
511	Ph3PO	29.10	314.9165	286.7182	285.3284	281.1367	279.8133							
512	(MeO)2P(O)Me isomer A		302.5451	274.2263	268.7528	264.1806	264.1880							
513	(MeO)2P(O)Me isomer B		302.2524	273.4097	268.2478	263.8064	263.9237							
514	(MeO)2P(O)Me isomer C		308.7816	280.1397	274.4665	269.8989	269.8946							
515	(MeO)2P(O)Me weighted avg	32.3	302.9828	274.6086	269.1394	264.5755	264.4304							
516	Me3PO	38.79	309.7011	281.2279	279.7265	275.6088	275.3962							
517	EtOP(O)Me2 isomer A		291.3956	261.2745	256.8167	252.2154	252.0788							
518	EtOP(O)Me2 isomer B		294.0662	264.0549	259.4254	254.7652	254.5539							
519	EtOP(O)Me2 weighted avg	50.3	291.4220	261.3019	256.8425	252.2406	252.1144							
520	**See Notes for Table S1.**													
521	(1) GIAO exceot as noted													
522	(2) CSGT used for NMR													

Table S11. Calculation of energy-weighted isomer ratios for Table S10 ωB97X-D optimization

	E (au) (1)	Relative E	
**Functional for Optimization**	**ωB97X-D**	**(kcal)**	**Ratio 298.15 K**
**Basis set**	**6-31+G(d,p)**		
P(OMe)3 Isomer			
A	-686.585746	0	0.8551
B	-686.583857	1.1854	0.1156
C	-686.582561	1.9986	0.0293
P(OMe)4+ BF4- isomer			
A	-1226.03242	0	0.9975
B	-1226.026778	3.5404	0.0025
(iPrO)2P(O)H isomer			
A	-804.474215	1.3121	0.0579
B	-804.476306	0	0.5305
C	-804.475881	0.2667	0.3382
D	-804.474439	1.1716	0.0734
(MeO)2P(O)H isomer			
A	-647.335606	0	0.6988
B	-647.333608	1.2538	0.0842
C	-647.334502	0.6928	0.2170
(iPrO)2P(O)Me isomer			
A	-843.771637	1.5267	0.0629
B	-843.77407	0	0.8274
C	-843.772162	1.1973	0.1097
(MeO)2P(O)Me isomer			
A	-686.634325	0	0.8874
B	-686.631886	1.5305	0.0670
C	-686.631522	1.7589	0.0456
EtOP(O)Me2 isomer			
A	-650.711626	0	0.9856
B	-650.707636	2.5038	0.0144

(1) Sum of electronic and thermal Free Energies from *Gaussian* vibrational calculation; see Table S1 notes for solvation.

Gas constant from IUPAC:		1.9872	
E conversion:		627.5095	

Table S12. Chemical shifts (1) for tri and tetracoordinate phosphorus compounds referenced to H3PO4 at 0.00 ppm for screened functionals

**Functional for Optimization**		**M06-2X**	**M06-2X**	**M06-2X**	**M06-2X**	**ωB97X-D**
**Functional for NMR (1)**		**M06-L**	**TPSSTPSS (2)**	**PBE0**	**ωB97X-D**	**ωB97X-D**
**Compound/NMR basis set**	**Exp vs H3PO4**	**6-311+G(2d,p)**	**6-311+G(2d,p)**	**6-311+G(2d,p)**	**6-311+G(2d,p)**	**6-311+G(2d,p)**
PH3	-238.00	-230.4243	-251.3861	-279.7547	-275.5620	-273.7793
PMeH2	-163.50	-154.6900	-168.2601	-190.1421	-188.0982	-186.6046
PMe2H	-98.50	-92.8551	-102.6957	-119.8440	-119.2192	-118.7866
PMe3	-61.58	-55.1300	-65.6827	-80.7167	-79.6734	-79.7423
PPh3	-5.28	-5.8521	-8.7274	-20.2813	-20.1837	-21.7539
Methoxyphospholane	133.30	153.6862	157.3653	155.0503	156.8645	159.5713
P(OMe)3 weighted avg (4)	141.41	143.5150	147.3921	145.8241	147.3460	149.2553
PCl3 (exp struc) (5)	219.79	233.8551	248.9704	238.2959	237.3358	235.8069

	A	B	C	D	E	F	G	H	I	J	K	L	M	N
573	PH4+ BF4- in MeOH	-105.3	-95.2907	-129.9245	-137.4246	-135.5022	-136.5917							
574	P(OPh)4+ PF6-	-28.0	-20.4891	-25.5775	-21.3740	-18.9243	-22.8491							
575	O=**P(**OCH2)3P=O **PO**	-18.1	-20.6435	-23.2270	-22.3581	-21.3867	-23.4833							
576	P(OMe)4+ BF4- weighted avg	1.9	-0.8872	-3.5774	-1.0441	-0.3560	-2.3437							
577	(iPrO)2P(O)H weighted avg	4.54	3.5938	-1.5853	2.1019	1.8768	0.9023							
578	O=P(OCH2)3**P**=O **PC**	6.4	-5.0987	-5.3209	-7.9166	-7.5785	-12.1930							
579	(MeO)2P(O)H weighted avg	11.3	7.4594	2.6578	6.1041	5.6566	4.4747							
580	PPh4+ Br-	23.17	12.4759	8.5823	5.8047	7.5129	6.1901							
581	PMe4+ Br-	25.1	23.2026	15.7931	9.2183	9.6587	8.7687							
582	(iPrO)2P(O)Me weighted avg	28.61	26.3011	26.6576	28.2072	28.8971	26.9122							
583	Ph3PO	29.10	18.2656	18.5844	15.6635	15.7926	15.5871							
584	(MeO)2P(O)Me weighted avg	32.3	30.1993	30.6940	31.8525	32.3538	30.9700							
585	Me3PO	38.79	23.4810	24.0747	21.2654	21.3205	20.0042							
586	EtOP(O)Me2 weighted avg	50.3	41.7601	44.0007	44.1494	44.6887	43.2860							
587	**See Notes for previous tables.**													
588	(1) GIAO exceot as noted													
589	(2) CSGT used for NMR													
590														
591	**Table S13. Linear regressions, P(Exp) vs P(calcd) chemical shifts, ppm, for screened functionals**													
592	**Functional for Optimization**		**M06-2X**	**M06-2X**	**M06-2X**	**M06-2X**	**ωB97X-D**							
593	**Functional for NMR (1)**		**M06-L**	**TPSSTPSS (2)**	**PBE0**	**ωB97X-D**	**ωB97X-D**							
594	**Basis set for NMR**		**6-311+G(2d,p)**	**6-311+G(2d,p)**	**6-311+G(2d,p)**	**6-311+G(2d,p)**	**6-311+G(2d,p)**							
595	Chi squared		1693.165787	1473.440959	1101.298227	1188.099216	1249.526454							
596	slope		0.997613	0.921300	0.880229	0.885284	0.886764							
597	std dev		0.02044604	0.01760469	0.01452809	0.01517969	0.01559554							
598	intercept		-0.390792	3.823927	8.355962	7.586321	8.371542							
599	std dev		1.961951	1.830608	1.586399	1.646811	1.689813							
600	Correlation coefficient		0.995826	0.996369	0.997287	0.997073	0.996921							
601	(1) GIAO exceot as noted													
602	(2) CSGT used for NMR													
603														
604														
605	**Table S14. Scaled chemical shifts (eq 3 in text) from best fit parameters in Table S13 and unscaled calculations in Table S12, deviation from experimental**													
606	**Functional for Optimization**		**M06-2X**		**M06-2X**		**M06-2X**		**M06-2X**		**ωB97X-D**			
607	**Functional for NMR (1)**		**M06-L**		**TPSSTPSS (2)**		**PBE0**		**ωB97X-D**		**ωB97X-D**			
608	**Compound/NMR basis set**	**Exp vs H3PO4**	**6-311+G(2d,p)**	**Deviation**	**6-311+G(2d,p)**	**Deviation**	**6-311+G(2d,p)**	**Deviation**	**6-311+G(2d,p)**	**Deviation**	**6-311+G(2d,p)**	**Deviation**		
609	PH3	-238	-230.27	7.73	-227.78	10.22	-237.89	0.11	-236.36	1.64	-234.41	3.59		
610	PMeH2	-163.5	-154.71	8.79	-151.19	12.31	-159.01	4.49	-158.93	4.57	-157.10	6.40		
611	PMe2H	-98.5	-93.02	5.48	-90.79	7.71	-97.13	1.37	-97.96	0.54	-96.96	1.54		
612	PMe3	-61.58	-55.39	6.19	-56.69	4.89	-62.69	1.11	-62.95	1.37	-62.34	0.76		
613	PPh3	-5.28	-6.23	0.95	-4.22	1.06	-9.50	4.22	-10.28	5.00	-10.92	5.64		
614	Methoxyphospholane	133.3	152.93	19.63	148.80	15.50	144.84	11.54	146.46	13.16	149.87	16.57		
615	P(OMe)3	141.41	142.78	1.37	139.62	1.79	136.71	4.70	138.03	3.38	140.73	0.68		
616	PCl3 (exp struc)	219.79	232.91	13.12	233.20	13.41	218.11	1.68	217.70	2.09	217.48	2.31		
617	PH4+ in MeOH	-105.3	-95.45	9.85	-115.88	10.58	-112.61	7.31	-112.37	7.07	-112.75	7.45		
618	PhO)4P+ PF6-	-28	-20.83	7.17	-19.74	8.26	-10.46	17.54	-9.17	18.83	-11.89	16.11		
619	O=POCH2)3P=O PO	-18.1	-20.99	2.89	-17.58	0.52	-11.32	6.78	-11.35	6.75	-12.45	5.65		
620	P(OMe)4+ BF4-	1.9	-1.28	3.18	0.53	1.37	7.44	5.54	7.27	5.37	6.29	4.39		
621	(iPrO)2P(O)H	4.54	3.19	1.35	2.36	2.18	10.21	5.67	9.25	4.71	9.17	4.63		
622	O=POCH2)3P=O PC	6.4	-5.48	11.88	-1.08	7.48	1.39	5.01	0.88	5.52	-2.44	8.84		

	A	B	C	D	E	F	G	H	I	J	K	L	M	N
623	MeO)2P(O)H	11.3	7.05	4.25	6.27	5.03	13.73	2.43	12.59	1.29	12.34	1.04		
624	Ph4P+Br-/Cl-	23.17	12.06	11.11	11.73	11.44	13.47	9.70	14.24	8.93	13.86	9.31		
625	Me4P Br-/Cl-	25.1	22.76	2.34	18.37	6.73	16.47	8.63	16.14	8.96	16.15	8.95		
626	(iPrO)2P(O)Me	28.61	25.85	2.76	28.38	0.23	33.18	4.57	33.17	4.56	32.24	3.63		
627	Ph3PO	29.1	17.83	11.27	20.95	8.15	22.14	6.96	21.57	7.53	22.19	6.91		
628	(MeO)2P(O)Me	32.3	29.74	2.56	32.10	0.20	36.39	4.09	36.23	3.93	35.83	3.53		
629	Me3PO	38.79	23.03	15.76	26.00	12.79	27.07	11.72	26.46	12.33	26.11	12.68		
630	EtOP(O)Me2	50.3	41.27	9.03	44.36	5.94	47.22	3.08	47.15	3.15	46.76	3.54		
631	**MAD**			**7.21**		**6.72**		**5.83**		**5.94**		**6.10**		
632	**RMSD**			**8.77**		**8.18**		**7.08**		**7.349**		**7.54**		
633	(1) GIAO exceot as noted													
634	(2) CSGT used for NMR													
635														
636														
637	**Table S15. Absolute chemical shifts for Table 2 and 3 phosphorus compounds for screened functionals**													
638	**Functional for Optimization**		**M06-2X**	**M06-2X**	**M06-2X**	**M06-2X**	**ωB97X-D**							
639	**Functional for NMR (1)**		**M06-L**	**TPSSTPSS (2)**	**PBE0**	**ωB97X-D**	**ωB97X-D**							
640	**Compound/NMR Basis set**		**6-311+G(2d,p)**	**6-311+G(2d,p)**	**6-311+G(2d,p)**	**6-311+G(2d,p)**	**6-311+G(2d,p)**							
641	**1a P(A) isomer A**		285.2064	259.0524	262.4327	261.7795	261.9609							
642	**1a P(B) isomer A**		43.9025	12.0193	-11.1916	-22.5126	-21.7125							
643	**1a P(A) isomer B**		282.9665	256.7087	262.4417	262.4710	263.3115							
644	**1a P(B) isomer B**		16.3629	-15.6027	-39.0322	-49.3511	-49.7637							
645	**1a P(A) weighted avg (5)**		285.0142	258.8513	262.4335	261.8388	262.0768							
646	**1a P(B) weighted avg (5)**		41.5397	9.6494	-13.5802	-24.8153	-24.1192							
647	**2**		449.8583	428.6414	433.3340	436.9648	437.2379							
648	**3**		387.2031	368.9777	366.5255	366.4235	368.1762							
649	**4**		313.6841	284.9889	281.3438	276.5208	275.4656							
650	**5 P(A)**		319.4912	298.8970	292.8461	286.2580	288.6388							
651	**5 P(B)**		310.0178	289.2342	283.6445	277.1203	280.1244							
652	**6**		339.9012	313.424	321.7115	317.3686	317.1658							
653	**7**		304.2409	273.3651	274.4774	272.2583	278.7106							
654	**8 P(A)**		261.4967	235.3843	236.3608	229.7845	232.2651							
655	**8 P(B)**		481.1844	458.4066	462.1651	454.5717	456.6064							
656	**9 P(A)**		368.5561	336.4156	344.0799	341.8511	340.3773							
657	**9 P(B)**		252.4136	219.3067	215.4493	210.8994	212.9768							
658	**9 P(C)**		334.8146	307.8336	308.6206	299.0388	304.0902							
659	**9 P(D)**		270.3821	239.7156	237.4539	234.4850	235.3410							
660	**10 P(A)**		346.4482	316.6875	327.0769	326.1998	330.0553							
661	**10 P(B)**		246.4322	214.0522	217.5977	215.8027	217.3011							
662	**11 isomer A**		173.5907	140.165	138.0728	132.1380	133.1156							
663	**11 isomer B**		196.6209	164.2993	161.4696	155.0285	151.3491							
664	**11 isomer C**		162.2448	129.3079	127.6965	121.0378	120.9201							
665	**11 weighted avg**		176.6270	143.3653	141.1780	135.1590	140.0094							
666	**12 isomer A**		193.2216	161.4264	159.5413	153.4960	153.3525							
667	**12 isomer B**		180.2496	147.7865	146.2787	141.0970	140.8546							
668	**12 weighted avg**		190.5135	158.5788	156.7725	150.9075	149.5473							
669	**13**		204.7060	176.0247	172.7088	163.4585	160.9856							
670	**14 isomer A**		200.3035	172.3512	167.9994	158.6002	152.3949							
671	**14 isomer B**		201.1357	173.0094	167.8346	157.3014	153.0320							
672	**14 weighted avg**		200.6161	172.5984	167.9375	158.1124	152.7689							

	A	B	C	D	E	F	G	H	I	J	K	L	M	N
673	**15**		199.5015	170.8921	168.5169	161.8240	161.4088							
674	**16 isomer A**		147.1408	115.4794	113.3857	107.2529	106.4488							
675	**16 isomer B**		190.9898	161.8046	158.7086	152.3863	152.4263							
676	**16 isomer C**		181.6280	152.6112	149.5322	143.1983	146.6148							
677	**16 weighted avg**		188.4422	159.3020	156.2109	149.8855	150.9466							
678	**17 isomer A (cis, chair)**		197.6582	170.7039	169.5882	163.2396	163.6931							
679	**17 isomer B (cis, twist 1)**		199.6715	173.4921	170.6007	164.4606	164.8237							
680	**17 isomer C (cis, twist 2)**		203.7120	178.9537	175.9433	169.4450	169.9377							
681	**17 weighted avg**		198.9161	172.4431	170.2905	164.0558	164.3994							
682	**18 isomer A (trans, chair)**		170.8856	143.3343	140.9575	134.8753	144.2112							
683	**18 isomer B trans, twist 1)**		183.9347	156.2495	154.8942	149.1976	150.6202							
684	**18 isomer C (trans, twist 2)**		199.9684	173.7563	171.2930	165.4042	165.5677							
685	**18 weighted avg**		179.6010	152.4397	150.0533	144.0337	147.5652							
686	**19**		142.2118	109.2051	112.2008	106.4569	108.9480							
687	**20**		177.8259	149.259	146.8650	138.7159	149.7349							
688	**21 (dicoordinate P)**		-22.1576	-44.1016	-90.5691	-117.0433	-116.7150							
689	**21 (PPh3)**		315.5961	292.7682	293.3479	285.3530	285.2988							
690	**22**		46.5668	25.7194	-24.8088	-63.7537	-59.8143							
691	**23**		476.0607	457.2848	458.3569	452.5472	456.9719							
692	**24**		485.4962	463.8392	467.7830	460.3060	463.9938							
693	**25**		264.9480	241.5298	238.0854	231.0150	233.5703							
694	**26**		153.8248	130.3636	106.2664	94.1408	94.0861							
695	**27 isomer A**		322.0260	295.8689	289.7603	284.9658	284.6682							
696	**27 isomer B**		321.5723	295.7261	288.6673	283.9616	285.9116							
697	**27 weighted avg**		322.0238	295.8682	289.7549	284.9609	284.6805							
698	**28**		318.1561	292.0633	286.2103	281.5510	281.7333							
699	**29**		236.2054	205.8746	197.6134	189.8068	188.6634							
700	*anti* -**30**		317.5516	288.1634	294.8167	289.0113	286.4126							
701	*syn* -**30 isomer A**		326.5582	298.2647	305.4482	299.1760	297.9573							
702	*syn* -**30 isomer B**		318.1882	290.7760	296.4434	290.1311	287.8389							
703	*syn* -**30 weighted average**		326.1185	297.8713	304.9752	298.7009	297.5255							
704	*anti* -**30[O]**		296.9067	264.7586	262.4712	258.0019	257.4716							
705	*syn* -**30[O] isomer A**		288.2432	255.3698	254.0472	249.8109	249.2173							
706	*syn* -**30[O] isomer B**		297.6188	267.0858	264.4270	259.6976	258.9144							
707	*syn* -**30[O] weighted average**		291.3496	259.2516	257.4863	253.0866	251.8866							
708	*anti* -**31**		369.1202	341.5652	343.7041	342.2729	346.1961							
709	*syn* -**31**		360.9197	332.8794	334.8087	332.9530	336.0561							
710	**32 isomer A**		505.7219	485.9662	502.6176	504.9365	498.1401							
711	**32 isomer B**		519.0955	502.6133	520.4951	523.0438	514.4876							
712	**32 weighted avg**		508.9662	490.0046	506.9544	509.3291	501.7277							
713	**33**		414.7308	399.6489	403.1816	390.2889	391.1944							
714	**34**		351.1976	329.9672	332.6139	323.1831	319.9407							
715	**33[O]**		306.4692	278.3290	275.9182	269.6592	270.1286							
716	**34[O]**		322.4085	297.2982	293.6983	287.4481	287.2834							
717	**See Notes for previous tables.**													
718	(1) GIAO exceot as noted													
719	(2) CSGT used for NMR													
720														
721														
722														

	A	B	C	D	E	F	G	H	I	J	K	L	M	N
723	**Table S16. Unscaled chemical shifts for 1a-34[O] referenced to H3PO4 at 0.00 ppm for screened functionals**													
724	**Functional for Optimization**		**M06-2X**	**M06-2X**	**M06-2X**	**M06-2X**	**ωB97X-D**							
725	**Functional for NMR (1)**		**M06-L**	**TPSSTPSS (2)**	**PBE0**	**ωB97X-D**	**ωB97X-D**							
726	**Compound/NMR basis set**	**Exp vs H3PO4**	**6-311+G(2d,p)**	**6-311+G(2d,p)**	**6-311+G(2d,p)**	**6-311+G(2d,p)**	**6-311+G(2d,p)**							
727	**1a P(A) weighted avg (5)**	39.6	48.1679	46.4513	38.5584	35.0905	33.3236							
728	**1a P(B) weighted avg (5)**	297.6	291.6424	295.6532	314.5721	321.7446	319.5196							
729	**2**	-110	-116.6762	-123.3388	-132.3421	-140.0355	-141.8375							
730	**3**	-54.5	-54.0210	-63.6751	-65.5336	-69.4942	-72.7758							
731	**4**	24	19.4980	20.3137	19.6481	20.4085	19.9348							
732	**5 P(A)**	18.3	13.6910	6.4056	8.1459	10.6714	6.7617							
733	**5 P(B)**	27.6	23.1643	16.0684	17.3474	19.8090	15.2760							
734	**6**	-10.2	-6.7191	-8.1214	-20.7196	-20.4393	-21.7654							
735	**7**	38.7	28.9413	31.9376	26.5146	24.6710	16.6899							
736	**8 P(A)**	76.7	71.6854	69.9183	64.6311	67.1448	63.1353							
737	**8 P(B)**	-157.7	-148.0023	-153.1040	-161.1732	-157.6424	-161.2060							
738	**9 P(A)**	-29.7	-35.3740	-31.1130	-43.0880	-44.9218	-44.9769							
739	**9 P(B)**	100.5	80.7685	85.9959	85.5426	86.0299	82.4236							
740	**9 P(C)**	-10.6	-1.6325	-2.5310	-7.6287	-2.1095	-8.6898							
741	**9 P(D)**	75.7	62.8000	65.5870	63.5380	62.4443	60.0594							
742	**10 P(A)**	-22.6	-13.2661	-11.3849	-26.0850	-29.2705	-34.6549							
743	**10 P(B)**	84.1	86.7499	91.2504	83.3942	81.1266	78.0993							
744	**11 weighted avg**	152.9	156.5551	161.9373	159.8139	161.7703	155.3910							
745	**12 weighted avg**	139.3	142.6686	146.7238	144.2194	146.0218	145.8531							
746	**13**	133.0	128.4761	129.2779	128.2831	133.4708	134.4148							
747	**14 weighted avg**	136.7	132.5660	132.7042	133.0544	138.8169	142.6315							
748	**15**	132.2	133.6806	134.4105	132.4750	135.1053	133.9916							
749	**16 weighted avg**	138.5	144.7399	146.0006	144.7810	147.0438	144.4538							
750	**17 weighted avg**	125.8	134.2660	132.8595	130.7014	132.8735	131.0010							
751	**18 weighted avg**	145.3	153.5811	152.8629	150.9386	152.8956	147.8352							
752	**19**	169	190.9703	196.0975	188.7911	190.4724	186.4524							
753	**20**	153	155.3562	156.0436	154.1269	158.2134	145.6655							
754	**21 (dicoordinate P)**	355.7	355.3397	349.4042	391.5610	413.9726	412.1154							
755	**21 (PPh3)**	26.2	17.5861	12.5345	7.6440	11.5764	10.1016							
756	**22**	302	286.6153	279.5832	325.8007	360.6830	355.2147							
757	**23**	-127.2	-142.8786	-151.9822	-157.3650	-155.6179	-161.5715							
758	**24**	-151.0	-152.3141	-158.5366	-166.7911	-163.3767	-168.5934							
759	**25**	63.5	68.2341	63.7728	62.9065	65.9143	61.8301							
760	**26**	187.9	179.3573	174.9390	194.7255	202.7885	201.3143							
761	**27 weighted avg**	13.9	11.1583	9.4344	11.2370	11.9684	10.7199							
762	**28**	16.0	15.0260	13.2393	14.7816	15.3783	13.6671							
763	**29**	93	96.9767	99.4280	103.3785	107.1225	106.7370							
764	*anti* -**30**	24.2	15.6305	17.1392	6.1752	7.9180	8.9878							
765	*syn* -**30 weighted average**	11.3	7.0636	7.4313	-3.9833	-1.7716	-2.1251							
766	*anti* -**30[O]**	54.1	36.2754	40.5440	38.5207	38.9274	37.9288							
767	*syn* -**30[O] weighted average**	61.8	41.8325	46.0510	43.5056	43.8427	43.5138							
768	*anti* -**31**	-24.4	-35.9381	-36.2626	-42.7122	-45.3436	-50.7957							
769	*syn* -**31**	-21.8	-27.7376	-27.5768	-33.8168	-36.0237	-40.6557							
770	**32 weighted avg**	-181	-175.7841	-184.7020	-205.9625	-212.3998	-206.3273							
771	**33**	-79	-81.5487	-94.3463	-102.1897	-93.3596	-95.7940							
772	**34**	-14	-18.0155	-24.6646	-31.6220	-26.2538	-24.5403							

	A	B	C	D	E	F	G	H	I	J	K	L	M	N
773	**33[O]**	38	26.7129	26.9736	25.0737	27.2701	25.2718							
774	**34[O]**	26	10.7736	8.0044	7.2936	9.4812	8.1170							
775	**See Notes for previous tables.**													
776	(1) GIAO exceot as noted													
777	(2) CSGT used for NMR													
778														
779														
780	**Table S17. Scaled chemical shifts (eq 3 in text) from best fit parameters in Table S13 and unscaled calculations in Table S16; deviation from experimental**													
781	**Functional for Optimization**		**M06-2X**		**M06-2X**		**M06-2X**		**M06-2X**		**ωB97X-D**			
782	**Functional for NMR (1)**		**M06-L**		**TPSSTPSS (2)**		**PBE0**		**ωB97X-D**		**ωB97X-D**			
783	**Compound/NMR basis set (2)**	**Exp vs H3PO4**	**6-311+G(2d,p)**	**Deviation**	**6-311+G(2d,p)**	**Deviation**	**6-311+G(2d,p)**	**Deviation**	**6-311+G(2d,p)**	**Deviation**	**6-311+G(2d,p)**	**Deviation**		
784	**1a P(A) weighted avg**	39.6	47.66	8.06	46.62	7.02	42.30	2.70	38.65	0.95	37.92	1.68		
785	**1a P(B) weighted avg**	297.6	290.56	7.04	276.21	21.39	285.25	12.35	292.42	5.18	291.71	5.89		
786	**2**	-110	-116.79	6.79	-109.81	0.19	-108.14	1.86	-116.38	6.38	-117.40	7.40		
787	**3**	-54.5	-54.28	0.22	-54.84	0.34	-49.33	5.17	-53.94	0.56	-56.16	1.66		
788	**4**	24	19.06	4.94	22.54	1.46	25.65	1.65	25.65	1.65	26.05	2.05		
789	**5 P(A)**	18.3	13.27	5.03	9.73	8.57	15.53	2.77	17.03	1.27	14.37	3.93		
790	**5 P(B)**	27.6	22.72	4.88	18.63	8.97	23.63	3.97	25.12	2.48	21.92	5.68		
791	**6**	-10.2	-7.09	3.11	-3.66	6.54	-9.88	0.32	-10.51	0.31	-10.93	0.73		
792	**7**	38.7	28.48	10.22	33.25	5.45	31.69	7.01	29.43	9.27	23.17	15.53		
793	**8 P(A)**	76.7	71.12	5.58	68.24	8.46	65.25	11.45	67.03	9.67	64.36	12.34		
794	**8 P(B)**	-157.7	-148.04	9.66	-137.23	20.47	-133.51	24.19	-131.97	25.73	-134.58	23.12		
795	**9 P(A)**	-29.7	-35.68	5.98	-24.84	4.86	-29.57	0.13	-32.18	2.48	-31.51	1.81		
796	**9 P(B)**	100.5	80.18	20.32	83.05	17.45	83.65	16.85	83.75	16.75	81.46	19.04		
797	**9 P(C)**	-10.6	-2.02	8.58	1.49	12.09	1.64	12.24	5.72	16.32	0.67	11.27		
798	**9 P(D)**	75.7	62.26	13.44	64.25	11.45	64.28	11.42	62.87	12.83	61.63	14.07		
799	**10 P(A)**	-22.6	-13.63	8.97	-6.66	15.94	-14.60	8.00	-18.33	4.27	-22.36	0.24		
800	**10 P(B)**	84.1	86.15	2.05	87.89	3.79	81.76	2.34	79.41	4.69	77.63	6.47		
801	**11 weighted avg**	152.9	155.79	2.89	153.02	0.12	149.03	3.87	150.80	2.10	146.17	6.73		
802	**12 weighted avg**	139.3	141.94	2.64	139.00	0.30	135.30	4.00	136.86	2.44	137.71	1.59		
803	**13**	133.0	127.78	5.22	122.93	10.07	121.27	11.73	125.75	7.25	127.57	5.43		
804	**14 weighted avg**	136.7	131.86	4.84	126.08	10.62	125.47	11.23	130.48	6.22	134.85	1.85		
805	**15**	132.2	132.97	0.77	127.66	4.54	124.96	7.24	127.19	5.01	127.19	5.01		
806	**16 weighted avg**	138.5	144.00	5.50	138.33	0.17	135.80	2.70	137.76	0.74	136.47	2.03		
807	**17 weighted avg**	125.8	133.55	7.75	126.23	0.43	123.40	2.40	125.22	0.58	124.54	1.26		
808	**18 weighted avg**	145.3	152.82	7.52	144.66	0.64	141.22	4.08	142.94	2.36	139.47	5.83		
809	**19**	169	190.12	21.12	184.49	15.49	174.54	5.54	176.21	7.21	173.71	4.71		
810	**20**	153	154.59	1.59	147.59	5.41	144.02	8.98	147.65	5.35	137.54	15.46		
811	**21 (dicoordinate P)**	355.7	354.10	1.60	325.73	29.97	353.02	2.68	374.07	18.37	373.82	18.12		
812	**21 (PPh3)**	26.2	17.15	9.05	15.37	10.83	15.08	11.12	17.83	8.37	17.33	8.87		
813	**22**	302	285.54	16.46	261.40	40.60	295.14	6.86	326.89	24.89	323.36	21.36		
814	**23**	-127.2	-142.93	15.73	-136.20	9.00	-130.16	2.96	-130.18	2.98	-134.90	7.70		
815	**24**	-151.0	-152.34	1.34	-142.24	8.76	-138.46	12.54	-137.05	13.95	-141.13	9.87		
816	**25**	63.5	67.68	4.18	62.58	0.92	63.73	0.23	65.94	2.44	63.20	0.30		
817	**26**	187.9	178.54	9.36	165.00	22.90	179.76	8.14	187.11	0.79	186.89	1.01		
818	**27 weighted avg**	13.9	10.74	3.16	12.52	1.38	18.25	4.35	18.18	4.28	17.88	3.98		
819	**28**	16.0	14.60	1.40	16.02	0.02	21.37	5.37	21.20	5.20	20.49	4.49		
820	**29**	93	96.35	3.35	95.43	2.43	99.35	6.35	102.42	9.42	103.02	10.02		
821	*anti* -**30**	24.2	15.20	9.00	19.61	4.59	13.79	10.41	14.60	9.60	16.34	7.86		
822	*syn* -**30 weighted average**	11.3	6.66	4.64	10.67	0.63	4.85	6.45	6.02	5.28	6.49	4.81		

	A	B	C	D	E	F	G	H	I	J	K	L	M	N
823	*anti* -**30[O]**	54.1	35.80	18.30	41.18	12.92	42.26	11.84	42.05	12.05	42.01	12.09		
824	*syn* -**30[O] weighted average**	61.8	41.34	20.46	46.25	15.55	46.65	15.15	46.40	15.40	46.96	14.84		
825	*anti* -**31**	-24.4	-36.24	11.84	-29.58	5.18	-29.24	4.84	-32.56	8.16	-36.67	12.27		
826	*syn* -**31**	-21.8	-28.06	6.26	-21.58	0.22	-21.41	0.39	-24.30	2.50	-27.68	5.88		
827	**32 weighted avg**	-181	-175.76	5.24	-166.34	14.66	-172.94	8.06	-180.45	0.55	-174.59	6.41		
828	**33**	-79	-81.74	2.74	-83.10	4.10	-81.59	2.59	-75.06	3.94	-76.58	2.42		
829	**34**	-14	-18.36	4.36	-18.90	4.90	-19.48	5.48	-15.66	1.66	-13.39	0.61		
830	**33[O]**	38	26.26	11.74	28.67	9.33	30.43	7.57	31.73	6.27	30.78	7.22		
831	**34[O]**	26	10.36	15.64	11.20	14.80	14.78	11.22	15.98	10.02	15.57	10.43		
832	**MAD**			**7.51**		**8.67**		**6.89**		**6.80**		**7.36**		
833	**RMSD**			**9.27**		**12.06**		**8.452**		**9.13**		**9.37**		
834	**See Notes for previous tables.**													
835	(1) GIAO exceot as noted													
836	(2) CSGT used for NMR													

Table S18. Coordinates of optimized structures for tricoordinate and tetracoordinate phosphorus compounds, B3LYP/6-31+G(d,p).

PH_3 B3LYP/6-31+G(d,p), chloroform IEFPCM:
Sum of electronic and thermal Free Energies= -343.143965

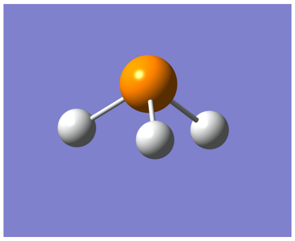

Center Number	Atomic Number	Atomic Type	Coordinates (Angstroms) X	Y	Z
1	15	0	0.000000	0.000000	0.126741
2	1	0	0.000000	1.201935	-0.633704
3	1	0	-1.040907	-0.600968	-0.633704
4	1	0	1.040907	-0.600968	-0.633704

$PMeH_2$ B3LYP/6-31+G(d,p), chloroform IEFPCM:
Sum of electronic and thermal Free Energies= -382.440065

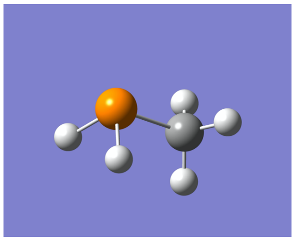

Center Number	Atomic Number	Atomic Type	Coordinates (Angstroms) X	Y	Z
1	15	0	0.668912	0.000000	-0.124050
2	1	0	0.945451	-1.039399	0.808394
3	1	0	0.945440	1.039404	0.808391
4	6	0	-1.198237	-0.000001	0.025651
5	1	0	-1.592186	0.883570	-0.484675
6	1	0	-1.592202	-0.883483	-0.484818
7	1	0	-1.550758	-0.000080	1.059554

PMe_2H B3LYP/6-31+G(d,p), chloroform IEFPCM:
Sum of electronic and thermal Free Energies= -421.737406

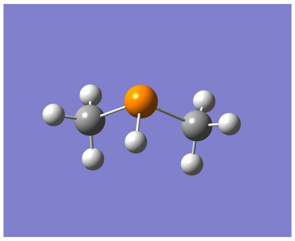

Center Number	Atomic Number	Atomic Type	Coordinates (Angstroms) X	Y	Z
1	15	0	0.000000	-0.655003	-0.113891
2	1	0	0.000000	-1.099451	1.239581
3	6	0	1.438976	0.529065	0.022632
4	1	0	2.364405	-0.039467	0.155867
5	1	0	1.525233	1.094003	-0.910730
6	1	0	1.331177	1.233320	0.853460
7	6	0	-1.438976	0.529065	0.022632
8	1	0	-1.525226	1.094009	-0.910726
9	1	0	-2.364406	-0.039467	0.155857
10	1	0	-1.331181	1.233314	0.853466

PMe_3 B3LYP/6-31+G(d,p), chloroform IEFPCM:
Sum of electronic and thermal Free Energies= -461.036573

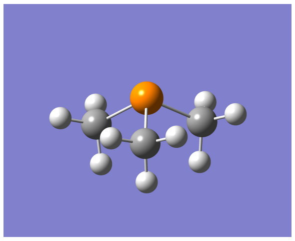

Center Number	Atomic Number	Atomic Type	Coordinates (Angstroms) X	Y	Z
1	15	0	0.000030	-0.000002	-0.596886
2	6	0	-0.999898	-1.308888	0.276519
3	1	0	-0.643756	-2.300511	-0.020308
4	1	0	-2.049882	-1.226843	-0.021903
5	6	0	-0.633733	1.520273	0.276588
6	1	0	-0.038376	2.388937	-0.022154
7	1	0	-0.590113	1.419496	1.367815
8	1	0	-0.934091	-1.221148	1.367761
9	1	0	-1.670821	1.706894	-0.019851
10	6	0	1.633606	-0.211389	0.276387

11	1	0	1.524817	-0.198734	1.367661
12	1	0	2.313858	0.593321	-0.020221
13	1	0	2.088064	-1.161352	-0.022480

PPh_3 B3LYP/6-31+G(d,p), chloroform IEFPCM:
Sum of electronic and thermal Free Energies= -1036.126559

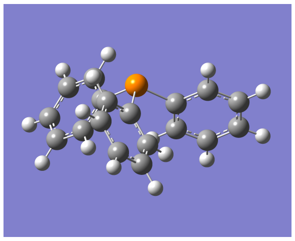

Center Number	Atomic Number	Atomic Type	Coordinates (Angstroms) X	Y	Z
1	15	0	0.000928	-0.000331	-1.202020
2	6	0	-0.615916	1.555699	-0.402173
3	6	0	-1.710673	2.195108	-1.011737
4	6	0	-0.042541	2.146540	0.735280
5	6	0	-2.233786	3.378592	-0.485570
6	1	0	-2.156186	1.764820	-1.905498
7	6	0	-0.556993	3.339103	1.254995
8	1	0	0.809602	1.679157	1.218570
9	6	0	-1.655500	3.955913	0.649803
10	1	0	-3.082968	3.854381	-0.967903
11	1	0	-0.098955	3.783196	2.134490
12	1	0	-2.053866	4.881662	1.054891
13	6	0	1.656558	-0.244530	-0.401507
14	6	0	1.879757	-1.031524	0.739785
15	6	0	2.759238	0.377398	-1.014854
16	6	0	3.169568	-1.183760	1.259471
17	1	0	1.047565	-1.530741	1.226001
18	6	0	4.045609	0.237201	-0.488792
19	1	0	2.610692	0.974320	-1.911536
20	6	0	4.254629	-0.547278	0.650340
21	1	0	3.323792	-1.798615	2.141912
22	1	0	4.883518	0.729622	-0.974145
23	1	0	5.255464	-0.666444	1.055252
24	6	0	-1.039028	-1.311250	-0.401166
25	6	0	-1.055690	-2.576241	-1.016193
26	6	0	-1.828542	-1.111197	0.742679
27	6	0	-1.821000	-3.619448	-0.489610
28	1	0	-0.467147	-2.745812	-1.914685
29	6	0	-2.605834	-2.151315	1.263031

30	1	0	-1.841405	-0.141566	1.230329
31	6	0	-2.601377	-3.408331	0.651936
32	1	0	-1.816797	-4.590594	-0.976421
33	1	0	-3.212594	-1.977614	2.147456
34	1	0	-3.205506	-4.214891	1.057277

Methoxyphospholane B3LYP/6-31+G(d,p), chloroform IEFPCM:
Sum of electronic and thermal Free Energies= -685.536554

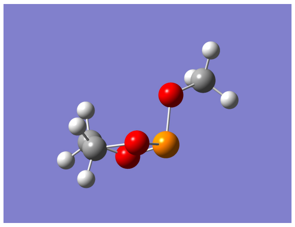

Center Number	Atomic Number	Atomic Type	Coordinates (Angstroms) X	Y	Z
1	8	0	-0.584264	1.089297	-0.652321
2	6	0	-1.536064	1.013620	0.438134
3	6	0	-1.984100	-0.449039	0.468172
4	1	0	-1.043220	1.308609	1.370117
5	1	0	-2.353463	1.702278	0.218385
6	1	0	-2.256823	-0.781736	1.472111
7	1	0	-2.813935	-0.634163	-0.221117
8	15	0	0.322012	-0.311601	-0.726006
9	8	0	-0.846346	-1.229950	0.022853
10	6	0	2.709894	0.217218	0.347566
11	1	0	3.310540	-0.213658	1.150468
12	1	0	3.074574	-0.156717	-0.616165
13	1	0	2.798936	1.307734	0.370208
14	8	0	1.344963	-0.177988	0.582323

$P(OMe)_3$ isomer A B3LYP/6-31+G(d,p), chloroform IEFPCM:
Sum of electronic and thermal Free Energies= -686.722417

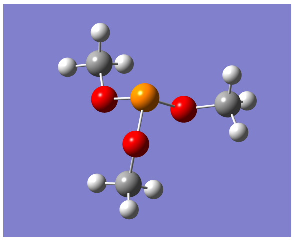

Center Number	Atomic Number	Atomic Type	Coordinates (Angstroms) X	Y	Z
1	15	0	-0.073306	-0.069752	-0.717485
2	6	0	1.587586	-1.968987	0.164331
3	1	0	1.579160	-2.755628	0.921020
4	1	0	1.437907	-2.418448	-0.824369
5	1	0	2.555312	-1.456917	0.184856
6	6	0	1.065964	1.991680	0.643421
7	1	0	1.839374	2.733489	0.439394
8	1	0	0.148659	2.487987	0.967976
9	1	0	1.408227	1.301137	1.418572
10	6	0	-2.514531	-0.432403	0.280271
11	1	0	-3.346364	0.165067	0.656689
12	1	0	-2.799654	-0.882007	-0.678532
13	1	0	-2.276305	-1.222807	0.998447
14	8	0	0.839976	1.288640	-0.600098
15	8	0	0.524882	-1.057228	0.493678
16	8	0	-1.399962	0.462670	0.125180

$P(OMe)_3$ isomer B B3LYP/6-31+G(d,p), chloroform IEFPCM:
Sum of electronic and thermal Free Energies= -686.720492

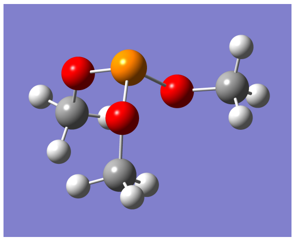

Center Number	Atomic Number	Atomic Type	Coordinates (Angstroms) X	Y	Z
1	6	0	2.319720	-0.752042	0.431076
2	8	0	0.884308	-0.827059	0.404468
3	15	0	0.092585	-0.180758	-0.921005
4	8	0	-1.414007	-0.722956	-0.544907
5	6	0	-1.963760	-0.955708	0.769837
6	8	0	0.103976	1.437520	-0.565939
7	1	0	2.655386	-1.416694	1.228968
8	1	0	2.752873	-1.083338	-0.519806
9	1	0	2.654048	0.268945	0.644346
10	1	0	-2.267189	-0.011678	1.230844
11	1	0	-2.842770	-1.586270	0.626149
12	1	0	-1.239555	-1.466388	1.407125

13	6	0	-0.202785	2.025430	0.714202
14	1	0	-1.284159	2.156469	0.815654
15	1	0	0.281810	3.003413	0.739740
16	1	0	0.176719	1.408192	1.533206

$P(OMe)_3$ isomer C B3LYP/6-31+G(d,p), chloroform IEFPCM:
Sum of electronic and thermal Free Energies= -686.720306

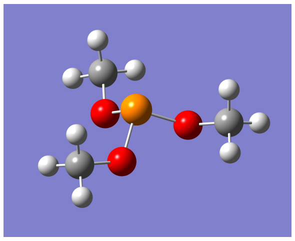

Center Number	Atomic Number	Atomic Type	Coordinates (Angstroms) X	Y	Z
1	15	0	0.000935	0.000847	0.428549
2	6	0	-0.898889	2.480558	0.072728
3	1	0	-1.771185	2.968331	-0.365135
4	1	0	-0.966505	2.532393	1.165760
5	1	0	0.009861	2.990509	-0.261565
6	6	0	2.597563	-0.464381	0.072416
7	1	0	3.456863	0.041784	-0.370218
8	1	0	2.680210	-0.428167	1.165043
9	1	0	2.579072	-1.507453	-0.258109
10	6	0	-1.699426	-2.015918	0.071981
11	1	0	-1.690071	-3.013605	-0.369660
12	1	0	-1.710843	-2.104595	1.164675
13	1	0	-2.593183	-1.478860	-0.260302
14	8	0	1.423873	0.230113	-0.388499
15	8	0	-0.909260	1.117393	-0.389745
16	8	0	-0.510662	-1.346991	-0.387960

PCl_3 experimental geometry B3LYP/6-31+G(d,p), chloroform IEFPCM:
Sum of electronic and thermal Free Energies= -1722.026391
(not a minimum, but 0 negative frequencies)

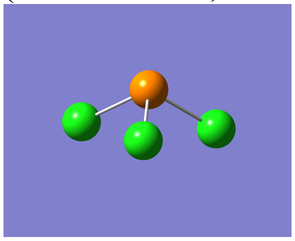

Center Number	Atomic Number	Atomic Type	Coordinates (Angstroms) X	Y	Z
1	15	0	0.000000	0.000000	0.727649
2	17	0	-0.024723	1.802585	-0.233562
3	17	0	-1.562204	-0.926353	-0.207892
4	17	0	1.569915	-0.893466	-0.226780

PCl_3 B3LYP/6-31+G(d,p), chloroform IEFPCM:
Sum of electronic and thermal Free Energies= -1722.031156

Center Number	Atomic Number	Atomic Type	Coordinates (Angstroms) X	Y	Z
1	15	0	0.000125	-0.000073	0.737818
2	17	0	-0.187298	1.856658	-0.216980
3	17	0	1.701751	-0.766151	-0.217049
4	17	0	-1.514564	-1.090443	-0.216988

H_3PO_4 B3LYP/6-31+G(d,p), water IEFPCM:
Sum of electronic and thermal Free Energies= -644.171996

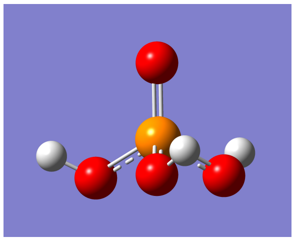

Center Number	Atomic Number	Atomic Type	Coordinates (Angstroms) X	Y	Z
1	15	0	-1.275844	1.094651	-0.164134
2	8	0	-1.127532	1.844381	1.248993
3	1	0	-0.269852	2.282685	1.368719
4	8	0	-0.707671	-0.368517	0.177684
5	1	0	-0.541245	-0.914600	-0.607269
6	8	0	-2.858294	0.825373	-0.232584
7	1	0	-3.361767	1.557485	-0.623378
8	8	0	-0.672161	1.779153	-1.338747

PH_4^+ BF_4^- B3LYP/6-31+G(d,p), chloroform IEFPCM:
Sum of electronic and thermal Free Energies= -768.196320

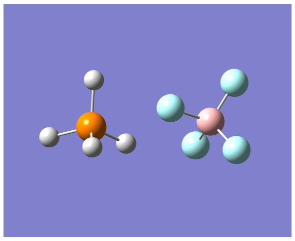

Center Number	Atomic Number	Atomic Type	Coordinates (Angstroms) X	Y	Z
1	15	0	-1.405531	0.991511	0.117559
2	1	0	-0.553249	-0.117894	0.098947
3	1	0	-1.147328	1.794646	1.226651
4	1	0	-1.271106	1.739029	-1.050389
5	1	0	-2.737904	0.572290	0.200673
6	5	0	2.129441	0.721389	-0.120944
7	9	0	1.115159	1.744291	-0.051467
8	9	0	3.020191	0.887782	0.940741
9	9	0	1.460234	-0.531295	-0.007077
10	9	0	2.778375	0.806311	-1.354693

$P(OPh)_4^+$ PF_6^- B3LYP/6-31+G(d,p), chloroform IEFPCM:
Sum of electronic and thermal Free Energies= -2509.259000

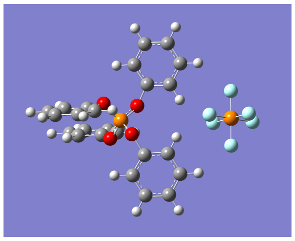

Center Number	Atomic Number	Atomic Type	Coordinates (Angstroms) X	Y	Z
1	15	0	-1.579380	0.008765	0.024305
2	8	0	-2.462947	0.634993	1.159277
3	8	0	-0.726662	1.180069	-0.562478
4	8	0	-0.782384	-1.167394	0.677065
5	8	0	-2.374613	-0.611870	-1.176447
6	6	0	-3.060118	0.086824	-2.218640
7	6	0	-4.448893	0.078720	-2.186324
8	6	0	-2.325942	0.657796	-3.251004

9	6	0	-5.134573	0.693689	-3.238209
10	1	0	-4.975896	-0.405378	-1.371766
11	6	0	-3.030047	1.270243	-4.292151
12	1	0	-1.242663	0.624408	-3.245487
13	6	0	-4.428094	1.291072	-4.286364
14	1	0	-6.219781	0.698818	-3.235957
15	1	0	-2.479109	1.725157	-5.108876
16	1	0	-4.965839	1.766022	-5.100586
17	6	0	-3.258982	-0.058165	2.123411
18	6	0	-4.638806	0.023557	1.984352
19	6	0	-2.638542	-0.695839	3.190932
20	6	0	-5.435642	-0.584525	2.958981
21	1	0	-5.074556	0.558193	1.148014
22	6	0	-3.452786	-1.300208	4.153620
23	1	0	-1.558117	-0.719211	3.271609
24	6	0	-4.845221	-1.247972	4.038722
25	1	0	-6.516140	-0.532597	2.872215
26	1	0	-2.991618	-1.806242	4.995522
27	1	0	-5.468696	-1.717272	4.792852
28	6	0	-0.086275	-2.228563	0.003190
29	6	0	1.295466	-2.143538	-0.082941
30	6	0	-0.818535	-3.319307	-0.449435
31	6	0	1.978379	-3.211023	-0.675730
32	1	0	1.833648	-1.285894	0.303442
33	6	0	-0.117121	-4.375699	-1.037738
34	1	0	-1.897177	-3.344748	-0.346030
35	6	0	1.275816	-4.321223	-1.152649
36	1	0	3.058681	-3.158007	-0.755569
37	1	0	-0.664107	-5.239687	-1.401557
38	1	0	1.813338	-5.145750	-1.610137
39	6	0	-0.050097	2.217896	0.165195
40	6	0	-0.791631	3.294180	0.637054
41	6	0	1.329210	2.127230	0.280291
42	6	0	-0.102772	4.327259	1.279361
43	1	0	-1.867104	3.327042	0.506340
44	6	0	1.999340	3.172181	0.925287
45	1	0	1.874696	1.284772	-0.128763
46	6	0	1.286937	4.265778	1.425612
47	1	0	-0.656955	5.179614	1.659382
48	1	0	3.078118	3.117150	1.023152
49	1	0	1.814436	5.073083	1.923695
50	15	0	5.003318	-0.015120	-0.041272
51	9	0	4.977443	1.599926	0.275930
52	9	0	6.224776	-0.212893	1.035704
53	9	0	3.897245	-0.263081	1.160669
54	9	0	5.012067	-1.630631	-0.357307

55	9	0	6.088426	0.232218	-1.246834
56	9	0	3.762690	0.183057	-1.114857

O=P(OCH2)3P=O B3LYP/6-31+G(d,p), chloroform IEFPCM:
Sum of electronic and thermal Free Energies= -1176.781610

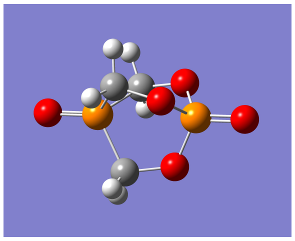

Center Number	Atomic Number	Atomic Type	Coordinates (Angstroms) X	Y	Z
1	6	0	-1.033549	0.473950	-1.358845
2	1	0	-1.417424	1.059965	-2.196308
3	6	0	-1.330108	-1.646848	0.508483
4	1	0	-0.601422	-2.347210	0.095509
5	6	0	-3.428087	0.229860	0.146366
6	1	0	-4.261860	-0.372611	0.511768
7	15	0	-1.111462	0.887948	1.259703
8	8	0	-0.534340	1.735609	2.314933
9	8	0	-0.584639	-0.644379	1.260230
10	8	0	-2.724235	0.742916	1.315782
11	8	0	-0.819758	1.397435	-0.250693
12	1	0	-3.813430	1.066854	-0.442634
13	1	0	-0.080841	0.015962	-1.639325
14	1	0	-2.007458	-2.179060	1.182468
15	15	0	-2.271451	-0.817749	-0.861237
16	8	0	-2.859262	-1.682197	-1.938207

$P(OMe)_4^+$ BF_4^- isomer A B3LYP/6-31+G(d,p), chloroform IEFPCM:
Sum of electronic and thermal Free Energies= -1226.318728

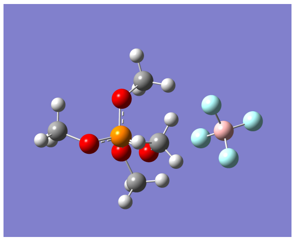

Center Number	Atomic Number	Atomic Type	Coordinates (Angstroms) X	Y	Z
1	15	0	-0.238893	0.625578	-0.451036
2	8	0	0.349916	1.703766	0.503145
3	8	0	0.074692	-0.765260	0.212293
4	8	0	-1.788933	0.638114	-0.661875
5	8	0	0.437494	0.813625	-1.846633
6	6	0	-0.100575	1.975452	1.873917
7	1	0	-0.126868	1.044237	2.440449
8	1	0	0.642476	2.658758	2.276826
9	1	0	-1.073616	2.461139	1.826166
10	6	0	1.710983	1.507693	-2.097190
11	1	0	2.511214	1.005676	-1.553343
12	1	0	1.861841	1.421682	-3.170908
13	1	0	1.598836	2.547800	-1.796794
14	6	0	-0.403968	-2.044405	-0.313049
15	1	0	-0.033193	-2.792827	0.383877
16	1	0	-1.493811	-2.043602	-0.336099
17	1	0	0.011961	-2.208978	-1.308004
18	6	0	-2.516187	1.616474	-1.493534
19	1	0	-2.148042	1.548212	-2.516697
20	1	0	-3.556104	1.303819	-1.431202
21	1	0	-2.370788	2.613686	-1.081786
22	9	0	0.526088	5.061237	0.383706
23	9	0	-1.647814	4.301799	0.190378
24	9	0	-0.193995	4.094140	-1.588405
25	9	0	-0.970773	6.180599	-0.972490
26	5	0	-0.566034	4.921149	-0.495797

$P(OMe)_4^+ BF_4^-$ isomer B B3LYP/6-31+G(d,p), chloroform IEFPCM:
Sum of electronic and thermal Free Energies= -1226.315558

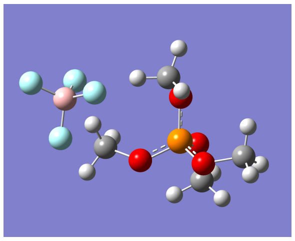

Center Number	Atomic Number	Atomic Type	Coordinates (Angstroms) X	Y	Z
1	15	0	-0.591752	0.335048	-0.086885
2	8	0	-0.068192	0.925221	1.260944

3	8	0	0.052583	-1.099609	-0.050089
4	8	0	-2.158586	0.296693	-0.188432
5	8	0	-0.224148	1.139282	-1.371423
6	6	0	-0.412707	2.267600	1.777112
7	1	0	-1.491431	2.414947	1.726388
8	1	0	-0.073917	2.251958	2.810314
9	1	0	0.117705	3.021459	1.198130
10	6	0	1.133910	1.615283	-1.706449
11	1	0	1.872812	0.860869	-1.433422
12	1	0	1.112542	1.761997	-2.783574
13	1	0	1.300785	2.558513	-1.189177
14	6	0	-0.103354	-2.068992	-1.138932
15	1	0	0.503227	-2.921365	-0.842098
16	1	0	-1.151488	-2.356282	-1.228832
17	1	0	0.264179	-1.643023	-2.072874
18	6	0	-3.009863	-0.384717	0.790917
19	1	0	-2.813587	0.002974	1.791010
20	1	0	-4.027013	-0.152140	0.484621
21	1	0	-2.832001	-1.459647	0.748196
22	9	0	0.873707	6.621936	-0.682369
23	9	0	-0.814728	5.352158	0.255941
24	9	0	1.283132	4.439647	-0.036230
25	9	0	-0.050636	4.867376	-1.869110
26	5	0	0.317079	5.335819	-0.589371

(*i*-PrO)$_2$P(O)H isomer A B3LYP/6-31+G(d,p), chloroform IEFPCM:
Sum of electronic and thermal Free Energies= -804.640120

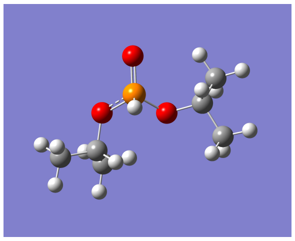

Center Number	Atomic Number	Atomic Type	Coordinates (Angstroms) X	Y	Z
1	15	0	-0.157156	0.251634	-1.038398
2	1	0	0.072968	0.177068	-2.424323
3	8	0	0.673282	1.284025	-0.357426
4	8	0	0.094263	-1.228047	-0.483981
5	8	0	-1.758610	0.407748	-0.921245
6	6	0	-2.425492	1.662091	-1.309632
7	1	0	-1.748548	2.221428	-1.967543
8	6	0	-0.556366	-2.420613	-1.053479

9	1	0	-1.069045	-2.123690	-1.976328
10	6	0	0.547001	-3.418127	-1.375858
11	1	0	1.092248	-3.692168	-0.466970
12	1	0	0.113286	-4.326359	-1.806870
13	1	0	1.256779	-2.999252	-2.094885
14	6	0	-1.572806	-2.934297	-0.043627
15	1	0	-1.075441	-3.204412	0.893531
16	1	0	-2.330183	-2.174991	0.166003
17	1	0	-2.071894	-3.824786	-0.440080
18	6	0	-3.678434	1.272682	-2.080149
19	1	0	-4.211866	2.172711	-2.402492
20	1	0	-3.426389	0.684602	-2.967413
21	1	0	-4.348995	0.682024	-1.447336
22	6	0	-2.705006	2.474254	-0.052562
23	1	0	-3.359721	1.915234	0.624033
24	1	0	-1.775357	2.710125	0.471316
25	1	0	-3.202255	3.412781	-0.319666

(i-PrO)$_2$P(O)H isomer B B3LYP/6-31+G(d,p), chloroform IEFPCM:
Sum of electronic and thermal Free Energies= -804.640982

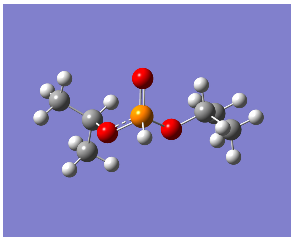

Center Number	Atomic Number	Atomic Type	Coordinates (Angstroms) X	Y	Z
1	15	0	-0.658131	0.061374	-2.534242
2	1	0	-1.483338	-0.631526	-3.427203
3	8	0	-0.088605	1.334067	-3.071514
4	8	0	0.352232	-1.126326	-2.164351
5	8	0	-1.515948	0.237684	-1.177289
6	6	0	-2.239326	1.477100	-0.853536
7	1	0	-1.733580	2.295433	-1.374782
8	6	0	1.456804	-0.951103	-1.204186
9	1	0	1.327464	0.016580	-0.707756
10	6	0	1.341199	-2.073198	-0.183047
11	1	0	1.426472	-3.047936	-0.674646
12	1	0	2.144653	-1.987575	0.555878
13	1	0	0.382192	-2.026929	0.339917
14	6	0	2.763885	-0.962712	-1.984211
15	1	0	2.884819	-1.913840	-2.513061

16	1	0	2.790365	-0.149026	-2.713747
17	1	0	3.607646	-0.839456	-1.297074
18	6	0	-3.678601	1.352623	-1.339232
19	1	0	-4.234788	2.262476	-1.090428
20	1	0	-3.722928	1.218020	-2.424503
21	1	0	-4.174520	0.501746	-0.860605
22	6	0	-2.122377	1.667710	0.651324
23	1	0	-2.584882	0.830510	1.184743
24	1	0	-1.073860	1.740057	0.953953
25	1	0	-2.631591	2.590233	0.948418

(*i*-PrO)$_2$P(O)H isomer C B3LYP/6-31+G(d,p), chloroform IEFPCM:
Sum of electronic and thermal Free Energies= -804.642354

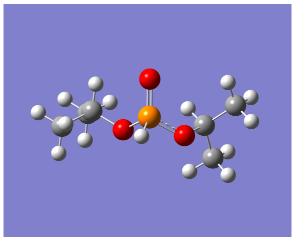

Center Number	Atomic Number	Atomic Type	Coordinates (Angstroms) X	Y	Z
1	15	0	-0.862163	-0.915970	-1.484482
2	1	0	-1.257065	-2.225678	-1.188966
3	8	0	-0.494146	-0.682463	-2.913699
4	8	0	0.304556	-0.553459	-0.428557
5	8	0	-2.132417	-0.139042	-0.891538
6	6	0	-2.241967	1.330456	-0.927398
7	1	0	-1.256190	1.739550	-1.173898
8	6	0	1.733834	-0.624157	-0.769504
9	1	0	1.816374	-0.514252	-1.854937
10	6	0	2.405468	0.551828	-0.076001
11	1	0	2.289444	0.478112	1.010458
12	1	0	3.475037	0.558387	-0.309626
13	1	0	1.973945	1.499206	-0.411348
14	6	0	2.279304	-1.978610	-0.332908
15	1	0	2.153507	-2.114891	0.746266
16	1	0	1.770940	-2.798294	-0.850163
17	1	0	3.346892	-2.042636	-0.567970
18	6	0	-3.245181	1.705706	-2.009276
19	1	0	-3.349560	2.794806	-2.057201
20	1	0	-2.916273	1.346346	-2.987891
21	1	0	-4.226554	1.273799	-1.787106
22	6	0	-2.649003	1.776803	0.469014

23	1	0	-3.614132	1.338906	0.744391
24	1	0	-1.902739	1.473417	1.208310
25	1	0	-2.743627	2.867238	0.497970

(i-PrO)$_2$P(O)H isomer D B3LYP/6-31+G(d,p), chloroform IEFPCM:
Sum of electronic and thermal Free Energies= -804.639339

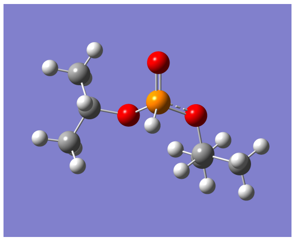

Center Number	Atomic Number	Atomic Type	Coordinates (Angstroms) X	Y	Z
1	15	0	-0.255597	-0.432840	-1.776216
2	1	0	-0.050753	-1.550673	-2.605630
3	8	0	0.042779	0.863630	-2.446562
4	8	0	0.589629	-0.780230	-0.446521
5	8	0	-1.769755	-0.550324	-1.271833
6	6	0	-2.318565	-1.776809	-0.668617
7	1	0	-1.597358	-2.588087	-0.826354
8	6	0	2.061951	-0.774698	-0.463372
9	1	0	2.393550	-0.895951	-1.502396
10	6	0	2.552382	0.560906	0.078881
11	1	0	2.206374	0.704358	1.107800
12	1	0	3.647237	0.584440	0.074556
13	1	0	2.184167	1.386618	-0.534900
14	6	0	2.512550	-1.973585	0.358053
15	1	0	2.155607	-1.886345	1.389500
16	1	0	2.130392	-2.906253	-0.066952
17	1	0	3.605998	-2.023819	0.374268
18	6	0	-2.502655	-1.534670	0.822910
19	1	0	-2.908034	-2.434478	1.297607
20	1	0	-1.548825	-1.290372	1.296738
21	1	0	-3.201956	-0.709249	0.991211
22	6	0	-3.613105	-2.091042	-1.404195
23	1	0	-4.326116	-1.268106	-1.291177
24	1	0	-3.429654	-2.249408	-2.470754
25	1	0	-4.064118	-2.999357	-0.991589

(MeO)$_2$P(O)H isomer A B3LYP/6-31+G(d,p), chloroform IEFPCM:
Sum of electronic and thermal Free Energies= -647.455600

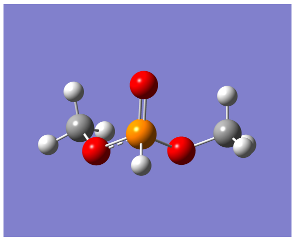

Center Number	Atomic Number	Atomic Type	Coordinates (Angstroms) X	Y	Z
1	15	0	0.008533	0.505973	0.389564
2	8	0	-1.435104	-0.168377	0.579883
3	8	0	0.965543	-0.787165	0.224406
4	8	0	0.166178	1.538332	-0.676092
5	6	0	-2.134189	-0.773518	-0.537223
6	1	0	-3.102763	-1.085302	-0.148205
7	1	0	-2.270735	-0.044721	-1.339254
8	1	0	-1.581122	-1.642531	-0.901348
9	6	0	2.318249	-0.647840	-0.273580
10	1	0	2.677797	-1.660632	-0.454293
11	1	0	2.326586	-0.073243	-1.201708
12	1	0	2.949348	-0.161421	0.476120
13	1	0	0.212813	0.966046	1.695502

(MeO)$_2$P(O)H isomer B B3LYP/6-31+G(d,p), chloroform IEFPCM:
Sum of electronic and thermal Free Energies= -647.454329

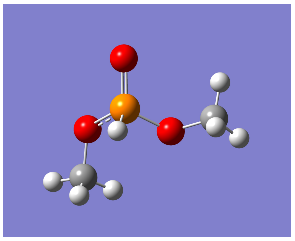

Center Number	Atomic Number	Atomic Type	Coordinates (Angstroms) X	Y	Z
1	15	0	-0.303964	-0.016031	0.592833
2	8	0	-1.607796	0.468114	-0.204322
3	8	0	-0.030706	-1.465267	-0.064950
4	8	0	0.815649	0.962275	0.547274
5	6	0	-2.802507	-0.347593	-0.280139
6	1	0	-3.557222	0.271764	-0.763428
7	1	0	-2.608393	-1.241590	-0.875935

8	1	0	-3.143940	-0.627301	0.721299
9	6	0	1.149519	-2.223273	0.294861
10	1	0	1.102244	-3.145896	-0.282777
11	1	0	2.051514	-1.664541	0.036709
12	1	0	1.144432	-2.457921	1.363666
13	1	0	-0.753672	-0.309928	1.893049

$(MeO)_2P(O)H$ isomer C B3LYP/6-31+G(d,p), chloroform IEFPCM:
Sum of electronic and thermal Free Energies= -647.454267

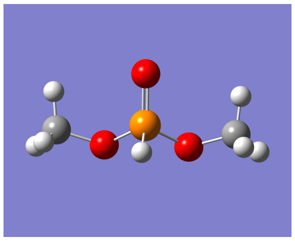

Center Number	Atomic Number	Atomic Type	Coordinates (Angstroms) X	Y	Z
1	15	0	0.083383	0.479543	0.631586
2	8	0	-1.292061	-0.282211	0.968591
3	8	0	0.943633	-0.810707	0.206400
4	8	0	0.019989	1.610416	-0.338563
5	6	0	-2.483923	0.485670	1.265586
6	1	0	-3.280382	-0.244403	1.405043
7	1	0	-2.349609	1.063258	2.185203
8	1	0	-2.725939	1.150897	0.434145
9	6	0	2.267590	-0.637795	-0.355411
10	1	0	2.616595	-1.638762	-0.606533
11	1	0	2.222548	-0.019493	-1.254333
12	1	0	2.941155	-0.187385	0.379964
13	1	0	0.600949	0.869865	1.879241

$Ph_4P^+ Cl^-$ B3LYP/6-31+G(d,p), chloroform IEFPCM:
Sum of electronic and thermal Free Energies= -1727.922386

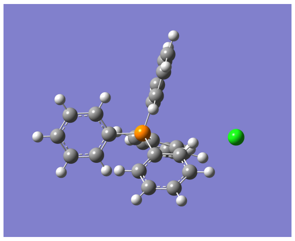

Center Number	Atomic Number	Atomic Type	Coordinates (Angstroms) X	Y	Z
1	15	0	0.744547	-0.000108	-0.010932
2	6	0	0.380974	-1.474010	-1.019253
3	6	0	-0.807372	-1.544609	-1.767282
4	6	0	1.260173	-2.571938	-0.994925
5	6	0	-1.093982	-2.698861	-2.498100
6	1	0	-1.534910	-0.738551	-1.775108
7	6	0	0.961256	-3.720130	-1.730247
8	1	0	2.172036	-2.540524	-0.409621
9	6	0	-0.212105	-3.782724	-2.486657
10	1	0	-2.016195	-2.744869	-3.067993
11	1	0	1.646392	-4.561871	-1.708758
12	1	0	-0.441786	-4.676081	-3.059517
13	6	0	0.333484	1.551674	-0.870872
14	6	0	-1.013791	1.862264	-1.129914
15	6	0	1.346499	2.446074	-1.258957
16	6	0	-1.332160	3.049991	-1.789949
17	1	0	-1.831478	1.204696	-0.842275
18	6	0	1.012970	3.634017	-1.911777
19	1	0	2.387857	2.232385	-1.050072
20	6	0	-0.324644	3.935802	-2.181092
21	1	0	-2.374720	3.270640	-1.994266
22	1	0	1.800393	4.321158	-2.205402
23	1	0	-0.580744	4.859625	-2.691069
24	6	0	2.522583	-0.006387	0.385097
25	6	0	2.956436	0.126596	1.712873
26	6	0	3.469154	-0.123677	-0.650048
27	6	0	4.323667	0.145226	2.001022
28	1	0	2.238214	0.211393	2.520818
29	6	0	4.831648	-0.103337	-0.353572
30	1	0	3.150231	-0.236821	-1.681528
31	6	0	5.260188	0.031856	0.971391
32	1	0	4.651992	0.245708	3.030587
33	1	0	5.555962	-0.194879	-1.156669
34	1	0	6.321651	0.045604	1.198721
35	6	0	-0.214764	-0.076195	1.529906
36	6	0	-0.504821	1.105158	2.233331
37	6	0	-0.631567	-1.317348	2.036790
38	6	0	-1.203435	1.038879	3.439161
39	1	0	-0.199552	2.069893	1.841441
40	6	0	-1.329730	-1.373118	3.243969
41	1	0	-0.428160	-2.232557	1.491119
42	6	0	-1.615038	-0.198013	3.944524

43	1	0	-1.433897	1.953450	3.976070
44	1	0	-1.660324	-2.332831	3.627907
45	1	0	-2.167050	-0.244636	4.878183
46	17	0	-4.139037	0.143727	-1.434061

Ph_4P^+ Br^- B3LYP/6-31+G(d,p), chloroform IEFPCM:
Sum of electronic and thermal Free Energies= -3839.455631

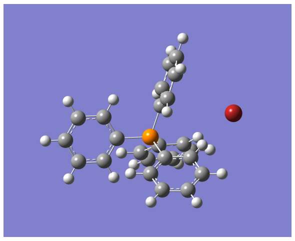

Center Number	Atomic Number	Atomic Type	Coordinates (Angstroms) X	Y	Z
1	15	0	0.773909	0.000473	-0.012692
2	6	0	0.388374	-1.474445	-1.017002
3	6	0	-0.944530	-1.690415	-1.411492
4	6	0	1.385870	-2.391152	-1.389531
5	6	0	-1.260044	-2.798837	-2.197672
6	1	0	-1.752416	-1.037514	-1.085240
7	6	0	1.056248	-3.496475	-2.177310
8	1	0	2.411753	-2.261881	-1.067119
9	6	0	-0.263293	-3.696903	-2.589806
10	1	0	-2.292457	-2.958737	-2.492172
11	1	0	1.832690	-4.200000	-2.461324
12	1	0	-0.515815	-4.556527	-3.203393
13	6	0	0.396813	1.537959	-0.921863
14	6	0	-0.942372	1.818782	-1.248188
15	6	0	1.411434	2.435297	-1.295372
16	6	0	-1.248870	2.974446	-1.967254
17	1	0	-1.759286	1.175962	-0.923925
18	6	0	1.090549	3.588599	-2.014864
19	1	0	2.444585	2.253722	-1.025684
20	6	0	-0.236970	3.855827	-2.358325
21	1	0	-2.285830	3.183597	-2.210479
22	1	0	1.880214	4.276996	-2.299503
23	1	0	-0.482823	4.752872	-2.918763
24	6	0	2.541079	-0.018139	0.430732
25	6	0	2.919889	-0.031413	1.782391
26	6	0	3.533589	-0.014902	-0.568429
27	6	0	4.273149	-0.041490	2.129503
28	1	0	2.167781	-0.034364	2.563283

29	6	0	4.882198	-0.025285	-0.212936
30	1	0	3.264207	-0.005681	-1.620015
31	6	0	5.253727	-0.038629	1.135552
32	1	0	4.555798	-0.052045	3.177326
33	1	0	5.640395	-0.023370	-0.989563
34	1	0	6.304455	-0.047020	1.408366
35	6	0	-0.229726	-0.044749	1.496619
36	6	0	-0.618945	1.152190	2.118086
37	6	0	-0.599650	-1.279556	2.052946
38	6	0	-1.373202	1.109666	3.290452
39	1	0	-0.354378	2.111183	1.685609
40	6	0	-1.352751	-1.312196	3.226265
41	1	0	-0.321034	-2.209527	1.568530
42	6	0	-1.741883	-0.119837	3.842824
43	1	0	-1.691821	2.036723	3.756085
44	1	0	-1.655899	-2.267789	3.641808
45	1	0	-2.345736	-0.148758	4.744577
46	35	0	-3.901928	0.005081	-0.038047

Me_4P^+ Cl^- B3LYP/6-31+G(d,p), chloroform IEFPCM:
Sum of electronic and thermal Free Energies= -961.164000

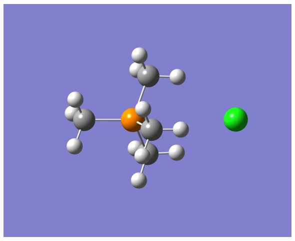

Center Number	Atomic Number	Atomic Type	Coordinates (Angstroms) X	Y	Z
1	15	0	1.502396	-0.001718	0.001334
2	6	0	3.321933	0.002786	0.002647
3	1	0	3.696764	-0.977872	-0.301493
4	1	0	3.688231	0.758950	-0.696294
5	1	0	3.691092	0.232913	1.005462
6	6	0	0.897275	1.623281	0.526554
7	1	0	1.260443	2.391883	-0.160384
8	1	0	-0.197199	1.586943	0.507052
9	1	0	1.249124	1.842098	1.537986
10	6	0	0.899659	-1.272793	1.143311
11	1	0	-0.194383	-1.227590	1.136719
12	1	0	1.235537	-2.258488	0.810817
13	1	0	1.279306	-1.077106	2.149210
14	6	0	0.900041	-0.356408	-1.670444

15	1	0	1.261360	-1.335904	-1.994471
16	1	0	-0.194627	-0.352050	-1.631241
17	1	0	1.254742	0.412095	-2.362458
18	17	0	-2.336350	0.008768	-0.004585

Me_4P^+ Br^- B3LYP/6-31+G(d,p), chloroform IEFPCM:
Sum of electronic and thermal Free Energies= -3072.691807

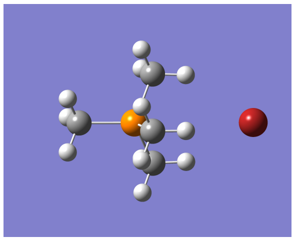

Center Number	Atomic Number	Atomic Type	Coordinates (Angstroms) X	Y	Z
1	15	0	1.506883	-0.001186	0.000760
2	6	0	3.326285	0.002279	0.003647
3	1	0	3.701052	-0.977946	-0.301989
4	1	0	3.693521	0.759734	-0.693454
5	1	0	3.694503	0.230316	1.007324
6	6	0	0.903935	1.625509	0.526006
7	1	0	1.261993	2.392325	-0.165649
8	1	0	-0.190657	1.587531	0.513721
9	1	0	1.262172	1.846846	1.534664
10	6	0	0.900156	-1.272969	1.140668
11	1	0	-0.194428	-1.232620	1.119165
12	1	0	1.246016	-2.257470	0.814797
13	1	0	1.266238	-1.071333	2.150437
14	6	0	0.905424	-0.355535	-1.672290
15	1	0	1.264463	-1.336507	-1.994571
16	1	0	-0.189366	-0.348856	-1.632565
17	1	0	1.262272	0.411606	-2.364718
18	35	0	-2.405115	-0.001938	0.013768

(i-PrO)$_2$P(O)Me isomer A B3LYP/6-31+G(d,p), chloroform IEFPCM:
Sum of electronic and thermal Free Energies= -843.946567

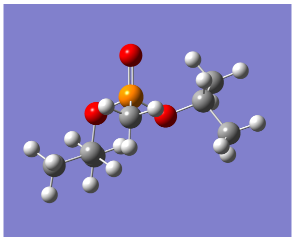

Center Number	Atomic Number	Atomic Type	Coordinates (Angstroms) X	Y	Z
1	15	0	-0.122454	0.263718	-1.278324
2	8	0	0.649399	1.332410	-0.575884
3	8	0	0.135907	-1.186395	-0.630262
4	8	0	-1.736323	0.393202	-1.189875
5	6	0	-2.418628	1.690935	-1.275776
6	1	0	-1.694688	2.437642	-1.620700
7	6	0	-0.628411	-2.398112	-0.953755
8	1	0	-1.308305	-2.168444	-1.781443
9	6	0	0.370130	-3.465357	-1.381644
10	1	0	1.075488	-3.675048	-0.571012
11	1	0	-0.157428	-4.392238	-1.629814
12	1	0	0.936684	-3.145218	-2.260907
13	6	0	-1.439731	-2.784180	0.275924
14	1	0	-0.775764	-2.982867	1.123534
15	1	0	-2.130457	-1.983188	0.550920
16	1	0	-2.020504	-3.689831	0.070763
17	6	0	-3.540169	1.545400	-2.295365
18	1	0	-4.077453	2.493974	-2.397202
19	1	0	-3.145937	1.266561	-3.277001
20	1	0	-4.253014	0.778317	-1.974811
21	6	0	-2.906997	2.066989	0.116908
22	1	0	-3.607968	1.314065	0.492686
23	1	0	-2.067423	2.144598	0.812435
24	1	0	-3.421375	3.033433	0.084619
25	6	0	0.267340	0.132541	-3.045923
26	1	0	-0.001326	1.067701	-3.544828
27	1	0	1.341431	-0.032621	-3.161142
28	1	0	-0.281539	-0.689415	-3.512416

(i-PrO)$_2$P(O)Me isomer B B3LYP/6-31+G(d,p), chloroform IEFPCM:
Sum of electronic and thermal Free Energies= -843.951165

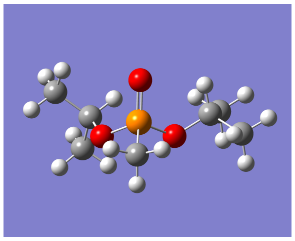

Center Number	Atomic Number	Atomic Type	Coordinates (Angstroms) X	Y	Z
1	15	0	0.143794	0.600696	-2.552158
2	8	0	0.763920	1.818752	-1.937962
3	8	0	0.898542	-0.800029	-2.286206
4	8	0	-1.358673	0.306217	-2.011626
5	6	0	-2.179854	1.318934	-1.338361
6	1	0	-1.519833	2.147123	-1.064011
7	6	0	1.264803	-1.228171	-0.929938
8	1	0	0.709617	-0.611410	-0.214450
9	6	0	0.837896	-2.682494	-0.791295
10	1	0	1.354115	-3.304386	-1.530305
11	1	0	1.091005	-3.052085	0.207901
12	1	0	-0.240647	-2.787598	-0.937781
13	6	0	2.761041	-1.010379	-0.744474
14	1	0	3.326771	-1.604463	-1.470116
15	1	0	3.018180	0.043764	-0.876482
16	1	0	3.062148	-1.317316	0.262889
17	6	0	-3.253387	1.799717	-2.307702
18	1	0	-3.891747	2.542776	-1.818160
19	1	0	-2.809593	2.266308	-3.192318
20	1	0	-3.882424	0.963778	-2.631864
21	6	0	-2.750093	0.672417	-0.083152
22	1	0	-3.373983	-0.190129	-0.340749
23	1	0	-1.948281	0.338098	0.581525
24	1	0	-3.367745	1.395010	0.460374
25	6	0	0.048751	0.597295	-4.356767
26	1	0	1.058415	0.650253	-4.771062
27	1	0	-0.446007	-0.309864	-4.710418
28	1	0	-0.517660	1.473076	-4.682807

(i-PrO)$_2$P(O)Me isomer C B3LYP/6-31+G(d,p), chloroform IEFPCM:
Sum of electronic and thermal Free Energies= -843.946808

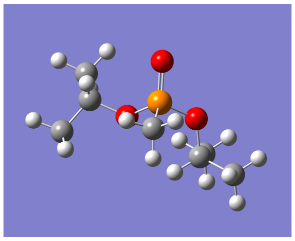

Center Number	Atomic Number	Atomic Type	Coordinates (Angstroms) X	Y	Z
1	15	0	-0.204032	-0.593156	-1.903175
2	8	0	0.139833	0.732076	-2.500431
3	8	0	0.641523	-1.008149	-0.583190
4	8	0	-1.717057	-0.628435	-1.357271
5	6	0	-2.287216	-1.699531	-0.530042
6	1	0	-1.537897	-2.491510	-0.424194
7	6	0	2.083437	-0.750871	-0.475359
8	1	0	2.468053	-0.520441	-1.475484
9	6	0	2.290441	0.452043	0.435420
10	1	0	1.885196	0.250291	1.432648
11	1	0	3.359851	0.667528	0.533176
12	1	0	1.794653	1.335515	0.025468
13	6	0	2.729251	-2.027518	0.045656
14	1	0	2.327113	-2.285794	1.030967
15	1	0	2.549740	-2.865026	-0.634927
16	1	0	3.810824	-1.886596	0.141088
17	6	0	-2.601111	-1.113102	0.839783
18	1	0	-3.032501	-1.884404	1.486685
19	1	0	-1.693746	-0.729588	1.313205
20	1	0	-3.322474	-0.294602	0.747370
21	6	0	-3.513447	-2.235424	-1.256990
22	1	0	-4.252266	-1.439836	-1.397620
23	1	0	-3.245670	-2.637343	-2.238505
24	1	0	-3.974845	-3.036895	-0.670677
25	6	0	-0.021207	-1.978091	-3.062189
26	1	0	-0.625593	-1.779262	-3.950666
27	1	0	1.027302	-2.063049	-3.360680
28	1	0	-0.337930	-2.920118	-2.607356

Ph$_3$PO B3LYP/6-31+G(d,p), chloroform IEFPCM:
Sum of electronic and thermal Free Energies= -1111.383399

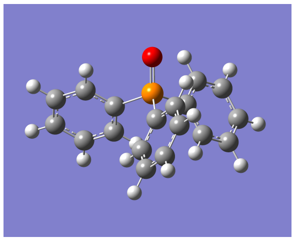

Center Number	Atomic Number	Atomic Type	Coordinates (Angstroms) X	Y	Z
1	15	0	-1.200961	1.372497	0.051918
2	8	0	-2.697737	1.429316	-0.172113
3	6	0	-0.261947	2.094450	-1.345403
4	6	0	-0.797881	1.943953	-2.635219
5	6	0	0.947427	2.786233	-1.178680
6	6	0	-0.127350	2.469230	-3.741606
7	1	0	-1.743305	1.426789	-2.767597
8	6	0	1.616498	3.311252	-2.288339
9	1	0	1.365951	2.926938	-0.186913
10	6	0	1.081350	3.152083	-3.569625
11	1	0	-0.550380	2.350690	-4.734781
12	1	0	2.549816	3.848946	-2.149532
13	1	0	1.600503	3.563654	-4.430277
14	6	0	-0.689724	2.291316	1.552018
15	6	0	-1.430646	3.434147	1.894966
16	6	0	0.390984	1.903189	2.358212
17	6	0	-1.087258	4.183340	3.022131
18	1	0	-2.279787	3.726047	1.284707
19	6	0	0.732746	2.655255	3.486100
20	1	0	0.962311	1.012130	2.116792
21	6	0	-0.003945	3.795844	3.817810
22	1	0	-1.667262	5.064085	3.281554
23	1	0	1.568578	2.345638	4.106572
24	1	0	0.260691	4.377035	4.696353
25	6	0	-0.595129	-0.343464	0.259412
26	6	0	-1.454730	-1.264652	0.880417
27	6	0	0.671684	-0.765859	-0.171533
28	6	0	-1.046784	-2.585435	1.077592
29	1	0	-2.443735	-0.947791	1.197020
30	6	0	1.077808	-2.088630	0.028146
31	1	0	1.340696	-0.071675	-0.670752
32	6	0	0.220905	-2.998267	0.654101
33	1	0	-1.718926	-3.291625	1.556035
34	1	0	2.058983	-2.407521	-0.311079

35	1	0	0.536618	-4.026386	0.805526

(MeO)$_2$P(O)Me isomer A B3LYP/6-31+G(d,p), chloroform IEFPCM:
Sum of electronic and thermal Free Energies= -686.765788

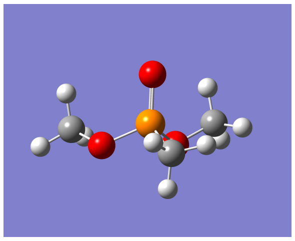

Center Number	Atomic Number	Atomic Type	Coordinates (Angstroms) X	Y	Z
1	15	0	0.005138	0.358880	0.166662
2	8	0	-0.115892	0.201960	1.649760
3	6	0	-0.396983	1.993023	-0.487149
4	1	0	-0.258940	2.013630	-1.570201
5	1	0	-1.438234	2.222733	-0.246460
6	1	0	0.249070	2.738791	-0.018037
7	8	0	1.470799	0.113707	-0.463092
8	8	0	-0.887484	-0.712436	-0.671678
9	6	0	2.218049	-1.078392	-0.124680
10	1	0	1.710755	-1.965461	-0.512905
11	1	0	3.191274	-0.969504	-0.602833
12	1	0	2.340744	-1.156591	0.958142
13	6	0	-2.144256	-1.196204	-0.147365
14	1	0	-2.440602	-2.026122	-0.789391
15	1	0	-2.023889	-1.540412	0.881789
16	1	0	-2.904841	-0.410475	-0.190656

(MeO)$_2$P(O)Me isomer B B3LYP/6-31+G(d,p), chloroform IEFPCM:
Sum of electronic and thermal Free Energies= -686.762492

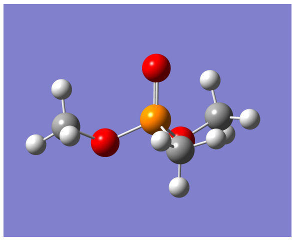

Center Number	Atomic Number	Atomic Type	Coordinates (Angstroms) X	Y	Z
1	15	0	-0.003370	0.151714	0.143822

2	8	0	-0.058052	-0.110033	1.615375
3	6	0	0.125451	1.906343	-0.291594
4	1	0	0.144460	2.026118	-1.377170
5	1	0	-0.732549	2.443641	0.121115
6	1	0	1.038015	2.326175	0.139942
7	8	0	1.186079	-0.598656	-0.659301
8	8	0	-1.241505	-0.428560	-0.719249
9	6	0	2.521346	-0.630316	-0.107492
10	1	0	2.509574	-1.049852	0.901019
11	1	0	3.104889	-1.269377	-0.770070
12	1	0	2.959017	0.372800	-0.090706
13	6	0	-2.549141	-0.577657	-0.120608
14	1	0	-3.137703	-1.160072	-0.829439
15	1	0	-2.478125	-1.106200	0.832017
16	1	0	-3.016835	0.400121	0.028400

$(MeO)_2P(O)Me$ isomer C B3LYP/6-31+G(d,p), chloroform IEFPCM:
Sum of electronic and thermal Free Energies= -686.761925

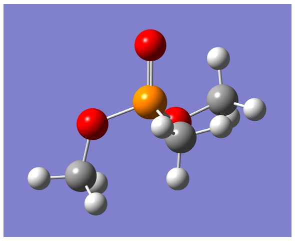

Center Number	Atomic Number	Atomic Type	Coordinates (Angstroms) X	Y	Z
1	15	0	0.036921	0.428951	-0.189735
2	8	0	0.676305	1.247755	-1.260158
3	6	0	-0.124292	1.283521	1.400752
4	1	0	-0.637273	0.660377	2.137295
5	1	0	0.871151	1.532766	1.777771
6	1	0	-0.684583	2.209411	1.249135
7	8	0	-1.437302	-0.066128	-0.615399
8	8	0	0.766341	-0.985959	0.133166
9	6	0	-2.189286	-1.060386	0.114818
10	1	0	-2.413753	-0.711399	1.127155
11	1	0	-3.119944	-1.196815	-0.435798
12	1	0	-1.636287	-2.001113	0.154921
13	6	0	2.207427	-1.082002	0.180499
14	1	0	2.436930	-2.143124	0.278911
15	1	0	2.646321	-0.687703	-0.738536
16	1	0	2.601976	-0.540967	1.046148

Me$_3$PO B3LYP/6-31+G(d,p), chloroform IEFPCM:
Sum of electronic and thermal Free Energies= -536.301776

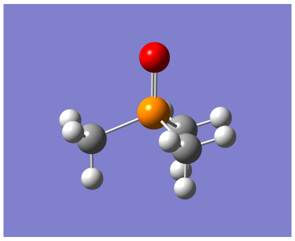

Center Number	Atomic Number	Atomic Type	Coordinates (Angstroms) X	Y	Z
1	15	0	-1.225885	1.330634	0.081116
2	6	0	-0.507434	2.157456	1.545095
3	1	0	-0.724193	3.228277	1.496013
4	1	0	-0.967630	1.748730	2.449074
5	1	0	0.575601	2.010723	1.593865
6	6	0	-0.324811	2.012364	-1.356561
7	1	0	-0.668187	1.511435	-2.266102
8	1	0	-0.541282	3.080877	-1.442693
9	1	0	0.755098	1.870349	-1.252716
10	6	0	-0.701252	-0.416826	0.201086
11	1	0	-1.150495	-0.866943	1.090847
12	1	0	-1.053925	-0.958743	-0.680971
13	1	0	0.387697	-0.501408	0.264850
14	8	0	-2.731202	1.498090	-0.021044

EtOP(O)Me$_2$ isomer A B3LYP/6-31+G(d,p), chloroform IEFPCM:
Sum of electronic and thermal Free Energies= -650.830403

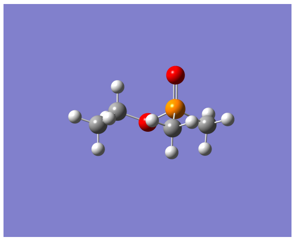

Center Number	Atomic Number	Atomic Type	Coordinates (Angstroms) X	Y	Z
1	15	0	-0.836556	0.003209	0.153891
2	8	0	-1.017240	0.288870	1.621591
3	8	0	0.692824	-0.375513	-0.284752
4	6	0	1.815175	0.405722	0.205184
5	1	0	1.738687	0.491171	1.293283

6	1	0	1.766373	1.411761	-0.228799
7	6	0	3.095402	-0.297842	-0.204556
8	1	0	3.163151	-0.386041	-1.293249
9	1	0	3.958482	0.275506	0.149882
10	1	0	3.143819	-1.300175	0.231491
11	6	0	-1.715302	-1.463160	-0.453397
12	1	0	-1.400519	-2.330079	0.133070
13	1	0	-2.790251	-1.314007	-0.320903
14	1	0	-1.498168	-1.640393	-1.509747
15	6	0	-1.312030	1.410291	-0.902724
16	1	0	-1.140317	1.182067	-1.957994
17	1	0	-2.370540	1.635367	-0.745606
18	1	0	-0.726519	2.289769	-0.621538

EtOP(O)Me$_2$ isomer B B3LYP/6-31+G(d,p), chloroform IEFPCM:
Sum of electronic and thermal Free Energies= -650.826215

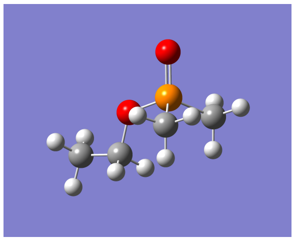

Center Number	Atomic Number	Atomic Type	Coordinates (Angstroms) X	Y	Z
1	15	0	-0.831344	0.005559	0.091534
2	8	0	-0.885394	0.221401	1.576837
3	8	0	0.715282	-0.249748	-0.369525
4	6	0	1.136373	-0.475405	-1.736616
5	1	0	0.855860	0.388288	-2.350266
6	1	0	0.629242	-1.363235	-2.131580
7	6	0	2.641630	-0.669797	-1.741311
8	1	0	2.923552	-1.533431	-1.131969
9	1	0	2.986816	-0.840170	-2.766472
10	1	0	3.148032	0.216258	-1.347409
11	6	0	-1.773483	-1.449557	-0.465839
12	1	0	-1.381417	-2.336921	0.038457
13	1	0	-2.821787	-1.317857	-0.183851
14	1	0	-1.713676	-1.589832	-1.548586
15	6	0	-1.418853	1.433427	-0.873792
16	1	0	-1.354300	1.258237	-1.951272
17	1	0	-2.461387	1.630753	-0.609447
18	1	0	-0.816999	2.307417	-0.610381

Table S19. Coordinates of optimized structures for tricoordinate and tetracoordinate phosphorus compounds, M06-2X/6-31+G(d,p).

PH_3 M06-2X/6-31+G(d,p), chloroform IEFPCM:
Sum of electronic and thermal Free Energies= -343.086297

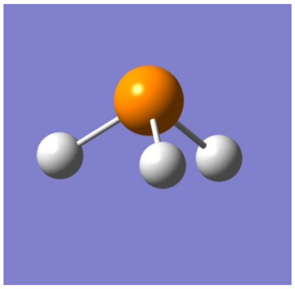

Center Number	Atomic Number	Atomic Type	Coordinates (Angstroms) X	Y	Z
1	15	0	0.000005	0.000083	0.123819
2	1	0	-0.000011	1.196124	-0.632695
3	1	0	-1.035517	-0.598096	-0.632749
4	1	0	1.035523	-0.598112	-0.632745

$PMeH_2$ M06-2X/6-31+G(d,p), chloroform IEFPCM:
Sum of electronic and thermal Free Energies= -382.358839

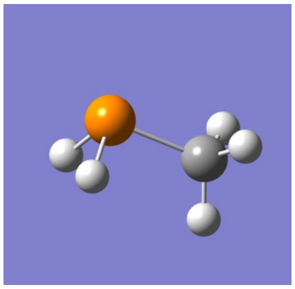

Center Number	Atomic Number	Atomic Type	Coordinates (Angstroms) X	Y	Z
1	15	0	0.664655	0.000028	-0.124084
2	1	0	0.932422	-1.034558	0.805468
3	1	0	0.932404	1.034523	0.805576
4	6	0	-1.192469	0.000007	0.026415
5	1	0	-1.585741	0.882484	-0.482574
6	1	0	-1.585729	-0.882443	-0.482632
7	1	0	-1.539121	-0.000029	1.060279

PMe_2H M06-2X/6-31+G(d,p), chloroform IEFPCM:
Sum of electronic and thermal Free Energies= -421.634342

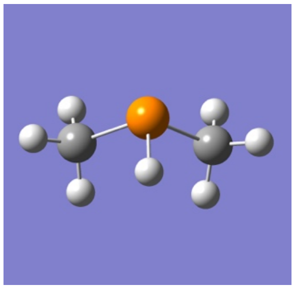

Center Number	Atomic Number	Atomic Type	Coordinates (Angstroms) X	Y	Z
1	15	0	0.000000	-0.665363	-0.108377
2	1	0	-0.000001	-1.094665	1.243436
3	6	0	1.418689	0.526423	0.022564
4	1	0	2.352767	-0.026197	0.148772
5	1	0	1.488259	1.093865	-0.909232
6	1	0	1.300883	1.225616	0.854446
7	6	0	-1.418688	0.526423	0.022562
8	1	0	-1.488257	1.093865	-0.909234
9	1	0	-2.352766	-0.026197	0.148769
10	1	0	-1.300883	1.225617	0.854444

PMe_3 M06-2X/6-31+G(d,p), chloroform IEFPCM:
Sum of electronic and thermal Free Energies= -460.912531

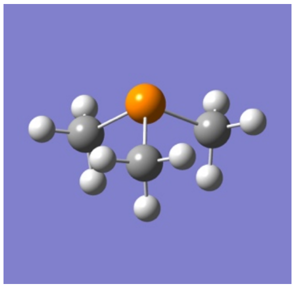

Center Number	Atomic Number	Atomic Type	Coordinates (Angstroms) X	Y	Z
1	15	0	0.000082	0.000135	-0.613309
2	6	0	-0.987888	-1.293208	0.273479
3	1	0	-0.633640	-2.287161	-0.012866
4	1	0	-2.039707	-1.213520	-0.014223
5	6	0	-0.626215	1.502122	0.273622
6	1	0	-0.032324	2.373726	-0.014540
7	1	0	-0.572979	1.378609	1.360951

8	1	0	-0.907010	-1.186015	1.360847
9	1	0	-1.664491	1.691306	-0.012092
10	6	0	1.614115	-0.208870	0.273485
11	1	0	1.480888	-0.192756	1.360870
12	1	0	2.297623	0.595084	-0.012747
13	1	0	2.071252	-1.159397	-0.014548

PPh_3 M06-2X/6-31+G(d,p), chloroform IEFPCM:
Sum of electronic and thermal Free Energies= -1035.775318

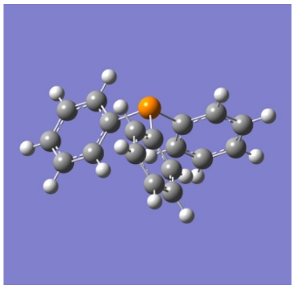

Center Number	Atomic Number	Atomic Type	Coordinates (Angstroms) X	Y	Z
1	15	0	-0.000227	-0.000430	-1.295959
2	6	0	-1.600736	-0.386172	-0.462574
3	6	0	-2.364502	-1.429442	-1.004047
4	6	0	-2.084207	0.291709	0.662133
5	6	0	-3.574059	-1.802934	-0.421746
6	1	0	-2.007173	-1.955553	-1.886521
7	6	0	-3.302128	-0.073129	1.237746
8	1	0	-1.509856	1.106298	1.094411
9	6	0	-4.046778	-1.122359	0.701031
10	1	0	-4.150654	-2.617265	-0.849484
11	1	0	-3.666166	0.462447	2.109234
12	1	0	-4.992942	-1.405596	1.151497
13	6	0	0.466477	1.578399	-0.463007
14	6	0	1.286618	1.658292	0.668090
15	6	0	-0.049875	2.761342	-1.009487
16	6	0	1.575502	2.895680	1.245311
17	1	0	1.700542	0.753401	1.104243
18	6	0	0.227488	3.995896	-0.425845
19	1	0	-0.677357	2.714769	-1.896789
20	6	0	1.043998	4.065184	0.703589
21	1	0	2.214064	2.943272	2.122163
22	1	0	-0.185556	4.902371	-0.857396
23	1	0	1.268408	5.026273	1.155622
24	6	0	1.135500	-1.194364	-0.465963
25	6	0	2.418439	-1.334677	-1.012840

26	6	0	0.795319	-1.949886	0.661765
27	6	0	3.349824	-2.193632	-0.432927
28	1	0	2.691188	-0.763723	-1.897626
29	6	0	1.723431	-2.820390	1.235138
30	1	0	-0.195520	-1.859165	1.098130
31	6	0	3.002158	-2.940939	0.693071
32	1	0	4.341541	-2.286079	-0.864759
33	1	0	1.445917	-3.401682	2.109190
34	1	0	3.722870	-3.617245	1.142032

Methoxyphospholane M06-2X/6-31+G(d,p), chloroform IEFPCM:
Sum of electronic and thermal Free Energies= -685.350482

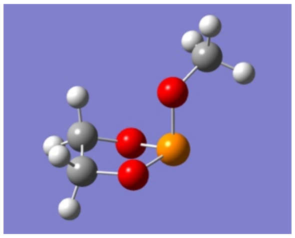

Center Number	Atomic Number	Atomic Type	Coordinates (Angstroms) X	Y	Z
1	8	0	-0.507662	1.027084	-0.703891
2	6	0	-1.384719	0.996568	0.433667
3	6	0	-1.932210	-0.433182	0.470662
4	1	0	-0.814003	1.232646	1.336448
5	1	0	-2.165772	1.741295	0.280739
6	1	0	-2.125936	-0.774942	1.488632
7	1	0	-2.836307	-0.535281	-0.134951
8	15	0	0.306864	-0.410639	-0.802788
9	8	0	-0.916715	-1.269873	-0.113552
10	6	0	2.560559	0.252351	0.378756
11	1	0	3.161619	-0.049825	1.236014
12	1	0	3.043511	-0.097322	-0.540594
13	1	0	2.483815	1.343442	0.354678
14	8	0	1.269661	-0.338419	0.540909

$P(OMe)_3$ isomer A M06-2X/6-31+G(d,p), chloroform IEFPCM:
Sum of electronic and thermal Free Energies= -686.524666

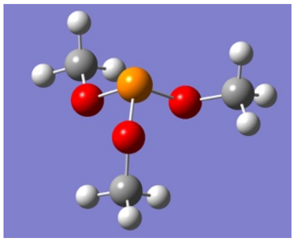

Center Number	Atomic Number	Atomic Type	Coordinates (Angstroms) X	Y	Z
1	15	0	-0.110606	-0.085800	-0.753841
2	6	0	1.583724	-1.873851	0.172466
3	1	0	1.643670	-2.637381	0.947994
4	1	0	1.484715	-2.359363	-0.804880
5	1	0	2.497936	-1.271769	0.184471
6	6	0	1.061079	1.872588	0.636798
7	1	0	1.878194	2.578513	0.490827
8	1	0	0.156368	2.402633	0.940186
9	1	0	1.332588	1.139964	1.401232
10	6	0	-2.495845	-0.400667	0.291026
11	1	0	-3.329208	0.195552	0.661787
12	1	0	-2.790732	-0.887911	-0.645543
13	1	0	-2.228671	-1.161168	1.029631
14	8	0	0.850646	1.223619	-0.625750
15	8	0	0.446144	-1.062911	0.464985
16	8	0	-1.403076	0.494445	0.081963

$P(OMe)_3$ isomer B M06-2X/6-31+G(d,p), chloroform IEFPCM:
Sum of electronic and thermal Free Energies= -686.523021

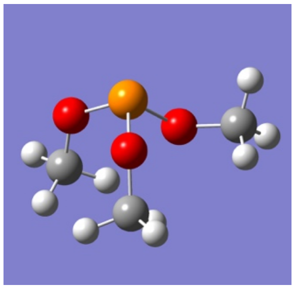

Center Number	Atomic Number	Atomic Type	Coordinates (Angstroms) X	Y	Z
1	6	0	2.253439	-0.662168	0.437337
2	8	0	0.851882	-0.913085	0.349555
3	15	0	0.068356	-0.265648	-0.962856
4	8	0	-1.433538	-0.745195	-0.545088

5	6	0	-1.964466	-0.817886	0.784451
6	8	0	0.144685	1.334169	-0.589866
7	1	0	2.642311	-1.292867	1.236534
8	1	0	2.759783	-0.915076	-0.500900
9	1	0	2.444562	0.389584	0.676340
10	1	0	-2.405680	0.142581	1.061694
11	1	0	-2.743278	-1.580753	0.769394
12	1	0	-1.188855	-1.098333	1.499120
13	6	0	-0.120158	1.868846	0.710313
14	1	0	-1.172943	2.153183	0.784186
15	1	0	0.502754	2.756500	0.829924
16	1	0	0.124604	1.144385	1.492946

$P(OMe)_3$ isomer C M06-2X/6-31+G(d,p), chloroform IEFPCM:
Sum of electronic and thermal Free Energies= -686.521162

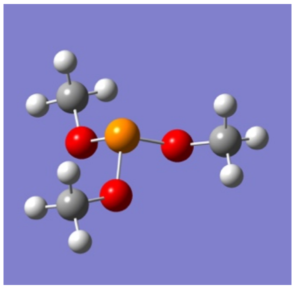

Center Number	Atomic Number	Atomic Type	Coordinates (Angstroms) X	Y	Z
1	15	0	0.002665	0.000692	0.445056
2	6	0	2.598282	0.058887	0.067417
3	1	0	3.352085	0.705187	-0.381316
4	1	0	2.685503	0.105625	1.158698
5	1	0	2.753775	-0.970465	-0.267264
6	6	0	-1.249178	-2.273844	0.069386
7	1	0	-1.070769	-3.250300	-0.380090
8	1	0	-1.254579	-2.373926	1.160569
9	1	0	-2.215802	-1.888426	-0.266588
10	6	0	-1.352458	2.215896	0.069120
11	1	0	-2.285618	2.545535	-0.386760
12	1	0	-1.445661	2.265594	1.159734
13	1	0	-0.536829	2.866883	-0.257585
14	8	0	-0.193645	-1.414353	-0.365039
15	8	0	1.324249	0.535883	-0.369646
16	8	0	-1.123978	0.875140	-0.368924

PCl_3 M06-2X/6-31+G(d,p), chloroform IEFPCM:
Sum of electronic and thermal Free Energies= -1721.891475

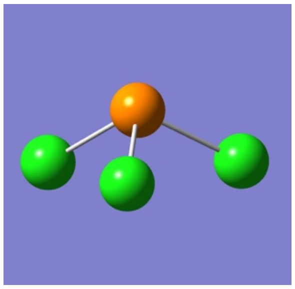

Center Number	Atomic Number	Atomic Type	Coordinates (Angstroms) X	Y	Z
1	15	0	0.000064	-0.000083	0.744597
2	17	0	-0.183463	1.821161	-0.219244
3	17	0	1.668891	-0.751318	-0.219328
4	17	0	-1.485478	-1.069769	-0.219224

H_3PO_4 M06-2X/6-31+G(d,p), water IEFPCM:
Sum of electronic and thermal Free Energies= -644.021221

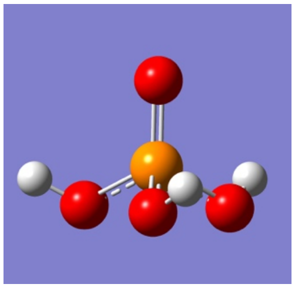

Center Number	Atomic Number	Atomic Type	Coordinates (Angstroms) X	Y	Z
1	15	0	-1.277689	1.090635	-0.158645
2	8	0	-1.125817	1.835208	1.242215
3	1	0	-0.276750	2.286987	1.358210
4	8	0	-0.713954	-0.362261	0.173654
5	1	0	-0.542544	-0.908256	-0.607996
6	8	0	-2.847997	0.828253	-0.232156
7	1	0	-3.353216	1.557292	-0.621887
8	8	0	-0.676399	1.772752	-1.324111

PH_4^+ BF_4^- M06-2X/6-31+G(d,p), chloroform IEFPCM:
Sum of electronic and thermal Free Energies= -767.994482

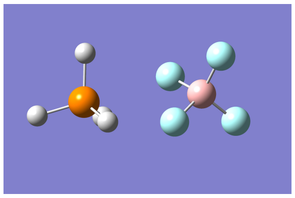

Center Number	Atomic Number	Atomic Type	Coordinates (Angstroms) X	Y	Z
1	15	0	-1.382539	0.988975	0.117484
2	1	0	-0.557532	-0.133920	0.100087
3	1	0	-1.102792	1.786251	1.219338
4	1	0	-1.234598	1.725421	-1.049981
5	1	0	-2.716078	0.594944	0.205800
6	5	0	2.105239	0.724157	-0.120738
7	9	0	1.081541	1.726533	-0.056794
8	9	0	2.980006	0.901596	0.943786
9	9	0	1.455935	-0.528573	-0.015946
10	9	0	2.759101	0.822677	-1.343033

$P(OPh)_4^+ PF_6^-$ M06-2X/6-31+G(d,p), chloroform IEFPCM:
Sum of electronic and thermal Free Energies= -2508.481539

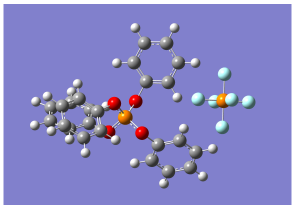

Center Number	Atomic Number	Atomic Type	Coordinates (Angstroms) X	Y	Z
1	15	0	1.377333	0.331603	-0.141449
2	8	0	2.562323	1.254947	-0.570937
3	8	0	0.356814	1.231614	0.609931
4	8	0	0.797682	-0.326293	-1.424955
5	8	0	1.823173	-0.807661	0.820236
6	6	0	2.755088	-0.634195	1.877523
7	6	0	3.807080	-1.535236	1.914275
8	6	0	2.583297	0.378471	2.807593
9	6	0	4.744307	-1.402419	2.935811
10	1	0	3.885803	-2.306474	1.155788
11	6	0	3.538686	0.499414	3.818015
12	1	0	1.729683	1.046397	2.755553
13	6	0	4.614673	-0.384232	3.882001
14	1	0	5.578136	-2.094170	2.987424

15	1	0	3.429863	1.284089	4.558667
16	1	0	5.349571	-0.284478	4.673353
17	6	0	3.766178	0.722363	-1.112661
18	6	0	4.900453	0.804195	-0.319925
19	6	0	3.766781	0.200649	-2.396987
20	6	0	6.098200	0.322589	-0.844282
21	1	0	4.838593	1.228829	0.677276
22	6	0	4.976004	-0.278559	-2.901803
23	1	0	2.855189	0.177256	-2.984183
24	6	0	6.135494	-0.219754	-2.129440
25	1	0	7.000767	0.373042	-0.244883
26	1	0	5.006795	-0.691981	-3.904000
27	1	0	7.071357	-0.592536	-2.531445
28	6	0	-0.000101	-1.511816	-1.401201
29	6	0	-1.364609	-1.392174	-1.213065
30	6	0	0.649219	-2.717641	-1.610787
31	6	0	-2.123616	-2.563090	-1.224626
32	1	0	-1.836177	-0.426227	-1.064585
33	6	0	-0.127274	-3.874539	-1.627067
34	1	0	1.724366	-2.741150	-1.755150
35	6	0	-1.507596	-3.796444	-1.432580
36	1	0	-3.193592	-2.492860	-1.061117
37	1	0	0.349685	-4.835179	-1.789275
38	1	0	-2.104850	-4.701943	-1.441021
39	6	0	-0.467902	2.199947	-0.040764
40	6	0	0.117384	3.269527	-0.701377
41	6	0	-1.835421	2.020876	0.073640
42	6	0	-0.731987	4.202292	-1.296858
43	1	0	1.195689	3.377626	-0.742728
44	6	0	-2.664817	2.965089	-0.530303
45	1	0	-2.244228	1.174504	0.616803
46	6	0	-2.115858	4.049380	-1.214858
47	1	0	-0.304449	5.049950	-1.821563
48	1	0	-3.738153	2.832401	-0.455767
49	1	0	-2.767885	4.779300	-1.682541
50	15	0	-4.876451	-0.347821	0.565755
51	9	0	-4.672198	1.204464	1.021990
52	9	0	-6.317295	0.026097	-0.079143
53	9	0	-4.147122	-0.014151	-0.859923
54	9	0	-5.062726	-1.897540	0.102812
55	9	0	-5.580273	-0.680211	1.988614
56	9	0	-3.413863	-0.714454	1.198203

O=P(OCH2)3P=O M06-2X/6-31+G(d,p), chloroform IEFPCM:
Sum of electronic and thermal Free Energies= -1176.518168

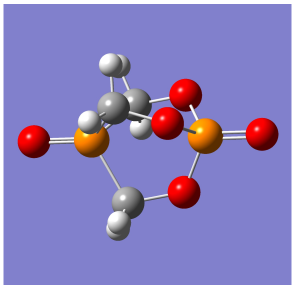

Center Number	Atomic Number	Atomic Type	Coordinates (Angstroms) X	Y	Z
1	6	0	-1.041716	0.472540	-1.345979
2	1	0	-1.409363	1.036049	-2.205181
3	6	0	-1.329730	-1.631854	0.504978
4	1	0	-0.626182	-2.358796	0.095962
5	6	0	-3.412353	0.226717	0.148979
6	1	0	-4.266995	-0.363971	0.482273
7	15	0	-1.118537	0.877517	1.248282
8	8	0	-0.544833	1.720382	2.295913
9	8	0	-0.555627	-0.628498	1.198907
10	8	0	-2.711980	0.673283	1.331232
11	8	0	-0.891609	1.406335	-0.253712
12	1	0	-3.761424	1.091101	-0.422431
13	1	0	-0.073504	0.024418	-1.585445
14	1	0	-2.006857	-2.127050	1.206430
15	15	0	-2.271711	-0.818995	-0.861599
16	8	0	-2.856906	-1.678732	-1.930616

$P(OMe)_4^+ BF_4^-$ isomer A M06-2X/6-31+G(d,p), chloroform IEFPCM:
Sum of electronic and thermal Free Energies= -1225.935583

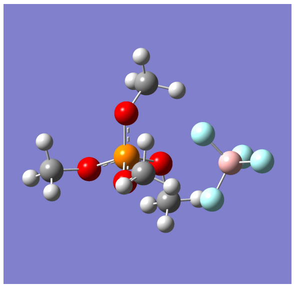

Center Number	Atomic Number	Atomic Type	Coordinates (Angstroms) X	Y	Z
1	15	0	-0.218580	0.823881	-0.451880
2	8	0	0.455441	1.858843	0.476336
3	8	0	0.010384	-0.561157	0.248124

4	8	0	-1.754690	0.904392	-0.646981
5	8	0	0.460098	0.904296	-1.847104
6	6	0	0.094934	2.027024	1.875434
7	1	0	0.586271	1.243596	2.451637
8	1	0	0.459554	3.016388	2.137234
9	1	0	-0.988711	1.992298	1.981296
10	6	0	1.759949	1.524295	-2.068406
11	1	0	2.521034	0.972319	-1.516939
12	1	0	1.929464	1.446718	-3.139010
13	1	0	1.703705	2.564258	-1.752611
14	6	0	-0.600667	-1.771568	-0.262856
15	1	0	-0.238160	-2.573411	0.375026
16	1	0	-1.685076	-1.685226	-0.195612
17	1	0	-0.285493	-1.936404	-1.294489
18	6	0	-2.477491	1.684679	-1.644112
19	1	0	-1.855374	1.811067	-2.528330
20	1	0	-3.367476	1.099761	-1.864331
21	1	0	-2.719688	2.646077	-1.200370
22	9	0	0.505512	4.709791	0.312672
23	9	0	-1.604740	3.804302	0.392583
24	9	0	-0.348301	3.588093	-1.509345
25	9	0	-1.191272	5.642093	-0.924667
26	5	0	-0.654721	4.453161	-0.427387

$P(OMe)_4^+ BF_4^-$ isomer B M06-2X/6-31+G(d,p), chloroform IEFPCM:
Sum of electronic and thermal Free Energies= -1225.928628

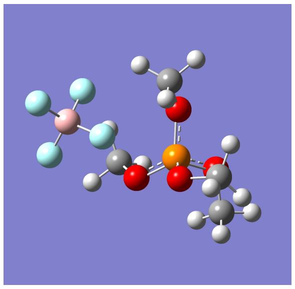

Center Number	Atomic Number	Atomic Type	Coordinates (Angstroms) X	Y	Z
1	15	0	-0.457129	0.717267	-0.121526
2	8	0	0.203263	1.300686	1.148248
3	8	0	-0.035220	-0.796397	-0.028780
4	8	0	-2.008943	0.853637	-0.154238
5	8	0	0.015346	1.307665	-1.472881
6	6	0	-0.341769	2.299896	2.060912
7	1	0	-1.393163	2.475806	1.839387
8	1	0	-0.201692	1.887118	3.057704

9	1	0	0.227932	3.211639	1.907074
10	6	0	1.429744	1.536444	-1.749119
11	1	0	1.971671	0.591676	-1.671328
12	1	0	1.455687	1.919117	-2.764819
13	1	0	1.807144	2.283984	-1.053298
14	6	0	-0.243359	-1.670753	-1.167505
15	1	0	0.036282	-2.663897	-0.826251
16	1	0	-1.292294	-1.654261	-1.469964
17	1	0	0.393832	-1.356805	-1.994864
18	6	0	-2.833684	0.093849	0.769343
19	1	0	-2.473719	0.217554	1.792418
20	1	0	-3.832767	0.508617	0.668680
21	1	0	-2.823058	-0.958743	0.485616
22	9	0	-0.476387	5.716161	-0.504431
23	9	0	-1.163726	3.531968	-0.316163
24	9	0	1.041196	4.060593	-0.007207
25	9	0	0.124923	4.181880	-2.107491
26	5	0	-0.121852	4.388302	-0.745209

(i-PrO)$_2$P(O)H isomer A M06-2X/6-31+G(d,p), chloroform IEFPCM:
Sum of electronic and thermal Free Energies= -804.375180

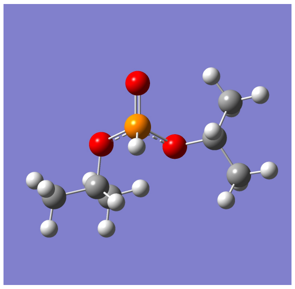

Center Number	Atomic Number	Atomic Type	Coordinates (Angstroms) X	Y	Z
1	15	0	-0.156176	0.266712	-0.976918
2	1	0	0.118274	0.244752	-2.352023
3	8	0	0.587685	1.311197	-0.238752
4	8	0	0.130037	-1.212649	-0.468891
5	8	0	-1.756016	0.342589	-0.915624
6	6	0	-2.422950	1.571007	-1.321389
7	1	0	-1.792102	2.077678	-2.065065
8	6	0	-0.520306	-2.353693	-1.098888
9	1	0	-0.929499	-2.029981	-2.065338
10	6	0	0.552342	-3.402525	-1.319948
11	1	0	0.987394	-3.698128	-0.360868
12	1	0	0.116643	-4.286321	-1.793634
13	1	0	1.347212	-3.015392	-1.961767

14	6	0	-1.645598	-2.822610	-0.195318
15	1	0	-1.240889	-3.117354	0.777281
16	1	0	-2.377836	-2.025646	-0.049936
17	1	0	-2.145055	-3.686512	-0.642774
18	6	0	-3.733341	1.157154	-1.961903
19	1	0	-4.282523	2.042496	-2.292910
20	1	0	-3.557211	0.510036	-2.824483
21	1	0	-4.347482	0.617020	-1.235051
22	6	0	-2.602116	2.459004	-0.103272
23	1	0	-3.216670	1.944695	0.641619
24	1	0	-1.634086	2.700606	0.340949
25	1	0	-3.103467	3.387529	-0.390476

(*i*-PrO)$_2$P(O)H isomer B M06-2X/6-31+G(d,p), chloroform IEFPCM:
Sum of electronic and thermal Free Energies=   -804.377586

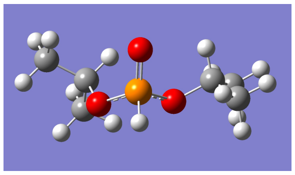

Center Number	Atomic Number	Atomic Type	Coordinates (Angstroms) X	Y	Z
1	15	0	-0.751650	-0.086706	-2.484960
2	1	0	-1.623054	-0.849108	-3.260779
3	8	0	-0.282996	1.173181	-3.114642
4	8	0	0.341751	-1.190707	-2.139400
5	8	0	-1.478958	0.128695	-1.070721
6	6	0	-2.161793	1.375917	-0.766670
7	1	0	-1.524495	2.192581	-1.120371
8	6	0	1.453100	-0.839135	-1.262569
9	1	0	1.296517	0.182891	-0.896320
10	6	0	1.431866	-1.811626	-0.099503
11	1	0	1.549504	-2.835519	-0.466709
12	1	0	2.253572	-1.590326	0.587198
13	1	0	0.487444	-1.737408	0.444900
14	6	0	2.724909	-0.901573	-2.086195
15	1	0	2.863491	-1.911839	-2.482660
16	1	0	2.676511	-0.197521	-2.920164
17	1	0	3.586762	-0.649318	-1.462249
18	6	0	-3.501846	1.409742	-1.482236
19	1	0	-4.035869	2.329773	-1.229546
20	1	0	-3.372273	1.384443	-2.568548
21	1	0	-4.112805	0.554783	-1.177176
22	6	0	-2.294038	1.425482	0.742664

23	1	0	-2.907494	0.591306	1.096201
24	1	0	-1.311388	1.365926	1.216777
25	1	0	-2.771514	2.362050	1.042499

(i-PrO)$_2$P(O)H isomer C M06-2X/6-31+G(d,p), chloroform IEFPCM:
Sum of electronic and thermal Free Energies= -804.377756

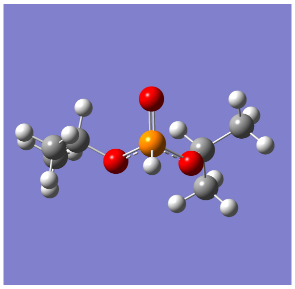

Center Number	Atomic Number	Atomic Type	Coordinates (Angstroms) X	Y	Z
1	15	0	-0.870505	-0.963102	-1.293791
2	1	0	-1.187268	-2.241282	-0.836853
3	8	0	-0.540502	-0.872839	-2.738279
4	8	0	0.289557	-0.415757	-0.329145
5	8	0	-2.146659	-0.165522	-0.775408
6	6	0	-2.224629	1.274846	-0.991118
7	1	0	-1.259540	1.620168	-1.381702
8	6	0	1.683427	-0.467302	-0.739994
9	1	0	1.731454	-0.125202	-1.778735
10	6	0	2.424322	0.494876	0.167336
11	1	0	2.349808	0.166178	1.208277
12	1	0	3.480265	0.530319	-0.113259
13	1	0	2.005971	1.500823	0.084712
14	6	0	2.194145	-1.894705	-0.635047
15	1	0	2.083922	-2.259257	0.390755
16	1	0	1.649827	-2.562210	-1.310162
17	1	0	3.252156	-1.932306	-0.908211
18	6	0	-3.316631	1.531860	-2.011811
19	1	0	-3.409198	2.605609	-2.197027
20	1	0	-3.085887	1.031055	-2.954909
21	1	0	-4.273743	1.157687	-1.636346
22	6	0	-2.490819	1.912772	0.358294
23	1	0	-3.434105	1.539478	0.768099
24	1	0	-1.684724	1.680427	1.058245
25	1	0	-2.562365	2.998493	0.250016

(i-PrO)$_2$P(O)H isomer D M06-2X/6-31+G(d,p), chloroform IEFPCM:
Sum of electronic and thermal Free Energies= -804.375028

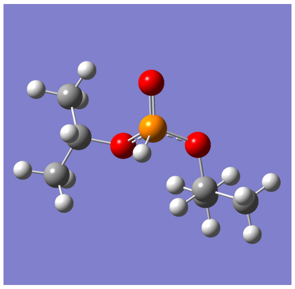

Center Number	Atomic Number	Atomic Type	Coordinates (Angstroms) X	Y	Z
1	15	0	-0.256619	-0.389163	-1.758384
2	1	0	-0.036398	-1.464057	-2.631844
3	8	0	0.081017	0.929093	-2.339110
4	8	0	0.537525	-0.816090	-0.433071
5	8	0	-1.773622	-0.525044	-1.301526
6	6	0	-2.267912	-1.766497	-0.721840
7	1	0	-1.553178	-2.566976	-0.955644
8	6	0	1.992924	-0.819278	-0.450522
9	1	0	2.326258	-1.012929	-1.479635
10	6	0	2.493724	0.538436	0.008129
11	1	0	2.145247	0.736056	1.026262
12	1	0	3.587232	0.552456	0.003011
13	1	0	2.125364	1.326271	-0.652547
14	6	0	2.430764	-1.957661	0.450131
15	1	0	2.065549	-1.787486	1.467423
16	1	0	2.038227	-2.911542	0.089772
17	1	0	3.522163	-2.013978	0.476333
18	6	0	-2.366709	-1.587517	0.781846
19	1	0	-2.744287	-2.505579	1.240773
20	1	0	-1.387059	-1.356250	1.205065
21	1	0	-3.057191	-0.771096	1.012851
22	6	0	-3.601103	-2.057816	-1.383024
23	1	0	-4.301708	-1.241992	-1.183016
24	1	0	-3.483672	-2.162903	-2.464098
25	1	0	-4.020410	-2.984803	-0.982879

(MeO)$_2$P(O)H isomer A M06-2X/6-31+G(d,p), chloroform IEFPCM:
Sum of electronic and thermal Free Energies= -647.278831

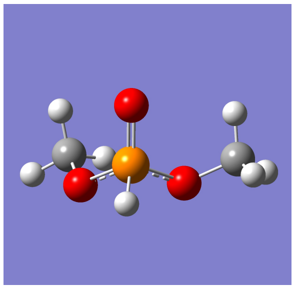

Center Number	Atomic Number	Atomic Type	Coordinates (Angstroms) X	Y	Z
1	15	0	0.002948	0.500975	0.421257
2	8	0	-1.440037	-0.150215	0.600694
3	8	0	0.933158	-0.797098	0.259181
4	8	0	0.174317	1.504116	-0.656841
5	6	0	-2.072359	-0.763485	-0.538333
6	1	0	-3.052333	-1.093555	-0.199923
7	1	0	-2.180069	-0.035972	-1.345265
8	1	0	-1.484791	-1.620596	-0.874388
9	6	0	2.254694	-0.652533	-0.287696
10	1	0	2.629449	-1.661469	-0.450063
11	1	0	2.215124	-0.106269	-1.231289
12	1	0	2.901831	-0.129253	0.421128
13	1	0	0.219203	0.970956	1.715309

$(MeO)_2P(O)H$ isomer B M06-2X/6-31+G(d,p), chloroform IEFPCM:
Sum of electronic and thermal Free Energies= -647.276646

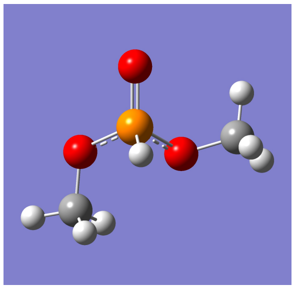

Center Number	Atomic Number	Atomic Type	Coordinates (Angstroms) X	Y	Z
1	15	0	-0.021073	-0.513241	0.238410
2	8	0	1.460541	-0.297690	-0.302226
3	8	0	-0.734063	0.848127	-0.219639
4	8	0	-0.653200	-1.769263	-0.215745
5	6	0	2.172668	0.907818	0.024398
6	1	0	3.197031	0.756796	-0.309929

7	1	0	1.723954	1.755949	-0.494882
8	1	0	2.161733	1.080387	1.104848
9	6	0	-2.148937	0.986336	-0.006282
10	1	0	-2.418815	1.975449	-0.370953
11	1	0	-2.688835	0.218828	-0.563593
12	1	0	-2.383582	0.911295	1.059543
13	1	0	0.076578	-0.388192	1.631526

$(MeO)_2P(O)H$ isomer C M06-2X/6-31+G(d,p), chloroform IEFPCM:
Sum of electronic and thermal Free Energies= -647.277217

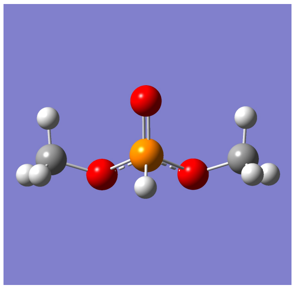

Center Number	Atomic Number	Atomic Type	Coordinates (Angstroms) X	Y	Z
1	15	0	-0.000020	0.168286	0.285183
2	8	0	-1.207076	-0.783023	-0.144449
3	8	0	1.207017	-0.783105	-0.144320
4	8	0	0.000071	1.542126	-0.269677
5	6	0	-2.541330	-0.248128	-0.095052
6	1	0	-3.196350	-1.034440	-0.464496
7	1	0	-2.813471	0.003522	0.933851
8	1	0	-2.617229	0.635566	-0.730973
9	6	0	2.541266	-0.248174	-0.095153
10	1	0	3.196250	-1.034491	-0.464650
11	1	0	2.617048	0.635487	-0.731133
12	1	0	2.813548	0.003539	0.933697
13	1	0	-0.000105	0.152705	1.685978

$Ph_4P^+ Cl^-$ M06-2X/6-31+G(d,p), chloroform IEFPCM:
Sum of electronic and thermal Free Energies= -1727.435636

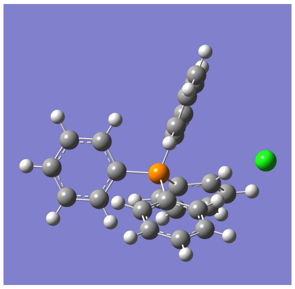

Center Number	Atomic Number	Atomic Type	Coordinates (Angstroms) X	Y	Z
1	15	0	0.779294	-0.012769	-0.030353
2	6	0	0.374297	-1.494651	-0.984138
3	6	0	-0.978233	-1.808124	-1.179114
4	6	0	1.378763	-2.313461	-1.513867
5	6	0	-1.315147	-2.930891	-1.930376
6	1	0	-1.775975	-1.201311	-0.747443
7	6	0	1.026055	-3.436257	-2.259201
8	1	0	2.427055	-2.095052	-1.341410
9	6	0	-0.317679	-3.741465	-2.472239
10	1	0	-2.362922	-3.168604	-2.081702
11	1	0	1.803619	-4.073302	-2.667162
12	1	0	-0.587041	-4.617519	-3.053575
13	6	0	0.430310	1.499233	-0.958522
14	6	0	-0.823084	1.627819	-1.571523
15	6	0	1.347454	2.556924	-0.985373
16	6	0	-1.141857	2.811591	-2.230641
17	1	0	-1.573646	0.843566	-1.503408
18	6	0	1.017238	3.734494	-1.653038
19	1	0	2.307665	2.474548	-0.486923
20	6	0	-0.222181	3.859268	-2.279106
21	1	0	-2.116783	2.912641	-2.695313
22	1	0	1.728082	4.553641	-1.677023
23	1	0	-0.476449	4.779163	-2.795869
24	6	0	2.531805	-0.037776	0.408336
25	6	0	2.917829	-0.232881	1.737680
26	6	0	3.501248	0.119178	-0.593931
27	6	0	4.273395	-0.275277	2.062425
28	1	0	2.170182	-0.346945	2.516728
29	6	0	4.850571	0.074684	-0.260543
30	1	0	3.206004	0.282602	-1.627649
31	6	0	5.235862	-0.123387	1.067192
32	1	0	4.572788	-0.424405	3.094191
33	1	0	5.599850	0.196986	-1.035231
34	1	0	6.289611	-0.155536	1.324103
35	6	0	-0.222065	-0.005775	1.466482

36	6	0	-0.604276	1.215761	2.030836
37	6	0	-0.578538	-1.216127	2.071931
38	6	0	-1.349367	1.221726	3.206479
39	1	0	-0.343411	2.152768	1.547132
40	6	0	-1.325927	-1.198452	3.245523
41	1	0	-0.289633	-2.163212	1.625284
42	6	0	-1.710925	0.017764	3.809787
43	1	0	-1.662023	2.165533	3.639546
44	1	0	-1.617735	-2.133665	3.710809
45	1	0	-2.305899	0.026628	4.717169
46	17	0	-3.831137	0.072068	-0.333965

Ph_4P^+ Br^- M06-2X/6-31+G(d,p), chloroform IEFPCM:
Sum of electronic and thermal Free Energies= -3839.079102

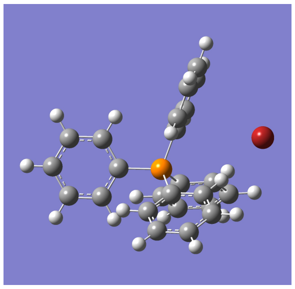

Center Number	Atomic Number	Atomic Type	Coordinates (Angstroms) X	Y	Z
1	15	0	0.788531	-0.003121	-0.025273
2	6	0	0.407919	-1.468437	-1.019850
3	6	0	-0.938262	-1.718296	-1.321620
4	6	0	1.407734	-2.339614	-1.466447
5	6	0	-1.270768	-2.824548	-2.097327
6	1	0	-1.736273	-1.090475	-0.925689
7	6	0	1.060624	-3.442090	-2.245506
8	1	0	2.447982	-2.179688	-1.206071
9	6	0	-0.274293	-3.680199	-2.567479
10	1	0	-2.314552	-3.017322	-2.322497
11	1	0	1.836137	-4.116957	-2.591949
12	1	0	-0.539221	-4.541138	-3.172922
13	6	0	0.423508	1.519680	-0.935523
14	6	0	-0.916725	1.779002	-1.254826
15	6	0	1.426300	2.426854	-1.295122
16	6	0	-1.239968	2.932130	-1.963169
17	1	0	-1.718460	1.121747	-0.919509
18	6	0	1.088784	3.575938	-2.008560
19	1	0	2.460710	2.257913	-1.017669
20	6	0	-0.239430	3.824056	-2.350077

21	1	0	-2.279480	3.131966	-2.201514
22	1	0	1.866404	4.278541	-2.288898
23	1	0	-0.496771	4.720805	-2.904703
24	6	0	2.535335	-0.022409	0.437490
25	6	0	2.892494	-0.068223	1.788695
26	6	0	3.529742	0.010868	-0.552598
27	6	0	4.240129	-0.080630	2.146670
28	1	0	2.127496	-0.093683	2.558575
29	6	0	4.871105	-0.001890	-0.186219
30	1	0	3.261673	0.048114	-1.605682
31	6	0	5.225927	-0.047363	1.163609
32	1	0	4.514825	-0.115714	3.195374
33	1	0	5.637737	0.024376	-0.953105
34	1	0	6.273437	-0.056585	1.446474
35	6	0	-0.233845	-0.039141	1.454097
36	6	0	-0.605046	1.162744	2.064921
37	6	0	-0.621326	-1.267799	1.997906
38	6	0	-1.370423	1.130361	3.226001
39	1	0	-0.315021	2.115650	1.631494
40	6	0	-1.389069	-1.289056	3.157716
41	1	0	-0.342517	-2.199246	1.513451
42	6	0	-1.765295	-0.092525	3.766542
43	1	0	-1.678418	2.059943	3.692388
44	1	0	-1.711304	-2.238559	3.571343
45	1	0	-2.381467	-0.112992	4.659674
46	35	0	-3.709554	0.018752	0.260376

Me_4P^+ Cl^- M06-2X/6-31+G(d,p), chloroform IEFPCM:
Sum of electronic and thermal Free Energies= -960.980847

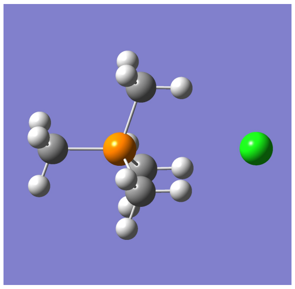

Center Number	Atomic Number	Atomic Type	Coordinates (Angstroms) X	Y	Z
1	15	0	1.476186	-0.000811	-0.000537
2	6	0	3.285136	0.002944	0.008332
3	1	0	3.657717	-0.979461	-0.289757
4	1	0	3.654045	0.757149	-0.690144
5	1	0	3.644152	0.235743	1.013485

6	6	0	0.896822	1.622140	0.521142
7	1	0	1.269978	2.377338	-0.174268
8	1	0	-0.196862	1.602182	0.511317
9	1	0	1.265940	1.834361	1.527042
10	6	0	0.897327	-1.272228	1.135539
11	1	0	-0.196171	-1.242397	1.136464
12	1	0	1.250143	-2.248094	0.793490
13	1	0	1.286934	-1.069796	2.135563
14	6	0	0.898351	-0.354383	-1.669232
15	1	0	1.268788	-1.333369	-1.981997
16	1	0	-0.195570	-0.350820	-1.645693
17	1	0	1.266728	0.415027	-2.351887
18	17	0	-2.214302	0.004264	0.020862

Me_4P^+ Br^- M06-2X/6-31+G(d,p), chloroform IEFPCM:
Sum of electronic and thermal Free Energies= -3072.584941

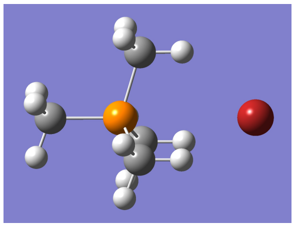

Center Number	Atomic Number	Atomic Type	Coordinates (Angstroms) X	Y	Z
1	15	0	1.418411	0.000019	0.000194
2	6	0	3.236908	0.000111	0.000378
3	1	0	3.609677	-0.976633	-0.317902
4	1	0	3.609513	0.764111	-0.686463
5	1	0	3.609688	0.212973	1.005356
6	6	0	0.896273	1.641510	0.532914
7	1	0	1.289514	2.385078	-0.165442
8	1	0	-0.199864	1.648200	0.534672
9	1	0	1.289020	1.833120	1.535066
10	6	0	0.896160	-1.282346	1.155144
11	1	0	-0.199996	-1.286843	1.160907
12	1	0	1.288022	-2.246079	0.819080
13	1	0	1.290383	-1.050305	2.148115
14	6	0	0.897180	-0.359554	-1.688118
15	1	0	1.291630	-1.335631	-1.982955
16	1	0	-0.198940	-0.362392	-1.695727
17	1	0	1.289549	0.413098	-2.354678
18	35	0	-2.097782	0.001351	-0.000820

(i-PrO)$_2$P(O)Me isomer A M06-2X/6-31+G(d,p), chloroform IEFPCM:
Sum of electronic and thermal Free Energies= -843.663617

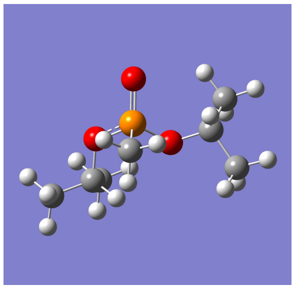

Center Number	Atomic Number	Atomic Type	Coordinates (Angstroms) X	Y	Z
1	15	0	-0.133008	0.256754	-1.161978
2	8	0	0.589945	1.315964	-0.415542
3	8	0	0.138240	-1.204749	-0.577968
4	8	0	-1.739921	0.337289	-1.090583
5	6	0	-2.398822	1.612120	-1.314624
6	1	0	-1.698697	2.284795	-1.827398
7	6	0	-0.630833	-2.363720	-0.998916
8	1	0	-1.263327	-2.077105	-1.847832
9	6	0	0.360865	-3.429513	-1.425581
10	1	0	1.012204	-3.690282	-0.586345
11	1	0	-0.171029	-4.328530	-1.748778
12	1	0	0.981495	-3.073212	-2.252134
13	6	0	-1.510130	-2.786612	0.163288
14	1	0	-0.888555	-3.041295	1.026752
15	1	0	-2.187690	-1.975868	0.440353
16	1	0	-2.101588	-3.663687	-0.114282
17	6	0	-3.595005	1.339821	-2.206532
18	1	0	-4.131817	2.270417	-2.409395
19	1	0	-3.277403	0.903705	-3.157372
20	1	0	-4.278452	0.643442	-1.711285
21	6	0	-2.770692	2.202066	0.033331
22	1	0	-3.449601	1.524409	0.560019
23	1	0	-1.875538	2.351853	0.641128
24	1	0	-3.271726	3.164636	-0.102973
25	6	0	0.267632	0.200879	-2.918172
26	1	0	-0.039337	1.138383	-3.388286
27	1	0	1.347567	0.086420	-3.029890
28	1	0	-0.244289	-0.629809	-3.409301

(i-PrO)$_2$P(O)Me isomer B M06-2X/6-31+G(d,p), chloroform IEFPCM:
Sum of electronic and thermal Free Energies= -843.666861

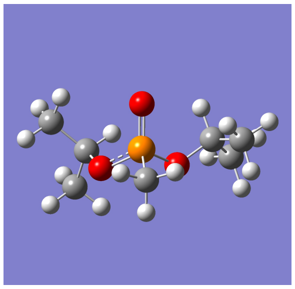

Center Number	Atomic Number	Atomic Type	Coordinates (Angstroms) X	Y	Z
1	15	0	0.044370	0.467640	-2.573403
2	8	0	0.658850	1.730595	-2.079977
3	8	0	0.875917	-0.872585	-2.295583
4	8	0	-1.368971	0.141939	-1.867344
5	6	0	-2.123684	1.181592	-1.193083
6	1	0	-1.419083	1.763623	-0.590163
7	6	0	1.308393	-1.150929	-0.935258
8	1	0	0.709813	-0.537147	-0.249815
9	6	0	1.032188	-2.619510	-0.677344
10	1	0	1.592693	-3.235205	-1.387360
11	1	0	1.342312	-2.886593	0.336651
12	1	0	-0.033272	-2.834853	-0.788529
13	6	0	2.774947	-0.779365	-0.814534
14	1	0	3.368938	-1.375822	-1.513730
15	1	0	2.918033	0.279859	-1.040376
16	1	0	3.129690	-0.975486	0.201286
17	6	0	-2.791742	2.081736	-2.219312
18	1	0	-3.407123	2.831832	-1.714855
19	1	0	-2.046425	2.608480	-2.822615
20	1	0	-3.433698	1.488335	-2.878344
21	6	0	-3.117836	0.465477	-0.299322
22	1	0	-3.805988	-0.133695	-0.903337
23	1	0	-2.599322	-0.194462	0.400579
24	1	0	-3.698358	1.195146	0.271402
25	6	0	-0.235277	0.360113	-4.341028
26	1	0	0.718420	0.454205	-4.863420
27	1	0	-0.700248	-0.595966	-4.586542
28	1	0	-0.894469	1.176934	-4.642118

(i-PrO)$_2$P(O)Me isomer C M06-2X/6-31+G(d,p), chloroform IEFPCM:
Sum of electronic and thermal Free Energies= -843.663337

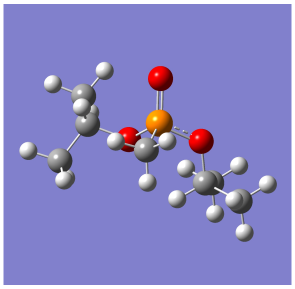

Center Number	Atomic Number	Atomic Type	Coordinates (Angstroms) X	Y	Z
1	15	0	-0.239164	-0.533036	-1.873348
2	8	0	0.096962	0.783526	-2.469363
3	8	0	0.562391	-0.904653	-0.526983
4	8	0	-1.753576	-0.614784	-1.373535
5	6	0	-2.246214	-1.717863	-0.565852
6	1	0	-1.471146	-2.492431	-0.517458
7	6	0	2.004591	-0.736753	-0.487528
8	1	0	2.366025	-0.554236	-1.507892
9	6	0	2.313940	0.470251	0.378371
10	1	0	1.928217	0.310136	1.389868
11	1	0	3.394970	0.625590	0.435553
12	1	0	1.849983	1.366158	-0.040331
13	6	0	2.592224	-2.029819	0.045528
14	1	0	2.209525	-2.226502	1.051566
15	1	0	2.328268	-2.871302	-0.600797
16	1	0	3.681913	-1.955402	0.094923
17	6	0	-2.516828	-1.188356	0.830176
18	1	0	-2.895017	-1.991661	1.468794
19	1	0	-1.599417	-0.789391	1.268765
20	1	0	-3.265685	-0.392034	0.787230
21	6	0	-3.482764	-2.262067	-1.255926
22	1	0	-4.241628	-1.478133	-1.334224
23	1	0	-3.241434	-2.616639	-2.261574
24	1	0	-3.896964	-3.094586	-0.680720
25	6	0	0.021701	-1.925460	-2.987580
26	1	0	-0.550077	-1.758048	-3.902409
27	1	0	1.082754	-1.991824	-3.240995
28	1	0	-0.293322	-2.861973	-2.521549

Ph_3PO M06-2X/6-31+G(d,p), chloroform IEFPCM:
Sum of electronic and thermal Free Energies= -1111.004592

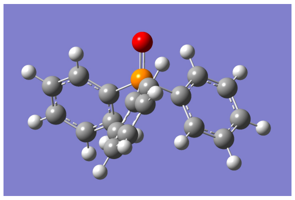

Center Number	Atomic Number	Atomic Type	Coordinates (Angstroms) X	Y	Z
1	15	0	-1.241820	1.379856	0.045577
2	8	0	-2.729526	1.435825	-0.176511
3	6	0	-0.294209	2.104525	-1.325742
4	6	0	-0.794482	1.920096	-2.619989
5	6	0	0.901930	2.803238	-1.134630
6	6	0	-0.096391	2.422534	-3.715548
7	1	0	-1.733432	1.391934	-2.761801
8	6	0	1.598900	3.304192	-2.233953
9	1	0	1.288485	2.963097	-0.131532
10	6	0	1.101452	3.112358	-3.522386
11	1	0	-0.487699	2.279977	-4.717718
12	1	0	2.525672	3.848308	-2.082769
13	1	0	1.644181	3.505410	-4.376343
14	6	0	-0.718513	2.271315	1.540359
15	6	0	-1.428666	3.428045	1.881527
16	6	0	0.360050	1.858078	2.328637
17	6	0	-1.055295	4.170887	2.999302
18	1	0	-2.276328	3.734234	1.274756
19	6	0	0.732247	2.604905	3.445927
20	1	0	0.906557	0.952775	2.077706
21	6	0	0.026333	3.760284	3.779819
22	1	0	-1.609783	5.065614	3.263583
23	1	0	1.567992	2.280641	4.057612
24	1	0	0.315147	4.338281	4.652070
25	6	0	-0.620764	-0.317652	0.237192
26	6	0	-1.453584	-1.236578	0.885934
27	6	0	0.642842	-0.717370	-0.209194
28	6	0	-1.019993	-2.543698	1.095142
29	1	0	-2.441239	-0.925723	1.215131
30	6	0	1.074618	-2.026273	0.003664
31	1	0	1.288673	-0.012920	-0.726889
32	6	0	0.244973	-2.937473	0.656353
33	1	0	-1.669045	-3.254980	1.595726
34	1	0	2.054984	-2.334379	-0.345338
35	1	0	0.581796	-3.956613	0.818258

$(MeO)_2P(O)Me$ isomer A M06-2X/6-31+G(d,p), chloroform IEFPCM:
Sum of electronic and thermal Free Energies= -686.566746

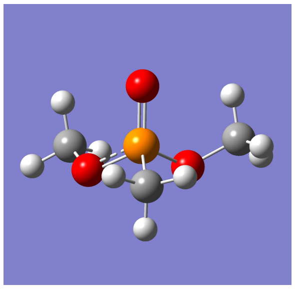

Center Number	Atomic Number	Atomic Type	Coordinates (Angstroms) X	Y	Z
1	15	0	0.016612	0.361368	0.152817
2	8	0	-0.110965	0.186014	1.623877
3	6	0	-0.411247	1.979154	-0.488389
4	1	0	-0.273755	1.999681	-1.570456
5	1	0	-1.455154	2.189022	-0.245014
6	1	0	0.223508	2.731800	-0.017656
7	8	0	1.478783	0.134729	-0.459904
8	8	0	-0.846734	-0.714517	-0.685353
9	6	0	2.176117	-1.075351	-0.120398
10	1	0	1.643320	-1.939479	-0.524526
11	1	0	3.161229	-0.999902	-0.576992
12	1	0	2.271310	-1.163735	0.963918
13	6	0	-2.091277	-1.182239	-0.145818
14	1	0	-2.416099	-2.000105	-0.787118
15	1	0	-1.951756	-1.535132	0.877336
16	1	0	-2.839184	-0.384179	-0.164416

$(MeO)_2P(O)Me$ isomer B M06-2X/6-31+G(d,p), chloroform IEFPCM:
Sum of electronic and thermal Free Energies= -686.564238

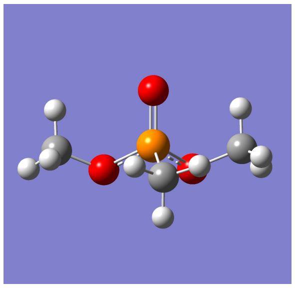

Center Number	Atomic Number	Atomic Type	Coordinates (Angstroms) X	Y	Z
1	15	0	0.020022	0.055108	0.139058

2	8	0	0.008123	-0.219854	1.599558
3	6	0	0.057822	1.807126	-0.272632
4	1	0	0.066737	1.936280	-1.356166
5	1	0	-0.821103	2.297966	0.151400
6	1	0	0.949919	2.263381	0.162226
7	8	0	1.218039	-0.626005	-0.682699
8	8	0	-1.199115	-0.574201	-0.692804
9	6	0	2.535916	-0.587848	-0.114803
10	1	0	2.527863	-1.013084	0.890537
11	1	0	3.167831	-1.185048	-0.769599
12	1	0	2.911439	0.439225	-0.083148
13	6	0	-2.517868	-0.485179	-0.132736
14	1	0	-3.170791	-1.046148	-0.798849
15	1	0	-2.535072	-0.922469	0.867295
16	1	0	-2.848212	0.556938	-0.090577

$(MeO)_2P(O)Me$ isomer C M06-2X/6-31+G(d,p), chloroform IEFPCM:
Sum of electronic and thermal Free Energies= -686.564411

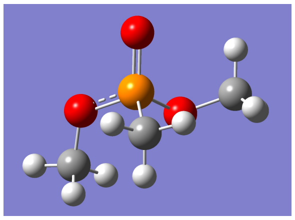

Center Number	Atomic Number	Atomic Type	Coordinates (Angstroms) X	Y	Z
1	15	0	0.035686	0.408138	-0.221867
2	8	0	0.688014	1.234902	-1.264768
3	6	0	-0.111001	1.213791	1.381376
4	1	0	-0.577788	0.551710	2.113278
5	1	0	0.885522	1.489691	1.733448
6	1	0	-0.707244	2.121329	1.268190
7	8	0	-1.431639	-0.065529	-0.647577
8	8	0	0.745966	-1.010144	0.066985
9	6	0	-2.173718	-1.013173	0.133067
10	1	0	-2.434849	-0.586726	1.105503
11	1	0	-3.084110	-1.222154	-0.425912
12	1	0	-1.596479	-1.930341	0.264762
13	6	0	2.174881	-1.055786	0.188723
14	1	0	2.442138	-2.106374	0.288887
15	1	0	2.643180	-0.632256	-0.701840
16	1	0	2.502091	-0.509892	1.078690

Me_3PO M06-2X/6-31+G(d,p), chloroform IEFPCM:
Sum of electronic and thermal Free Energies= -536.149640

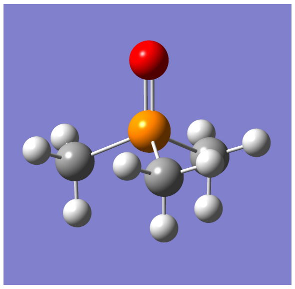

Center Number	Atomic Number	Atomic Type	Coordinates (Angstroms) X	Y	Z
1	15	0	-1.227003	1.330907	0.081187
2	6	0	-0.507924	2.150032	1.532849
3	1	0	-0.726175	3.219562	1.484683
4	1	0	-0.963134	1.737791	2.436536
5	1	0	0.574307	2.001611	1.570852
6	6	0	-0.327371	2.005899	-1.344067
7	1	0	-0.667036	1.502553	-2.252440
8	1	0	-0.543576	3.073411	-1.430473
9	1	0	0.750209	1.861473	-1.230300
10	6	0	-0.701796	-0.402727	0.200421
11	1	0	-1.151157	-0.852353	1.089129
12	1	0	-1.050532	-0.943072	-0.682918
13	1	0	0.386633	-0.478111	0.266156
14	8	0	-2.723346	1.498039	-0.019754

$EtOP(O)Me_2$ isomer A M06-2X/6-31+G(d,p), chloroform IEFPCM:
Sum of electronic and thermal Free Energies= -650.632536

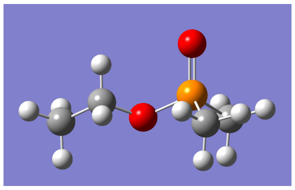

Center Number	Atomic Number	Atomic Type	Coordinates (Angstroms) X	Y	Z
1	15	0	-0.818888	-0.024301	0.159320
2	8	0	-0.975605	0.240264	1.624239
3	8	0	0.691116	-0.412862	-0.292103
4	6	0	1.781313	0.413393	0.158433
5	1	0	1.689529	0.564629	1.238280
6	1	0	1.718702	1.388254	-0.339957

7	6	0	3.074622	-0.289519	-0.192345
8	1	0	3.146886	-0.445505	-1.271551
9	1	0	3.925155	0.316493	0.130430
10	1	0	3.127500	-1.259695	0.307402
11	6	0	-1.719734	-1.453419	-0.463963
12	1	0	-1.404048	-2.337006	0.094574
13	1	0	-2.789214	-1.294077	-0.310410
14	1	0	-1.517845	-1.600707	-1.526849
15	6	0	-1.268930	1.398893	-0.863743
16	1	0	-1.080698	1.191242	-1.919408
17	1	0	-2.328032	1.622473	-0.716316
18	1	0	-0.685356	2.267971	-0.550909

EtOP(O)Me_2 isomer B M06-2X/6-31+G(d,p), chloroform IEFPCM:
Sum of electronic and thermal Free Energies= -650.628185

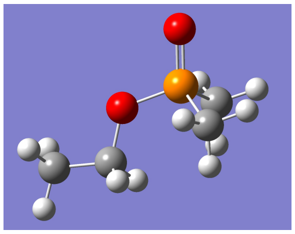

Center Number	Atomic Number	Atomic Type	Coordinates (Angstroms) X	Y	Z
1	15	0	-0.824624	0.010374	0.094704
2	8	0	-0.913934	0.273156	1.561328
3	8	0	0.720906	-0.219800	-0.340051
4	6	0	1.130714	-0.313530	-1.712693
5	1	0	0.994558	0.660923	-2.194792
6	1	0	0.510665	-1.051662	-2.235741
7	6	0	2.586494	-0.727245	-1.736927
8	1	0	2.714547	-1.700822	-1.258306
9	1	0	2.936091	-0.794779	-2.770370
10	1	0	3.197712	0.007241	-1.207461
11	6	0	-1.704226	-1.480553	-0.426803
12	1	0	-1.290432	-2.334733	0.113887
13	1	0	-2.759891	-1.372134	-0.167416
14	1	0	-1.618958	-1.650917	-1.502577
15	6	0	-1.427756	1.369315	-0.934497
16	1	0	-1.394040	1.122152	-1.998323
17	1	0	-2.461205	1.582260	-0.651614
18	1	0	-0.818475	2.256141	-0.743834

Table S20. Coordinates of optimized structures for tricoordinate and tetracoordinate phosphorus compounds, PBE0 (pbe1pbe)/6-31+G(d,p), CHCl3 solvation.

PH_3 PBE0/6-31+G(d,p), chloroform IEFPCM:
Sum of electronic and thermal Free Energies= -342.984425

Center Number	Atomic Number	Atomic Type	Coordinates (Angstroms) X	Y	Z
1	15	0	0.000000	0.000000	0.126771
2	1	0	0.000000	1.199273	-0.633856
3	1	0	-1.038600	-0.599636	-0.633856
4	1	0	1.038600	-0.599636	-0.633856

$PMeH_2$ PBE0/6-31+G(d,p), chloroform IEFPCM:
Sum of electronic and thermal Free Energies= -382.231318

Center Number	Atomic Number	Atomic Type	Coordinates (Angstroms) X	Y	Z
1	15	0	0.663884	0.000005	-0.124089
2	1	0	0.942426	-1.038199	0.806421
3	1	0	0.942426	1.038130	0.806511
4	6	0	-1.188981	-0.000001	0.026184
5	1	0	-1.584035	0.882548	-0.484214
6	1	0	-1.584035	-0.882509	-0.484286
7	1	0	-1.541152	-0.000043	1.059793

PMe_2H PBE0/6-31+G(d,p), chloroform IEFPCM:
Sum of electronic and thermal Free Energies= -421.479761

Center Number	Atomic Number	Atomic Type	Coordinates (Angstroms) X	Y	Z
1	15	0	0.000000	-0.650838	-0.114070
2	1	0	0.000000	-1.099371	1.236603
3	6	0	1.426715	0.525189	0.022954
4	1	0	2.354032	-0.040205	0.153671
5	1	0	1.512097	1.092193	-0.908923
6	1	0	1.318123	1.227844	0.854752
7	6	0	-1.426715	0.525189	0.022954
8	1	0	-1.512097	1.092192	-0.908923
9	1	0	-2.354032	-0.040205	0.153671

10	1	0	-1.318123	1.227844	0.854752

PMe_3 PBE0/6-31+G(d,p), chloroform IEFPCM:
Sum of electronic and thermal Free Energies= -460.730319

Center Number	Atomic Number	Atomic Type	Coordinates (Angstroms) X	Y	Z
1	15	0	0.000018	0.000056	-0.593069
2	6	0	-0.991437	-1.297784	0.275204
3	1	0	-0.636518	-2.290259	-0.019312
4	1	0	-2.041830	-1.217557	-0.021337
5	6	0	-0.628332	1.507454	0.275335
6	1	0	-0.034407	2.377389	-0.021627
7	1	0	-0.582992	1.403771	1.366131
8	1	0	-0.923853	-1.207310	1.366034
9	1	0	-1.665593	1.695529	-0.018642
10	6	0	1.619734	-0.209639	0.275148
11	1	0	1.507608	-0.196696	1.366005
12	1	0	2.301508	0.594262	-0.019218
13	1	0	2.075798	-1.159163	-0.021724

PPh_3 PBE0/6-31+G(d,p), chloroform IEFPCM:
Sum of electronic and thermal Free Energies= -1035.147360

Center Number	Atomic Number	Atomic Type	Coordinates (Angstroms) X	Y	Z
1	15	0	0.001566	-0.002250	-1.224462
2	6	0	-0.603969	1.541632	-0.422304
3	6	0	-1.712094	2.171399	-1.007261
4	6	0	-0.014706	2.126816	0.704618
5	6	0	-2.233532	3.343784	-0.465335
6	1	0	-2.169672	1.740129	-1.895116
7	6	0	-0.528225	3.308476	1.239258
8	1	0	0.850260	1.659730	1.167429
9	6	0	-1.639975	3.917206	0.659389
10	1	0	-3.095848	3.815691	-0.928241
11	1	0	-0.058216	3.751787	2.113065
12	1	0	-2.038679	4.837215	1.077646
13	6	0	1.639740	-0.248702	-0.418744
14	6	0	1.850339	-1.053862	0.706835
15	6	0	2.739920	0.397911	-1.000421
16	6	0	3.129613	-1.200559	1.243318

17	1	0	1.012832	-1.570698	1.167231
18	6	0	4.014996	0.262950	-0.456428
19	1	0	2.596525	1.011595	-1.887269
20	6	0	4.213284	-0.540162	0.666908
21	1	0	3.277367	-1.831192	2.115928
22	1	0	4.855309	0.775388	-0.916710
23	1	0	5.208769	-0.655269	1.086542
24	6	0	-1.033470	-1.295639	-0.418688
25	6	0	-1.041187	-2.566836	-1.010710
26	6	0	-1.819933	-1.078146	0.718828
27	6	0	-1.797697	-3.601805	-0.465877
28	1	0	-0.450600	-2.746863	-1.906488
29	6	0	-2.588253	-2.110642	1.256604
30	1	0	-1.834679	-0.097906	1.187270
31	6	0	-2.576153	-3.374677	0.669195
32	1	0	-1.788321	-4.582019	-0.934545
33	1	0	-3.195619	-1.925569	2.138551
34	1	0	-3.175081	-4.177682	1.089672

Methoxyphospholane PBE0/6-31+G(d,p), chloroform IEFPCM:
Sum of electronic and thermal Free Energies= -685.007833

Center Number	Atomic Number	Atomic Type	Coordinates (Angstroms) X	Y	Z
1	8	0	-0.567992	1.069540	-0.676046
2	6	0	-1.487968	1.024665	0.425817
3	6	0	-1.965137	-0.420153	0.474310
4	1	0	-0.968449	1.311123	1.346678
5	1	0	-2.295087	1.731018	0.224010
6	1	0	-2.243558	-0.733694	1.483109
7	1	0	-2.803154	-0.592615	-0.209076
8	15	0	0.317316	-0.332985	-0.717681
9	8	0	-0.855830	-1.221249	0.036389
10	6	0	2.668656	0.225527	0.342138
11	1	0	3.285936	-0.191771	1.139654
12	1	0	3.031386	-0.147236	-0.623401
13	1	0	2.746348	1.317151	0.357424
14	8	0	1.323014	-0.183220	0.588810

$P(OMe)_3$ isomer A PBE0/6-31+G(d,p), chloroform IEFPCM:
Sum of electronic and thermal Free Energies= -686.181104

Center Number	Atomic Number	Atomic Type	Coordinates (Angstroms) X	Y	Z
1	15	0	-0.075904	-0.071365	-0.713398
2	6	0	1.567573	-1.941861	0.167577
3	1	0	1.557303	-2.740982	0.911092
4	1	0	1.424988	-2.378052	-0.828539
5	1	0	2.536744	-1.432881	0.199574
6	6	0	1.054780	1.960447	0.633154
7	1	0	1.835858	2.699297	0.448175
8	1	0	0.138342	2.461339	0.954154
9	1	0	1.382835	1.262096	1.408165
10	6	0	-2.483141	-0.425457	0.284168
11	1	0	-3.326186	0.162353	0.650738
12	1	0	-2.761686	-0.893347	-0.668204
13	1	0	-2.237575	-1.204206	1.012923
14	8	0	0.839222	1.275485	-0.607143
15	8	0	0.513216	-1.040884	0.500362
16	8	0	-1.389443	0.474512	0.120554

$P(OMe)_3$ isomer B PBE0/6-31+G(d,p), chloroform IEFPCM:
Sum of electronic and thermal Free Energies= -686.179161

Center Number	Atomic Number	Atomic Type	Coordinates (Angstroms) X	Y	Z
1	6	0	2.289116	-0.714612	0.422593
2	8	0	0.871043	-0.854219	0.387389
3	15	0	0.084460	-0.208820	-0.927704
4	8	0	-1.411303	-0.748399	-0.550216
5	6	0	-1.960727	-0.906603	0.761819
6	8	0	0.104104	1.396717	-0.562144
7	1	0	2.655548	-1.375198	1.210217
8	1	0	2.743611	-1.008705	-0.531000
9	1	0	2.577327	0.316922	0.653674
10	1	0	-2.379057	0.040357	1.114790
11	1	0	-2.762315	-1.642717	0.678313
12	1	0	-1.204238	-1.265872	1.462747
13	6	0	-0.175300	1.949429	0.725582
14	1	0	-1.246856	2.147867	0.823108
15	1	0	0.370508	2.892375	0.798864

16	1	0	0.151279	1.278557	1.525924

P(OMe)$_3$ isomer C PBE0/6-31+G(d,p), chloroform IEFPCM:
Sum of electronic and thermal Free Energies= -686.178948

Center Number	Atomic Number	Atomic Type	Coordinates (Angstroms) X	Y	Z
1	15	0	0.000901	0.000160	0.412068
2	6	0	-0.889965	2.451254	0.072407
3	1	0	-1.766149	2.949554	-0.345438
4	1	0	-0.941241	2.493583	1.167142
5	1	0	0.014866	2.964554	-0.267850
6	6	0	2.568560	-0.456520	0.072322
7	1	0	3.438715	0.051211	-0.346759
8	1	0	2.631891	-0.432407	1.166982
9	1	0	2.558510	-1.497056	-0.266970
10	6	0	-1.679228	-1.994287	0.072491
11	1	0	-1.677429	-3.000585	-0.349307
12	1	0	-1.689217	-2.064149	1.166961
13	1	0	-2.574379	-1.462286	-0.264642
14	8	0	1.413190	0.235842	-0.400018
15	8	0	-0.909910	1.104662	-0.400364
16	8	0	-0.500763	-1.341573	-0.399067

PCl$_3$ experimental geometry PBE0/6-31+G(d,p), chloroform IEFPCM:
Sum of electronic and thermal Free Energies= -1721.389452
(not a minimum, but 0 negative frequencies)

Center Number	Atomic Number	Atomic Type	Coordinates (Angstroms) X	Y	Z
1	15	0	0.000000	0.000000	0.727649
2	17	0	-0.024723	1.802585	-0.233562
3	17	0	-1.562204	-0.926353	-0.207892
4	17	0	1.569915	-0.893466	-0.226780

PCl_3 PBE0/6-31+G(d,p), chloroform IEFPCM:
Sum of electronic and thermal Free Energies= -1721.390683

Center Number	Atomic Number	Atomic Type	Coordinates (Angstroms) X	Y	Z
1	15	0	0.000000	0.000000	0.731656
2	17	0	0.000000	1.840361	-0.215193
3	17	0	-1.593800	-0.920181	-0.215193
4	17	0	1.593800	-0.920181	-0.215193

H_3PO_4 PBE0/6-31+G(d,p), water IEFPCM:
Sum of electronic and thermal Free Energies= -643.715865

Center Number	Atomic Number	Atomic Type	Coordinates (Angstroms) X	Y	Z
1	15	0	-1.277636	1.092876	-0.160954
2	8	0	-1.130315	1.840277	1.243601
3	1	0	-0.274145	2.275361	1.358177
4	8	0	-0.710847	-0.361782	0.178691
5	1	0	-0.545061	-0.901939	-0.606277
6	8	0	-2.851347	0.824688	-0.230724
7	1	0	-3.348829	1.556154	-0.621921
8	8	0	-0.676185	1.774975	-1.331310

PH_4^+ BF_4^- PBE0/6-31+G(d,p), chloroform IEFPCM:
Sum of electronic and thermal Free Energies= -767.621602

Center Number	Atomic Number	Atomic Type	Coordinates (Angstroms) X	Y	Z
1	15	0	-1.371517	0.992507	0.117753
2	1	0	-0.511588	-0.114592	0.099611
3	1	0	-1.119200	1.802802	1.223795
4	1	0	-1.253126	1.737010	-1.054696
5	1	0	-2.702161	0.566855	0.208668
6	5	0	2.099431	0.720164	-0.121325
7	9	0	1.082702	1.734772	-0.060155
8	9	0	2.970237	0.885855	0.948234
9	9	0	1.435041	-0.530096	-0.020693
10	9	0	2.758464	0.812784	-1.341193

$P(OPh)_4^+ PF_6^-$ PBE0/6-31+G(d,p), chloroform IEFPCM:
Sum of electronic and thermal Free Energies= -2506.997461

Center Number	Atomic Number	Atomic Type	Coordinates (Angstroms) X	Y	Z
1	15	0	-1.544590	0.005142	0.048482
2	8	0	-2.408630	0.651082	1.177548
3	8	0	-0.702391	1.160125	-0.567316
4	8	0	-0.743000	-1.153907	0.711510
5	8	0	-2.348845	-0.638436	-1.124694
6	6	0	-3.042414	0.049999	-2.151870
7	6	0	-4.427444	0.020979	-2.114598
8	6	0	-2.323539	0.639390	-3.180055
9	6	0	-5.124310	0.632544	-3.155366
10	1	0	-4.943287	-0.478757	-1.301382
11	6	0	-3.039226	1.248297	-4.209742
12	1	0	-1.239151	0.621059	-3.179497
13	6	0	-4.433308	1.248023	-4.198069
14	1	0	-6.209760	0.620966	-3.149157
15	1	0	-2.498514	1.718170	-5.025251
16	1	0	-4.981730	1.721767	-5.006213
17	6	0	-3.239182	-0.035556	2.098600
18	6	0	-4.607499	0.141055	1.966252
19	6	0	-2.666063	-0.770994	3.124525
20	6	0	-5.442049	-0.468578	2.901295
21	1	0	-5.005085	0.749641	1.160738
22	6	0	-3.518333	-1.375609	4.047427
23	1	0	-1.588861	-0.866856	3.203568
24	6	0	-4.900129	-1.228468	3.936911
25	1	0	-6.517073	-0.342799	2.818369
26	1	0	-3.093100	-1.958090	4.858669
27	1	0	-5.554791	-1.700457	4.662786
28	6	0	-0.058518	-2.199877	0.028362
29	6	0	1.318815	-2.113499	-0.072091
30	6	0	-0.793038	-3.282868	-0.428915
31	6	0	1.993155	-3.170055	-0.684078
32	1	0	1.861320	-1.259779	0.319709
33	6	0	-0.099819	-4.328266	-1.036161
34	1	0	-1.871170	-3.309057	-0.313361
35	6	0	1.287732	-4.271447	-1.165643
36	1	0	3.073007	-3.114373	-0.775039
37	1	0	-0.650411	-5.188293	-1.404759
38	1	0	1.820632	-5.089927	-1.639767
39	6	0	-0.033036	2.193111	0.149760
40	6	0	-0.776246	3.263547	0.622313

41	6	0	1.343319	2.108224	0.264325
42	6	0	-0.093486	4.295303	1.263929
43	1	0	-1.852555	3.291504	0.491565
44	6	0	2.006869	3.151988	0.908949
45	1	0	1.892919	1.267772	-0.146037
46	6	0	1.292438	4.239007	1.409571
47	1	0	-0.650993	5.145250	1.645297
48	1	0	3.086304	3.100216	1.006541
49	1	0	1.817090	5.047653	1.909086
50	15	0	4.960583	-0.003827	-0.055508
51	9	0	4.934360	1.612256	0.194249
52	9	0	6.168886	-0.158238	1.027048
53	9	0	3.856051	-0.200679	1.143409
54	9	0	4.968830	-1.620489	-0.304358
55	9	0	6.043539	0.192928	-1.257491
56	9	0	3.731935	0.150058	-1.134296

O=P(OCH2)3P=O PBE0/6-31+G(d,p), chloroform IEFPCM:
Sum of electronic and thermal Free Energies= -1175.973979

Center Number	Atomic Number	Atomic Type	Coordinates (Angstroms) X	Y	Z
1	6	0	-1.040087	0.472656	-1.344815
2	1	0	-1.414798	1.039919	-2.200035
3	6	0	-1.329676	-1.631384	0.506625
4	1	0	-0.620147	-2.351191	0.092054
5	6	0	-3.412379	0.228913	0.149705
6	1	0	-4.262106	-0.367838	0.488766
7	15	0	-1.119239	0.876689	1.247066
8	8	0	-0.544178	1.722163	2.297571
9	8	0	-0.564896	-0.637009	1.221138
10	8	0	-2.719522	0.697180	1.326478
11	8	0	-0.866316	1.403817	-0.255357
12	1	0	-3.776925	1.082345	-0.430296
13	1	0	-0.077728	0.020074	-1.603184
14	1	0	-2.006788	-2.144940	1.196277
15	15	0	-2.269088	-0.814381	-0.855963
16	8	0	-2.855451	-1.676569	-1.928036

$P(OMe)_4^+ BF_4^-$ isomer A PBE0/6-31+G(d,p), chloroform IEFPCM:
Sum of electronic and thermal Free Energies= -1225.235861

Center Number	Atomic Number	Atomic Type	Coordinates (Angstroms) X	Y	Z
1	15	0	-0.265199	0.650960	-0.466805
2	8	0	0.298487	1.754385	0.460736
3	8	0	0.065447	-0.717632	0.217327
4	8	0	-1.808058	0.638960	-0.681135
5	8	0	0.402001	0.831759	-1.860765
6	6	0	-0.114987	1.986938	1.833629
7	1	0	0.294266	1.196677	2.463743
8	1	0	0.301656	2.958991	2.085193
9	1	0	-1.202832	2.023158	1.890499
10	6	0	1.688605	1.478410	-2.080726
11	1	0	2.467041	0.944186	-1.534877
12	1	0	1.857269	1.406392	-3.153021
13	1	0	1.610414	2.518555	-1.766963
14	6	0	-0.398638	-1.990312	-0.298472
15	1	0	-0.024697	-2.737143	0.398718
16	1	0	-1.488852	-2.000920	-0.322955
17	1	0	0.017090	-2.159441	-1.293154
18	6	0	-2.514176	1.619796	-1.502005
19	1	0	-2.153777	1.546313	-2.528110
20	1	0	-3.560980	1.330608	-1.438527
21	1	0	-2.351352	2.617566	-1.094702
22	9	0	0.552749	4.922708	0.435334
23	9	0	-1.637440	4.273098	0.166042
24	9	0	-0.161618	4.072508	-1.582746
25	9	0	-0.855901	6.156254	-0.900402
26	5	0	-0.520610	4.866788	-0.469942

$P(OMe)_4^+ BF_4^-$ isomer B PBE0/6-31+G(d,p), chloroform IEFPCM:
Sum of electronic and thermal Free Energies= -1225.229980

Center Number	Atomic Number	Atomic Type	Coordinates (Angstroms) X	Y	Z
1	15	0	-0.616851	0.377553	-0.056976
2	8	0	-0.098288	0.956562	1.289674
3	8	0	0.055413	-1.036815	-0.044031
4	8	0	-2.176373	0.314773	-0.146271
5	8	0	-0.276596	1.200226	-1.329432
6	6	0	-0.472126	2.278141	1.795212

7	1	0	-1.558687	2.360919	1.842674
8	1	0	-0.043059	2.318795	2.793799
9	1	0	-0.046130	3.050058	1.155133
10	6	0	1.084607	1.586516	-1.696374
11	1	0	1.769739	0.754067	-1.526746
12	1	0	1.028454	1.825416	-2.755719
13	1	0	1.362936	2.468985	-1.120988
14	6	0	-0.072939	-1.954084	-1.162094
15	1	0	0.536036	-2.814795	-0.894959
16	1	0	-1.115991	-2.249766	-1.284480
17	1	0	0.305093	-1.489953	-2.074263
18	6	0	-2.978190	-0.384644	0.840903
19	1	0	-2.727880	-0.042641	1.846358
20	1	0	-4.008425	-0.130459	0.602019
21	1	0	-2.820320	-1.460029	0.748848
22	9	0	0.794237	6.545554	-0.485866
23	9	0	-0.888209	5.034633	-0.055649
24	9	0	1.300791	4.346506	-0.029212
25	9	0	0.299226	4.904121	-2.021458
26	5	0	0.371789	5.223365	-0.655796

(*i*-PrO)$_2$P(O)H isomer A PBE0/6-31+G(d,p), chloroform IEFPCM:
Sum of electronic and thermal Free Energies= -803.955953

Center Number	Atomic Number	Atomic Type	Coordinates (Angstroms) X	Y	Z
1	15	0	-0.168540	0.254983	-0.992962
2	1	0	0.068588	0.205238	-2.379567
3	8	0	0.632834	1.288515	-0.292212
4	8	0	0.102913	-1.223465	-0.464448
5	8	0	-1.766738	0.374399	-0.886432
6	6	0	-2.424101	1.605401	-1.306542
7	1	0	-1.769107	2.122177	-2.022216
8	6	0	-0.534682	-2.382402	-1.077167
9	1	0	-0.987805	-2.067722	-2.027033
10	6	0	0.556452	-3.397329	-1.345731
11	1	0	1.038101	-3.694260	-0.408825
12	1	0	0.129122	-4.289732	-1.813168
13	1	0	1.317965	-2.986811	-2.014388
14	6	0	-1.613965	-2.885167	-0.140819
15	1	0	-1.176412	-3.176530	0.819255
16	1	0	-2.365537	-2.111635	0.034386
17	1	0	-2.106949	-3.759992	-0.576354
18	6	0	-3.706943	1.199722	-2.000490

19	1	0	-4.242247	2.089459	-2.345476
20	1	0	-3.500969	0.562858	-2.865252
21	1	0	-4.356419	0.652766	-1.309476
22	6	0	-2.642989	2.484270	-0.091850
23	1	0	-3.281106	1.972405	0.635651
24	1	0	-1.690598	2.728576	0.385057
25	1	0	-3.134606	3.415943	-0.389324

(*i*-PrO)$_2$P(O)H isomer B PBE0/6-31+G(d,p), chloroform IEFPCM:
Sum of electronic and thermal Free Energies= -803.957148

Center Number	Atomic Number	Atomic Type	Coordinates (Angstroms) X	Y	Z
1	15	0	-0.665546	0.048431	-2.532426
2	1	0	-1.496916	-0.638817	-3.424537
3	8	0	-0.102836	1.321640	-3.059188
4	8	0	0.343677	-1.134607	-2.176271
5	8	0	-1.505081	0.213157	-1.171379
6	6	0	-2.205163	1.448661	-0.850729
7	1	0	-1.684975	2.265030	-1.363745
8	6	0	1.425347	-0.933633	-1.217705
9	1	0	1.286229	0.042717	-0.737748
10	6	0	1.313156	-2.031854	-0.181569
11	1	0	1.412160	-3.013733	-0.655559
12	1	0	2.108696	-1.926040	0.562500
13	1	0	0.348907	-1.985506	0.331223
14	6	0	2.734148	-0.948249	-1.979646
15	1	0	2.867196	-1.908319	-2.488552
16	1	0	2.760030	-0.148769	-2.724519
17	1	0	3.570245	-0.804969	-1.287957
18	6	0	-3.636619	1.349852	-1.342230
19	1	0	-4.182051	2.264207	-1.088545
20	1	0	-3.678717	1.224731	-2.428596
21	1	0	-4.145308	0.501831	-0.872508
22	6	0	-2.099946	1.630206	0.648595
23	1	0	-2.582434	0.798592	1.172453
24	1	0	-1.053505	1.681967	0.961537
25	1	0	-2.595438	2.559462	0.945920

(i-PrO)$_2$P(O)H isomer C PBE0/6-31+G(d,p), chloroform IEFPCM:
Sum of electronic and thermal Free Energies= -803.958154

Center Number	Atomic Number	Atomic Type	Coordinates (Angstroms) X	Y	Z
1	15	0	-0.869659	-0.923566	-1.408791
2	1	0	-1.243748	-2.229169	-1.069654
3	8	0	-0.525084	-0.724306	-2.842855
4	8	0	0.301603	-0.514373	-0.386410
5	8	0	-2.131940	-0.146089	-0.819249
6	6	0	-2.223986	1.307574	-0.906993
7	1	0	-1.225974	1.703625	-1.131375
8	6	0	1.704253	-0.581613	-0.769920
9	1	0	1.757306	-0.415981	-1.851484
10	6	0	2.406254	0.543205	-0.039321
11	1	0	2.327019	0.408006	1.044271
12	1	0	3.466671	0.556564	-0.308963
13	1	0	1.970689	1.510414	-0.304680
14	6	0	2.250213	-1.953629	-0.423884
15	1	0	2.154023	-2.144924	0.649754
16	1	0	1.721221	-2.742124	-0.968209
17	1	0	3.309521	-2.013001	-0.692610
18	6	0	-3.182031	1.659008	-2.026709
19	1	0	-3.271586	2.746490	-2.113059
20	1	0	-2.826276	1.263676	-2.981602
21	1	0	-4.174834	1.245710	-1.821694
22	6	0	-2.672151	1.801410	0.452098
23	1	0	-3.650449	1.380798	0.705954
24	1	0	-1.955551	1.514992	1.226364
25	1	0	-2.757221	2.892411	0.442962

(i-PrO)$_2$P(O)H isomer D PBE0/6-31+G(d,p), chloroform IEFPCM:
Sum of electronic and thermal Free Energies= -803.955049

Center Number	Atomic Number	Atomic Type	Coordinates (Angstroms) X	Y	Z
1	15	0	-0.255907	-0.419692	-1.759249
2	1	0	-0.040139	-1.537033	-2.587659
3	8	0	0.055642	0.873508	-2.416605
4	8	0	0.562365	-0.774458	-0.423407
5	8	0	-1.770273	-0.537370	-1.277816
6	6	0	-2.289631	-1.762829	-0.683433
7	1	0	-1.562557	-2.567846	-0.856685

8	6	0	2.019278	-0.784696	-0.456481
9	1	0	2.342554	-0.923010	-1.497703
10	6	0	2.528377	0.545279	0.060756
11	1	0	2.197255	0.701814	1.092395
12	1	0	3.622652	0.559710	0.041559
13	1	0	2.157429	1.366944	-0.556789
14	6	0	2.467538	-1.969814	0.372106
15	1	0	2.124523	-1.862161	1.406114
16	1	0	2.070266	-2.904656	-0.032992
17	1	0	3.560003	-2.031004	0.375905
18	6	0	-2.456790	-1.538909	0.805340
19	1	0	-2.843176	-2.447497	1.277841
20	1	0	-1.500240	-1.284244	1.267933
21	1	0	-3.165539	-0.725100	0.988451
22	6	0	-3.585321	-2.086084	-1.397188
23	1	0	-4.305705	-1.273029	-1.263426
24	1	0	-3.417855	-2.230948	-2.467947
25	1	0	-4.018623	-3.003219	-0.986565

$(MeO)_2P(O)H$ isomer A PBE0/6-31+G(d,p), chloroform IEFPCM:
Sum of electronic and thermal Free Energies= -646.967743

Center Number	Atomic Number	Atomic Type	Coordinates (Angstroms) X	Y	Z
1	15	0	0.007116	0.503630	0.389842
2	8	0	-1.432558	-0.160126	0.585321
3	8	0	0.949123	-0.791601	0.231048
4	8	0	0.168994	1.520191	-0.682180
5	6	0	-2.102662	-0.770487	-0.529955
6	1	0	-3.079786	-1.081654	-0.162106
7	1	0	-2.225001	-0.050962	-1.343131
8	1	0	-1.543203	-1.642748	-0.877276
9	6	0	2.284455	-0.647236	-0.274274
10	1	0	2.643283	-1.655160	-0.482325
11	1	0	2.287085	-0.052330	-1.190150
12	1	0	2.927522	-0.178928	0.477102
13	1	0	0.216767	0.973012	1.691856

$(MeO)_2P(O)H$ isomer B PBE0/6-31+G(d,p), chloroform IEFPCM:
Sum of electronic and thermal Free Energies= -646.966285

Center Number	Atomic Number	Atomic Type	Coordinates (Angstroms) X	Y	Z

Center Number	Atomic Number	Atomic Type	X	Y	Z
1	15	0	-0.023899	-0.515188	0.229837
2	8	0	1.460046	-0.282318	-0.309045
3	8	0	-0.756888	0.835557	-0.240949
4	8	0	-0.625052	-1.792572	-0.219731
5	6	0	2.187128	0.906581	0.034504
6	1	0	3.199660	0.762864	-0.341253
7	1	0	1.731089	1.778512	-0.439374
8	1	0	2.219235	1.044083	1.120193
9	6	0	-2.164975	0.988305	-0.010077
10	1	0	-2.436821	1.964172	-0.411486
11	1	0	-2.723629	0.204622	-0.526990
12	1	0	-2.386969	0.960929	1.061605
13	1	0	0.065075	-0.382949	1.628242

$(MeO)_2P(O)H$ isomer C PBE0/6-31+G(d,p), chloroform IEFPCM:
Sum of electronic and thermal Free Energies= -646.967203

Center Number	Atomic Number	Atomic Type	Coordinates (Angstroms) X	Y	Z
1	15	0	-0.000021	0.201026	0.272521
2	8	0	-1.203798	-0.764173	-0.152968
3	8	0	1.203710	-0.764173	-0.153100
4	8	0	-0.000056	1.577540	-0.285647
5	6	0	-2.546526	-0.259971	-0.090095
6	1	0	-3.185844	-1.055314	-0.471863
7	1	0	-2.826788	-0.032383	0.943229
8	1	0	-2.651445	0.632364	-0.711457
9	6	0	2.546454	-0.260014	-0.090209
10	1	0	3.185749	-1.055367	-0.471992
11	1	0	2.651402	0.632332	-0.711551
12	1	0	2.826720	-0.032457	0.943121
13	1	0	0.000063	0.190458	1.678820

$Ph_4P^+ Cl^-$ PBE0/6-31+G(d,p), chloroform IEFPCM:
Sum of electronic and thermal Free Energies= -1726.501836

Center Number	Atomic Number	Atomic Type	Coordinates (Angstroms) X	Y	Z
1	15	0	0.411491	0.007029	-0.021484
2	6	0	-0.124627	-1.506239	-0.859035
3	6	0	-1.457908	-1.634431	-1.272176

4	6	0	0.770460	-2.572783	-1.023520
5	6	0	-1.877964	-2.821320	-1.865649
6	1	0	-2.195132	-0.850440	-1.109070
7	6	0	0.336413	-3.753164	-1.620580
8	1	0	1.799192	-2.491471	-0.687829
9	6	0	-0.984781	-3.875829	-2.047031
10	1	0	-2.913800	-2.916411	-2.175869
11	1	0	1.033228	-4.575977	-1.748542
12	1	0	-1.320777	-4.797758	-2.512749
13	6	0	-0.193394	1.502242	-0.841653
14	6	0	-1.564529	1.793027	-0.798587
15	6	0	0.691568	2.365987	-1.500548
16	6	0	-2.040000	2.935520	-1.435442
17	1	0	-2.284365	1.150907	-0.288621
18	6	0	0.201720	3.508391	-2.127272
19	1	0	1.756875	2.162365	-1.520198
20	6	0	-1.162762	3.791745	-2.098779
21	1	0	-3.104426	3.147016	-1.405081
22	1	0	0.890339	4.177334	-2.634486
23	1	0	-1.541434	4.683236	-2.590361
24	6	0	2.220673	0.034002	0.001557
25	6	0	2.908141	0.218751	1.205552
26	6	0	2.933635	-0.112167	-1.198413
27	6	0	4.300961	0.260279	1.207697
28	1	0	2.362948	0.325503	2.138255
29	6	0	4.323606	-0.068198	-1.186313
30	1	0	2.410189	-0.267072	-2.138084
31	6	0	5.007200	0.118666	0.015697
32	1	0	4.831383	0.401076	2.144344
33	1	0	4.872137	-0.182633	-2.116125
34	1	0	6.092656	0.150148	0.021405
35	6	0	-0.211471	-0.007576	1.672214
36	6	0	-0.349261	1.197227	2.373816
37	6	0	-0.519231	-1.225022	2.289850
38	6	0	-0.789735	1.177152	3.693127
39	1	0	-0.128024	2.145388	1.892380
40	6	0	-0.959990	-1.233543	3.609839
41	1	0	-0.431783	-2.158430	1.741944
42	6	0	-1.094836	-0.035864	4.309689
43	1	0	-0.905955	2.110796	4.234589
44	1	0	-1.211449	-2.176219	4.085596
45	1	0	-1.449563	-0.046172	5.336000
46	17	0	-4.507277	-0.023656	-0.132698

$Ph_4P^+ Br^-$ PBE0/6-31+G(d,p), chloroform IEFPCM:
Sum of electronic and thermal Free Energies= -3837.870214

Center Number	Atomic Number	Atomic Type	Coordinates (Angstroms) X	Y	Z
1	15	0	0.772838	0.000766	-0.017527
2	6	0	0.387643	-1.466792	-1.010058
3	6	0	-0.952318	-1.710054	-1.344117
4	6	0	1.390670	-2.346934	-1.435201
5	6	0	-1.271710	-2.814702	-2.127381
6	1	0	-1.762301	-1.077847	-0.973964
7	6	0	1.055810	-3.448913	-2.218307
8	1	0	2.426447	-2.191140	-1.154126
9	6	0	-0.272266	-3.679037	-2.571754
10	1	0	-2.311918	-2.999066	-2.378587
11	1	0	1.836177	-4.129467	-2.545225
12	1	0	-0.529319	-4.539156	-3.183319
13	6	0	0.403824	1.527977	-0.922280
14	6	0	-0.928771	1.787115	-1.273764
15	6	0	1.412024	2.438936	-1.261436
16	6	0	-1.234901	2.939996	-1.989854
17	1	0	-1.743465	1.128909	-0.965783
18	6	0	1.090628	3.588602	-1.978993
19	1	0	2.440968	2.269002	-0.963871
20	6	0	-0.229220	3.835273	-2.351428
21	1	0	-2.269395	3.136707	-2.254766
22	1	0	1.875014	4.292449	-2.240435
23	1	0	-0.475606	4.732213	-2.912402
24	6	0	2.527239	-0.020827	0.426954
25	6	0	2.897567	-0.069627	1.775443
26	6	0	3.517072	0.010201	-0.568338
27	6	0	4.246136	-0.087312	2.124700
28	1	0	2.138780	-0.093507	2.551423
29	6	0	4.860835	-0.008125	-0.209906
30	1	0	3.246910	0.049065	-1.620249
31	6	0	5.226018	-0.056720	1.135693
32	1	0	4.527156	-0.124894	3.172616
33	1	0	5.622388	0.015828	-0.983246
34	1	0	6.276252	-0.070614	1.411431
35	6	0	-0.227646	-0.039185	1.479900
36	6	0	-0.603541	1.158677	2.097871
37	6	0	-0.598464	-1.269290	2.034586
38	6	0	-1.348205	1.120820	3.271902
39	1	0	-0.334315	2.114752	1.658896
40	6	0	-1.342467	-1.296526	3.209379

41	1	0	-0.325225	-2.199987	1.546175
42	6	0	-1.719144	-0.103786	3.824704
43	1	0	-1.659374	2.049469	3.739988
44	1	0	-1.649293	-2.249685	3.628462
45	1	0	-2.317544	-0.128543	4.730602
46	35	0	-3.834937	0.016725	-0.031412

Me_4P^+ Cl^- PBE0/6-31+G(d,p), chloroform IEFPCM:
Sum of electronic and thermal Free Energies= -960.641567

Center Number	Atomic Number	Atomic Type	Coordinates (Angstroms) X	Y	Z
1	15	0	1.501436	-0.001058	0.000259
2	6	0	3.308896	0.001982	0.005650
3	1	0	3.684078	-0.978675	-0.297800
4	1	0	3.678576	0.758458	-0.691112
5	1	0	3.675300	0.230708	1.009783
6	6	0	0.899551	1.613294	0.518945
7	1	0	1.258897	2.380701	-0.171176
8	1	0	-0.195979	1.570113	0.499644
9	1	0	1.250529	1.836412	1.529594
10	6	0	0.896997	-1.263732	1.130243
11	1	0	-0.198406	-1.217098	1.106719
12	1	0	1.238470	-2.249087	0.802772
13	1	0	1.262597	-1.065505	2.140666
14	6	0	0.902379	-0.352060	-1.660063
15	1	0	1.259066	-1.332766	-1.985069
16	1	0	-0.193246	-0.344876	-1.614302
17	1	0	1.255555	0.415696	-2.353417
18	17	0	-2.269352	-0.002719	0.028387

Me_4P^+ Br^- PBE0/6-31+G(d,p), chloroform IEFPCM:
Sum of electronic and thermal Free Energies= -3072.003656

Center Number	Atomic Number	Atomic Type	Coordinates (Angstroms) X	Y	Z
1	15	0	1.504548	-0.000529	0.001249
2	6	0	3.312033	0.002116	0.005007
3	1	0	3.686261	-0.977152	-0.304148
4	1	0	3.681301	0.761933	-0.688419
5	1	0	3.679597	0.225584	1.009936
6	6	0	0.904136	1.614788	0.520887

7	1	0	1.258972	2.380656	-0.173267
8	1	0	-0.191859	1.569343	0.506929
9	1	0	1.261042	1.839477	1.529121
10	6	0	0.901482	-1.264584	1.131949
11	1	0	-0.194444	-1.219933	1.105482
12	1	0	1.245901	-2.249145	0.804913
13	1	0	1.265158	-1.064778	2.142788
14	6	0	0.905348	-0.353405	-1.659482
15	1	0	1.264574	-1.333561	-1.983307
16	1	0	-0.190707	-0.348390	-1.610945
17	1	0	1.255973	0.415226	-2.353228
18	35	0	-2.333967	0.002141	0.014255

(*i*-PrO)$_2$P(O)Me isomer A PBE0/6-31+G(d,p), chloroform IEFPCM:
Sum of electronic and thermal Free Energies= -843.214641

Center Number	Atomic Number	Atomic Type	Coordinates (Angstroms) X	Y	Z
1	15	0	-0.139302	0.254116	-1.202430
2	8	0	0.631061	1.309602	-0.491290
3	8	0	0.119585	-1.198281	-0.581001
4	8	0	-1.745475	0.373425	-1.097795
5	6	0	-2.399182	1.657841	-1.289109
6	1	0	-1.668924	2.364017	-1.705263
7	6	0	-0.634620	-2.382227	-0.958657
8	1	0	-1.319510	-2.117821	-1.773648
9	6	0	0.357844	-3.423590	-1.435044
10	1	0	1.062060	-3.670838	-0.634393
11	1	0	-0.168556	-4.337614	-1.727163
12	1	0	0.925628	-3.062054	-2.297157
13	6	0	-1.439308	-2.826038	0.246139
14	1	0	-0.772474	-3.061603	1.081644
15	1	0	-2.130361	-2.039489	0.559300
16	1	0	-2.018254	-3.722215	0.001347
17	6	0	-3.529279	1.447857	-2.276156
18	1	0	-4.052579	2.392352	-2.454024
19	1	0	-3.151447	1.077807	-3.233637
20	1	0	-4.249705	0.723689	-1.881995
21	6	0	-2.863620	2.161605	0.062525
22	1	0	-3.569795	1.454014	0.509218
23	1	0	-2.014105	2.285942	0.738785
24	1	0	-3.365064	3.128322	-0.048326
25	6	0	0.231825	0.153018	-2.963917
26	1	0	-0.047595	1.092881	-3.447792

27	1	0	1.306098	-0.000229	-3.091777
28	1	0	-0.314460	-0.665918	-3.438679

(*i*-PrO)$_2$P(O)Me isomer B PBE0/6-31+G(d,p), chloroform IEFPCM:
Sum of electronic and thermal Free Energies= -843.218585

Center Number	Atomic Number	Atomic Type	Coordinates (Angstroms) X	Y	Z
1	15	0	0.140732	0.577337	-2.562852
2	8	0	0.768991	1.784570	-1.950126
3	8	0	0.882481	-0.823367	-2.306518
4	8	0	-1.349273	0.288278	-2.009246
5	6	0	-2.136896	1.308704	-1.340287
6	1	0	-1.461920	2.131895	-1.081746
7	6	0	1.214049	-1.235834	-0.952039
8	1	0	0.602045	-0.651727	-0.253142
9	6	0	0.853053	-2.701879	-0.835334
10	1	0	1.427765	-3.294044	-1.554997
11	1	0	1.082692	-3.064980	0.171237
12	1	0	-0.212280	-2.856193	-1.026496
13	6	0	2.682627	-0.953095	-0.705249
14	1	0	3.302158	-1.516883	-1.410436
15	1	0	2.894947	0.112468	-0.823283
16	1	0	2.957335	-1.252056	0.311417
17	6	0	-3.213791	1.793566	-2.291838
18	1	0	-3.833380	2.550944	-1.801318
19	1	0	-2.778025	2.243135	-3.188997
20	1	0	-3.859611	0.963110	-2.595696
21	6	0	-2.697170	0.686649	-0.077311
22	1	0	-3.338316	-0.167405	-0.319435
23	1	0	-1.891626	0.344304	0.578517
24	1	0	-3.295051	1.422877	0.468941
25	6	0	0.029872	0.579883	-4.354295
26	1	0	1.035532	0.625893	-4.778564
27	1	0	-0.476066	-0.321696	-4.705813
28	1	0	-0.531804	1.461437	-4.672568

(i-PrO)$_2$P(O)Me isomer C PBE0/6-31+G(d,p), chloroform IEFPCM:
Sum of electronic and thermal Free Energies= -843.215346

Center Number	Atomic Number	Atomic Type	Coordinates (Angstroms) X	Y	Z
1	15	0	-0.222298	-0.553380	-1.871799
2	8	0	0.109741	0.760492	-2.485480
3	8	0	0.599190	-0.927205	-0.533865
4	8	0	-1.733349	-0.604549	-1.345580
5	6	0	-2.267256	-1.693553	-0.544156
6	1	0	-1.496237	-2.467640	-0.446105
7	6	0	2.040744	-0.747840	-0.482402
8	1	0	2.407130	-0.554454	-1.499119
9	6	0	2.339104	0.453519	0.391457
10	1	0	1.955460	0.290686	1.403967
11	1	0	3.419978	0.615692	0.452357
12	1	0	1.874728	1.352975	-0.020450
13	6	0	2.639102	-2.037713	0.040814
14	1	0	2.263173	-2.251178	1.046695
15	1	0	2.388998	-2.879845	-0.610903
16	1	0	3.729005	-1.952575	0.091250
17	6	0	-2.598364	-1.142278	0.827704
18	1	0	-3.006686	-1.935135	1.462413
19	1	0	-1.702793	-0.738595	1.306654
20	1	0	-3.343808	-0.344781	0.746265
21	6	0	-3.470994	-2.252458	-1.275359
22	1	0	-4.232001	-1.476242	-1.403645
23	1	0	-3.191596	-2.630516	-2.262975
24	1	0	-3.909254	-3.075395	-0.702213
25	6	0	-0.000656	-1.949408	-2.991464
26	1	0	-0.598088	-1.780215	-3.890566
27	1	0	1.051675	-2.021459	-3.279462
28	1	0	-0.304423	-2.888236	-2.521322

Ph_3PO PBE0/6-31+G(d,p), chloroform IEFPCM:
Sum of electronic and thermal Free Energies= -1110.328676

Center Number	Atomic Number	Atomic Type	Coordinates (Angstroms) X	Y	Z
1	15	0	-1.216398	1.375876	0.048949
2	8	0	-2.707056	1.432631	-0.174871
3	6	0	-0.273606	2.094459	-1.332688
4	6	0	-0.796353	1.936613	-2.622193

5	6	0	0.931573	2.782168	-1.154817
6	6	0	-0.113660	2.451195	-3.721234
7	1	0	-1.742891	1.420724	-2.758490
8	6	0	1.612994	3.295138	-2.257570
9	1	0	1.336588	2.927291	-0.156883
10	6	0	1.092446	3.128482	-3.539864
11	1	0	-0.525491	2.328172	-4.718804
12	1	0	2.546776	3.830951	-2.112973
13	1	0	1.623017	3.531998	-4.397692
14	6	0	-0.701375	2.281659	1.541347
15	6	0	-1.434105	3.423797	1.886437
16	6	0	0.380149	1.887330	2.336102
17	6	0	-1.080359	4.168680	3.008472
18	1	0	-2.286377	3.717032	1.279651
19	6	0	0.732320	2.636047	3.458034
20	1	0	0.943582	0.991734	2.088056
21	6	0	0.004270	3.776576	3.793369
22	1	0	-1.654392	5.052010	3.273285
23	1	0	1.571044	2.323460	4.073470
24	1	0	0.277760	4.356663	4.670251
25	6	0	-0.604398	-0.326448	0.252003
26	6	0	-1.448554	-1.243708	0.889974
27	6	0	0.655675	-0.740273	-0.192876
28	6	0	-1.030256	-2.556592	1.090338
29	1	0	-2.435516	-0.927910	1.216775
30	6	0	1.071805	-2.055179	0.010614
31	1	0	1.310969	-0.041900	-0.706574
32	6	0	0.230920	-2.962384	0.653204
33	1	0	-1.690561	-3.264481	1.583051
34	1	0	2.050195	-2.371446	-0.339671
35	1	0	0.555327	-3.987608	0.807749

$(MeO)_2P(O)Me$ isomer A PBE0/6-31+G(d,p), chloroform IEFPCM:
Sum of electronic and thermal Free Energies= -686.229522

Center Number	Atomic Number	Atomic Type	Coordinates (Angstroms) X	Y	Z
1	15	0	0.010097	0.357214	0.162667
2	8	0	-0.110759	0.190797	1.638811
3	6	0	-0.399883	1.980269	-0.480182
4	1	0	-0.264889	2.005388	-1.563359
5	1	0	-1.441426	2.204187	-0.236001
6	1	0	0.242687	2.728315	-0.010355
7	8	0	1.469838	0.122808	-0.464784

8	8	0	-0.867775	-0.713542	-0.675716
9	6	0	2.188844	-1.072663	-0.131329
10	1	0	1.681675	-1.948757	-0.544819
11	1	0	3.175753	-0.972303	-0.582764
12	1	0	2.285780	-1.172439	0.952797
13	6	0	-2.112838	-1.184215	-0.147352
14	1	0	-2.398531	-2.045341	-0.752269
15	1	0	-2.000722	-1.481708	0.897765
16	1	0	-2.883144	-0.410885	-0.231203

$(MeO)_2P(O)Me$ isomer B PBE0/6-31+G(d,p), chloroform IEFPCM:
Sum of electronic and thermal Free Energies= -686.226505

Center Number	Atomic Number	Atomic Type	Coordinates (Angstroms) X	Y	Z
1	15	0	0.020135	0.079849	0.156182
2	8	0	0.011463	-0.174332	1.624008
3	6	0	0.063901	1.826171	-0.281891
4	1	0	0.067215	1.944951	-1.367573
5	1	0	-0.809893	2.330152	0.139404
6	1	0	0.960956	2.286719	0.140084
7	8	0	1.209861	-0.623581	-0.668952
8	8	0	-1.200659	-0.563031	-0.672630
9	6	0	2.534212	-0.607288	-0.122957
10	1	0	2.534348	-0.988167	0.901202
11	1	0	3.134738	-1.256965	-0.759640
12	1	0	2.951653	0.404675	-0.143333
13	6	0	-2.522331	-0.495940	-0.124104
14	1	0	-3.149406	-1.118613	-0.762198
15	1	0	-2.536043	-0.880451	0.898595
16	1	0	-2.898603	0.532041	-0.140135

$(MeO)_2P(O)Me$ isomer C PBE0/6-31+G(d,p), chloroform IEFPCM:
Sum of electronic and thermal Free Energies= -686.226152

Center Number	Atomic Number	Atomic Type	Coordinates (Angstroms) X	Y	Z
1	15	0	0.034516	0.414807	-0.212985
2	8	0	0.666555	1.242631	-1.272530
3	6	0	-0.110446	1.238467	1.381592
4	1	0	-0.601112	0.598543	2.118787
5	1	0	0.887458	1.497197	1.745102

6	1	0	-0.684484	2.159388	1.253988
7	8	0	-1.434658	-0.073448	-0.632845
8	8	0	0.756728	-1.000762	0.081859
9	6	0	-2.178583	-1.030356	0.129097
10	1	0	-2.436209	-0.628750	1.114082
11	1	0	-3.094429	-1.220112	-0.430767
12	1	0	-1.612369	-1.958325	0.238596
13	6	0	2.183276	-1.062626	0.189587
14	1	0	2.441488	-2.119774	0.255154
15	1	0	2.654835	-0.619922	-0.691178
16	1	0	2.528087	-0.549772	1.093409

Me_3PO PBE0/6-31+G(d,p), chloroform IEFPCM:
Sum of electronic and thermal Free Energies= -535.920587

Center Number	Atomic Number	Atomic Type	Coordinates (Angstroms) X	Y	Z
1	15	0	-1.225175	1.330603	0.081216
2	6	0	-0.508394	2.150700	1.534026
3	1	0	-0.725554	3.221336	1.486768
4	1	0	-0.965851	1.741419	2.438918
5	1	0	0.574611	2.004497	1.579880
6	6	0	-0.327280	2.006549	-1.345140
7	1	0	-0.668481	1.505579	-2.255246
8	1	0	-0.542900	3.074925	-1.432740
9	1	0	0.752109	1.864227	-1.238772
10	6	0	-0.701412	-0.403950	0.200421
11	1	0	-1.150087	-0.855251	1.089628
12	1	0	-1.052395	-0.946644	-0.681605
13	1	0	0.387374	-0.486616	0.264780
14	8	0	-2.724464	1.497641	-0.020275

$EtOP(O)Me_2$ isomer A PBE0/6-31+G(d,p), chloroform IEFPCM:
Sum of electronic and thermal Free Energies= -650.322495

Center Number	Atomic Number	Atomic Type	Coordinates (Angstroms) X	Y	Z
1	15	0	-0.822014	-0.011769	0.152789
2	8	0	-0.983826	0.252478	1.620717
3	8	0	0.693706	-0.390177	-0.303733
4	6	0	1.793221	0.413347	0.161400
5	1	0	1.697984	0.558336	1.242610

6	1	0	1.750835	1.395850	-0.325602
7	6	0	3.078399	-0.300110	-0.183685
8	1	0	3.165767	-0.448093	-1.264062
9	1	0	3.932906	0.295112	0.152975
10	1	0	3.122900	-1.276321	0.307515
11	6	0	-1.706484	-1.454713	-0.465387
12	1	0	-1.384978	-2.333583	0.098905
13	1	0	-2.778871	-1.306905	-0.313929
14	1	0	-1.505638	-1.611309	-1.527954
15	6	0	-1.295721	1.403776	-0.871703
16	1	0	-1.131201	1.193460	-1.931641
17	1	0	-2.352465	1.629076	-0.704780
18	1	0	-0.708049	2.278068	-0.579307

EtOP(O)Me_2 isomer B PBE0/6-31+G(d,p), chloroform IEFPCM:
Sum of electronic and thermal Free Energies= -650.318228

Center Number	Atomic Number	Atomic Type	Coordinates (Angstroms) X	Y	Z
1	15	0	-0.825532	0.006330	0.088938
2	8	0	-0.891018	0.230956	1.566576
3	8	0	0.717626	-0.243225	-0.358305
4	6	0	1.123787	-0.444033	-1.719419
5	1	0	0.866649	0.441924	-2.312548
6	1	0	0.592690	-1.307138	-2.139324
7	6	0	2.615764	-0.678872	-1.736462
8	1	0	2.875589	-1.565824	-1.151987
9	1	0	2.954108	-0.830422	-2.766152
10	1	0	3.147278	0.181450	-1.320328
11	6	0	-1.746881	-1.448291	-0.464433
12	1	0	-1.352486	-2.330387	0.046935
13	1	0	-2.797684	-1.322718	-0.189727
14	1	0	-1.680497	-1.593973	-1.546005
15	6	0	-1.410037	1.412264	-0.887447
16	1	0	-1.361717	1.219948	-1.962686
17	1	0	-2.447950	1.619589	-0.613376
18	1	0	-0.801541	2.287808	-0.645737

Table S21. Coordinates of optimized structures for tricoordinate and tetracoordinate phosphorus compounds, PBE0 (pbe1pbe)/6-31+G(d), no solvent.

PH_3 PBE0/6-31+G(d):
Sum of electronic and thermal Free Energies= -342.979461

Center Number	Atomic Number	Atomic Type	Coordinates (Angstroms) X	Y	Z
1	15	0	-0.000019	0.000020	0.133119
2	1	0	0.000037	1.195255	-0.635964
3	1	0	-1.035032	-0.597664	-0.635969
4	1	0	1.035014	-0.597610	-0.635983

$PMeH_2$ PBE0/6-31+G(d):
Sum of electronic and thermal Free Energies= -382.222142

Center Number	Atomic Number	Atomic Type	Coordinates (Angstroms) X	Y	Z
1	15	0	0.671084	0.000005	-0.131260
2	1	0	0.937801	-1.035556	0.807995
3	1	0	0.937801	1.035486	0.808083
4	6	0	-1.184638	-0.000001	0.027265
5	1	0	-1.584994	0.881824	-0.483196
6	1	0	-1.584994	-0.881785	-0.483269
7	1	0	-1.541526	-0.000044	1.060703

PMe_2H PBE0/6-31+G(d):
Sum of electronic and thermal Free Energies= -421.467376

Center Number	Atomic Number	Atomic Type	Coordinates (Angstroms) X	Y	Z
1	15	0	0.000000	-0.661376	-0.122776
2	1	0	0.000000	-1.096600	1.233899
3	6	0	1.422506	0.523589	0.023694
4	1	0	2.353866	-0.036697	0.158433
5	1	0	1.515175	1.092126	-0.908096
6	1	0	1.313158	1.229887	0.854128
7	6	0	-1.422506	0.523589	0.023694
8	1	0	-1.515175	1.092126	-0.908096
9	1	0	-2.353866	-0.036697	0.158433

10	1	0	-1.313158	1.229887	0.854128

PMe3 PBE0/6-31+G(d):

Sum of electronic and thermal Free Energies= -460.714826

Center Number	Atomic Number	Atomic Type	Coordinates (Angstroms) X	Y	Z
1	15	0	-0.000465	-0.000327	-0.607263
2	6	0	0.905403	1.354742	0.274666
3	1	0	0.495062	2.327096	-0.019433
4	1	0	1.961980	1.343130	-0.015025
5	6	0	0.722098	-1.461938	0.273611
6	1	0	0.185244	-2.371808	-0.016758
7	1	0	0.675443	-1.360497	1.365878
8	1	0	0.840399	1.262674	1.366862
9	1	0	1.769376	-1.590768	-0.021331
10	6	0	-1.626719	0.106753	0.274708
11	1	0	-1.515129	0.099986	1.366997
12	1	0	-2.262892	-0.736001	-0.017517
13	1	0	-2.145464	1.026318	-0.018045

PPh3 PBE0/6-31+G(d):

Sum of electronic and thermal Free Energies= -1035.118033

Center Number	Atomic Number	Atomic Type	Coordinates (Angstroms) X	Y	Z
1	15	0	0.001362	0.001472	-1.224758
2	6	0	-1.599116	-0.421833	-0.416054
3	6	0	-2.357864	-1.441788	-1.007185
4	6	0	-2.106441	0.221159	0.719152
5	6	0	-3.580705	-1.827196	-0.464270
6	1	0	-1.986184	-1.936348	-1.902862
7	6	0	-3.337542	-0.155548	1.255185
8	1	0	-1.539489	1.022825	1.186222
9	6	0	-4.075050	-1.182209	0.668907
10	1	0	-4.153493	-2.623631	-0.933216
11	1	0	-3.719476	0.355583	2.135869
12	1	0	-5.034461	-1.474158	1.088861
13	6	0	0.433656	1.598987	-0.415868
14	6	0	1.259223	1.717256	0.707931
15	6	0	-0.086109	2.765327	-0.994783
16	6	0	1.548904	2.971232	1.245057

17	1	0	1.680580	0.825437	1.165084
18	6	0	0.191141	4.016341	-0.450183
19	1	0	-0.712800	2.690685	-1.881763
20	6	0	1.012958	4.122378	0.671473
21	1	0	2.194672	3.046656	2.116856
22	1	0	-0.224981	4.909896	-0.909221
23	1	0	1.239791	5.098950	1.092066
24	6	0	1.168506	-1.173291	-0.416710
25	6	0	2.434951	-1.314598	-1.001289
26	6	0	0.859579	-1.942817	0.710678
27	6	0	3.377646	-2.184114	-0.459106
28	1	0	2.682431	-0.738458	-1.891168
29	6	0	1.798674	-2.824065	1.245775
30	1	0	-0.120826	-1.856489	1.172373
31	6	0	3.060014	-2.944056	0.666192
32	1	0	4.356612	-2.277473	-0.923050
33	1	0	1.542194	-3.417203	2.120506
34	1	0	3.790632	-3.631744	1.084934

Methoxyphospholane PBE0/6-31+G(d):
Sum of electronic and thermal Free Energies= -684.993079

Center Number	Atomic Number	Atomic Type	Coordinates (Angstroms) X	Y	Z
1	8	0	-0.575767	1.065459	-0.681327
2	6	0	-1.475893	0.999726	0.428164
3	6	0	-1.947670	-0.451126	0.467522
4	1	0	-0.944182	1.275720	1.346415
5	1	0	-2.288466	1.707204	0.246955
6	1	0	-2.187338	-0.785378	1.480942
7	1	0	-2.812800	-0.613968	-0.185478
8	15	0	0.315479	-0.333261	-0.746123
9	8	0	-0.862509	-1.240387	-0.027961
10	6	0	2.641378	0.224734	0.365027
11	1	0	3.242865	-0.184870	1.179928
12	1	0	3.033164	-0.147797	-0.590982
13	1	0	2.712344	1.318445	0.378221
14	8	0	1.299780	-0.193462	0.580028

P(OMe)$_3$ isomer A PBE0/6-31+G(d):
Sum of electronic and thermal Free Energies= -686.164323

Center Number	Atomic Number	Atomic Type	Coordinates (Angstroms) X	Y	Z
1	15	0	-0.057615	-0.066042	-0.730045
2	6	0	1.569569	-1.944469	0.155776
3	1	0	1.571336	-2.735095	0.910042
4	1	0	1.424183	-2.394667	-0.835399
5	1	0	2.537459	-1.429096	0.173505
6	6	0	1.017426	1.973444	0.636960
7	1	0	1.827803	2.689427	0.484546
8	1	0	0.094733	2.502083	0.891013
9	1	0	1.278337	1.283010	1.445459
10	6	0	-2.451103	-0.429228	0.295791
11	1	0	-3.295534	0.157257	0.664542
12	1	0	-2.740345	-0.917627	-0.644875
13	1	0	-2.185228	-1.193073	1.034650
14	8	0	0.860448	1.272507	-0.598435
15	8	0	0.515346	-1.051497	0.485165
16	8	0	-1.374731	0.476096	0.100918

P(OMe)$_3$ isomer B PBE0/6-31+G(d):
Sum of electronic and thermal Free Energies= -686.162240

Center Number	Atomic Number	Atomic Type	Coordinates (Angstroms) X	Y	Z
1	6	0	2.264338	-0.805001	0.475305
2	8	0	0.844613	-0.803850	0.464201
3	15	0	0.106509	-0.177276	-0.894698
4	8	0	-1.424224	-0.575268	-0.481595
5	6	0	-1.935239	-0.816328	0.826145
6	8	0	0.233838	1.439374	-0.610753
7	1	0	2.577046	-1.426241	1.317909
8	1	0	2.672586	-1.227596	-0.452073
9	1	0	2.659161	0.209829	0.608409
10	1	0	-2.166933	0.126608	1.333310
11	1	0	-2.857386	-1.387680	0.696028
12	1	0	-1.223764	-1.390153	1.425431
13	6	0	-0.147963	2.073148	0.603529
14	1	0	-1.232383	2.228838	0.623257
15	1	0	0.351035	3.045182	0.630491

16	1	0	0.157843	1.483471	1.475297

P(OMe)$_3$ isomer C PBE0/6-31+G(d):
Sum of electronic and thermal Free Energies= -686.160934

Center Number	Atomic Number	Atomic Type	Coordinates (Angstroms) X	Y	Z
1	15	0	0.003405	-0.001051	0.459547
2	6	0	-2.025322	-1.620886	0.072959
3	1	0	-2.217023	-2.611402	-0.345431
4	1	0	-2.100965	-1.672650	1.167731
5	1	0	-2.771512	-0.915177	-0.308569
6	6	0	-0.408701	2.563634	0.072138
7	1	0	-1.182962	3.214482	-0.340052
8	1	0	-0.411686	2.651246	1.167218
9	1	0	0.568845	2.874355	-0.312975
10	6	0	2.431855	-0.924279	0.075047
11	1	0	3.379344	-0.583526	-0.348168
12	1	0	2.515968	-0.948742	1.170109
13	1	0	2.211933	-1.932272	-0.293778
14	8	0	-0.711552	1.240538	-0.348580
15	8	0	-0.721514	-1.239719	-0.344350
16	8	0	1.433190	-0.008444	-0.353258

PCl$_3$ experimental geometry PBE0/6-31+G(d):
Sum of electronic and thermal Free Energies= -1721.387772
(not a minimum, but 0 negative frequencies)

Center Number	Atomic Number	Atomic Type	Coordinates (Angstroms) X	Y	Z
1	15	0	0.000000	0.000000	0.727649
2	17	0	-0.024723	1.802585	-0.233562
3	17	0	-1.562204	-0.926353	-0.207892
4	17	0	1.569915	-0.893466	-0.226780

PCl_3 PBE0/6-31+G(d):
Sum of electronic and thermal Free Energies= -1721.389915

Center Number	Atomic Number	Atomic Type	Coordinates (Angstroms) X	Y	Z
1	15	0	-0.000027	-0.000042	0.717325
2	17	0	-0.025078	1.835076	-0.230309
3	17	0	-1.590019	-0.942854	-0.204200
4	17	0	1.598111	-0.909416	-0.223401

H_3PO_4 PBE0/6-31+G(d):
Sum of electronic and thermal Free Energies= -643.679785

Center Number	Atomic Number	Atomic Type	Coordinates (Angstroms) X	Y	Z
1	15	0	-1.286314	1.085942	-0.148712
2	8	0	-1.124880	1.830911	1.261404
3	1	0	-0.270330	2.282138	1.329918
4	8	0	-0.728429	-0.379041	0.185369
5	1	0	-0.519825	-0.873583	-0.621161
6	8	0	-2.867691	0.831362	-0.209714
7	1	0	-3.329258	1.560207	-0.650542
8	8	0	-0.687639	1.762673	-1.317279

$PH_4^+ BF_4^-$ PBE0/6-31+G(d):
Sum of electronic and thermal Free Energies= -767.583213

Center Number	Atomic Number	Atomic Type	Coordinates (Angstroms) X	Y	Z
1	15	0	2.082064	0.089423	-0.029175
2	1	0	1.291402	-0.065819	1.118264
3	1	0	2.441622	-1.107348	-0.646033
4	1	0	1.622410	1.052181	-0.924084
5	1	0	3.319465	0.598981	0.416820
6	5	0	-0.942912	-0.099635	0.024995
7	9	0	0.123792	-0.771507	-0.762979
8	9	0	-2.157918	-0.530654	-0.398326
9	9	0	-0.657073	-0.432525	1.361238
10	9	0	-0.716682	1.267026	-0.169117

$P(OPh)_4^+ PF_6^-$ PBE0/6-31+G(d):
Sum of electronic and thermal Free Energies= -2506.938104

Center Number	Atomic Number	Atomic Type	Coordinates (Angstroms) X	Y	Z
1	15	0	-0.875062	0.392813	-0.169939
2	8	0	-2.300975	1.090849	-0.124522
3	8	0	0.116063	1.546942	0.129955
4	8	0	-0.912909	-0.749500	0.877358
5	8	0	-0.568852	-0.191879	-1.577568
6	6	0	-0.084737	0.555957	-2.688209
7	6	0	-0.996243	1.258497	-3.461934
8	6	0	1.268088	0.491436	-2.973852
9	6	0	-0.520022	1.940025	-4.580552
10	1	0	-2.050362	1.273995	-3.202046
11	6	0	1.721777	1.181283	-4.097034
12	1	0	1.944305	-0.062373	-2.330897
13	6	0	0.836647	1.901631	-4.897400
14	1	0	-1.214457	2.498386	-5.202332
15	1	0	2.780972	1.151990	-4.335842
16	1	0	1.204020	2.434972	-5.769811
17	6	0	-3.504698	0.398421	0.086726
18	6	0	-4.221056	-0.062545	-1.009201
19	6	0	-3.965358	0.256002	1.387952
20	6	0	-5.441343	-0.698980	-0.786190
21	1	0	-3.833945	0.074343	-2.014354
22	6	0	-5.185585	-0.383239	1.594081
23	1	0	-3.375578	0.636311	2.216536
24	6	0	-5.922915	-0.861124	0.511397
25	1	0	-6.014952	-1.065443	-1.633000
26	1	0	-5.558488	-0.506880	2.606885
27	1	0	-6.874258	-1.357933	0.679151
28	6	0	-0.746173	-2.155827	0.737839
29	6	0	0.393483	-2.726572	1.272632
30	6	0	-1.765740	-2.887579	0.149491
31	6	0	0.521016	-4.111632	1.187095
32	1	0	1.172005	-2.111199	1.708433
33	6	0	-1.617795	-4.271257	0.076759
34	1	0	-2.654562	-2.395682	-0.235352
35	6	0	-0.476348	-4.882259	0.593005
36	1	0	1.420832	-4.578349	1.576581
37	1	0	-2.400301	-4.867636	-0.384408
38	1	0	-0.362352	-5.960796	0.527552
39	6	0	0.111858	2.323457	1.318889
40	6	0	-0.741693	3.413941	1.394645

41	6	0	1.015976	1.989711	2.313264
42	6	0	-0.694502	4.202293	2.542854
43	1	0	-1.415912	3.642688	0.575386
44	6	0	1.043994	2.792423	3.452979
45	1	0	1.690274	1.148108	2.186803
46	6	0	0.193518	3.890440	3.571394
47	1	0	-1.349519	5.065308	2.625858
48	1	0	1.749185	2.556681	4.244940
49	1	0	0.229930	4.511547	4.462258
50	15	0	3.507729	-0.623478	0.340989
51	9	0	3.440583	1.006098	0.357507
52	9	0	5.035482	-0.596168	0.852848
53	9	0	2.954950	-0.643144	1.897274
54	9	0	3.502551	-2.249127	0.333256
55	9	0	3.973704	-0.597421	-1.219350
56	9	0	1.917579	-0.645392	-0.164830

O=P(OCH2)3P=O PBE0/6-31+G(d):
Sum of electronic and thermal Free Energies= -1175.949373

Center Number	Atomic Number	Atomic Type	Coordinates (Angstroms) X	Y	Z
1	6	0	0.622763	-1.322913	-0.934714
2	1	0	1.068722	-2.298541	-0.721389
3	6	0	0.622477	1.470876	-0.676899
4	1	0	1.068920	1.774795	-1.627989
5	6	0	0.622295	-0.148879	1.613552
6	1	0	1.068170	0.523796	2.351775
7	15	0	-1.440488	-0.000675	-0.000117
8	8	0	-2.899987	-0.000890	-0.000304
9	8	0	-0.745979	1.121356	-0.938914
10	8	0	-0.746666	0.251797	1.441331
11	8	0	-0.745864	-1.374519	-0.502120
12	1	0	0.666513	-1.175441	1.995022
13	1	0	0.667386	-1.139718	-2.014396
14	1	0	0.666083	2.314013	0.022053
15	15	0	1.512778	-0.000486	0.000744
16	8	0	3.003668	-0.000689	0.000721

$P(OMe)_4^+$ BF_4^- isomer A PBE0/6-31+G(d):
Sum of electronic and thermal Free Energies= -1225.192551

Center Number	Atomic Number	Atomic Type	Coordinates (Angstroms) X	Y	Z
1	15	0	-1.355818	0.027962	-0.140518
2	8	0	-0.394951	-0.229142	-1.319158
3	8	0	-2.781799	-0.379042	-0.684913
4	8	0	-1.221561	-0.815848	1.157048
5	8	0	-1.317050	1.545154	0.207819
6	6	0	0.010703	-1.526334	-1.832796
7	1	0	-0.872541	-2.056261	-2.196483
8	1	0	0.701135	-1.295756	-2.640736
9	1	0	0.539684	-2.064864	-1.046986
10	6	0	-0.597621	2.555579	-0.554585
11	1	0	-1.053900	2.652674	-1.541560
12	1	0	-0.723253	3.473570	0.017688
13	1	0	0.451580	2.268327	-0.612585
14	6	0	-3.948526	-0.366651	0.155265
15	1	0	-4.777135	-0.663435	-0.487219
16	1	0	-3.827419	-1.081165	0.972493
17	1	0	-4.121739	0.639706	0.546357
18	6	0	-0.325362	-0.609675	2.289839
19	1	0	-0.201901	0.457717	2.468404
20	1	0	-0.828332	-1.099367	3.123479
21	1	0	0.631588	-1.070268	2.053198
22	9	0	2.569838	0.044245	-1.258694
23	9	0	2.028864	-1.385940	0.460364
24	9	0	1.493641	0.836820	0.611926
25	9	0	3.696741	0.179851	0.749288
26	5	0	2.491839	-0.078705	0.131905

$P(OMe)_4^+$ BF_4^- isomer B PBE0/6-31+G(d):
Sum of electronic and thermal Free Energies= -1225.190805

Center Number	Atomic Number	Atomic Type	Coordinates (Angstroms) X	Y	Z
1	15	0	-1.533893	-0.152488	0.445027
2	8	0	-0.812351	0.163470	1.771741
3	8	0	-2.837404	-0.849995	1.010464
4	8	0	-2.038735	1.054207	-0.398645
5	8	0	-0.802659	-1.142647	-0.507670
6	6	0	-0.065603	1.324202	2.223275

7	1	0	-0.372635	2.207359	1.664578
8	1	0	-0.317334	1.417626	3.279873
9	1	0	0.991751	1.122033	2.066459
10	6	0	0.215240	-2.097743	-0.096530
11	1	0	-0.263218	-2.897277	0.474518
12	1	0	0.638057	-2.471875	-1.026122
13	1	0	0.989560	-1.585908	0.474603
14	6	0	-3.807654	-1.404584	0.109649
15	1	0	-4.571947	-1.856635	0.741457
16	1	0	-4.250140	-0.612582	-0.501031
17	1	0	-3.349774	-2.166692	-0.527139
18	6	0	-1.552802	1.518468	-1.688191
19	1	0	-0.907838	0.768364	-2.142946
20	1	0	-2.449800	1.697411	-2.282431
21	1	0	-0.990612	2.434484	-1.522074
22	9	0	2.977912	1.752461	-0.793396
23	9	0	0.774873	1.238320	-0.368931
24	9	0	2.433203	0.033450	0.645321
25	9	0	2.058445	-0.198113	-1.614587
26	5	0	2.109017	0.710252	-0.554360

$(i\text{-PrO})_2P(O)H$ isomer A PBE0/6-31+G(d):
Sum of electronic and thermal Free Energies= -803.926120

Center Number	Atomic Number	Atomic Type	Coordinates (Angstroms) X	Y	Z
1	15	0	-0.003483	0.996486	-0.352617
2	1	0	-0.205095	0.937361	-1.747662
3	8	0	0.573981	2.273028	0.113082
4	8	0	0.844490	-0.341690	-0.054196
5	8	0	-1.430167	0.636603	0.264031
6	6	0	-2.169787	-0.538306	-0.151692
7	1	0	-1.728280	-0.920423	-1.083996
8	6	0	2.270456	-0.356725	-0.328086
9	1	0	2.473747	0.341145	-1.153432
10	6	0	3.022525	0.093339	0.909251
11	1	0	2.809028	-0.579974	1.747052
12	1	0	4.101938	0.078987	0.719118
13	1	0	2.728997	1.108914	1.188934
14	6	0	2.612041	-1.767299	-0.762946
15	1	0	2.383961	-2.478101	0.039072
16	1	0	2.042523	-2.054253	-1.652762
17	1	0	3.679874	-1.842234	-0.995752
18	6	0	-2.055668	-1.593135	0.931531

19	1	0	-2.613773	-2.491365	0.643584
20	1	0	-1.009834	-1.867263	1.094471
21	1	0	-2.469658	-1.214227	1.872267
22	6	0	-3.595278	-0.093600	-0.411654
23	1	0	-4.033112	0.325618	0.500599
24	1	0	-3.631238	0.671681	-1.193267
25	1	0	-4.205731	-0.945867	-0.730143

(*i*-PrO)$_2$P(O)H isomer B PBE0/6-31+G(d):
Sum of electronic and thermal Free Energies= -803.929481

Center Number	Atomic Number	Atomic Type	Coordinates (Angstroms) X	Y	Z
1	15	0	0.009128	-0.897946	-0.484085
2	1	0	0.267320	-1.525621	-1.709827
3	8	0	0.047835	-1.781811	0.705957
4	8	0	-1.370332	-0.191453	-0.870551
5	8	0	1.030896	0.347253	-0.417611
6	6	0	2.238569	0.276016	0.382281
7	1	0	2.020163	-0.347369	1.256919
8	6	0	-2.098818	0.591279	0.113626
9	1	0	-1.383080	0.955823	0.862187
10	6	0	-2.696916	1.768241	-0.628431
11	1	0	-3.382971	1.417710	-1.407515
12	1	0	-3.257660	2.405111	0.064747
13	1	0	-1.913699	2.369705	-1.099646
14	6	0	-3.132640	-0.296330	0.778985
15	1	0	-3.835732	-0.684709	0.033691
16	1	0	-2.651558	-1.140566	1.281326
17	1	0	-3.698458	0.276823	1.522528
18	6	0	3.351223	-0.353190	-0.436346
19	1	0	4.278886	-0.383350	0.146111
20	1	0	3.102846	-1.381988	-0.719672
21	1	0	3.533048	0.226838	-1.348181
22	6	0	2.549913	1.693651	0.816319
23	1	0	2.728747	2.333613	-0.054993
24	1	0	1.719406	2.115126	1.391296
25	1	0	3.446655	1.706517	1.445545

$(MeO)_2P(O)H$ isomer A PBE0/6-31+G(d):
Sum of electronic and thermal Free Energies= -646.950502

Center Number	Atomic Number	Atomic Type	Coordinates (Angstroms) X	Y	Z
1	15	0	-0.002619	0.512707	-0.398190
2	8	0	1.433804	-0.164704	-0.583939
3	8	0	-0.966750	-0.777139	-0.298150
4	8	0	-0.183704	1.501042	0.689208
5	6	0	2.077148	-0.774498	0.540073
6	1	0	3.072922	-1.061650	0.200348
7	1	0	2.156755	-0.064721	1.368640
8	1	0	1.523980	-1.664118	0.856553
9	6	0	-2.269689	-0.643998	0.275857
10	1	0	-2.603335	-1.654169	0.518924
11	1	0	-2.234434	-0.029541	1.179380
12	1	0	-2.960123	-0.198402	-0.448857
13	1	0	-0.174494	1.000677	-1.700173

$(MeO)_2P(O)H$ isomer B PBE0/6-31+G(d):
Sum of electronic and thermal Free Energies= -646.947220

Center Number	Atomic Number	Atomic Type	Coordinates (Angstroms) X	Y	Z
1	15	0	0.023758	0.508003	0.268672
2	8	0	-1.443534	0.274235	-0.319910
3	8	0	0.741824	-0.890269	-0.093002
4	8	0	0.699003	1.735926	-0.189452
5	6	0	-2.181983	-0.899189	0.020039
6	1	0	-3.189657	-0.750319	-0.370293
7	1	0	-1.726841	-1.781129	-0.438874
8	1	0	-2.231786	-1.032262	1.107886
9	6	0	2.166130	-0.984299	-0.010752
10	1	0	2.435872	-1.945255	-0.451293
11	1	0	2.637303	-0.169941	-0.567490
12	1	0	2.494836	-0.957965	1.034545
13	1	0	-0.157916	0.426123	1.664959

$(MeO)_2P(O)H$ isomer C PBE0/6-31+G(d):
Sum of electronic and thermal Free Energies= -646.949207

Center Number	Atomic Number	Atomic Type	Coordinates (Angstroms) X	Y	Z
1	15	0	0.000004	0.176853	0.375298
2	8	0	-1.209663	-0.793044	-0.027516
3	8	0	1.209670	-0.793038	-0.027535
4	8	0	-0.000005	1.544779	-0.189605
5	6	0	-2.525901	-0.241599	-0.118064
6	1	0	-3.150338	-1.020753	-0.557086
7	1	0	-2.908668	0.012796	0.877034
8	1	0	-2.530352	0.646976	-0.755246
9	6	0	2.525911	-0.241592	-0.118046
10	1	0	3.150359	-1.020743	-0.557058
11	1	0	2.530378	0.646988	-0.755220
12	1	0	2.908654	0.012795	0.877064
13	1	0	0.000017	0.167189	1.783575

$Ph_4P^+ Br^-$ PBE0/6-31+G(d):
Sum of electronic and thermal Free Energies= -3837.807464

Center Number	Atomic Number	Atomic Type	Coordinates (Angstroms) X	Y	Z
1	15	0	0.739670	-0.000836	-0.018839
2	6	0	0.379044	-1.483548	-1.000155
3	6	0	-0.958646	-1.747945	-1.326054
4	6	0	1.394764	-2.353077	-1.420754
5	6	0	-1.261636	-2.865939	-2.098373
6	1	0	-1.781557	-1.128812	-0.943617
7	6	0	1.074725	-3.463742	-2.196256
8	1	0	2.429052	-2.184128	-1.138966
9	6	0	-0.251858	-3.716142	-2.543004
10	1	0	-2.302657	-3.066411	-2.337365
11	1	0	1.864475	-4.136102	-2.521088
12	1	0	-0.497460	-4.585061	-3.148731
13	6	0	0.384977	1.522554	-0.937804
14	6	0	-0.951462	1.804113	-1.254264
15	6	0	1.403951	2.405565	-1.320752
16	6	0	-1.250101	2.953735	-1.980396
17	1	0	-1.776656	1.172300	-0.898438
18	6	0	1.088265	3.547976	-2.050567
19	1	0	2.437218	2.222005	-1.044335

20	6	0	-0.237075	3.818289	-2.388526
21	1	0	-2.290174	3.167057	-2.212262
22	1	0	1.880395	4.230795	-2.346493
23	1	0	-0.479152	4.712006	-2.958535
24	6	0	2.501975	-0.013306	0.423416
25	6	0	2.873795	-0.040400	1.771461
26	6	0	3.496240	0.004950	-0.567428
27	6	0	4.222142	-0.048738	2.123503
28	1	0	2.110804	-0.054743	2.544815
29	6	0	4.840205	-0.003634	-0.208916
30	1	0	3.224692	0.025954	-1.620193
31	6	0	5.204404	-0.030325	1.137304
32	1	0	4.501545	-0.069638	3.173329
33	1	0	5.603223	0.010646	-0.982554
34	1	0	6.255069	-0.036801	1.415264
35	6	0	-0.239809	-0.030585	1.487107
36	6	0	-0.618005	1.172196	2.092063
37	6	0	-0.623515	-1.255927	2.041230
38	6	0	-1.379903	1.143853	3.253580
39	1	0	-0.349594	2.124198	1.642659
40	6	0	-1.385791	-1.272513	3.202715
41	1	0	-0.359562	-2.189649	1.552479
42	6	0	-1.766521	-0.075511	3.804347
43	1	0	-1.704465	2.076598	3.705659
44	1	0	-1.714944	-2.221810	3.615325
45	1	0	-2.391434	-0.092730	4.693162
46	35	0	-3.553471	0.002923	0.203744

Me_4P^+ Br^- PBE0/6-31+G(d):

Sum of electronic and thermal Free Energies= -3071.955940

Center Number	Atomic Number	Atomic Type	Coordinates (Angstroms) X	Y	Z
1	15	0	-1.523729	0.000461	0.000390
2	6	0	-3.340572	0.000585	-0.005129
3	1	0	-3.719653	-0.922190	0.445210
4	1	0	-3.719964	0.852830	0.567414
5	1	0	-3.715915	0.070987	-1.030888
6	6	0	-0.951888	1.535051	-0.748873
7	1	0	-1.315378	2.392946	-0.174493
8	1	0	0.149708	1.492120	-0.722931
9	1	0	-1.307613	1.604519	-1.781701
10	6	0	-0.950933	-1.416804	-0.951877
11	1	0	0.150492	-1.378493	-0.917327

12	1	0	-1.315927	-2.345402	-0.501872
13	1	0	-1.303712	-1.343211	-1.985394
14	6	0	-0.960219	-0.119510	1.706753
15	1	0	-1.320523	-1.050137	2.156545
16	1	0	0.141596	-0.114932	1.661864
17	1	0	-1.323750	0.735496	2.285407
18	35	0	2.074096	0.003936	0.017217

(*i*-PrO)$_2$P(O)Me isomer A PBE0/6-31+G(d):
Sum of electronic and thermal Free Energies= -843.181092

Center Number	Atomic Number	Atomic Type	Coordinates (Angstroms) X	Y	Z
1	15	0	0.101302	1.137102	0.035670
2	8	0	0.787658	1.967307	-0.980240
3	8	0	-1.112305	0.295638	-0.613464
4	8	0	1.037910	0.046244	0.779664
5	6	0	2.322068	-0.323349	0.215438
6	1	0	2.731891	0.566148	-0.276770
7	6	0	-1.862469	-0.706263	0.106531
8	1	0	-1.359649	-0.910229	1.061556
9	6	0	-3.263575	-0.176406	0.355217
10	1	0	-3.754265	0.051773	-0.597262
11	1	0	-3.865225	-0.923696	0.885057
12	1	0	-3.246243	0.737377	0.957785
13	6	0	-1.859790	-1.968211	-0.735240
14	1	0	-2.313646	-1.770615	-1.712508
15	1	0	-0.839076	-2.326888	-0.895360
16	1	0	-2.432847	-2.759404	-0.238689
17	6	0	3.199420	-0.728208	1.382655
18	1	0	4.200335	-0.995583	1.026096
19	1	0	3.296343	0.092646	2.100069
20	1	0	2.775306	-1.594944	1.902355
21	6	0	2.144897	-1.430260	-0.807116
22	1	0	1.703534	-2.319399	-0.341595
23	1	0	1.502178	-1.104000	-1.630965
24	1	0	3.117332	-1.710750	-1.227876
25	6	0	-0.596443	2.029374	1.439636
26	1	0	0.202277	2.593461	1.929252
27	1	0	-1.354194	2.729346	1.076241
28	1	0	-1.045378	1.348856	2.169240

(i-PrO)$_2$P(O)Me isomer B PBE0/6-31+G(d):
Sum of electronic and thermal Free Energies= -843.187041

Center Number	Atomic Number	Atomic Type	Coordinates (Angstroms) X	Y	Z
1	15	0	-0.028601	0.883794	0.012766
2	8	0	-0.104872	1.126593	1.477596
3	8	0	1.392505	0.414175	-0.573846
4	8	0	-1.020423	-0.294090	-0.490333
5	6	0	-2.100827	-0.773450	0.345157
6	1	0	-1.807505	-0.620690	1.390051
7	6	0	2.092867	-0.695635	0.040802
8	1	0	1.355320	-1.332378	0.547991
9	6	0	2.743265	-1.476628	-1.082955
10	1	0	3.451711	-0.841733	-1.626800
11	1	0	3.289660	-2.336584	-0.679607
12	1	0	1.990685	-1.840648	-1.789129
13	6	0	3.082529	-0.154458	1.054833
14	1	0	3.814397	0.495260	0.561384
15	1	0	2.563284	0.421320	1.826365
16	1	0	3.621457	-0.979185	1.535534
17	6	0	-3.361559	0.018076	0.047165
18	1	0	-4.198182	-0.361517	0.644734
19	1	0	-3.229869	1.076919	0.294406
20	1	0	-3.627268	-0.066026	-1.012899
21	6	0	-2.252134	-2.253199	0.053361
22	1	0	-2.509865	-2.415035	-0.999450
23	1	0	-1.322312	-2.789872	0.267392
24	1	0	-3.047175	-2.681100	0.674117
25	6	0	-0.364650	2.295596	-1.050418
26	1	0	0.375010	3.077651	-0.858165
27	1	0	-0.321258	2.002177	-2.102386
28	1	0	-1.360423	2.685163	-0.821123

(i-PrO)$_2$P(O)Me isomer C PBE0/6-31+G(d):
Sum of electronic and thermal Free Energies= -843.181556

Center Number	Atomic Number	Atomic Type	Coordinates (Angstroms) X	Y	Z
1	15	0	-0.135386	1.103706	-0.095186
2	8	0	-0.837111	1.818385	-1.185788
3	8	0	-1.049073	0.065152	0.745817
4	8	0	1.100648	0.234628	-0.660622

5	6	0	1.873393	-0.673785	0.153548
6	1	0	1.378333	-0.789560	1.127285
7	6	0	-2.321179	-0.386735	0.214791
8	1	0	-2.752254	0.444534	-0.354964
9	6	0	-2.111736	-1.574936	-0.705402
10	1	0	-1.655099	-2.409776	-0.160648
11	1	0	-3.074462	-1.912971	-1.105728
12	1	0	-1.469794	-1.308265	-1.551014
13	6	0	-3.192209	-0.709749	1.411679
14	1	0	-2.747865	-1.515736	2.006702
15	1	0	-3.313448	0.168428	2.053529
16	1	0	-4.184348	-1.033818	1.078321
17	6	0	1.894495	-2.015179	-0.554094
18	1	0	2.483477	-2.739673	0.019668
19	1	0	0.880805	-2.408076	-0.672945
20	1	0	2.343416	-1.910947	-1.547993
21	6	0	3.263335	-0.091571	0.339782
22	1	0	3.745216	0.051394	-0.633499
23	1	0	3.226997	0.877326	0.848024
24	1	0	3.883578	-0.768600	0.938163
25	6	0	0.534387	2.140595	1.220032
26	1	0	1.273010	2.824969	0.793107
27	1	0	-0.280972	2.724834	1.655547
28	1	0	1.000674	1.543339	2.009245

Ph_3PO PBE0/6-31+G(d):
Sum of electronic and thermal Free Energies= -1110.295029

Center Number	Atomic Number	Atomic Type	Coordinates (Angstroms) X	Y	Z
1	15	0	-0.003198	-0.006698	0.927888
2	8	0	-0.008247	-0.010661	2.429332
3	6	0	1.638678	-0.372824	0.226790
4	6	0	2.749296	0.059266	0.961087
5	6	0	1.830584	-1.050910	-0.980971
6	6	0	4.036054	-0.169242	0.481418
7	1	0	2.594835	0.557697	1.915003
8	6	0	3.120342	-1.278005	-1.458583
9	1	0	0.975138	-1.417884	-1.543562
10	6	0	4.222509	-0.834552	-0.730044
11	1	0	4.895049	0.165812	1.057308
12	1	0	3.263521	-1.810040	-2.395839
13	1	0	5.227970	-1.015653	-1.101984
14	6	0	-1.142562	-1.238760	0.217296

15	6	0	-1.377593	-2.392527	0.973612
16	6	0	-1.777001	-1.081833	-1.019148
17	6	0	-2.223756	-3.385937	0.487823
18	1	0	-0.906348	-2.493127	1.948307
19	6	0	-2.622480	-2.078515	-1.503005
20	1	0	-1.626819	-0.174519	-1.600007
21	6	0	-2.843345	-3.231634	-0.751927
22	1	0	-2.405568	-4.278552	1.081096
23	1	0	-3.116697	-1.949175	-2.462702
24	1	0	-3.506655	-4.006386	-1.128561
25	6	0	-0.499001	1.601156	0.228696
26	6	0	-1.383920	2.374562	0.988343
27	6	0	-0.042761	2.081565	-1.002872
28	6	0	-1.821917	3.606795	0.510456
29	1	0	-1.708512	2.008915	1.959534
30	6	0	-0.484458	3.314653	-1.479199
31	1	0	0.671087	1.502815	-1.584914
32	6	0	-1.376057	4.075520	-0.724913
33	1	0	-2.506530	4.205334	1.106137
34	1	0	-0.123910	3.685447	-2.435525
35	1	0	-1.716135	5.039241	-1.095987

$(MeO)_2P(O)Me$ isomer A PBE0/6-31+G(d):
Sum of electronic and thermal Free Energies= -686.208822

Center Number	Atomic Number	Atomic Type	Coordinates (Angstroms) X	Y	Z
1	15	0	-0.017572	0.379582	0.161528
2	8	0	-0.155673	0.193290	1.627780
3	6	0	-0.405794	2.013798	-0.479663
4	1	0	-0.277066	2.040358	-1.564624
5	1	0	-1.441840	2.258127	-0.227669
6	1	0	0.252914	2.751594	-0.013585
7	8	0	1.445493	0.152369	-0.466084
8	8	0	-0.905346	-0.665275	-0.705469
9	6	0	2.159852	-1.037794	-0.126723
10	1	0	1.661166	-1.914365	-0.553032
11	1	0	3.154295	-0.933136	-0.563292
12	1	0	2.238335	-1.142710	0.959568
13	6	0	-2.085904	-1.233493	-0.138482
14	1	0	-2.295994	-2.143046	-0.704854
15	1	0	-1.934435	-1.471931	0.917832
16	1	0	-2.929206	-0.541034	-0.241969

(MeO)$_2$P(O)Me isomer B PBE0/6-31+G(d):
Sum of electronic and thermal Free Energies= -686.208768

Center Number	Atomic Number	Atomic Type	Coordinates (Angstroms) X	Y	Z
1	15	0	-0.134811	0.663974	0.182837
2	8	0	-0.091763	0.559339	1.663064
3	6	0	0.167688	2.293084	-0.514902
4	1	0	0.116562	2.258458	-1.606066
5	1	0	-0.580363	2.993912	-0.134438
6	1	0	1.159543	2.635214	-0.205410
7	8	0	0.895548	-0.343147	-0.562684
8	8	0	-1.524418	0.286300	-0.532688
9	6	0	2.074225	-0.779829	0.114010
10	1	0	1.867199	-0.972707	1.170168
11	1	0	2.395613	-1.697896	-0.382028
12	1	0	2.865043	-0.026052	0.025622
13	6	0	-2.168096	-0.939398	-0.178845
14	1	0	-3.142281	-0.926711	-0.670059
15	1	0	-1.584733	-1.792358	-0.540668
16	1	0	-2.298049	-1.006912	0.905471

(MeO)$_2$P(O)Me isomer C PBE0/6-31+G(d):
Sum of electronic and thermal Free Energies= -686.204713

Center Number	Atomic Number	Atomic Type	Coordinates (Angstroms) X	Y	Z
1	15	0	0.268649	0.604837	0.157720
2	8	0	1.085544	1.478917	-0.710705
3	6	0	-0.396206	1.344344	1.658752
4	1	0	-0.929730	0.612437	2.271661
5	1	0	0.431045	1.757429	2.241973
6	1	0	-1.074460	2.156615	1.382009
7	8	0	-0.990574	-0.003790	-0.657237
8	8	0	1.033104	-0.686012	0.769552
9	6	0	-1.866423	-0.961058	-0.073529
10	1	0	-2.528040	-0.487917	0.661747
11	1	0	-2.472236	-1.364616	-0.887278
12	1	0	-1.306332	-1.772766	0.403542
13	6	0	2.006490	-1.347940	-0.043360
14	1	0	2.526380	-2.049509	0.611276
15	1	0	1.521102	-1.895911	-0.858347

16	1	0	2.712756	-0.624324	-0.459514

Me$_3$PO PBE0/6-31+G(d):
Sum of electronic and thermal Free Energies= -535.896672

Center Number	Atomic Number	Atomic Type	Coordinates (Angstroms) X	Y	Z
1	15	0	-0.115106	0.815098	0.113120
2	8	0	0.384689	1.521888	1.338548
3	6	0	0.425487	-0.921407	-0.008325
4	1	0	0.073870	-1.468516	0.872210
5	1	0	0.044746	-1.410451	-0.911483
6	1	0	1.519584	-0.955779	-0.015196
7	6	0	-1.932066	0.745210	-0.008632
8	1	0	-2.329966	1.764957	-0.015452
9	1	0	-2.264759	0.222875	-0.912060
10	1	0	-2.330448	0.230875	0.871693
11	6	0	0.425014	1.578779	-1.451047
12	1	0	0.041599	1.042314	-2.325661
13	1	0	0.074933	2.615461	-1.483059
14	1	0	1.519051	1.588595	-1.486195

EtOP(O)Me$_2$ isomer A PBE0/6-31+G(d):
Sum of electronic and thermal Free Energies= -650.297318

Center Number	Atomic Number	Atomic Type	Coordinates (Angstroms) X	Y	Z
1	15	0	0.808927	-0.027906	0.141517
2	8	0	0.915851	-0.283825	1.607042
3	8	0	-0.693951	0.303682	-0.394850
4	6	0	-1.803956	-0.426421	0.147932
5	1	0	-1.658482	-0.550792	1.226684
6	1	0	-1.835573	-1.422544	-0.314809
7	6	0	-3.069257	0.342733	-0.153291
8	1	0	-3.198020	0.477231	-1.232263
9	1	0	-3.938244	-0.201427	0.233732
10	1	0	-3.041230	1.330162	0.318202
11	6	0	1.678434	1.435605	-0.465599
12	1	0	1.322368	2.307637	0.090505
13	1	0	2.752024	1.319947	-0.288354
14	1	0	1.496940	1.591031	-1.533133
15	6	0	1.362482	-1.431266	-0.870211

16	1	0	1.240668	-1.227565	-1.938521
17	1	0	2.414704	-1.643112	-0.655744
18	1	0	0.778762	-2.318049	-0.603817

EtOP(O)Me_2 isomer B PBE0/6-31+G(d):
Sum of electronic and thermal Free Energies= -650.290734

Center Number	Atomic Number	Atomic Type	Coordinates (Angstroms) X	Y	Z
1	15	0	-0.886685	-0.005147	0.204752
2	8	0	-1.696231	-0.041398	1.449787
3	8	0	0.707906	-0.016687	0.535857
4	6	0	1.727637	0.139568	-0.447915
5	1	0	1.670629	1.147503	-0.881002
6	1	0	1.585855	-0.588557	-1.259168
7	6	0	3.065661	-0.073077	0.223672
8	1	0	3.128820	-1.080311	0.646767
9	1	0	3.876621	0.053247	-0.502416
10	1	0	3.206051	0.649391	1.033455
11	6	0	-1.154179	-1.434431	-0.882753
12	1	0	-0.919945	-2.343434	-0.320146
13	1	0	-2.209573	-1.469708	-1.170312
14	1	0	-0.542026	-1.402091	-1.789880
15	6	0	-1.157090	1.477201	-0.809218
16	1	0	-0.582470	1.469535	-1.741215
17	1	0	-2.222107	1.544580	-1.051973
18	1	0	-0.883865	2.359335	-0.221461

H_2P-PH-PH_2 isomer A, PBE0/6-31+G(d), no solvent, 213.15 K:
Sum of electronic and thermal Free Energies= -1026.578464 au

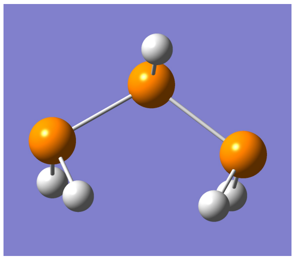

Center Number	Atomic Number	Atomic Type	Coordinates (Angstroms) X	Y	Z
1	15	0	0.000000	0.910582	-0.100152
2	1	0	-0.000001	1.329275	1.255147

3	15	0	1.823333	-0.340208	0.046159
4	1	0	1.297002	-1.500333	0.682440
5	1	0	1.709745	-0.885564	-1.260465
6	15	0	-1.823333	-0.340209	0.046151
7	1	0	-1.709753	-0.885550	-1.260479
8	1	0	-1.297001	-1.500342	0.682414

H_2P-PH-PH_2 isomer B, PBE0/6-31+G(d), no solvent, 213.15 K:
Sum of electronic and thermal Free Energies= -1026.577561

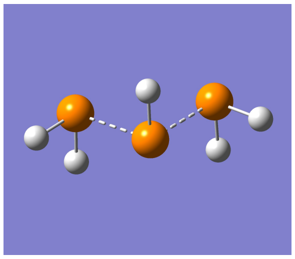

Center Number	Atomic Number	Atomic Type	Coordinates (Angstroms) X	Y	Z
1	15	0	0.000007	0.971523	0.138514
2	1	0	-0.000026	1.335974	-1.239374
3	15	0	-1.670688	-0.503858	-0.115865
4	1	0	-1.703365	-0.862091	1.261959
5	1	0	-2.693187	0.473029	0.050665
6	15	0	1.670730	-0.503816	-0.115935
7	1	0	2.693210	0.473100	0.050539
8	1	0	1.703483	-0.862039	1.261889

H_2P-PH-PH_2 isomer C, PBE0/6-31+G(d), no solvent, 213.15 K:
Sum of electronic and thermal Free Energies= -1026.577115

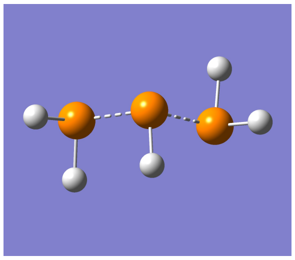

Center Number	Atomic Number	Atomic Type	Coordinates (Angstroms) X	Y	Z
1	15	0	-0.000887	0.976610	-0.104558
2	1	0	0.015878	1.336566	1.272646
3	15	0	1.656666	-0.516840	0.089340

4	1	0	1.801494	-0.706707	-1.312309
5	1	0	2.683509	0.467920	0.139045
6	15	0	-1.660704	-0.510754	-0.076304
7	1	0	-1.722720	-0.785524	1.316592
8	1	0	-2.704291	0.452511	-0.043137

H_2P-PH_2 isomer A, PBE0/6-31+G(d), no solvent, 213.15 K:
Sum of electronic and thermal Free Energies= -684.773859

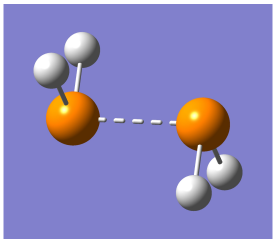

Center Number	Atomic Number	Atomic Type	Coordinates (Angstroms) X	Y	Z
1	15	0	0.000000	1.120768	0.000000
2	1	0	0.978814	1.228602	-1.027904
3	1	0	0.978814	1.228602	1.027904
4	15	0	0.000000	-1.120768	0.000000
5	1	0	-0.978814	-1.228602	-1.027904
6	1	0	-0.978814	-1.228602	1.027904

H_2P-PH_2 isomer B, PBE0/6-31+G(d), no solvent, 213.15 K:
Sum of electronic and thermal Free Energies= -684.773724

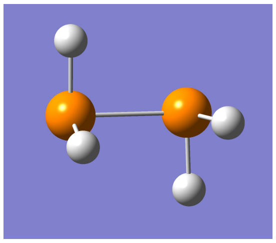

Center Number	Atomic Number	Atomic Type	Coordinates (Angstroms) X	Y	Z
1	15	0	0.000000	1.111751	-0.085602
2	1	0	1.412338	1.243551	-0.015080
3	1	0	-0.191495	1.372386	1.299110
4	15	0	0.000000	-1.111751	-0.085602
5	1	0	-1.412338	-1.243551	-0.015080
6	1	0	0.191495	-1.372386	1.299110

Table S22. Coordinates of optimized structures for 1a–34[O], B3LYP/6-31+G(d,p), IEFPCM solvation.

1a isomer A, B3LYP/6-31+G(d,p), benzene IEFPCM:
Sum of electronic and thermal Free Energies= -1310.118082

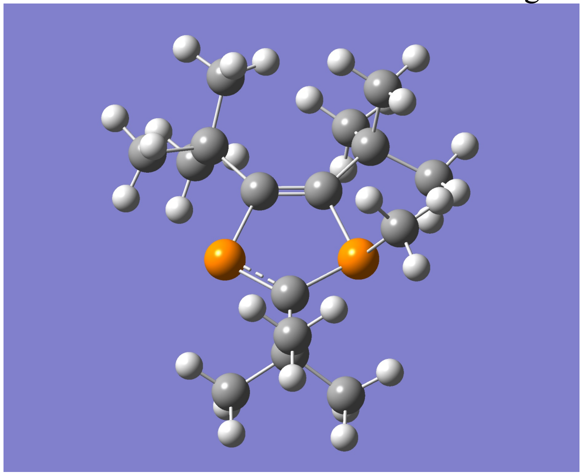

Center Number	Atomic Number	Atomic Type	Coordinates (Angstroms) X	Y	Z
1	6	0	-2.369328	4.061722	0.464237
2	6	0	-1.064708	4.314332	0.088423
3	6	0	-2.136291	6.726220	0.932199
4	15	0	-3.286493	5.500946	1.187092
5	15	0	-0.564884	6.066240	0.378412
6	6	0	-3.228195	2.751334	0.467596
7	6	0	-3.044726	1.874698	-0.793649
8	6	0	-2.961528	1.938979	1.760675
9	6	0	-4.748901	3.084293	0.471816
10	1	0	-3.280872	2.448920	-1.696111
11	1	0	-2.048110	1.459182	-0.911165
12	1	0	-3.741204	1.029988	-0.746812
13	1	0	-3.199852	2.542125	2.643402
14	1	0	-3.600532	1.047491	1.782299
15	1	0	-1.925040	1.615054	1.851212
16	1	0	-5.312009	2.148965	0.382446
17	1	0	-5.075777	3.574452	1.391369
18	1	0	-5.028362	3.723735	-0.372783
19	6	0	0.092882	3.383473	-0.411669
20	6	0	0.194221	2.044129	0.358438
21	6	0	-0.041068	3.134661	-1.936976
22	6	0	1.489210	4.033355	-0.189503
23	1	0	0.316471	2.228482	1.430826
24	1	0	-0.658940	1.385683	0.224766
25	1	0	1.079692	1.498852	0.011831

26	1	0	0.024249	4.076542	-2.490693
27	1	0	0.774250	2.487829	-2.282783
28	1	0	-0.985183	2.661518	-2.204866
29	1	0	2.256776	3.345363	-0.560597
30	1	0	1.625417	4.973686	-0.726732
31	1	0	1.686484	4.216030	0.870572
32	6	0	-2.375322	8.205127	1.279880
33	6	0	-3.442340	8.355833	2.387851
34	6	0	-1.077168	8.885187	1.773155
35	6	0	-2.888121	8.948818	0.017987
36	1	0	-4.404081	7.929390	2.085966
37	1	0	-3.126308	7.858193	3.310402
38	1	0	-3.602123	9.417744	2.607233
39	1	0	-0.289885	8.875954	1.012340
40	1	0	-1.279551	9.932097	2.026598
41	1	0	-0.685875	8.386853	2.665691
42	1	0	-3.062895	10.006004	0.251549
43	1	0	-2.169790	8.901472	-0.805197
44	1	0	-3.830032	8.513369	-0.330054
45	6	0	-0.313570	6.802914	-1.330502
46	1	0	-1.188289	6.637602	-1.964856
47	1	0	-0.143330	7.876775	-1.222184
48	1	0	0.567592	6.371613	-1.808610

1a isomer B, B3LYP/6-31+G(d,p), benzene IEFPCM:
Sum of electronic and thermal Free Energies= -1310.115709

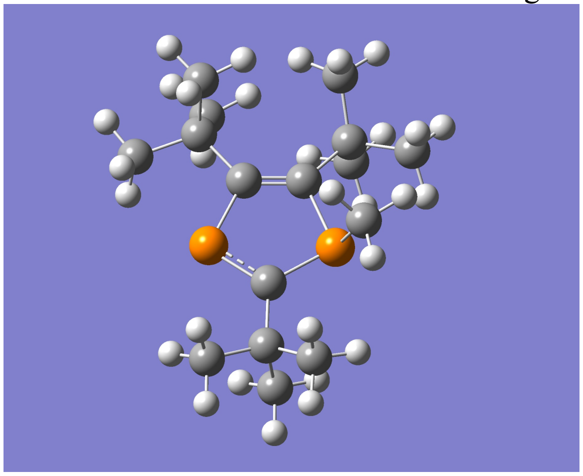

Center Number	Atomic Number	Atomic Type	Coordinates (Angstroms) X	Y	Z
1	6	0	-2.398315	4.026359	0.392542
2	6	0	-1.094973	4.272914	0.011107
3	6	0	-2.196981	6.713309	0.738799

4	15	0	-3.359362	5.500499	0.975835
5	15	0	-0.615476	6.059120	0.165693
6	6	0	-3.238050	2.702555	0.413518
7	6	0	-4.628478	2.897834	1.082225
8	6	0	-3.548271	2.271792	-1.044014
9	6	0	-2.584791	1.557889	1.223859
10	1	0	-4.548232	3.196233	2.132310
11	1	0	-5.245557	3.638859	0.566779
12	1	0	-5.164730	1.943069	1.046442
13	1	0	-2.655459	2.093684	-1.639345
14	1	0	-4.147229	1.352900	-1.042896
15	1	0	-4.132741	3.049960	-1.547304
16	1	0	-3.259992	0.694511	1.240277
17	1	0	-1.633758	1.215309	0.825701
18	1	0	-2.422048	1.870986	2.261016
19	6	0	0.115341	3.319062	-0.277568
20	6	0	1.303000	4.052484	-0.958995
21	6	0	0.657934	2.820410	1.090287
22	6	0	-0.172789	2.128426	-1.220928
23	1	0	1.068622	4.337961	-1.987787
24	1	0	1.630610	4.938676	-0.407474
25	1	0	2.156216	3.367540	-1.000805
26	1	0	-0.094735	2.287665	1.670836
27	1	0	1.510890	2.148742	0.932047
28	1	0	1.003806	3.668165	1.691047
29	1	0	0.763975	1.589663	-1.403083
30	1	0	-0.883791	1.405685	-0.830123
31	1	0	-0.541373	2.482240	-2.189367
32	6	0	-2.380230	8.161091	1.227165
33	6	0	-1.676395	9.187745	0.312244
34	6	0	-3.874770	8.540749	1.311775
35	6	0	-1.761655	8.277258	2.646999
36	1	0	-0.595559	9.017378	0.263446
37	1	0	-2.082231	9.164828	-0.704246
38	1	0	-1.828850	10.197492	0.709416
39	1	0	-4.420875	7.890160	2.001617
40	1	0	-3.976224	9.570733	1.671999
41	1	0	-4.357070	8.473488	0.330906
42	1	0	-1.859049	9.305698	3.015798
43	1	0	-2.270511	7.608848	3.348354
44	1	0	-0.698753	8.016519	2.636672
45	6	0	-0.566661	6.644890	-1.621516
46	1	0	0.340614	6.294473	-2.112379
47	1	0	-1.443320	6.289239	-2.169882
48	1	0	-0.555688	7.734564	-1.630346

2, B3LYP/6-31+G(d,p), benzene IEFPCM:
Sum of electronic and thermal Free Energies= -1423.068071

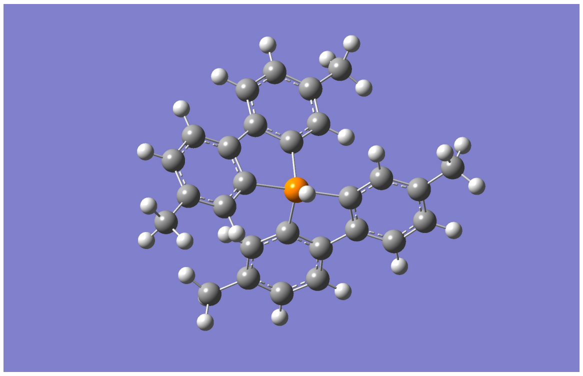

Center Number	Atomic Number	Atomic Type	Coordinates (Angstroms) X	Y	Z
1	6	0	-2.373962	1.390650	0.199894
2	6	0	-1.923626	0.212582	0.809752
3	6	0	-2.814167	-0.564339	1.550380
4	6	0	-4.170020	-0.211993	1.662037
5	6	0	-4.604307	0.964253	1.025631
6	6	0	-3.719928	1.768682	0.304898
7	1	0	-2.463305	-1.465806	2.054385
8	1	0	-5.647096	1.261156	1.110842
9	1	0	-4.081560	2.687028	-0.150346
10	6	0	-1.289855	2.138362	-0.466826
11	6	0	-1.433578	3.297916	-1.239285
12	6	0	-0.002879	1.592725	-0.284870
13	6	0	-0.316209	3.891721	-1.826207
14	1	0	-2.416044	3.733942	-1.395619
15	6	0	1.106375	2.191793	-0.886322
16	6	0	0.969522	3.351318	-1.664582
17	1	0	-0.445282	4.788858	-2.427581
18	1	0	2.091461	1.757840	-0.754107
19	6	0	1.286837	-2.132898	-0.499478
20	6	0	1.425866	-3.283577	-1.285965
21	6	0	0.305060	-3.870313	-1.873430
22	6	0	-0.979595	-3.331487	-1.698492
23	6	0	-1.111777	-2.181134	-0.905956
24	6	0	0.001016	-1.589264	-0.303904
25	1	0	2.407259	-3.718185	-1.452689
26	1	0	0.430558	-4.760573	-2.485679
27	1	0	-2.096023	-1.748676	-0.762958
28	6	0	2.374868	-1.393024	0.169615
29	6	0	1.928166	-0.222185	0.795801

30	6	0	3.721443	-1.772117	0.262320
31	6	0	2.823047	0.546176	1.540133
32	6	0	4.610027	-0.976108	0.987189
33	1	0	4.080502	-2.684957	-0.205834
34	6	0	4.179503	0.192640	1.639800
35	1	0	2.475035	1.441769	2.056407
36	1	0	5.653313	-1.273893	1.062702
37	15	0	0.001954	-0.004156	0.688753
38	6	0	-2.186127	-3.984612	-2.333262
39	1	0	-2.429497	-4.933945	-1.840056
40	1	0	-3.067632	-3.340891	-2.265846
41	1	0	-2.009291	-4.206353	-3.391481
42	6	0	5.156355	1.053584	2.408053
43	1	0	4.650804	1.630245	3.188878
44	1	0	5.936126	0.448765	2.882125
45	1	0	5.658814	1.770604	1.745882
46	6	0	2.172391	4.012387	-2.298055
47	1	0	2.420538	4.953881	-1.792330
48	1	0	3.053524	3.366686	-2.246587
49	1	0	1.988318	4.250275	-3.351507
50	6	0	-5.142316	-1.081963	2.425908
51	1	0	-4.632688	-1.664914	3.199394
52	1	0	-5.921454	-0.483166	2.908555
53	1	0	-5.645848	-1.793813	1.758985
54	1	0	0.006208	-0.012800	2.126881

3, B3LYP/6-31+G(d,p), benzene IEFPCM:
Sum of electronic and thermal Free Energies= -875.241712

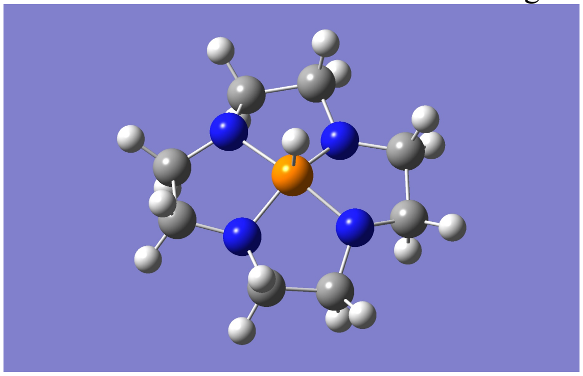

Center Number	Atomic Number	Atomic Type	Coordinates (Angstroms) X	Y	Z
1	6	0	2.918580	3.079743	-0.425959
2	6	0	3.277131	4.525892	-0.054445
3	6	0	1.892044	6.637129	0.186712

4	6	0	0.394319	6.928276	0.011958
5	1	0	3.708015	2.383426	-0.114298
6	1	0	3.783315	4.567883	0.925958
7	1	0	2.235582	6.925678	1.195666
8	1	0	2.491835	7.190799	-0.545324
9	1	0	0.164920	7.039387	-1.064309
10	6	0	-0.578015	1.973676	0.152759
11	6	0	0.864184	1.713840	-0.307046
12	6	0	-1.663831	5.566657	0.122845
13	6	0	-1.962736	4.083623	0.387386
14	1	0	-1.300589	1.492641	-0.517069
15	1	0	-0.744759	1.580442	1.171047
16	1	0	1.219217	0.734421	0.039407
17	1	0	-2.377543	6.213774	0.649279
18	1	0	-2.758805	3.716007	-0.270849
19	15	0	0.694736	4.281669	0.596339
20	1	0	0.815218	4.202602	2.000345
21	7	0	-0.708781	3.415969	0.102328
22	7	0	-0.299452	5.782387	0.577461
23	7	0	1.999641	5.209176	-0.033677
24	7	0	1.656916	2.802578	0.241493
25	1	0	0.903049	1.712400	-1.412409
26	1	0	-2.285513	3.926042	1.431616
27	1	0	-1.759105	5.773760	-0.959499
28	1	0	0.112260	7.864495	0.511093
29	1	0	3.946417	4.972696	-0.799195
30	1	0	2.812529	2.997783	-1.523955

4, B3LYP/6-31+G(d,p), benzene IEFPCM:
Sum of electronic and thermal Free Energies= -1114.382851

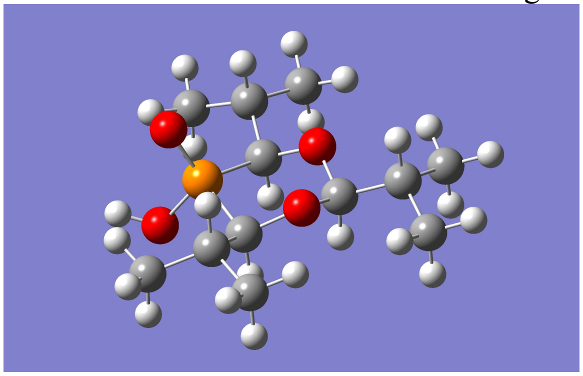

Center Number	Atomic Number	Atomic Type	Coordinates (Angstroms) X	Y	Z
1	6	0	-1.395659	-0.955654	-0.159917

2	6	0	-1.445617	1.569961	1.242162
3	6	0	-3.332595	0.145950	0.762316
4	1	0	-1.277457	-1.534384	0.772759
5	1	0	-1.331578	1.088011	2.228793
6	1	0	-3.088176	-0.373655	1.707950
7	15	0	-0.388819	0.600739	0.048607
8	8	0	-2.809585	1.462527	0.811878
9	8	0	-2.770181	-0.578059	-0.318316
10	8	0	0.038382	1.303848	-1.205549
11	8	0	0.856609	0.099940	0.999682
12	1	0	1.715519	0.312900	0.604800
13	6	0	-1.092660	3.063562	1.352292
14	6	0	0.366207	3.286515	1.783546
15	6	0	-2.054669	3.766686	2.324339
16	1	0	-1.228075	3.494978	0.352758
17	1	0	1.079719	2.921364	1.040246
18	1	0	0.550161	4.357849	1.912477
19	1	0	0.580645	2.793633	2.739266
20	1	0	-3.095910	3.644732	2.018830
21	1	0	-1.944964	3.366838	3.340546
22	1	0	-1.831293	4.838197	2.361410
23	6	0	-4.847158	0.230278	0.558541
24	6	0	-5.507916	1.039569	1.684767
25	6	0	-5.466633	-1.171181	0.450410
26	1	0	-4.997383	0.758247	-0.392261
27	1	0	-5.113537	2.056823	1.735908
28	1	0	-6.588653	1.098526	1.519473
29	1	0	-5.348060	0.561035	2.659542
30	1	0	-5.045141	-1.735758	-0.384163
31	1	0	-5.302675	-1.746365	1.370656
32	1	0	-6.548131	-1.091813	0.299223
33	6	0	-0.998154	-1.827281	-1.364413
34	6	0	-1.902489	-3.068746	-1.441785
35	6	0	0.482870	-2.238849	-1.325051
36	1	0	-1.163543	-1.217776	-2.261378
37	1	0	-2.958452	-2.794262	-1.488838
38	1	0	-1.660332	-3.653374	-2.335679
39	1	0	-1.753598	-3.716338	-0.568315
40	1	0	1.153544	-1.381506	-1.429292
41	1	0	0.729795	-2.761255	-0.393279
42	1	0	0.695707	-2.920041	-2.155112

5, B3LYP/6-31+G(d,p), chloroform IEFPCM:
Sum of electronic and thermal Free Energies= -2108.595736

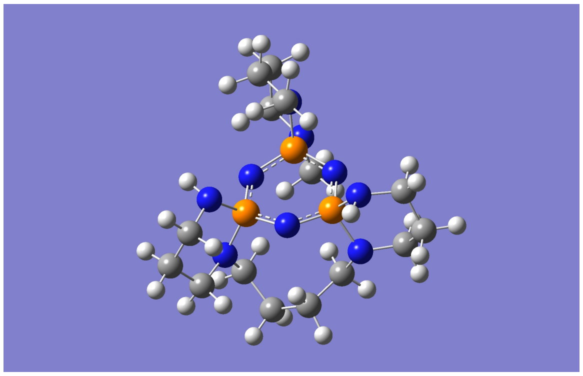

Center Number	Atomic Number	Atomic Type	Coordinates (Angstroms) X	Y	Z
1	15	0	9.528601	9.259623	5.963473
2	15	0	10.127408	8.134901	3.430641
3	15	0	7.477103	8.928031	4.010912
4	7	0	10.588873	8.776920	4.853107
5	7	0	8.549761	8.066774	3.140468
6	7	0	8.025843	9.566324	5.383901
7	7	0	9.482519	8.159842	7.268300
8	7	0	10.135756	10.636733	6.746177
9	7	0	10.762204	6.568510	3.332451
10	1	0	10.283606	6.049289	2.599227
11	7	0	10.912423	8.945727	2.143814
12	7	0	6.750677	10.161419	3.086855
13	7	0	6.174954	7.896240	4.353475
14	1	0	5.676868	8.261227	5.162923
15	6	0	9.084692	6.794133	6.902902
16	1	0	9.337168	6.124669	7.731878
17	1	0	9.636189	6.466828	6.019121
18	1	0	8.006406	6.705541	6.698269
19	6	0	8.816935	8.630575	8.496902
20	1	0	8.916856	7.835163	9.244256
21	1	0	7.735934	8.782928	8.330688
22	6	0	9.460263	9.911811	9.022379
23	1	0	10.495359	9.701632	9.313715
24	1	0	8.919735	10.243315	9.916552
25	6	0	9.449646	11.036749	7.989750
26	1	0	8.416219	11.364181	7.784400
27	1	0	9.987915	11.903976	8.387898
28	6	0	10.466120	11.776772	5.886208
29	1	0	9.575141	12.329935	5.549487
30	1	0	11.022253	11.429781	5.014226
31	1	0	11.105871	12.464876	6.448645
32	6	0	12.232855	6.478383	3.203496

33	1	0	12.499254	5.421016	3.111142
34	1	0	12.670133	6.841339	4.140158
35	6	0	12.776133	7.286832	2.017654
36	1	0	13.870636	7.214420	1.994308
37	1	0	12.396607	6.859789	1.080444
38	6	0	12.381794	8.766643	2.090535
39	1	0	12.876886	9.243578	2.953483
40	1	0	12.738783	9.279198	1.190635
41	6	0	10.441000	10.329546	1.907744
42	1	0	11.251039	10.869807	1.406790
43	1	0	10.282977	10.852203	2.862241
44	6	0	9.161077	10.383641	1.042224
45	1	0	9.446589	10.492608	-0.011440
46	1	0	8.649076	9.423778	1.130013
47	6	0	8.180877	11.508527	1.413226
48	1	0	7.406508	11.561885	0.637727
49	1	0	8.689390	12.483512	1.389355
50	6	0	7.501543	11.396396	2.797505
51	1	0	8.233882	11.521739	3.598501
52	1	0	6.799320	12.237671	2.892160
53	6	0	5.727523	9.803168	2.084796
54	1	0	6.183671	9.345917	1.191444
55	1	0	5.259274	10.738965	1.756623
56	6	0	4.647086	8.866247	2.633995
57	1	0	3.963709	8.603723	1.816849
58	1	0	4.060206	9.390827	3.398969
59	6	0	5.248600	7.595633	3.243095
60	1	0	4.466101	6.931958	3.624232
61	1	0	5.805378	7.034026	2.484201

6, B3LYP/6-31+G(d,p), chloroform IEFPCM:
Sum of electronic and thermal Free Energies= -923.021783

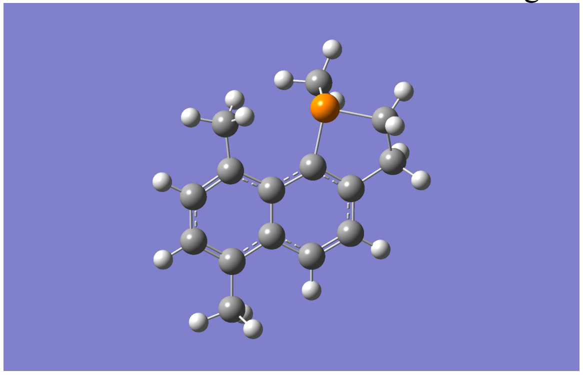

Center Number	Atomic Number	Atomic Type	Coordinates (Angstroms) X	Y	Z
1	6	0	-4.330765	-0.898942	-0.160401
2	6	0	-2.951258	-0.936525	-0.261289
3	6	0	-2.212176	0.297766	-0.136015
4	6	0	-2.966455	1.518829	0.074012
5	6	0	-4.399305	1.502945	0.166732
6	6	0	-5.050780	0.294688	0.051796
7	1	0	-4.885695	-1.829016	-0.252337
8	6	0	-0.775580	0.407963	-0.204696
9	6	0	-2.275180	2.756549	0.186601
10	1	0	-6.134837	0.255090	0.121313
11	6	0	-0.904921	2.827611	0.107432
12	6	0	-0.151689	1.650381	-0.086005
13	1	0	-2.843637	3.667434	0.334295
14	1	0	-0.399219	3.786011	0.194478
15	6	0	-2.317636	-2.286037	-0.517010
16	1	0	-1.763688	-2.312058	-1.459107
17	1	0	-1.618774	-2.581715	0.271759
18	1	0	-3.096253	-3.052865	-0.563078
19	6	0	-5.190491	2.771997	0.388800
20	1	0	-4.915712	3.268418	1.327161
21	1	0	-5.036479	3.498910	-0.417638
22	1	0	-6.259466	2.547711	0.432961
23	6	0	1.361652	1.667265	-0.131687
24	1	0	1.754197	1.759998	0.890758
25	1	0	1.731116	2.541350	-0.680856
26	6	0	1.822853	0.351054	-0.775509
27	1	0	2.798413	0.010241	-0.415051
28	1	0	1.895490	0.462014	-1.863080
29	15	0	0.506345	-0.938606	-0.443850
30	6	0	0.887693	-1.355829	1.346821
31	1	0	1.793611	-1.970295	1.376109
32	1	0	1.035356	-0.467453	1.969006
33	1	0	0.068633	-1.944159	1.770742

7, B3LYP/6-31+G(d,p), chloroform IEFPCM:
Sum of electronic and thermal Free Energies= -2480.376348

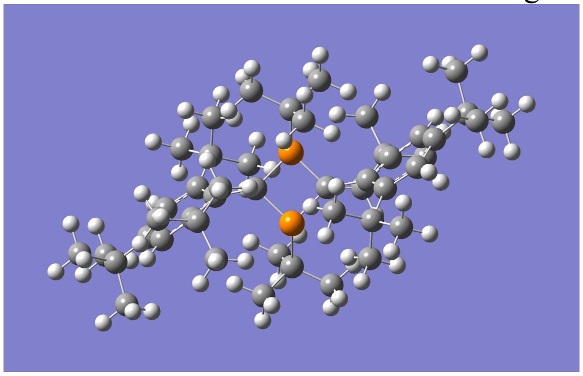

Center Number	Atomic Number	Atomic Type	Coordinates (Angstroms) X	Y	Z
1	6	0	-0.049514	11.584763	5.731342
2	6	0	-0.180938	10.161333	5.318157
3	6	0	1.020939	9.438913	4.978477
4	6	0	0.931482	8.375223	4.065622
5	1	0	1.851522	7.912069	3.731344
6	6	0	-0.271638	7.870859	3.581871
7	6	0	-1.429365	8.408962	4.150254
8	1	0	-2.374507	7.964614	3.884052
9	6	0	-1.439991	9.510945	5.016959
10	6	0	2.459365	9.660176	5.561261
11	6	0	2.495692	10.420429	6.897783
12	1	0	1.875472	9.919208	7.647242
13	1	0	2.152271	11.445197	6.802796
14	1	0	3.525479	10.436097	7.273643
15	6	0	3.386332	10.375482	4.550754
16	1	0	3.057774	11.401216	4.367115
17	1	0	3.413767	9.848445	3.590860
18	1	0	4.410057	10.413920	4.943306
19	6	0	3.094266	8.273079	5.882223
20	1	0	4.037973	8.427216	6.416781
21	1	0	3.326124	7.683831	4.991786
22	1	0	2.435721	7.675817	6.522041
23	6	0	-0.288139	6.735511	2.540358
24	6	0	0.480858	7.188234	1.274785
25	1	0	1.520164	7.445116	1.501917
26	1	0	0.008361	8.067763	0.823458
27	1	0	0.490023	6.386028	0.527278
28	6	0	0.397609	5.476135	3.125246
29	1	0	-0.127378	5.127310	4.021390
30	1	0	1.438522	5.672341	3.400518

31	1	0	0.393144	4.663066	2.389409
32	6	0	-1.718382	6.348136	2.115568
33	1	0	-1.673769	5.565355	1.350539
34	1	0	-2.260448	7.199542	1.689810
35	1	0	-2.302325	5.954752	2.954583
36	6	0	-2.830125	9.894821	5.621766
37	6	0	-3.923081	8.835591	5.326680
38	1	0	-3.641358	7.837055	5.676937
39	1	0	-4.179083	8.773619	4.264152
40	1	0	-4.835291	9.126678	5.858422
41	6	0	-2.712110	9.943629	7.159084
42	1	0	-2.010666	10.707636	7.480332
43	1	0	-2.369689	8.981126	7.555245
44	1	0	-3.684425	10.174941	7.610263
45	6	0	-3.388267	11.232617	5.084858
46	1	0	-4.333671	11.464176	5.590527
47	1	0	-3.593440	11.161425	4.012064
48	1	0	-2.708414	12.064885	5.250489
49	6	0	0.142132	12.954693	2.853835
50	6	0	1.137534	12.008416	2.149624
51	1	0	0.913140	11.999430	1.075179
52	1	0	1.059088	10.981592	2.516260
53	1	0	2.172621	12.341137	2.272992
54	6	0	-1.287904	12.452533	2.603074
55	1	0	-2.039053	13.102062	3.054646
56	1	0	-1.429827	11.439585	2.982512
57	1	0	-1.466743	12.435980	1.519082
58	6	0	0.307952	14.371194	2.273566
59	1	0	-0.273152	15.120860	2.814582
60	1	0	-0.048314	14.357957	1.235673
61	1	0	1.352333	14.689761	2.253734
62	15	0	0.602248	12.919399	4.742211
63	6	0	0.049621	14.114392	5.938094
64	6	0	0.181012	15.537812	6.351338
65	6	0	-1.020899	16.260070	6.691250
66	6	0	-0.931482	17.323624	7.604267
67	1	0	-1.851549	17.786609	7.938709
68	6	0	0.271616	17.828006	8.088045
69	6	0	1.429361	17.290039	7.519575
70	1	0	2.374491	17.734344	7.785894
71	6	0	1.440035	16.188204	6.652679
72	6	0	-2.459408	16.038578	6.108775
73	6	0	-2.495886	15.278365	4.772233
74	1	0	-1.875956	15.779758	4.022648
75	1	0	-2.152207	14.253673	4.867107
76	1	0	-3.525757	15.262475	4.396615

77	6	0	-3.386008	15.323067	7.119475
78	1	0	-3.057224	14.297383	7.302991
79	1	0	-3.413294	15.850058	8.079398
80	1	0	-4.409824	15.284470	6.727173
81	6	0	-3.094638	17.425573	5.788038
82	1	0	-4.038429	17.271295	5.253667
83	1	0	-3.326424	18.014716	6.678564
84	1	0	-2.436340	18.023004	5.148123
85	6	0	0.288073	18.963142	9.129791
86	6	0	-0.480856	18.510101	10.395292
87	1	0	-1.520151	18.253197	10.168135
88	1	0	-0.008285	17.630509	10.846418
89	1	0	-0.490052	19.312149	11.142970
90	6	0	-0.397779	20.222595	8.545190
91	1	0	0.127159	20.571650	7.649106
92	1	0	-1.438686	20.026378	8.269904
93	1	0	-0.393350	21.035506	9.281202
94	6	0	1.718302	19.350527	9.554619
95	1	0	1.673657	20.133125	10.319834
96	1	0	2.260448	18.499062	9.980157
97	1	0	2.302183	19.744153	8.715675
98	6	0	2.830253	15.804358	6.048055
99	6	0	3.923166	16.863562	6.343398
100	1	0	3.641562	17.862100	5.993052
101	1	0	4.178897	16.925546	7.405991
102	1	0	4.835503	16.572441	5.811894
103	6	0	2.712492	15.755648	4.510717
104	1	0	2.011100	14.991665	4.189306
105	1	0	2.370146	16.718177	4.114555
106	1	0	3.684882	15.524355	4.059689
107	6	0	3.388288	14.466529	6.584990
108	1	0	4.333774	14.234978	6.079468
109	1	0	3.593283	14.537666	7.657822
110	1	0	2.708448	13.634280	6.419209
111	6	0	-0.142127	12.744434	8.815563
112	6	0	-1.137478	13.690800	9.519725
113	1	0	-0.913132	13.699777	10.594179
114	1	0	-1.058930	14.717613	9.153080
115	1	0	-2.172589	13.358167	9.396312
116	6	0	1.287953	13.246461	9.066365
117	1	0	2.039050	12.596865	8.614801
118	1	0	1.429983	14.259398	8.686937
119	1	0	1.466769	13.262989	10.150361
120	6	0	-0.308106	11.327936	9.395802
121	1	0	0.272966	10.578225	8.854811
122	1	0	0.048093	11.341121	10.433719

123	1	0	-1.352517	11.009460	9.415555
124	15	0	-0.602161	12.779759	6.927182

8, B3LYP/6-31+G(d,p), chloroform IEFPCM:
Sum of electronic and thermal Free Energies= -1381.638169

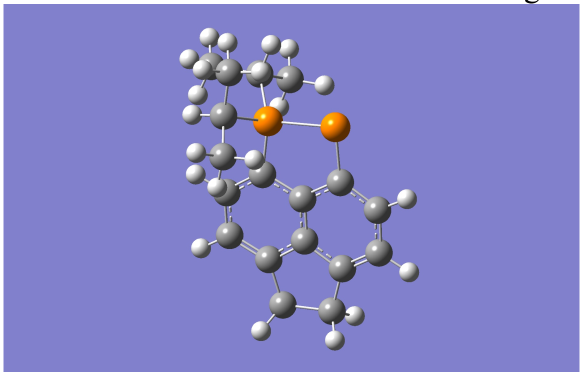

Center Number	Atomic Number	Atomic Type	Coordinates (Angstroms) X	Y	Z
1	15	0	8.550398	0.088368	11.999237
2	15	0	9.216769	-1.676686	10.917776
3	6	0	6.800986	0.136315	11.494795
4	6	0	5.780502	1.015884	11.847109
5	6	0	4.463616	0.870495	11.322835
6	6	0	4.188782	-0.167621	10.447000
7	6	0	5.226137	-1.056023	10.102763
8	6	0	4.825076	-2.078232	9.215133
9	6	0	5.784842	-2.997288	8.835997
10	6	0	7.119203	-2.893957	9.338468
11	6	0	7.526387	-1.885366	10.217511
12	6	0	6.529131	-0.935331	10.603910
13	6	0	2.927845	-0.606571	9.719146
14	6	0	3.349434	-1.883193	8.899123
15	6	0	8.664703	-0.013559	13.865717
16	6	0	7.809269	-1.168253	14.406363
17	6	0	10.131052	-0.137853	14.313841
18	6	0	9.427537	1.673311	11.500543
19	6	0	9.072425	2.893527	12.366482
20	6	0	9.201718	1.959362	10.007691
21	1	0	5.968378	1.832700	12.537682
22	1	0	3.696809	1.579402	11.623203
23	1	0	5.549796	-3.813070	8.156067
24	1	0	7.839544	-3.641823	9.015172
25	1	0	2.120483	-0.832142	10.424968
26	1	0	2.554833	0.186593	9.061231

27	1	0	2.752457	-2.754087	9.193717
28	1	0	3.178867	-1.735545	7.826417
29	1	0	8.251339	0.932798	14.236279
30	1	0	7.878896	-1.188359	15.499633
31	1	0	8.163007	-2.130165	14.022222
32	1	0	6.755392	-1.058340	14.135709
33	1	0	10.581598	-1.055977	13.921076
34	1	0	10.176372	-0.185643	15.407207
35	1	0	10.744339	0.710060	13.994369
36	1	0	10.488346	1.429739	11.645640
37	1	0	8.011143	3.150230	12.283282
38	1	0	9.644507	3.759643	12.015558
39	1	0	9.311922	2.749874	13.423302
40	1	0	9.848688	2.785903	9.694439
41	1	0	8.164078	2.250698	9.814021
42	1	0	9.436393	1.086884	9.391866

9, B3LYP/6-31+G(d,p), chloroform IEFPCM:
Sum of electronic and thermal Free Energies= -3370.931411

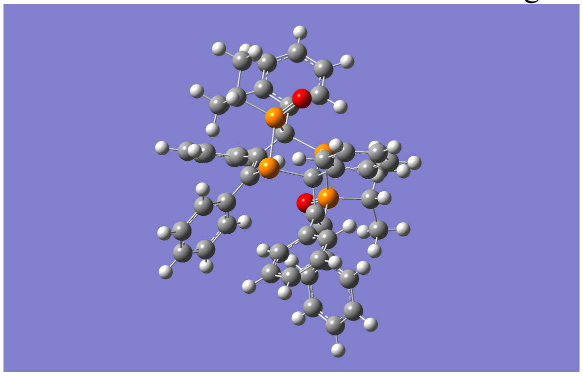

Center Number	Atomic Number	Atomic Type	Coordinates (Angstroms) X	Y	Z
1	15	0	0.990898	15.172085	4.161554
2	15	0	-0.336051	16.937197	3.746209
3	8	0	0.203708	18.181461	3.066839
4	6	0	-0.859151	17.176657	5.488992
5	6	0	-0.240696	16.422232	6.430260
6	6	0	0.809397	15.352764	6.059820
7	15	0	2.519750	15.912405	6.812209
8	15	0	3.622823	14.683293	5.297421
9	8	0	3.299507	13.210596	5.200325
10	6	0	2.837797	15.763482	3.910800
11	6	0	3.094247	17.181408	4.423111
12	6	0	2.887173	17.390005	5.762397

13	6	0	-1.764274	16.203912	2.797238
14	1	0	-2.158897	15.392167	3.422858
15	6	0	-2.869844	17.249693	2.561713
16	1	0	-3.683058	16.788923	1.989740
17	1	0	-2.484508	18.094370	1.982620
18	1	0	-3.288194	17.635421	3.493287
19	6	0	-1.281896	15.618193	1.458019
20	1	0	-2.139504	15.214487	0.908895
21	1	0	-0.564040	14.804830	1.593128
22	1	0	-0.818647	16.390341	0.835272
23	6	0	-1.914089	18.193063	5.770448
24	6	0	-3.110590	17.825937	6.412221
25	1	0	-3.256409	16.796680	6.723452
26	6	0	-4.113312	18.768269	6.645755
27	1	0	-5.030337	18.464335	7.142418
28	6	0	-3.939762	20.095780	6.240774
29	1	0	-4.719646	20.829324	6.423377
30	6	0	-2.757708	20.470197	5.595603
31	1	0	-2.614084	21.497745	5.273934
32	6	0	-1.754797	19.527265	5.356483
33	1	0	-0.845500	19.820657	4.843966
34	6	0	-0.514752	16.648828	7.891781
35	6	0	-1.230237	15.733625	8.681852
36	1	0	-1.610834	14.819018	8.242890
37	6	0	-1.478466	15.999607	10.030854
38	1	0	-2.041138	15.282083	10.621208
39	6	0	-1.010747	17.178796	10.617161
40	1	0	-1.202780	17.381609	11.666795
41	6	0	-0.300313	18.097383	9.840020
42	1	0	0.063916	19.021108	10.280034
43	6	0	-0.059597	17.837006	8.489136
44	1	0	0.484247	18.561571	7.892329
45	6	0	0.380868	13.983641	6.640115
46	6	0	-0.728121	13.323213	6.082167
47	1	0	-1.247868	13.758145	5.234023
48	6	0	-1.185815	12.110030	6.596443
49	1	0	-2.044246	11.624943	6.140747
50	6	0	-0.540068	11.521202	7.688386
51	1	0	-0.889992	10.574376	8.088882
52	6	0	0.560813	12.164862	8.253979
53	1	0	1.075729	11.724272	9.102735
54	6	0	1.011652	13.384031	7.737474
55	1	0	1.859679	13.863937	8.213415
56	6	0	5.459454	15.002995	5.461474
57	1	0	5.660087	15.988333	5.029128
58	6	0	6.241083	13.924510	4.689215

59	1	0	7.312275	14.143615	4.761173
60	1	0	5.971369	13.886927	3.631946
61	1	0	6.062573	12.935512	5.119724
62	6	0	5.874658	15.020092	6.945434
63	1	0	6.961155	15.143778	7.011389
64	1	0	5.616487	14.079639	7.443699
65	1	0	5.411093	15.839593	7.500701
66	6	0	3.386146	15.362270	2.538428
67	6	0	4.677428	15.757203	2.136137
68	1	0	5.274549	16.397303	2.772399
69	6	0	5.218613	15.350575	0.916395
70	1	0	6.215994	15.681719	0.641836
71	6	0	4.487549	14.527725	0.055710
72	1	0	4.906595	14.214350	-0.895844
73	6	0	3.213083	14.112547	0.441384
74	1	0	2.627747	13.465621	-0.205509
75	6	0	2.675132	14.521717	1.664968
76	1	0	1.690177	14.164969	1.939211
77	6	0	3.670825	18.241645	3.538666
78	6	0	4.855619	18.888873	3.938856
79	1	0	5.308747	18.634330	4.891764
80	6	0	5.460321	19.849450	3.127346
81	1	0	6.376489	20.331504	3.456469
82	6	0	4.887354	20.188129	1.897534
83	1	0	5.353567	20.937358	1.264091
84	6	0	3.710054	19.553590	1.490781
85	1	0	3.253075	19.812097	0.539746
86	6	0	3.111543	18.582983	2.296836
87	1	0	2.185043	18.118512	1.985526
88	6	0	2.951192	18.712101	6.442203
89	6	0	3.628537	18.870538	7.665123
90	1	0	4.129643	18.019733	8.117383
91	6	0	3.674538	20.110513	8.306247
92	1	0	4.213430	20.209740	9.244198
93	6	0	3.027875	21.216253	7.746307
94	1	0	3.058545	22.179961	8.246186
95	6	0	2.333724	21.068080	6.540769
96	1	0	1.816776	21.916749	6.101876
97	6	0	2.294456	19.831434	5.895626
98	1	0	1.749760	19.722878	4.963507

10, B3LYP/6-31+G(d,p), chloroform IEFPCM:
Sum of electronic and thermal Free Energies= -1950.234043

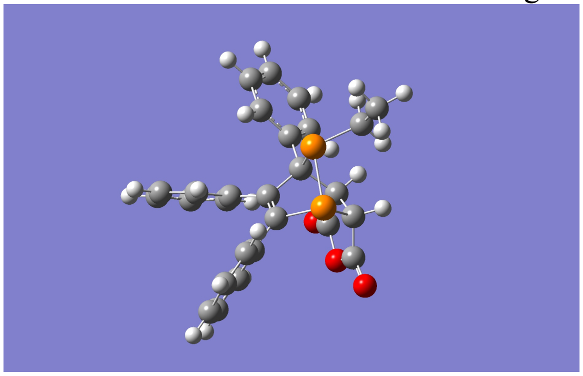

(mirror image of coordinates below shown for clarity)

Center Number	Atomic Number	Atomic Type	Coordinates (Angstroms) X	Y	Z
1	15	0	13.090320	8.791447	14.940301
2	15	0	13.064013	10.592626	13.620313
3	8	0	14.349022	10.096365	18.274292
4	8	0	14.702266	7.917834	17.808374
5	8	0	14.194419	12.334575	18.213699
6	6	0	13.083271	11.584634	15.284206
7	6	0	11.807536	10.999015	15.938017
8	6	0	11.722470	9.636966	15.898533
9	6	0	14.517337	9.548143	15.980950
10	6	0	14.543426	9.034317	17.393267
11	6	0	14.279005	11.304324	17.599250
12	6	0	14.346834	11.072101	16.092614
13	6	0	13.074790	13.078356	14.974781
14	6	0	14.063647	13.964937	15.427908
15	1	0	14.849323	13.626963	16.091851
16	6	0	14.041097	15.318104	15.070687
17	1	0	14.819267	15.978192	15.442809
18	6	0	13.026755	15.817869	14.254331
19	1	0	13.008601	16.868255	13.979315
20	6	0	12.029913	14.948816	13.798484
21	1	0	11.228703	15.319153	13.165553
22	6	0	12.056794	13.600738	14.152472
23	1	0	11.273976	12.945153	13.785256
24	6	0	10.741058	11.875871	16.502527
25	6	0	11.015896	12.912094	17.412169
26	1	0	12.033037	13.092975	17.738871
27	6	0	9.986186	13.711069	17.914105
28	1	0	10.220468	14.499283	18.623756
29	6	0	8.666100	13.505377	17.503813

30	1	0	7.868327	14.132884	17.890357
31	6	0	8.382224	12.489907	16.585252
32	1	0	7.362386	12.325721	16.249335
33	6	0	9.408395	11.683281	16.092563
34	1	0	9.179002	10.900648	15.376718
35	6	0	10.713669	8.782157	16.573802
36	6	0	10.380722	8.976142	17.927374
37	1	0	10.858226	9.773871	18.486693
38	6	0	9.451442	8.149339	18.559653
39	1	0	9.214375	8.313212	19.606867
40	6	0	8.833193	7.112566	17.853330
41	1	0	8.109441	6.470329	18.346411
42	6	0	9.161780	6.902167	16.510688
43	1	0	8.691920	6.097105	15.953159
44	6	0	10.100192	7.722105	15.881111
45	1	0	10.350806	7.545121	14.838895
46	6	0	14.791349	10.765541	12.912950
47	1	0	14.903823	11.831670	12.680200
48	1	0	15.576389	10.512450	13.632424
49	6	0	14.955568	9.928688	11.633392
50	1	0	14.861988	8.856842	11.837578
51	1	0	15.447945	9.244960	15.493330
52	1	0	15.234147	11.593545	15.723574
53	1	0	15.944229	10.097770	11.194250
54	1	0	14.204816	10.194606	10.882417

----

11 isomer A, B3LYP/6-31+G(d,p), toluene IEFPCM:
Sum of electronic and thermal Free Energies= -1091.671654

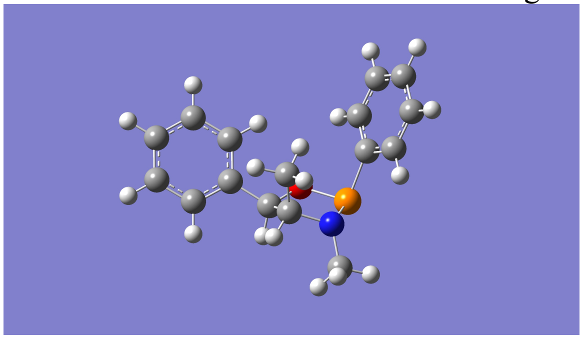

Center Number	Atomic Number	Atomic Type	Coordinates (Angstroms) X	Y	Z
1	6	0	0.507295	1.516348	0.776568
2	6	0	1.238918	0.971776	-0.494520
3	1	0	1.102341	2.340778	1.185287

4	1	0	1.655244	1.840842	-1.025277
5	15	0	-1.271284	1.183921	-1.130262
6	8	0	0.219918	0.387939	-1.332486
7	7	0	-0.760297	2.089399	0.258037
8	6	0	-0.777324	3.554407	0.134573
9	1	0	-1.700732	3.869989	-0.357043
10	1	0	-0.758086	4.004378	1.132850
11	1	0	0.070839	3.955051	-0.443184
12	6	0	2.359889	-0.012759	-0.249530
13	6	0	2.152363	-1.397912	-0.275956
14	6	0	3.643116	0.474688	0.038789
15	6	0	3.205050	-2.276635	-0.006023
16	1	0	1.167239	-1.780785	-0.518285
17	6	0	4.694664	-0.402256	0.316078
18	1	0	3.823770	1.547371	0.041526
19	6	0	4.478062	-1.783399	0.295096
20	1	0	3.030352	-3.348579	-0.034357
21	1	0	5.682324	-0.007631	0.537315
22	1	0	5.295357	-2.468025	0.502671
23	6	0	0.274539	0.486751	1.885088
24	1	0	-0.262804	-0.391322	1.520219
25	1	0	1.227052	0.153727	2.307751
26	1	0	-0.320968	0.941727	2.681874
27	6	0	-2.242401	-0.212633	-0.388339
28	6	0	-3.218569	0.043700	0.586551
29	6	0	-2.129814	-1.510351	-0.912770
30	6	0	-4.046590	-0.982730	1.049636
31	1	0	-3.316174	1.043491	1.001059
32	6	0	-2.957362	-2.537058	-0.449144
33	1	0	-1.384908	-1.722876	-1.674651
34	6	0	-3.917525	-2.276012	0.533529
35	1	0	-4.788889	-0.773471	1.815090
36	1	0	-2.852368	-3.539468	-0.855154
37	1	0	-4.561571	-3.073644	0.892588

----

11 isomer B, B3LYP/6-31+G(d,p), toluene IEFPCM:
Sum of electronic and thermal Free Energies= -1091.670033

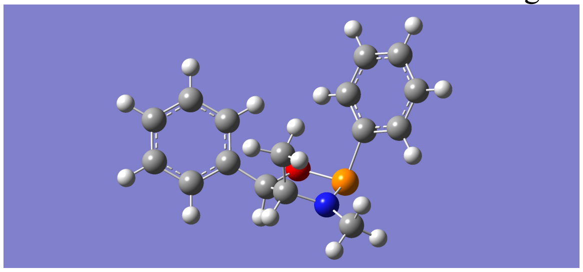

Center Number	Atomic Number	Atomic Type	Coordinates (Angstroms) X	Y	Z
1	6	0	0.461198	1.334976	1.011698
2	6	0	1.162796	0.939132	-0.318735
3	1	0	1.051429	2.137167	1.476332
4	1	0	1.544626	1.868216	-0.762465
5	15	0	-1.379396	1.145302	-0.898045
6	8	0	0.125756	0.432759	-1.194207
7	7	0	-0.780551	1.932015	0.511735
8	6	0	2.308234	-0.041713	-0.208775
9	6	0	2.134007	-1.424048	-0.348917
10	6	0	3.591218	0.452352	0.070607
11	6	0	3.217561	-2.294535	-0.201742
12	1	0	1.150790	-1.814812	-0.582386
13	6	0	4.673952	-0.416193	0.227455
14	1	0	3.746339	1.525145	0.160665
15	6	0	4.489769	-1.795464	0.091634
16	1	0	3.066647	-3.364100	-0.318223
17	1	0	5.660107	-0.015452	0.444167
18	1	0	5.330888	-2.473275	0.204965
19	6	0	0.272989	0.207490	2.036185
20	1	0	-0.250582	-0.649560	1.605821
21	1	0	1.239666	-0.134455	2.418532
22	1	0	-0.314474	0.572647	2.884874
23	6	0	-2.310486	-0.384753	-0.346479
24	6	0	-3.522588	-0.207491	0.341539
25	6	0	-1.932099	-1.683974	-0.716432
26	6	0	-4.313735	-1.304156	0.693541
27	1	0	-3.857594	0.792918	0.609403
28	6	0	-2.728695	-2.782585	-0.377807
29	1	0	-1.012450	-1.834341	-1.272806
30	6	0	-3.917082	-2.596623	0.334425
31	1	0	-5.240419	-1.150369	1.239680
32	1	0	-2.421587	-3.782812	-0.671654
33	1	0	-4.534705	-3.450136	0.598968
34	6	0	-1.564223	2.819943	1.350199
35	1	0	-2.324676	3.319047	0.739756
36	1	0	-2.077120	2.305338	2.176849
37	1	0	-0.917521	3.594981	1.780560

11 isomer C, B3LYP/6-31+G(d,p), toluene IEFPCM:
Sum of electronic and thermal Free Energies= -1091.667783

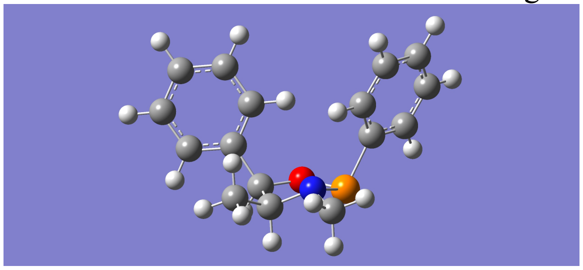

Center Number	Atomic Number	Atomic Type	Coordinates (Angstroms) X	Y	Z
1	6	0	0.316068	1.333361	0.408317
2	6	0	1.206788	0.116267	0.001846
3	1	0	1.906098	0.463509	-0.767289
4	15	0	-1.312896	-0.457771	-0.555644
5	8	0	0.330287	-0.839362	-0.638272
6	7	0	-1.022708	0.773835	0.665683
7	6	0	2.028867	-0.539252	1.096881
8	6	0	3.381572	-0.192739	1.228110
9	6	0	1.490483	-1.485813	1.978570
10	6	0	4.173953	-0.754571	2.232981
11	1	0	3.823618	0.518545	0.534022
12	6	0	2.280808	-2.053953	2.980738
13	1	0	0.457402	-1.792433	1.869761
14	6	0	3.623543	-1.687337	3.115959
15	1	0	5.220085	-0.473712	2.316331
16	1	0	1.847765	-2.790071	3.652365
17	1	0	4.237331	-2.133660	3.893139
18	1	0	0.275400	2.000561	-0.474670
19	6	0	0.851310	2.150286	1.583858
20	1	0	0.861558	1.558047	2.502797
21	1	0	1.872522	2.486227	1.377221
22	1	0	0.241036	3.043221	1.746665
23	6	0	-2.088260	1.771237	0.802736
24	1	0	-3.056134	1.268570	0.885732
25	1	0	-1.943865	2.352154	1.718137
26	1	0	-2.133587	2.469105	-0.050732
27	6	0	-2.043658	-1.816288	0.476661
28	6	0	-2.546951	-2.931451	-0.213778
29	6	0	-2.187295	-1.759061	1.871944
30	6	0	-3.145741	-3.987759	0.477514
31	1	0	-2.470051	-2.974938	-1.298354
32	6	0	-2.805117	-2.804853	2.562894
33	1	0	-1.813439	-0.890584	2.406096

34	6	0	-3.277569	-3.923544	1.868157
35	1	0	-3.518035	-4.851073	-0.066643
36	1	0	-2.916152	-2.748945	3.642466
37	1	0	-3.754174	-4.737367	2.407378

----

12 isomer A, B3LYP/6-31+G(d,p), toluene IEFPCM:
Sum of electronic and thermal Free Energies= -1091.671458

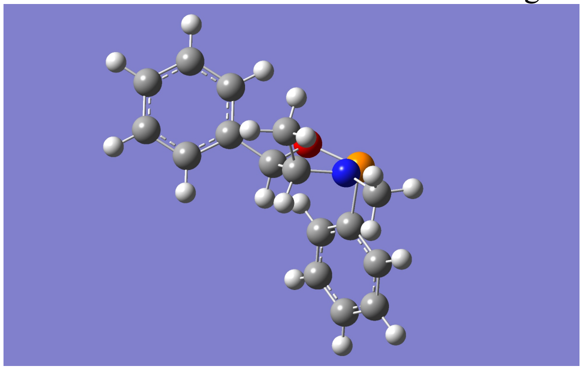

Center Number	Atomic Number	Atomic Type	Coordinates (Angstroms) X	Y	Z
1	6	0	-0.504386	1.432258	0.775857
2	6	0	-0.754610	0.029576	0.122651
3	1	0	-0.219640	1.256760	1.825806
4	1	0	-0.157630	-0.701683	0.680813
5	15	0	1.210642	0.964324	-1.236782
6	8	0	-0.220076	0.096545	-1.224761
7	7	0	0.637622	1.983684	0.026547
8	6	0	1.413907	3.063176	0.610557
9	1	0	2.130969	3.439938	-0.126230
10	1	0	0.764660	3.900139	0.891326
11	1	0	1.973712	2.752417	1.507610
12	6	0	2.352136	-0.290714	-0.428119
13	6	0	3.524011	0.147426	0.211144
14	6	0	2.144306	-1.672183	-0.572685
15	6	0	4.448704	-0.768853	0.720654
16	1	0	3.726142	1.211089	0.314531
17	6	0	3.069204	-2.590931	-0.067116
18	1	0	1.252385	-2.030126	-1.079939
19	6	0	4.222057	-2.141967	0.584220
20	1	0	5.345724	-0.411531	1.219039
21	1	0	2.889602	-3.656417	-0.183104
22	1	0	4.941391	-2.855535	0.975741
23	6	0	-2.186272	-0.452347	0.085393
24	6	0	-3.012188	-0.229317	-1.023983
25	6	0	-2.708539	-1.131878	1.195198

26	6	0	-4.339279	-0.667348	-1.016731
27	1	0	-2.607438	0.276201	-1.894048
28	6	0	-4.037588	-1.562718	1.207161
29	1	0	-2.071307	-1.329862	2.054132
30	6	0	-4.858139	-1.330651	0.099265
31	1	0	-4.967428	-0.492089	-1.885629
32	1	0	-4.426884	-2.087662	2.074872
33	1	0	-5.889617	-1.671000	0.102564
34	6	0	-1.698883	2.392859	0.742209
35	1	0	-2.051665	2.541219	-0.282484
36	1	0	-2.527646	2.009091	1.344904
37	1	0	-1.414356	3.367705	1.150023

----

12 isomer B, B3LYP/6-31+G(d,p), toluene IEFPCM:
Sum of electronic and thermal Free Energies= -1091.671506

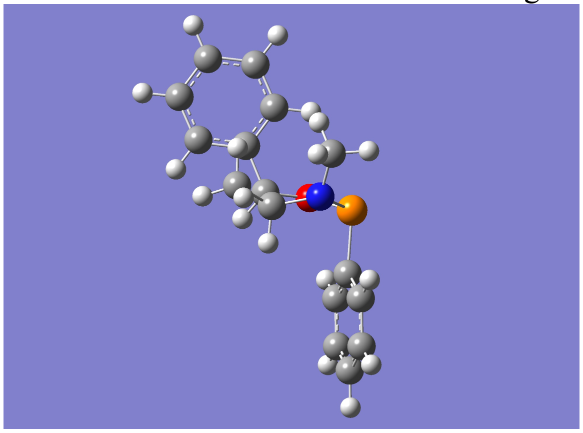

Center Number	Atomic Number	Atomic Type	Coordinates (Angstroms) X	Y	Z
1	6	0	-0.265144	0.695432	0.789356
2	6	0	-0.958629	-0.609498	0.222156
3	1	0	0.600407	0.333445	1.355837
4	1	0	-0.753698	-1.422314	0.929487
5	15	0	0.853628	0.245348	-1.479614
6	8	0	-0.285745	-0.924592	-1.017769
7	7	0	0.295228	1.434444	-0.367347
8	6	0	2.348193	-0.392347	-0.575096
9	6	0	3.351708	0.498223	-0.160690
10	6	0	2.562428	-1.771774	-0.428245
11	6	0	4.534265	0.020832	0.410673
12	1	0	3.201157	1.569268	-0.269054
13	6	0	3.746924	-2.249905	0.140875
14	1	0	1.795541	-2.471176	-0.749069
15	6	0	4.734884	-1.355219	0.562633

16	1	0	5.297356	0.721851	0.737763
17	1	0	3.896424	-3.320017	0.255896
18	1	0	5.655240	-1.726828	1.004090
19	6	0	-2.458425	-0.547097	-0.008259
20	6	0	-3.005693	-0.167634	-1.241352
21	6	0	-3.331191	-0.885153	1.036723
22	6	0	-4.390666	-0.100405	-1.416256
23	1	0	-2.344371	0.051892	-2.072781
24	6	0	-4.716228	-0.816294	0.866632
25	1	0	-2.925980	-1.213599	1.991097
26	6	0	-5.251775	-0.418809	-0.362028
27	1	0	-4.796612	0.191983	-2.380740
28	1	0	-5.374710	-1.084200	1.688131
29	1	0	-6.328045	-0.371252	-0.500413
30	6	0	-1.112069	1.543167	1.733024
31	1	0	-2.018532	1.924949	1.257417
32	1	0	-1.417591	0.943227	2.595589
33	1	0	-0.525465	2.387950	2.106280
34	6	0	-0.456395	2.575197	-0.916693
35	1	0	-0.459609	3.398479	-0.196475
36	1	0	0.054959	2.925775	-1.816214
37	1	0	-1.496060	2.341617	-1.181498

13, B3LYP/6-31+G(d,p), chloroform IEFPCM:
Sum of electronic and thermal Free Energies= -1792.906321

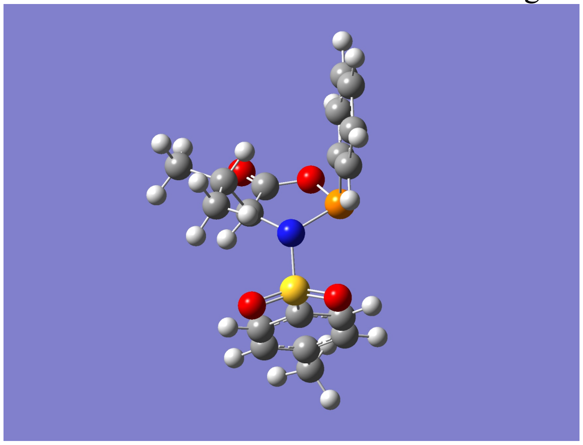

Center Number	Atomic Number	Atomic Type	Coordinates (Angstroms) X	Y	Z
1	15	0	0.763836	-1.236737	-0.512581
2	16	0	-0.957001	-0.177180	1.656280
3	7	0	0.306899	0.105594	0.544981
4	8	0	0.514517	-0.266625	-1.906418
5	8	0	0.038025	1.825018	-2.563050

6	8	0	-0.630951	-1.486012	2.240349
7	8	0	-1.045075	1.028984	2.488072
8	6	0	0.281360	1.058951	-1.662858
9	6	0	0.375940	1.402772	-0.167134
10	6	0	1.665805	2.226548	0.154540
11	6	0	1.892612	2.336292	1.668027
12	6	0	1.592542	3.623573	-0.483433
13	6	0	-6.150116	-0.682292	-1.505851
14	6	0	3.208654	-1.448079	0.825516
15	6	0	4.599694	-1.515255	0.923238
16	6	0	5.393583	-1.309640	-0.210501
17	6	0	4.792860	-1.037675	-1.442736
18	6	0	3.400202	-0.966444	-1.545808
19	6	0	2.602087	-1.157393	-0.407698
20	6	0	-3.303754	0.796812	0.577567
21	6	0	-4.485296	0.674787	-0.156054
22	6	0	-4.868194	-0.550530	-0.720818
23	6	0	-4.034479	-1.666079	-0.526515
24	6	0	-2.851024	-1.567244	0.200704
25	6	0	-2.491112	-0.327546	0.743758
26	1	0	-0.495522	2.010927	0.094060
27	1	0	2.517577	1.689187	-0.279584
28	1	0	2.807900	2.907322	1.855042
29	1	0	2.000745	1.355362	2.135418
30	1	0	1.060319	2.853385	2.156629
31	1	0	2.512995	4.173701	-0.263646
32	1	0	0.754542	4.196925	-0.068352
33	1	0	1.470590	3.583344	-1.567886
34	1	0	-6.648311	0.283186	-1.623182
35	1	0	-6.846152	-1.364542	-1.004479
36	1	0	-5.958671	-1.091904	-2.503578
37	1	0	2.600114	-1.609510	1.711609
38	1	0	5.062194	-1.727510	1.882520
39	1	0	6.475354	-1.364715	-0.133288
40	1	0	5.405382	-0.879860	-2.325504
41	1	0	2.940962	-0.753293	-2.505999
42	1	0	-3.029641	1.743779	1.028820
43	1	0	-5.120221	1.546706	-0.283582
44	1	0	-4.319050	-2.627641	-0.944815
45	1	0	-2.225209	-2.438342	0.357864

----

14 isomer A, B3LYP/6-31+G(d,p), chloroform IEFPCM:
Sum of electronic and thermal Free Energies= -1792.903243

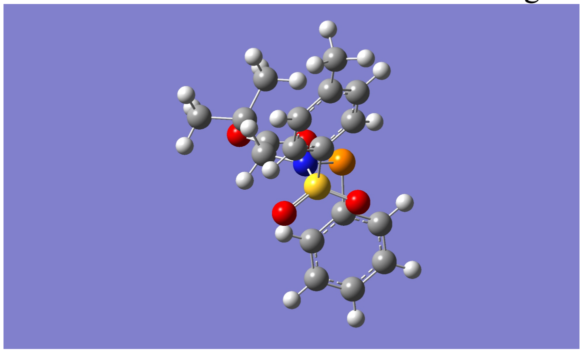

Center Number	Atomic Number	Atomic Type	Coordinates (Angstroms) X	Y	Z
1	15	0	-1.466964	-0.122292	-1.331186
2	16	0	0.417606	-0.966917	0.809231
3	7	0	-0.317115	0.282655	-0.030344
4	8	0	-1.824960	1.561702	-1.449745
5	8	0	-1.710638	3.524653	-0.375174
6	8	0	0.339311	-0.677791	2.248339
7	8	0	-0.219816	-2.167908	0.236301
8	6	0	-1.356857	2.373626	-0.466873
9	6	0	-0.328499	1.683793	0.435265
10	6	0	1.061963	2.384510	0.370564
11	6	0	1.056774	3.709524	1.148052
12	6	0	1.573084	2.561183	-1.065631
13	6	0	6.311928	-1.207937	-0.743332
14	6	0	2.503158	-1.364474	-0.952999
15	6	0	3.851194	-1.428623	-1.293176
16	6	0	4.856207	-1.141562	-0.351275
17	6	0	4.469624	-0.791402	0.950021
18	6	0	3.122030	-0.718466	1.311183
19	6	0	2.149884	-1.001531	0.351738
20	1	0	-0.698277	1.738124	1.466206
21	1	0	1.746561	1.708150	0.893815
22	1	0	2.071789	4.118473	1.181610
23	1	0	0.718932	3.565236	2.180264
24	1	0	0.405136	4.450373	0.677509
25	1	0	2.602250	2.933171	-1.052356
26	1	0	0.967299	3.287664	-1.619182
27	1	0	1.568298	1.616015	-1.617235
28	1	0	6.541008	-2.151220	-1.250094
29	1	0	6.965425	-1.119319	0.128123
30	1	0	6.564851	-0.398118	-1.437920
31	1	0	1.740899	-1.604082	-1.686906

32	1	0	4.129146	-1.712925	-2.304316
33	1	0	5.228738	-0.576944	1.696707
34	1	0	2.830824	-0.456731	2.322160
35	6	0	-2.990930	-0.684215	-0.469686
36	6	0	-3.814741	-1.549066	-1.207048
37	6	0	-3.378414	-0.302359	0.825621
38	6	0	-5.016929	-2.014949	-0.665695
39	1	0	-3.517004	-1.863517	-2.204580
40	6	0	-4.574542	-0.771188	1.366910
41	1	0	-2.745661	0.346097	1.423342
42	6	0	-5.396339	-1.625427	0.620470
43	1	0	-5.648335	-2.682831	-1.243736
44	1	0	-4.865409	-0.475406	2.370404
45	1	0	-6.326633	-1.989604	1.046268

----

14 isomer B, B3LYP/6-31+G(d,p), chloroform IEFPCM:
Sum of electronic and thermal Free Energies= -1792.901771

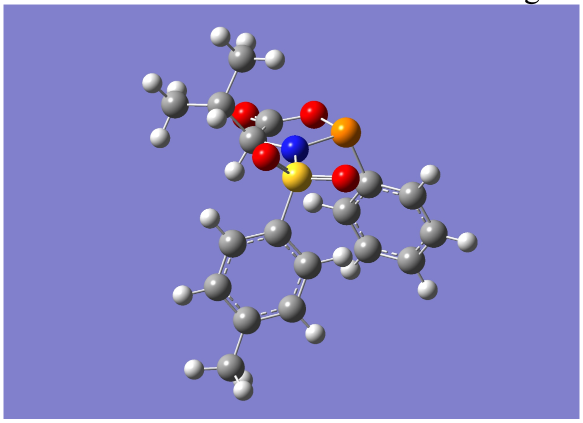

Center Number	Atomic Number	Atomic Type	Coordinates (Angstroms) X	Y	Z
1	15	0	-1.551596	0.170216	-1.649559
2	16	0	0.110746	-1.232260	0.405661
3	7	0	-0.600998	0.182019	-0.129922
4	8	0	-1.992340	1.796524	-1.310979
5	8	0	-1.991041	3.415636	0.237858
6	8	0	-0.213413	-2.189145	-0.669285
7	8	0	1.510764	-0.981265	0.774262
8	6	0	-1.598139	2.333191	-0.125613
9	6	0	-0.596589	1.449697	0.621994
10	6	0	0.805435	2.131307	0.723970
11	6	0	0.792539	3.289282	1.732965
12	6	0	1.349757	2.565469	-0.643649
13	6	0	-2.810952	-2.876365	5.503750

14	6	0	-0.226409	-1.335757	3.143459
15	6	0	-0.906500	-1.706834	4.304959
16	6	0	-2.092263	-2.453773	4.245858
17	6	0	-2.587544	-2.822954	2.982869
18	6	0	-1.928502	-2.457622	1.811476
19	6	0	-0.748523	-1.711259	1.903064
20	1	0	-0.992051	1.296161	1.634757
21	1	0	1.465823	1.351662	1.114764
22	1	0	1.812149	3.662778	1.872809
23	1	0	0.418446	2.965613	2.711063
24	1	0	0.169450	4.120245	1.391101
25	1	0	2.378786	2.922498	-0.537157
26	1	0	0.760735	3.384253	-1.073859
27	1	0	1.361612	1.734465	-1.355987
28	1	0	-2.674744	-3.949111	5.685840
29	1	0	-3.887828	-2.696143	5.423917
30	1	0	-2.436097	-2.338816	6.378433
31	1	0	0.703190	-0.780802	3.201554
32	1	0	-0.501763	-1.416626	5.270285
33	1	0	-3.500369	-3.408366	2.915304
34	1	0	-2.316290	-2.754996	0.843895
35	6	0	-3.144517	-0.649000	-1.220106
36	6	0	-3.478258	-1.773704	-1.991648
37	6	0	-4.040679	-0.199014	-0.235602
38	6	0	-4.679837	-2.453332	-1.770118
39	1	0	-2.796837	-2.122113	-2.763690
40	6	0	-5.243583	-0.871546	-0.021674
41	1	0	-3.813933	0.680299	0.359149
42	6	0	-5.562432	-2.001117	-0.786174
43	1	0	-4.926964	-3.325201	-2.368112
44	1	0	-5.934205	-0.514863	0.736676
45	1	0	-6.500803	-2.521354	-0.617762

----

15 isomer A (cis, chair), B3LYP/6-31+G(d,p), benzene IEFPCM:
Sum of electronic and thermal Free Energies= -901.378764

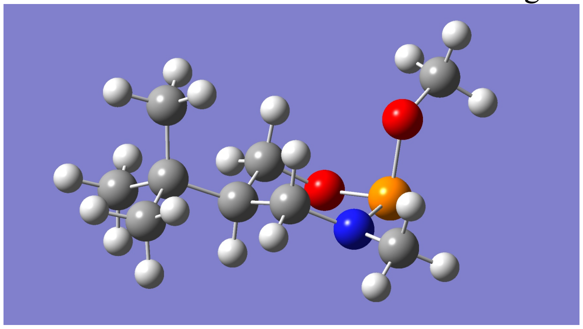

Center Number	Atomic Number	Atomic Type	Coordinates (Angstroms) X	Y	Z
1	6	0	0.289539	-1.224917	-0.558268
2	6	0	0.264961	1.132834	0.315839
3	6	0	1.095459	0.075338	-0.439678
4	1	0	0.050770	-1.638575	0.427679
5	1	0	0.822546	-1.982073	-1.134690
6	1	0	0.789105	2.093231	0.293079
7	1	0	0.146227	0.852178	1.374627
8	1	0	1.229654	0.454251	-1.462855
9	6	0	2.534975	-0.140148	0.149164
10	6	0	2.513333	-0.804933	1.543762
11	1	0	3.535046	-0.891222	1.931190
12	1	0	1.936561	-0.221546	2.268839
13	1	0	2.091955	-1.815047	1.512107
14	6	0	3.353279	-1.032123	-0.812880
15	1	0	3.378391	-0.607000	-1.823171
16	1	0	4.387427	-1.115181	-0.460174
17	1	0	2.954215	-2.048599	-0.883649
18	6	0	3.265953	1.216769	0.257828
19	1	0	4.315864	1.055334	0.526681
20	1	0	3.244962	1.758093	-0.695575
21	1	0	2.831505	1.863807	1.026052
22	15	0	-2.072042	0.060082	-0.707902
23	8	0	-0.946820	-1.012329	-1.282082
24	7	0	-1.047976	1.362770	-0.310008
25	6	0	-1.715611	2.580759	0.149301
26	1	0	-1.095741	3.453391	-0.088096
27	1	0	-2.674137	2.696097	-0.366857
28	1	0	-1.907774	2.575017	1.233405
29	8	0	-2.360695	-0.555128	0.845564
30	6	0	-3.327500	-1.604258	0.975383
31	1	0	-2.898766	-2.571654	0.688202
32	1	0	-3.626518	-1.642778	2.025581
33	1	0	-4.214352	-1.410850	0.357808

15 isomer B (cis, twist 1), B3LYP/6-31+G(d,p), benzene IEFPCM:
Sum of electronic and thermal Free Energies= -901.370816

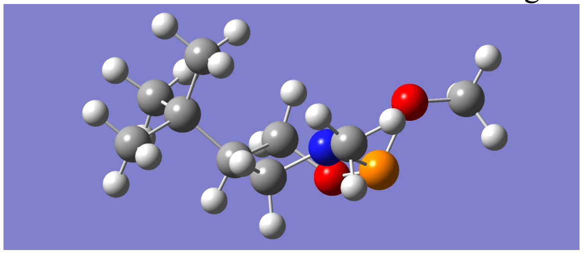

Center Number	Atomic Number	Atomic Type	Coordinates (Angstroms) X	Y	Z
1	6	0	-0.029823	-1.289106	-0.137612
2	6	0	0.177280	1.185385	0.320454
3	6	0	0.867327	-0.053173	-0.320747
4	1	0	0.077590	-1.724656	0.859954
5	1	0	0.189057	-2.056775	-0.881018
6	1	0	-0.295985	1.795401	-0.467649
7	1	0	0.922925	1.833289	0.789667
8	1	0	0.920884	0.139380	-1.400795
9	6	0	2.344788	-0.293866	0.146399
10	6	0	2.468067	-0.400799	1.680951
11	1	0	3.515935	-0.562925	1.959400
12	1	0	2.131442	0.511131	2.184032
13	1	0	1.886167	-1.235032	2.084715
14	6	0	2.885778	-1.593915	-0.490715
15	1	0	2.765758	-1.583916	-1.580814
16	1	0	3.954753	-1.703699	-0.275454
17	1	0	2.384061	-2.485968	-0.101782
18	6	0	3.226503	0.873083	-0.350245
19	1	0	4.269590	0.715079	-0.053319
20	1	0	3.197578	0.951652	-1.443479
21	1	0	2.913697	1.837688	0.063249
22	15	0	-2.189411	-0.034748	0.811914
23	8	0	-1.418278	-0.933816	-0.347798
24	7	0	-0.788716	0.822536	1.372852
25	6	0	-1.065920	1.901342	2.322839
26	1	0	-0.142435	2.170448	2.847928
27	1	0	-1.466621	2.810619	1.843889
28	1	0	-1.784831	1.559329	3.072068
29	8	0	-2.299634	-1.039203	2.149391
30	6	0	-3.519823	-1.773149	2.336458
31	1	0	-3.486720	-2.719578	1.785924
32	1	0	-3.609146	-1.981854	3.404867
33	1	0	-4.392829	-1.196829	2.006884

15 isomer C (cis, twist 2), B3LYP/6-31+G(d,p), benzene IEFPCM:
Sum of electronic and thermal Free Energies= -901.370902

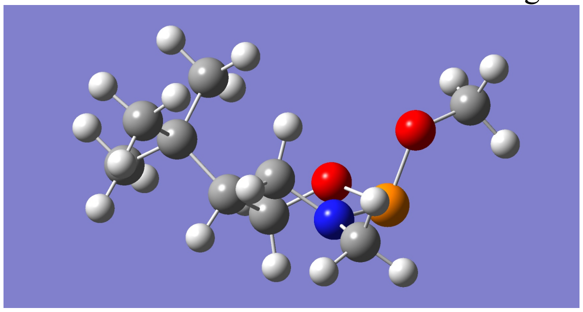

Center Number	Atomic Number	Atomic Type	Coordinates (Angstroms) X	Y	Z
1	6	0	0.694325	-1.280663	-1.322705
2	6	0	0.536030	1.170765	-0.726356
3	6	0	1.509632	-0.009266	-0.989616
4	1	0	1.207706	-2.191507	-1.009410
5	1	0	0.533304	-1.349797	-2.405026
6	1	0	1.019535	2.112872	-0.997715
7	1	0	0.281441	1.237196	0.339167
8	1	0	2.071411	0.238737	-1.901812
9	6	0	2.579301	-0.193411	0.138706
10	6	0	1.965872	-0.747096	1.442362
11	1	0	2.745021	-0.851795	2.206505
12	1	0	1.192302	-0.086915	1.846080
13	1	0	1.512088	-1.731895	1.292964
14	6	0	3.673995	-1.166423	-0.353489
15	1	0	4.153882	-0.791852	-1.265703
16	1	0	4.452136	-1.282361	0.409651
17	1	0	3.279602	-2.165352	-0.567529
18	6	0	3.259398	1.162340	0.433714
19	1	0	4.088086	1.024747	1.137573
20	1	0	3.671283	1.607326	-0.480561
21	1	0	2.568571	1.883592	0.881497
22	15	0	-1.754378	-0.222974	-1.159635
23	8	0	-0.580197	-1.309418	-0.648953
24	7	0	-0.687395	1.046869	-1.538676
25	6	0	-1.256093	2.287037	-2.061557
26	1	0	-2.141106	2.058358	-2.664411
27	1	0	-1.557051	2.984492	-1.265669
28	1	0	-0.527422	2.789646	-2.707762
29	8	0	-2.318982	0.233164	0.356866
30	6	0	-3.418322	-0.492416	0.922525
31	1	0	-3.061138	-1.387078	1.444316
32	1	0	-3.911759	0.168844	1.638641

33	1	0	-4.141467	-0.789818	0.152016

----

16 isomer A (trans, chair), B3LYP/6-31+G(d,p), benzene IEFPCM:
Sum of electronic and thermal Free Energies= -901.369257

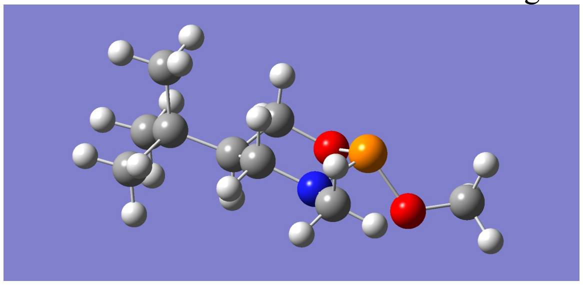

Center Number	Atomic Number	Atomic Type	Coordinates (Angstroms) X	Y	Z
1	6	0	0.478853	-1.352187	0.358630
2	6	0	0.443803	1.147202	0.152682
3	6	0	1.158570	-0.141727	-0.299425
4	1	0	0.562867	-1.306100	1.454286
5	1	0	0.910028	-2.297410	0.026649
6	1	0	0.866516	2.009243	-0.371572
7	1	0	0.615480	1.320337	1.231564
8	1	0	0.980591	-0.237938	-1.378940
9	6	0	2.716117	-0.110195	-0.102401
10	6	0	3.131299	-0.049595	1.384361
11	1	0	4.224018	-0.017017	1.464620
12	1	0	2.740677	0.842255	1.885198
13	1	0	2.790803	-0.927405	1.943645
14	6	0	3.337022	-1.375470	-0.738551
15	1	0	3.038331	-1.479488	-1.788236
16	1	0	4.430585	-1.313638	-0.706791
17	1	0	3.050610	-2.291982	-0.213472
18	6	0	3.308755	1.115117	-0.834393
19	1	0	4.403365	1.065609	-0.822496
20	1	0	2.987200	1.145974	-1.882291
21	1	0	3.023148	2.061189	-0.364133
22	15	0	-1.891640	-0.181035	0.533789
23	8	0	-0.910875	-1.417475	-0.017844
24	7	0	-0.999619	1.141927	-0.162770
25	6	0	-1.614891	2.454606	0.061243
26	1	0	-1.132061	3.190431	-0.591443
27	1	0	-2.674162	2.418554	-0.204602
28	1	0	-1.523607	2.808917	1.102593
29	6	0	-4.313753	-0.917542	-0.279226
30	1	0	-4.575615	-0.785473	0.776933

31	1	0	-5.096169	-0.481607	-0.903645
32	1	0	-4.226524	-1.986013	-0.500726
33	8	0	-3.089252	-0.239568	-0.608828

----

16 isomer B (trans, twist 1), B3LYP/6-31+G(d,p), benzene IEFPCM:
Sum of electronic and thermal Free Energies= -901.374270

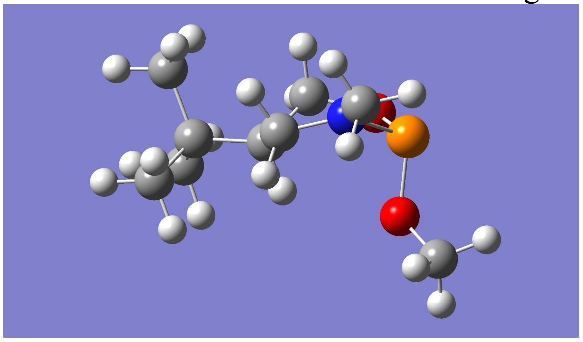

Center Number	Atomic Number	Atomic Type	Coordinates (Angstroms) X	Y	Z
1	6	0	0.671735	-1.631004	1.054929
2	6	0	0.494595	0.863263	1.066920
3	6	0	0.972709	-0.358789	0.252502
4	1	0	1.079613	-1.559787	2.070549
5	1	0	1.082617	-2.522612	0.578453
6	1	0	0.328113	1.721512	0.399523
7	1	0	1.268221	1.163358	1.788828
8	1	0	0.360485	-0.414295	-0.655927
9	6	0	2.463387	-0.249649	-0.221217
10	6	0	3.461973	-0.448399	0.939951
11	1	0	4.487696	-0.310059	0.579651
12	1	0	3.301126	0.270082	1.751019
13	1	0	3.398355	-1.455796	1.364973
14	6	0	2.734826	-1.320592	-1.301519
15	1	0	2.057771	-1.199452	-2.154795
16	1	0	3.761819	-1.230855	-1.673290
17	1	0	2.615093	-2.339467	-0.919917
18	6	0	2.707269	1.134458	-0.861984
19	1	0	3.711984	1.174622	-1.297691
20	1	0	1.987295	1.333284	-1.664570
21	1	0	2.635921	1.947768	-0.132692
22	15	0	-1.824223	-0.655008	1.416872
23	8	0	-0.748035	-1.884525	1.152353
24	7	0	-0.727501	0.581596	1.833272
25	6	0	-3.407242	0.310655	-0.507080
26	1	0	-3.253799	1.395051	-0.437662

27	1	0	-3.698282	0.060651	-1.530180
28	1	0	-4.219900	0.023088	0.173194
29	8	0	-2.201511	-0.404545	-0.221263
30	6	0	-1.196021	1.698805	2.646513
31	1	0	-0.434968	1.970881	3.389578
32	1	0	-1.413550	2.592112	2.040572
33	1	0	-2.106892	1.415178	3.181467

----

16 isomer C (trans, twist 2), B3LYP/6-31+G(d,p), benzene IEFPCM:
Sum of electronic and thermal Free Energies= -901.373778

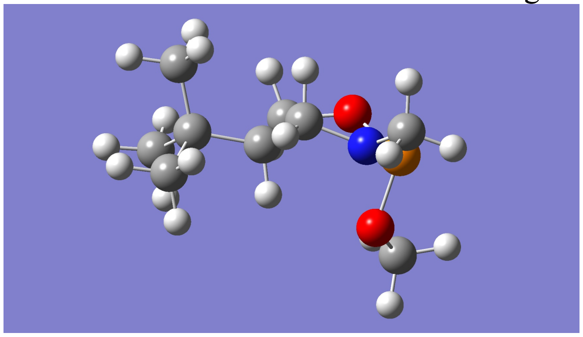

Center Number	Atomic Number	Atomic Type	Coordinates (Angstroms) X	Y	Z
1	6	0	0.104625	-1.262257	0.501664
2	6	0	0.342631	1.132380	-0.233822
3	6	0	0.872537	-0.300600	-0.425190
4	1	0	0.514363	-1.245410	1.516438
5	1	0	0.156756	-2.287899	0.125678
6	1	0	0.943017	1.838345	-0.818799
7	1	0	0.431035	1.433002	0.823975
8	1	0	0.636710	-0.584566	-1.457545
9	6	0	2.429898	-0.425049	-0.274204
10	6	0	2.946051	0.189649	1.044758
11	1	0	4.027571	0.034185	1.130221
12	1	0	2.766031	1.268738	1.089617
13	1	0	2.480212	-0.266035	1.925251
14	6	0	2.839121	-1.914477	-0.326358
15	1	0	2.452012	-2.402029	-1.229003
16	1	0	3.931211	-2.003550	-0.341925
17	1	0	2.480107	-2.474816	0.543042
18	6	0	3.116987	0.289899	-1.459058
19	1	0	4.204082	0.166644	-1.395083
20	1	0	2.788238	-0.129166	-2.416992
21	1	0	2.912363	1.365152	-1.473592
22	15	0	-2.148629	-0.016621	-0.432395

23	8	0	-1.299667	-0.930192	0.665696
24	7	0	-1.050197	1.261025	-0.677241
25	6	0	-2.705226	-2.019542	-2.091995
26	1	0	-3.755270	-1.810376	-1.848708
27	1	0	-2.628643	-2.267169	-3.153111
28	1	0	-2.367260	-2.876739	-1.497506
29	8	0	-1.874545	-0.873344	-1.865119
30	6	0	-1.565237	2.626779	-0.672352
31	1	0	-2.626014	2.629577	-0.937593
32	1	0	-1.456383	3.109702	0.312071
33	1	0	-1.031376	3.235858	-1.412903

----

17 isomer A (cis, chair), B3LYP/6-31+G(d,p), benzene IEFPCM:
Sum of electronic and thermal Free Energies= -920.765886

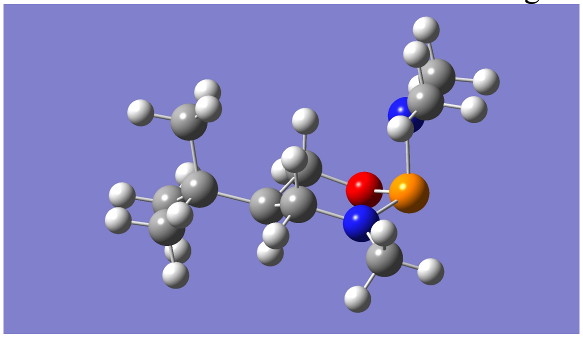

Center Number	Atomic Number	Atomic Type	Coordinates (Angstroms) X	Y	Z
1	6	0	0.488213	-1.166529	-0.732980
2	6	0	0.415215	1.119119	0.349104
3	6	0	1.287712	0.113595	-0.438329
4	1	0	0.192820	-1.666447	0.194666
5	1	0	1.062943	-1.863827	-1.344609
6	1	0	0.949301	2.069402	0.436806
7	1	0	0.230515	0.755680	1.371642
8	1	0	1.496793	0.583757	-1.410009
9	6	0	2.681323	-0.174608	0.226480
10	6	0	2.558143	-0.979889	1.539321
11	1	0	3.549548	-1.118903	1.985861
12	1	0	1.934255	-0.467692	2.279166
13	1	0	2.133579	-1.975126	1.372241
14	6	0	3.567300	-0.968357	-0.761220
15	1	0	3.667759	-0.439423	-1.716326
16	1	0	4.572365	-1.098725	-0.344269
17	1	0	3.172104	-1.967361	-0.969100
18	6	0	3.404139	1.157025	0.529908

19	1	0	4.432890	0.959886	0.851735
20	1	0	3.450558	1.798176	-0.358732
21	1	0	2.917470	1.720915	1.331784
22	15	0	-1.849434	0.150890	-0.906944
23	8	0	-0.700964	-0.891212	-1.512362
24	7	0	-0.847358	1.406550	-0.345574
25	6	0	-1.348460	2.771922	-0.259262
26	1	0	-0.606389	3.479242	-0.652310
27	1	0	-2.258750	2.868116	-0.858897
28	1	0	-1.590294	3.071924	0.772697
29	7	0	-2.203996	-0.735466	0.592977
30	6	0	-2.893472	-2.008172	0.360117
31	1	0	-2.905258	-2.591178	1.288996
32	1	0	-3.940053	-1.879941	0.029004
33	1	0	-2.363204	-2.589828	-0.397969
34	6	0	-2.880197	0.059734	1.617453
35	1	0	-2.929869	-0.518410	2.548031
36	1	0	-2.317822	0.973992	1.823096
37	1	0	-3.914020	0.341601	1.344684

----

17 isomer B (cis, twist 1), B3LYP/6-31+G(d,p), benzene IEFPCM:
Sum of electronic and thermal Free Energies= -920.766061

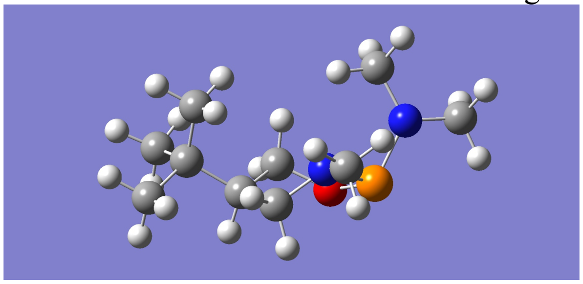

Center Number	Atomic Number	Atomic Type	Coordinates (Angstroms) X	Y	Z
1	6	0	0.136795	-1.451970	-0.070853
2	6	0	0.154539	1.033809	0.372864
3	6	0	0.919699	-0.145711	-0.292478
4	1	0	0.326548	-1.868227	0.923384
5	1	0	0.409565	-2.205499	-0.812394
6	1	0	-0.383325	1.610003	-0.400352
7	1	0	0.860574	1.735406	0.826428
8	1	0	0.910149	0.041340	-1.374785
9	6	0	2.431446	-0.254457	0.108659
10	6	0	2.631500	-0.353598	1.635949
11	1	0	3.700041	-0.430030	1.868749
12	1	0	2.243366	0.525994	2.159312

13	1	0	2.139478	-1.235824	2.057633
14	6	0	3.057797	-1.500797	-0.557288
15	1	0	2.894052	-1.497023	-1.641629
16	1	0	4.139923	-1.518545	-0.384378
17	1	0	2.648770	-2.433905	-0.156184
18	6	0	3.185693	0.986127	-0.418144
19	1	0	4.252156	0.915841	-0.174710
20	1	0	3.095629	1.068519	-1.507810
21	1	0	2.813243	1.917972	0.020267
22	15	0	-2.147328	-0.300531	0.852221
23	8	0	-1.275464	-1.229703	-0.248161
24	7	0	-0.760418	0.599292	1.445679
25	6	0	-1.110836	1.689310	2.360448
26	1	0	-0.202446	2.058327	2.850719
27	1	0	-1.596747	2.541781	1.855158
28	1	0	-1.787834	1.320587	3.135225
29	7	0	-2.477975	-1.216145	2.255328
30	6	0	-1.486902	-1.844151	3.125875
31	1	0	-1.830691	-1.805250	4.168110
32	1	0	-1.324583	-2.901299	2.864062
33	1	0	-0.541308	-1.305065	3.053539
34	6	0	-3.814692	-1.795184	2.373285
35	1	0	-4.180867	-1.693508	3.403382
36	1	0	-4.507710	-1.272335	1.708238
37	1	0	-3.825480	-2.864317	2.111720

----

17 isomer C (cis, twist 2), B3LYP/6-31+G(d,p), benzene IEFPCM:
Sum of electronic and thermal Free Energies= -920.763725

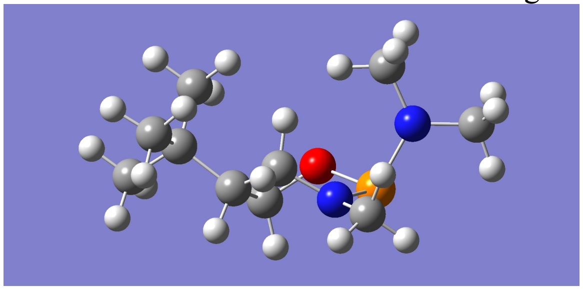

Center Number	Atomic Number	Atomic Type	Coordinates (Angstroms) X	Y	Z
1	6	0	0.687407	-1.358765	-0.997552
2	6	0	0.583826	1.131277	-0.584936
3	6	0	1.528184	-0.061764	-0.883883
4	1	0	1.225704	-2.230452	-0.619601
5	1	0	0.441060	-1.557848	-2.048304
6	1	0	1.044752	2.055961	-0.945276

7	1	0	0.447509	1.256185	0.499046
8	1	0	1.952808	0.120017	-1.881386
9	6	0	2.748411	-0.160383	0.093074
10	6	0	2.331001	-0.615475	1.507252
11	1	0	3.212051	-0.678890	2.156641
12	1	0	1.630882	0.086065	1.972232
13	1	0	1.855789	-1.601593	1.497204
14	6	0	3.772468	-1.167127	-0.476761
15	1	0	4.118089	-0.857297	-1.470268
16	1	0	4.649613	-1.231381	0.177435
17	1	0	3.359524	-2.177490	-0.565568
18	6	0	3.448706	1.213084	0.193420
19	1	0	4.369629	1.127204	0.781425
20	1	0	3.722213	1.593003	-0.798530
21	1	0	2.818696	1.963299	0.682099
22	15	0	-1.759634	-0.342182	-0.876385
23	8	0	-0.526644	-1.294576	-0.226795
24	7	0	-0.698483	0.974537	-1.280853
25	6	0	-1.324944	2.194971	-1.770793
26	1	0	-2.226207	1.941197	-2.340355
27	1	0	-1.621355	2.885706	-0.963632
28	1	0	-0.643839	2.730376	-2.443645
29	7	0	-2.595408	-0.054030	0.586234
30	6	0	-1.992876	0.320313	1.860748
31	1	0	-0.937729	0.046604	1.867788
32	1	0	-2.089842	1.398548	2.061631
33	1	0	-2.484612	-0.220886	2.680523
34	6	0	-4.050248	0.038679	0.551161
35	1	0	-4.497643	-0.615955	1.312287
36	1	0	-4.400513	1.065251	0.738987
37	1	0	-4.420214	-0.274388	-0.429283

----

18 isomer A (trans, chair), B3LYP/6-31+G(d,p), benzene IEFPCM:
Sum of electronic and thermal Free Energies= -920.768892

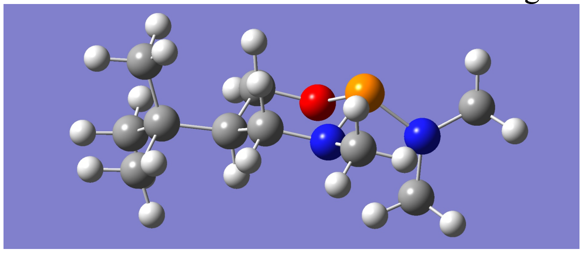

Center Number	Atomic Number	Atomic Type	Coordinates (Angstroms) X	Y	Z
1	6	0	-0.719527	-1.341483	-0.456006

2	6	0	-0.687608	1.150511	-0.205028
3	6	0	-1.376600	-0.148560	0.256378
4	1	0	-0.863989	-1.274110	-1.545154
5	1	0	-1.129587	-2.295774	-0.120702
6	1	0	-1.101300	2.005019	0.339124
7	1	0	-0.896997	1.329254	-1.277009
8	1	0	-1.148652	-0.262987	1.325229
9	6	0	-2.941667	-0.121091	0.136131
10	6	0	-3.429195	-0.009921	-1.325713
11	1	0	-4.524902	0.012971	-1.352931
12	1	0	-3.071126	0.904604	-1.809508
13	1	0	-3.106479	-0.862011	-1.933216
14	6	0	-3.525410	-1.412129	0.755545
15	1	0	-3.172250	-1.553839	1.783915
16	1	0	-4.619405	-1.354487	0.782876
17	1	0	-3.263593	-2.306681	0.182126
18	6	0	-3.504900	1.073099	0.939576
19	1	0	-4.598654	1.017481	0.978838
20	1	0	-3.133405	1.067482	1.971431
21	1	0	-3.246233	2.037546	0.491567
22	15	0	1.635901	-0.141967	-0.767198
23	8	0	0.684709	-1.400007	-0.156812
24	7	0	0.765958	1.152051	0.057710
25	6	0	1.345892	2.477525	-0.182788
26	1	0	0.874850	3.202762	0.490830
27	1	0	2.416900	2.459370	0.034139
28	1	0	1.207239	2.835390	-1.218451
29	7	0	3.015171	-0.240388	0.205180
30	6	0	4.255082	-0.730477	-0.391347
31	1	0	4.202963	-0.650985	-1.480514
32	1	0	5.105324	-0.130513	-0.041600
33	1	0	4.448018	-1.781770	-0.129401
34	6	0	2.973503	-0.318177	1.664468
35	1	0	2.028619	0.090666	2.024589
36	1	0	3.071819	-1.356132	2.013876
37	1	0	3.797472	0.268098	2.091815

----

18 isomer B (trans, twist 1), B3LYP/6-31+G(d,p), benzene IEFPCM:
Sum of electronic and thermal Free Energies= -920.762439

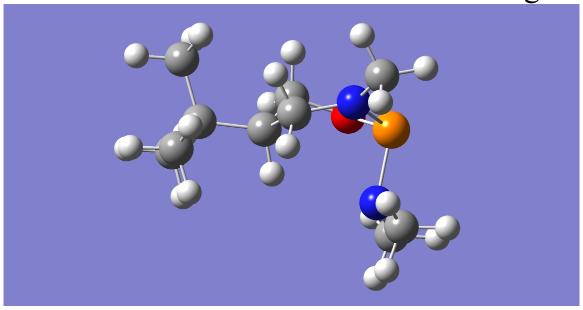

Center Number	Atomic Number	Atomic Type	Coordinates (Angstroms) X	Y	Z
1	6	0	0.763011	-1.620574	1.012235
2	6	0	0.543895	0.880056	0.922544
3	6	0	1.097158	-0.365978	0.190809
4	1	0	1.112107	-1.511879	2.046644
5	1	0	1.223576	-2.514851	0.587990
6	1	0	0.399619	1.705791	0.210055
7	1	0	1.271512	1.227139	1.670521
8	1	0	0.553117	-0.459001	-0.755445
9	6	0	2.618415	-0.245458	-0.173287
10	6	0	3.532854	-0.387021	1.063137
11	1	0	4.579683	-0.241085	0.773219
12	1	0	3.301544	0.354391	1.835695
13	1	0	3.455476	-1.380740	1.516930
14	6	0	2.989058	-1.344926	-1.194086
15	1	0	2.370984	-1.269153	-2.096163
16	1	0	4.037236	-1.242212	-1.497454
17	1	0	2.866927	-2.353148	-0.785873
18	6	0	2.887714	1.120790	-0.842355
19	1	0	3.923073	1.166654	-1.198713
20	1	0	2.229893	1.276102	-1.705623
21	1	0	2.743730	1.957489	-0.151311
22	15	0	-1.752886	-0.665081	1.223893
23	8	0	-0.653035	-1.912023	1.043643
24	7	0	-0.704195	0.603197	1.645170
25	6	0	-2.630933	-1.482319	-1.219284
26	1	0	-3.671941	-1.693963	-0.912739
27	1	0	-2.637309	-1.269584	-2.295090
28	1	0	-2.031283	-2.379636	-1.052881
29	6	0	-1.126530	1.639314	2.577324
30	1	0	-0.349624	1.815555	3.334022
31	1	0	-1.327722	2.596094	2.070197
32	1	0	-2.040338	1.327514	3.090579

33	7	0	-2.038975	-0.345715	-0.505594
34	6	0	-2.814408	0.878426	-0.722115
35	1	0	-3.856892	0.810915	-0.359453
36	1	0	-2.332293	1.725939	-0.226434
37	1	0	-2.850711	1.095963	-1.796038

----

18 isomer C (trans, twist 2), B3LYP/6-31+G(d,p), benzene IEFPCM:
Sum of electronic and thermal Free Energies= -920.765909

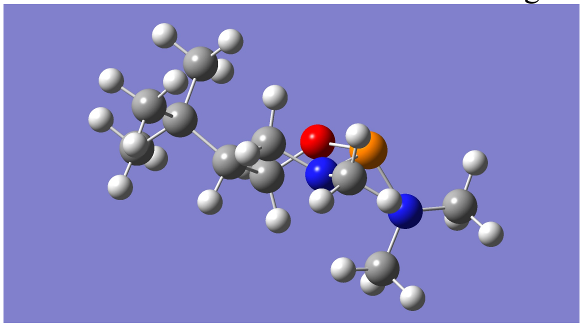

Center Number	Atomic Number	Atomic Type	Coordinates (Angstroms) X	Y	Z
1	6	0	0.792276	-1.444564	-0.739973
2	6	0	0.658094	1.072649	-0.772118
3	6	0	1.605522	-0.140607	-0.918193
4	1	0	1.394002	-2.249377	-0.311880
5	1	0	0.423849	-1.790268	-1.710573
6	1	0	1.057468	1.914949	-1.344281
7	1	0	0.609028	1.408111	0.278256
8	1	0	1.957294	-0.141369	-1.960273
9	6	0	2.890566	-0.041109	-0.028217
10	6	0	2.581999	-0.247291	1.470003
11	1	0	3.505657	-0.169464	2.055479
12	1	0	1.883499	0.503312	1.852657
13	1	0	2.146133	-1.232151	1.664938
14	6	0	3.905536	-1.113641	-0.482459
15	1	0	4.174687	-0.981256	-1.537398
16	1	0	4.825683	-1.041924	0.108679
17	1	0	3.519834	-2.131149	-0.359609
18	6	0	3.553602	1.340948	-0.221641
19	1	0	4.513685	1.376320	0.305683
20	1	0	3.748923	1.543718	-1.281862
21	1	0	2.936318	2.154769	0.171859
22	15	0	-1.611923	-0.273821	-0.245367
23	8	0	-0.319328	-1.271770	0.160975
24	7	0	-0.686658	0.779265	-1.304574
25	6	0	-3.816882	-1.676129	-0.952043

26	1	0	-4.082438	-1.300588	0.040193
27	1	0	-4.647767	-1.461427	-1.637051
28	1	0	-3.702416	-2.768976	-0.888019
29	6	0	-1.414303	1.983113	-1.717492
30	1	0	-2.399520	1.705826	-2.101856
31	1	0	-1.556402	2.708714	-0.898245
32	1	0	-0.864473	2.482122	-2.523475
33	7	0	-2.595788	-1.024671	-1.422069
34	6	0	-2.182525	-1.447091	-2.758270
35	1	0	-1.927150	-2.517684	-2.787284
36	1	0	-3.001385	-1.278264	-3.470377
37	1	0	-1.319790	-0.860739	-3.077432

19, B3LYP/6-31+G(d,p), chloroform IEFPCM:
Sum of electronic and thermal Free Energies= -2126.548780

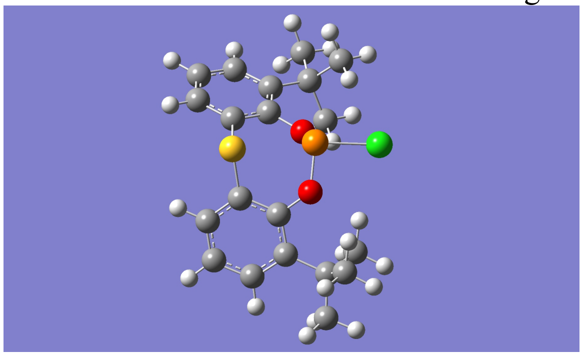

Center Number	Atomic Number	Atomic Type	Coordinates (Angstroms) X	Y	Z
1	15	0	0.080360	-1.296009	-1.677303
2	8	0	-0.900487	-0.954002	-0.350259
3	8	0	1.569745	-0.821996	-1.084387
4	16	0	-0.159000	1.477526	-1.942060
5	6	0	-1.855362	0.044430	-0.281681
6	6	0	-1.624668	1.273623	-0.924624
7	6	0	-2.553770	2.312123	-0.831917
8	1	0	-2.365138	3.251489	-1.340784
9	6	0	-3.709288	2.117211	-0.081414
10	6	0	-3.918774	0.899240	0.568630
11	1	0	-4.824866	0.785859	1.149495
12	6	0	-3.013812	-0.174081	0.498533
13	6	0	1.911166	0.371403	-0.476430
14	6	0	1.175569	1.540493	-0.743725
15	6	0	1.530254	2.756281	-0.150133
16	1	0	0.956789	3.650193	-0.371440
17	6	0	2.622197	2.796656	0.707446

18	6	0	3.357882	1.632922	0.949421
19	1	0	4.210892	1.704817	1.611490
20	6	0	3.042110	0.391162	0.377034
21	6	0	-3.282368	-1.502169	1.241843
22	6	0	-4.615362	-1.461810	2.019125
23	1	0	-4.626275	-0.679546	2.785643
24	1	0	-4.754694	-2.420837	2.527866
25	1	0	-5.475701	-1.313515	1.357684
26	6	0	-3.375918	-2.671688	0.230687
27	1	0	-4.174229	-2.490384	-0.497824
28	1	0	-3.611791	-3.599809	0.763739
29	1	0	-2.440623	-2.826015	-0.307414
30	6	0	-2.155718	-1.773185	2.270710
31	1	0	-1.179108	-1.864838	1.794012
32	1	0	-2.363228	-2.707499	2.804643
33	1	0	-2.106168	-0.966359	3.010687
34	6	0	3.891833	-0.865995	0.672648
35	6	0	5.091805	-0.540183	1.588318
36	1	0	5.771035	0.191369	1.137425
37	1	0	5.663299	-1.458132	1.758530
38	1	0	4.777026	-0.165278	2.568029
39	6	0	4.467691	-1.449168	-0.642619
40	1	0	5.092798	-0.707589	-1.152969
41	1	0	3.683505	-1.770745	-1.328418
42	1	0	5.095811	-2.317351	-0.413381
43	6	0	3.031239	-1.927919	1.400110
44	1	0	2.646023	-1.529913	2.345651
45	1	0	3.646854	-2.805238	1.630059
46	1	0	2.188172	-2.259164	0.794044
47	17	0	0.372325	-3.403037	-1.105200
48	1	0	-4.445489	2.910248	0.003594
49	1	0	2.914267	3.729719	1.178821

20, B3LYP/6-31+G(d,p), chloroform IEFPCM:
Sum of electronic and thermal Free Energies= -3749.839753

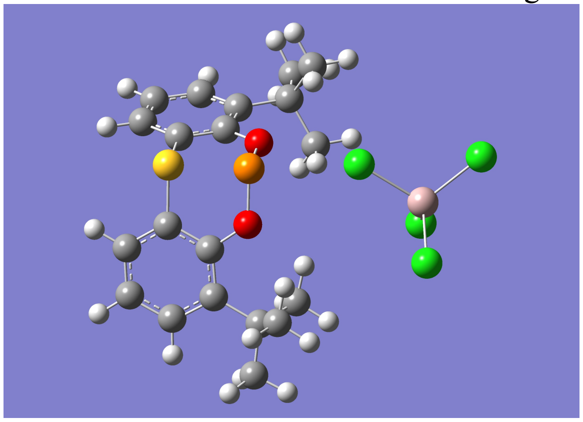

Center Number	Atomic Number	Atomic Type	Coordinates (Angstroms) X	Y	Z
1	15	0	-0.673029	-0.006540	-1.733198
2	8	0	-0.946195	-1.283404	-0.729900
3	8	0	-0.615711	1.329721	-0.776525
4	16	0	-3.021364	0.285679	-1.932039
5	6	0	-2.188888	-1.752222	-0.324331
6	6	0	-3.318867	-1.115885	-0.852755
7	6	0	-4.610481	-1.561055	-0.572747
8	1	0	-5.475408	-1.069924	-1.004276
9	6	0	-4.742267	-2.651429	0.281546
10	6	0	-3.608215	-3.271885	0.814837
11	1	0	-3.759710	-4.119126	1.470986
12	6	0	-2.292724	-2.862276	0.532721
13	6	0	-1.712640	2.047405	-0.313468
14	6	0	-2.970724	1.687268	-0.812265
15	6	0	-4.123176	2.398391	-0.477542
16	1	0	-5.090316	2.117919	-0.879849
17	6	0	-3.983809	3.471132	0.397381
18	6	0	-2.724729	3.815975	0.898369
19	1	0	-2.665149	4.661139	1.571475
20	6	0	-1.542128	3.131834	0.565524
21	6	0	-1.059879	-3.587762	1.110413
22	6	0	-1.473290	-4.751407	2.034752
23	1	0	-2.056964	-4.407347	2.895262
24	1	0	-0.568784	-5.229819	2.421703
25	1	0	-2.048486	-5.518519	1.505399
26	6	0	-0.210865	-4.178142	-0.043564
27	1	0	-0.798352	-4.889261	-0.634831
28	1	0	0.647565	-4.714259	0.374574
29	1	0	0.172971	-3.404369	-0.710151

30	6	0	-0.209274	-2.600744	1.947792
31	1	0	0.183396	-1.778043	1.348693
32	1	0	0.646184	-3.130994	2.378388
33	1	0	-0.797560	-2.183482	2.772441
34	6	0	-0.163887	3.547913	1.118752
35	6	0	-0.280032	4.755221	2.072223
36	1	0	-0.673380	5.644829	1.568724
37	1	0	0.717929	5.004697	2.444475
38	1	0	-0.909289	4.538283	2.942079
39	6	0	0.770361	3.957649	-0.047252
40	1	0	0.344488	4.796594	-0.608592
41	1	0	0.955487	3.134366	-0.738819
42	1	0	1.736712	4.275960	0.356644
43	6	0	0.457423	2.375202	1.917041
44	1	0	-0.190912	2.085361	2.751306
45	1	0	1.422114	2.685058	2.331031
46	1	0	0.634682	1.497038	1.294586
47	17	0	2.493975	-0.640835	-1.550477
48	1	0	-5.729167	-3.026479	0.530606
49	1	0	-4.855650	4.047014	0.688871
50	13	0	4.204375	-0.278642	-0.224591
51	17	0	5.751564	-1.713263	-0.710455
52	17	0	4.914468	1.742176	-0.550758
53	17	0	3.567156	-0.527412	1.830889

21, B3LYP/6-31+G(d,p), dichloromethane IEFPCM:
Sum of electronic and thermal Free Energies= -3592.289546

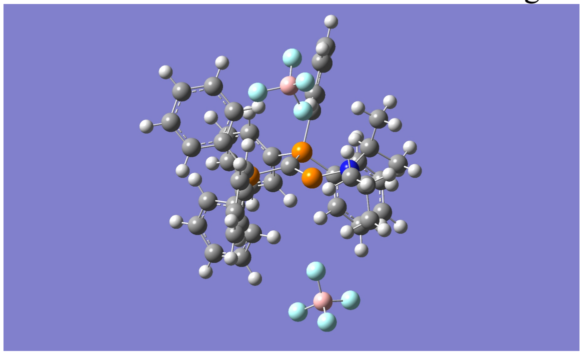

Center Number	Atomic Number	Atomic Type	Coordinates (Angstroms) X	Y	Z
1	6	0	8.576318	10.460861	7.702140
2	6	0	10.928365	6.988072	6.959121
3	1	0	11.418671	6.341919	7.686434
4	6	0	12.006420	7.582595	6.047717
5	1	0	12.547470	6.771361	5.550132

6	1	0	12.725590	8.179666	6.613934
7	1	0	11.564885	8.217939	5.273134
8	6	0	9.917626	6.125339	6.192564
9	1	0	9.462021	6.667387	5.359104
10	1	0	9.122126	5.765986	6.849764
11	1	0	10.443270	5.259775	5.777037
12	6	0	10.544389	7.955105	9.280659
13	1	0	10.059990	8.811289	9.744486
14	6	0	9.902230	6.683360	9.857413
15	1	0	8.845225	6.616176	9.593058
16	1	0	9.991516	6.709061	10.947910
17	1	0	10.407170	5.778131	9.507396
18	6	0	12.042844	8.036477	9.598408
19	1	0	12.587787	7.158705	9.239748
20	1	0	12.166672	8.076527	10.684450
21	1	0	12.498626	8.929495	9.168092
22	6	0	7.999896	12.893147	6.025821
23	6	0	7.175756	14.027346	6.099416
24	1	0	6.181851	13.967503	6.524685
25	6	0	7.643982	15.259530	5.639148
26	1	0	6.999815	16.130799	5.703667
27	6	0	8.931294	15.372575	5.108886
28	1	0	9.288938	16.333139	4.750612
29	6	0	9.762995	14.250591	5.050096
30	1	0	10.770542	14.318208	4.652258
31	6	0	9.302093	13.015771	5.508015
32	1	0	9.973384	12.165920	5.448555
33	6	0	7.253741	10.209815	5.008615
34	6	0	6.448575	9.056366	5.072335
35	1	0	5.968675	8.752663	5.997073
36	6	0	6.267484	8.276059	3.931564
37	1	0	5.649855	7.385662	3.990257
38	6	0	6.873126	8.639406	2.723940
39	1	0	6.725423	8.030424	1.837315
40	6	0	7.662708	9.789262	2.658267
41	1	0	8.129642	10.081975	1.723270
42	6	0	7.855942	10.576762	3.796040
43	1	0	8.465362	11.468409	3.720725
44	6	0	5.692460	11.335440	7.124152
45	6	0	4.721773	11.882458	6.261893
46	1	0	4.998040	12.268276	5.286081
47	6	0	3.383052	11.912134	6.648086
48	1	0	2.643375	12.338426	5.977960
49	6	0	2.994530	11.381859	7.883498
50	1	0	1.949617	11.398507	8.177098
51	6	0	3.948809	10.811641	8.726262

52	1	0	3.653449	10.372340	9.673445
53	6	0	5.293416	10.784309	8.346663
54	1	0	6.012699	10.309423	8.999165
55	6	0	8.435955	13.045309	9.329013
56	6	0	7.141976	13.360307	9.779701
57	1	0	6.440102	12.579695	10.045801
58	6	0	6.753091	14.693503	9.913087
59	1	0	5.753016	14.924678	10.265783
60	6	0	7.650165	15.720768	9.608366
61	1	0	7.347875	16.757308	9.721579
62	6	0	8.938788	15.412571	9.165054
63	1	0	9.642118	16.204913	8.930466
64	6	0	9.334653	14.082217	9.025096
65	1	0	10.341223	13.863180	8.689470
66	6	0	10.810453	11.357404	9.430642
67	6	0	11.617715	11.526174	8.293834
68	1	0	11.196782	11.514408	7.295890
69	6	0	12.994582	11.718234	8.432721
70	1	0	13.599326	11.849145	7.541019
71	6	0	13.572139	11.740295	9.704690
72	1	0	14.641878	11.890960	9.812405
73	6	0	12.774375	11.557915	10.838343
74	1	0	13.219903	11.559351	11.828090
75	6	0	11.398341	11.367212	10.707310
76	1	0	10.800591	11.219007	11.598449
77	6	0	8.225433	10.497132	10.707327
78	6	0	7.599388	9.245684	10.604381
79	1	0	7.537131	8.721279	9.657023
80	6	0	7.013583	8.662264	11.730703
81	1	0	6.515234	7.704630	11.624820
82	6	0	7.057448	9.315805	12.963668
83	1	0	6.601612	8.859840	13.837111
84	6	0	7.674184	10.566990	13.070387
85	1	0	7.698718	11.087016	14.022697
86	6	0	8.248665	11.163458	11.948451
87	1	0	8.690293	12.149771	12.042745
88	7	0	10.267108	8.067811	7.815457
89	15	0	9.269773	9.045005	6.934501
90	15	0	7.413571	11.235989	6.521111
91	15	0	8.990793	11.299827	9.252664
92	5	0	13.284374	12.170324	4.414359
93	5	0	5.651732	6.549671	8.235474
94	9	0	14.337018	12.160794	5.358912
95	9	0	12.931342	13.507131	4.116485
96	9	0	13.693039	11.512254	3.237150
97	9	0	12.160000	11.501315	4.964387

98	9	0	6.916590	7.184182	8.084774
99	9	0	5.213428	6.708854	9.568363
100	9	0	4.728592	7.152217	7.353360
101	9	0	5.779298	5.179199	7.934106

22, B3LYP/6-31+G(d,p), acetonitrile IEFPCM:
Sum of electronic and thermal Free Energies= -4592.526936

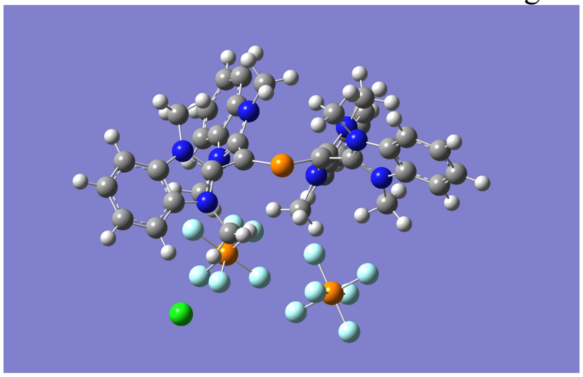

Center Number	Atomic Number	Atomic Type	Coordinates (Angstroms) X	Y	Z
1	6	0	3.217625	6.622572	11.634043
2	6	0	2.790693	7.006836	10.294248
3	6	0	2.258381	6.936061	8.116847
4	6	0	1.997087	6.563527	6.795646
5	1	0	2.079599	5.534904	6.464023
6	6	0	1.603530	7.582482	5.931698
7	1	0	1.387007	7.342336	4.896134
8	6	0	1.476419	8.918515	6.368973
9	1	0	1.168338	9.679798	5.660069
10	6	0	1.736131	9.285715	7.687210
11	1	0	1.641942	10.313791	8.016743
12	6	0	2.129798	8.261852	8.553507
13	6	0	3.159325	4.808438	9.093806
14	1	0	3.702142	4.527874	9.992736
15	1	0	3.837756	4.751301	8.240468
16	1	0	2.324214	4.130291	8.923923
17	6	0	2.420461	9.473425	10.740348
18	1	0	2.365242	9.194558	11.789559
19	1	0	1.533511	10.053367	10.482656
20	1	0	3.312705	10.078586	10.564807
21	6	0	4.336860	7.377271	12.202810
22	6	0	6.335161	8.380997	12.394742
23	6	0	7.616013	8.907230	12.204380
24	1	0	8.148832	8.799506	11.267002

25	6	0	8.180101	9.584393	13.282645
26	1	0	9.173994	10.006814	13.179220
27	6	0	7.492279	9.733454	14.505451
28	1	0	7.969939	10.266773	15.320517
29	6	0	6.215456	9.209883	14.692906
30	1	0	5.694416	9.320147	15.636658
31	6	0	5.650268	8.532746	13.608383
32	6	0	5.901936	7.166887	10.230166
33	1	0	5.457009	6.191337	10.050907
34	1	0	6.983614	7.043188	10.225265
35	1	0	5.611610	7.879041	9.454891
36	6	0	3.367033	7.985003	14.478660
37	1	0	2.389564	7.859385	14.019130
38	1	0	3.411361	8.969972	14.944821
39	1	0	3.520121	7.211542	15.232899
40	6	0	1.215253	4.517650	12.179081
41	6	0	0.227690	4.877301	11.166887
42	6	0	-1.303189	6.012513	9.982015
43	6	0	-2.190946	6.959233	9.463455
44	1	0	-2.279294	7.955024	9.881962
45	6	0	-2.959269	6.558149	8.373492
46	1	0	-3.662316	7.259196	7.936248
47	6	0	-2.843589	5.264433	7.820613
48	1	0	-3.457206	4.997909	6.966590
49	6	0	-1.961049	4.321478	8.340782
50	1	0	-1.867583	3.332066	7.908863
51	6	0	-1.195091	4.724533	9.438440
52	6	0	-0.385166	7.187303	12.000505
53	1	0	-1.405113	7.396819	12.329100
54	1	0	0.026684	8.078247	11.526465
55	1	0	0.221323	6.916086	12.860734
56	6	0	0.147388	2.657189	9.949786
57	1	0	1.121285	2.462731	10.391795
58	1	0	0.204638	2.491798	8.874232
59	1	0	-0.596324	1.985042	10.384445
60	6	0	0.918293	3.324347	12.975984
61	6	0	1.094747	1.371481	14.067519
62	6	0	1.521863	0.168735	14.638555
63	1	0	2.535685	-0.206167	14.530632
64	6	0	0.575624	-0.532561	15.382578
65	1	0	0.861844	-1.471187	15.845469
66	6	0	-0.740985	-0.054338	15.552383
67	1	0	-1.444791	-0.635316	16.139255
68	6	0	-1.160487	1.146646	14.985522
69	1	0	-2.173801	1.508019	15.115307
70	6	0	-0.210601	1.851667	14.241056

71	6	0	3.126156	2.064241	12.779922
72	1	0	3.323977	2.681243	11.907830
73	1	0	3.215380	1.013412	12.501473
74	1	0	3.850290	2.301417	13.560621
75	6	0	-1.439763	3.961017	13.631710
76	1	0	-1.103744	4.995415	13.622208
77	1	0	-1.958922	3.782388	14.572705
78	1	0	-2.124732	3.772166	12.802587
79	7	0	2.682353	6.188662	9.213205
80	7	0	2.455469	8.269387	9.907012
81	7	0	5.503436	7.647332	11.555478
82	7	0	4.410651	7.918309	13.451980
83	7	0	-0.414167	6.073062	11.052563
84	7	0	-0.237682	4.051253	10.191774
85	7	0	1.761850	2.295315	13.265655
86	7	0	-0.280686	3.069012	13.571422
87	15	0	2.683795	5.323636	12.675440
88	9	0	5.605659	3.796368	11.225956
89	9	0	7.176132	2.667344	12.523810
90	9	0	6.451076	1.746153	10.515312
91	9	0	8.621941	2.569866	10.700483
92	9	0	7.776053	4.619702	11.412470
93	9	0	7.051129	3.699442	9.403571
94	9	0	-0.276623	1.636113	6.629211
95	9	0	1.920015	0.878191	6.507302
96	9	0	0.514690	0.661805	4.665002
97	9	0	2.272252	2.190522	4.617763
98	9	0	1.479412	3.161707	6.582167
99	9	0	0.075013	2.945558	4.737196
100	17	0	4.863294	-1.633418	15.129737
101	15	0	7.118339	3.179585	10.963308
102	15	0	0.999078	1.909778	5.618595

23, B3LYP/6-31+G(d,p), benzene IEFPCM:
Sum of electronic and thermal Free Energies= -1267.055828

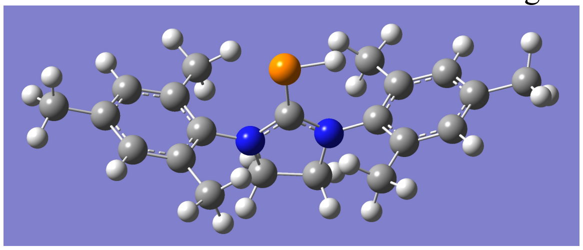

Center Number	Atomic Number	Atomic Type	Coordinates (Angstroms) X	Y	Z

1	15	0	0.135727	-0.007233	-1.852108
2	7	0	1.104339	-0.037255	0.732737
3	6	0	0.007651	0.000356	-0.093330
4	6	0	0.743152	0.144042	2.145046
5	1	0	0.997728	1.155950	2.488065
6	1	0	1.275562	-0.573354	2.776475
7	6	0	2.468745	-0.009084	0.295344
8	6	0	3.156275	-1.228959	0.151122
9	6	0	4.500195	-1.195023	-0.238832
10	1	0	5.036895	-2.134025	-0.354489
11	6	0	5.166514	0.010562	-0.487930
12	6	0	4.454577	1.205978	-0.331929
13	1	0	4.955213	2.152725	-0.523009
14	6	0	3.109732	1.222261	0.056471
15	6	0	2.460212	-2.545909	0.399025
16	1	0	2.092197	-2.623259	1.428719
17	1	0	3.141989	-3.382240	0.223351
18	1	0	1.596591	-2.661681	-0.264256
19	6	0	6.606994	0.020857	-0.945560
20	1	0	7.135438	0.908631	-0.584376
21	1	0	6.668894	0.027011	-2.041091
22	1	0	7.144655	-0.864463	-0.592705
23	6	0	2.372913	2.532727	0.204048
24	1	0	1.432882	2.519510	-0.356160
25	1	0	2.981695	3.360002	-0.170094
26	1	0	2.132814	2.748504	1.252246
27	1	0	-1.274496	-0.011195	-2.045435
28	7	0	-1.109658	0.050046	0.704510
29	6	0	-0.774804	-0.090756	2.131289
30	1	0	-1.037763	-1.092717	2.496028
31	1	0	-1.320847	0.643789	2.730400
32	6	0	-2.472506	0.011651	0.266376
33	6	0	-3.167344	1.227377	0.110594
34	6	0	-4.512301	1.183160	-0.270438
35	1	0	-5.052332	2.118852	-0.398747
36	6	0	-5.176853	-0.029554	-0.496672
37	6	0	-4.456543	-1.218188	-0.338568
38	1	0	-4.951855	-2.169056	-0.521627
39	6	0	-3.107737	-1.223584	0.041937
40	6	0	-2.472665	2.550041	0.331533
41	1	0	-2.098595	2.645551	1.357485
42	1	0	-3.157458	3.381856	0.146458
43	1	0	-1.611967	2.658810	-0.336882
44	6	0	-6.635769	-0.046569	-0.891982
45	1	0	-6.952168	-1.045344	-1.205584
46	1	0	-6.832309	0.646267	-1.717342

47	1	0	-7.275413	0.257904	-0.054510
48	6	0	-2.359304	-2.527916	0.183512
49	1	0	-1.448965	-2.528009	-0.424589
50	1	0	-2.983665	-3.366627	-0.135347
51	1	0	-2.060201	-2.715261	1.221556

24, B3LYP/6-31+G(d,p), benzene IEFPCM:
Sum of electronic and thermal Free Energies= -2285.295395

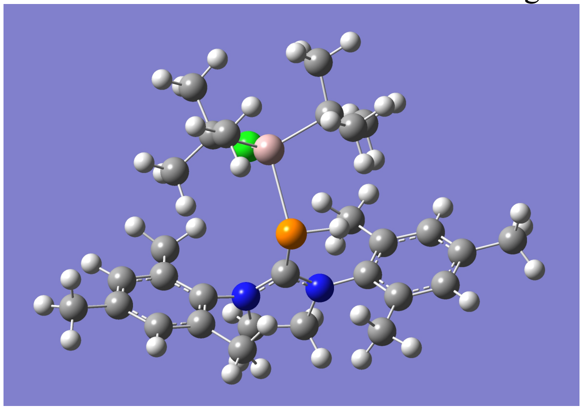

Center Number	Atomic Number	Atomic Type	Coordinates (Angstroms) X	Y	Z
1	15	0	-0.031658	0.067869	-1.049406
2	17	0	0.213315	2.025546	2.268076
3	13	0	0.231392	2.367028	0.059928
4	7	0	0.942516	-2.004131	0.573351
5	7	0	-1.267301	-1.952919	0.511071
6	6	0	2.325637	-1.835803	0.212491
7	6	0	2.777668	-2.332006	-1.029226
8	6	0	-0.133875	-1.338494	0.092471
9	6	0	3.222541	-1.303435	1.158537
10	6	0	4.578543	-1.235231	0.816635
11	1	0	5.275049	-0.813284	1.537230
12	6	0	5.057403	-1.676817	-0.420559
13	6	0	1.843267	-2.980243	-2.022157
14	1	0	1.176686	-3.703886	-1.540937
15	1	0	2.411606	-3.506149	-2.793279
16	1	0	1.209706	-2.234668	-2.514621
17	6	0	4.141151	-2.227046	-1.324610
18	1	0	4.496718	-2.599525	-2.282494
19	6	0	-2.632815	-1.631198	0.193371
20	6	0	2.051088	3.080792	-0.490041
21	6	0	0.545567	-3.120107	1.460123
22	1	0	0.819611	-2.894945	2.495039
23	1	0	1.059496	-4.035282	1.155346

24	6	0	-3.408916	-0.946103	1.146838
25	6	0	-1.464242	3.348561	-0.508695
26	6	0	2.766285	-0.824563	2.515896
27	1	0	1.826956	-0.269011	2.461847
28	1	0	3.517794	-0.167049	2.960010
29	1	0	2.623720	-1.664302	3.207988
30	6	0	3.160747	2.054032	-0.199908
31	1	0	3.206045	1.792803	0.863343
32	1	0	4.148707	2.461709	-0.477238
33	1	0	3.026964	1.124926	-0.765279
34	6	0	-4.757166	-0.709116	0.850373
35	1	0	-5.363037	-0.172648	1.576434
36	6	0	6.518720	-1.547313	-0.780508
37	1	0	6.712218	-0.589318	-1.279063
38	1	0	6.833865	-2.341232	-1.464488
39	1	0	7.154934	-1.587348	0.108641
40	6	0	-0.974396	-3.192319	1.262561
41	1	0	-1.285303	-4.064678	0.676012
42	1	0	-1.523906	-3.200599	2.206115
43	6	0	-3.190353	-2.096067	-1.015066
44	6	0	-5.340546	-1.134377	-0.347765
45	6	0	2.081392	3.414303	-1.997034
46	1	0	1.866296	2.536953	-2.620931
47	1	0	3.079131	3.782005	-2.293269
48	1	0	1.361244	4.196709	-2.265333
49	6	0	-2.822153	-0.471450	2.454290
50	1	0	-2.520037	-1.310810	3.092288
51	1	0	-3.557384	0.113850	3.011617
52	1	0	-1.939385	0.155888	2.300989
53	6	0	-2.705574	2.824178	0.238830
54	1	0	-2.916580	1.773277	0.010306
55	1	0	-3.600996	3.402221	-0.048946
56	1	0	-2.596022	2.910773	1.325759
57	6	0	-4.541145	-1.830222	-1.263384
58	1	0	-4.979400	-2.180505	-2.195033
59	6	0	2.374498	4.365234	0.306449
60	1	0	1.659555	5.172849	0.112145
61	1	0	3.373089	4.746437	0.030800
62	1	0	2.382523	4.184004	1.387723
63	6	0	-1.728855	3.243766	-2.025968
64	1	0	-0.891375	3.620164	-2.624749
65	1	0	-2.616566	3.837406	-2.304668
66	1	0	-1.927627	2.213323	-2.345462
67	6	0	-1.291532	4.843715	-0.153084
68	1	0	-1.090983	4.996207	0.914755
69	1	0	-2.211112	5.404895	-0.393333

70	1	0	-0.476101	5.312636	-0.715889
71	6	0	-2.368069	-2.852774	-2.031580
72	1	0	-1.604377	-2.211726	-2.485578
73	1	0	-3.006357	-3.232334	-2.833164
74	1	0	-1.847994	-3.707560	-1.586297
75	6	0	-6.788417	-0.834282	-0.657342
76	1	0	-7.365027	-0.664020	0.256342
77	1	0	-7.256999	-1.653608	-1.211476
78	1	0	-6.875063	0.068678	-1.274363
79	1	0	-1.435079	0.138767	-1.212686

25, B3LYP/6-31+G(d,p), chloroform IEFPCM:
Sum of electronic and thermal Free Energies= -1077.920660

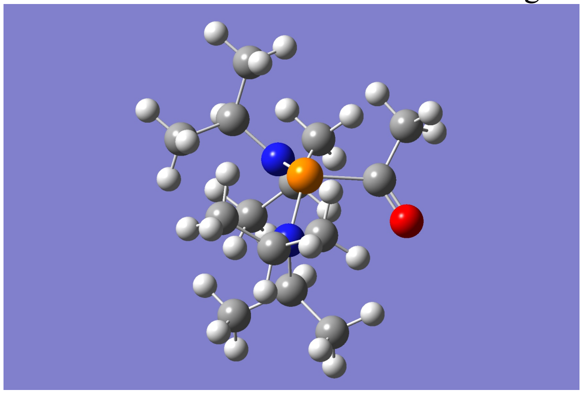

Center Number	Atomic Number	Atomic Type	Coordinates (Angstroms) X	Y	Z
1	15	0	2.776518	7.433755	4.054505
2	8	0	3.858512	6.065345	1.915226
3	7	0	2.733908	9.115033	3.697052
4	7	0	1.349518	6.616824	3.585167
5	6	0	4.079457	6.888064	2.795027
6	6	0	2.791213	9.710227	2.335480
7	1	0	2.890487	8.868280	1.643227
8	6	0	0.805472	5.585597	4.514468
9	1	0	-0.087412	5.193130	4.019019
10	6	0	-0.772382	7.348582	2.479153
11	1	0	-1.425641	6.634726	2.993221
12	1	0	-1.227868	7.566448	1.506347
13	1	0	-0.752084	8.270742	3.067027
14	6	0	1.508034	10.470285	1.951838
15	1	0	0.622944	9.839026	2.058346
16	1	0	1.568566	10.803626	0.909749
17	1	0	1.367192	11.361999	2.572289
18	6	0	0.617571	5.499259	1.438494

19	1	0	1.630664	5.125853	1.279364
20	1	0	0.164503	5.714462	0.463568
21	1	0	0.017907	4.711355	1.907733
22	6	0	2.605255	10.077223	4.827880
23	1	0	2.599588	11.068988	4.366825
24	6	0	0.344037	6.160937	5.862296
25	1	0	1.190792	6.543380	6.441377
26	1	0	-0.142290	5.379496	6.457739
27	1	0	-0.370095	6.976415	5.715400
28	6	0	0.647091	6.781326	2.290010
29	1	0	1.222650	7.520007	1.727053
30	6	0	4.027262	10.606594	2.130158
31	1	0	3.989585	11.503870	2.757164
32	1	0	4.075526	10.939414	1.087405
33	1	0	4.950864	10.069676	2.359360
34	6	0	1.764130	4.399467	4.717214
35	1	0	2.065689	3.970533	3.757590
36	1	0	1.280784	3.614843	5.311422
37	1	0	2.668386	4.713541	5.250432
38	6	0	1.281813	9.935869	5.594473
39	1	0	0.426922	9.983709	4.913406
40	1	0	1.182916	10.742370	6.330310
41	1	0	1.235719	8.985118	6.133947
42	6	0	3.809579	10.035997	5.781828
43	1	0	3.875243	9.068886	6.291065
44	1	0	3.715279	10.815139	6.547012
45	1	0	4.744883	10.202148	5.238115
46	6	0	5.501011	7.357736	3.065453
47	1	0	6.055403	6.515997	3.498156
48	1	0	5.986229	7.624021	2.121480
49	1	0	5.546043	8.197467	3.760626

26, B3LYP/6-31+G(d,p), chloroform IEFPCM:
Sum of electronic and thermal Free Energies= -613.431512

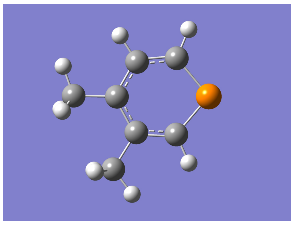

Center Number	Atomic Number	Atomic Type	Coordinates (Angstroms) X	Y	Z
1	6	0	-0.048641	-0.479374	0.033559

2	6	0	1.346872	-0.552827	0.089963
3	6	0	1.362903	2.109425	0.014198
4	6	0	-0.023778	1.981038	-0.035204
5	6	0	-0.725537	0.764758	-0.027587
6	1	0	1.786865	-1.548085	0.136110
7	1	0	1.780874	3.113949	0.001355
8	1	0	-0.616819	2.893262	-0.083758
9	15	0	2.479233	0.774997	0.095718
10	6	0	-0.851353	-1.764045	0.038474
11	1	0	-1.467193	-1.855888	-0.864080
12	1	0	-0.194944	-2.635862	0.088170
13	1	0	-1.535382	-1.806638	0.894348
14	6	0	-2.235575	0.799639	-0.085948
15	1	0	-2.617499	0.271735	-0.968184
16	1	0	-2.684341	0.314506	0.789253
17	1	0	-2.603061	1.827942	-0.124980

----

27 isomer A, B3LYP/6-31+G(d,p), benzene IEFPCM:
Sum of electronic and thermal Free Energies= -1242.190806

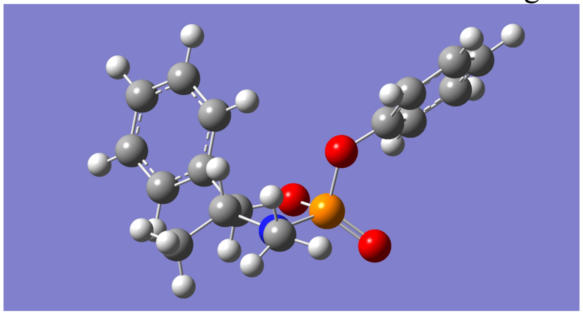

Center Number	Atomic Number	Atomic Type	Coordinates (Angstroms) X	Y	Z
1	6	0	-1.791127	0.610080	0.643906
2	6	0	-1.520851	1.638944	-0.487144
3	8	0	-0.484929	0.178391	1.140659
4	7	0	-0.265140	2.293930	-0.070938
5	15	0	0.702553	1.207772	0.741337
6	8	0	1.587328	1.677223	1.835007
7	8	0	1.527162	0.437352	-0.450989
8	6	0	-2.677359	2.620888	-0.656064
9	1	0	-3.600391	2.072787	-0.869227
10	1	0	-2.500809	3.302052	-1.492831
11	1	0	-2.820112	3.213631	0.253337
12	6	0	-2.604322	-0.588318	0.221733
13	6	0	-3.931036	-0.716942	0.650567
14	6	0	-2.059792	-1.570916	-0.618772

15	6	0	-4.711294	-1.800238	0.234083
16	1	0	-4.356411	0.029257	1.316889
17	6	0	-2.834956	-2.656304	-1.028724
18	1	0	-1.025323	-1.493047	-0.939443
19	6	0	-4.164269	-2.771986	-0.606613
20	1	0	-5.739041	-1.887177	0.574099
21	1	0	-2.401246	-3.414434	-1.674310
22	1	0	-4.765636	-3.617999	-0.926256
23	1	0	-1.367701	1.099768	-1.436639
24	1	0	-2.286603	1.122603	1.475861
25	6	0	0.319870	3.321062	-0.933384
26	1	0	0.492556	2.949800	-1.953517
27	1	0	-0.338557	4.192319	-0.977853
28	1	0	1.273595	3.645447	-0.511190
29	6	0	2.636840	-0.385749	-0.266957
30	6	0	3.646734	-0.298145	-1.225757
31	6	0	2.716622	-1.302091	0.783169
32	6	0	4.756784	-1.140172	-1.128696
33	1	0	3.553247	0.422941	-2.031212
34	6	0	3.836431	-2.133497	0.872668
35	1	0	1.923289	-1.365115	1.520257
36	6	0	4.857271	-2.058795	-0.079185
37	1	0	5.544623	-1.073037	-1.873210
38	1	0	3.905091	-2.843017	1.691902
39	1	0	5.723165	-2.709188	-0.003128

----

27 isomer B, B3LYP/6-31+G(d,p), benzene IEFPCM:
Sum of electronic and thermal Free Energies= -1242.186316

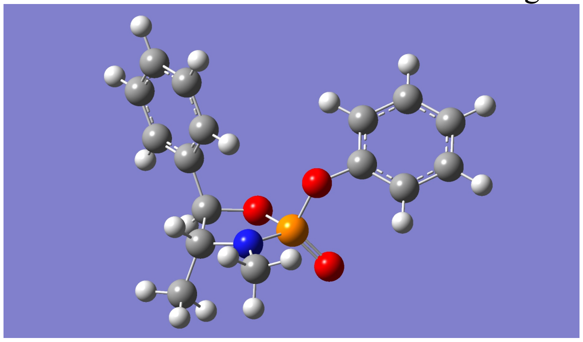

Center Number	Atomic Number	Atomic Type	Coordinates (Angstroms) X	Y	Z
1	6	0	-1.107586	0.226662	0.820668
2	6	0	-1.121004	1.274880	-0.337359
3	8	0	0.211857	0.312163	1.453013

4	7	0	0.293405	1.439876	-0.701838
5	15	0	1.328491	1.041406	0.532948
6	8	0	2.236616	-0.141651	-0.141176
7	6	0	-1.810173	2.582174	0.073137
8	1	0	-2.865581	2.394649	0.299240
9	1	0	-1.771669	3.319239	-0.734523
10	1	0	-1.335073	3.016779	0.959143
11	6	0	-1.426275	-1.196875	0.416961
12	6	0	-2.425117	-1.893795	1.108730
13	6	0	-0.756543	-1.839552	-0.637027
14	6	0	-2.757564	-3.205954	0.757125
15	1	0	-2.946869	-1.408315	1.929805
16	6	0	-1.084813	-3.149785	-0.987219
17	1	0	0.027037	-1.318805	-1.178187
18	6	0	-2.086620	-3.837104	-0.291991
19	1	0	-3.535111	-3.731193	1.304049
20	1	0	-0.557493	-3.636110	-1.802915
21	1	0	-2.339755	-4.856789	-0.567322
22	1	0	-1.648563	0.846914	-1.198916
23	1	0	-1.808891	0.536139	1.598789
24	6	0	0.670943	2.265516	-1.843827
25	1	0	0.091693	1.959720	-2.721819
26	1	0	0.508363	3.335233	-1.665040
27	1	0	1.728692	2.112901	-2.070878
28	6	0	3.435549	-0.608920	0.410403
29	6	0	4.598355	0.156916	0.333479
30	6	0	3.443892	-1.890033	0.961585
31	6	0	5.792502	-0.376409	0.825506
32	1	0	4.560234	1.154444	-0.088618
33	6	0	4.645461	-2.413319	1.447199
34	1	0	2.521209	-2.459234	1.003040
35	6	0	5.821101	-1.659546	1.380997
36	1	0	6.701471	0.215670	0.774175
37	1	0	4.659140	-3.410437	1.877353
38	1	0	6.752814	-2.068357	1.760145
39	8	0	2.163114	2.055871	1.228687

28, B3LYP/6-31+G(d,p), benzene IEFPCM:
Sum of electronic and thermal Free Energies= -1242.190832

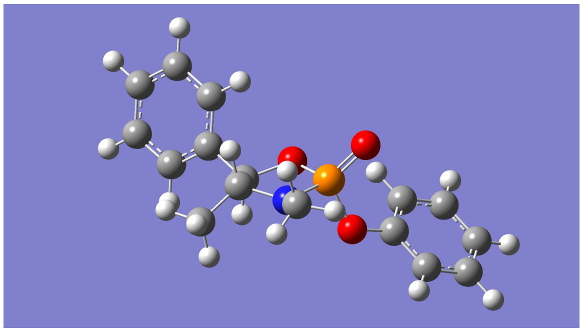

Center Number	Atomic Number	Atomic Type	Coordinates (Angstroms) X	Y	Z
1	6	0	1.449575	0.152831	-0.492313
2	6	0	1.525635	1.677295	-0.219831
3	8	0	0.398823	-0.356434	0.384881
4	7	0	0.126445	2.061101	0.053481
5	15	0	-0.740676	0.768887	0.654120
6	8	0	-1.881130	0.537001	-0.495917
7	8	0	-1.309201	0.786711	2.026211
8	6	0	-2.986932	-0.300741	-0.341400
9	6	0	-4.213655	0.187822	-0.790550
10	6	0	-2.867745	-1.592723	0.172845
11	6	0	-5.342611	-0.631618	-0.718680
12	1	0	-4.270674	1.195906	-1.187797
13	6	0	-4.006715	-2.399208	0.246748
14	1	0	-1.905502	-1.963356	0.509742
15	6	0	-5.244447	-1.925556	-0.197789
16	1	0	-6.299601	-0.253865	-1.066471
17	1	0	-3.920374	-3.403126	0.651766
18	1	0	-6.124238	-2.558888	-0.138627
19	6	0	2.135525	2.446353	-1.389186
20	1	0	3.147903	2.081479	-1.589236
21	1	0	2.210036	3.513762	-1.163706
22	1	0	1.529155	2.324066	-2.292631
23	6	0	2.731272	-0.601182	-0.240304
24	6	0	3.483035	-1.078148	-1.321135
25	6	0	3.198622	-0.815147	1.064952
26	6	0	4.692889	-1.745440	-1.106078
27	1	0	3.120157	-0.933230	-2.335518
28	6	0	4.401793	-1.488385	1.280878
29	1	0	2.611014	-0.470267	1.910354
30	6	0	5.154331	-1.951869	0.195844
31	1	0	5.265707	-2.110458	-1.953501
32	1	0	4.750810	-1.654734	2.295815
33	1	0	6.090056	-2.476273	0.366058
34	1	0	2.136394	1.847333	0.682049

35	1	0	1.121854	-0.006554	-1.526763
36	6	0	-0.151582	3.426173	0.501298
37	1	0	0.454220	3.696533	1.377354
38	1	0	0.050482	4.136927	-0.304919
39	1	0	-1.205092	3.515420	0.773601

29, B3LYP/6-31+G(d,p), chloroform IEFPCM:
Sum of electronic and thermal Free Energies= -2862.315343

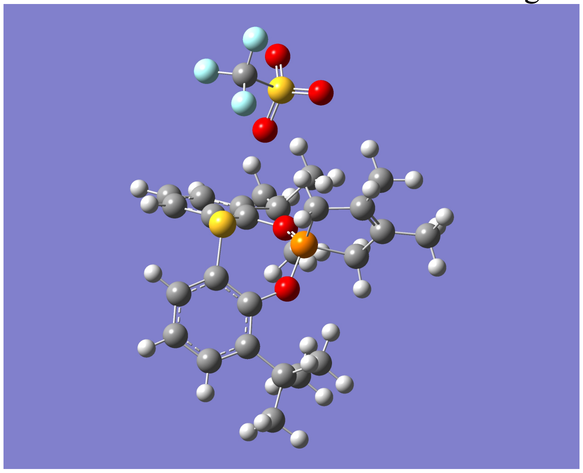

Center Number	Atomic Number	Atomic Type	Coordinates (Angstroms) X	Y	Z
1	8	0	0.362378	1.096829	0.854412
2	8	0	2.304059	-0.655931	0.229409
3	16	0	0.145130	-0.220105	-1.843369
4	6	0	0.003719	1.972957	-0.177528
5	6	0	-0.071971	1.521602	-1.499915
6	6	0	-0.412477	2.409345	-2.523197
7	1	0	-0.489514	2.048238	-3.542894
8	6	0	-0.663273	3.739139	-2.206707
9	6	0	-0.587285	4.166404	-0.879607
10	1	0	-0.800913	5.205960	-0.670014
11	6	0	-0.256210	3.311127	0.184629
12	6	0	2.772549	-0.640536	-1.089828
13	6	0	1.900754	-0.383985	-2.156445
14	6	0	2.390727	-0.358624	-3.465311
15	1	0	1.707946	-0.174259	-4.287623
16	6	0	3.744912	-0.579751	-3.686758
17	6	0	4.594693	-0.834711	-2.608480
18	1	0	5.641547	-1.007759	-2.819243
19	6	0	4.151583	-0.877755	-1.275779
20	6	0	-0.191297	3.827789	1.640538
21	6	0	-0.554333	5.326124	1.728313

22	1	0	0.139219	5.955585	1.160935
23	1	0	-0.499115	5.639692	2.775367
24	1	0	-1.572806	5.524941	1.379723
25	6	0	-1.204351	3.062191	2.528068
26	1	0	-2.224215	3.195923	2.153828
27	1	0	-1.164060	3.454609	3.550229
28	1	0	-1.000443	1.991804	2.568813
29	6	0	1.246580	3.676044	2.197538
30	1	0	1.578517	2.637059	2.216519
31	1	0	1.285973	4.060957	3.222569
32	1	0	1.957889	4.251039	1.594511
33	6	0	5.130398	-1.166652	-0.113738
34	6	0	6.566988	-1.406121	-0.627151
35	1	0	6.628382	-2.270181	-1.296814
36	1	0	7.216906	-1.608702	0.229602
37	1	0	6.973218	-0.532046	-1.146433
38	6	0	4.701335	-2.447775	0.645028
39	1	0	4.689746	-3.310428	-0.029876
40	1	0	3.712803	-2.355285	1.096570
41	1	0	5.419057	-2.657273	1.445663
42	6	0	5.188001	0.044528	0.850975
43	1	0	5.532477	0.941208	0.324586
44	1	0	5.896661	-0.163776	1.660087
45	1	0	4.219212	0.265753	1.300789
46	6	0	1.293280	-0.582242	2.699616
47	6	0	-0.338502	-1.778543	0.860144
48	6	0	0.460767	-1.760564	3.179419
49	1	0	1.043565	0.345045	3.227496
50	1	0	2.369601	-0.747616	2.822229
51	6	0	-0.345565	-2.347024	2.270115
52	1	0	-1.308901	-1.371907	0.545138
53	1	0	-0.026401	-2.508062	0.104030
54	15	0	0.872726	-0.423349	0.933661
55	6	0	-1.279957	-3.503878	2.485927
56	1	0	-1.011389	-4.342119	1.829995
57	1	0	-1.271195	-3.863340	3.516041
58	1	0	-2.303448	-3.210150	2.225360
59	6	0	0.619667	-2.134001	4.626379
60	1	0	-0.001821	-2.983866	4.911434
61	1	0	1.664403	-2.387731	4.847247
62	1	0	0.355270	-1.288866	5.274528
63	1	0	4.143151	-0.562423	-4.696056
64	1	0	-0.931970	4.444346	-2.986435
65	16	0	-4.222055	-0.682029	0.107200
66	8	0	-4.141285	-1.810524	1.066486
67	8	0	-5.298868	0.300472	0.361532

68	8	0	-2.905921	-0.097251	-0.263395
69	6	0	-4.769403	-1.519237	-1.487236
70	9	0	-5.956445	-2.139121	-1.330766
71	9	0	-4.899984	-0.625709	-2.489494
72	9	0	-3.870272	-2.448039	-1.881487

----

anti-**30**, B3LYP/6-31+G(d,p), toluene IEFPCM:
Sum of electronic and thermal Free Energies= -695.556834

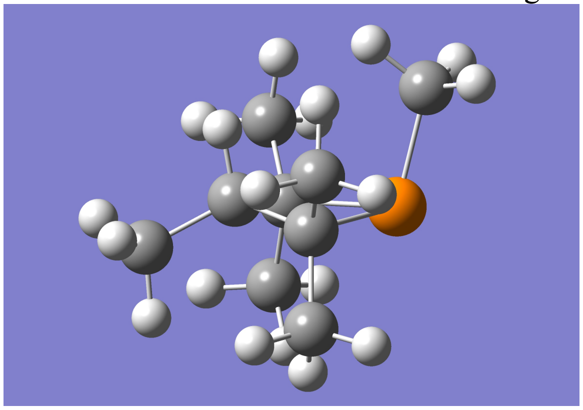

Center Number	Atomic Number	Atomic Type	Coordinates (Angstroms) X	Y	Z
1	6	0	-0.646210	1.048378	0.224886
2	6	0	0.786531	0.482883	-0.074640
3	6	0	-0.409936	2.555946	-0.139262
4	1	0	-0.772530	1.009205	1.315300
5	6	0	1.317233	-0.478767	0.992271
6	1	0	0.740327	-1.413831	0.978619
7	1	0	2.365929	-0.741076	0.806812
8	1	0	1.246545	-0.064654	2.002314
9	6	0	0.955109	-0.169073	-1.457309
10	1	0	2.011266	-0.399067	-1.638281
11	1	0	0.393247	-1.110355	-1.513001
12	1	0	0.617332	0.467546	-2.279247
13	6	0	-0.848416	2.965372	-1.555451
14	1	0	-1.942667	2.955583	-1.641984
15	1	0	-0.505999	3.983195	-1.774281
16	1	0	-0.448590	2.314562	-2.337584
17	6	0	-1.005509	3.545824	0.865888
18	1	0	-0.700663	4.575077	0.640823
19	1	0	-2.102985	3.514358	0.822153
20	1	0	-0.712335	3.325204	1.896341
21	6	0	-1.852239	0.332047	-0.391187
22	1	0	-1.900246	-0.709224	-0.051218
23	1	0	-2.788053	0.817589	-0.090325

24	1	0	-1.818270	0.323449	-1.484854
25	15	0	1.493991	2.272645	-0.103381
26	6	0	1.947746	2.591444	1.681723
27	1	0	2.931193	2.150566	1.873691
28	1	0	2.038462	3.672669	1.827106
29	1	0	1.239946	2.198671	2.417606

----

syn-**30** isomer A, B3LYP/6-31+G(d,p), toluene IEFPCM:
Sum of electronic and thermal Free Energies= -695.556246

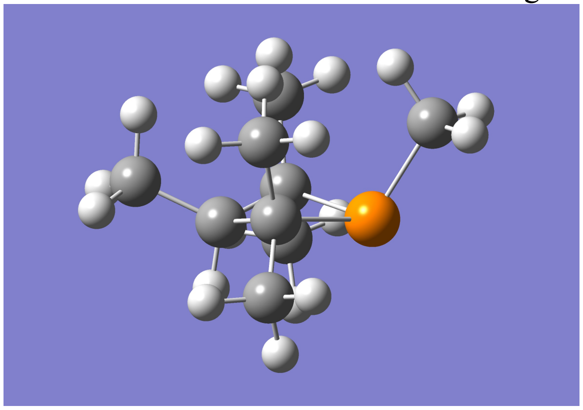

Center Number	Atomic Number	Atomic Type	Coordinates (Angstroms) X	Y	Z
1	6	0	-0.622226	1.061953	0.210369
2	6	0	0.837034	0.510144	0.012851
3	6	0	-0.362563	2.590710	-0.052384
4	1	0	-0.849944	0.964351	1.280677
5	6	0	1.261163	-0.498348	1.088165
6	1	0	0.672904	-1.423115	1.006889
7	1	0	2.318583	-0.769819	0.981962
8	1	0	1.120447	-0.096104	2.096851
9	6	0	1.127161	-0.079689	-1.373137
10	1	0	2.200182	-0.267437	-1.495059
11	1	0	0.613057	-1.042404	-1.492085
12	1	0	0.809167	0.562032	-2.197974
13	6	0	-0.694227	3.067362	-1.472123
14	1	0	-1.781893	3.094260	-1.619075
15	1	0	-0.318488	4.083922	-1.635846
16	1	0	-0.277130	2.431216	-2.256175
17	6	0	-1.050406	3.513027	0.962412
18	1	0	-0.753459	4.558185	0.812111
19	1	0	-2.143108	3.461700	0.856711
20	1	0	-0.797581	3.239988	1.992311
21	6	0	-1.761309	0.381309	-0.552395
22	1	0	-1.844397	-0.673173	-0.264431

23	1	0	-2.720767	0.859398	-0.323112
24	1	0	-1.621179	0.419851	-1.636883
25	15	0	1.494370	2.286144	0.326116
26	6	0	2.466863	2.802853	-1.181234
27	1	0	2.599537	3.889315	-1.148101
28	1	0	3.462646	2.351342	-1.120561
29	1	0	2.016294	2.533115	-2.139701

----

syn-**30** isomer B, B3LYP/6-31+G(d,p), toluene IEFPCM:
Sum of electronic and thermal Free Energies= -695.553205

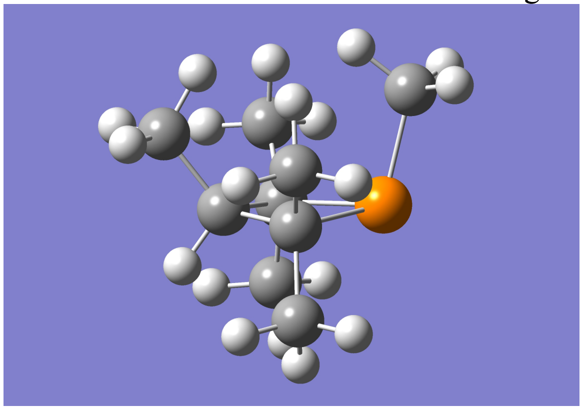

Center Number	Atomic Number	Atomic Type	Coordinates (Angstroms) X	Y	Z
1	6	0	-0.623965	1.062108	0.222394
2	6	0	0.836693	0.511675	-0.017530
3	6	0	-0.361856	2.589605	-0.082390
4	6	0	1.446196	-0.364753	1.080889
5	1	0	0.931751	-1.333632	1.130899
6	1	0	2.500853	-0.569766	0.861220
7	1	0	1.397449	0.084778	2.075357
8	6	0	0.952084	-0.232418	-1.358750
9	1	0	1.995879	-0.483177	-1.580747
10	1	0	0.378769	-1.169256	-1.323280
11	1	0	0.570264	0.357215	-2.198165
12	6	0	-0.913069	2.996224	-1.459802
13	1	0	-2.011466	2.965586	-1.450813
14	1	0	-0.605492	4.015281	-1.721774
15	1	0	-0.570756	2.332974	-2.260421
16	6	0	-0.845896	3.607118	0.954886
17	1	0	-0.489439	4.611279	0.695722
18	1	0	-1.943161	3.647226	0.971822
19	1	0	-0.509091	3.388632	1.970709
20	15	0	1.532528	2.294079	-0.190543
21	6	0	2.155770	2.702409	1.524364

22	1	0	3.147186	2.254322	1.645566
23	1	0	2.275455	3.787990	1.599364
24	1	0	1.517055	2.364230	2.344667
25	6	0	-1.256108	0.739887	1.580838
26	1	0	-2.253794	1.185756	1.656236
27	1	0	-1.371619	-0.342363	1.703461
28	1	0	-0.666713	1.106315	2.426309
29	1	0	-1.299614	0.648490	-0.540821

----

anti-**30[O]**, B3LYP/6-31+G(d,p), toluene IEFPCM:
Sum of electronic and thermal Free Energies= -770.817738

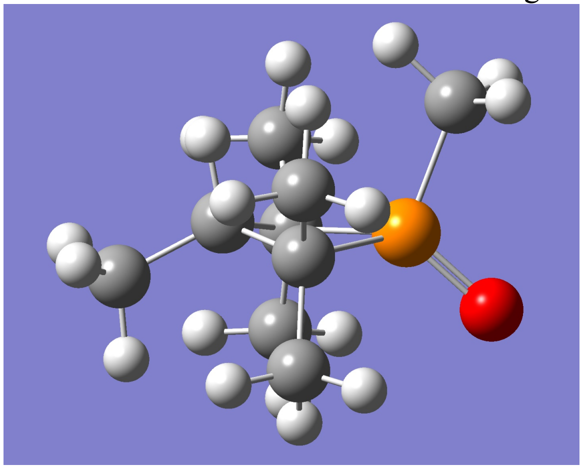

Center Number	Atomic Number	Atomic Type	Coordinates (Angstroms) X	Y	Z
1	6	0	-0.553666	1.091828	-0.111022
2	6	0	0.932067	0.547873	-0.117698
3	6	0	-0.280744	2.648709	-0.178316
4	15	0	1.490662	2.284133	0.320319
5	6	0	1.647281	2.425905	2.143834
6	1	0	2.580221	1.944125	2.449722
7	1	0	1.701670	3.486579	2.404854
8	1	0	0.812116	1.966254	2.677562
9	8	0	2.654482	2.935022	-0.396245
10	1	0	-0.960727	0.884444	0.887573
11	6	0	-1.526862	0.500276	-1.132123
12	1	0	-1.640247	-0.578634	-0.977363
13	1	0	-2.519240	0.952699	-1.026357
14	1	0	-1.196241	0.657080	-2.162533
15	6	0	1.195244	-0.569166	0.898919
16	1	0	0.670122	-1.483929	0.595323
17	1	0	2.262815	-0.809327	0.958149
18	1	0	0.849730	-0.309674	1.904345

19	6	0	1.465766	0.121818	-1.495798
20	1	0	2.539279	-0.082401	-1.435282
21	1	0	0.965028	-0.796356	-1.824544
22	1	0	1.326224	0.878773	-2.270227
23	6	0	-1.142392	3.481074	0.778080
24	1	0	-0.818396	4.527751	0.794459
25	1	0	-2.189135	3.467627	0.447896
26	1	0	-1.116606	3.102067	1.804220
27	6	0	-0.344525	3.262406	-1.587504
28	1	0	-1.381634	3.283092	-1.942791
29	1	0	0.024624	4.292380	-1.564067
30	1	0	0.253889	2.723229	-2.324609

----

syn-**30[O]** isomer A, B3LYP/6-31+G(d,p), toluene IEFPCM:
Sum of electronic and thermal Free Energies= -770.816337

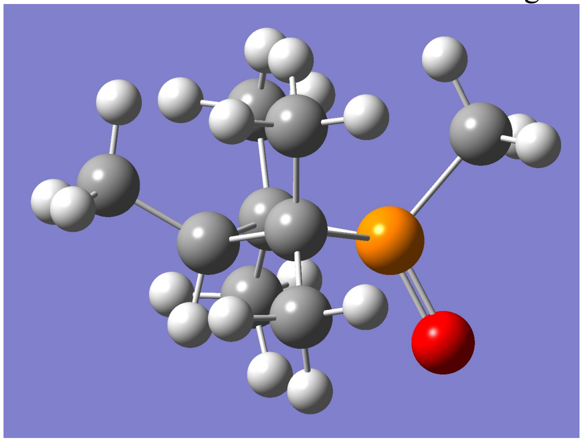

Center Number	Atomic Number	Atomic Type	Coordinates (Angstroms) X	Y	Z
1	6	0	-0.644649	1.048312	0.198997
2	6	0	0.824099	0.487082	0.013634
3	6	0	-0.388711	2.590411	-0.053113
4	1	0	-0.883594	0.944096	1.264685
5	6	0	1.258269	-0.477569	1.121929
6	1	0	0.627610	-1.375978	1.105420
7	1	0	2.297394	-0.797316	0.985592
8	1	0	1.178158	-0.017519	2.110497
9	6	0	1.113957	-0.127152	-1.364751
10	1	0	2.185469	-0.323185	-1.480111
11	1	0	0.591997	-1.086000	-1.467197
12	1	0	0.799397	0.504715	-2.199674
13	6	0	-0.740265	3.081870	-1.466375
14	1	0	-1.828797	3.102009	-1.597882
15	1	0	-0.372095	4.102017	-1.620398

16	1	0	-0.328919	2.455740	-2.263124
17	6	0	-1.033911	3.500957	0.996953
18	1	0	-0.782183	4.552427	0.818889
19	1	0	-2.126882	3.407710	0.954500
20	1	0	-0.704222	3.246401	2.007894
21	6	0	-1.767559	0.375978	-0.591484
22	1	0	-1.852731	-0.681296	-0.316410
23	1	0	-2.730734	0.849449	-0.370453
24	1	0	-1.611748	0.427169	-1.672967
25	15	0	1.435664	2.249448	0.229613
26	8	0	2.003669	2.620452	1.584788
27	6	0	2.515043	2.831874	-1.136207
28	1	0	2.618966	3.917279	-1.048267
29	1	0	3.502841	2.379411	-1.009316
30	1	0	2.126756	2.584038	-2.125726

----

syn-**30[O]** isomer B, B3LYP/6-31+G(d,p), toluene IEFPCM:
Sum of electronic and thermal Free Energies= -770.814962

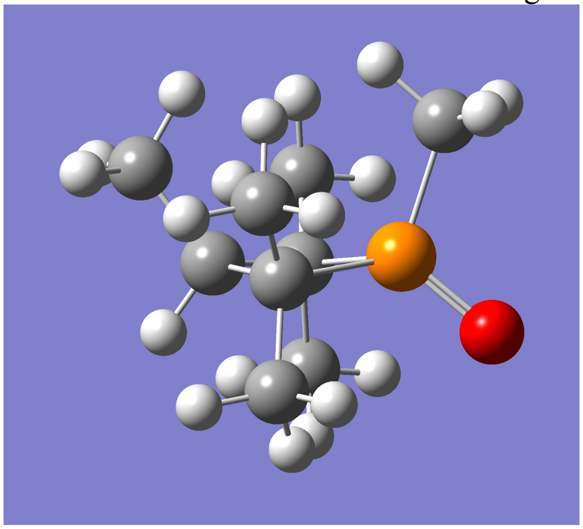

Center Number	Atomic Number	Atomic Type	Coordinates (Angstroms) X	Y	Z
1	6	0	0.000000	1.257670	-0.492403
2	6	0	1.216149	0.248514	-0.304301
3	6	0	-1.216149	0.248515	-0.304301
4	1	0	0.000000	1.618200	-1.528563
5	6	0	0.000000	-1.103532	2.116927
6	1	0	-0.888214	-1.649600	2.446956
7	1	0	0.888212	-1.649602	2.446956
8	1	0	0.000000	-0.109512	2.569409
9	15	0	0.000000	-1.049650	0.281823

10	6	0	2.326295	0.659337	0.671466
11	1	0	3.028389	-0.169029	0.817332
12	1	0	2.897287	1.503055	0.264956
13	1	0	1.953687	0.956803	1.654691
14	6	0	1.849406	-0.145223	-1.650378
15	1	0	2.380053	0.718288	-2.071350
16	1	0	2.569370	-0.959466	-1.520549
17	1	0	1.111121	-0.477152	-2.384270
18	6	0	0.000000	2.489710	0.417065
19	1	0	0.882601	3.108301	0.224780
20	1	0	-0.882601	3.108301	0.224780
21	1	0	0.000000	2.239748	1.482843
22	6	0	-1.849406	-0.145223	-1.650378
23	1	0	-2.569370	-0.959465	-1.520550
24	1	0	-2.380053	0.718288	-2.071350
25	1	0	-1.111121	-0.477152	-2.384270
26	6	0	-2.326295	0.659337	0.671465
27	1	0	-2.897287	1.503055	0.264956
28	1	0	-3.028389	-0.169028	0.817331
29	1	0	-1.953687	0.956803	1.654691
30	8	0	0.000000	-2.439396	-0.318896

----

anti-**31**, B3LYP/6-31+G(d,p), toluene IEFPCM:
Sum of electronic and thermal Free Energies= -1288.034505

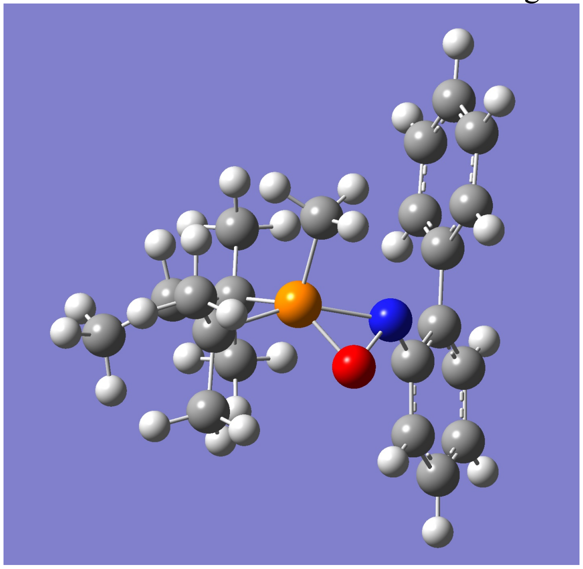

Center Number	Atomic Number	Atomic Type	Coordinates (Angstroms) X	Y	Z
1	6	0	-3.006001	-0.813439	1.039698
2	6	0	-3.312939	-0.244698	-0.396601

3	6	0	-1.467741	-0.473227	1.149600
4	15	0	-1.443240	-0.293643	-0.722921
5	6	0	-1.118025	-1.888105	-1.563769
6	1	0	-1.261040	-1.730527	-2.636689
7	1	0	-0.089076	-2.198173	-1.384163
8	1	0	-1.810789	-2.660022	-1.224300
9	8	0	-1.132151	1.028125	-1.617366
10	7	0	0.265183	0.455402	-1.236873
11	6	0	1.046683	1.447626	-0.653100
12	6	0	2.300637	1.068202	-0.079388
13	6	0	0.684750	2.811659	-0.656059
14	6	0	3.106415	2.070769	0.480307
15	6	0	1.519484	3.779838	-0.100140
16	1	0	-0.261956	3.094590	-1.098660
17	6	0	2.736853	3.418238	0.481182
18	1	0	4.064297	1.780674	0.903590
19	1	0	1.211893	4.822217	-0.123644
20	1	0	3.394446	4.166703	0.912372
21	6	0	2.785147	-0.341580	-0.052445
22	6	0	3.337535	-0.877748	1.126088
23	6	0	2.765146	-1.157708	-1.199799
24	6	0	3.854373	-2.175849	1.159498
25	1	0	3.352611	-0.273019	2.028586
26	6	0	3.283939	-2.453593	-1.168278
27	1	0	2.346237	-0.764822	-2.119591
28	6	0	3.829781	-2.971703	0.011353
29	1	0	4.271099	-2.564826	2.084647
30	1	0	3.268738	-3.058540	-2.071089
31	1	0	4.231072	-3.980911	0.033806
32	1	0	-3.083464	-1.907083	0.974011
33	6	0	-3.907930	-0.366287	2.191918
34	1	0	-4.943750	-0.670751	2.005009
35	1	0	-3.594780	-0.831035	3.133577
36	1	0	-3.898976	0.717649	2.333959
37	6	0	-4.246069	-1.123364	-1.239540
38	1	0	-5.268022	-1.077355	-0.840822
39	1	0	-4.287645	-0.776369	-2.278376
40	1	0	-3.947560	-2.175549	-1.245729
41	6	0	-3.858624	1.196053	-0.410008
42	1	0	-3.904729	1.568335	-1.438261
43	1	0	-4.875828	1.218572	-0.000767
44	1	0	-3.253643	1.902520	0.161998
45	6	0	-0.608702	-1.616413	1.703489
46	1	0	0.458795	-1.403654	1.594324
47	1	0	-0.816978	-1.741747	2.773691
48	1	0	-0.818024	-2.572125	1.213235

49	6	0	-1.139723	0.823202	1.908973
50	1	0	-1.398179	0.696692	2.966758
51	1	0	-0.071040	1.039871	1.848470
52	1	0	-1.674106	1.699272	1.537048

----

syn-**31**, B3LYP/6-31+G(d,p), toluene IEFPCM:
Sum of electronic and thermal Free Energies= -1288.034037

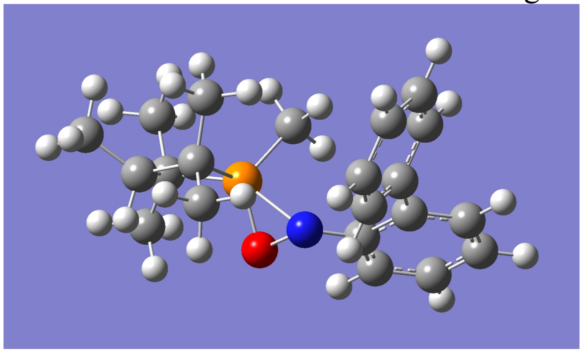

Center Number	Atomic Number	Atomic Type	Coordinates (Angstroms) X	Y	Z
1	6	0	-3.273729	0.684160	0.237863
2	6	0	-0.641187	0.788114	1.798181
3	15	0	-1.379716	0.489356	0.147290
4	6	0	-3.856964	0.700176	1.659957
5	1	0	-3.615748	1.641972	2.164866
6	1	0	-4.950247	0.623648	1.618818
7	1	0	-3.500478	-0.119060	2.291084
8	6	0	-3.866592	1.852916	-0.561505
9	1	0	-4.954999	1.734520	-0.649796
10	1	0	-3.679964	2.810825	-0.062883
11	1	0	-3.449359	1.913291	-1.570210
12	8	0	-0.950096	1.513987	-1.030212
13	7	0	0.321698	0.634649	-0.839517
14	6	0	1.394686	1.451581	-0.492992
15	6	0	2.608262	0.818947	-0.080024
16	6	0	1.353176	2.862094	-0.549963
17	6	0	3.698981	1.624845	0.272576
18	6	0	2.465633	3.630914	-0.206972
19	1	0	0.436307	3.341681	-0.869101
20	6	0	3.648257	3.020990	0.215782
21	1	0	4.619609	1.134275	0.578055
22	1	0	2.400826	4.714241	-0.270832
23	1	0	4.517831	3.613129	0.483654
24	6	0	2.757878	-0.663574	-0.008508
25	6	0	2.500046	-1.485232	-1.121183

26	6	0	3.232099	-1.272512	1.167590
27	6	0	2.717263	-2.863002	-1.060306
28	1	0	2.135880	-1.033681	-2.037844
29	6	0	3.443941	-2.653045	1.232127
30	1	0	3.432167	-0.656479	2.040186
31	6	0	3.187862	-3.455072	0.117063
32	1	0	2.523686	-3.475992	-1.936582
33	1	0	3.808674	-3.099308	2.153378
34	1	0	3.354543	-4.527562	0.162647
35	6	0	-1.919325	-1.243001	-0.324197
36	6	0	-3.391736	-0.703846	-0.500337
37	1	0	-3.510493	-0.482337	-1.568380
38	6	0	-1.325525	-1.814432	-1.613421
39	1	0	-0.282599	-2.106736	-1.474616
40	1	0	-1.900042	-2.703230	-1.906606
41	1	0	-1.367039	-1.093529	-2.434170
42	6	0	-1.711717	-2.233904	0.835035
43	1	0	-2.128056	-1.890475	1.786525
44	1	0	-2.190722	-3.189695	0.593689
45	1	0	-0.644396	-2.427020	0.982192
46	6	0	-4.533044	-1.639733	-0.100238
47	1	0	-5.502561	-1.166070	-0.290057
48	1	0	-4.499291	-2.562241	-0.690749
49	1	0	-4.499833	-1.919331	0.956648
50	1	0	0.145194	0.055799	1.989962
51	1	0	-0.190202	1.783854	1.784097
52	1	0	-1.393657	0.746428	2.585835

----

32 isomer A, B3LYP/6-31+G(d,p), $CHCl_3$ IEFPCM:
Sum of electronic and thermal Free Energies= -882.443537

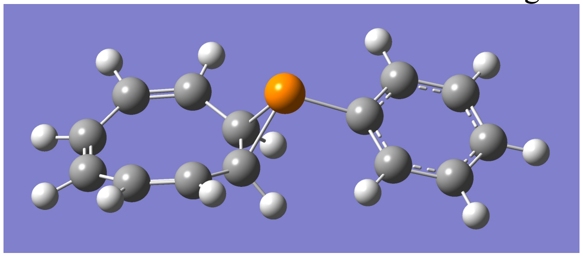

Center Number	Atomic Number	Atomic Type	Coordinates (Angstroms) X	Y	Z
1	6	0	0.795083	0.814185	0.450827
2	6	0	1.809172	0.478834	-0.563857
3	6	0	1.003149	4.045229	-0.686360
4	6	0	2.209701	1.074032	-1.714745
5	6	0	1.345787	3.496079	-1.982003

6	6	0	1.835176	2.295000	-2.397373
7	1	0	2.262676	-0.492474	-0.367799
8	1	0	0.072846	0.005508	0.575964
9	1	0	2.924537	0.483480	-2.287481
10	1	0	2.067288	2.256805	-3.461252
11	6	0	0.264239	2.128667	0.909507
12	1	0	-0.762590	2.057152	1.272674
13	6	0	0.586194	3.504384	0.484669
14	1	0	0.360253	4.226172	1.269128
15	15	0	1.450491	1.336534	2.171340
16	1	0	1.259925	4.236784	-2.776435
17	1	0	1.037642	5.134544	-0.677814
18	6	0	0.299502	0.457980	3.330317
19	6	0	-0.769315	1.114138	3.964426
20	6	0	0.567511	-0.878717	3.675726
21	6	0	-1.561599	0.445684	4.902863
22	1	0	-0.987107	2.152919	3.730530
23	6	0	-0.226522	-1.548790	4.610408
24	1	0	1.398608	-1.402786	3.209822
25	6	0	-1.294380	-0.887985	5.225866
26	1	0	-2.385520	0.967883	5.381135
27	1	0	-0.010405	-2.584257	4.857897
28	1	0	-1.910202	-1.406773	5.954679

----

32 isomer B, B3LYP/6-31+G(d,p), $CHCl_3$ IEFPCM:
Sum of electronic and thermal Free Energies= -882.442977

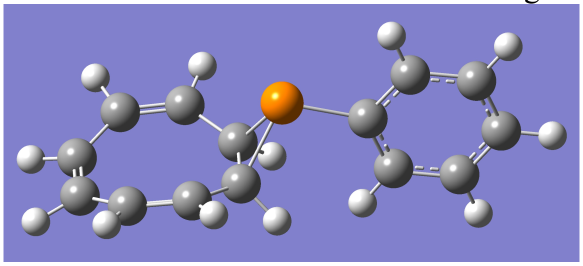

Center Number	Atomic Number	Atomic Type	Coordinates (Angstroms) X	Y	Z
1	6	0	0.840885	0.744431	0.422109
2	6	0	1.860947	1.716846	-0.009457
3	6	0	3.215591	-1.685936	-0.073600
4	6	0	3.215397	1.685875	-0.073130
5	6	0	4.215222	-0.681157	0.222209
6	6	0	4.215138	0.681109	0.222368
7	1	0	1.411174	2.677933	-0.256897
8	1	0	0.201691	1.166318	1.199692

9	1	0	3.660773	2.640511	-0.352357
10	1	0	5.192618	1.121828	0.415792
11	6	0	0.840841	-0.744743	0.421238
12	1	0	0.201548	-1.167392	1.198361
13	6	0	1.861129	-1.716935	-0.010335
14	1	0	1.411413	-2.677947	-0.258184
15	15	0	-0.245625	0.000522	-0.958393
16	1	0	5.192746	-1.121803	0.415563
17	1	0	3.661061	-2.640525	-0.352834
18	6	0	-1.963625	0.000057	-0.268125
19	6	0	-2.325031	-0.000359	1.091306
20	6	0	-2.991005	0.000301	-1.230111
21	6	0	-3.668283	-0.000458	1.473261
22	1	0	-1.566880	-0.000628	1.869623
23	6	0	-4.336071	0.000154	-0.849320
24	1	0	-2.737610	0.000581	-2.287786
25	6	0	-4.678548	-0.000190	0.504974
26	1	0	-3.925919	-0.000761	2.528615
27	1	0	-5.111665	0.000344	-1.609724
28	1	0	-5.722093	-0.000255	0.805693

33, B3LYP/6-31+G(d,p), $CHCl_3$ IEFPCM:
Sum of electronic and thermal Free Energies= -882.454271

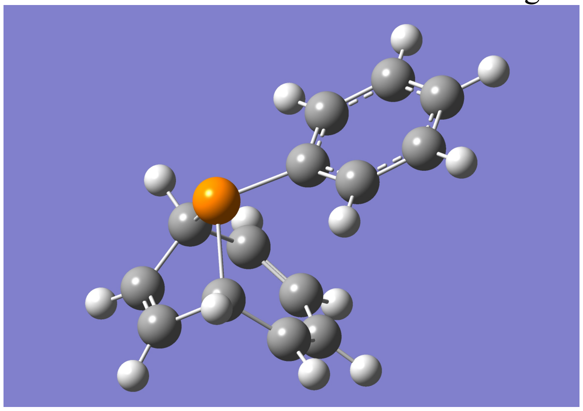

Center Number	Atomic Number	Atomic Type	Coordinates (Angstroms) X	Y	Z
1	6	0	0.132729	0.728102	-0.063130
2	6	0	1.413912	0.377121	-0.254360
3	6	0	0.761357	3.698813	-0.900154
4	6	0	2.449084	1.264595	0.418819
5	6	0	2.152563	3.316711	-1.109241
6	6	0	2.894170	2.322889	-0.559773
7	1	0	1.725931	-0.427449	-0.915592
8	1	0	-0.705513	0.238433	-0.552492

9	1	0	3.313420	0.685137	0.753464
10	1	0	3.935987	2.264642	-0.872649
11	6	0	-0.092473	1.960631	0.798495
12	1	0	-1.001766	1.866779	1.397732
13	6	0	-0.195339	3.170620	-0.096919
14	1	0	-1.165624	3.665746	-0.107422
15	15	0	1.432357	1.820974	1.927456
16	6	0	1.960988	3.540066	2.335621
17	6	0	1.018855	4.494059	2.758386
18	6	0	3.324794	3.872701	2.409425
19	6	0	1.425730	5.747723	3.222844
20	1	0	-0.042717	4.263330	2.725115
21	6	0	3.733547	5.125915	2.873959
22	1	0	4.078640	3.153282	2.100442
23	6	0	2.785190	6.069645	3.280242
24	1	0	0.679893	6.471985	3.538721
25	1	0	4.792823	5.363958	2.916875
26	1	0	3.102132	7.044042	3.640618
27	1	0	2.678318	3.954872	-1.817466
28	1	0	0.455323	4.566267	-1.482527

34, B3LYP/6-31+G(d,p), $CHCl_3$ IEFPCM:
Sum of electronic and thermal Free Energies= -882.455741

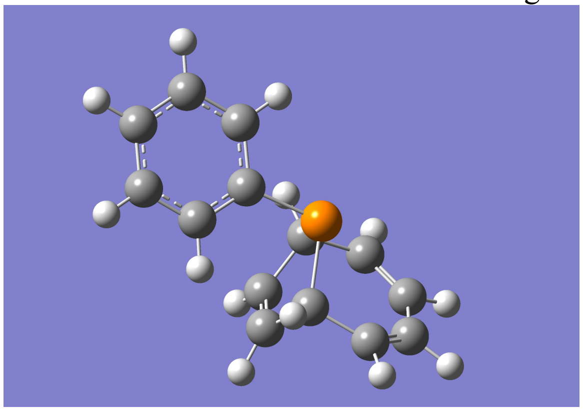

Center Number	Atomic Number	Atomic Type	Coordinates (Angstroms) X	Y	Z
1	6	0	-0.518705	0.878455	-0.196692
2	6	0	0.696206	0.332528	-0.346379
3	6	0	0.531727	3.659744	-1.193942
4	6	0	1.832641	1.099441	0.311040
5	6	0	1.861584	3.065259	-1.352717
6	6	0	2.444330	2.004069	-0.743836
7	1	0	0.899710	-0.525497	-0.983113
8	1	0	-1.412189	0.508579	-0.694486

9	1	0	2.608808	0.449019	0.723020
10	1	0	3.454700	1.750591	-1.060187
11	6	0	-0.554788	2.171768	0.598153
12	1	0	-1.441606	2.258564	1.231769
13	6	0	-0.504667	3.319908	-0.389539
14	1	0	-1.409511	3.919703	-0.473415
15	15	0	1.018450	2.131405	1.672269
16	1	0	2.472605	3.568576	-2.099992
17	1	0	0.359355	4.513357	-1.847047
18	6	0	0.536684	0.862336	2.937676
19	6	0	-0.151694	1.345288	4.066776
20	6	0	0.866181	-0.502587	2.896489
21	6	0	-0.522760	0.490491	5.107407
22	1	0	-0.395830	2.403159	4.137008
23	6	0	0.511016	-1.357159	3.945416
24	1	0	1.396374	-0.915043	2.045034
25	6	0	-0.188917	-0.866343	5.050906
26	1	0	-1.060289	0.886135	5.964624
27	1	0	0.778763	-2.408965	3.893641
28	1	0	-0.466039	-1.532573	5.862774

33[O], B3LYP/6-31+G(d,p), $CHCl_3$ IEFPCM:
Sum of electronic and thermal Free Energies= -957.707969

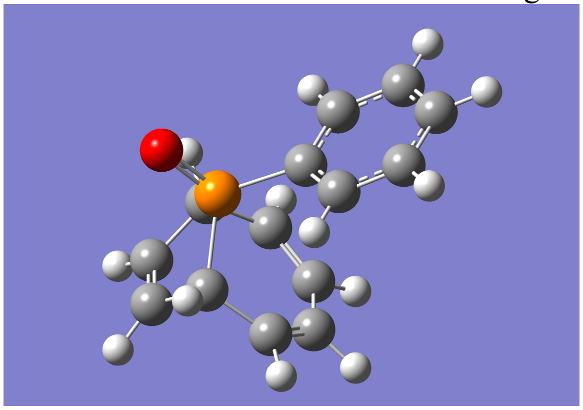

Center Number	Atomic Number	Atomic Type	Coordinates (Angstroms) X	Y	Z
1	6	0	0.132656	0.716140	-0.037789
2	6	0	1.414324	0.364743	-0.229373
3	6	0	0.746357	3.672824	-0.931969
4	6	0	2.451898	1.266368	0.427055
5	6	0	2.144794	3.288654	-1.141721
6	6	0	2.891292	2.304965	-0.588597
7	1	0	1.726910	-0.459070	-0.864482
8	1	0	-0.708961	0.208584	-0.500149

9	1	0	3.312967	0.705557	0.799950
10	1	0	3.918244	2.210428	-0.935188
11	6	0	-0.090697	1.963698	0.807481
12	1	0	-0.977614	1.882632	1.441284
13	6	0	-0.208555	3.156046	-0.124523
14	1	0	-1.193982	3.614955	-0.169650
15	15	0	1.445758	1.909267	1.854004
16	6	0	1.974126	3.577145	2.356491
17	6	0	1.023939	4.515999	2.787787
18	6	0	3.339277	3.893328	2.435667
19	6	0	1.433225	5.758396	3.276982
20	1	0	-0.036510	4.284748	2.743871
21	6	0	3.746880	5.136151	2.925561
22	1	0	4.088880	3.175351	2.115385
23	6	0	2.794501	6.070427	3.344011
24	1	0	0.690926	6.479769	3.605359
25	1	0	4.805113	5.373429	2.980474
26	1	0	3.112099	7.036920	3.724186
27	1	0	2.657406	3.906213	-1.876595
28	1	0	0.433824	4.517611	-1.542592
29	8	0	1.356866	0.949194	3.023789

34[O], B3LYP/6-31+G(d,p), $CHCl_3$ IEFPCM:
Sum of electronic and thermal Free Energies= -957.711652

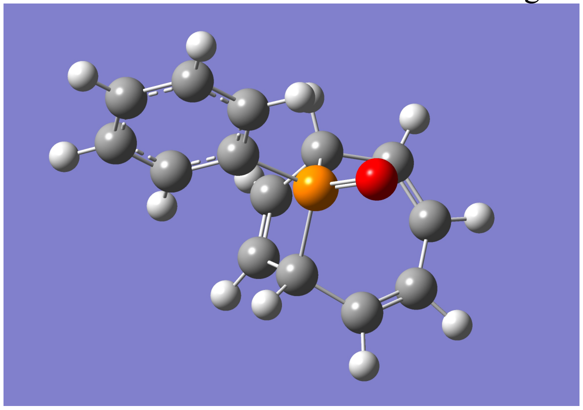

Center Number	Atomic Number	Atomic Type	Coordinates (Angstroms) X	Y	Z
1	6	0	1.192786	0.363493	1.749697
2	6	0	1.216621	1.469758	0.986555
3	6	0	3.453459	-0.965396	-0.009532
4	6	0	1.127755	1.244108	-0.512200
5	6	0	3.476063	0.241222	-0.841187
6	6	0	2.530187	1.176846	-1.088443
7	1	0	1.352364	2.471624	1.385353

8	1	0	1.306470	0.378183	2.830210
9	1	0	0.546577	2.016580	-1.022808
10	1	0	2.803786	1.996228	-1.749312
11	6	0	1.086792	-0.951810	1.002304
12	1	0	0.472774	-1.688311	1.527116
13	6	0	2.479413	-1.502693	0.761393
14	1	0	2.719718	-2.419276	1.295614
15	15	0	0.278815	-0.412725	-0.592083
16	1	0	4.429001	0.400594	-1.342047
17	1	0	4.392644	-1.514955	-0.023089
18	6	0	-1.510382	-0.165740	-0.278434
19	6	0	-2.372654	-1.080150	-0.907388
20	6	0	-2.059863	0.860115	0.509595
21	6	0	-3.756062	-0.978982	-0.741457
22	1	0	-1.952595	-1.860085	-1.534054
23	6	0	-3.444332	0.961202	0.667934
24	1	0	-1.417564	1.579817	1.003545
25	6	0	-4.294805	0.041492	0.046802
26	1	0	-4.410015	-1.693141	-1.233212
27	1	0	-3.856887	1.760999	1.275975
28	1	0	-5.370458	0.123541	0.172760
29	8	0	0.500793	-1.279132	-1.809437

Table S23. Coordinates of optimized structures for 1a–34[O], M06-2X/6-31+G(d,p), IEFPCM solvation.

1a isomer A, M06-2X/6-31+G(d,p), benzene IEFPCM:

Sum of electronic and thermal Free Energies= -1309.720519

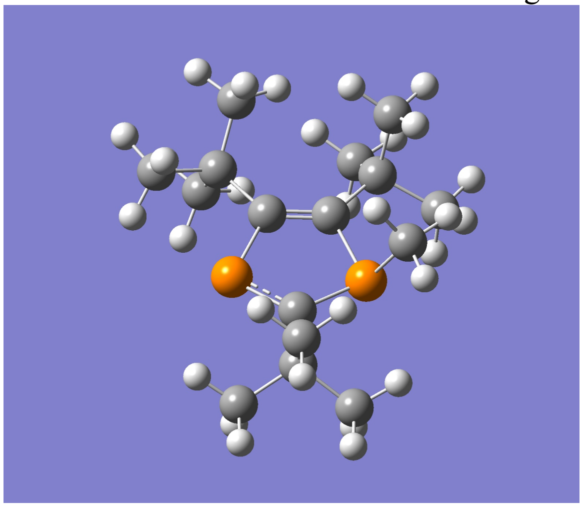

Center Number	Atomic Number	Atomic Type	Coordinates (Angstroms) X	Y	Z
1	6	0	-2.364132	4.070166	0.481020
2	6	0	-1.061724	4.319895	0.126812
3	6	0	-2.132477	6.707128	0.954870
4	15	0	-3.290068	5.503807	1.188663
5	15	0	-0.551097	6.057441	0.428701
6	6	0	-3.210777	2.766147	0.457870
7	6	0	-3.021614	1.926183	-0.817777
8	6	0	-2.945901	1.942656	1.733590
9	6	0	-4.725027	3.089357	0.465239
10	1	0	-3.279096	2.522937	-1.699737
11	1	0	-2.019931	1.533573	-0.959543
12	1	0	-3.703730	1.069998	-0.783049
13	1	0	-3.201610	2.536171	2.618061
14	1	0	-3.577147	1.046275	1.734883
15	1	0	-1.906561	1.632170	1.831766
16	1	0	-5.280678	2.154941	0.337770
17	1	0	-5.056530	3.543000	1.401537
18	1	0	-4.999262	3.759363	-0.357070
19	6	0	0.078412	3.394349	-0.385054
20	6	0	0.167968	2.050573	0.362412
21	6	0	-0.054879	3.187026	-1.907983
22	6	0	1.471762	4.026841	-0.151072
23	1	0	0.285070	2.227819	1.436631
24	1	0	-0.684928	1.394274	0.220542
25	1	0	1.055021	1.510809	0.013231

26	1	0	0.057174	4.140948	-2.433094
27	1	0	0.736132	2.515134	-2.260573
28	1	0	-1.017380	2.766616	-2.195378
29	1	0	2.232396	3.339675	-0.535407
30	1	0	1.611495	4.976976	-0.670696
31	1	0	1.665556	4.188537	0.912936
32	6	0	-2.366373	8.185166	1.270622
33	6	0	-3.402899	8.355695	2.392070
34	6	0	-1.063389	8.872963	1.708470
35	6	0	-2.905835	8.885372	0.006690
36	1	0	-4.369639	7.924738	2.113863
37	1	0	-3.064699	7.870107	3.312755
38	1	0	-3.555239	9.421096	2.595951
39	1	0	-0.302063	8.843836	0.921671
40	1	0	-1.260837	9.924573	1.942105
41	1	0	-0.646414	8.392177	2.598594
42	1	0	-3.070745	9.949176	0.212546
43	1	0	-2.206729	8.805855	-0.830333
44	1	0	-3.856104	8.438250	-0.300294
45	6	0	-0.353942	6.764108	-1.288363
46	1	0	-1.247225	6.569817	-1.887799
47	1	0	-0.197335	7.842045	-1.204356
48	1	0	0.519586	6.337465	-1.783774

1a isomer B, M06-2X/6-31+G(d,p), benzene IEFPCM:
Sum of electronic and thermal Free Energies= -1309.718285

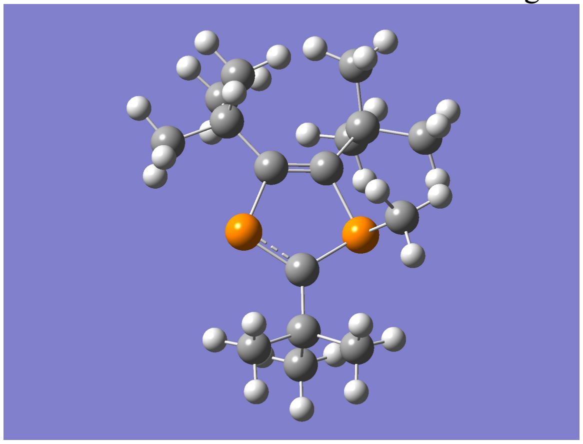

Center Number	Atomic Number	Atomic Type	Coordinates (Angstroms) X	Y	Z
1	6	0	-2.403478	4.040724	0.378578
2	6	0	-1.099434	4.283216	0.029099
3	6	0	-2.201949	6.699621	0.738341
4	15	0	-3.382221	5.513567	0.917089

5	15	0	-0.603969	6.053078	0.216430
6	6	0	-3.223925	2.718789	0.396703
7	6	0	-4.608663	2.901283	1.062060
8	6	0	-3.530894	2.288006	-1.051994
9	6	0	-2.560329	1.598681	1.219091
10	1	0	-4.526584	3.223323	2.104964
11	1	0	-5.237964	3.620773	0.531826
12	1	0	-5.125799	1.936347	1.046785
13	1	0	-2.639925	2.140506	-1.657028
14	1	0	-4.107364	1.355512	-1.048499
15	1	0	-4.138648	3.057480	-1.540487
16	1	0	-3.219201	0.723411	1.230092
17	1	0	-1.593439	1.273885	0.847282
18	1	0	-2.426063	1.929302	2.255006
19	6	0	0.092586	3.329734	-0.267745
20	6	0	1.264712	4.051603	-0.970938
21	6	0	0.649114	2.847589	1.090301
22	6	0	-0.209656	2.145149	-1.202660
23	1	0	1.008379	4.326982	-1.997271
24	1	0	1.607251	4.940127	-0.432508
25	1	0	2.112068	3.360747	-1.021123
26	1	0	-0.107850	2.361851	1.705435
27	1	0	1.472921	2.143206	0.925450
28	1	0	1.038939	3.701194	1.654994
29	1	0	0.723038	1.598992	-1.380555
30	1	0	-0.932120	1.431219	-0.819600
31	1	0	-0.568632	2.509672	-2.170720
32	6	0	-2.379406	8.139363	1.222597
33	6	0	-1.674927	9.149525	0.304628
34	6	0	-3.863524	8.520993	1.312768
35	6	0	-1.757178	8.249167	2.630447
36	1	0	-0.596267	8.963889	0.252693
37	1	0	-2.089478	9.121679	-0.708153
38	1	0	-1.815516	10.161056	0.699785
39	1	0	-4.401184	7.879829	2.017829
40	1	0	-3.956830	9.555713	1.659011
41	1	0	-4.351048	8.437967	0.336019
42	1	0	-1.859545	9.274696	3.003752
43	1	0	-2.261240	7.571633	3.326123
44	1	0	-0.693519	7.992932	2.609891
45	6	0	-0.554610	6.621649	-1.565634
46	1	0	0.379982	6.319375	-2.035696
47	1	0	-1.402339	6.205563	-2.116429
48	1	0	-0.608236	7.709054	-1.589375

2, M06-2X/6-31+G(d,p), benzene IEFPCM:
Sum of electronic and thermal Free Energies= -1422.544854

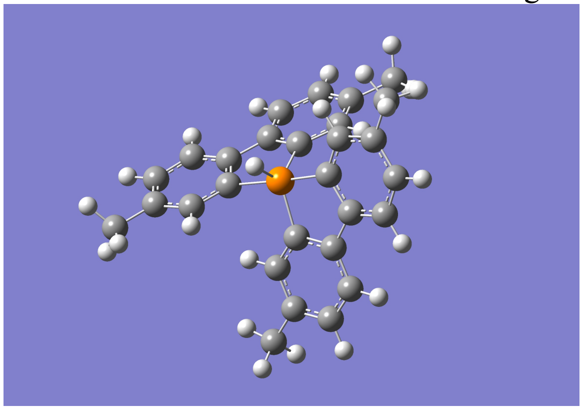

Center Number	Atomic Number	Atomic Type	Coordinates (Angstroms) X	Y	Z
1	6	0	2.201933	1.615601	-0.187153
2	6	0	1.878528	0.413993	-0.814095
3	6	0	2.851875	-0.271118	-1.537414
4	6	0	4.163585	0.209028	-1.612493
5	6	0	4.471925	1.411677	-0.957068
6	6	0	3.504175	2.121503	-0.253259
7	1	0	2.598465	-1.201920	-2.048240
8	1	0	5.486314	1.799841	-1.013580
9	1	0	3.766781	3.061781	0.224332
10	6	0	1.036278	2.243469	0.469787
11	6	0	1.052974	3.401754	1.248723
12	6	0	-0.178043	1.570297	0.265559
13	6	0	-0.125906	3.867668	1.822873
14	1	0	1.983275	3.936238	1.417792
15	6	0	-1.350493	2.038531	0.856311
16	6	0	-1.340711	3.195335	1.642454
17	1	0	-0.104378	4.769177	2.430991
18	1	0	-2.284663	1.504680	0.711550
19	6	0	-1.036276	-2.243466	0.469798
20	6	0	-1.052969	-3.401748	1.248738
21	6	0	0.125915	-3.867662	1.822882
22	6	0	1.340720	-3.195332	1.642452
23	6	0	1.350498	-2.038530	0.856306
24	6	0	0.178045	-1.570296	0.265560
25	1	0	-1.983269	-3.936229	1.417816
26	1	0	0.104389	-4.769167	2.431006
27	1	0	2.284667	-1.504681	0.711537
28	6	0	-2.201933	-1.615598	-0.187137
29	6	0	-1.878532	-0.413994	-0.814085
30	6	0	-3.504180	-2.121493	-0.253223

31	6	0	-2.851886	0.271121	-1.537393
32	6	0	-4.471935	-1.411665	-0.957021
33	1	0	-3.766788	-3.061763	0.224384
34	6	0	-4.163597	-0.209021	-1.612459
35	1	0	-2.598480	1.201926	-2.048215
36	1	0	-5.486329	-1.799819	-1.013511
37	15	0	-0.000002	0.000000	-0.717551
38	6	0	2.604908	-3.714842	2.279989
39	1	0	2.858012	-4.708191	1.896089
40	1	0	3.448032	-3.049891	2.079905
41	1	0	2.490371	-3.801401	3.364805
42	6	0	-5.231136	0.536701	-2.373689
43	1	0	-4.814108	1.407396	-2.885136
44	1	0	-5.700863	-0.106370	-3.124292
45	1	0	-6.021611	0.886004	-1.701499
46	6	0	-2.604892	3.714836	2.280012
47	1	0	-2.857916	4.708256	1.896245
48	1	0	-3.448050	3.049967	2.079801
49	1	0	-2.490394	3.801232	3.364845
50	6	0	5.231129	-0.536729	-2.373684
51	1	0	4.813971	-1.407039	-2.885681
52	1	0	5.701372	0.106515	-3.123811
53	1	0	6.021224	-0.886641	-1.701361
54	1	0	-0.000005	-0.000002	-2.150122

3, M06-2X/6-31+G(d,p), benzene IEFPCM:
Sum of electronic and thermal Free Energies= -874.969511

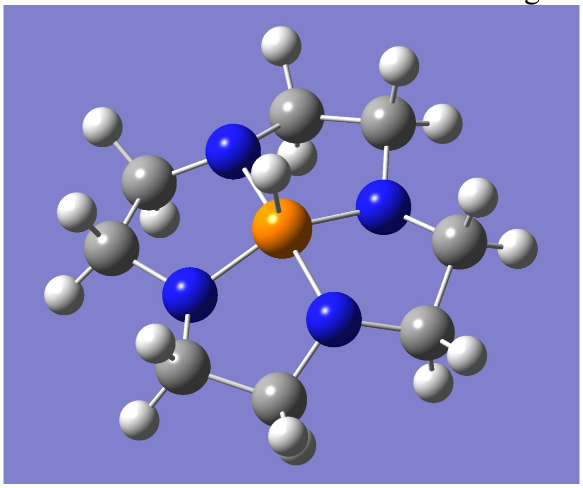

Center Number	Atomic Number	Atomic Type	Coordinates (Angstroms) X	Y	Z
1	6	0	2.907682	3.082855	-0.423339

2	6	0	3.260127	4.524241	-0.050238
3	6	0	1.883778	6.621692	0.186329
4	6	0	0.392759	6.915375	0.008327
5	1	0	3.695946	2.386904	-0.113197
6	1	0	3.740535	4.568474	0.941499
7	1	0	2.219015	6.886359	1.203122
8	1	0	2.490684	7.180841	-0.533504
9	1	0	0.166839	7.013460	-1.068520
10	6	0	-0.570190	1.988263	0.153972
11	6	0	0.863644	1.728440	-0.312731
12	6	0	-1.652030	5.561952	0.119385
13	6	0	-1.946037	4.085165	0.390843
14	1	0	-1.299164	1.502887	-0.503427
15	1	0	-0.724870	1.614356	1.179937
16	1	0	1.219996	0.747319	0.022474
17	1	0	-2.366058	6.212218	0.638240
18	1	0	-2.749246	3.711875	-0.253083
19	15	0	0.694152	4.282089	0.588936
20	1	0	0.818225	4.205551	1.989873
21	7	0	-0.696917	3.426101	0.085068
22	7	0	-0.294393	5.774357	0.577752
23	7	0	1.983149	5.200549	-0.055573
24	7	0	1.651206	2.809416	0.243657
25	1	0	0.896087	1.745877	-1.416764
26	1	0	-2.242449	3.928993	1.441614
27	1	0	-1.736326	5.756896	-0.964629
28	1	0	0.109488	7.852351	0.502038
29	1	0	3.939448	4.972806	-0.782683
30	1	0	2.795699	3.007188	-1.519721

4, M06-2X/6-31+G(d,p), benzene IEFPCM:
Sum of electronic and thermal Free Energies= -1113.987522

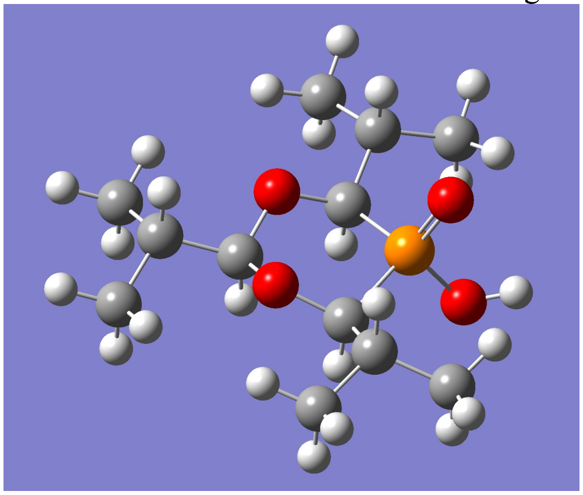

Center Number	Atomic Number	Atomic Type	Coordinates (Angstroms) X	Y	Z
1	6	0	-1.424486	-0.947325	-0.144965
2	6	0	-1.476458	1.553327	1.245628
3	6	0	-3.343943	0.147464	0.758139
4	1	0	-1.316502	-1.546907	0.777522
5	1	0	-1.370833	1.091993	2.244711
6	1	0	-3.101788	-0.374556	1.705393
7	15	0	-0.417517	0.581402	0.088645
8	8	0	-2.827208	1.455125	0.810990
9	8	0	-2.785209	-0.570123	-0.315464
10	8	0	0.038384	1.279324	-1.147900
11	8	0	0.793222	0.072394	1.052302
12	1	0	1.662540	0.279690	0.681104
13	6	0	-1.101039	3.033164	1.317346
14	6	0	0.365485	3.218460	1.714384
15	6	0	-2.028831	3.753589	2.297274
16	1	0	-1.249348	3.444699	0.311373
17	1	0	1.046176	2.850340	0.941748
18	1	0	0.576869	4.281348	1.859762
19	1	0	0.587973	2.698376	2.653234
20	1	0	-3.077070	3.624123	2.020302
21	1	0	-1.887998	3.364805	3.313129
22	1	0	-1.803923	4.823986	2.312133
23	6	0	-4.848414	0.230194	0.555013
24	6	0	-5.491825	1.029391	1.688253
25	6	0	-5.448171	-1.172459	0.456613
26	1	0	-5.000130	0.758384	-0.394893
27	1	0	-5.093432	2.044783	1.737102
28	1	0	-6.573980	1.086838	1.541663
29	1	0	-5.311389	0.541737	2.654094
30	1	0	-5.020978	-1.732304	-0.377729
31	1	0	-5.264144	-1.735864	1.379587
32	1	0	-6.530782	-1.109933	0.315425
33	6	0	-1.001193	-1.779757	-1.354948
34	6	0	-1.886282	-3.023091	-1.459678
35	6	0	0.478092	-2.165521	-1.278448
36	1	0	-1.157076	-1.150743	-2.240040
37	1	0	-2.943491	-2.754797	-1.511007
38	1	0	-1.629681	-3.594488	-2.356445
39	1	0	-1.735482	-3.673964	-0.589848
40	1	0	1.131940	-1.293699	-1.370481
41	1	0	0.704044	-2.675011	-0.334603
42	1	0	0.723948	-2.847980	-2.096588

5, M06-2X/6-31+G(d,p), chloroform IEFPCM:
Sum of electronic and thermal Free Energies= -2108.041657

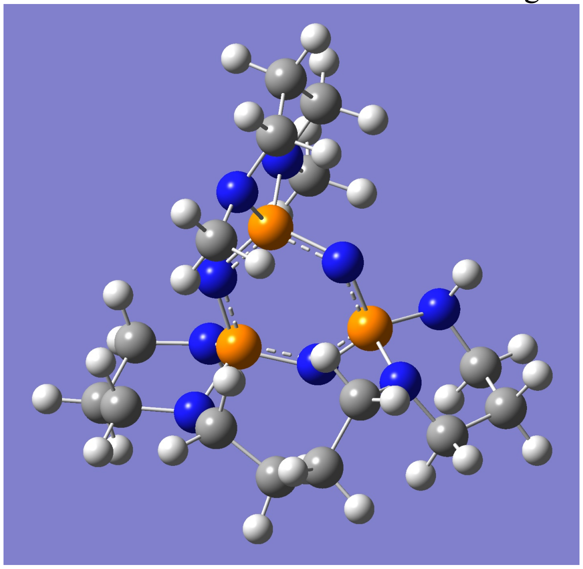

Center Number	Atomic Number	Atomic Type	Coordinates (Angstroms) X	Y	Z
1	15	0	1.756619	-0.062426	0.189419
2	15	0	-0.445280	1.467533	-0.656338
3	15	0	-0.687823	-1.324456	-0.459139
4	7	0	1.035973	1.352171	-0.011280
5	7	0	-1.173149	0.102819	-1.057932
6	7	0	0.750510	-1.345551	0.245575
7	7	0	2.954815	-0.292432	-0.985453
8	7	0	2.680578	-0.023243	1.595759
9	7	0	-0.332267	2.460699	-2.006485
10	1	0	-1.189309	2.413087	-2.553879
11	7	0	-1.436949	2.360416	0.401003
12	7	0	-1.808683	-1.991058	0.615798
13	7	0	-0.722845	-2.406908	-1.751554
14	1	0	-0.170563	-3.228360	-1.511213
15	6	0	2.414864	-0.306385	-2.347076
16	1	0	3.249646	-0.270709	-3.052601
17	1	0	1.788048	0.575015	-2.507656
18	1	0	1.816680	-1.208739	-2.548969
19	6	0	3.848611	-1.430415	-0.735025
20	1	0	4.580525	-1.455167	-1.549435
21	1	0	3.289041	-2.382025	-0.764538
22	6	0	4.564826	-1.275221	0.600122
23	1	0	5.188382	-0.375888	0.568371
24	1	0	5.218202	-2.138458	0.761180
25	6	0	3.591214	-1.165207	1.766551

26	1	0	3.021623	-2.102721	1.884724
27	1	0	4.144709	-0.998148	2.696431
28	6	0	1.921091	0.267501	2.809994
29	1	0	1.288103	-0.579360	3.118071
30	1	0	1.295567	1.147263	2.647022
31	1	0	2.625284	0.487462	3.617279
32	6	0	0.055389	3.849833	-1.699570
33	1	0	0.091452	4.408291	-2.638244
34	1	0	1.072133	3.819879	-1.293405
35	6	0	-0.889800	4.516723	-0.697961
36	1	0	-0.544997	5.535335	-0.489733
37	1	0	-1.893836	4.584854	-1.134096
38	6	0	-0.971997	3.742938	0.617490
39	1	0	0.008445	3.756678	1.123498
40	1	0	-1.693530	4.228990	1.282051
41	6	0	-1.761688	1.625288	1.635494
42	1	0	-1.943874	2.360691	2.426495
43	1	0	-0.894065	1.033738	1.971345
44	6	0	-2.992872	0.724101	1.462412
45	1	0	-3.895842	1.307927	1.672666
46	1	0	-3.048442	0.420927	0.414341
47	6	0	-2.970142	-0.523949	2.346365
48	1	0	-3.944316	-1.022607	2.277533
49	1	0	-2.847628	-0.246229	3.402190
50	6	0	-1.861582	-1.534902	2.008483
51	1	0	-0.874916	-1.132232	2.253687
52	1	0	-2.002045	-2.421158	2.642735
53	6	0	-3.083700	-2.481829	0.076936
54	1	0	-3.740276	-1.646900	-0.219864
55	1	0	-3.591683	-3.015848	0.888392
56	6	0	-2.896849	-3.426511	-1.108588
57	1	0	-3.882795	-3.710143	-1.492210
58	1	0	-2.394329	-4.340010	-0.767864
59	6	0	-2.067151	-2.782763	-2.218609
60	1	0	-1.958235	-3.456799	-3.072296
61	1	0	-2.557071	-1.872002	-2.581269

6, M06-2X/6-31+G(d,p), chloroform IEFPCM:
Sum of electronic and thermal Free Energies= -922.709921

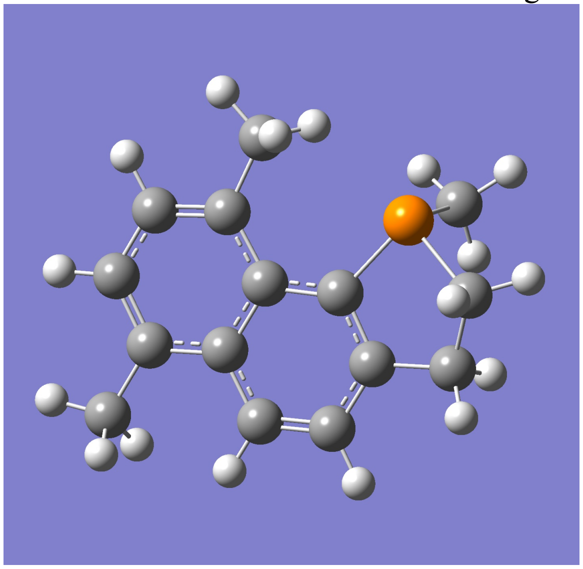

Center Number	Atomic Number	Atomic Type	Coordinates (Angstroms) X	Y	Z
1	6	0	-4.321209	-0.903282	-0.167070
2	6	0	-2.948278	-0.937247	-0.264264
3	6	0	-2.212976	0.296011	-0.141252
4	6	0	-2.958402	1.507038	0.074152
5	6	0	-4.389337	1.492672	0.161789
6	6	0	-5.041263	0.292215	0.041876
7	1	0	-4.874151	-1.834437	-0.257548
8	6	0	-0.780554	0.400191	-0.216512
9	6	0	-2.268905	2.743203	0.202347
10	1	0	-6.125366	0.255929	0.108007
11	6	0	-0.902726	2.810023	0.131362
12	6	0	-0.155889	1.631892	-0.077878
13	1	0	-2.838744	3.652572	0.357889
14	1	0	-0.390320	3.763102	0.233085
15	6	0	-2.302100	-2.278978	-0.508458
16	1	0	-1.762126	-2.307584	-1.457397
17	1	0	-1.590214	-2.548492	0.277249
18	1	0	-3.072578	-3.053173	-0.531314
19	6	0	-5.168314	2.764206	0.385252
20	1	0	-4.888679	3.249363	1.326282
21	1	0	-5.000089	3.487704	-0.419290
22	1	0	-6.237652	2.547013	0.423853
23	6	0	1.354280	1.640657	-0.118606
24	1	0	1.740061	1.699824	0.907658
25	1	0	1.729920	2.524768	-0.644082

26	6	0	1.798196	0.340688	-0.793628
27	1	0	2.775908	-0.012536	-0.453702
28	1	0	1.851222	0.473844	-1.878744
29	15	0	0.491940	-0.943591	-0.462206
30	6	0	0.861191	-1.304167	1.330373
31	1	0	1.761079	-1.922741	1.390233
32	1	0	1.008633	-0.390280	1.913288
33	1	0	0.032803	-1.865680	1.770423

7, M06-2X/6-31+G(d,p), chloroform IEFPCM:
Sum of electronic and thermal Free Energies= -2479.445640

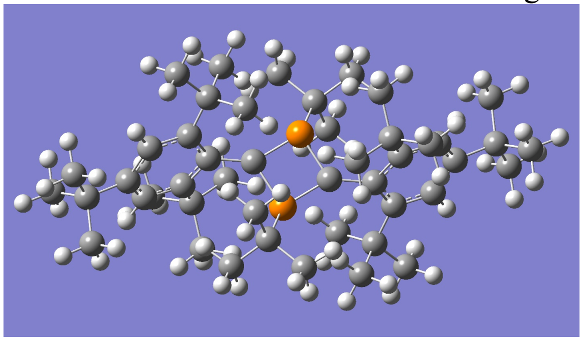

Center Number	Atomic Number	Atomic Type	Coordinates (Angstroms) X	Y	Z
1	6	0	-1.166233	0.073158	0.472704
2	6	0	-2.630719	0.176960	0.673425
3	6	0	-3.359319	-1.043553	0.823791
4	6	0	-4.700595	-1.074410	0.419942
5	1	0	-5.214822	-2.030124	0.416453
6	6	0	-5.403424	0.052931	0.018792
7	6	0	-4.741636	1.274673	0.153307
8	1	0	-5.298052	2.180014	-0.039895
9	6	0	-3.389490	1.384034	0.487730
10	6	0	-2.839793	-2.371841	1.443234
11	6	0	-1.557768	-2.239945	2.273202
12	1	0	-1.644240	-1.431393	3.006011
13	1	0	-0.685954	-2.048677	1.654943
14	1	0	-1.390876	-3.176466	2.817826
15	6	0	-2.618593	-3.455109	0.373440
16	1	0	-1.762454	-3.208020	-0.261595
17	1	0	-3.500465	-3.574835	-0.265690
18	1	0	-2.413735	-4.417346	0.857617
19	6	0	-3.911071	-2.888961	2.436432
20	1	0	-3.517184	-3.761254	2.967651
21	1	0	-4.836974	-3.200714	1.948493

22	1	0	-4.158199	-2.119326	3.175239
23	6	0	-6.842585	-0.073718	-0.486201
24	6	0	-6.851721	-0.971772	-1.736541
25	1	0	-6.462647	-1.970438	-1.514400
26	1	0	-6.232669	-0.536066	-2.528332
27	1	0	-7.873238	-1.082322	-2.117414
28	6	0	-7.725047	-0.708771	0.603105
29	1	0	-7.727423	-0.090238	1.506655
30	1	0	-7.371620	-1.707496	0.877233
31	1	0	-8.756824	-0.802564	0.246251
32	6	0	-7.446413	1.283762	-0.863802
33	1	0	-8.462646	1.137047	-1.243469
34	1	0	-6.863090	1.780075	-1.646637
35	1	0	-7.506585	1.954193	0.000137
36	6	0	-2.821604	2.812083	0.681363
37	6	0	-3.925987	3.884794	0.721689
38	1	0	-4.682018	3.666739	1.482926
39	1	0	-4.425244	4.009314	-0.244210
40	1	0	-3.462693	4.844331	0.973458
41	6	0	-2.130787	2.875112	2.051395
42	1	0	-1.344590	2.131690	2.135392
43	1	0	-2.853344	2.685232	2.853219
44	1	0	-1.683331	3.863020	2.211636
45	6	0	-1.856506	3.240624	-0.438048
46	1	0	-1.419727	4.215295	-0.189234
47	1	0	-2.395252	3.343343	-1.385960
48	1	0	-1.043787	2.532098	-0.579803
49	6	0	-1.224810	-0.574883	-2.591948
50	6	0	-2.384468	-1.582759	-2.634368
51	1	0	-2.876309	-1.505216	-3.612011
52	1	0	-3.132340	-1.377283	-1.861184
53	1	0	-2.031020	-2.611321	-2.512983
54	6	0	-1.788792	0.832381	-2.799802
55	1	0	-1.004446	1.592167	-2.814095
56	1	0	-2.513128	1.085416	-2.021454
57	1	0	-2.304291	0.858484	-3.768959
58	6	0	-0.229158	-0.912760	-3.708527
59	1	0	0.703077	-0.347508	-3.623210
60	1	0	-0.693591	-0.657024	-4.668470
61	1	0	0.011062	-1.978648	-3.732018
62	15	0	-0.399513	-0.756066	-0.884957
63	6	0	1.166230	-0.073171	-0.472688
64	6	0	2.630718	-0.176962	-0.673415
65	6	0	3.359310	1.043557	-0.823783
66	6	0	4.700588	1.074420	-0.419946
67	1	0	5.214812	2.030136	-0.416461

68	6	0	5.403428	-0.052918	-0.018805
69	6	0	4.741646	-1.274663	-0.153318
70	1	0	5.298069	-2.180000	0.039877
71	6	0	3.389498	-1.384031	-0.487731
72	6	0	2.839767	2.371846	-1.443214
73	6	0	1.557772	2.239923	-2.273222
74	1	0	1.644311	1.431411	-3.006069
75	1	0	0.685952	2.048576	-1.654998
76	1	0	1.390845	3.176461	-2.817807
77	6	0	2.618508	3.455090	-0.373408
78	1	0	1.762356	3.207972	0.261599
79	1	0	3.500360	3.574823	0.265750
80	1	0	2.413644	4.417330	-0.857574
81	6	0	3.911056	2.889022	-2.436372
82	1	0	3.517143	3.761297	-2.967601
83	1	0	4.836925	3.200822	-1.948399
84	1	0	4.158251	2.119403	-3.175174
85	6	0	6.842595	0.073737	0.486170
86	6	0	6.851746	0.971792	1.736508
87	1	0	6.462666	1.970457	1.514372
88	1	0	6.232706	0.536085	2.528308
89	1	0	7.873267	1.082345	2.117368
90	6	0	7.725040	0.708792	-0.603150
91	1	0	7.727405	0.090257	-1.506699
92	1	0	7.371606	1.707514	-0.877275
93	1	0	8.756822	0.802588	-0.246311
94	6	0	7.446432	-1.283740	0.863764
95	1	0	8.462671	-1.137022	1.243416
96	1	0	6.863122	-1.780054	1.646607
97	1	0	7.506595	-1.954173	-0.000176
98	6	0	2.821623	-2.812085	-0.681368
99	6	0	3.926016	-3.884785	-0.721708
100	1	0	4.682037	-3.666723	-1.482954
101	1	0	4.425285	-4.009302	0.244185
102	1	0	3.462727	-4.844326	-0.973471
103	6	0	2.130795	-2.875114	-2.051395
104	1	0	1.344557	-2.131732	-2.135363
105	1	0	2.853333	-2.685178	-2.853223
106	1	0	1.683388	-3.863042	-2.211655
107	6	0	1.856536	-3.240639	0.438046
108	1	0	1.419755	-4.215307	0.189226
109	1	0	2.395290	-3.343367	1.385953
110	1	0	1.043819	-2.532112	0.579814
111	6	0	1.224807	0.574866	2.591963
112	6	0	2.384482	1.582722	2.634373
113	1	0	2.876326	1.505174	3.612015

114	1	0	3.132347	1.377228	1.861187
115	1	0	2.031053	2.611289	2.512985
116	6	0	1.788764	-0.832407	2.799824
117	1	0	1.004406	-1.592181	2.814109
118	1	0	2.513103	-1.085452	2.021481
119	1	0	2.304255	-0.858518	3.768985
120	6	0	0.229164	0.912769	3.708542
121	1	0	-0.703077	0.347526	3.623241
122	1	0	0.693602	0.657043	4.668486
123	1	0	-0.011044	1.978660	3.732019
124	15	0	0.399509	0.756041	0.884973

8, M06-2X/6-31+G(d,p), chloroform IEFPCM:
Sum of electronic and thermal Free Energies= -1381.236674

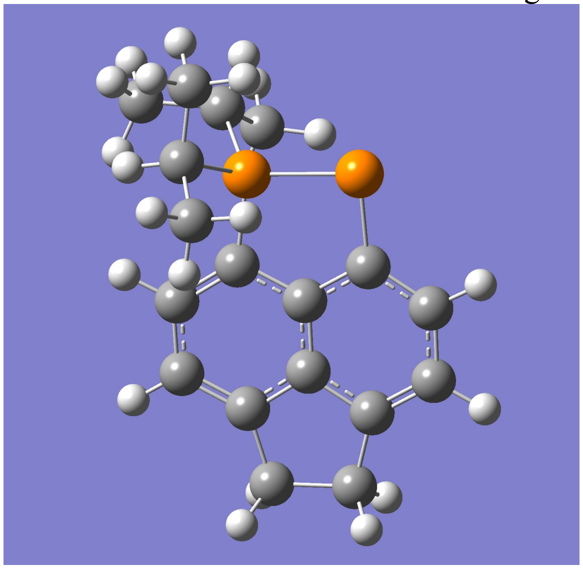

Center Number	Atomic Number	Atomic Type	Coordinates (Angstroms) X	Y	Z
1	15	0	8.561116	0.065420	12.007881
2	15	0	9.222207	-1.699672	10.962125
3	6	0	6.822101	0.133148	11.505240
4	6	0	5.811763	1.010271	11.865190
5	6	0	4.496216	0.875386	11.335237
6	6	0	4.223278	-0.148624	10.450661
7	6	0	5.256734	-1.038448	10.104280
8	6	0	4.854088	-2.053304	9.212220
9	6	0	5.805937	-2.974243	8.840284
10	6	0	7.137051	-2.883120	9.355919
11	6	0	7.542503	-1.885925	10.236280
12	6	0	6.550045	-0.929300	10.610123

13	6	0	2.965869	-0.579527	9.716766
14	6	0	3.384147	-1.844752	8.890405
15	6	0	8.641442	0.006226	13.853600
16	6	0	7.809383	-1.160858	14.385707
17	6	0	10.101337	-0.086949	14.307167
18	6	0	9.418726	1.626624	11.485991
19	6	0	9.087545	2.831163	12.370609
20	6	0	9.098492	1.910253	10.015988
21	1	0	6.004164	1.815475	12.569357
22	1	0	3.729806	1.581539	11.640742
23	1	0	5.570235	-3.786447	8.157339
24	1	0	7.853579	-3.637465	9.040200
25	1	0	2.161783	-0.809674	10.422186
26	1	0	2.599732	0.220640	9.066475
27	1	0	2.785045	-2.715552	9.173293
28	1	0	3.227980	-1.684182	7.819323
29	1	0	8.201220	0.949756	14.201674
30	1	0	7.852628	-1.167835	15.479151
31	1	0	8.203967	-2.112414	14.016290
32	1	0	6.761393	-1.083043	14.082959
33	1	0	10.561501	-1.003087	13.920368
34	1	0	10.146289	-0.124068	15.399586
35	1	0	10.699943	0.766570	13.974910
36	1	0	10.486519	1.386612	11.574854
37	1	0	8.014512	3.050020	12.353314
38	1	0	9.605983	3.713844	11.983468
39	1	0	9.398185	2.692627	13.409167
40	1	0	9.720013	2.737012	9.659921
41	1	0	8.047980	2.197116	9.899593
42	1	0	9.290561	1.035489	9.388657

9, M06-2X/6-31+G(d,p), chloroform IEFPCM:
Sum of electronic and thermal Free Energies= -3369.951591

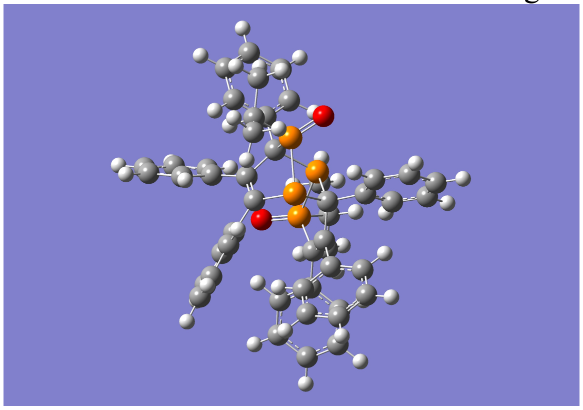

Center Number	Atomic Number	Atomic Type	Coordinates (Angstroms) X	Y	Z
1	15	0	0.642896	1.112329	1.392179
2	15	0	-0.452008	-0.708087	2.058494
3	8	0	0.134366	-2.087294	1.879570
4	6	0	-1.949971	-0.404367	1.073453
5	6	0	-1.898512	0.605221	0.179095
6	6	0	-0.668312	1.518729	0.072859
7	15	0	0.036917	1.223111	-1.689165
8	15	0	2.055145	1.845894	-1.030208
9	8	0	2.207130	3.232562	-0.472859
10	6	0	2.060950	0.465017	0.260530
11	6	0	1.623415	-0.733611	-0.566205
12	6	0	0.602194	-0.514549	-1.443628
13	6	0	-0.798751	-0.348023	3.827094
14	1	0	-1.187642	0.678316	3.868496
15	6	0	-1.831112	-1.323310	4.405792
16	1	0	-1.920914	-1.153901	5.483433
17	1	0	-1.510306	-2.358306	4.249396
18	1	0	-2.818848	-1.198670	3.956093
19	6	0	0.525213	-0.428259	4.596714
20	1	0	0.353989	-0.189727	5.650437
21	1	0	1.271670	0.273374	4.210194
22	1	0	0.939100	-1.439345	4.535427
23	6	0	-3.118760	-1.297115	1.286560
24	6	0	-4.389107	-0.749296	1.515803
25	1	0	-4.512426	0.330397	1.507890
26	6	0	-5.482266	-1.575923	1.756037
27	1	0	-6.459474	-1.137791	1.933440
28	6	0	-5.321951	-2.962918	1.771435
29	1	0	-6.174953	-3.607737	1.957974
30	6	0	-4.060773	-3.514573	1.551697
31	1	0	-3.927633	-4.591928	1.566406
32	6	0	-2.961960	-2.688902	1.316574
33	1	0	-1.976433	-3.117568	1.163648
34	6	0	-2.993258	0.796379	-0.830212
35	6	0	-3.713792	1.988643	-0.988373
36	1	0	-3.521817	2.839248	-0.345412
37	6	0	-4.711536	2.086826	-1.958133
38	1	0	-5.265402	3.015108	-2.057762
39	6	0	-4.999415	1.007350	-2.790960
40	1	0	-5.772524	1.092081	-3.548246
41	6	0	-4.290655	-0.183851	-2.638678
42	1	0	-4.503674	-1.036582	-3.276133

43	6	0	-3.303820	-0.290104	-1.663205
44	1	0	-2.766477	-1.226613	-1.542466
45	6	0	-1.094927	2.975173	0.296976
46	6	0	-1.760071	3.291492	1.490537
47	1	0	-1.941314	2.511438	2.227302
48	6	0	-2.218752	4.580394	1.737701
49	1	0	-2.737254	4.798514	2.666018
50	6	0	-2.015602	5.586857	0.790923
51	1	0	-2.374698	6.593876	0.977153
52	6	0	-1.341369	5.288022	-0.389138
53	1	0	-1.167139	6.062389	-1.129326
54	6	0	-0.882167	3.992151	-0.633052
55	1	0	-0.359281	3.793156	-1.562832
56	6	0	3.277405	1.447623	-2.358540
57	1	0	3.487931	0.374096	-2.304082
58	6	0	4.556454	2.254963	-2.106447
59	1	0	5.302834	1.984412	-2.859650
60	1	0	4.979362	2.066847	-1.116654
61	1	0	4.350614	3.325474	-2.187872
62	6	0	2.683166	1.766691	-3.736487
63	1	0	3.459000	1.649565	-4.499106
64	1	0	2.320400	2.799286	-3.782811
65	1	0	1.855299	1.098795	-3.991806
66	6	0	3.413308	0.434698	0.963439
67	6	0	4.529629	-0.148351	0.339496
68	1	0	4.425114	-0.641044	-0.620581
69	6	0	5.787429	-0.121312	0.933110
70	1	0	6.624831	-0.586785	0.422731
71	6	0	5.971650	0.495774	2.169913
72	1	0	6.951880	0.513630	2.635281
73	6	0	4.881722	1.093141	2.794770
74	1	0	5.003273	1.589330	3.752403
75	6	0	3.621304	1.066085	2.196986
76	1	0	2.799945	1.560025	2.703343
77	6	0	2.346690	-2.033622	-0.498506
78	6	0	2.740997	-2.639767	-1.700563
79	1	0	2.469607	-2.175360	-2.645216
80	6	0	3.481380	-3.818800	-1.696276
81	1	0	3.779636	-4.270993	-2.636951
82	6	0	3.840208	-4.410773	-0.485198
83	1	0	4.417293	-5.330254	-0.476977
84	6	0	3.460098	-3.810056	0.715323
85	1	0	3.739980	-4.261763	1.661893
86	6	0	2.727804	-2.625876	0.711054
87	1	0	2.432416	-2.167358	1.647056
88	6	0	-0.153555	-1.578943	-2.146328

89	6	0	-0.580665	-1.423386	-3.470910
90	1	0	-0.303377	-0.530845	-4.026098
91	6	0	-1.376871	-2.393086	-4.077757
92	1	0	-1.700935	-2.256824	-5.104881
93	6	0	-1.769978	-3.524396	-3.363213
94	1	0	-2.402823	-4.272221	-3.830687
95	6	0	-1.351403	-3.685769	-2.040270
96	1	0	-1.663854	-4.557427	-1.472712
97	6	0	-0.547452	-2.724030	-1.435594
98	1	0	-0.236715	-2.830723	-0.398121

10, M06-2X/6-31+G(d,p), chloroform IEFPCM:
Sum of electronic and thermal Free Energies= -1949.645049

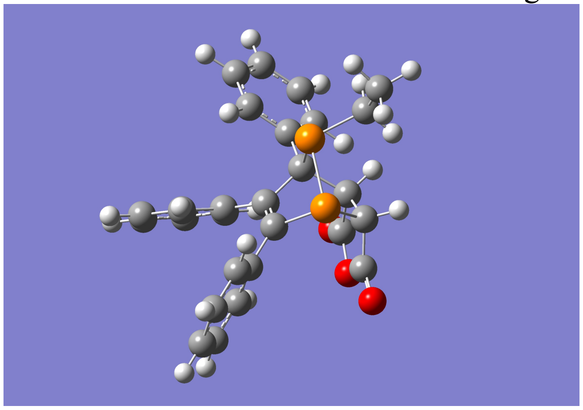

(mirror image of coordinates below shown for clarity)

Center Number	Atomic Number	Atomic Type	Coordinates (Angstroms) X	Y	Z
1	15	0	-0.862117	-2.296661	-0.707188
2	15	0	1.078318	-1.638581	-1.554306
3	8	0	-0.723644	-1.015006	2.825416
4	8	0	-2.192382	-2.622854	2.292073
5	8	0	1.095911	0.259605	3.060813
6	6	0	1.145108	-0.465778	-0.047885
7	6	0	-0.173360	0.301175	-0.246673
8	6	0	-1.248688	-0.485673	-0.509183
9	6	0	-0.101513	-2.422859	1.037033
10	6	0	-1.140892	-2.097648	2.075900
11	6	0	0.532132	-0.600355	2.449095
12	6	0	0.998454	-1.374413	1.226511
13	6	0	2.414344	0.362905	-0.110551
14	6	0	3.388759	0.354512	0.891069
15	1	0	3.244764	-0.215409	1.801451
16	6	0	4.551389	1.118859	0.763708
17	1	0	5.289300	1.099823	1.559510

18	6	0	4.758345	1.905831	-0.364336
19	1	0	5.659763	2.502719	-0.459044
20	6	0	3.791793	1.923572	-1.372037
21	1	0	3.935697	2.534939	-2.257253
22	6	0	2.638108	1.159263	-1.245891
23	1	0	1.891162	1.186068	-2.035168
24	6	0	-0.234046	1.786822	-0.196212
25	6	0	0.391491	2.531585	0.811953
26	1	0	0.941553	2.029004	1.600481
27	6	0	0.314058	3.923054	0.812217
28	1	0	0.796581	4.484309	1.606112
29	6	0	-0.369420	4.591417	-0.202534
30	1	0	-0.421687	5.675618	-0.203443
31	6	0	-0.976655	3.858852	-1.223122
32	1	0	-1.500192	4.369271	-2.025368
33	6	0	-0.909669	2.468909	-1.218564
34	1	0	-1.378454	1.900015	-2.016585
35	6	0	-2.666128	-0.056087	-0.543064
36	6	0	-3.186389	0.773901	0.460476
37	1	0	-2.537896	1.109665	1.265140
38	6	0	-4.521845	1.164107	0.431830
39	1	0	-4.911317	1.802646	1.218379
40	6	0	-5.357805	0.732344	-0.598995
41	1	0	-6.398929	1.038282	-0.620697
42	6	0	-4.853035	-0.104232	-1.594087
43	1	0	-5.498493	-0.450419	-2.395114
44	6	0	-3.518966	-0.504230	-1.559836
45	1	0	-3.131188	-1.161775	-2.333803
46	6	0	2.272636	-2.996795	-1.119273
47	1	0	3.262240	-2.524330	-1.084972
48	1	0	2.084188	-3.425339	-0.130426
49	6	0	2.242577	-4.095860	-2.186614
50	1	0	1.258574	-4.572413	-2.235774
51	1	0	0.234816	-3.451512	1.191917
52	1	0	1.962761	-1.832747	1.459441
53	1	0	2.978534	-4.870818	-1.957916
54	1	0	2.471434	-3.691987	-3.176483

----

11 isomer A, M06-2X/6-31+G(d,p), toluene IEFPCM:
Sum of electronic and thermal Free Energies= -1091.302278

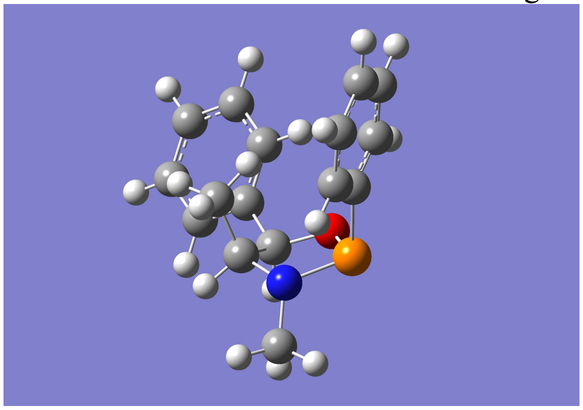

Center Number	Atomic Number	Atomic Type	Coordinates (Angstroms) X	Y	Z
1	6	0	0.445851	1.546183	0.734580
2	6	0	1.192485	1.058071	-0.541952
3	1	0	1.036756	2.342738	1.201523
4	1	0	1.659100	1.932843	-1.018991
5	15	0	-1.309173	1.273451	-1.165224
6	8	0	0.194135	0.549149	-1.430776
7	7	0	-0.803373	2.149901	0.227953
8	6	0	-0.743688	3.607415	0.078397
9	1	0	-1.655833	3.962090	-0.405326
10	1	0	-0.682533	4.067874	1.068467
11	1	0	0.116886	3.945371	-0.518298
12	6	0	2.254226	0.021763	-0.280182
13	6	0	1.975529	-1.343542	-0.364373
14	6	0	3.528057	0.437007	0.116030
15	6	0	2.956997	-2.280529	-0.045213
16	1	0	0.986901	-1.660920	-0.681458
17	6	0	4.508301	-0.499002	0.440774
18	1	0	3.754710	1.499737	0.169165
19	6	0	4.223915	-1.862048	0.361298
20	1	0	2.732066	-3.340394	-0.115052
21	1	0	5.494490	-0.164560	0.747620
22	1	0	4.986986	-2.593374	0.608515
23	6	0	0.195317	0.450353	1.767055
24	1	0	-0.258214	-0.436820	1.318331
25	1	0	1.137003	0.149098	2.234266
26	1	0	-0.481549	0.829367	2.536761
27	6	0	-2.165898	-0.167075	-0.398378
28	6	0	-3.089762	0.026463	0.632738
29	6	0	-1.973615	-1.452038	-0.916270
30	6	0	-3.790602	-1.056662	1.160839

31	1	0	-3.234926	1.022949	1.042598
32	6	0	-2.673986	-2.535616	-0.387121
33	1	0	-1.261517	-1.606371	-1.723362
34	6	0	-3.581808	-2.339688	0.653701
35	1	0	-4.494956	-0.901071	1.972443
36	1	0	-2.510480	-3.532183	-0.786082
37	1	0	-4.125911	-3.183217	1.067002

----

11 isomer B, M06-2X/6-31+G(d,p), toluene IEFPCM:
Sum of electronic and thermal Free Energies= -1091.300589

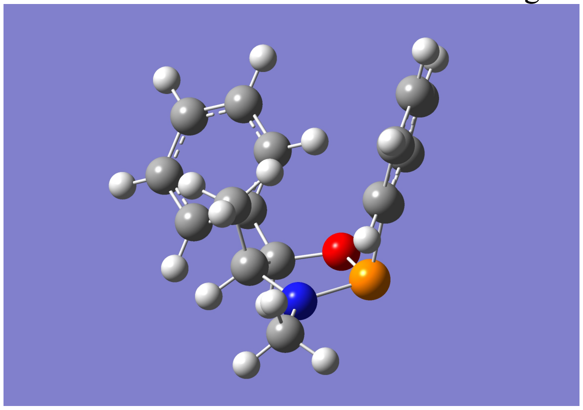

Center Number	Atomic Number	Atomic Type	Coordinates (Angstroms) X	Y	Z
1	6	0	-0.428891	-1.660668	0.643797
2	6	0	-1.197625	-1.146592	-0.598342
3	1	0	-0.978425	-2.518101	1.056367
4	1	0	-1.634033	-2.019270	-1.101668
5	15	0	1.321275	-1.194069	-1.264228
6	8	0	-0.223568	-0.575073	-1.484735
7	7	0	0.796674	-2.167035	0.035805
8	6	0	-2.291078	-0.156444	-0.291503
9	6	0	-2.055746	1.219276	-0.284390
10	6	0	-3.557883	-0.634603	0.053267
11	6	0	-3.072982	2.103952	0.070013
12	1	0	-1.072651	1.588189	-0.558081
13	6	0	-4.574195	0.248176	0.414170
14	1	0	-3.750532	-1.705089	0.036026
15	6	0	-4.333162	1.621719	0.423046
16	1	0	-2.881053	3.172552	0.070151
17	1	0	-5.554569	-0.135399	0.679248
18	1	0	-5.124486	2.312231	0.697370
19	6	0	-0.213746	-0.624660	1.748496
20	1	0	0.201774	0.305609	1.351995
21	1	0	-1.158865	-0.393244	2.248161

22	1	0	0.483488	-1.019708	2.493912
23	6	0	2.081529	0.315943	-0.488800
24	6	0	3.115750	0.161984	0.440890
25	6	0	1.714905	1.604643	-0.888900
26	6	0	3.742951	1.276589	0.995740
27	1	0	3.428992	-0.834310	0.747740
28	6	0	2.344835	2.721785	-0.339878
29	1	0	0.927659	1.732181	-1.627564
30	6	0	3.354727	2.559652	0.608390
31	1	0	4.533272	1.145428	1.728710
32	1	0	2.047524	3.718594	-0.651946
33	1	0	3.842337	3.428790	1.038751
34	6	0	1.626963	-3.108062	0.755570
35	1	0	2.424820	-3.463615	0.096291
36	1	0	2.091849	-2.677157	1.653679
37	1	0	1.027488	-3.972943	1.062130

----

11 isomer C, M06-2X/6-31+G(d,p), toluene IEFPCM:
Sum of electronic and thermal Free Energies= -1091.298642

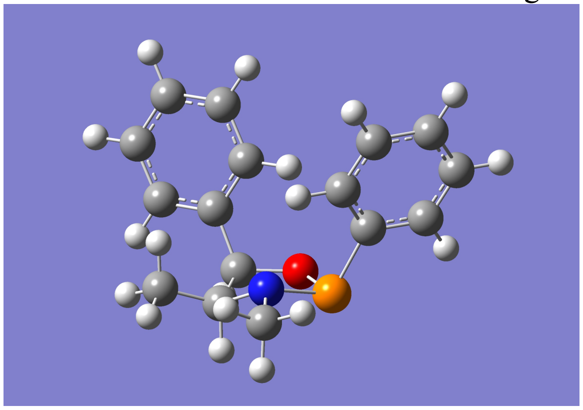

Center Number	Atomic Number	Atomic Type	Coordinates (Angstroms) X	Y	Z
1	6	0	0.338776	1.337698	0.330240
2	6	0	1.192985	0.114243	-0.093489
3	1	0	1.920536	0.445512	-0.843638
4	15	0	-1.329950	-0.414728	-0.561156
5	8	0	0.294990	-0.788429	-0.749255
6	7	0	-0.967167	0.768436	0.666681
7	6	0	1.948833	-0.566395	1.026385
8	6	0	3.235999	-0.123538	1.346544
9	6	0	1.394975	-1.618481	1.755902
10	6	0	3.943973	-0.695550	2.401290
11	1	0	3.690917	0.674026	0.762982
12	6	0	2.102553	-2.195962	2.809959

13	1	0	0.416282	-1.997130	1.485280
14	6	0	3.374908	-1.732480	3.140955
15	1	0	4.942162	-0.340188	2.637981
16	1	0	1.659063	-3.015290	3.367838
17	1	0	3.925924	-2.184656	3.959691
18	1	0	0.248030	1.990421	-0.560056
19	6	0	0.922259	2.158875	1.469959
20	1	0	0.952711	1.569238	2.390412
21	1	0	1.939918	2.478532	1.226968
22	1	0	0.325939	3.058686	1.640771
23	6	0	-2.020048	1.749656	0.905679
24	1	0	-2.978301	1.236610	1.029316
25	1	0	-1.818738	2.295156	1.831357
26	1	0	-2.116919	2.474161	0.080738
27	6	0	-1.991740	-1.775173	0.497265
28	6	0	-2.486322	-2.907446	-0.160446
29	6	0	-2.085052	-1.698523	1.891897
30	6	0	-3.028297	-3.967936	0.564353
31	1	0	-2.445043	-2.961912	-1.246622
32	6	0	-2.647019	-2.748378	2.616143
33	1	0	-1.698293	-0.817795	2.398079
34	6	0	-3.111359	-3.886150	1.954107
35	1	0	-3.394375	-4.849164	0.046930
36	1	0	-2.718414	-2.682507	3.697670
37	1	0	-3.543832	-4.705052	2.520864

----

12 isomer A, M06-2X/6-31+G(d,p), toluene IEFPCM:
Sum of electronic and thermal Free Energies= -1091.305029

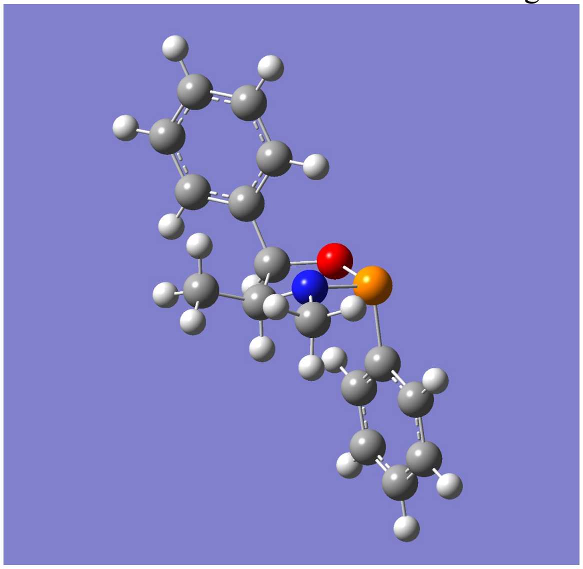

Center Number	Atomic Number	Atomic Type	Coordinates (Angstroms) X	Y	Z
1	6	0	-0.296150	0.485043	0.983929
2	6	0	-0.943126	-0.731889	0.274488
3	1	0	0.576588	0.117770	1.554955
4	1	0	-0.815955	-1.624207	0.899595
5	15	0	0.844230	0.290516	-1.335002
6	8	0	-0.208309	-0.938820	-0.944441
7	7	0	0.152317	1.322409	-0.129043
8	6	0	0.884738	2.526116	0.232752
9	1	0	1.242378	3.020831	-0.675257
10	1	0	0.224132	3.223353	0.754457
11	1	0	1.752125	2.311532	0.878296
12	6	0	2.382434	-0.335446	-0.507050
13	6	0	3.549354	0.422648	-0.661363
14	6	0	2.433744	-1.513460	0.246491
15	6	0	4.738618	0.031923	-0.047123
16	1	0	3.532549	1.324569	-1.271964
17	6	0	3.624659	-1.913427	0.851732
18	1	0	1.540930	-2.125463	0.343382
19	6	0	4.776007	-1.138088	0.711112
20	1	0	5.635637	0.631183	-0.169308
21	1	0	3.656087	-2.831425	1.430846
22	1	0	5.702627	-1.450722	1.182471
23	6	0	-2.411769	-0.558692	-0.038516
24	6	0	-2.831348	0.186155	-1.143906
25	6	0	-3.370135	-1.116010	0.809923
26	6	0	-4.189513	0.378616	-1.386658
27	1	0	-2.090626	0.610896	-1.814347
28	6	0	-4.730402	-0.921529	0.571449
29	1	0	-3.050941	-1.710198	1.663483
30	6	0	-5.142687	-0.171129	-0.528166
31	1	0	-4.505441	0.957088	-2.249332
32	1	0	-5.464956	-1.363072	1.237798
33	1	0	-6.200508	-0.022270	-0.721112
34	6	0	-1.225947	1.219729	1.939767
35	1	0	-2.042935	1.698326	1.392716
36	1	0	-1.656853	0.517706	2.660204
37	1	0	-0.677947	1.979037	2.503350

12 isomer B, M06-2X/6-31+G(d,p), toluene IEFPCM:
Sum of electronic and thermal Free Energies= -1091.303771

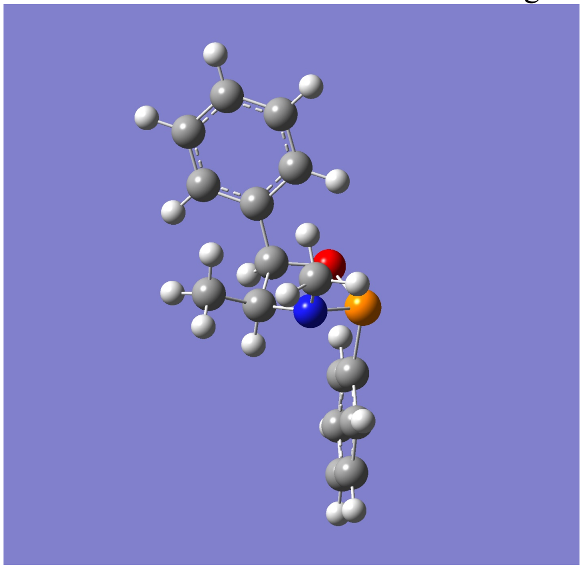

Center Number	Atomic Number	Atomic Type	Coordinates (Angstroms) X	Y	Z
1	6	0	-0.224905	0.598258	0.782397
2	6	0	-0.909509	-0.681706	0.181538
3	1	0	0.669267	0.238521	1.305176
4	1	0	-0.716076	-1.522673	0.859789
5	15	0	0.825397	0.261781	-1.515699
6	8	0	-0.255417	-0.949108	-1.063326
7	7	0	0.264483	1.391540	-0.359509
8	6	0	2.319656	-0.358332	-0.621541
9	6	0	3.350808	0.534698	-0.309449
10	6	0	2.478099	-1.715894	-0.328476
11	6	0	4.515444	0.080120	0.305628
12	1	0	3.234415	1.592746	-0.532906
13	6	0	3.646309	-2.172093	0.283857
14	1	0	1.678750	-2.410834	-0.571038
15	6	0	4.665003	-1.275571	0.602785
16	1	0	5.305979	0.781879	0.553643
17	1	0	3.758675	-3.227262	0.514212
18	1	0	5.573297	-1.630584	1.079764
19	6	0	-2.402210	-0.567115	-0.043498
20	6	0	-2.924377	-0.167834	-1.275516
21	6	0	-3.283217	-0.849509	1.004656
22	6	0	-4.300720	-0.022784	-1.446812
23	1	0	-2.247596	0.012707	-2.105587
24	6	0	-4.658800	-0.704850	0.837185
25	1	0	-2.889927	-1.189899	1.960096

26	6	0	-5.171761	-0.285093	-0.390199
27	1	0	-4.693148	0.286988	-2.410603
28	1	0	-5.329812	-0.928577	1.660788
29	1	0	-6.243261	-0.176490	-0.525504
30	6	0	-1.065830	1.386976	1.772472
31	1	0	-1.993384	1.755667	1.327421
32	1	0	-1.332598	0.749900	2.620517
33	1	0	-0.490625	2.234538	2.154325
34	6	0	-0.573538	2.494385	-0.843279
35	1	0	-0.583334	3.299590	-0.104073
36	1	0	-0.130129	2.888198	-1.760069
37	1	0	-1.609697	2.202685	-1.055102

13, M06-2X/6-31+G(d,p), chloroform IEFPCM:
Sum of electronic and thermal Free Energies= -1792.390827

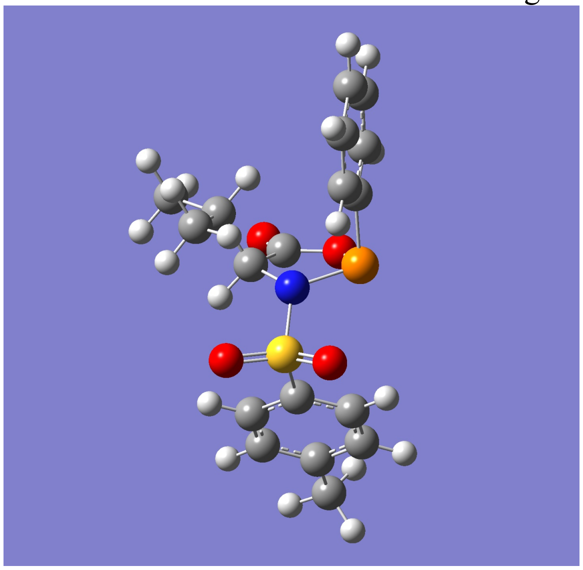

Center Number	Atomic Number	Atomic Type	Coordinates (Angstroms) X	Y	Z
1	15	0	0.676666	-1.392661	-0.358521
2	16	0	-0.966919	-0.230107	1.776293
3	7	0	0.287873	-0.030946	0.674975
4	8	0	0.305507	-0.491169	-1.751879
5	8	0	-0.023175	1.604059	-2.459905
6	8	0	-0.718257	-1.528506	2.388307
7	8	0	-1.009164	0.989176	2.570416
8	6	0	0.190004	0.850240	-1.551109
9	6	0	0.358703	1.234039	-0.075739
10	6	0	1.668229	2.025880	0.175704
11	6	0	1.919155	2.157371	1.676595

12	6	0	1.589010	3.404940	-0.483080
13	6	0	-5.948467	-0.532047	-1.664510
14	6	0	3.184080	-1.257781	0.839650
15	6	0	4.570672	-1.132121	0.862792
16	6	0	5.274566	-0.945422	-0.328613
17	6	0	4.592008	-0.889393	-1.542996
18	6	0	3.202604	-1.012545	-1.571609
19	6	0	2.497556	-1.182577	-0.377128
20	6	0	-3.194287	0.836954	0.590642
21	6	0	-4.327756	0.761793	-0.216122
22	6	0	-4.727715	-0.450075	-0.786970
23	6	0	-3.971645	-1.601993	-0.525333
24	6	0	-2.838068	-1.550664	0.275428
25	6	0	-2.458676	-0.322430	0.820928
26	1	0	-0.486917	1.875950	0.200361
27	1	0	2.498224	1.465045	-0.271421
28	1	0	2.838974	2.724854	1.844108
29	1	0	2.022283	1.180231	2.154245
30	1	0	1.090995	2.684931	2.161815
31	1	0	2.514192	3.954656	-0.289840
32	1	0	0.759931	3.983377	-0.058820
33	1	0	1.444377	3.342490	-1.563073
34	1	0	-6.459157	0.430676	-1.724858
35	1	0	-6.653369	-1.273847	-1.278381
36	1	0	-5.671907	-0.837770	-2.678006
37	1	0	2.634635	-1.389175	1.769596
38	1	0	5.100864	-1.175073	1.808701
39	1	0	6.354999	-0.844596	-0.308960
40	1	0	5.138612	-0.744953	-2.469360
41	1	0	2.670818	-0.954328	-2.516932
42	1	0	-2.896851	1.773638	1.051103
43	1	0	-4.911551	1.659061	-0.398162
44	1	0	-4.281272	-2.552338	-0.951018
45	1	0	-2.262873	-2.445736	0.490331

----

14 isomer A, M06-2X/6-31+G(d,p), chloroform IEFPCM:
Sum of electronic and thermal Free Energies= -1792.387462

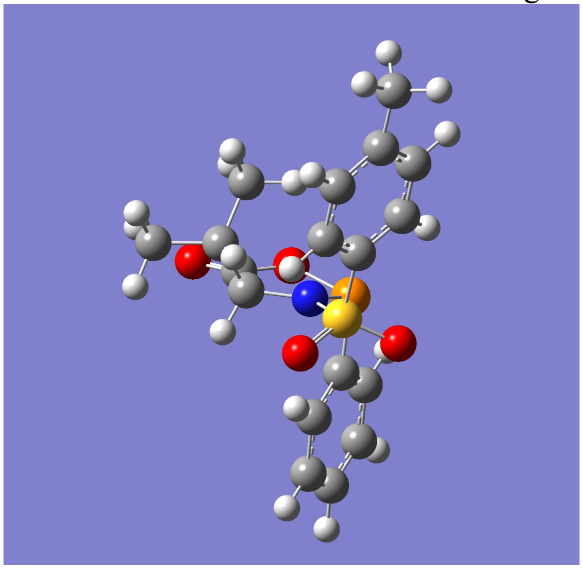

Center Number	Atomic Number	Atomic Type	Coordinates (Angstroms) X	Y	Z
1	15	0	-1.274853	-0.480192	-1.291271
2	16	0	0.509859	-1.048481	0.987888
3	7	0	-0.296353	0.059624	0.060100
4	8	0	-1.491927	1.153084	-1.728722
5	8	0	-1.565829	3.213043	-0.865823
6	8	0	0.453940	-0.598052	2.372801
7	8	0	-0.075896	-2.323788	0.578491
8	6	0	-1.227577	2.066742	-0.763157
9	6	0	-0.418519	1.482213	0.397373
10	6	0	0.942944	2.197967	0.542885
11	6	0	0.774366	3.610690	1.104208
12	6	0	1.728918	2.197076	-0.767742
13	6	0	6.254117	-0.909908	-0.938897
14	6	0	2.510583	-1.567363	-0.792413
15	6	0	3.825704	-1.517536	-1.233065
16	6	0	4.834393	-0.950241	-0.439056
17	6	0	4.494228	-0.437622	0.815669
18	6	0	3.178372	-0.474074	1.275142
19	6	0	2.202186	-1.031669	0.459058
20	1	0	-1.004204	1.615034	1.316406
21	1	0	1.494651	1.616211	1.289997
22	1	0	1.759312	4.036418	1.315143
23	1	0	0.201370	3.602762	2.036497
24	1	0	0.262877	4.262204	0.392105
25	1	0	2.741535	2.573002	-0.596449

26	1	0	1.250770	2.849661	-1.507439
27	1	0	1.811182	1.191857	-1.193494
28	1	0	6.631731	-1.922861	-1.107170
29	1	0	6.912512	-0.413070	-0.224036
30	1	0	6.312924	-0.375474	-1.891496
31	1	0	1.733947	-2.014727	-1.406002
32	1	0	4.079170	-1.928430	-2.206300
33	1	0	5.265915	-0.006228	1.446265
34	1	0	2.913736	-0.081776	2.251863
35	6	0	-2.898438	-0.758317	-0.480345
36	6	0	-3.967282	-1.091082	-1.321538
37	6	0	-3.104986	-0.675421	0.900256
38	6	0	-5.237314	-1.307576	-0.791344
39	1	0	-3.811242	-1.179738	-2.394682
40	6	0	-4.373823	-0.903607	1.430011
41	1	0	-2.279609	-0.447594	1.569395
42	6	0	-5.439697	-1.213818	0.585596
43	1	0	-6.063336	-1.557406	-1.449229
44	1	0	-4.528101	-0.842458	2.502449
45	1	0	-6.426398	-1.390935	1.001683

----

14 isomer B, M06-2X/6-31+G(d,p), chloroform IEFPCM:
Sum of electronic and thermal Free Energies= -1792.386982

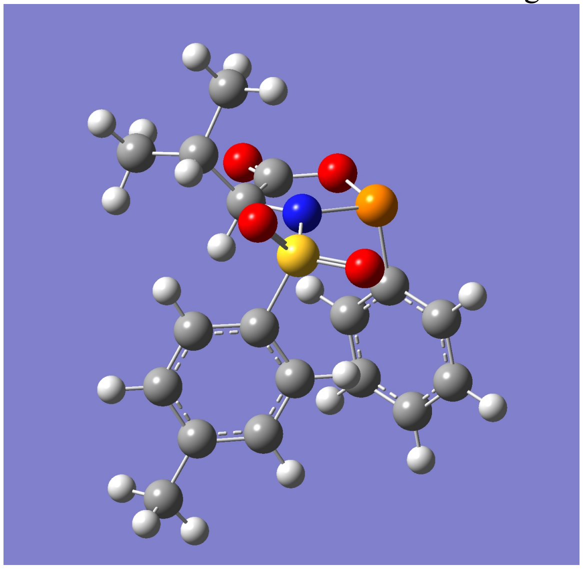

Center Number	Atomic Number	Atomic Type	Coordinates (Angstroms) X	Y	Z
1	15	0	1.390768	1.951742	-0.730527
2	16	0	0.345452	-0.598659	-1.808667
3	7	0	1.330783	0.175338	-0.722558

4	8	0	2.267511	1.874844	0.718774
5	8	0	2.790167	0.580992	2.460519
6	8	0	-0.103616	0.477980	-2.690795
7	8	0	1.043890	-1.753444	-2.356568
8	6	0	2.321732	0.682964	1.360699
9	6	0	1.762335	-0.464885	0.521965
10	6	0	2.816561	-1.574648	0.300723
11	6	0	3.064090	-2.371362	1.581876
12	6	0	4.106606	-1.001619	-0.282997
13	6	0	-4.267503	-2.679587	1.532395
14	6	0	-0.947572	-2.494252	-0.318143
15	6	0	-2.000444	-2.954178	0.464052
16	6	0	-3.125813	-2.153409	0.703494
17	6	0	-3.173057	-0.873521	0.141002
18	6	0	-2.126942	-0.390453	-0.640745
19	6	0	-1.023677	-1.210341	-0.860771
20	1	0	0.902648	-0.876049	1.074306
21	1	0	2.374852	-2.248045	-0.441642
22	1	0	3.713690	-3.222812	1.360838
23	1	0	2.126911	-2.759426	1.995439
24	1	0	3.547600	-1.758704	2.346412
25	1	0	4.794136	-1.811021	-0.541646
26	1	0	4.612956	-0.354570	0.443164
27	1	0	3.907219	-0.423419	-1.190280
28	1	0	-4.823526	-3.440532	0.975617
29	1	0	-4.962486	-1.881792	1.801210
30	1	0	-3.901068	-3.146453	2.450449
31	1	0	-0.084953	-3.122009	-0.520222
32	1	0	-1.952780	-3.952247	0.890433
33	1	0	-4.039318	-0.241659	0.315620
34	1	0	-2.170282	0.603462	-1.076465
35	6	0	-0.254552	2.397327	-0.053682
36	6	0	-1.113341	3.081452	-0.920295
37	6	0	-0.695548	2.050433	1.229355
38	6	0	-2.414132	3.390094	-0.521171
39	1	0	-0.771858	3.355167	-1.915780
40	6	0	-1.991352	2.363672	1.629157
41	1	0	-0.035286	1.543508	1.928323
42	6	0	-2.852536	3.026438	0.751319
43	1	0	-3.079935	3.912816	-1.200072
44	1	0	-2.331514	2.090435	2.622861
45	1	0	-3.863768	3.266188	1.064914

----

15 isomer A (cis, chair), M06-2X/6-31+G(d,p), benzene IEFPCM:
Sum of electronic and thermal Free Energies= -901.076471

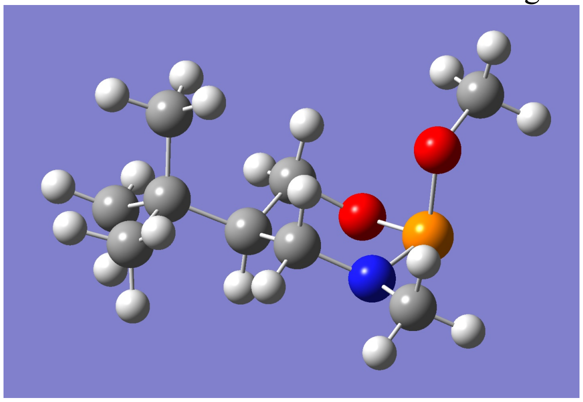

Center Number	Atomic Number	Atomic Type	Coordinates (Angstroms) X	Y	Z
1	6	0	0.221364	-1.209329	-0.510378
2	6	0	0.261954	1.176020	0.232302
3	6	0	1.059459	0.067874	-0.467114
4	1	0	-0.037118	-1.535695	0.504455
5	1	0	0.743693	-2.022184	-1.018760
6	1	0	0.810802	2.121057	0.165817
7	1	0	0.129461	0.944609	1.303123
8	1	0	1.205767	0.392661	-1.507779
9	6	0	2.469389	-0.169209	0.147659
10	6	0	2.397997	-0.929885	1.481284
11	1	0	3.395044	-0.978603	1.932183
12	1	0	1.732572	-0.436802	2.197803
13	1	0	2.049974	-1.958859	1.344964
14	6	0	3.321672	-0.980296	-0.841184
15	1	0	3.436696	-0.442796	-1.788812
16	1	0	4.320685	-1.148999	-0.425031
17	1	0	2.887510	-1.960936	-1.057099
18	6	0	3.178306	1.172818	0.383333
19	1	0	4.224823	0.996248	0.652917
20	1	0	3.162769	1.793503	-0.520260
21	1	0	2.720299	1.740913	1.199031
22	15	0	-2.077053	0.099827	-0.703504
23	8	0	-0.989303	-1.007421	-1.252817
24	7	0	-1.035420	1.405709	-0.410622
25	6	0	-1.690196	2.624593	0.050424
26	1	0	-1.063726	3.489524	-0.190522
27	1	0	-2.650548	2.745786	-0.459152
28	1	0	-1.871082	2.613426	1.135270
29	8	0	-2.266689	-0.429567	0.877097
30	6	0	-3.069056	-1.589633	1.060699

31	1	0	-2.527977	-2.490234	0.748497
32	1	0	-3.305203	-1.661251	2.123269
33	1	0	-4.003067	-1.521277	0.489113

----

15 isomer B (cis, twist 1), M06-2X/6-31+G(d,p), benzene IEFPCM:
Sum of electronic and thermal Free Energies= -901.069505

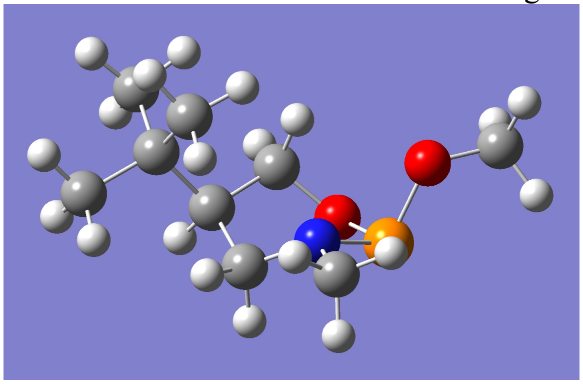

Center Number	Atomic Number	Atomic Type	Coordinates (Angstroms) X	Y	Z
1	6	0	-0.095261	-1.212479	-0.245660
2	6	0	0.149913	1.231395	0.233148
3	6	0	0.837440	-0.000760	-0.390433
4	1	0	0.031146	-1.693618	0.730490
5	1	0	0.085782	-1.952294	-1.027160
6	1	0	-0.411242	1.783261	-0.538917
7	1	0	0.897263	1.929772	0.622894
8	1	0	0.958987	0.198165	-1.464419
9	6	0	2.255588	-0.284141	0.177150
10	6	0	2.264154	-0.307417	1.712552
11	1	0	3.268066	-0.561033	2.070970
12	1	0	1.996413	0.668283	2.131189
13	1	0	1.561405	-1.040951	2.118145
14	6	0	2.760262	-1.636445	-0.350787
15	1	0	2.704544	-1.677465	-1.445130
16	1	0	3.805997	-1.785912	-0.061716
17	1	0	2.185239	-2.475625	0.053713
18	6	0	3.223371	0.802328	-0.312993
19	1	0	4.228766	0.619562	0.081552
20	1	0	3.284849	0.805609	-1.407059
21	1	0	2.920393	1.802806	0.012203
22	15	0	-2.140616	0.012138	0.871374
23	8	0	-1.466005	-0.808981	-0.387060
24	7	0	-0.712137	0.845516	1.355533
25	6	0	-0.930515	1.901654	2.335223

26	1	0	0.026781	2.174454	2.791872
27	1	0	-1.378990	2.808345	1.899306
28	1	0	-1.585346	1.535314	3.130342
29	8	0	-2.112558	-1.079598	2.120387
30	6	0	-3.279005	-1.880526	2.299408
31	1	0	-3.303535	-2.696622	1.570414
32	1	0	-3.230048	-2.298597	3.305345
33	1	0	-4.192096	-1.282784	2.200530

----

15 isomer C (cis, twist 2), M06-2X/6-31+G(d,p), benzene IEFPCM:
Sum of electronic and thermal Free Energies= -901.069472

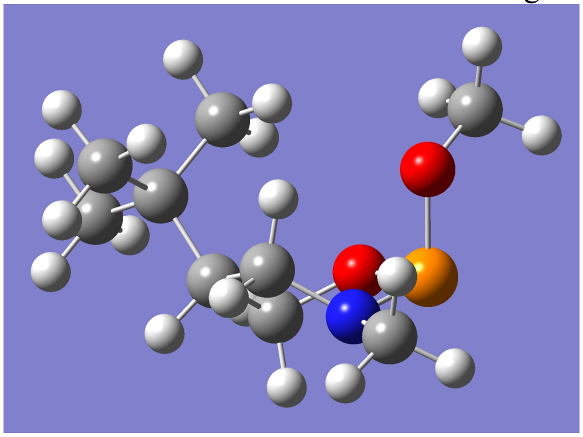

Center Number	Atomic Number	Atomic Type	Coordinates (Angstroms) X	Y	Z
1	6	0	0.756894	-1.203189	-1.635145
2	6	0	0.569365	1.251290	-1.063887
3	6	0	1.518833	0.026731	-1.129715
4	1	0	1.303328	-2.125488	-1.428277
5	1	0	0.622901	-1.126340	-2.719419
6	1	0	1.063648	2.125367	-1.500154
7	1	0	0.330160	1.504062	-0.022478
8	1	0	2.275533	0.233949	-1.900601
9	6	0	2.302314	-0.196115	0.192957
10	6	0	1.383711	-0.692987	1.318065
11	1	0	1.953899	-0.787320	2.249168
12	1	0	0.546809	-0.008893	1.493155
13	1	0	0.958384	-1.672124	1.074403
14	6	0	3.419916	-1.226029	-0.031343
15	1	0	4.089316	-0.910676	-0.840094
16	1	0	4.019631	-1.330518	0.879172
17	1	0	3.030303	-2.218201	-0.277231
18	6	0	2.971831	1.122492	0.612735
19	1	0	3.609054	0.958239	1.488221

20	1	0	3.603817	1.513536	-0.193883
21	1	0	2.242172	1.892964	0.878546
22	15	0	-1.683878	-0.197333	-1.203831
23	8	0	-0.526212	-1.371557	-1.025157
24	7	0	-0.678455	1.030806	-1.809003
25	6	0	-1.356600	2.263566	-2.196793
26	1	0	-2.296507	2.023644	-2.701616
27	1	0	-1.579721	2.905848	-1.332647
28	1	0	-0.725289	2.822777	-2.894036
29	8	0	-1.798805	0.306842	0.384420
30	6	0	-2.445944	-0.574878	1.293809
31	1	0	-1.795930	-1.423877	1.531511
32	1	0	-2.653728	-0.011391	2.204480
33	1	0	-3.391139	-0.949248	0.880664

----

16 isomer A (trans, chair), M06-2X/6-31+G(d,p), benzene IEFPCM:
Sum of electronic and thermal Free Energies= -901.064889

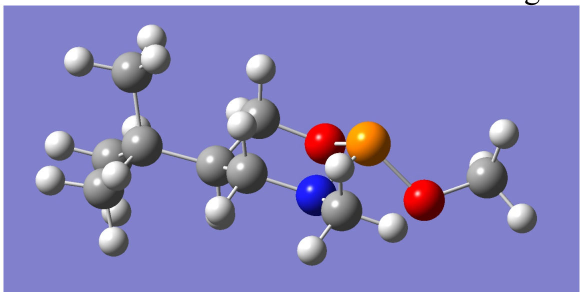

Center Number	Atomic Number	Atomic Type	Coordinates (Angstroms) X	Y	Z
1	6	0	0.464473	-1.299702	0.399705
2	6	0	0.454453	1.168186	0.027214
3	6	0	1.156021	-0.147993	-0.333826
4	1	0	0.528052	-1.152832	1.489358
5	1	0	0.910032	-2.267678	0.162678
6	1	0	0.870722	1.987496	-0.567077
7	1	0	0.640600	1.419000	1.090331
8	1	0	0.989082	-0.309597	-1.408063
9	6	0	2.693849	-0.124681	-0.097791
10	6	0	3.055877	-0.220868	1.392929
11	1	0	4.137148	-0.098714	1.518427
12	1	0	2.563755	0.558093	1.984844
13	1	0	2.785874	-1.193760	1.816201
14	6	0	3.333509	-1.309172	-0.839613
15	1	0	3.136387	-1.247554	-1.915381
16	1	0	4.419034	-1.301700	-0.693777
17	1	0	2.962068	-2.273568	-0.480611

18	6	0	3.298212	1.166377	-0.669689
19	1	0	4.391180	1.099978	-0.660279
20	1	0	2.978563	1.327976	-1.705792
21	1	0	3.017978	2.046075	-0.081879
22	15	0	-1.846075	-0.125870	0.534239
23	8	0	-0.906956	-1.386933	0.006585
24	7	0	-0.984193	1.136501	-0.270726
25	6	0	-1.599337	2.449652	-0.091810
26	1	0	-1.128098	3.158571	-0.779533
27	1	0	-2.661777	2.395309	-0.339979
28	1	0	-1.490699	2.839409	0.933819
29	6	0	-4.253866	-0.931573	-0.136512
30	1	0	-4.515902	-0.705830	0.902760
31	1	0	-5.061551	-0.606011	-0.792295
32	1	0	-4.107927	-2.009573	-0.249024
33	8	0	-3.080019	-0.226519	-0.541025

----

16 isomer B (trans, twist 1), M06-2X/6-31+G(d,p), benzene IEFPCM:
Sum of electronic and thermal Free Energies= -901.072457

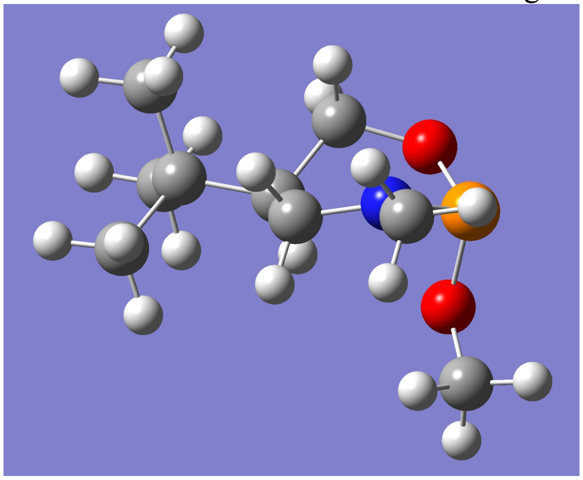

Center Number	Atomic Number	Atomic Type	Coordinates (Angstroms) X	Y	Z
1	6	0	0.705037	-1.648654	1.128603
2	6	0	0.378956	0.814953	0.993637
3	6	0	0.907065	-0.419632	0.242012
4	1	0	1.124484	-1.470984	2.127368
5	1	0	1.177943	-2.537351	0.705710
6	1	0	0.130330	1.618389	0.282807
7	1	0	1.159925	1.207064	1.663066
8	1	0	0.286352	-0.566694	-0.650594
9	6	0	2.374709	-0.253008	-0.240841
10	6	0	3.378123	-0.386859	0.914394

11	1	0	4.390940	-0.182005	0.551501
12	1	0	3.168713	0.318438	1.725720
13	1	0	3.377683	-1.397960	1.334982
14	6	0	2.686977	-1.324259	-1.296673
15	1	0	2.014959	-1.230796	-2.156473
16	1	0	3.715122	-1.209210	-1.656375
17	1	0	2.589827	-2.339003	-0.899401
18	6	0	2.548817	1.122579	-0.901555
19	1	0	3.543906	1.196536	-1.352777
20	1	0	1.806414	1.274505	-1.693623
21	1	0	2.452748	1.940197	-0.180015
22	15	0	-1.803608	-0.805717	1.474626
23	8	0	-0.680330	-1.988002	1.275072
24	7	0	-0.783421	0.499853	1.822855
25	6	0	-3.005818	0.470293	-0.456338
26	1	0	-2.505835	1.435137	-0.299012
27	1	0	-3.293648	0.385958	-1.504991
28	1	0	-3.911436	0.436768	0.164058
29	8	0	-2.130949	-0.608778	-0.164185
30	6	0	-1.268195	1.610804	2.623313
31	1	0	-0.494832	1.931257	3.331609
32	1	0	-1.541524	2.474891	2.000084
33	1	0	-2.148755	1.302821	3.192766

----

16 isomer C (trans, twist 2), M06-2X/6-31+G(d,p), benzene IEFPCM:
Sum of electronic and thermal Free Energies= -901.071523

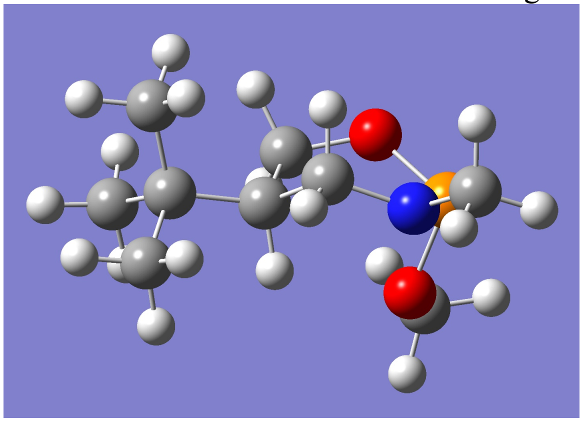

Center Number	Atomic Number	Atomic Type	Coordinates (Angstroms) X	Y	Z
1	6	0	0.050046	-1.249991	0.401707
2	6	0	0.335941	1.145716	-0.250939
3	6	0	0.841616	-0.279693	-0.490434
4	1	0	0.497655	-1.313794	1.399090

5	1	0	0.040402	-2.253716	-0.036870
6	1	0	0.967731	1.872923	-0.774814
7	1	0	0.384679	1.385492	0.825822
8	1	0	0.614738	-0.524548	-1.535255
9	6	0	2.378030	-0.430068	-0.310109
10	6	0	2.869681	0.188433	1.006442
11	1	0	3.939496	-0.009868	1.132102
12	1	0	2.732098	1.274581	1.016685
13	1	0	2.351935	-0.227284	1.877348
14	6	0	2.754761	-1.919792	-0.339457
15	1	0	2.365361	-2.405319	-1.241876
16	1	0	3.844318	-2.029822	-0.340989
17	1	0	2.371789	-2.459415	0.532661
18	6	0	3.097645	0.257189	-1.480090
19	1	0	4.181532	0.137950	-1.376275
20	1	0	2.798189	-0.185693	-2.436065
21	1	0	2.888158	1.330139	-1.522072
22	15	0	-2.134190	0.044021	-0.489606
23	8	0	-1.311537	-0.847673	0.633752
24	7	0	-1.033715	1.311500	-0.735916
25	6	0	-2.517140	-2.070317	-1.979539
26	1	0	-3.593171	-1.909033	-1.839464
27	1	0	-2.341644	-2.452898	-2.985567
28	1	0	-2.171013	-2.808123	-1.246368
29	8	0	-1.791874	-0.851116	-1.859196
30	6	0	-1.536858	2.671526	-0.598849
31	1	0	-2.584241	2.717022	-0.905720
32	1	0	-1.459534	3.039888	0.435178
33	1	0	-0.963772	3.342881	-1.247401

----

17 isomer A (cis, chair), M06-2X/6-31+G(d,p), benzene IEFPCM:
Sum of electronic and thermal Free Energies= -920.449712

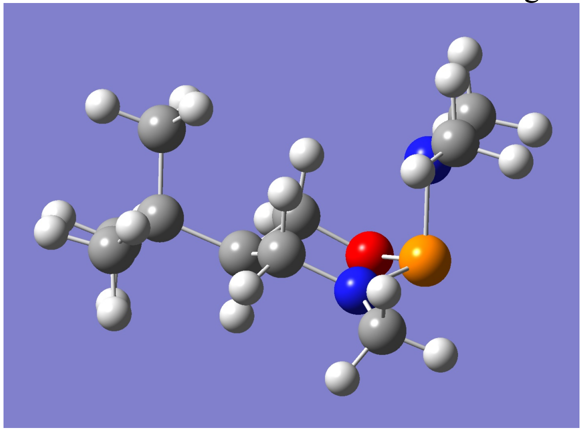

Center Number	Atomic Number	Atomic Type	Coordinates (Angstroms) X	Y	Z
1	6	0	0.462912	-1.171499	-0.747913
2	6	0	0.404422	1.130879	0.275652
3	6	0	1.267030	0.103759	-0.477560
4	1	0	0.163223	-1.642556	0.194123
5	1	0	1.041448	-1.886073	-1.337219
6	1	0	0.948237	2.078229	0.343109
7	1	0	0.201465	0.790697	1.305203
8	1	0	1.495110	0.553724	-1.455433
9	6	0	2.625742	-0.196705	0.220268
10	6	0	2.456087	-1.088660	1.460709
11	1	0	3.412855	-1.174822	1.987262
12	1	0	1.724521	-0.675974	2.163410
13	1	0	2.136705	-2.100751	1.193326
14	6	0	3.563939	-0.901801	-0.772295
15	1	0	3.739217	-0.276757	-1.654892
16	1	0	4.532346	-1.096876	-0.299012
17	1	0	3.165761	-1.863083	-1.109623
18	6	0	3.301318	1.115501	0.646850
19	1	0	4.325096	0.914604	0.979489
20	1	0	3.351078	1.824916	-0.187727
21	1	0	2.775358	1.597288	1.477060
22	15	0	-1.844347	0.132141	-0.916566
23	8	0	-0.713252	-0.906861	-1.526397
24	7	0	-0.838866	1.409027	-0.441280
25	6	0	-1.371695	2.752227	-0.299829
26	1	0	-0.660439	3.485465	-0.697375
27	1	0	-2.304518	2.838505	-0.864133
28	1	0	-1.581618	3.012867	0.748469
29	7	0	-2.088821	-0.688580	0.620610
30	6	0	-2.742136	-1.984902	0.467861
31	1	0	-2.733477	-2.510360	1.428799
32	1	0	-3.790783	-1.899441	0.134305
33	1	0	-2.197055	-2.592150	-0.259433
34	6	0	-2.772046	0.142294	1.602946
35	1	0	-2.832518	-0.400456	2.552038
36	1	0	-2.206866	1.061916	1.778110
37	1	0	-3.800028	0.414700	1.303962

17 isomer B (cis, twist 1), M06-2X/6-31+G(d,p), benzene IEFPCM:
Sum of electronic and thermal Free Energies= -920.449992

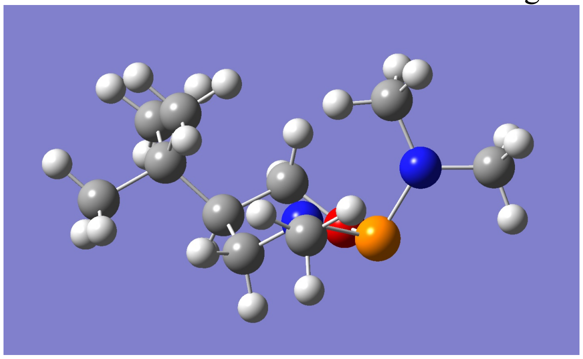

Center Number	Atomic Number	Atomic Type	Coordinates (Angstroms) X	Y	Z
1	6	0	0.045326	-1.380328	-0.191247
2	6	0	0.109606	1.077666	0.275606
3	6	0	0.876012	-0.099652	-0.362934
4	1	0	0.234016	-1.839735	0.786202
5	1	0	0.287922	-2.113844	-0.963273
6	1	0	-0.490971	1.598683	-0.489366
7	1	0	0.810436	1.821312	0.668254
8	1	0	0.949767	0.100290	-1.441096
9	6	0	2.329573	-0.263073	0.159930
10	6	0	2.386476	-0.313056	1.693625
11	1	0	3.426157	-0.422631	2.021728
12	1	0	1.985282	0.600474	2.144755
13	1	0	1.819664	-1.158831	2.095372
14	6	0	2.937973	-1.555870	-0.406024
15	1	0	2.859805	-1.581073	-1.499360
16	1	0	3.999256	-1.619805	-0.142837
17	1	0	2.446246	-2.448976	-0.007141
18	6	0	3.182820	0.913807	-0.334893
19	1	0	4.216481	0.803743	0.010588
20	1	0	3.194752	0.952596	-1.429881
21	1	0	2.814669	1.876460	0.033852
22	15	0	-2.140125	-0.233028	0.855793
23	8	0	-1.347548	-1.097701	-0.332192
24	7	0	-0.724503	0.626378	1.395195
25	6	0	-1.008796	1.675791	2.365863
26	1	0	-0.067737	2.027484	2.803136
27	1	0	-1.530342	2.541176	1.925662
28	1	0	-1.628188	1.271750	3.171349
29	7	0	-2.350516	-1.209109	2.225183
30	6	0	-1.289708	-1.802436	3.025321
31	1	0	-1.594615	-1.819606	4.078996

32	1	0	-1.078319	-2.837140	2.716793
33	1	0	-0.383580	-1.201186	2.932538
34	6	0	-3.638556	-1.865628	2.392086
35	1	0	-3.945832	-1.825101	3.443892
36	1	0	-4.396447	-1.358188	1.789892
37	1	0	-3.600098	-2.920383	2.085704

----

17 isomer C (cis, twist 2), M06-2X/6-31+G(d,p), benzene IEFPCM:
Sum of electronic and thermal Free Energies= -920.446891

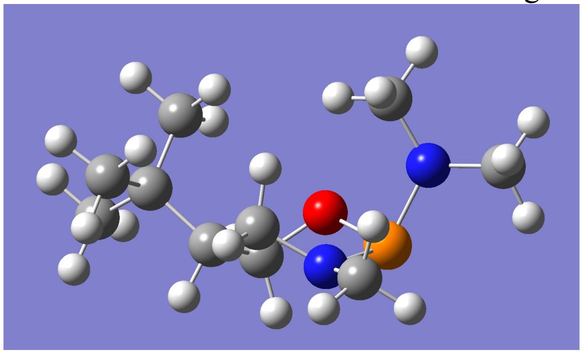

Center Number	Atomic Number	Atomic Type	Coordinates (Angstroms) X	Y	Z
1	6	0	0.733615	-1.382655	-1.075164
2	6	0	0.561251	1.087525	-0.663545
3	6	0	1.536446	-0.076997	-0.932504
4	1	0	1.305596	-2.252121	-0.743834
5	1	0	0.461048	-1.547507	-2.125435
6	1	0	1.000459	2.020421	-1.034082
7	1	0	0.408682	1.223734	0.418689
8	1	0	2.005382	0.115950	-1.908216
9	6	0	2.687768	-0.155884	0.105534
10	6	0	2.188022	-0.630732	1.477351
11	1	0	3.033352	-0.729675	2.167292
12	1	0	1.484573	0.081387	1.920451
13	1	0	1.686895	-1.602165	1.414164
14	6	0	3.763295	-1.128985	-0.400423
15	1	0	4.150618	-0.809920	-1.374664
16	1	0	4.603060	-1.162331	0.302018
17	1	0	3.384626	-2.150538	-0.504307
18	6	0	3.339802	1.226673	0.256910
19	1	0	4.218872	1.160621	0.906988
20	1	0	3.668071	1.613806	-0.715132
21	1	0	2.654700	1.955268	0.701766
22	15	0	-1.704145	-0.463619	-0.911997
23	8	0	-0.448943	-1.360029	-0.272228

24	7	0	-0.702633	0.882569	-1.367830
25	6	0	-1.456885	2.098510	-1.624333
26	1	0	-2.369713	1.855349	-2.179625
27	1	0	-1.752339	2.618060	-0.697339
28	1	0	-0.864753	2.789203	-2.233522
29	7	0	-2.480172	-0.123711	0.558249
30	6	0	-1.798239	0.313366	1.765320
31	1	0	-0.794131	-0.112556	1.791721
32	1	0	-1.734630	1.410616	1.829467
33	1	0	-2.345232	-0.047894	2.644369
34	6	0	-3.902142	0.176286	0.534197
35	1	0	-4.406323	-0.319434	1.372549
36	1	0	-4.094031	1.256860	0.609791
37	1	0	-4.343691	-0.187656	-0.397263

----

18 isomer A (trans, chair), M06-2X/6-31+G(d,p), benzene IEFPCM:
Sum of electronic and thermal Free Energies= -920.450264

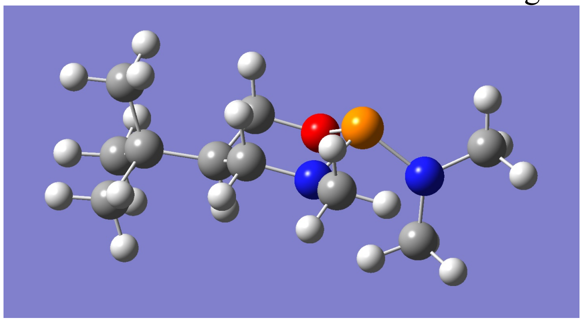

Center Number	Atomic Number	Atomic Type	Coordinates (Angstroms) X	Y	Z
1	6	0	-0.719527	-1.341483	-0.456006
2	6	0	-0.687608	1.150511	-0.205028
3	6	0	-1.376600	-0.148560	0.256378
4	1	0	-0.863989	-1.274110	-1.545154
5	1	0	-1.129587	-2.295774	-0.120702
6	1	0	-1.101300	2.005019	0.339124
7	1	0	-0.896997	1.329254	-1.277009
8	1	0	-1.148652	-0.262987	1.325229
9	6	0	-2.941667	-0.121091	0.136131
10	6	0	-3.429195	-0.009921	-1.325713
11	1	0	-4.524902	0.012971	-1.352931
12	1	0	-3.071126	0.904604	-1.809508
13	1	0	-3.106479	-0.862011	-1.933216
14	6	0	-3.525410	-1.412129	0.755545
15	1	0	-3.172250	-1.553839	1.783915
16	1	0	-4.619405	-1.354487	0.782876

17	1	0	-3.263593	-2.306681	0.182126
18	6	0	-3.504900	1.073099	0.939576
19	1	0	-4.598654	1.017481	0.978838
20	1	0	-3.133405	1.067482	1.971431
21	1	0	-3.246233	2.037546	0.491567
22	15	0	1.635901	-0.141967	-0.767198
23	8	0	0.684709	-1.400007	-0.156812
24	7	0	0.765958	1.152051	0.057710
25	6	0	1.345892	2.477525	-0.182788
26	1	0	0.874850	3.202762	0.490830
27	1	0	2.416900	2.459370	0.034139
28	1	0	1.207239	2.835390	-1.218451
29	7	0	3.015171	-0.240388	0.205180
30	6	0	4.255082	-0.730477	-0.391347
31	1	0	4.202963	-0.650985	-1.480514
32	1	0	5.105324	-0.130513	-0.041600
33	1	0	4.448018	-1.781770	-0.129401
34	6	0	2.973503	-0.318177	1.664468
35	1	0	2.028619	0.090666	2.024589
36	1	0	3.071819	-1.356132	2.013876
37	1	0	3.797472	0.268098	2.091815

----

18 isomer B (trans, twist 1), M06-2X/6-31+G(d,p), benzene IEFPCM:
Sum of electronic and thermal Free Energies= -920.446674

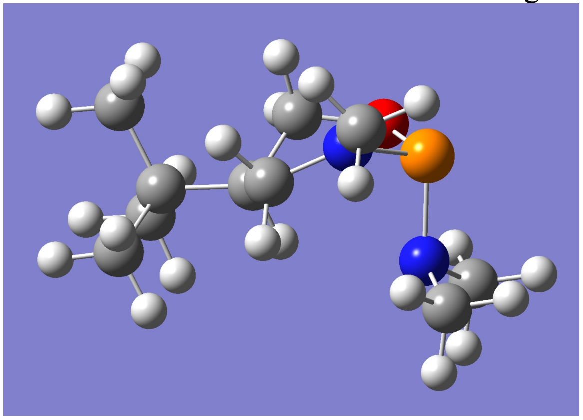

Center Number	Atomic Number	Atomic Type	Coordinates (Angstroms) X	Y	Z
1	6	0	0.764863	-1.618589	1.022633
2	6	0	0.513116	0.861174	0.878634
3	6	0	1.076800	-0.386956	0.170917
4	1	0	1.106948	-1.464138	2.054359
5	1	0	1.249863	-2.514775	0.628936
6	1	0	0.304862	1.654622	0.143301

7	1	0	1.260569	1.259778	1.581188
8	1	0	0.533918	-0.508589	-0.773485
9	6	0	2.585313	-0.254294	-0.177492
10	6	0	3.479435	-0.403844	1.062274
11	1	0	4.524811	-0.220459	0.791591
12	1	0	3.212116	0.308219	1.850350
13	1	0	3.420407	-1.413149	1.483158
14	6	0	2.969803	-1.335064	-1.199454
15	1	0	2.373667	-1.235961	-2.113095
16	1	0	4.026030	-1.236378	-1.472473
17	1	0	2.825793	-2.346482	-0.807882
18	6	0	2.844997	1.114476	-0.824586
19	1	0	3.878853	1.169321	-1.181883
20	1	0	2.181387	1.274505	-1.682274
21	1	0	2.696150	1.937868	-0.118853
22	15	0	-1.730469	-0.690842	1.227075
23	8	0	-0.635496	-1.924912	1.051606
24	7	0	-0.682470	0.561398	1.664367
25	6	0	-2.567958	-1.451925	-1.212381
26	1	0	-3.604195	-1.663021	-0.894090
27	1	0	-2.585705	-1.214484	-2.281373
28	1	0	-1.967717	-2.352902	-1.069524
29	6	0	-1.125724	1.635303	2.533307
30	1	0	-0.343071	1.878971	3.262066
31	1	0	-1.362128	2.549957	1.968622
32	1	0	-2.021457	1.326753	3.077989
33	7	0	-1.967775	-0.338303	-0.482783
34	6	0	-2.740210	0.886219	-0.663876
35	1	0	-3.776249	0.806143	-0.289046
36	1	0	-2.250449	1.720473	-0.152241
37	1	0	-2.787123	1.126868	-1.730915

----

18 isomer C (trans, twist 2), M06-2X/6-31+G(d,p), benzene IEFPCM:
Sum of electronic and thermal Free Energies= -920.449452

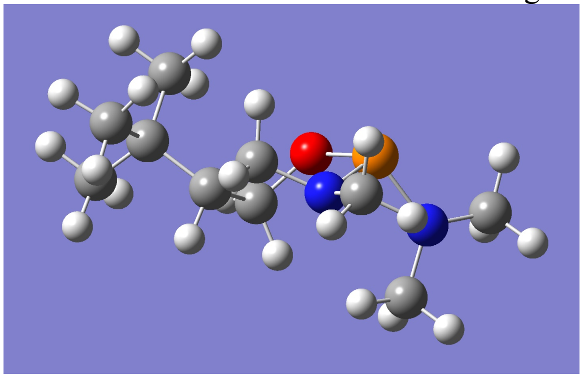

Center Number	Atomic Number	Atomic Type	Coordinates (Angstroms) X	Y	Z
1	6	0	0.785688	-1.412677	-0.805621
2	6	0	0.685825	1.086203	-0.836633
3	6	0	1.619996	-0.131208	-0.971595
4	1	0	1.386739	-2.245948	-0.433140
5	1	0	0.359907	-1.712339	-1.768387
6	1	0	1.081819	1.920907	-1.423503
7	1	0	0.646069	1.433754	0.211387
8	1	0	2.000758	-0.134519	-2.003920
9	6	0	2.857109	-0.054830	-0.037488
10	6	0	2.478463	-0.307740	1.428744
11	1	0	3.370657	-0.229314	2.059930
12	1	0	1.744625	0.417587	1.793130
13	1	0	2.051601	-1.305790	1.567958
14	6	0	3.889834	-1.103937	-0.474997
15	1	0	4.210446	-0.929072	-1.508361
16	1	0	4.776204	-1.052876	0.166526
17	1	0	3.497389	-2.123645	-0.409461
18	6	0	3.512433	1.329037	-0.162336
19	1	0	4.449422	1.354609	0.404297
20	1	0	3.745718	1.560062	-1.208764
21	1	0	2.869526	2.123877	0.228689
22	15	0	-1.548671	-0.227240	-0.233951
23	8	0	-0.264335	-1.222367	0.145819
24	7	0	-0.655319	0.777398	-1.345333
25	6	0	-3.735790	-1.671045	-0.863502
26	1	0	-3.992033	-1.288409	0.127661
27	1	0	-4.582558	-1.489403	-1.536054
28	1	0	-3.583539	-2.756898	-0.788392
29	6	0	-1.397033	1.961775	-1.765985
30	1	0	-2.380115	1.663145	-2.140217
31	1	0	-1.541442	2.690751	-0.951902
32	1	0	-0.857222	2.455993	-2.579709
33	7	0	-2.549045	-0.994440	-1.365879
34	6	0	-2.162647	-1.423097	-2.701997
35	1	0	-1.875287	-2.484782	-2.719364
36	1	0	-3.010568	-1.291488	-3.385503
37	1	0	-1.329718	-0.813549	-3.055457

19, M06-2X/6-31+G(d,p), chloroform IEFPCM:
Sum of electronic and thermal Free Energies= -2126.042561

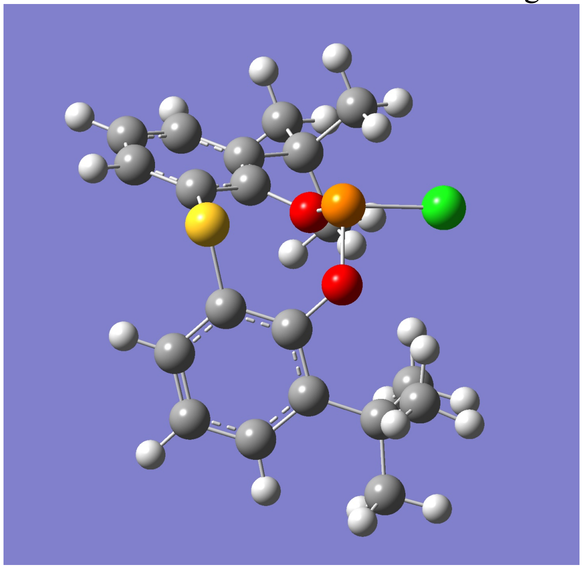

Center Number	Atomic Number	Atomic Type	Coordinates (Angstroms) X	Y	Z
1	15	0	0.020777	-0.727048	-1.792567
2	8	0	-0.841088	-0.470687	-0.391832
3	8	0	1.529944	-0.347767	-1.221237
4	16	0	-0.102237	2.110460	-1.694148
5	6	0	-1.809128	0.498925	-0.239721
6	6	0	-1.581167	1.784858	-0.748075
7	6	0	-2.520002	2.795404	-0.555105
8	1	0	-2.331716	3.785968	-0.955752
9	6	0	-3.678589	2.511841	0.156582
10	6	0	-3.884080	1.233253	0.671585
11	1	0	-4.793621	1.049766	1.229896
12	6	0	-2.967480	0.188274	0.495043
13	6	0	1.836553	0.678903	-0.354043
14	6	0	1.154671	1.899957	-0.445850
15	6	0	1.483424	2.960858	0.398160
16	1	0	0.943488	3.898336	0.311878
17	6	0	2.502191	2.797718	1.323430
18	6	0	3.181062	1.581262	1.397983
19	1	0	3.975612	1.488257	2.127747
20	6	0	2.875102	0.487386	0.581264
21	6	0	-3.220795	-1.216663	1.060348
22	6	0	-4.532055	-1.270148	1.857154
23	1	0	-4.518711	-0.586545	2.712464
24	1	0	-4.665836	-2.284722	2.243654
25	1	0	-5.401216	-1.036388	1.233904

26	6	0	-3.339451	-2.225896	-0.096505
27	1	0	-4.156075	-1.944363	-0.769641
28	1	0	-3.557245	-3.220654	0.307334
29	1	0	-2.416256	-2.296441	-0.673001
30	6	0	-2.083593	-1.631699	2.014039
31	1	0	-1.124719	-1.708975	1.500031
32	1	0	-2.318262	-2.608696	2.449975
33	1	0	-1.986560	-0.909369	2.831628
34	6	0	3.603815	-0.855451	0.734540
35	6	0	4.750430	-0.760192	1.751693
36	1	0	5.504488	-0.025557	1.449617
37	1	0	5.241225	-1.735717	1.816531
38	1	0	4.392863	-0.505200	2.754169
39	6	0	4.218916	-1.314447	-0.601421
40	1	0	4.894570	-0.547960	-0.996270
41	1	0	3.459467	-1.531174	-1.352004
42	1	0	4.802661	-2.225519	-0.432751
43	6	0	2.604031	-1.903890	1.254913
44	1	0	2.225079	-1.611878	2.240197
45	1	0	3.103398	-2.874349	1.352647
46	1	0	1.756791	-2.026685	0.579314
47	17	0	0.245468	-2.838002	-1.474584
48	1	0	-4.421135	3.285746	0.318754
49	1	0	2.777348	3.612687	1.984225

20, M06-2X/6-31+G(d,p), chloroform IEFPCM:
Sum of electronic and thermal Free Energies= -3749.202917

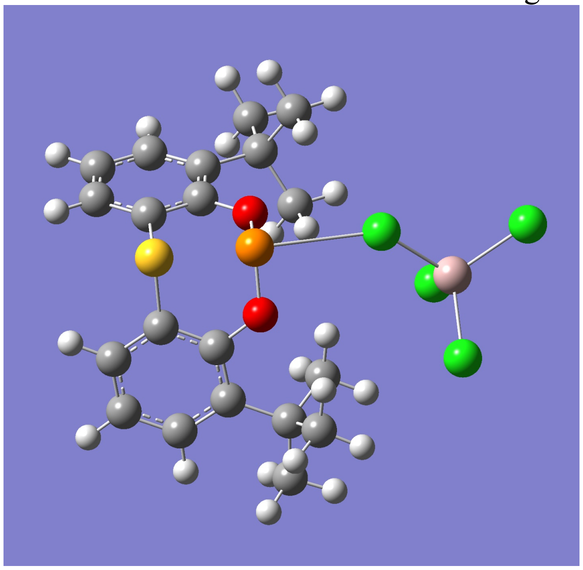

Center Number	Atomic Number	Atomic Type	Coordinates (Angstroms) X	Y	Z
1	15	0	-0.811062	0.059483	-1.991786
2	8	0	-1.056737	-1.122451	-0.891797
3	8	0	-0.492129	1.384530	-1.107494
4	16	0	-3.111144	0.576272	-1.858223
5	6	0	-2.269061	-1.505845	-0.363128
6	6	0	-3.404517	-0.816383	-0.785283
7	6	0	-4.677584	-1.170404	-0.352123
8	1	0	-5.553988	-0.632626	-0.696811
9	6	0	-4.776886	-2.232768	0.537460
10	6	0	-3.630987	-2.903928	0.969253
11	1	0	-3.755203	-3.721932	1.668376
12	6	0	-2.338930	-2.573869	0.538770
13	6	0	-1.427551	2.066267	-0.349858
14	6	0	-2.771267	1.850422	-0.653583
15	6	0	-3.785105	2.562264	-0.021243
16	1	0	-4.827234	2.393546	-0.270162
17	6	0	-3.410404	3.484930	0.946890
18	6	0	-2.063299	3.676379	1.259824
19	1	0	-1.817918	4.400228	2.027214
20	6	0	-1.022946	2.982392	0.628355
21	6	0	-1.080755	-3.309498	1.011577
22	6	0	-1.431832	-4.401473	2.029575
23	1	0	-1.915326	-3.988278	2.920946
24	1	0	-0.508029	-4.891188	2.349424
25	1	0	-2.083022	-5.169155	1.598649
26	6	0	-0.385933	-3.979648	-0.189052
27	1	0	-1.061733	-4.686706	-0.681612
28	1	0	0.489272	-4.532973	0.166053
29	1	0	-0.041167	-3.247456	-0.921386
30	6	0	-0.117927	-2.315042	1.688595
31	1	0	0.242921	-1.550051	0.997268
32	1	0	0.758787	-2.854295	2.060197
33	1	0	-0.602655	-1.823771	2.539290
34	6	0	0.454219	3.189828	0.975097
35	6	0	0.616242	4.202016	2.116030
36	1	0	0.236781	5.192044	1.842443
37	1	0	1.681129	4.305252	2.341234
38	1	0	0.112906	3.870952	3.030374
39	6	0	1.208836	3.731927	-0.253464
40	1	0	0.763513	4.672744	-0.594096
41	1	0	1.210429	3.019660	-1.080795
42	1	0	2.251115	3.920372	0.020399

43	6	0	1.071798	1.855024	1.432243
44	1	0	0.538268	1.454629	2.301258
45	1	0	2.116820	2.014153	1.714724
46	1	0	1.059665	1.101681	0.639981
47	17	0	2.058193	-0.909779	-1.428484
48	1	0	-5.750461	-2.542478	0.900097
49	1	0	-4.169947	4.057512	1.467131
50	13	0	3.804914	-0.475078	-0.197009
51	17	0	5.408991	-1.730185	-0.872157
52	17	0	4.313753	1.596000	-0.444853
53	17	0	3.334339	-0.906074	1.853023

21, M06-2X/6-31+G(d,p), dichloromethane IEFPCM:
Sum of electronic and thermal Free Energies= -3591.157898

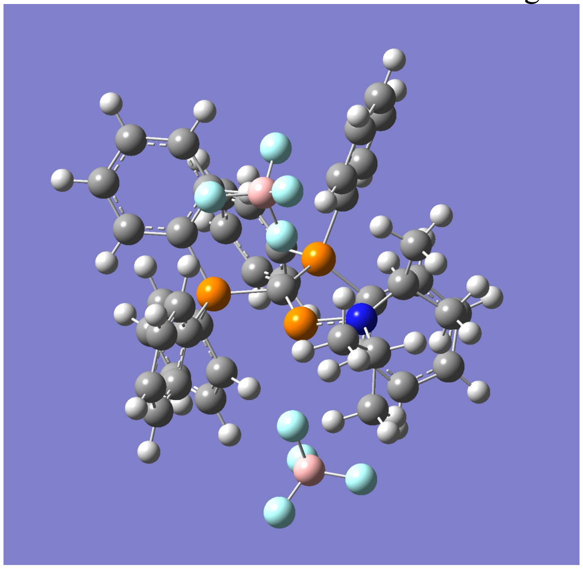

Center Number	Atomic Number	Atomic Type	Coordinates (Angstroms) X	Y	Z
1	6	0	-0.059638	-0.058062	-0.004905
2	6	0	0.584487	-4.208503	0.137632
3	1	0	0.521075	-4.854290	1.013952
4	6	0	2.006751	-4.282570	-0.406418
5	1	0	2.253246	-5.322609	-0.638587
6	1	0	2.726386	-3.899185	0.321971
7	1	0	2.111113	-3.688020	-1.319110
8	6	0	-0.464524	-4.683883	-0.866670
9	1	0	-0.399936	-4.126908	-1.806928
10	1	0	-1.474184	-4.564283	-0.465415
11	1	0	-0.291942	-5.740755	-1.088083
12	6	0	-0.045697	-2.783432	2.109924

13	1	0	-0.237615	-1.742378	2.354306
14	6	0	-1.327534	-3.570185	2.394595
15	1	0	-2.129031	-3.275361	1.711632
16	1	0	-1.647195	-3.367474	3.421140
17	1	0	-1.169057	-4.648517	2.300280
18	6	0	1.128957	-3.249683	2.968209
19	1	0	1.315937	-4.321235	2.855455
20	1	0	0.893446	-3.062544	4.019840
21	1	0	2.042263	-2.705714	2.714782
22	6	0	0.997209	2.252937	-1.551420
23	6	0	0.724320	3.614908	-1.710026
24	1	0	-0.294444	3.984849	-1.728488
25	6	0	1.779594	4.520216	-1.804320
26	1	0	1.564253	5.577832	-1.917683
27	6	0	3.098794	4.076420	-1.728387
28	1	0	3.914428	4.789150	-1.797316
29	6	0	3.369994	2.720479	-1.545890
30	1	0	4.388912	2.356730	-1.467959
31	6	0	2.322757	1.808239	-1.456037
32	1	0	2.550240	0.755553	-1.315406
33	6	0	-0.237119	-0.031341	-2.953825
34	6	0	-1.396191	-0.743851	-3.295749
35	1	0	-2.299294	-0.670799	-2.694735
36	6	0	-1.380361	-1.583733	-4.404121
37	1	0	-2.274450	-2.141668	-4.662130
38	6	0	-0.221835	-1.708202	-5.173115
39	1	0	-0.212944	-2.365354	-6.036791
40	6	0	0.923145	-0.988266	-4.836953
41	1	0	1.823250	-1.079760	-5.435319
42	6	0	0.922243	-0.147551	-3.725094
43	1	0	1.823065	0.398936	-3.471051
44	6	0	-1.950515	1.728556	-1.503709
45	6	0	-2.268353	2.632472	-2.529349
46	1	0	-1.521801	2.930207	-3.260061
47	6	0	-3.563069	3.128844	-2.631165
48	1	0	-3.806295	3.832719	-3.419971
49	6	0	-4.549710	2.706663	-1.735635
50	1	0	-5.560522	3.090921	-1.826137
51	6	0	-4.245974	1.769456	-0.752519
52	1	0	-5.013585	1.392922	-0.085116
53	6	0	-2.947084	1.276143	-0.638997
54	1	0	-2.732286	0.511960	0.097519
55	6	0	0.261288	2.524189	1.492118
56	6	0	-0.877218	3.328866	1.351850
57	1	0	-1.867103	2.885209	1.297054
58	6	0	-0.740921	4.713598	1.302221

59	1	0	-1.623674	5.334918	1.193797
60	6	0	0.522806	5.294568	1.404252
61	1	0	0.626062	6.374435	1.370184
62	6	0	1.654188	4.492829	1.553249
63	1	0	2.638030	4.943419	1.631574
64	6	0	1.529273	3.106823	1.597710
65	1	0	2.414848	2.489434	1.714070
66	6	0	1.553421	0.087744	2.433244
67	6	0	2.677472	-0.239735	1.667092
68	1	0	2.647189	-0.204897	0.583118
69	6	0	3.858075	-0.626868	2.300193
70	1	0	4.716706	-0.884727	1.689728
71	6	0	3.912600	-0.693645	3.691442
72	1	0	4.829844	-1.000214	4.183720
73	6	0	2.786297	-0.379827	4.453926
74	1	0	2.822708	-0.445957	5.536186
75	6	0	1.604694	0.012603	3.830525
76	1	0	0.732765	0.239789	4.434983
77	6	0	-1.392143	0.495725	2.649560
78	6	0	-2.379983	-0.436061	2.323773
79	1	0	-2.304684	-1.037305	1.421000
80	6	0	-3.495130	-0.581890	3.147285
81	1	0	-4.263946	-1.293885	2.867811
82	6	0	-3.619145	0.194025	4.297366
83	1	0	-4.487445	0.078858	4.938063
84	6	0	-2.637583	1.133918	4.619757
85	1	0	-2.739484	1.750311	5.506515
86	6	0	-1.529323	1.295476	3.795006
87	1	0	-0.786847	2.051397	4.036738
88	7	0	0.276158	-2.824564	0.665469
89	15	0	0.193379	-1.703751	-0.520652
90	15	0	-0.299805	0.988121	-1.450319
91	15	0	0.072212	0.719753	1.596317
92	5	0	4.384288	-1.305079	-1.248265
93	5	0	-3.971215	-2.052747	-0.698781
94	9	0	4.796112	-2.286960	-0.340213
95	9	0	4.732515	-0.031101	-0.754617
96	9	0	4.973803	-1.512141	-2.497311
97	9	0	2.971563	-1.350989	-1.380690
98	9	0	-2.560148	-2.025441	-0.520266
99	9	0	-4.558440	-1.302590	0.333823
100	9	0	-4.279079	-1.485021	-1.941892
101	9	0	-4.414010	-3.376528	-0.641291

22, M06-2X/6-31+G(d,p), acetonitrile IEFPCM:
Sum of electronic and thermal Free Energies= -4591.215889

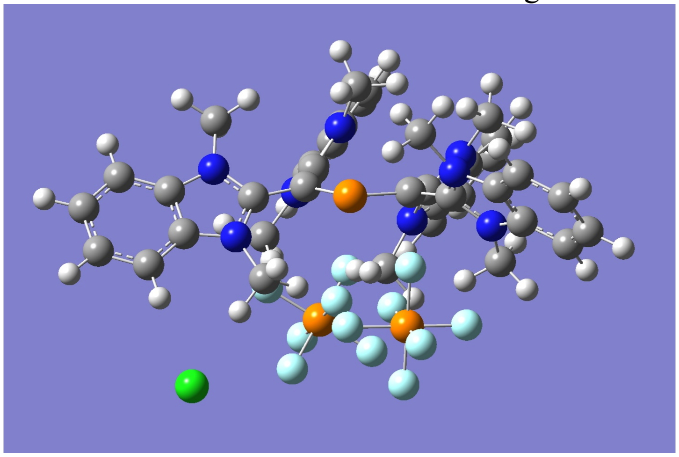

Center Number	Atomic Number	Atomic Type	Coordinates (Angstroms) X	Y	Z
1	6	0	1.643132	-1.005826	-0.885210
2	6	0	0.574129	-1.959968	-0.644916
3	6	0	-1.298531	-2.925144	0.085742
4	6	0	-2.522098	-3.220003	0.688007
5	1	0	-3.016503	-2.521225	1.355206
6	6	0	-3.084071	-4.444487	0.355869
7	1	0	-4.041533	-4.717477	0.786479
8	6	0	-2.450708	-5.339505	-0.530749
9	1	0	-2.931185	-6.285107	-0.756600
10	6	0	-1.234271	-5.041446	-1.128474
11	1	0	-0.751377	-5.730173	-1.812032
12	6	0	-0.675144	-3.805012	-0.802563
13	6	0	-0.624128	-0.809348	1.242271
14	1	0	0.111139	-0.019441	1.105247
15	1	0	-0.451122	-1.302019	2.202708
16	1	0	-1.636157	-0.407522	1.232341
17	6	0	1.442134	-3.766913	-2.164354
18	1	0	2.014988	-2.980860	-2.654972
19	1	0	0.893396	-4.331412	-2.918892
20	1	0	2.116333	-4.434287	-1.622192
21	6	0	2.986242	-1.528234	-0.685551
22	6	0	4.683664	-2.631615	0.246196
23	6	0	5.522549	-3.404664	1.049235
24	1	0	5.158298	-3.928148	1.925430
25	6	0	6.855922	-3.470338	0.669624
26	1	0	7.546105	-4.056650	1.266051
27	6	0	7.337082	-2.792957	-0.468292
28	1	0	8.387934	-2.870039	-0.724315

29	6	0	6.500640	-2.025433	-1.266724
30	1	0	6.874617	-1.496715	-2.135778
31	6	0	5.160085	-1.963343	-0.884095
32	6	0	2.475337	-2.717646	1.459335
33	1	0	1.895353	-1.847720	1.769372
34	1	0	3.117136	-3.007089	2.288434
35	1	0	1.829459	-3.556870	1.188876
36	6	0	4.164820	-0.562211	-2.702256
37	1	0	3.198531	-0.570963	-3.205126
38	1	0	4.895993	-1.057969	-3.340520
39	1	0	4.474195	0.465448	-2.506104
40	6	0	-0.070996	1.172202	-1.572383
41	6	0	-1.228275	0.321876	-1.776645
42	6	0	-2.538244	-1.344054	-2.468836
43	6	0	-3.074229	-2.491384	-3.054025
44	1	0	-2.514966	-3.084365	-3.769712
45	6	0	-4.355733	-2.841546	-2.652068
46	1	0	-4.813928	-3.732454	-3.067569
47	6	0	-5.073797	-2.076926	-1.709983
48	1	0	-6.068046	-2.397878	-1.418948
49	6	0	-4.540199	-0.935020	-1.130625
50	1	0	-5.085326	-0.361828	-0.389435
51	6	0	-3.249053	-0.590390	-1.530607
52	6	0	-0.371473	-1.047793	-3.701025
53	1	0	-0.937578	-1.092819	-4.633140
54	1	0	0.114574	-2.007126	-3.526932
55	1	0	0.377450	-0.260342	-3.768738
56	6	0	-2.779683	1.484188	-0.176269
57	1	0	-1.896007	1.817939	0.367923
58	1	0	-3.496970	1.065555	0.530078
59	1	0	-3.230754	2.324565	-0.710068
60	6	0	-0.321208	2.601339	-1.653024
61	6	0	-0.341120	4.794514	-1.257717
62	6	0	-0.131517	6.083207	-0.764132
63	1	0	0.533547	6.276268	0.074534
64	6	0	-0.834728	7.101095	-1.394303
65	1	0	-0.709921	8.120850	-1.046565
66	6	0	-1.707903	6.846826	-2.470709
67	1	0	-2.236447	7.674916	-2.930053
68	6	0	-1.913676	5.562912	-2.956841
69	1	0	-2.593699	5.371170	-3.778606
70	6	0	-1.205510	4.539840	-2.324829
71	6	0	1.067319	3.429156	0.299965
72	1	0	0.880365	2.480055	0.804717
73	1	0	0.863757	4.251725	0.988259
74	1	0	2.110004	3.471687	-0.014580

75	6	0	-1.819437	2.500799	-3.669317
76	1	0	-1.161903	1.728403	-4.068376
77	1	0	-1.993905	3.238846	-4.450553
78	1	0	-2.775727	2.070642	-3.361135
79	7	0	-0.483506	-1.802746	0.178737
80	7	0	0.482040	-3.170807	-1.238743
81	7	0	3.332062	-2.336071	0.340218
82	7	0	4.074254	-1.313488	-1.454715
83	7	0	-1.299140	-0.733581	-2.618504
84	7	0	-2.404766	0.437020	-1.126044
85	7	0	0.177018	3.569110	-0.854432
86	7	0	-1.158679	3.170967	-2.553240
87	15	0	1.559650	0.685120	-1.237749
88	9	0	2.262240	0.519202	1.846780
89	9	0	4.221584	0.621401	0.639355
90	9	0	3.488067	2.467712	1.803152
91	9	0	5.354401	1.320999	2.521962
92	9	0	4.125728	-0.628526	2.567232
93	9	0	3.394127	1.215468	3.731630
94	9	0	-5.632492	0.470521	1.617953
95	9	0	-4.841021	0.646435	3.770673
96	9	0	-6.693877	-0.651446	3.334568
97	9	0	-4.689287	-1.645775	3.907326
98	9	0	-3.640249	-0.521577	2.189096
99	9	0	-5.487117	-1.822674	1.757420
100	17	0	1.666046	6.444409	2.406667
101	15	0	3.815695	0.924780	2.191386
102	15	0	-5.173396	-0.590134	2.772786

23, M06-2X/6-31+G(d,p), benzene IEFPCM:
Sum of electronic and thermal Free Energies= -1266.592709

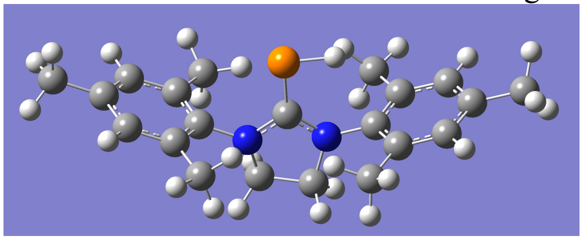

Center Number	Atomic Number	Atomic Type	Coordinates (Angstroms) X	Y	Z
1	15	0	-0.135308	-0.003938	-1.790255
2	7	0	-1.093359	0.093248	0.783705
3	6	0	-0.006774	0.002199	-0.042911
4	6	0	-0.734215	-0.160452	2.178237

5	1	0	-0.930939	-1.207273	2.448411
6	1	0	-1.299648	0.492295	2.847392
7	6	0	-2.442374	0.035432	0.323926
8	6	0	-3.112992	1.245012	0.087828
9	6	0	-4.435971	1.194453	-0.346056
10	1	0	-4.966805	2.125464	-0.534479
11	6	0	-5.092456	-0.023323	-0.551489
12	6	0	-4.394677	-1.206665	-0.311605
13	1	0	-4.889762	-2.161370	-0.476544
14	6	0	-3.066694	-1.201556	0.124759
15	6	0	-2.397933	2.555390	0.287044
16	1	0	-2.014815	2.646592	1.308800
17	1	0	-3.067952	3.395320	0.092133
18	1	0	-1.540597	2.626795	-0.390972
19	6	0	-6.514910	-0.047423	-1.051668
20	1	0	-6.965762	-1.033203	-0.916192
21	1	0	-6.552589	0.195398	-2.118642
22	1	0	-7.129925	0.687665	-0.525104
23	6	0	-2.333268	-2.495285	0.372048
24	1	0	-1.279358	-2.412561	0.090890
25	1	0	-2.779698	-3.303367	-0.211499
26	1	0	-2.384301	-2.782680	1.428577
27	1	0	1.272455	-0.029549	-1.969009
28	7	0	1.099452	-0.086631	0.756958
29	6	0	0.763954	0.144100	2.164380
30	1	0	0.966625	1.186492	2.445921
31	1	0	1.343946	-0.518473	2.811071
32	6	0	2.448371	-0.031570	0.300047
33	6	0	3.117591	-1.241146	0.063209
34	6	0	4.443245	-1.192051	-0.363601
35	1	0	4.971476	-2.123430	-0.557522
36	6	0	5.104529	0.025098	-0.557921
37	6	0	4.404568	1.209183	-0.326268
38	1	0	4.899540	2.163459	-0.493834
39	6	0	3.073991	1.204723	0.102267
40	6	0	2.392757	-2.549155	0.241674
41	1	0	2.001837	-2.650183	1.259550
42	1	0	3.057861	-3.391594	0.040980
43	1	0	1.537775	-2.607607	-0.441067
44	6	0	6.545565	0.047684	-1.001873
45	1	0	6.840409	1.044777	-1.336734
46	1	0	6.714792	-0.654224	-1.823119
47	1	0	7.209052	-0.241070	-0.180021
48	6	0	2.329807	2.496318	0.326838
49	1	0	1.291621	2.416736	-0.009994
50	1	0	2.806886	3.311916	-0.220793

51	1	0	2.322163	2.770646	1.387815

24, M06-2X/6-31+G(d,p), benzene IEFPCM:
Sum of electronic and thermal Free Energies= -2284.605178

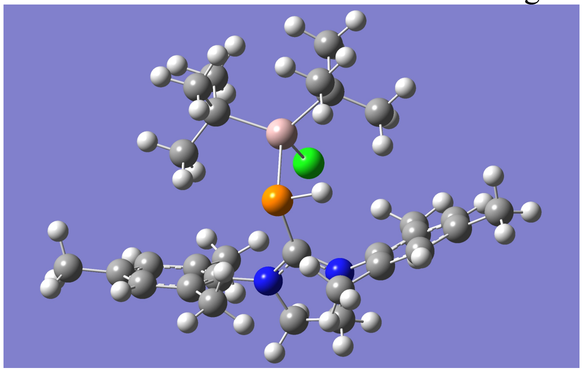

Center Number	Atomic Number	Atomic Type	Coordinates (Angstroms) X	Y	Z
1	15	0	-0.039958	0.019295	-1.115282
2	17	0	0.268674	1.840269	2.218358
3	13	0	0.281839	2.227329	0.037953
4	7	0	0.896638	-1.880293	0.703063
5	7	0	-1.297803	-1.793044	0.653033
6	6	0	2.258085	-1.785028	0.270573
7	6	0	2.615159	-2.318788	-0.980257
8	6	0	-0.165462	-1.262153	0.155022
9	6	0	3.218633	-1.249931	1.140917
10	6	0	4.542055	-1.191583	0.703669
11	1	0	5.290127	-0.755919	1.362695
12	6	0	4.922152	-1.642346	-0.560303
13	6	0	1.617689	-3.013319	-1.870573
14	1	0	0.883445	-3.576150	-1.285210
15	1	0	2.130499	-3.709064	-2.537993
16	1	0	1.065927	-2.292393	-2.482213
17	6	0	3.948128	-2.214923	-1.380067
18	1	0	4.233975	-2.609100	-2.352781
19	6	0	-2.643188	-1.512452	0.250332
20	6	0	2.093172	2.880095	-0.499165
21	6	0	0.488246	-2.828172	1.753357
22	1	0	0.713582	-2.411762	2.739712
23	1	0	1.024391	-3.771300	1.628768
24	6	0	-3.451976	-0.744806	1.097549
25	6	0	-1.367100	3.237060	-0.493357
26	6	0	2.852658	-0.742936	2.510644
27	1	0	1.871637	-0.261945	2.513095
28	1	0	3.586940	-0.009408	2.851851

29	1	0	2.842549	-1.561127	3.240466
30	6	0	3.140623	1.785146	-0.277684
31	1	0	3.181530	1.480964	0.775429
32	1	0	4.147537	2.144476	-0.553752
33	1	0	2.942600	0.884850	-0.875460
34	6	0	-4.773355	-0.515558	0.709301
35	1	0	-5.409508	0.090422	1.350529
36	6	0	6.345099	-1.496796	-1.034004
37	1	0	6.499481	-0.503854	-1.469749
38	1	0	6.586385	-2.237906	-1.799774
39	1	0	7.051225	-1.608758	-0.207476
40	6	0	-1.019664	-2.952070	1.515860
41	1	0	-1.293661	-3.874089	0.988870
42	1	0	-1.601264	-2.887516	2.436889
43	6	0	-3.131241	-2.065459	-0.943066
44	6	0	-5.289871	-1.028936	-0.481253
45	6	0	2.102807	3.283578	-1.979418
46	1	0	1.805238	2.454891	-2.635950
47	1	0	3.113955	3.596917	-2.289318
48	1	0	1.428911	4.126146	-2.178275
49	6	0	-2.907413	-0.172134	2.377975
50	1	0	-2.736170	-0.956830	3.123767
51	1	0	-3.611422	0.545183	2.804957
52	1	0	-1.955251	0.339776	2.211354
53	6	0	-2.604685	2.706266	0.238693
54	1	0	-2.815595	1.660071	-0.019006
55	1	0	-3.497319	3.293703	-0.036025
56	1	0	-2.491238	2.763882	1.327965
57	6	0	-4.456774	-1.805464	-1.290809
58	1	0	-4.848500	-2.222199	-2.215967
59	6	0	2.490397	4.097630	0.349473
60	1	0	1.800554	4.939659	0.218760
61	1	0	3.495284	4.452613	0.064668
62	1	0	2.517875	3.853872	1.418113
63	6	0	-1.631306	3.169417	-2.004176
64	1	0	-0.785762	3.542915	-2.593478
65	1	0	-2.508820	3.783224	-2.268206
66	1	0	-1.847015	2.147941	-2.341929
67	6	0	-1.163610	4.710357	-0.104086
68	1	0	-0.943622	4.828448	0.964807
69	1	0	-2.073403	5.295859	-0.318664
70	1	0	-0.342969	5.171479	-0.667466
71	6	0	-2.248950	-2.897169	-1.837408
72	1	0	-1.495025	-2.275436	-2.333491
73	1	0	-2.843160	-3.389938	-2.609268
74	1	0	-1.714487	-3.669294	-1.275154

75	6	0	-6.707324	-0.730647	-0.897973
76	1	0	-7.354085	-0.592706	-0.028283
77	1	0	-7.117611	-1.536927	-1.510818
78	1	0	-6.745621	0.189944	-1.489717
79	1	0	-1.443860	0.117191	-1.254789

25, M06-2X/6-31+G(d,p), chloroform IEFPCM:
Sum of electronic and thermal Free Energies= -1077.517235

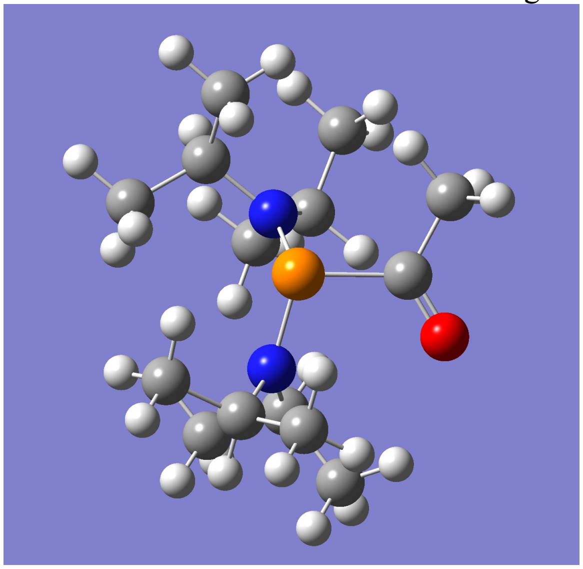

Center Number	Atomic Number	Atomic Type	Coordinates (Angstroms) X	Y	Z
1	15	0	0.039748	-0.121313	-0.872892
2	8	0	0.874594	-2.675197	-0.444810
3	7	0	-1.391628	0.241713	-0.021721
4	7	0	1.421240	0.221166	0.046875
5	6	0	-0.061712	-1.994762	-0.820986
6	6	0	-1.933904	-0.502416	1.130240
7	1	0	-1.298540	-1.388014	1.254557
8	6	0	2.595541	0.795083	-0.641596
9	1	0	3.339246	0.977424	0.140917
10	6	0	1.755754	1.085949	2.337655
11	1	0	2.701959	1.597549	2.129274
12	1	0	1.754132	0.812090	3.398022
13	1	0	0.942283	1.793547	2.153262
14	6	0	-1.878123	0.299485	2.435342
15	1	0	-0.860130	0.623595	2.659972
16	1	0	-2.241227	-0.310197	3.268845
17	1	0	-2.513476	1.189727	2.375319
18	6	0	2.760741	-1.135208	1.684010

19	1	0	2.631997	-2.025016	1.065987
20	1	0	2.788967	-1.431136	2.737905
21	1	0	3.725963	-0.673597	1.448841
22	6	0	-2.152458	1.441316	-0.433094
23	1	0	-2.958534	1.554458	0.298322
24	6	0	2.298106	2.146543	-1.291375
25	1	0	1.578613	2.038499	-2.109731
26	1	0	3.216570	2.575614	-1.704383
27	1	0	1.885061	2.842622	-0.555130
28	6	0	1.601870	-0.158639	1.455724
29	1	0	0.682771	-0.674979	1.752101
30	6	0	-3.364204	-0.997253	0.878888
31	1	0	-4.074823	-0.165144	0.839686
32	1	0	-3.676666	-1.655832	1.695055
33	1	0	-3.435478	-1.550443	-0.059994
34	6	0	3.208707	-0.185932	-1.644591
35	1	0	3.418129	-1.144841	-1.163065
36	1	0	4.138337	0.214394	-2.062819
37	1	0	2.513002	-0.365655	-2.472444
38	6	0	-1.312042	2.716686	-0.372836
39	1	0	-0.844282	2.823822	0.611044
40	1	0	-1.937702	3.594420	-0.563578
41	1	0	-0.520176	2.696271	-1.128375
42	6	0	-2.798228	1.260292	-1.807837
43	1	0	-2.028104	1.136128	-2.576689
44	1	0	-3.404270	2.133879	-2.068844
45	1	0	-3.440754	0.374659	-1.816664
46	6	0	-1.290268	-2.639327	-1.431657
47	1	0	-0.967063	-3.373104	-2.174827
48	1	0	-1.834291	-3.173990	-0.645999
49	1	0	-1.956254	-1.906171	-1.891408

26, M06-2X/6-31+G(d,p), benzene IEFPCM:
Sum of electronic and thermal Free Energies= -613.252746

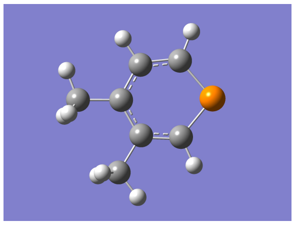

Center Number	Atomic Number	Atomic Type	Coordinates (Angstroms) X	Y	Z
1	6	0	-0.046989	-0.473492	0.033552

2	6	0	1.344248	-0.548648	0.088905
3	6	0	1.359657	2.100503	0.015012
4	6	0	-0.025082	1.977092	-0.034451
5	6	0	-0.722816	0.764634	-0.027272
6	1	0	1.781515	-1.545049	0.133557
7	1	0	1.779034	3.103823	0.003020
8	1	0	-0.617090	2.889712	-0.082632
9	15	0	2.469386	0.773248	0.094736
10	6	0	-0.855525	-1.749576	0.038586
11	1	0	-1.470939	-1.831032	-0.863662
12	1	0	-0.204367	-2.623899	0.087246
13	1	0	-1.536877	-1.782060	0.895413
14	6	0	-2.229151	0.787418	-0.086112
15	1	0	-2.600690	0.254069	-0.967761
16	1	0	-2.668896	0.299527	0.790456
17	1	0	-2.602791	1.812260	-0.127190

----

27 isomer A, M06-2X/6-31+G(d,p), benzene IEFPCM:
Sum of electronic and thermal Free Energies= -1241.770093

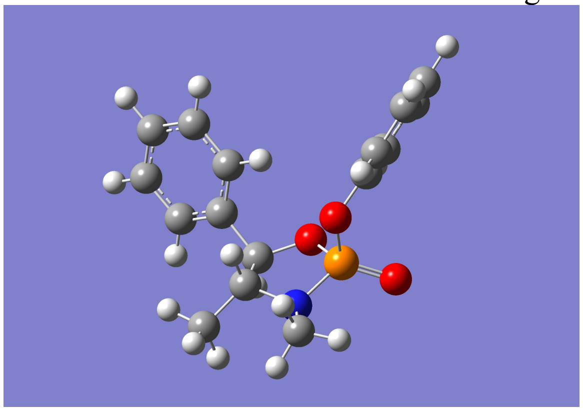

Center Number	Atomic Number	Atomic Type	Coordinates (Angstroms) X	Y	Z
1	6	0	-1.693652	0.720125	0.747421
2	6	0	-1.476551	1.737654	-0.398862
3	8	0	-0.394320	0.433898	1.314713
4	7	0	-0.294942	2.498249	0.030479
5	15	0	0.740164	1.443733	0.780030
6	8	0	1.723318	1.921431	1.765766
7	8	0	1.424779	0.655734	-0.472814
8	6	0	-2.696446	2.615976	-0.617248
9	1	0	-3.570546	1.988781	-0.817259
10	1	0	-2.559499	3.276651	-1.476572
11	1	0	-2.888790	3.224980	0.271166

12	6	0	-2.346864	-0.555150	0.288032
13	6	0	-3.704599	-0.772710	0.520379
14	6	0	-1.603625	-1.510111	-0.412649
15	6	0	-4.322712	-1.930421	0.046112
16	1	0	-4.279937	-0.039256	1.080113
17	6	0	-2.217946	-2.668702	-0.878288
18	1	0	-0.541585	-1.345902	-0.580486
19	6	0	-3.579884	-2.879325	-0.652531
20	1	0	-5.379476	-2.093399	0.232237
21	1	0	-1.634546	-3.409779	-1.415434
22	1	0	-4.057149	-3.784068	-1.015448
23	1	0	-1.247963	1.186342	-1.327207
24	1	0	-2.286972	1.193841	1.538104
25	6	0	0.235798	3.500201	-0.888029
26	1	0	0.419420	3.075589	-1.884139
27	1	0	-0.468389	4.330449	-0.973341
28	1	0	1.173541	3.891408	-0.488028
29	6	0	2.380264	-0.337148	-0.320132
30	6	0	3.329859	-0.451888	-1.330691
31	6	0	2.364696	-1.214561	0.761567
32	6	0	4.283091	-1.464151	-1.255363
33	1	0	3.308948	0.251080	-2.156586
34	6	0	3.329728	-2.218707	0.826332
35	1	0	1.613792	-1.118356	1.539838
36	6	0	4.288055	-2.349420	-0.177175
37	1	0	5.026401	-1.556620	-2.040779
38	1	0	3.326126	-2.902080	1.669333
39	1	0	5.034667	-3.134291	-0.119123

----

27 isomer B, M06-2X/6-31+G(d,p), benzene IEFPCM:
Sum of electronic and thermal Free Energies= -1241.765078

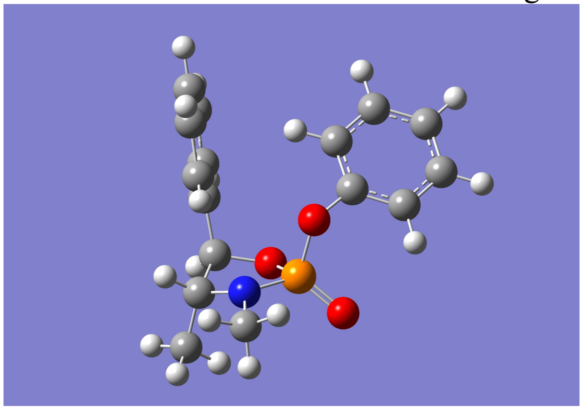

Center Number	Atomic Number	Atomic Type	Coordinates (Angstroms) X	Y	Z
1	6	0	-0.918369	0.413325	0.750520
2	6	0	-1.183519	1.396230	-0.411568
3	8	0	0.384336	0.783357	1.279371
4	7	0	0.143823	1.587354	-1.009460
5	15	0	1.345873	1.301355	0.093742
6	8	0	2.068582	-0.029102	-0.486888
7	6	0	-1.808928	2.699009	0.080336
8	1	0	-2.797420	2.504408	0.506072
9	1	0	-1.932326	3.409939	-0.740457
10	1	0	-1.178190	3.159726	0.847757
11	6	0	-0.927435	-1.059052	0.396866
12	6	0	-1.094192	-1.983865	1.431590
13	6	0	-0.717104	-1.523766	-0.904445
14	6	0	-1.052735	-3.352588	1.175070
15	1	0	-1.247930	-1.626494	2.446803
16	6	0	-0.681906	-2.892908	-1.164823
17	1	0	-0.551903	-0.818049	-1.713152
18	6	0	-0.847637	-3.809550	-0.126930
19	1	0	-1.182429	-4.059782	1.988124
20	1	0	-0.514294	-3.243103	-2.178366
21	1	0	-0.816929	-4.875080	-0.331860
22	1	0	-1.843663	0.923271	-1.149366
23	1	0	-1.618760	0.596717	1.569039
24	6	0	0.314742	2.530888	-2.106103
25	1	0	-0.424404	2.313405	-2.882615
26	1	0	0.205655	3.572800	-1.785251
27	1	0	1.308656	2.404313	-2.539445
28	6	0	2.977574	-0.732690	0.300806
29	6	0	4.200064	-0.170049	0.653482
30	6	0	2.627982	-2.024897	0.679932
31	6	0	5.092712	-0.931201	1.407246
32	1	0	4.427604	0.845954	0.350217
33	6	0	3.533226	-2.774122	1.428626
34	1	0	1.660470	-2.421707	0.386516
35	6	0	4.765097	-2.230611	1.793848
36	1	0	6.048737	-0.503388	1.691751
37	1	0	3.271132	-3.783718	1.729213
38	1	0	5.466480	-2.816799	2.378637
39	8	0	2.304565	2.365439	0.452688

28, M06-2X/6-31+G(d,p), benzene IEFPCM:
Sum of electronic and thermal Free Energies= -1241.769251

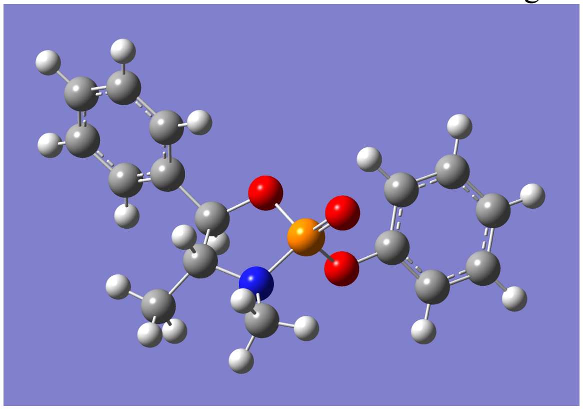

Center Number	Atomic Number	Atomic Type	Coordinates (Angstroms) X	Y	Z
1	6	0	1.379996	0.151458	-0.497364
2	6	0	1.514632	1.660892	-0.212713
3	8	0	0.350155	-0.320438	0.397698
4	7	0	0.132827	2.086294	0.041488
5	15	0	-0.759033	0.826242	0.645164
6	8	0	-1.881643	0.593810	-0.502108
7	8	0	-1.334023	0.876428	2.003052
8	6	0	-2.953151	-0.271679	-0.334043
9	6	0	-4.150226	0.095584	-0.939596
10	6	0	-2.830400	-1.473333	0.358138
11	6	0	-5.247203	-0.757482	-0.848241
12	1	0	-4.204641	1.039604	-1.471054
13	6	0	-3.938696	-2.313688	0.447435
14	1	0	-1.888156	-1.747442	0.822288
15	6	0	-5.146673	-1.962744	-0.153381
16	1	0	-6.183580	-0.475258	-1.318866
17	1	0	-3.851415	-3.249302	0.990320
18	1	0	-6.003904	-2.623569	-0.080109
19	6	0	2.158598	2.414432	-1.363836
20	1	0	3.158555	2.015138	-1.557823
21	1	0	2.265069	3.475703	-1.126215
22	1	0	1.550330	2.316943	-2.268256
23	6	0	2.650950	-0.619410	-0.271392
24	6	0	3.468191	-0.944442	-1.354753
25	6	0	3.046512	-0.969706	1.022245
26	6	0	4.680087	-1.603328	-1.148786
27	1	0	3.154146	-0.686615	-2.363439
28	6	0	4.252303	-1.635244	1.226680
29	1	0	2.398790	-0.732066	1.861196
30	6	0	5.073140	-1.949731	0.142379

31	1	0	5.309706	-1.853211	-1.996783
32	1	0	4.551322	-1.911470	2.232863
33	1	0	6.012405	-2.469067	0.303966
34	1	0	2.117492	1.793360	0.701884
35	1	0	1.026765	0.013014	-1.528122
36	6	0	-0.091087	3.447413	0.513533
37	1	0	0.547173	3.678351	1.376075
38	1	0	0.114078	4.160394	-0.288667
39	1	0	-1.133832	3.562562	0.814789

29, M06-2X/6-31+G(d,p), chloroform IEFPCM:
Sum of electronic and thermal Free Energies= -2861.501629

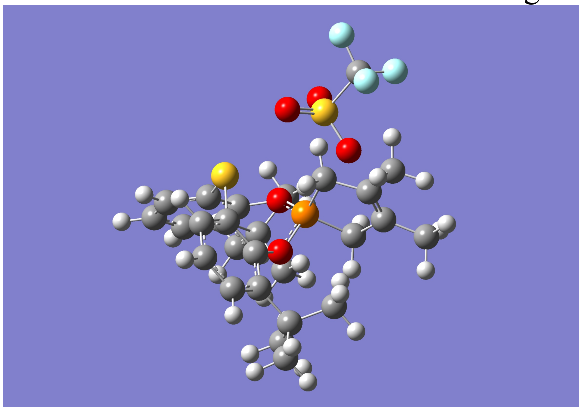

Center Number	Atomic Number	Atomic Type	Coordinates (Angstroms) X	Y	Z
1	8	0	-0.484730	1.008835	0.285756
2	8	0	1.771673	-0.175823	0.323241
3	16	0	0.680641	0.544939	-2.440899
4	6	0	0.056961	2.155581	-0.288073
5	6	0	0.644013	2.088480	-1.552351
6	6	0	1.156442	3.246605	-2.137231
7	1	0	1.605943	3.189503	-3.123129
8	6	0	1.066816	4.448741	-1.452133
9	6	0	0.469606	4.491207	-0.192624
10	1	0	0.410028	5.447681	0.311095
11	6	0	-0.048132	3.354202	0.438173
12	6	0	2.662515	-0.407678	-0.724677
13	6	0	2.313910	-0.052654	-2.034240
14	6	0	3.235437	-0.252026	-3.062243
15	1	0	2.963187	0.018286	-4.076974
16	6	0	4.484060	-0.782955	-2.768626
17	6	0	4.813644	-1.107479	-1.455055
18	1	0	5.800211	-1.507816	-1.259737
19	6	0	3.921043	-0.934024	-0.389120

20	6	0	-0.663483	3.415673	1.843993
21	6	0	-0.720404	4.859902	2.362778
22	1	0	0.274645	5.307487	2.450860
23	1	0	-1.170178	4.855593	3.359740
24	1	0	-1.338137	5.496380	1.721161
25	6	0	-2.104207	2.870124	1.841723
26	1	0	-2.718935	3.410236	1.114692
27	1	0	-2.544195	3.017564	2.833760
28	1	0	-2.157606	1.807424	1.602746
29	6	0	0.217754	2.613814	2.819997
30	1	0	0.297914	1.562862	2.535798
31	1	0	-0.215919	2.653613	3.824799
32	1	0	1.227911	3.034083	2.863338
33	6	0	4.302254	-1.310234	1.051697
34	6	0	5.751607	-1.812252	1.131312
35	1	0	5.907165	-2.715084	0.532603
36	1	0	5.978209	-2.062606	2.171616
37	1	0	6.466093	-1.047954	0.809413
38	6	0	3.397234	-2.452976	1.548935
39	1	0	3.508295	-3.336259	0.911226
40	1	0	2.341927	-2.176628	1.560565
41	1	0	3.683023	-2.730382	2.569235
42	6	0	4.194433	-0.086686	1.983008
43	1	0	4.838790	0.722863	1.625022
44	1	0	4.530123	-0.368093	2.986532
45	1	0	3.175363	0.294531	2.059397
46	6	0	-0.116335	-1.062086	2.066448
47	6	0	-0.296960	-1.865454	-0.551070
48	6	0	-0.357465	-2.538876	1.807247
49	1	0	-1.000493	-0.571439	2.487265
50	1	0	0.746455	-0.876402	2.717380
51	6	0	-0.462649	-2.926214	0.524085
52	1	0	-1.206140	-1.650073	-1.120085
53	1	0	0.503911	-2.118274	-1.256242
54	15	0	0.187985	-0.422899	0.414372
55	6	0	-0.722869	-4.311988	0.014598
56	1	0	0.093336	-4.635175	-0.641119
57	1	0	-0.826644	-5.040452	0.818253
58	1	0	-1.641415	-4.323890	-0.581590
59	6	0	-0.471028	-3.400374	3.028279
60	1	0	-0.661459	-4.446141	2.788787
61	1	0	0.452181	-3.344574	3.616775
62	1	0	-1.284451	-3.040365	3.667447
63	1	0	5.208692	-0.937077	-3.560866
64	1	0	1.452856	5.359603	-1.896822
65	16	0	-3.450532	-0.079852	-0.366033

66	8	0	-2.668911	-0.632885	0.760391
67	8	0	-4.418142	0.958484	-0.010433
68	8	0	-2.655177	0.157060	-1.579967
69	6	0	-4.507097	-1.519968	-0.840608
70	9	0	-5.289243	-1.900764	0.172799
71	9	0	-5.288841	-1.222539	-1.881486
72	9	0	-3.739969	-2.565001	-1.185183

----

anti-**30**, M06-2X/6-31+G(d,p), toluene IEFPCM:
Sum of electronic and thermal Free Energies= -695.324600

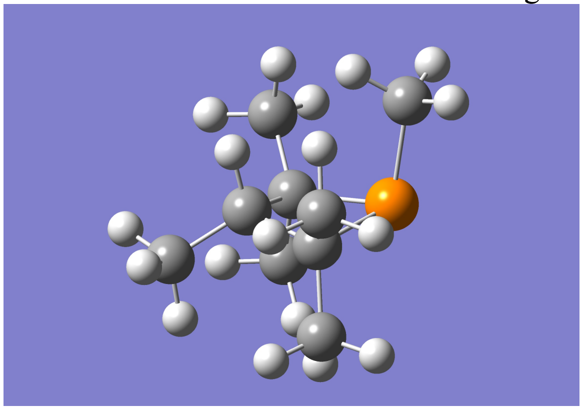

Center Number	Atomic Number	Atomic Type	Coordinates (Angstroms) X	Y	Z
1	6	0	-0.624615	1.061800	0.242476
2	6	0	0.793634	0.500909	-0.075197
3	6	0	-0.390675	2.553866	-0.139415
4	1	0	-0.742223	1.028355	1.335011
5	6	0	1.337213	-0.459565	0.976329
6	1	0	0.755702	-1.391016	0.964271
7	1	0	2.382130	-0.719943	0.772304
8	1	0	1.279584	-0.047007	1.987154
9	6	0	0.931487	-0.149893	-1.453903
10	1	0	1.982898	-0.385881	-1.649426
11	1	0	0.360914	-1.086251	-1.494697
12	1	0	0.583580	0.488142	-2.270016
13	6	0	-0.846995	2.933385	-1.550458
14	1	0	-1.941649	2.904162	-1.620372
15	1	0	-0.520243	3.952075	-1.784730
16	1	0	-0.445835	2.276225	-2.325958
17	6	0	-0.977334	3.553312	0.850836
18	1	0	-0.674048	4.577725	0.606135
19	1	0	-2.074103	3.514811	0.811761
20	1	0	-0.675700	3.345178	1.880909
21	6	0	-1.823426	0.350732	-0.375566

22	1	0	-1.873786	-0.689803	-0.036551
23	1	0	-2.758120	0.842437	-0.084385
24	1	0	-1.772912	0.345700	-1.468750
25	15	0	1.493151	2.272399	-0.108679
26	6	0	1.888658	2.556809	1.684853
27	1	0	2.860211	2.104911	1.904385
28	1	0	1.974922	3.633502	1.857060
29	1	0	1.147792	2.149090	2.378147

----

syn-**30** isomer A, M06-2X/6-31+G(d,p), toluene IEFPCM:
Sum of electronic and thermal Free Energies= -695.323112

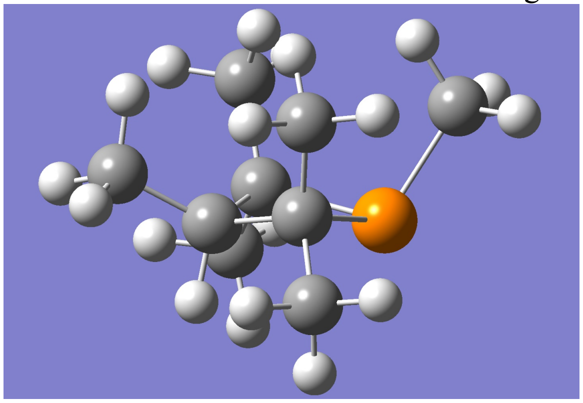

Center Number	Atomic Number	Atomic Type	Coordinates (Angstroms) X	Y	Z
1	6	0	-0.609581	1.069772	0.223745
2	6	0	0.839140	0.524555	0.021517
3	6	0	-0.349932	2.585835	-0.043039
4	1	0	-0.838981	0.971042	1.294527
5	6	0	1.257667	-0.495068	1.077694
6	1	0	0.650967	-1.406464	0.991140
7	1	0	2.308729	-0.780277	0.955894
8	1	0	1.131478	-0.096127	2.088904
9	6	0	1.112655	-0.048196	-1.367702
10	1	0	2.185008	-0.227142	-1.503754
11	1	0	0.598332	-1.010761	-1.482968
12	1	0	0.778291	0.599710	-2.180855
13	6	0	-0.671714	3.043080	-1.464455
14	1	0	-1.759108	3.075287	-1.609189
15	1	0	-0.284400	4.053057	-1.638999
16	1	0	-0.259341	2.390970	-2.237487
17	6	0	-1.050371	3.507143	0.952479
18	1	0	-0.767422	4.553000	0.788074
19	1	0	-2.140418	3.433380	0.840412
20	1	0	-0.795113	3.246968	1.984392

21	6	0	-1.733658	0.396586	-0.554203
22	1	0	-1.811469	-0.661470	-0.281466
23	1	0	-2.695553	0.870261	-0.330007
24	1	0	-1.574872	0.453882	-1.635355
25	15	0	1.485353	2.281859	0.341894
26	6	0	2.434258	2.782184	-1.171833
27	1	0	2.553862	3.869462	-1.160688
28	1	0	3.433862	2.341604	-1.114450
29	1	0	1.973062	2.487958	-2.117074

----

syn-**30** isomer B, M06-2X/6-31+G(d,p), toluene IEFPCM:
Sum of electronic and thermal Free Energies= -695.320381

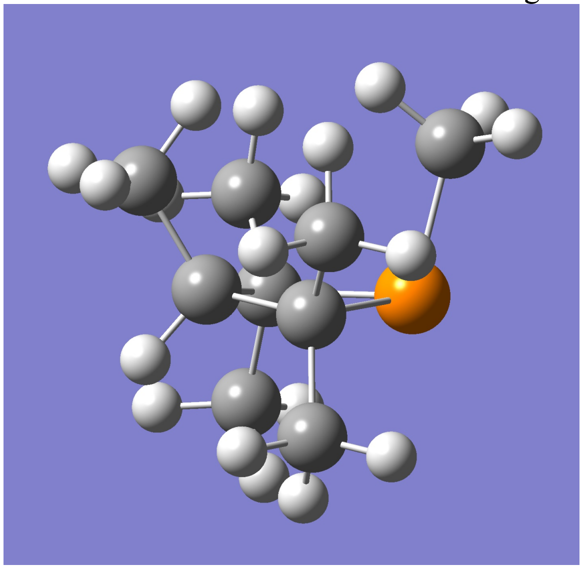

Center Number	Atomic Number	Atomic Type	Coordinates (Angstroms) X	Y	Z
1	6	0	-0.610994	1.069427	0.236729
2	6	0	0.834298	0.523826	-0.028587
3	6	0	-0.351677	2.579814	-0.093032
4	6	0	1.451501	-0.366644	1.044839
5	1	0	0.937907	-1.335943	1.078165
6	1	0	2.504524	-0.560529	0.810594
7	1	0	1.407384	0.067497	2.046050
8	6	0	0.930778	-0.197189	-1.375345
9	1	0	1.971123	-0.448092	-1.608791
10	1	0	0.351224	-1.128980	-1.341963
11	1	0	0.542599	0.406827	-2.200588
12	6	0	-0.890393	2.960335	-1.474292
13	1	0	-1.987655	2.925247	-1.469465
14	1	0	-0.580699	3.975048	-1.746703

15	1	0	-0.539255	2.284955	-2.259814
16	6	0	-0.841953	3.608945	0.920214
17	1	0	-0.474683	4.605489	0.649388
18	1	0	-1.938615	3.651243	0.920694
19	1	0	-0.517023	3.400304	1.941853
20	15	0	1.525858	2.289263	-0.174123
21	6	0	2.067677	2.656164	1.566070
22	1	0	3.054100	2.210204	1.722894
23	1	0	2.171212	3.739590	1.675626
24	1	0	1.391062	2.289547	2.341613
25	6	0	-1.178269	0.786208	1.625599
26	1	0	-2.168056	1.240338	1.734337
27	1	0	-1.285424	-0.291558	1.782731
28	1	0	-0.545704	1.176155	2.428123
29	1	0	-1.314954	0.640323	-0.493148

----

anti-**30[O]**, M06-2X/6-31+G(d,p), toluene IEFPCM:
Sum of electronic and thermal Free Energies= -770.557377

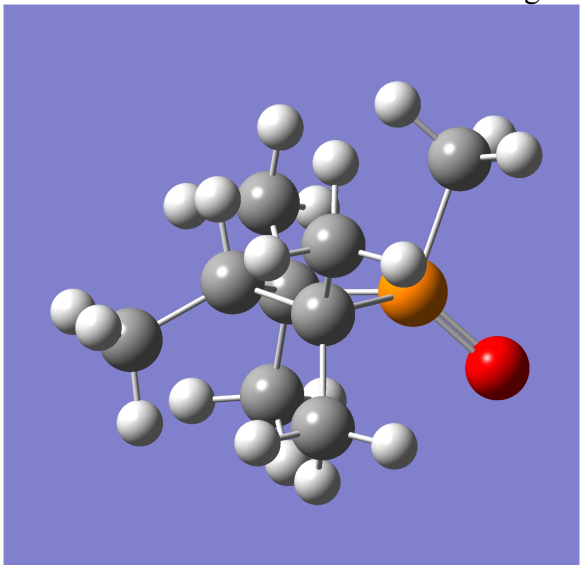

Center Number	Atomic Number	Atomic Type	Coordinates (Angstroms) X	Y	Z
1	6	0	-0.542203	1.099751	-0.091740
2	6	0	0.932526	0.559098	-0.111115
3	6	0	-0.269276	2.644649	-0.173298
4	15	0	1.481005	2.277545	0.311918
5	6	0	1.611371	2.406089	2.123954
6	1	0	2.539535	1.922875	2.439368
7	1	0	1.652823	3.464241	2.394248
8	1	0	0.766778	1.935059	2.631981

9	8	0	2.637361	2.922004	-0.402290
10	1	0	-0.947574	0.895976	0.909529
11	6	0	-1.503880	0.514893	-1.117439
12	1	0	-1.613060	-0.564686	-0.970708
13	1	0	-2.495360	0.968709	-1.017854
14	1	0	-1.157070	0.683045	-2.140881
15	6	0	1.204014	-0.554687	0.895870
16	1	0	0.666767	-1.462801	0.595649
17	1	0	2.270656	-0.798681	0.937041
18	1	0	0.873346	-0.292017	1.904943
19	6	0	1.445419	0.139772	-1.491499
20	1	0	2.517510	-0.070283	-1.438623
21	1	0	0.932712	-0.772178	-1.818224
22	1	0	1.304456	0.903520	-2.258687
23	6	0	-1.121656	3.481627	0.775998
24	1	0	-0.799057	4.527959	0.777867
25	1	0	-2.169038	3.457202	0.450689
26	1	0	-1.085140	3.110104	1.804206
27	6	0	-0.339718	3.235847	-1.583930
28	1	0	-1.377092	3.233328	-1.937655
29	1	0	0.014721	4.270472	-1.568629
30	1	0	0.269927	2.697228	-2.311910

----

syn-**30[O]** isomer A, M06-2X/6-31+G(d,p), toluene IEFPCM:
Sum of electronic and thermal Free Energies= -770.555380

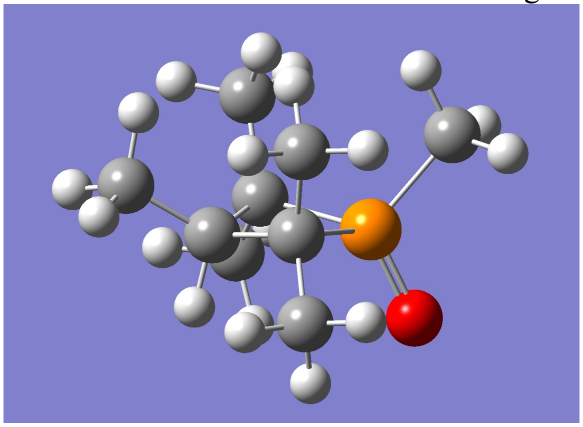

Center Number	Atomic Number	Atomic Type	Coordinates (Angstroms) X	Y	Z
1	6	0	-0.634340	1.055239	0.209260
2	6	0	0.823473	0.499648	0.016905
3	6	0	-0.380283	2.584778	-0.049963
4	1	0	-0.872839	0.951917	1.276185
5	6	0	1.259003	-0.466432	1.113122

6	1	0	0.615553	-1.355001	1.100631
7	1	0	2.292306	-0.795201	0.962530
8	1	0	1.193140	-0.001260	2.100189
9	6	0	1.097983	-0.097810	-1.364416
10	1	0	2.168614	-0.289944	-1.490210
11	1	0	0.572091	-1.054114	-1.467273
12	1	0	0.773865	0.543798	-2.188478
13	6	0	-0.723583	3.056098	-1.464187
14	1	0	-1.811542	3.071572	-1.597605
15	1	0	-0.352603	4.073485	-1.626503
16	1	0	-0.306873	2.418803	-2.249091
17	6	0	-1.025583	3.495211	0.988877
18	1	0	-0.789060	4.546280	0.794393
19	1	0	-2.116251	3.381269	0.955208
20	1	0	-0.679209	3.252140	1.996856
21	6	0	-1.744215	0.388764	-0.591977
22	1	0	-1.824360	-0.670966	-0.328327
23	1	0	-2.708987	0.859537	-0.376172
24	1	0	-1.571600	0.454770	-1.670170
25	15	0	1.419450	2.242041	0.237541
26	8	0	1.969326	2.605744	1.592024
27	6	0	2.487213	2.812648	-1.122809
28	1	0	2.592579	3.897539	-1.042371
29	1	0	3.472413	2.355815	-0.999087
30	1	0	2.086610	2.556462	-2.105144

----

syn-**30[O]** isomer B, M06-2X/6-31+G(d,p), toluene IEFPCM:
Sum of electronic and thermal Free Energies= -770.554717

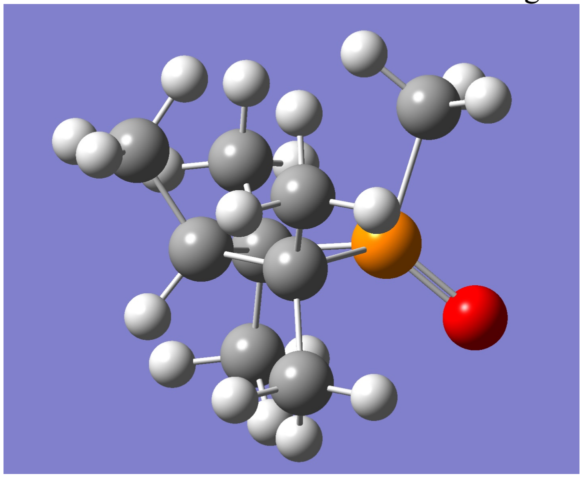

Center Number	Atomic Number	Atomic Type	Coordinates (Angstroms) X	Y	Z
1	6	0	0.000000	1.254270	-0.477563

2	6	0	1.205900	0.246135	-0.312665
3	6	0	-1.205900	0.246135	-0.312665
4	1	0	0.000000	1.654538	-1.500074
5	6	0	0.000000	-1.021091	2.103047
6	1	0	-0.889512	-1.552754	2.450453
7	1	0	0.889511	-1.552754	2.450453
8	1	0	0.000000	-0.008894	2.513622
9	15	0	0.000000	-1.024545	0.281463
10	6	0	2.325584	0.650785	0.643647
11	1	0	3.020890	-0.183753	0.781535
12	1	0	2.894636	1.489440	0.225549
13	1	0	1.963777	0.952613	1.629534
14	6	0	1.811894	-0.155765	-1.660248
15	1	0	2.337097	0.704409	-2.092174
16	1	0	2.528563	-0.972189	-1.531720
17	1	0	1.060285	-0.490626	-2.378266
18	6	0	0.000000	2.434513	0.488930
19	1	0	0.884355	3.058672	0.330985
20	1	0	-0.884354	3.058672	0.330985
21	1	0	0.000000	2.122777	1.538345
22	6	0	-1.811894	-0.155764	-1.660249
23	1	0	-2.528563	-0.972189	-1.531720
24	1	0	-2.337097	0.704410	-2.092174
25	1	0	-1.060285	-0.490626	-2.378266
26	6	0	-2.325584	0.650785	0.643646
27	1	0	-2.894636	1.489441	0.225549
28	1	0	-3.020890	-0.183752	0.781534
29	1	0	-1.963777	0.952614	1.629534
30	8	0	0.000000	-2.414614	-0.294159

----

anti-**31**, M06-2X/6-31+G(d,p), toluene IEFPCM:
Sum of electronic and thermal Free Energies= -1287.563870

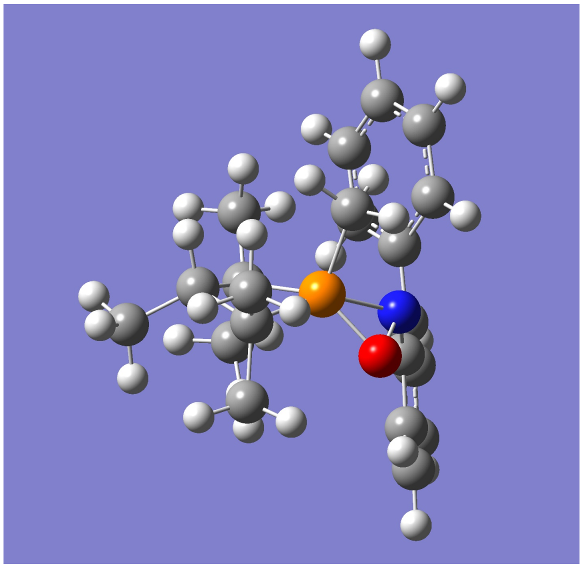

Center Number	Atomic Number	Atomic Type	Coordinates (Angstroms) X	Y	Z
1	6	0	-2.766294	-0.882920	1.059795
2	6	0	-3.192692	-0.115817	-0.232207
3	6	0	-1.217145	-0.634682	1.025351
4	15	0	-1.394940	-0.187034	-0.772330
5	6	0	-1.239115	-1.674325	-1.802370
6	1	0	-1.482190	-1.394116	-2.830750
7	1	0	-0.211750	-2.038977	-1.756423
8	1	0	-1.929157	-2.450929	-1.468011
9	8	0	-1.183981	1.207780	-1.578976
10	7	0	0.189627	0.603237	-1.346275
11	6	0	0.999710	1.519296	-0.672660
12	6	0	2.230754	1.072706	-0.123287
13	6	0	0.655655	2.877141	-0.566282
14	6	0	3.063808	2.010484	0.494272
15	6	0	1.508822	3.782517	0.056887
16	1	0	-0.290533	3.205997	-0.977671
17	6	0	2.723147	3.357926	0.591085
18	1	0	4.013629	1.668197	0.896539
19	1	0	1.218947	4.827368	0.120128
20	1	0	3.397560	4.060180	1.069282
21	6	0	2.626598	-0.359984	-0.123398
22	6	0	3.195636	-0.920412	1.030586
23	6	0	2.449049	-1.186965	-1.242773
24	6	0	3.565657	-2.263040	1.072139
25	1	0	3.319028	-0.302340	1.915820
26	6	0	2.817020	-2.530236	-1.200778
27	1	0	2.017408	-0.768715	-2.145948

28	6	0	3.371481	-3.076964	-0.042936
29	1	0	3.993224	-2.675463	1.981005
30	1	0	2.673758	-3.152128	-2.079799
31	1	0	3.651270	-4.125321	-0.011653
32	1	0	-2.924832	-1.954431	0.872905
33	6	0	-3.489543	-0.522852	2.351386
34	1	0	-4.561305	-0.726356	2.256959
35	1	0	-3.108746	-1.117482	3.188337
36	1	0	-3.368123	0.533958	2.605001
37	6	0	-4.260704	-0.816847	-1.067252
38	1	0	-5.225136	-0.786086	-0.544200
39	1	0	-4.394721	-0.318485	-2.033467
40	1	0	-4.023458	-1.867639	-1.255417
41	6	0	-3.630841	1.331381	0.021061
42	1	0	-3.764507	1.848909	-0.933598
43	1	0	-4.588179	1.348112	0.555358
44	1	0	-2.909444	1.909772	0.603579
45	6	0	-0.385440	-1.890013	1.286358
46	1	0	0.668064	-1.731540	1.037275
47	1	0	-0.444824	-2.145675	2.351806
48	1	0	-0.749074	-2.750095	0.716266
49	6	0	-0.719359	0.503306	1.922004
50	1	0	-0.924518	0.251287	2.968753
51	1	0	0.361598	0.624849	1.808408
52	1	0	-1.182652	1.469559	1.708302

----

syn-**31**, M06-2X/6-31+G(d,p), toluene IEFPCM:
Sum of electronic and thermal Free Energies= -1287.563088

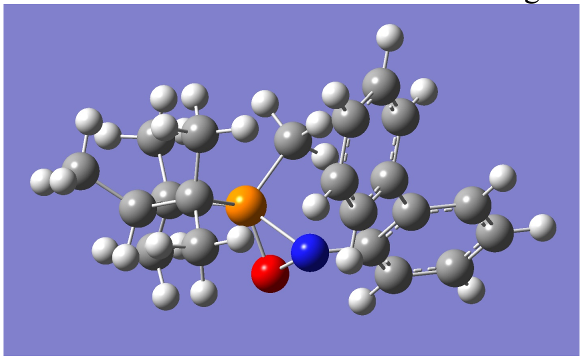

Center Number	Atomic Number	Atomic Type	Coordinates (Angstroms) X	Y	Z
1	6	0	3.049000	-0.841621	0.369172
2	6	0	0.346506	-0.708977	1.687765
3	15	0	1.195401	-0.588493	0.080059
4	6	0	3.499267	-0.761924	1.829492

5	1	0	3.163651	-1.644129	2.384990
6	1	0	4.594415	-0.734995	1.879665
7	1	0	3.130561	0.126713	2.351371
8	6	0	3.662668	-2.076876	-0.285908
9	1	0	4.757382	-1.993380	-0.291019
10	1	0	3.399203	-2.986737	0.264033
11	1	0	3.323479	-2.196004	-1.318318
12	8	0	0.837220	-1.708882	-1.032761
13	7	0	-0.342364	-0.745396	-0.991202
14	6	0	-1.486571	-1.422436	-0.565442
15	6	0	-2.573484	-0.645006	-0.089767
16	6	0	-1.602121	-2.820826	-0.586091
17	6	0	-3.727679	-1.297510	0.347306
18	6	0	-2.771941	-3.441651	-0.155204
19	1	0	-0.761842	-3.407081	-0.938383
20	6	0	-3.843239	-2.687221	0.317870
21	1	0	-4.555820	-0.692171	0.707745
22	1	0	-2.840350	-4.525198	-0.187649
23	1	0	-4.755311	-3.168046	0.654971
24	6	0	-2.490284	0.838589	-0.007581
25	6	0	-2.139527	1.613335	-1.121199
26	6	0	-2.791577	1.494447	1.194185
27	6	0	-2.096731	3.003109	-1.035394
28	1	0	-1.900670	1.113843	-2.055270
29	6	0	-2.740586	2.885233	1.283899
30	1	0	-3.057338	0.903610	2.067518
31	6	0	-2.392694	3.644921	0.167872
32	1	0	-1.829319	3.587416	-1.911205
33	1	0	-2.971209	3.373471	2.225902
34	1	0	-2.353355	4.727716	0.234054
35	6	0	1.837706	1.052251	-0.473745
36	6	0	3.293281	0.476742	-0.439602
37	1	0	3.534350	0.181372	-1.469684
38	6	0	1.413175	1.506772	-1.864508
39	1	0	0.387495	1.880012	-1.859826
40	1	0	2.081030	2.315295	-2.188894
41	1	0	1.476792	0.690570	-2.589666
42	6	0	1.520434	2.132974	0.564289
43	1	0	1.834301	1.862665	1.577847
44	1	0	2.026953	3.067231	0.296092
45	1	0	0.440511	2.326081	0.580840
46	6	0	4.388981	1.417169	0.045113
47	1	0	5.363258	0.918071	0.021810
48	1	0	4.451065	2.298109	-0.602587
49	1	0	4.213535	1.765003	1.067269
50	1	0	-0.462453	0.024355	1.728499

51	1	0	-0.087918	-1.710447	1.759219
52	1	0	1.037881	-0.556080	2.515163

32 isomer A, M06-2X/6-31+G(d,p), $CHCl_3$ IEFPCM:
Sum of electronic and thermal Free Energies= -882.156376

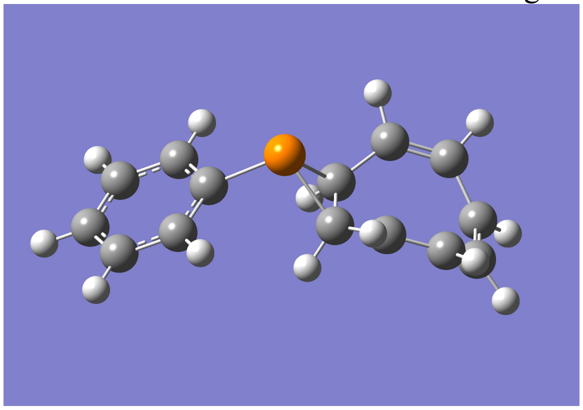

Center Number	Atomic Number	Atomic Type	Coordinates (Angstroms) X	Y	Z
1	6	0	-0.841457	-0.784479	0.013770
2	6	0	-1.967804	-1.592731	-0.531411
3	6	0	-3.184324	1.607404	-0.029525
4	6	0	-3.258987	-1.556240	-0.168139
5	6	0	-3.891112	0.673235	0.848517
6	6	0	-3.922985	-0.670180	0.789542
7	1	0	-1.673231	-2.380381	-1.222681
8	1	0	-0.314265	-1.283124	0.828425
9	1	0	-3.901638	-2.332276	-0.583549
10	1	0	-4.604598	-1.173789	1.474076
11	6	0	-0.806234	0.710234	0.078463
12	1	0	-0.256046	1.111471	0.930835
13	6	0	-1.892257	1.614814	-0.391478
14	1	0	-1.559731	2.445343	-1.011723
15	15	0	0.335015	-0.010328	-1.216415
16	1	0	-4.549473	1.146750	1.575945
17	1	0	-3.789176	2.445959	-0.374221
18	6	0	1.915154	-0.085720	-0.264663
19	6	0	2.592222	1.092005	0.076028
20	6	0	2.502842	-1.317226	0.049920
21	6	0	3.820696	1.040522	0.733908
22	1	0	2.153285	2.056864	-0.167676
23	6	0	3.731551	-1.370833	0.707511
24	1	0	1.994463	-2.241684	-0.214566
25	6	0	4.392323	-0.191581	1.051566
26	1	0	4.330475	1.961885	0.998544

27	1	0	4.171916	-2.332897	0.951051
28	1	0	5.348596	-0.232575	1.563513

---

32 isomer B, M06-2X/6-31+G(d,p), $CHCl_3$ IEFPCM:
Sum of electronic and thermal Free Energies= -882.155301

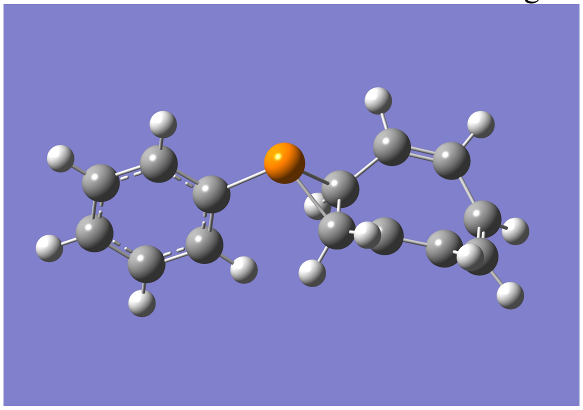

Center Number	Atomic Number	Atomic Type	Coordinates (Angstroms) X	Y	Z
1	6	0	-0.836209	-0.747394	0.082852
2	6	0	-1.934761	-1.610139	-0.434979
3	6	0	-3.233576	1.585756	-0.105228
4	6	0	-3.233540	-1.586200	-0.098945
5	6	0	-3.939845	0.674112	0.796083
6	6	0	-3.939846	-0.670997	0.798722
7	1	0	-1.607292	-2.426587	-1.076029
8	1	0	-0.300427	-1.206345	0.915379
9	1	0	-3.847827	-2.398981	-0.485983
10	1	0	-4.626905	-1.158147	1.489710
11	6	0	-0.836231	0.747730	0.079823
12	1	0	-0.300496	1.210080	0.910496
13	6	0	-1.934822	1.608349	-0.441453
14	1	0	-1.607387	2.422199	-1.085817
15	15	0	0.325354	-0.002342	-1.176946
16	1	0	-4.626895	1.163988	1.485152
17	1	0	-3.847908	2.396952	-0.495516
18	6	0	1.944763	-0.000575	-0.292424
19	6	0	2.125544	0.002509	1.097385
20	6	0	3.081835	-0.002531	-1.112128
21	6	0	3.405410	0.003645	1.648553
22	1	0	1.271508	0.004061	1.770020
23	6	0	4.363947	-0.001423	-0.562883
24	1	0	2.964077	-0.004953	-2.193427
25	6	0	4.528887	0.001691	0.820658
26	1	0	3.524721	0.006067	2.727706

27	1	0	5.230667	-0.002989	-1.216552
28	1	0	5.524528	0.002590	1.252830

33, M06-2X/6-31+G(d,p), $CHCl_3$ IEFPCM:
Sum of electronic and thermal Free Energies= -882.174021

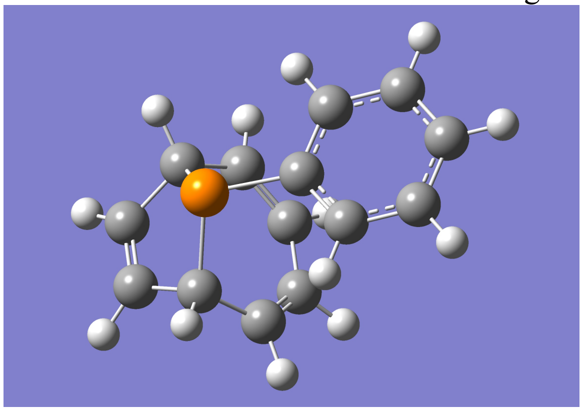

Center Number	Atomic Number	Atomic Type	Coordinates (Angstroms) X	Y	Z
1	6	0	0.141468	0.757768	-0.085593
2	6	0	1.418991	0.409563	-0.275134
3	6	0	0.784677	3.737209	-0.783769
4	6	0	2.445759	1.255996	0.454283
5	6	0	2.181324	3.357253	-0.989897
6	6	0	2.910645	2.345429	-0.474325
7	1	0	1.736601	-0.358917	-0.974490
8	1	0	-0.691716	0.302855	-0.614152
9	1	0	3.298018	0.655664	0.780503
10	1	0	3.956197	2.297511	-0.774888
11	6	0	-0.086649	1.946328	0.829853
12	1	0	-0.999303	1.827029	1.418009
13	6	0	-0.180046	3.187194	-0.016757
14	1	0	-1.147762	3.686560	-0.020176
15	15	0	1.421436	1.776646	1.950368
16	6	0	1.945066	3.494104	2.323649
17	6	0	1.003485	4.453494	2.715647
18	6	0	3.303849	3.829269	2.367262
19	6	0	1.408482	5.721919	3.128246
20	1	0	-0.057462	4.213345	2.693371
21	6	0	3.711572	5.096952	2.779566
22	1	0	4.053179	3.098316	2.070712
23	6	0	2.764356	6.047896	3.159387
24	1	0	0.664516	6.455471	3.423808
25	1	0	4.769103	5.341922	2.802485
26	1	0	3.080879	7.035665	3.479452

27	1	0	2.716768	4.022087	-1.664421
28	1	0	0.490875	4.627061	-1.336512

34, M06-2X/6-31+G(d,p), $CHCl_3$ IEFPCM:
Sum of electronic and thermal Free Energies= -882.174258

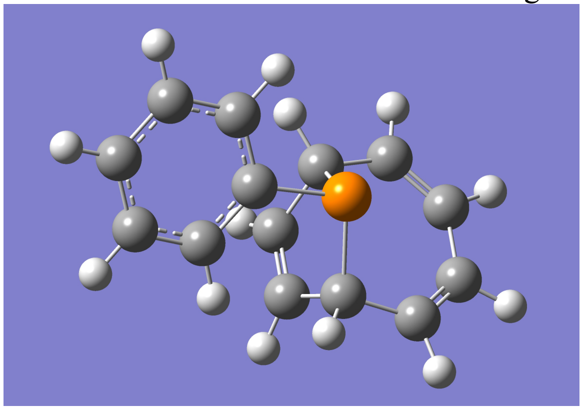

Center Number	Atomic Number	Atomic Type	Coordinates (Angstroms) X	Y	Z
1	6	0	-0.500850	0.871200	-0.126434
2	6	0	0.710216	0.329955	-0.284754
3	6	0	0.508705	3.626783	-1.185091
4	6	0	1.848775	1.112902	0.341091
5	6	0	1.842220	3.033744	-1.355211
6	6	0	2.440071	1.994062	-0.738269
7	1	0	0.908762	-0.538979	-0.907215
8	1	0	-1.399973	0.488990	-0.602685
9	1	0	2.631843	0.476128	0.760749
10	1	0	3.446790	1.742056	-1.065244
11	6	0	-0.528480	2.174375	0.645638
12	1	0	-1.399634	2.263957	1.299939
13	6	0	-0.507654	3.301568	-0.360264
14	1	0	-1.415247	3.896852	-0.438847
15	15	0	1.053247	2.164812	1.676319
16	1	0	2.436329	3.526060	-2.122065
17	1	0	0.322972	4.465987	-1.851791
18	6	0	0.567326	0.892133	2.924299
19	6	0	-0.135286	1.362911	4.043363
20	6	0	0.880218	-0.470951	2.859600
21	6	0	-0.536040	0.495477	5.057714
22	1	0	-0.369077	2.422705	4.124567
23	6	0	0.494563	-1.338712	3.881812
24	1	0	1.419144	-0.872342	2.007314
25	6	0	-0.218530	-0.860481	4.980027
26	1	0	-1.085709	0.880029	5.911336

27	1	0	0.748080	-2.392398	3.814770
28	1	0	-0.520640	-1.538613	5.771987

33[O], M06-2X/6-31+G(d,p), $CHCl_3$ IEFPCM:
Sum of electronic and thermal Free Energies= -957.399963

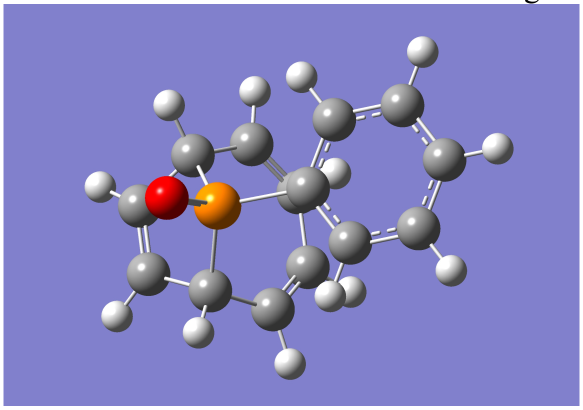

Center Number	Atomic Number	Atomic Type	Coordinates (Angstroms) X	Y	Z
1	6	0	0.140700	0.735587	-0.050820
2	6	0	1.419095	0.386990	-0.239835
3	6	0	0.762928	3.704209	-0.842807
4	6	0	2.450122	1.262903	0.453374
5	6	0	2.166275	3.321226	-1.051557
6	6	0	2.903986	2.324948	-0.524634
7	1	0	1.735026	-0.414437	-0.900416
8	1	0	-0.696725	0.248427	-0.540567
9	1	0	3.300568	0.688927	0.829450
10	1	0	3.935275	2.243592	-0.860218
11	6	0	-0.086636	1.954201	0.829140
12	1	0	-0.971166	1.852279	1.462516
13	6	0	-0.197791	3.170812	-0.063842
14	1	0	-1.178911	3.638771	-0.099313
15	15	0	1.437011	1.882373	1.861985
16	6	0	1.961811	3.545416	2.341988
17	6	0	1.011985	4.486728	2.750085
18	6	0	3.322791	3.860312	2.398397
19	6	0	1.421002	5.738534	3.204598
20	1	0	-0.048209	4.247912	2.709808
21	6	0	3.730701	5.112456	2.852997
22	1	0	4.066877	3.132482	2.083308
23	6	0	2.779667	6.051119	3.253813
24	1	0	0.681662	6.468205	3.518529
25	1	0	4.787526	5.355457	2.893547
26	1	0	3.097957	7.027038	3.606661

27	1	0	2.686033	3.961614	-1.760323
28	1	0	0.461124	4.569178	-1.428738
29	8	0	1.345257	0.923212	3.019582

34[O], M06-2X/6-31+G(d,p), $CHCl_3$ IEFPCM:
Sum of electronic and thermal Free Energies= -957.402798

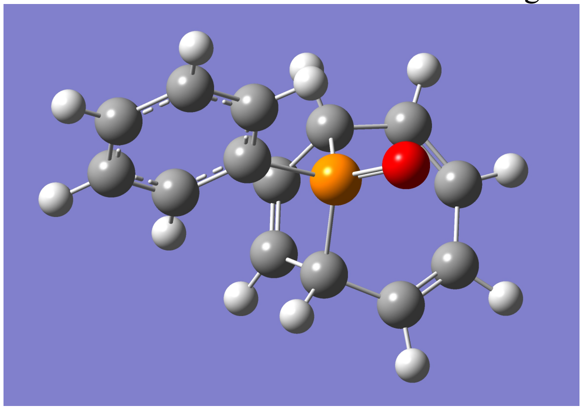

Center Number	Atomic Number	Atomic Type	Coordinates (Angstroms) X	Y	Z
1	6	0	1.165394	0.311540	1.754296
2	6	0	1.193318	1.435827	1.025971
3	6	0	3.418904	-0.966781	-0.030619
4	6	0	1.108365	1.254231	-0.475959
5	6	0	3.444512	0.267048	-0.829252
6	6	0	2.507298	1.208724	-1.050319
7	1	0	1.329040	2.425137	1.453810
8	1	0	1.277519	0.295323	2.834292
9	1	0	0.518707	2.033899	-0.965058
10	1	0	2.783281	2.043868	-1.689013
11	6	0	1.061777	-0.977870	0.969253
12	1	0	0.434832	-1.724976	1.461757
13	6	0	2.449650	-1.524727	0.720374
14	1	0	2.688459	-2.458071	1.224113
15	15	0	0.291631	-0.394610	-0.606057
16	1	0	4.398211	0.436730	-1.323560
17	1	0	4.357296	-1.515527	-0.061186
18	6	0	-1.484179	-0.151932	-0.292368
19	6	0	-2.335501	-1.122181	-0.834696
20	6	0	-2.028653	0.926105	0.416398
21	6	0	-3.714236	-1.025855	-0.656000
22	1	0	-1.909777	-1.940114	-1.408388
23	6	0	-3.408500	1.022054	0.587253
24	1	0	-1.385527	1.692577	0.836388
25	6	0	-4.251372	0.045537	0.056290

26	1	0	-4.367061	-1.782211	-1.079739
27	1	0	-3.824800	1.862600	1.133211
28	1	0	-5.325473	0.124380	0.191859
29	8	0	0.527285	-1.227318	-1.832878

Table S24. Coordinates of optimized structures for 1a, 11–34[O], PBE0 (pbe1pbe)/6-31+G(d), no solvent.

1a isomer A, PBE0/6-31+G(d):
Sum of electronic and thermal Free Energies= -1309.012606

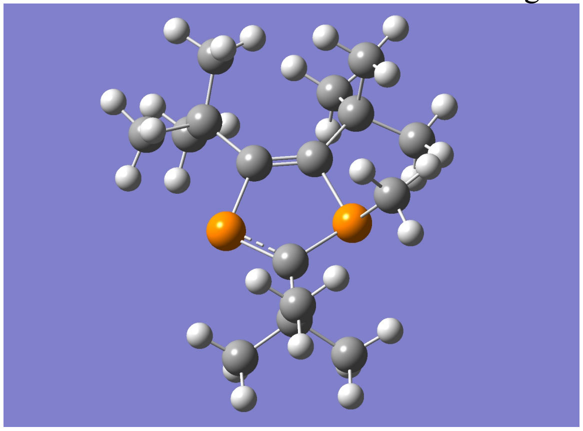

Center Number	Atomic Number	Atomic Type	Coordinates (Angstroms) X	Y	Z
1	6	0	-2.368473	4.066309	0.469600
2	6	0	-1.066792	4.316336	0.091727
3	6	0	-2.133592	6.712184	0.922579
4	15	0	-3.280468	5.493174	1.182823
5	15	0	-0.572149	6.051972	0.383114
6	6	0	-3.219599	2.766765	0.473175
7	6	0	-3.050254	1.910453	-0.791657
8	6	0	-2.945738	1.951349	1.749889
9	6	0	-4.728930	3.100761	0.495960
10	1	0	-3.299677	2.496239	-1.684354
11	1	0	-2.052819	1.498727	-0.928452
12	1	0	-3.744013	1.061845	-0.747898
13	1	0	-3.176074	2.552030	2.637632
14	1	0	-3.587978	1.060947	1.772275
15	1	0	-1.908454	1.624155	1.833044
16	1	0	-5.296644	2.167483	0.397836
17	1	0	-5.048166	3.578474	1.426705
18	1	0	-5.015912	3.754565	-0.336644
19	6	0	0.081228	3.394832	-0.405294
20	6	0	0.179886	2.057174	0.347529
21	6	0	-0.043827	3.163223	-1.923256
22	6	0	1.464812	4.040678	-0.167654
23	1	0	0.292519	2.231901	1.423566
24	1	0	-0.669629	1.394141	0.200398
25	1	0	1.071726	1.518158	0.003734
26	1	0	0.027057	4.111356	-2.467612

27	1	0	0.771585	2.516490	-2.272399
28	1	0	-0.989987	2.697017	-2.202151
29	1	0	2.239450	3.357526	-0.537164
30	1	0	1.605798	4.987140	-0.696109
31	1	0	1.651619	4.217699	0.896460
32	6	0	-2.372244	8.180955	1.264025
33	6	0	-3.432338	8.327529	2.364519
34	6	0	-1.081084	8.855523	1.749972
35	6	0	-2.884331	8.912874	0.008545
36	1	0	-4.393824	7.897795	2.061541
37	1	0	-3.115593	7.827740	3.286737
38	1	0	-3.595974	9.389559	2.586053
39	1	0	-0.295596	8.845867	0.985185
40	1	0	-1.280455	9.903590	2.005882
41	1	0	-0.685174	8.354061	2.639756
42	1	0	-3.061968	9.971846	0.236834
43	1	0	-2.166544	8.862984	-0.816583
44	1	0	-3.825899	8.472108	-0.336639
45	6	0	-0.312117	6.785945	-1.310157
46	1	0	-1.185960	6.623301	-1.948509
47	1	0	-0.141818	7.861295	-1.201816
48	1	0	0.570967	6.359146	-1.792227

1a isomer B, PBE0/6-31+G(d):
Sum of electronic and thermal Free Energies= -1309.010347

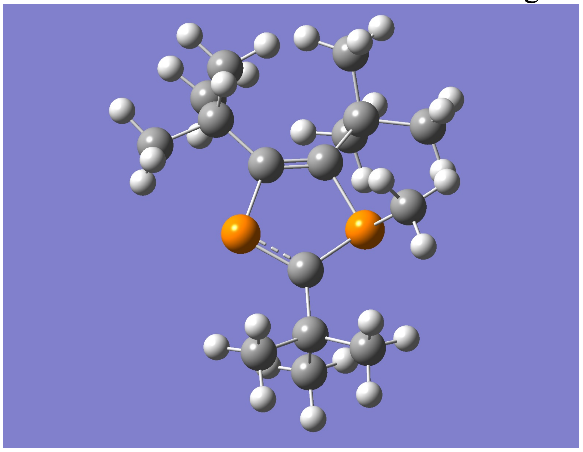

Center Number	Atomic Number	Atomic Type	Coordinates (Angstroms) X	Y	Z
1	6	0	-2.402823	4.032915	0.383253
2	6	0	-1.101506	4.276118	0.003288
3	6	0	-2.192753	6.697919	0.732117
4	15	0	-3.356011	5.493441	0.964834

5	15	0	-0.622277	6.044025	0.169149
6	6	0	-3.233556	2.719583	0.408061
7	6	0	-4.620901	2.922111	1.055938
8	6	0	-3.529705	2.277054	-1.037469
9	6	0	-2.590228	1.595097	1.235177
10	1	0	-4.553171	3.231519	2.105011
11	1	0	-5.233225	3.659859	0.526854
12	1	0	-5.159950	1.967175	1.023800
13	1	0	-2.632357	2.098770	-1.628144
14	1	0	-4.125449	1.354839	-1.032862
15	1	0	-4.114785	3.049337	-1.550905
16	1	0	-3.263448	0.728849	1.257403
17	1	0	-1.631852	1.248494	0.853678
18	1	0	-2.439935	1.924010	2.270384
19	6	0	0.098976	3.331565	-0.281187
20	6	0	1.268869	4.061836	-0.972274
21	6	0	0.646569	2.861219	1.082618
22	6	0	-0.180015	2.133930	-1.201340
23	1	0	1.028006	4.328732	-2.006029
24	1	0	1.589163	4.961035	-0.433283
25	1	0	2.131636	3.386325	-1.005829
26	1	0	-0.104751	2.342231	1.680048
27	1	0	1.498306	2.184257	0.932535
28	1	0	0.996626	3.721667	1.664435
29	1	0	0.761871	1.599598	-1.377443
30	1	0	-0.885729	1.408808	-0.800880
31	1	0	-0.551205	2.471444	-2.175748
32	6	0	-2.372822	8.132796	1.224205
33	6	0	-1.675807	9.155865	0.317765
34	6	0	-3.857948	8.504354	1.317263
35	6	0	-1.750445	8.236358	2.631132
36	1	0	-0.593891	8.983102	0.265999
37	1	0	-2.086159	9.138587	-0.698253
38	1	0	-1.824506	10.165415	0.719923
39	1	0	-4.397642	7.849154	2.009943
40	1	0	-3.962982	9.534271	1.679971
41	1	0	-4.346667	8.434418	0.338712
42	1	0	-1.847029	9.261796	3.011677
43	1	0	-2.254331	7.558760	3.328697
44	1	0	-0.686472	7.975796	2.611357
45	6	0	-0.561326	6.634960	-1.600286
46	1	0	0.361463	6.313234	-2.085308
47	1	0	-1.422679	6.261036	-2.162768
48	1	0	-0.579110	7.725988	-1.606566

----

11 isomer A, PBE0/6-31+G(d):
Sum of electronic and thermal Free Energies= -1090.612361

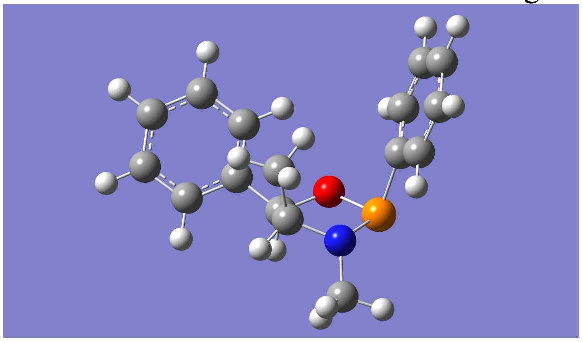

Center Number	Atomic Number	Atomic Type	Coordinates (Angstroms) X	Y	Z
1	6	0	0.259857	1.184018	1.161729
2	6	0	1.008754	0.947408	-0.179906
3	1	0	0.804563	1.943803	1.736711
4	1	0	1.356583	1.929156	-0.541152
5	15	0	-1.492120	1.093349	-0.761536
6	8	0	0.037642	0.453731	-1.100737
7	7	0	-1.032185	1.765861	0.759861
8	6	0	2.193594	0.023194	-0.100712
9	6	0	2.089262	-1.335522	-0.404382
10	6	0	3.424317	0.529566	0.327333
11	6	0	3.193938	-2.173902	-0.269120
12	1	0	1.138637	-1.727021	-0.753897
13	6	0	4.527648	-0.308476	0.468846
14	1	0	3.522447	1.591687	0.547809
15	6	0	4.414690	-1.665712	0.171422
16	1	0	3.100640	-3.229634	-0.512254
17	1	0	5.478238	0.100642	0.802286
18	1	0	5.275687	-2.321339	0.274489
19	6	0	0.099662	-0.062239	2.020049
20	1	0	-0.362551	-0.881326	1.462665
21	1	0	1.073516	-0.405366	2.384409
22	1	0	-0.539384	0.165126	2.879209
23	6	0	-2.338953	-0.465751	-0.266037
24	6	0	-3.296123	-0.452789	0.753653
25	6	0	-2.144697	-1.639145	-1.003676
26	6	0	-4.026820	-1.601453	1.050231
27	1	0	-3.449212	0.454236	1.334695
28	6	0	-2.874957	-2.788034	-0.705584
29	1	0	-1.408303	-1.658067	-1.804290
30	6	0	-3.817282	-2.771847	0.321977
31	1	0	-4.758101	-1.584377	1.855002

32	1	0	-2.708175	-3.698006	-1.277283
33	1	0	-4.387718	-3.667995	0.553272
34	6	0	-1.107994	3.217828	0.872556
35	1	0	-2.042364	3.572852	0.428770
36	1	0	-1.109251	3.502601	1.931193
37	1	0	-0.275494	3.741526	0.373317

----

11 isomer B, PBE0/6-31+G(d):
Sum of electronic and thermal Free Energies= -1090.607811

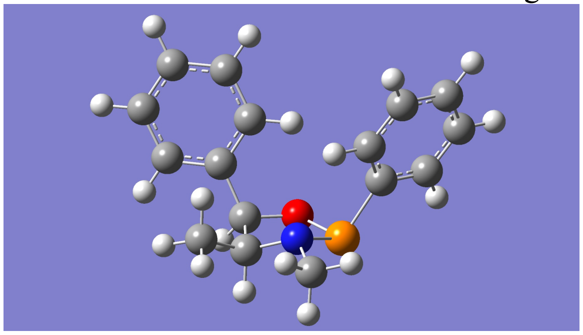

Center Number	Atomic Number	Atomic Type	Coordinates (Angstroms) X	Y	Z
1	6	0	0.325013	1.317717	0.371030
2	6	0	1.199334	0.099521	-0.031567
3	1	0	1.913188	0.439974	-0.792952
4	15	0	-1.303708	-0.453177	-0.558214
5	8	0	0.326473	-0.831728	-0.672263
6	7	0	-0.991285	0.756674	0.664873
7	6	0	1.994477	-0.553570	1.076064
8	6	0	3.301117	-0.119418	1.320881
9	6	0	1.473682	-1.585162	1.858731
10	6	0	4.060310	-0.676048	2.347373
11	1	0	3.735434	0.659412	0.695502
12	6	0	2.232264	-2.148530	2.882942
13	1	0	0.478912	-1.963896	1.650222
14	6	0	3.524611	-1.692452	3.136320
15	1	0	5.074770	-0.325454	2.521404
16	1	0	1.812356	-2.954887	3.479633
17	1	0	4.116393	-2.136215	3.933109
18	1	0	0.261998	1.971135	-0.523495
19	6	0	0.877519	2.145093	1.519320
20	1	0	0.909061	1.560017	2.443573
21	1	0	1.894157	2.484305	1.292049
22	1	0	0.267190	3.038791	1.685874
23	6	0	-2.050442	1.737212	0.834046
24	1	0	-3.012828	1.225766	0.941590

25	1	0	-1.886457	2.315504	1.749265
26	1	0	-2.126134	2.438832	-0.015638
27	6	0	-2.015647	-1.797517	0.484753
28	6	0	-2.563425	-2.895345	-0.189944
29	6	0	-2.101845	-1.744171	1.880624
30	6	0	-3.151140	-3.941841	0.517310
31	1	0	-2.529192	-2.930439	-1.278198
32	6	0	-2.709035	-2.779423	2.588015
33	1	0	-1.685560	-0.883947	2.400039
34	6	0	-3.226554	-3.882261	1.908152
35	1	0	-3.560578	-4.796528	-0.015540
36	1	0	-2.776823	-2.728409	3.672405
37	1	0	-3.696745	-4.691143	2.462289

----

12 isomer A, PBE0/6-31+G(d):
Sum of electronic and thermal Free Energies= -1090.614472

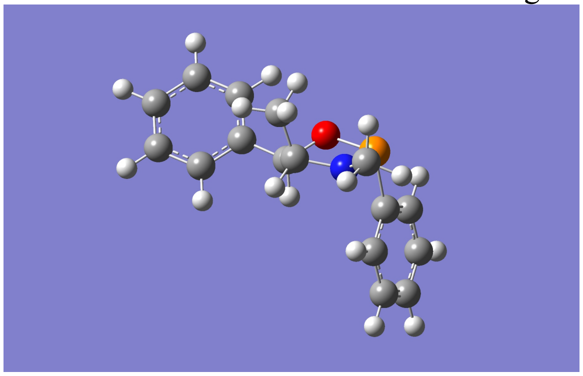

Center Number	Atomic Number	Atomic Type	Coordinates (Angstroms) X	Y	Z
1	6	0	-0.498069	1.495992	0.799726
2	6	0	-0.734406	0.067591	0.258501
3	1	0	-0.463686	1.449687	1.899696
4	1	0	-0.092819	-0.619678	0.831566
5	15	0	1.195673	0.915457	-1.146036
6	8	0	-0.265336	0.101533	-1.090804
7	7	0	0.840391	1.812558	0.293639
8	6	0	1.315846	3.176490	0.407918
9	1	0	2.377326	3.218031	0.142878
10	1	0	0.779439	3.894795	-0.231835
11	1	0	1.229547	3.510250	1.449546
12	6	0	2.348177	-0.374251	-0.484342
13	6	0	2.800685	-0.402179	0.838014
14	6	0	2.830670	-1.330902	-1.386820
15	6	0	3.701269	-1.380512	1.257588

16	1	0	2.439512	0.355044	1.530941
17	6	0	3.720374	-2.316494	-0.967618
18	1	0	2.504656	-1.306468	-2.426065
19	6	0	4.157922	-2.341302	0.356905
20	1	0	4.047011	-1.393830	2.288798
21	1	0	4.080189	-3.060235	-1.674570
22	1	0	4.860418	-3.104296	0.683522
23	6	0	-2.154940	-0.422228	0.318264
24	6	0	-2.969890	-0.446713	-0.814385
25	6	0	-2.678177	-0.849854	1.542279
26	6	0	-4.291034	-0.881412	-0.720238
27	1	0	-2.557532	-0.133446	-1.768713
28	6	0	-3.999745	-1.277466	1.638164
29	1	0	-2.044404	-0.854437	2.427842
30	6	0	-4.811643	-1.293751	0.504614
31	1	0	-4.915207	-0.900440	-1.610424
32	1	0	-4.392866	-1.607667	2.596606
33	1	0	-5.841875	-1.633530	0.575331
34	6	0	-1.573335	2.490900	0.373892
35	1	0	-1.667887	2.524680	-0.717395
36	1	0	-2.544139	2.208248	0.793466
37	1	0	-1.338514	3.499430	0.730153

----

12 isomer B, PBE0/6-31+G(d):
Sum of electronic and thermal Free Energies= -1090.612567

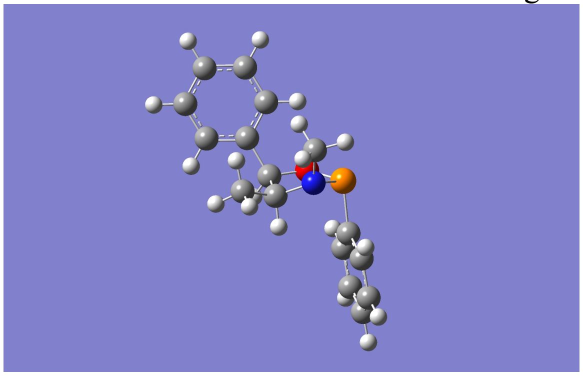

Center Number	Atomic Number	Atomic Type	Coordinates (Angstroms) X	Y	Z
1	6	0	-0.237384	0.637921	0.783427
2	6	0	-0.939599	-0.643942	0.207596
3	1	0	0.651910	0.265982	1.308767
4	1	0	-0.750805	-1.469523	0.907191
5	15	0	0.827864	0.235289	-1.488301
6	8	0	-0.279129	-0.950264	-1.022174

7	7	0	0.273391	1.401224	-0.363435
8	6	0	2.320565	-0.378622	-0.591133
9	6	0	3.303860	0.524066	-0.170099
10	6	0	2.546862	-1.750776	-0.439022
11	6	0	4.482070	0.063797	0.413501
12	1	0	3.135520	1.593475	-0.281256
13	6	0	3.727552	-2.211021	0.142265
14	1	0	1.787645	-2.458154	-0.765134
15	6	0	4.696704	-1.305399	0.570413
16	1	0	5.233828	0.774873	0.748159
17	1	0	3.889462	-3.279626	0.263208
18	1	0	5.617375	-1.664845	1.023469
19	6	0	-2.431228	-0.544165	-0.022091
20	6	0	-2.960427	-0.214670	-1.272055
21	6	0	-3.313590	-0.794020	1.033765
22	6	0	-4.337791	-0.104091	-1.453115
23	1	0	-2.285812	-0.069856	-2.111222
24	6	0	-4.690587	-0.682953	0.857009
25	1	0	-2.920626	-1.088633	2.005671
26	6	0	-5.208075	-0.331286	-0.388664
27	1	0	-4.732527	0.149787	-2.434065
28	1	0	-5.360300	-0.882930	1.689946
29	1	0	-6.282621	-0.250206	-0.532075
30	6	0	-1.051259	1.456496	1.767598
31	1	0	-1.983017	1.831478	1.334347
32	1	0	-1.314460	0.843949	2.636583
33	1	0	-0.459913	2.305204	2.127185
34	6	0	-0.529917	2.506007	-0.879582
35	1	0	-0.528698	3.332877	-0.161392
36	1	0	-0.068329	2.869375	-1.802023
37	1	0	-1.572806	2.238147	-1.099195

----

12 isomer C, PBE0/6-31+G(d):
Sum of electronic and thermal Free Energies= -1090.613769

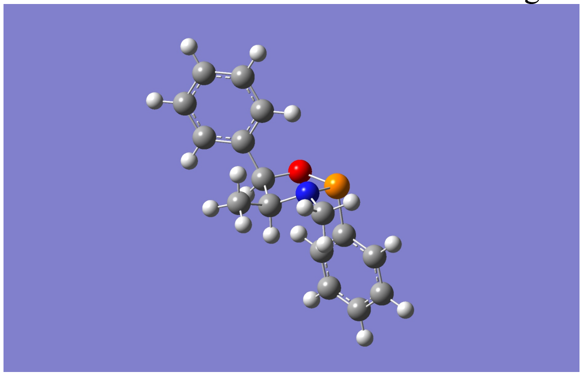

Center Number	Atomic Number	Atomic Type	Coordinates (Angstroms) X	Y	Z
1	6	0	-0.212504	0.492706	0.928727
2	6	0	-0.907716	-0.723945	0.266395
3	1	0	0.650165	0.107230	1.505741
4	1	0	-0.784341	-1.597915	0.919301
5	15	0	0.902740	0.177195	-1.386980
6	8	0	-0.200407	-0.993583	-0.955187
7	7	0	0.258850	1.272405	-0.210677
8	6	0	2.432151	-0.496746	-0.580990
9	6	0	3.623152	0.216658	-0.766982
10	6	0	2.468003	-1.684562	0.158090
11	6	0	4.815183	-0.222558	-0.194636
12	1	0	3.622857	1.121230	-1.375314
13	6	0	3.661605	-2.133684	0.720659
14	1	0	1.558952	-2.269887	0.272097
15	6	0	4.834997	-1.399734	0.552284
16	1	0	5.730785	0.345111	-0.342677
17	1	0	3.677431	-3.061116	1.288586
18	1	0	5.765730	-1.751314	0.990678
19	6	0	-2.379334	-0.550290	-0.021194
20	6	0	-2.827256	0.222766	-1.096387
21	6	0	-3.321593	-1.158243	0.810896
22	6	0	-4.190310	0.391907	-1.322911
23	1	0	-2.102495	0.687335	-1.758505
24	6	0	-4.687311	-0.988028	0.588892
25	1	0	-2.984676	-1.779672	1.639446
26	6	0	-5.124963	-0.209754	-0.479806
27	1	0	-4.525474	0.993114	-2.164650
28	1	0	-5.407146	-1.472566	1.244048
29	1	0	-6.188868	-0.079761	-0.661986
30	6	0	-1.102406	1.286007	1.872075
31	1	0	-1.920952	1.768775	1.329441
32	1	0	-1.538242	0.623844	2.628517
33	1	0	-0.525710	2.051593	2.401600
34	6	0	0.988704	2.489295	0.073545
35	1	0	1.875104	2.322650	0.709950
36	1	0	1.321325	2.939088	-0.868563
37	1	0	0.335977	3.213526	0.572253

13, PBE0/6-31+G(d):
Sum of electronic and thermal Free Energies= -1791.374119

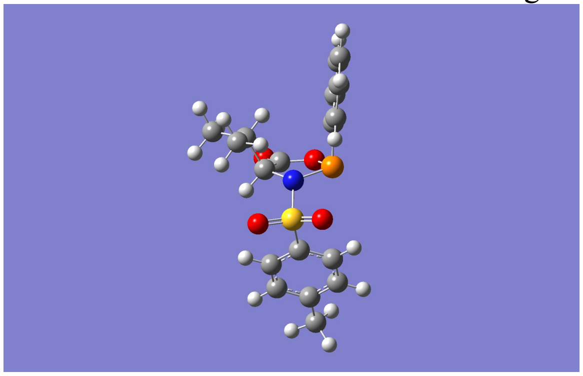

Center Number	Atomic Number	Atomic Type	Coordinates (Angstroms) X	Y	Z
1	15	0	0.683680	-1.200873	-0.508069
2	16	0	-0.946582	-0.114492	1.672874
3	7	0	0.285846	0.137111	0.548358
4	8	0	0.439205	-0.244967	-1.889170
5	8	0	0.086172	1.867074	-2.539026
6	8	0	-0.618972	-1.399092	2.277839
7	8	0	-1.043898	1.115546	2.446222
8	6	0	0.277881	1.088632	-1.647343
9	6	0	0.382633	1.419377	-0.155633
10	6	0	1.682700	2.206681	0.158146
11	6	0	1.944851	2.255799	1.659773
12	6	0	1.601342	3.617754	-0.423165
13	6	0	-6.061178	-0.742084	-1.536206
14	6	0	3.121224	-1.436738	0.804641
15	6	0	4.508509	-1.497767	0.897694
16	6	0	5.295451	-1.284291	-0.234428
17	6	0	4.692421	-1.011822	-1.460559
18	6	0	3.303191	-0.947107	-1.559376
19	6	0	2.513668	-1.145295	-0.422545
20	6	0	-3.254169	0.816985	0.515994
21	6	0	-4.418402	0.667509	-0.232270
22	6	0	-4.801300	-0.579830	-0.734106
23	6	0	-3.987968	-1.687853	-0.460679
24	6	0	-2.821158	-1.560448	0.281567
25	6	0	-2.458583	-0.300032	0.759674
26	1	0	-0.474488	2.047796	0.114250
27	1	0	2.517797	1.676053	-0.319505
28	1	0	2.860912	2.826266	1.851375
29	1	0	2.071835	1.256012	2.084021

30	1	0	1.119427	2.741807	2.191785
31	1	0	2.540924	4.149752	-0.237084
32	1	0	0.797261	4.188057	0.060384
33	1	0	1.414495	3.617849	-1.500327
34	1	0	-6.568615	0.215412	-1.685729
35	1	0	-6.762039	-1.420292	-1.034943
36	1	0	-5.847314	-1.168562	-2.523177
37	1	0	2.509815	-1.602008	1.690360
38	1	0	4.976261	-1.711292	1.855387
39	1	0	6.378802	-1.333783	-0.160303
40	1	0	5.303229	-0.847176	-2.344627
41	1	0	2.835222	-0.730037	-2.516446
42	1	0	-2.977544	1.782230	0.929833
43	1	0	-5.043854	1.536801	-0.421485
44	1	0	-4.277843	-2.669044	-0.830702
45	1	0	-2.203336	-2.424515	0.506373

----

14 isomer A, PBE0/6-31+G(d):
Sum of electronic and thermal Free Energies= -1791.371252

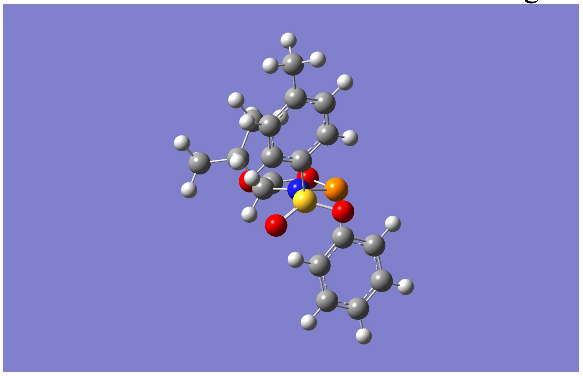

Center Number	Atomic Number	Atomic Type	Coordinates (Angstroms) X	Y	Z
1	15	0	-1.376772	-0.269382	-1.332926
2	16	0	0.452004	-0.988173	0.849403
3	7	0	-0.290242	0.207042	-0.031157
4	8	0	-1.696744	1.385664	-1.603583
5	8	0	-1.680709	3.391185	-0.614130
6	8	0	0.383438	-0.628556	2.261118
7	8	0	-0.158147	-2.207086	0.319891
8	6	0	-1.324186	2.245758	-0.626159
9	6	0	-0.373098	1.605430	0.385082
10	6	0	0.993121	2.328444	0.415110
11	6	0	0.887476	3.678083	1.121585
12	6	0	1.612834	2.455970	-0.972924

13	6	0	6.300997	-1.125704	-0.768518
14	6	0	2.512543	-1.436106	-0.900823
15	6	0	3.852623	-1.469595	-1.260041
16	6	0	4.856070	-1.084635	-0.358888
17	6	0	4.477771	-0.669293	0.920442
18	6	0	3.137513	-0.625044	1.298504
19	6	0	2.165552	-1.003966	0.379670
20	1	0	-0.835977	1.683987	1.378337
21	1	0	1.644391	1.689780	1.025828
22	1	0	1.882668	4.127850	1.212326
23	1	0	0.476492	3.568792	2.132329
24	1	0	0.245767	4.370436	0.568963
25	1	0	2.630388	2.854790	-0.896379
26	1	0	1.035509	3.142259	-1.604533
27	1	0	1.673196	1.486918	-1.480663
28	1	0	6.575455	-2.115020	-1.152220
29	1	0	6.963141	-0.893047	0.070624
30	1	0	6.503675	-0.401487	-1.566946
31	1	0	1.742727	-1.755777	-1.598151
32	1	0	4.128544	-1.808432	-2.256390
33	1	0	5.242149	-0.380536	1.638257
34	1	0	2.844952	-0.316274	2.297857
35	6	0	-2.926880	-0.722780	-0.467618
36	6	0	-3.919863	-1.299824	-1.266866
37	6	0	-3.167656	-0.533211	0.897163
38	6	0	-5.149200	-1.656467	-0.717016
39	1	0	-3.731796	-1.475167	-2.325197
40	6	0	-4.391615	-0.899446	1.447996
41	1	0	-2.391993	-0.125937	1.540798
42	6	0	-5.384894	-1.454861	0.640968
43	1	0	-5.916817	-2.100055	-1.345778
44	1	0	-4.569037	-0.757586	2.510942
45	1	0	-6.339512	-1.740796	1.075324

----

14 isomer B, PBE0/6-31+G(d):
Sum of electronic and thermal Free Energies= -1791.369465

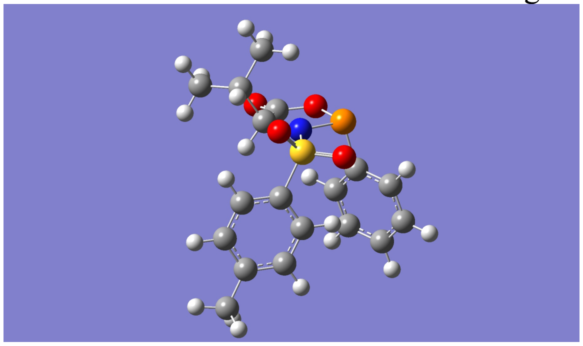

Center Number	Atomic Number	Atomic Type	Coordinates (Angstroms) X	Y	Z
1	15	0	1.352509	1.815568	-0.659531
2	16	0	0.285190	-0.749618	-1.684063
3	7	0	1.218695	0.047795	-0.571564
4	8	0	2.183859	1.775857	0.821232
5	8	0	2.682771	0.505919	2.591989
6	8	0	-0.153492	0.329381	-2.569075
7	8	0	1.004049	-1.908703	-2.195473
8	6	0	2.259368	0.588213	1.472306
9	6	0	1.804507	-0.581149	0.605334
10	6	0	2.981441	-1.536435	0.282775
11	6	0	3.388332	-2.349479	1.509038
12	6	0	4.165719	-0.795764	-0.330613
13	6	0	-4.464639	-2.880112	1.445240
14	6	0	-1.092155	-2.687929	-0.318258
15	6	0	-2.177713	-3.167686	0.409994
16	6	0	-3.283748	-2.355936	0.678272
17	6	0	-3.278299	-1.040214	0.195643
18	6	0	-2.203625	-0.541266	-0.528810
19	6	0	-1.111825	-1.373131	-0.777559
20	1	0	1.038600	-1.130755	1.173075
21	1	0	2.586221	-2.224767	-0.473858
22	1	0	4.147187	-3.087729	1.226497
23	1	0	2.535526	-2.893906	1.933454
24	1	0	3.805535	-1.714914	2.297056
25	1	0	4.925368	-1.511939	-0.661827
26	1	0	4.641589	-0.123153	0.394495
27	1	0	3.863856	-0.208823	-1.205221
28	1	0	-5.337963	-2.990587	0.790596
29	1	0	-4.749817	-2.195869	2.252286
30	1	0	-4.254584	-3.858230	1.888024
31	1	0	-0.247059	-3.329539	-0.550101
32	1	0	-2.166467	-4.194932	0.767242
33	1	0	-4.133548	-0.395536	0.387026
34	1	0	-2.212931	0.476405	-0.907256
35	6	0	-0.270491	2.424321	-0.050989
36	6	0	-1.070210	3.081369	-0.993413
37	6	0	-0.719846	2.305363	1.269573
38	6	0	-2.317687	3.585516	-0.630012
39	1	0	-0.720263	3.184878	-2.018608
40	6	0	-1.961881	2.817021	1.633617
41	1	0	-0.096567	1.831823	2.023803
42	6	0	-2.763237	3.452702	0.683883

43	1	0	-2.935554	4.088735	-1.369290
44	1	0	-2.302634	2.727717	2.662118
45	1	0	-3.731039	3.855273	0.972841

----

15 isomer A (cis, chair), PBE0/6-31+G(d):
Sum of electronic and thermal Free Energies= -900.544416

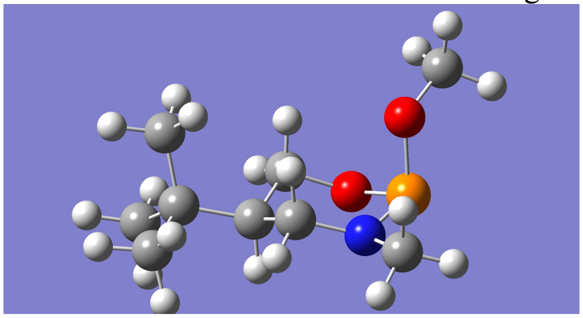

Center Number	Atomic Number	Atomic Type	Coordinates (Angstroms) X	Y	Z
1	6	0	0.270571	-1.221910	-0.518467
2	6	0	0.270092	1.142994	0.278746
3	6	0	1.085867	0.066246	-0.445593
4	1	0	0.018996	-1.584883	0.486124
5	1	0	0.805267	-2.012684	-1.049862
6	1	0	0.800124	2.101307	0.225140
7	1	0	0.155421	0.893627	1.347909
8	1	0	1.216725	0.416256	-1.481086
9	6	0	2.510461	-0.148318	0.145630
10	6	0	2.480934	-0.867194	1.501732
11	1	0	3.494315	-0.919854	1.918714
12	1	0	1.852900	-0.344026	2.232019
13	1	0	2.113084	-1.896084	1.416204
14	6	0	3.346858	-0.981524	-0.836638
15	1	0	3.415313	-0.489349	-1.814813
16	1	0	4.367182	-1.106653	-0.453907
17	1	0	2.935732	-1.984757	-0.992842
18	6	0	3.217240	1.202282	0.323433
19	1	0	4.270574	1.042679	0.584212
20	1	0	3.189139	1.794521	-0.600294
21	1	0	2.772898	1.801023	1.126270
22	15	0	-2.053561	0.050084	-0.701164
23	8	0	-0.940504	-1.029334	-1.256248
24	7	0	-1.031707	1.356166	-0.344831
25	6	0	-1.700484	2.565078	0.097509
26	1	0	-1.087351	3.440515	-0.152085
27	1	0	-2.661487	2.667406	-0.419107

28	1	0	-1.891656	2.570667	1.182726
29	8	0	-2.309070	-0.521551	0.861647
30	6	0	-3.254373	-1.566953	1.013154
31	1	0	-2.823861	-2.534910	0.725733
32	1	0	-3.537842	-1.601875	2.068581
33	1	0	-4.154000	-1.387403	0.407656

----

15 isomer B (cis, twist 1), PBE0/6-31+G(d):
Sum of electronic and thermal Free Energies= -900.537471

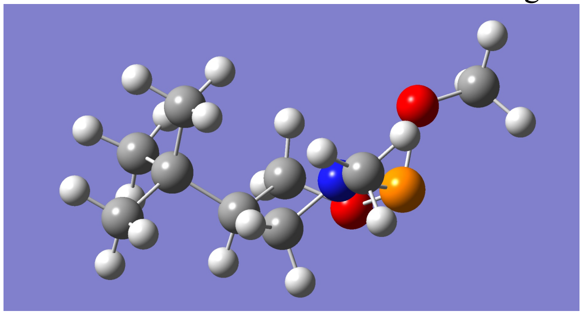

Center Number	Atomic Number	Atomic Type	Coordinates (Angstroms) X	Y	Z
1	6	0	-0.059545	-1.244876	-0.188348
2	6	0	0.161421	1.200973	0.280975
3	6	0	0.853684	-0.026200	-0.349702
4	1	0	0.052134	-1.701251	0.801655
5	1	0	0.147239	-2.003321	-0.946712
6	1	0	-0.354054	1.784716	-0.502197
7	1	0	0.907140	1.877986	0.712288
8	1	0	0.939741	0.171972	-1.428394
9	6	0	2.299507	-0.286330	0.162630
10	6	0	2.373255	-0.357391	1.692529
11	1	0	3.406064	-0.554897	2.006578
12	1	0	2.060890	0.581856	2.162259
13	1	0	1.743058	-1.153227	2.103010
14	6	0	2.822438	-1.605744	-0.422896
15	1	0	2.734821	-1.619852	-1.517092
16	1	0	3.882388	-1.738309	-0.173518
17	1	0	2.285709	-2.475663	-0.027280
18	6	0	3.218162	0.839127	-0.330699
19	1	0	4.247230	0.669288	0.009204
20	1	0	3.231748	0.886493	-1.426980
21	1	0	2.911606	1.823181	0.042932
22	15	0	-2.159929	-0.015798	0.825775
23	8	0	-1.428781	-0.869542	-0.378123
24	7	0	-0.748705	0.822866	1.359593

25	6	0	-0.995627	1.868393	2.334820
26	1	0	-0.052827	2.141152	2.825088
27	1	0	-1.433030	2.782627	1.896978
28	1	0	-1.673262	1.496553	3.109728
29	8	0	-2.210076	-1.056182	2.124538
30	6	0	-3.406742	-1.799266	2.311255
31	1	0	-3.387627	-2.719547	1.715438
32	1	0	-3.463735	-2.061038	3.370979
33	1	0	-4.295279	-1.215397	2.036097

----

15 isomer C (cis, twist 2), PBE0/6-31+G(d):
Sum of electronic and thermal Free Energies= -900.537248

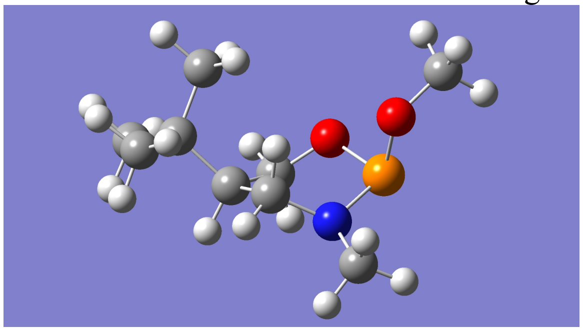

Center Number	Atomic Number	Atomic Type	Coordinates (Angstroms) X	Y	Z
1	6	0	0.692772	-1.254068	-1.372256
2	6	0	0.542559	1.175822	-0.776572
3	6	0	1.509722	-0.002472	-1.017274
4	1	0	1.215641	-2.174523	-1.099140
5	1	0	0.513931	-1.288979	-2.454884
6	1	0	1.027935	2.115768	-1.059869
7	1	0	0.284051	1.259925	0.288568
8	1	0	2.103270	0.241399	-1.912294
9	6	0	2.527639	-0.199807	0.139528
10	6	0	1.864935	-0.763755	1.402145
11	1	0	2.611536	-0.873834	2.198949
12	1	0	1.069742	-0.111821	1.779034
13	1	0	1.419885	-1.748578	1.222764
14	6	0	3.632643	-1.162721	-0.315516
15	1	0	4.149851	-0.780140	-1.204895
16	1	0	4.380659	-1.288020	0.477038
17	1	0	3.246274	-2.160172	-0.554655
18	6	0	3.192022	1.143437	0.471965
19	1	0	3.991375	1.000451	1.209440
20	1	0	3.642956	1.596173	-0.421180
21	1	0	2.483242	1.862888	0.896834

22	15	0	-1.727794	-0.215052	-1.156099
23	8	0	-0.553325	-1.299736	-0.680540
24	7	0	-0.669106	1.037963	-1.582625
25	6	0	-1.256958	2.264069	-2.083926
26	1	0	-2.148943	2.026312	-2.674707
27	1	0	-1.555518	2.953099	-1.278551
28	1	0	-0.545308	2.780440	-2.739890
29	8	0	-2.217024	0.272408	0.362515
30	6	0	-3.243478	-0.473511	0.996152
31	1	0	-2.827082	-1.353812	1.500259
32	1	0	-3.711406	0.177631	1.739260
33	1	0	-4.007083	-0.800833	0.276420

----

16 isomer A (trans, chair), PBE0/6-31+G(d):
Sum of electronic and thermal Free Energies= -900.533284

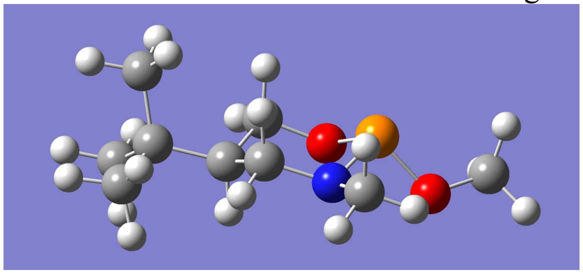

Center Number	Atomic Number	Atomic Type	Coordinates (Angstroms) X	Y	Z
1	6	0	0.463058	-1.311382	0.392025
2	6	0	0.447419	1.160055	0.072820
3	6	0	1.153242	-0.144362	-0.313882
4	1	0	0.525543	-1.195898	1.486457
5	1	0	0.909019	-2.274156	0.131373
6	1	0	0.868164	1.993007	-0.501688
7	1	0	0.630966	1.391374	1.141442
8	1	0	0.980094	-0.285343	-1.390800
9	6	0	2.695309	-0.119175	-0.095652
10	6	0	3.082039	-0.167844	1.389021
11	1	0	4.170334	-0.077751	1.494574
12	1	0	2.629195	0.650791	1.960362
13	1	0	2.788328	-1.112604	1.860801
14	6	0	3.324332	-1.325490	-0.808557
15	1	0	3.095419	-1.314036	-1.881390
16	1	0	4.415622	-1.303249	-0.699489
17	1	0	2.978887	-2.281952	-0.401308
18	6	0	3.296354	1.148802	-0.717534
19	1	0	4.391400	1.085871	-0.713245

20	1	0	2.973979	1.275715	-1.758980
21	1	0	3.022687	2.053751	-0.163459
22	15	0	-1.855640	-0.148425	0.532387
23	8	0	-0.902136	-1.397543	-0.006178
24	7	0	-0.984409	1.137367	-0.230446
25	6	0	-1.600119	2.442943	-0.056375
26	1	0	-1.123072	3.156722	-0.738991
27	1	0	-2.660391	2.390166	-0.319773
28	1	0	-1.508864	2.838785	0.971181
29	6	0	-4.259063	-0.924574	-0.180841
30	1	0	-4.489400	-0.778837	0.882512
31	1	0	-5.076852	-0.524989	-0.785385
32	1	0	-4.151997	-1.996127	-0.382963
33	8	0	-3.078977	-0.229120	-0.563609

----

16 isomer B (trans, twist 1), PBE0/6-31+G(d):
Sum of electronic and thermal Free Energies= -900.540373

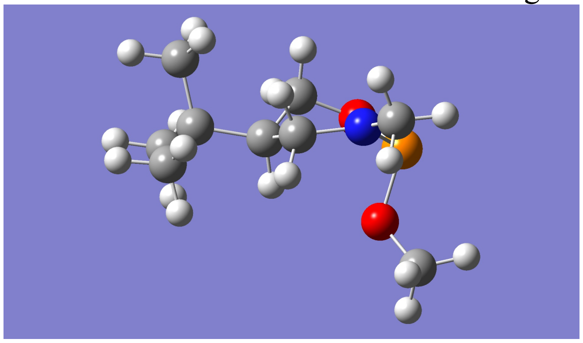

Center Number	Atomic Number	Atomic Type	Coordinates (Angstroms) X	Y	Z
1	6	0	0.675445	-1.614327	1.086880
2	6	0	0.474095	0.858604	1.051254
3	6	0	0.954262	-0.363948	0.256566
4	1	0	1.082049	-1.503647	2.101493
5	1	0	1.112413	-2.509937	0.638052
6	1	0	0.295069	1.708634	0.373632
7	1	0	1.252943	1.175802	1.763522
8	1	0	0.331812	-0.443571	-0.645039
9	6	0	2.429751	-0.248919	-0.221056
10	6	0	3.426553	-0.449943	0.927847
11	1	0	4.451493	-0.298185	0.567601
12	1	0	3.261037	0.257997	1.748713
13	1	0	3.373030	-1.463671	1.341804
14	6	0	2.697519	-1.307333	-1.300000
15	1	0	2.026741	-1.174116	-2.157516

16	1	0	3.728608	-1.225672	-1.665373
17	1	0	2.564764	-2.328860	-0.926922
18	6	0	2.666097	1.129697	-0.851676
19	1	0	3.668098	1.174848	-1.295765
20	1	0	1.939062	1.332986	-1.648122
21	1	0	2.599642	1.939593	-0.116216
22	15	0	-1.803756	-0.676810	1.412579
23	8	0	-0.721850	-1.893022	1.185920
24	7	0	-0.727747	0.571126	1.821882
25	6	0	-3.292508	0.325805	-0.514446
26	1	0	-3.089352	1.402456	-0.429469
27	1	0	-3.580389	0.103959	-1.545666
28	1	0	-4.131638	0.072270	0.149420
29	8	0	-2.141009	-0.443687	-0.222195
30	6	0	-1.198867	1.671424	2.633769
31	1	0	-0.439921	1.947410	3.379364
32	1	0	-1.424589	2.567383	2.032600
33	1	0	-2.108178	1.377184	3.167895

----

16 isomer C (trans, twist 2), PBE0/6-31+G(d):
Sum of electronic and thermal Free Energies= -900.539625

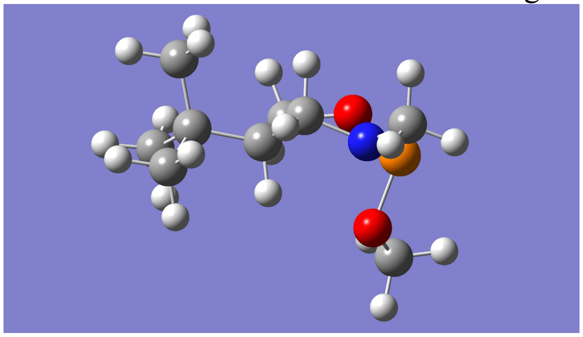

Center Number	Atomic Number	Atomic Type	Coordinates (Angstroms) X	Y	Z
1	6	0	0.088340	-1.251031	0.474325
2	6	0	0.339575	1.132046	-0.230989
3	6	0	0.859987	-0.292063	-0.439710
4	1	0	0.514773	-1.259860	1.484023
5	1	0	0.124103	-2.272545	0.079968
6	1	0	0.952245	1.847626	-0.794858
7	1	0	0.415634	1.412558	0.835179
8	1	0	0.620905	-0.562012	-1.476916
9	6	0	2.403195	-0.424547	-0.285120
10	6	0	2.915494	0.196562	1.020397
11	1	0	3.995393	0.029109	1.116249

12	1	0	2.748959	1.279567	1.049196
13	1	0	2.437850	-0.240677	1.905184
14	6	0	2.799262	-1.907387	-0.321033
15	1	0	2.408878	-2.402241	-1.219477
16	1	0	3.891618	-2.006218	-0.334757
17	1	0	2.434927	-2.456644	0.554707
18	6	0	3.094530	0.269747	-1.466520
19	1	0	4.182508	0.150007	-1.393350
20	1	0	2.773588	-0.163172	-2.421887
21	1	0	2.888343	1.345432	-1.498632
22	15	0	-2.125523	-0.008310	-0.449796
23	8	0	-1.290005	-0.900216	0.661702
24	7	0	-1.034795	1.267518	-0.687468
25	6	0	-2.625407	-2.020055	-2.057374
26	1	0	-3.683541	-1.810412	-1.845730
27	1	0	-2.523899	-2.305762	-3.107539
28	1	0	-2.299424	-2.856415	-1.425170
29	8	0	-1.814905	-0.873701	-1.853967
30	6	0	-1.550237	2.619818	-0.657636
31	1	0	-2.611247	2.623042	-0.926141
32	1	0	-1.447051	3.085481	0.336808
33	1	0	-1.016962	3.245854	-1.385749

----

17 isomer A (cis, chair), PBE0/6-31+G(d):
Sum of electronic and thermal Free Energies= -919.896464

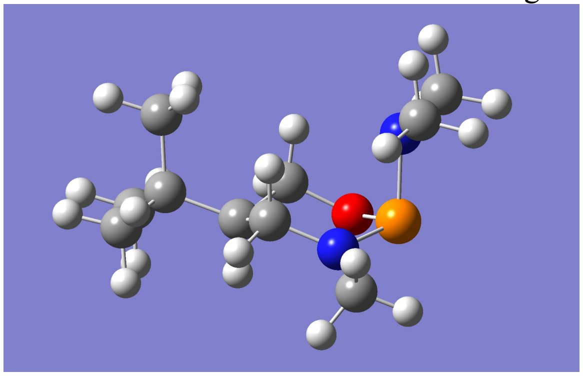

Center Number	Atomic Number	Atomic Type	Coordinates (Angstroms) X	Y	Z
1	6	0	0.466059	-1.159331	-0.722646
2	6	0	0.403933	1.117730	0.339451
3	6	0	1.267738	0.112317	-0.439129
4	1	0	0.163807	-1.641218	0.214419
5	1	0	1.045490	-1.870776	-1.316264
6	1	0	0.941597	2.068647	0.423586

7	1	0	0.217423	0.758621	1.365201
8	1	0	1.476999	0.578838	-1.414388
9	6	0	2.645531	-0.181449	0.226550
10	6	0	2.514095	-1.034573	1.496072
11	1	0	3.497469	-1.160077	1.966301
12	1	0	1.852232	-0.571852	2.237187
13	1	0	2.126960	-2.036382	1.278545
14	6	0	3.547759	-0.921604	-0.772145
15	1	0	3.670249	-0.345973	-1.698186
16	1	0	4.544473	-1.074960	-0.340364
17	1	0	3.157287	-1.909439	-1.039229
18	6	0	3.344947	1.135507	0.590213
19	1	0	4.375985	0.938244	0.908211
20	1	0	3.387252	1.816993	-0.269455
21	1	0	2.847500	1.657390	1.415347
22	15	0	-1.840558	0.143706	-0.901028
23	8	0	-0.702378	-0.892995	-1.505614
24	7	0	-0.845734	1.399595	-0.353589
25	6	0	-1.331225	2.759259	-0.309660
26	1	0	-0.588735	3.450774	-0.732560
27	1	0	-2.246713	2.840540	-0.905384
28	1	0	-1.563662	3.095297	0.714482
29	7	0	-2.151798	-0.719390	0.606203
30	6	0	-2.823936	-1.992217	0.401740
31	1	0	-2.815483	-2.564919	1.338157
32	1	0	-3.876920	-1.880400	0.082889
33	1	0	-2.296845	-2.577004	-0.358167
34	6	0	-2.819675	0.081942	1.613471
35	1	0	-2.850168	-0.475466	2.558557
36	1	0	-2.264707	1.008708	1.790425
37	1	0	-3.860842	0.346350	1.347675

----

17 isomer B (cis, twist 1), PBE0/6-31+G(d):
Sum of electronic and thermal Free Energies= -919.896422

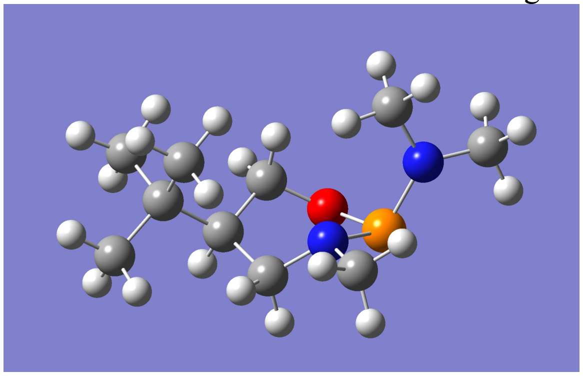

Center Number	Atomic Number	Atomic Type	Coordinates (Angstroms) X	Y	Z
1	6	0	0.091016	-1.412510	-0.130131
2	6	0	0.130859	1.046024	0.333434
3	6	0	0.897277	-0.123248	-0.319782
4	1	0	0.282459	-1.859315	0.852777
5	1	0	0.349580	-2.153548	-0.891326
6	1	0	-0.441500	1.595984	-0.435529
7	1	0	0.834193	1.773451	0.754400
8	1	0	0.926688	0.075990	-1.401202
9	6	0	2.377923	-0.259076	0.136185
10	6	0	2.513935	-0.335513	1.661419
11	1	0	3.571415	-0.430556	1.938857
12	1	0	2.123130	0.561737	2.154283
13	1	0	1.985301	-1.199318	2.079107
14	6	0	2.993964	-1.522702	-0.481303
15	1	0	2.869693	-1.534514	-1.571876
16	1	0	4.069461	-1.564474	-0.269417
17	1	0	2.547887	-2.439767	-0.079478
18	6	0	3.177267	0.946367	-0.375781
19	1	0	4.230707	0.860006	-0.081739
20	1	0	3.139795	1.008876	-1.470715
21	1	0	2.805547	1.896045	0.026324
22	15	0	-2.133625	-0.273157	0.852687
23	8	0	-1.302057	-1.162264	-0.289599
24	7	0	-0.736040	0.603530	1.425942
25	6	0	-1.064066	1.670556	2.356261
26	1	0	-0.144620	2.037502	2.830081
27	1	0	-1.568554	2.528028	1.876787
28	1	0	-1.719793	1.286048	3.143729
29	7	0	-2.406178	-1.214642	2.241283
30	6	0	-1.390558	-1.825072	3.074039
31	1	0	-1.709083	-1.803258	4.125998
32	1	0	-1.211218	-2.877336	2.799460
33	1	0	-0.458307	-1.262268	2.983278
34	6	0	-3.718532	-1.813206	2.382054
35	1	0	-4.063089	-1.733262	3.422554
36	1	0	-4.435742	-1.291009	1.740437
37	1	0	-3.718779	-2.878892	2.103571

17 isomer C (cis, twist 2), PBE0/6-31+G(d):
Sum of electronic and thermal Free Energies= -919.894191

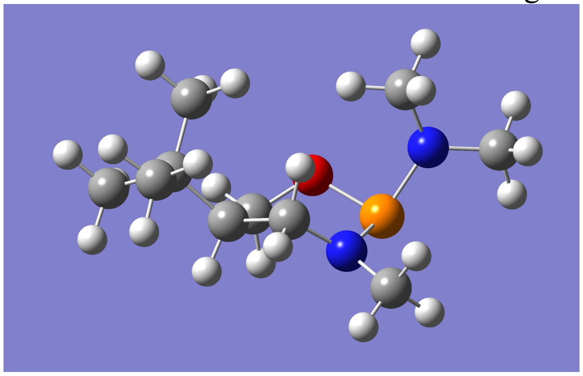

Center Number	Atomic Number	Atomic Type	Coordinates (Angstroms) X	Y	Z
1	6	0	0.683105	-1.337774	-1.020426
2	6	0	0.574533	1.129202	-0.606955
3	6	0	1.521731	-0.052517	-0.894893
4	1	0	1.231907	-2.219137	-0.676588
5	1	0	0.415446	-1.512630	-2.071707
6	1	0	1.029091	2.057495	-0.971881
7	1	0	0.435094	1.262042	0.477579
8	1	0	1.964325	0.131734	-1.885886
9	6	0	2.711009	-0.153156	0.099993
10	6	0	2.265446	-0.620103	1.490557
11	1	0	3.134088	-0.700729	2.156225
12	1	0	1.563632	0.082854	1.953120
13	1	0	1.778467	-1.601035	1.457931
14	6	0	3.746582	-1.143604	-0.449749
15	1	0	4.113736	-0.825447	-1.433911
16	1	0	4.610213	-1.208106	0.223727
17	1	0	3.342011	-2.156940	-0.554651
18	6	0	3.395885	1.214347	0.226479
19	1	0	4.302898	1.131533	0.837944
20	1	0	3.692891	1.603166	-0.756406
21	1	0	2.748091	1.958234	0.704085
22	15	0	-1.737299	-0.355979	-0.868320
23	8	0	-0.499445	-1.286189	-0.227899
24	7	0	-0.693439	0.953197	-1.298210
25	6	0	-1.332199	2.148065	-1.799010
26	1	0	-2.231970	1.871308	-2.362354
27	1	0	-1.637366	2.846454	-1.000546
28	1	0	-0.661457	2.684325	-2.483219
29	7	0	-2.539823	-0.058394	0.600068
30	6	0	-1.914102	0.280474	1.859150

31	1	0	-0.873615	-0.049461	1.854796
32	1	0	-1.954644	1.362320	2.065396
33	1	0	-2.425688	-0.238711	2.682226
34	6	0	-3.978503	0.086420	0.578141
35	1	0	-4.443760	-0.553300	1.342489
36	1	0	-4.291923	1.125229	0.768957
37	1	0	-4.366815	-0.213394	-0.400871

----

18 isomer A (trans, chair), PBE0/6-31+G(d):
Sum of electronic and thermal Free Energies= -919.899774

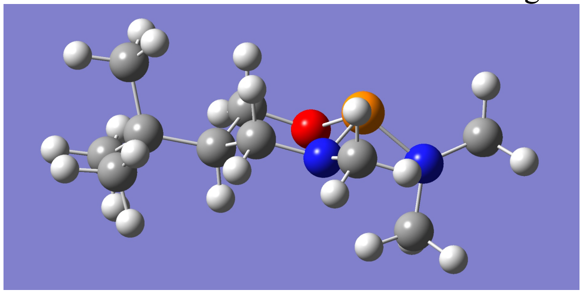

Center Number	Atomic Number	Atomic Type	Coordinates (Angstroms) X	Y	Z
1	6	0	-0.705808	-1.304833	-0.479962
2	6	0	-0.686336	1.160675	-0.135313
3	6	0	-1.369189	-0.149590	0.270256
4	1	0	-0.832928	-1.180972	-1.568316
5	1	0	-1.129450	-2.273421	-0.201511
6	1	0	-1.092752	1.989519	0.455748
7	1	0	-0.909331	1.392383	-1.196625
8	1	0	-1.146296	-0.301179	1.337109
9	6	0	-2.920280	-0.127064	0.131135
10	6	0	-3.383985	-0.111819	-1.332093
11	1	0	-4.477921	-0.039716	-1.377169
12	1	0	-2.976949	0.742610	-1.884946
13	1	0	-3.097259	-1.025768	-1.864715
14	6	0	-3.506968	-1.368822	0.819157
15	1	0	-3.202200	-1.419810	1.872026
16	1	0	-4.603061	-1.335638	0.790151
17	1	0	-3.199612	-2.301620	0.334272
18	6	0	-3.493846	1.107326	0.840991
19	1	0	-4.587356	1.037857	0.892398
20	1	0	-3.116626	1.188830	1.868658
21	1	0	-3.254077	2.038708	0.316102
22	15	0	1.603972	-0.114866	-0.763656
23	8	0	0.677902	-1.378590	-0.163441
24	7	0	0.757037	1.146193	0.109631

25	6	0	1.337910	2.463047	-0.098696
26	1	0	0.882277	3.172588	0.603373
27	1	0	2.412901	2.430045	0.102655
28	1	0	1.187535	2.852099	-1.122322
29	7	0	2.991552	-0.222126	0.181673
30	6	0	4.207574	-0.710713	-0.435785
31	1	0	4.145334	-0.597230	-1.522613
32	1	0	5.071763	-0.134728	-0.077483
33	1	0	4.386485	-1.773238	-0.207643
34	6	0	2.955161	-0.345600	1.626086
35	1	0	2.016774	0.068541	2.001486
36	1	0	3.035099	-1.396362	1.942602
37	1	0	3.790897	0.213624	2.068743

----

18 isomer B (trans, twist 1), PBE0/6-31+G(d):
Sum of electronic and thermal Free Energies= -919.893379

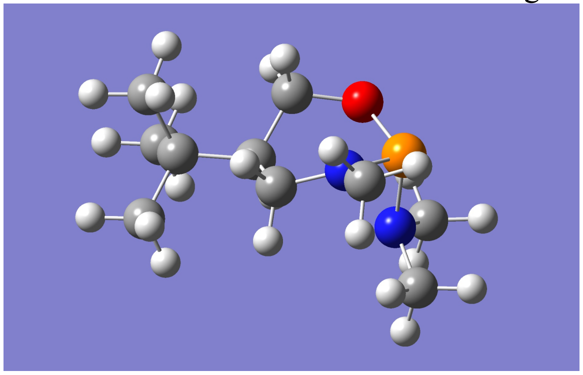

Center Number	Atomic Number	Atomic Type	Coordinates (Angstroms) X	Y	Z
1	6	0	0.752459	-1.600215	1.029190
2	6	0	0.529197	0.879009	0.906089
3	6	0	1.077301	-0.365735	0.188269
4	1	0	1.096112	-1.461304	2.063732
5	1	0	1.232182	-2.498084	0.630344
6	1	0	0.372236	1.698087	0.185782
7	1	0	1.265675	1.239064	1.642350
8	1	0	0.524036	-0.472341	-0.753301
9	6	0	2.585004	-0.246566	-0.177200
10	6	0	3.495361	-0.398301	1.048429
11	1	0	4.542668	-0.245015	0.760183
12	1	0	3.262340	0.332908	1.831713
13	1	0	3.422046	-1.398810	1.490383
14	6	0	2.948932	-1.332785	-1.199177
15	1	0	2.337501	-1.241754	-2.105384

16	1	0	4.001163	-1.239705	-1.495369
17	1	0	2.812435	-2.344781	-0.802130
18	6	0	2.853374	1.114745	-0.832892
19	1	0	3.887757	1.162029	-1.195159
20	1	0	2.190729	1.279040	-1.692043
21	1	0	2.715158	1.947556	-0.134003
22	15	0	-1.737820	-0.667090	1.221511
23	8	0	-0.643449	-1.905879	1.055950
24	7	0	-0.696736	0.599169	1.639289
25	6	0	-2.574233	-1.472446	-1.210405
26	1	0	-3.620512	-1.677257	-0.915000
27	1	0	-2.565890	-1.263999	-2.287950
28	1	0	-1.981071	-2.373525	-1.032822
29	6	0	-1.116220	1.623981	2.566211
30	1	0	-0.337774	1.803970	3.322047
31	1	0	-1.324410	2.581716	2.061225
32	1	0	-2.027725	1.306009	3.082000
33	7	0	-1.988971	-0.346585	-0.498493
34	6	0	-2.753770	0.869799	-0.713747
35	1	0	-3.800210	0.802672	-0.360024
36	1	0	-2.273630	1.714319	-0.207628
37	1	0	-2.779733	1.095084	-1.787303

----

18 isomer C (trans, twist 2), PBE0/6-31+G(d):
Sum of electronic and thermal Free Energies= -919.896586

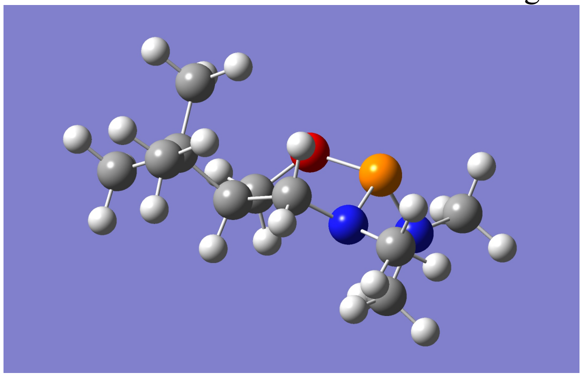

Center Number	Atomic Number	Atomic Type	Coordinates (Angstroms) X	Y	Z
1	6	0	0.785481	-1.427063	-0.766358
2	6	0	0.666933	1.068194	-0.801620
3	6	0	1.607686	-0.139761	-0.939770
4	1	0	1.388812	-2.248510	-0.368801
5	1	0	0.390631	-1.751563	-1.735582
6	1	0	1.064274	1.908869	-1.381038

7	1	0	0.621482	1.412928	0.247837
8	1	0	1.973323	-0.142797	-1.978751
9	6	0	2.866583	-0.046861	-0.033743
10	6	0	2.531544	-0.268964	1.446095
11	1	0	3.443901	-0.194096	2.051240
12	1	0	1.821278	0.473948	1.824600
13	1	0	2.093289	-1.257302	1.621700
14	6	0	3.886720	-1.104467	-0.476387
15	1	0	4.178884	-0.959454	-1.524544
16	1	0	4.795046	-1.038769	0.135171
17	1	0	3.501141	-2.125378	-0.373670
18	6	0	3.522817	1.330498	-0.200712
19	1	0	4.472754	1.367245	0.346596
20	1	0	3.739243	1.543973	-1.255870
21	1	0	2.893138	2.139028	0.187444
22	15	0	-1.573179	-0.260949	-0.237904
23	8	0	-0.287055	-1.250055	0.155347
24	7	0	-0.667559	0.767191	-1.321608
25	6	0	-3.763242	-1.664477	-0.914926
26	1	0	-4.014046	-1.289872	0.082868
27	1	0	-4.606936	-1.452034	-1.586454
28	1	0	-3.644211	-2.757715	-0.852874
29	6	0	-1.400761	1.953080	-1.733741
30	1	0	-2.388997	1.664550	-2.105798
31	1	0	-1.540872	2.685049	-0.918784
32	1	0	-0.863101	2.449512	-2.550889
33	7	0	-2.561807	-1.013271	-1.397586
34	6	0	-2.166741	-1.423945	-2.729235
35	1	0	-1.900923	-2.492769	-2.767951
36	1	0	-2.997306	-1.260117	-3.430624
37	1	0	-1.313314	-0.825391	-3.056987

19, PBE0/6-31+G(d):
Sum of electronic and thermal Free Energies= -2124.964576

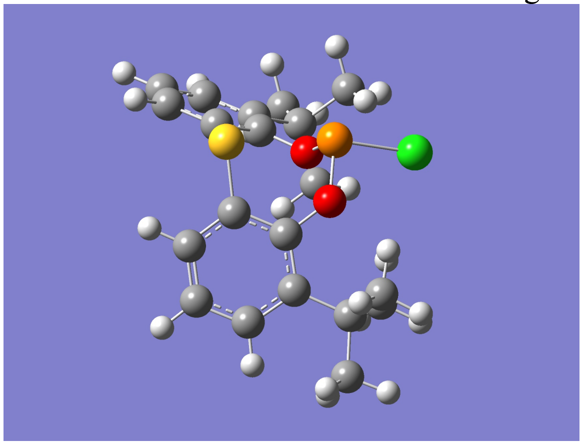

Center Number	Atomic Number	Atomic Type	Coordinates (Angstroms) X	Y	Z
1	15	0	0.012562	-0.741133	-1.755224
2	8	0	-0.932363	-0.532014	-0.381615
3	8	0	1.514321	-0.410527	-1.107394
4	16	0	-0.095061	2.039174	-1.625741
5	6	0	-1.865838	0.459847	-0.228958
6	6	0	-1.596373	1.747437	-0.714825
7	6	0	-2.514196	2.780118	-0.539757
8	1	0	-2.291915	3.768285	-0.932285
9	6	0	-3.696410	2.521562	0.139077
10	6	0	-3.940574	1.246225	0.642182
11	1	0	-4.867772	1.079715	1.178688
12	6	0	-3.048439	0.178158	0.483211
13	6	0	1.850515	0.636129	-0.291714
14	6	0	1.162294	1.855168	-0.381540
15	6	0	1.512467	2.932828	0.431553
16	1	0	0.969040	3.869048	0.342224
17	6	0	2.556311	2.788692	1.329871
18	6	0	3.248127	1.579917	1.395082
19	1	0	4.069707	1.503236	2.098140
20	6	0	2.931407	0.472038	0.603089
21	6	0	-3.350255	-1.210929	1.061048
22	6	0	-4.690496	-1.234639	1.805702
23	1	0	-4.707704	-0.541228	2.655080
24	1	0	-4.854794	-2.241973	2.204061
25	1	0	-5.536188	-1.003079	1.147058
26	6	0	-3.436485	-2.249873	-0.070215
27	1	0	-4.210791	-1.971840	-0.795305
28	1	0	-3.703438	-3.228072	0.348601
29	1	0	-2.488378	-2.366263	-0.597991
30	6	0	-2.257547	-1.613181	2.068048
31	1	0	-1.272509	-1.676838	1.601247
32	1	0	-2.494649	-2.595853	2.494181
33	1	0	-2.205106	-0.891893	2.892537
34	6	0	3.717382	-0.841195	0.719474
35	6	0	4.878570	-0.718727	1.714388
36	1	0	5.601742	0.050122	1.416609
37	1	0	5.413202	-1.674474	1.751217
38	1	0	4.534598	-0.500826	2.732517
39	6	0	4.325067	-1.229598	-0.640319
40	1	0	4.993403	-0.440782	-1.006416
41	1	0	3.560605	-1.413942	-1.396961
42	1	0	4.916856	-2.146254	-0.527728

43	6	0	2.790984	-1.958615	1.228449
44	1	0	2.378292	-1.701031	2.211340
45	1	0	3.360614	-2.890304	1.335141
46	1	0	1.962980	-2.151448	0.544500
47	17	0	0.209057	-2.850812	-1.492098
48	1	0	-4.427321	3.311900	0.285966
49	1	0	2.846853	3.615514	1.971990

20, PBE0/6-31+G(d):
Sum of electronic and thermal Free Energies= -3747.609220

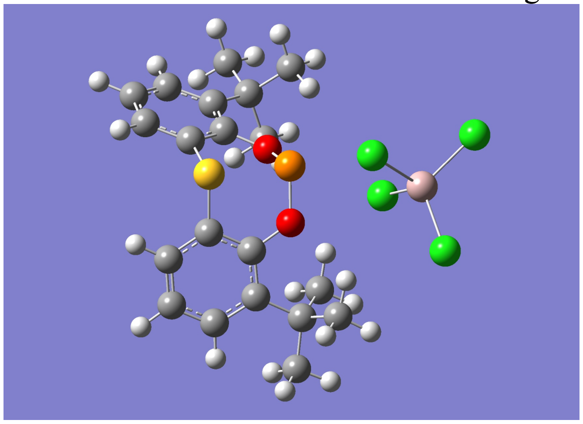

Center Number	Atomic Number	Atomic Type	Coordinates (Angstroms) X	Y	Z
1	15	0	-0.637137	-0.045444	-1.714866
2	8	0	-0.993357	-1.077656	-0.466966
3	8	0	-0.562279	1.403378	-0.954344
4	16	0	-3.059363	0.323473	-1.876353
5	6	0	-2.201924	-1.703093	-0.298759
6	6	0	-3.329423	-1.135825	-0.901635
7	6	0	-4.592522	-1.705322	-0.774524
8	1	0	-5.451794	-1.258801	-1.266086
9	6	0	-4.709380	-2.857752	-0.011156
10	6	0	-3.582078	-3.409560	0.596301
11	1	0	-3.712756	-4.311242	1.183810
12	6	0	-2.294669	-2.867867	0.481279
13	6	0	-1.623345	2.030433	-0.337054
14	6	0	-2.919494	1.631067	-0.680615
15	6	0	-4.041007	2.270161	-0.158383
16	1	0	-5.039722	1.955574	-0.446097
17	6	0	-3.838939	3.310275	0.734793
18	6	0	-2.542869	3.698200	1.074226
19	1	0	-2.424875	4.519310	1.772024
20	6	0	-1.392355	3.087824	0.560588

21	6	0	-1.079713	-3.495536	1.170740
22	6	0	-1.464752	-4.763897	1.940216
23	1	0	-2.187427	-4.562749	2.740316
24	1	0	-0.565343	-5.172718	2.412272
25	1	0	-1.871853	-5.543289	1.284526
26	6	0	-0.020056	-3.887618	0.125605
27	1	0	-0.430005	-4.603343	-0.597451
28	1	0	0.828618	-4.364826	0.628604
29	1	0	0.370005	-3.024846	-0.417734
30	6	0	-0.491058	-2.494897	2.180097
31	1	0	-0.137131	-1.579174	1.702600
32	1	0	0.370209	-2.945270	2.685541
33	1	0	-1.234290	-2.231171	2.942304
34	6	0	0.015264	3.535303	0.963394
35	6	0	-0.028327	4.711283	1.945662
36	1	0	-0.507211	5.599164	1.514746
37	1	0	0.999229	4.988290	2.203239
38	1	0	-0.538648	4.453639	2.881532
39	6	0	0.802609	3.994383	-0.276751
40	1	0	0.293184	4.828416	-0.775079
41	1	0	0.949143	3.189986	-0.999779
42	1	0	1.797243	4.334743	0.030318
43	6	0	0.736268	2.370361	1.660355
44	1	0	0.200111	2.064908	2.566707
45	1	0	1.747595	2.675352	1.947982
46	1	0	0.839363	1.497207	1.013926
47	17	0	1.867005	-0.528044	-1.451228
48	1	0	-5.677286	-3.335245	0.109618
49	1	0	-4.689275	3.829575	1.167029
50	13	0	3.583478	-0.207547	0.019457
51	17	0	5.070718	-1.542513	-0.694827
52	17	0	4.099718	1.852821	-0.148158
53	17	0	2.831450	-0.720584	1.949136

21, PBE0/6-31+G(d):
Sum of electronic and thermal Free Energies= -3588.861130

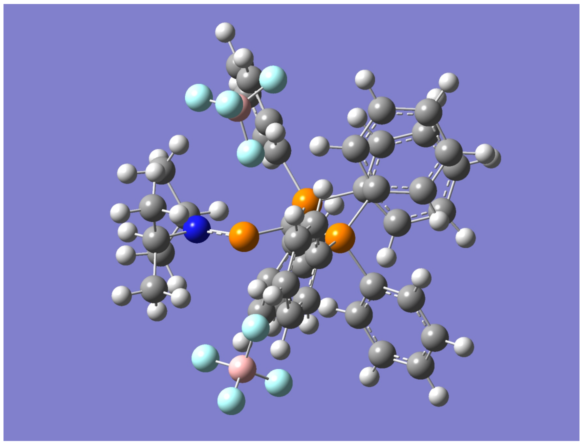

Center Number	Atomic Number	Atomic Type	Coordinates (Angstroms) X	Y	Z
1	6	0	8.646323	10.520995	7.635482
2	6	0	10.651639	6.862093	6.966566
3	1	0	11.328874	6.343738	7.649401
4	6	0	11.444647	7.224355	5.716511
5	1	0	11.861383	6.307756	5.283024
6	1	0	12.265205	7.913080	5.936157
7	1	0	10.812424	7.695455	4.956362
8	6	0	9.476309	5.934943	6.678859
9	1	0	8.780803	6.379443	5.958462
10	1	0	8.910495	5.699696	7.584324
11	1	0	9.854561	5.001357	6.246568
12	6	0	10.765620	8.166852	9.117950
13	1	0	10.335594	9.066780	9.559579
14	6	0	10.327595	6.977129	9.971698
15	1	0	9.251995	6.795225	9.905484
16	1	0	10.583724	7.181889	11.017530
17	1	0	10.847996	6.056683	9.685141
18	6	0	12.283036	8.333048	9.101496
19	1	0	12.785746	7.419151	8.766380
20	1	0	12.632256	8.544910	10.118082
21	1	0	12.593227	9.151175	8.447024
22	6	0	8.093283	13.013770	6.094267
23	6	0	7.259698	14.132207	6.209073
24	1	0	6.251668	14.036600	6.598298
25	6	0	7.737071	15.390129	5.852275
26	1	0	7.083983	16.254166	5.945039
27	6	0	9.043788	15.544388	5.391275
28	1	0	9.408076	16.529563	5.110423
29	6	0	9.883669	14.436645	5.300108
30	1	0	10.909884	14.529726	4.955274
31	6	0	9.413136	13.174476	5.651482

32	1	0	10.086318	12.326082	5.567345
33	6	0	7.449500	10.392145	4.903734
34	6	0	6.551686	9.315428	4.828865
35	1	0	5.956535	9.024865	5.689519
36	6	0	6.454692	8.580487	3.653576
37	1	0	5.769619	7.738375	3.609406
38	6	0	7.239584	8.914944	2.550310
39	1	0	7.163087	8.335449	1.633764
40	6	0	8.126314	9.986110	2.623512
41	1	0	8.749557	10.245612	1.772489
42	6	0	8.235883	10.727274	3.796728
43	1	0	8.944033	11.545943	3.836509
44	6	0	5.800371	11.349874	7.011994
45	6	0	4.842691	11.996199	6.213223
46	1	0	5.139847	12.524312	5.310807
47	6	0	3.497092	11.926393	6.552827
48	1	0	2.760146	12.429678	5.932466
49	6	0	3.092349	11.186261	7.665960
50	1	0	2.036541	11.115766	7.914601
51	6	0	4.033970	10.503677	8.429145
52	1	0	3.731107	9.866276	9.254217
53	6	0	5.387226	10.586366	8.104132
54	1	0	6.102730	10.002310	8.670380
55	6	0	8.335228	13.020881	9.308497
56	6	0	7.001807	13.219792	9.690094
57	1	0	6.346802	12.372693	9.868964
58	6	0	6.512775	14.511318	9.861046
59	1	0	5.478152	14.656434	10.160482
60	6	0	7.350198	15.608035	9.664758
61	1	0	6.968962	16.615644	9.810347
62	6	0	8.677549	15.412789	9.288705
63	1	0	9.334952	16.263872	9.134122
64	6	0	9.172844	14.124509	9.110747
65	1	0	10.209818	13.981706	8.822516
66	6	0	10.795239	11.513203	9.422717
67	6	0	11.607006	11.714337	8.303229
68	1	0	11.206877	11.618940	7.300253
69	6	0	12.960738	12.006012	8.466292
70	1	0	13.571206	12.142124	7.578961
71	6	0	13.503533	12.086398	9.745104
72	1	0	14.560058	12.307624	9.873277
73	6	0	12.699385	11.861931	10.864341
74	1	0	13.127100	11.903497	11.862818
75	6	0	11.346896	11.577461	10.710256
76	1	0	10.735095	11.392920	11.588001
77	6	0	8.276714	10.474144	10.617275

78	6	0	7.767016	9.180645	10.491116
79	1	0	7.767240	8.670523	9.532463
80	6	0	7.207418	8.538358	11.595099
81	1	0	6.795224	7.542979	11.460537
82	6	0	7.164837	9.184217	12.826164
83	1	0	6.726033	8.684649	13.686086
84	6	0	7.664399	10.482192	12.956324
85	1	0	7.614643	10.995342	13.913250
86	6	0	8.211564	11.132970	11.857090
87	1	0	8.561187	12.157357	11.959967
88	7	0	10.222481	8.086372	7.747369
89	15	0	9.213568	9.040326	6.880645
90	15	0	7.532247	11.324437	6.468519
91	15	0	9.004194	11.326492	9.186706
92	5	0	12.411223	10.937218	4.935083
93	5	0	6.073193	6.853820	8.463887
94	9	0	13.327413	10.238774	5.727963
95	9	0	12.599037	10.702573	3.587022
96	9	0	12.487540	12.319438	5.227527
97	9	0	11.083062	10.513061	5.309493
98	9	0	6.823224	7.823299	7.705127
99	9	0	5.132947	7.558027	9.235178
100	9	0	5.463982	5.968935	7.595971
101	9	0	6.982368	6.200987	9.319841

22, PBE0/6-31+G(d):
Sum of electronic and thermal Free Energies= -2247.734866

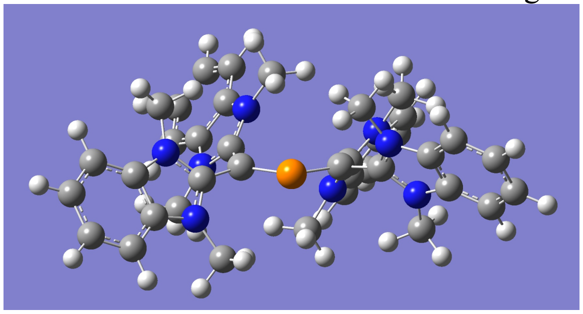

Center Number	Atomic Number	Atomic Type	Coordinates (Angstroms) X	Y	Z
1	6	0	3.175211	6.650784	11.608353
2	6	0	2.774793	7.082430	10.280816
3	6	0	2.298635	7.101312	8.091537
4	6	0	2.065473	6.784467	6.753920
5	1	0	2.100156	5.764934	6.382218
6	6	0	1.791675	7.849309	5.906167

7	1	0	1.609580	7.655501	4.853394
8	6	0	1.752715	9.176969	6.373525
9	1	0	1.541669	9.977554	5.670944
10	6	0	1.988314	9.491268	7.705137
11	1	0	1.972381	10.521012	8.048660
12	6	0	2.263162	8.420482	8.555586
13	6	0	2.977911	4.902444	9.051580
14	1	0	3.473003	4.567583	9.963419
15	1	0	3.662197	4.783096	8.206353
16	1	0	2.085466	4.297783	8.872212
17	6	0	2.582205	9.537949	10.777768
18	1	0	2.364733	9.244758	11.805215
19	1	0	1.817946	10.243567	10.444607
20	1	0	3.559855	10.027632	10.733856
21	6	0	4.298928	7.384390	12.201536
22	6	0	6.311082	8.344494	12.423319
23	6	0	7.604053	8.839208	12.251807
24	1	0	8.156571	8.723832	11.324617
25	6	0	8.159369	9.501179	13.337099
26	1	0	9.164472	9.903227	13.253229
27	6	0	7.455734	9.665784	14.546411
28	1	0	7.935050	10.188675	15.368421
29	6	0	6.169377	9.175429	14.715371
30	1	0	5.640847	9.299846	15.655249
31	6	0	5.610504	8.512009	13.622324
32	6	0	5.879040	7.143573	10.267147
33	1	0	5.380219	6.196306	10.059605
34	1	0	6.955972	6.965822	10.279929
35	1	0	5.648260	7.872176	9.483941
36	6	0	3.317973	8.001656	14.449226
37	1	0	2.343023	7.866705	13.979785
38	1	0	3.354311	8.991750	14.910171
39	1	0	3.460594	7.237453	15.217553
40	6	0	1.191940	4.510172	12.128285
41	6	0	0.189606	4.818502	11.123525
42	6	0	-1.374521	5.878734	9.919454
43	6	0	-2.274321	6.793882	9.374947
44	1	0	-2.334546	7.823711	9.713521
45	6	0	-3.107436	6.317738	8.371501
46	1	0	-3.830790	6.989820	7.919692
47	6	0	-3.045953	4.982368	7.929210
48	1	0	-3.723092	4.654292	7.146296
49	6	0	-2.151756	4.071247	8.474844
50	1	0	-2.126729	3.039798	8.136861
51	6	0	-1.315604	4.550959	9.482983
52	6	0	-0.357140	7.176136	11.791108

53	1	0	-1.366117	7.461280	12.103531
54	1	0	0.096271	8.012205	11.252864
55	1	0	0.238800	6.940394	12.673015
56	6	0	0.061065	2.539678	10.071071
57	1	0	1.089158	2.401143	10.406536
58	1	0	-0.000604	2.283205	9.011105
59	1	0	-0.602741	1.877037	10.634788
60	6	0	0.923044	3.324962	12.949381
61	6	0	1.138648	1.392551	14.058195
62	6	0	1.586113	0.201658	14.631279
63	1	0	2.595308	-0.171440	14.488577
64	6	0	0.670982	-0.487289	15.413301
65	1	0	0.971222	-1.419048	15.882871
66	6	0	-0.638433	-0.009198	15.617419
67	1	0	-1.318890	-0.584726	16.237645
68	6	0	-1.081689	1.176133	15.049140
69	1	0	-2.094912	1.529825	15.211660
70	6	0	-0.159704	1.869861	14.265098
71	6	0	3.110377	2.080075	12.702162
72	1	0	3.256913	2.670883	11.797544
73	1	0	3.211932	1.020501	12.454340
74	1	0	3.867186	2.354814	13.441427
75	6	0	-1.406748	3.951528	13.641364
76	1	0	-1.081267	4.990335	13.575867
77	1	0	-1.898117	3.809288	14.605644
78	1	0	-2.120309	3.727065	12.842582
79	7	0	2.626100	6.303111	9.181352
80	7	0	2.549714	8.368396	9.914324
81	7	0	5.475478	7.629731	11.575546
82	7	0	4.364704	7.923957	13.443013
83	7	0	-0.437644	6.005960	10.938279
84	7	0	-0.329669	3.930057	10.240538
85	7	0	1.772801	2.305802	13.225446
86	7	0	-0.250790	3.073331	13.578364
87	15	0	2.639671	5.341839	12.624752

23, PBE0/6-31+G(d):

Sum of electronic and thermal Free Energies= -1265.762490

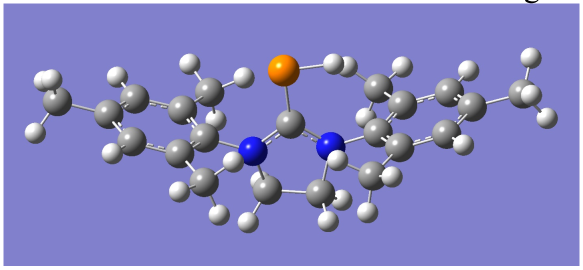

Center Number	Atomic Number	Atomic Type	Coordinates (Angstroms) X	Y	Z
1	15	0	0.136823	-0.016797	-1.817403
2	7	0	1.097380	-0.056293	0.751999
3	6	0	0.006725	-0.005493	-0.076057
4	6	0	0.735708	0.168515	2.143487
5	1	0	0.953558	1.203111	2.448789
6	1	0	1.290554	-0.506309	2.803739
7	6	0	2.448348	-0.016323	0.305778
8	6	0	3.126145	-1.232025	0.117187
9	6	0	4.459475	-1.193474	-0.285375
10	1	0	4.990729	-2.132095	-0.438048
11	6	0	5.126564	0.014636	-0.505763
12	6	0	4.422343	1.202903	-0.317325
13	1	0	4.922244	2.153265	-0.498584
14	6	0	3.084328	1.213272	0.085087
15	6	0	2.417003	-2.539821	0.323541
16	1	0	2.024202	-2.633544	1.343346
17	1	0	3.092795	-3.381726	0.144061
18	1	0	1.566270	-2.628852	-0.362539
19	6	0	6.563669	0.023576	-0.949662
20	1	0	6.948870	1.044259	-1.037887
21	1	0	6.678790	-0.462712	-1.926017
22	1	0	7.202921	-0.517069	-0.241401
23	6	0	2.349055	2.512589	0.257697
24	1	0	1.359960	2.469219	-0.210773
25	1	0	2.906255	3.334492	-0.202260
26	1	0	2.210354	2.765753	1.317091
27	1	0	-1.277188	-0.021367	-1.997217
28	7	0	-1.103549	0.061076	0.724743
29	6	0	-0.765999	-0.113072	2.132532
30	1	0	-0.991122	-1.136254	2.468861
31	1	0	-1.334341	0.584316	2.756903
32	6	0	-2.453490	0.018561	0.280471
33	6	0	-3.131053	1.231178	0.080858
34	6	0	-4.467847	1.189018	-0.314040
35	1	0	-4.998302	2.126043	-0.477879
36	6	0	-5.137788	-0.020368	-0.512534
37	6	0	-4.430883	-1.207792	-0.319351
38	1	0	-4.931188	-2.159927	-0.490654
39	6	0	-3.091461	-1.213674	0.075620
40	6	0	-2.417504	2.540304	0.263435
41	1	0	-2.033245	2.654454	1.284497
42	1	0	-3.087819	3.381282	0.060718

43	1	0	-1.558527	2.613398	-0.414317
44	6	0	-6.587932	-0.039112	-0.911080
45	1	0	-6.859050	-0.984413	-1.392175
46	1	0	-6.822786	0.774602	-1.605710
47	1	0	-7.238247	0.082489	-0.035048
48	6	0	-2.348662	-2.508472	0.249180
49	1	0	-1.374962	-2.474875	-0.252110
50	1	0	-2.920301	-3.341427	-0.171491
51	1	0	-2.170054	-2.737194	1.307857

24, PBE0/6-31+G(d):
Sum of electronic and thermal Free Energies= -2283.284055

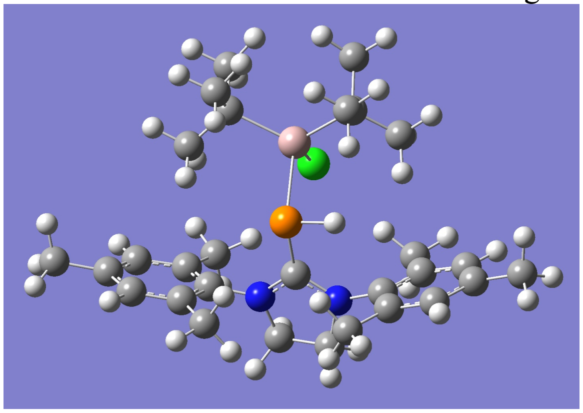

Center Number	Atomic Number	Atomic Type	Coordinates (Angstroms) X	Y	Z
1	15	0	-0.032178	0.052545	-1.080573
2	17	0	0.203559	1.898038	2.228399
3	13	0	0.237332	2.310243	0.055591
4	7	0	0.934133	-1.945067	0.607252
5	7	0	-1.264081	-1.883759	0.558371
6	6	0	2.303353	-1.800261	0.226489
7	6	0	2.724053	-2.304076	-1.018231
8	6	0	-0.135866	-1.296586	0.101524
9	6	0	3.220784	-1.270542	1.147693
10	6	0	4.564420	-1.208907	0.775848
11	1	0	5.278814	-0.783600	1.478792
12	6	0	5.011298	-1.651122	-0.468366
13	6	0	1.770691	-2.954548	-1.978990
14	1	0	1.078852	-3.634700	-1.468623
15	1	0	2.318236	-3.531347	-2.730347
16	1	0	1.161973	-2.205551	-2.499640
17	6	0	4.076209	-2.202542	-1.345202
18	1	0	4.408903	-2.579497	-2.311045

19	6	0	-2.616836	-1.586396	0.210814
20	6	0	2.050680	3.016896	-0.475136
21	6	0	0.533736	-2.967229	1.578078
22	1	0	0.758975	-2.630069	2.596323
23	1	0	1.079758	-3.895854	1.388588
24	6	0	-3.414190	-0.895036	1.136083
25	6	0	-1.449708	3.283428	-0.504395
26	6	0	2.797716	-0.780860	2.503221
27	1	0	1.827998	-0.275028	2.476370
28	1	0	3.528222	-0.067939	2.896820
29	1	0	2.737004	-1.607399	3.224536
30	6	0	3.137123	1.970884	-0.223359
31	1	0	3.180942	1.677628	0.832936
32	1	0	4.131989	2.371626	-0.491015
33	1	0	2.987944	1.059845	-0.817362
34	6	0	-4.752184	-0.671424	0.808336
35	1	0	-5.377002	-0.125728	1.513274
36	6	0	6.455592	-1.519130	-0.862604
37	1	0	6.641462	-0.543114	-1.329006
38	1	0	6.746771	-2.288451	-1.585339
39	1	0	7.117901	-1.597401	0.005752
40	6	0	-0.968157	-3.090157	1.333465
41	1	0	-1.233266	-3.982870	0.750167
42	1	0	-1.550510	-3.101814	2.258374
43	6	0	-3.141898	-2.068627	-0.999229
44	6	0	-5.304374	-1.113503	-0.394497
45	6	0	2.077539	3.398346	-1.959228
46	1	0	1.845791	2.545624	-2.612746
47	1	0	3.080110	3.761286	-2.248711
48	1	0	1.367839	4.201673	-2.196529
49	6	0	-2.852872	-0.399159	2.437296
50	1	0	-2.595793	-1.227171	3.111097
51	1	0	-3.584484	0.226232	2.956759
52	1	0	-1.946095	0.197130	2.290767
53	6	0	-2.677964	2.757211	0.241290
54	1	0	-2.896926	1.709718	-0.003011
55	1	0	-3.574407	3.344035	-0.029496
56	1	0	-2.557403	2.825247	1.329673
57	6	0	-4.484629	-1.814741	-1.280390
58	1	0	-4.900831	-2.178802	-2.218361
59	6	0	2.383593	4.257310	0.365019
60	1	0	1.678196	5.081258	0.199808
61	1	0	3.388329	4.637843	0.107437
62	1	0	2.384734	4.034586	1.439723
63	6	0	-1.715830	3.195061	-2.010964
64	1	0	-0.874973	3.567263	-2.609659

65	1	0	-2.598649	3.800854	-2.283020
66	1	0	-1.927502	2.168768	-2.340062
67	6	0	-1.262905	4.762280	-0.133765
68	1	0	-1.054066	4.901121	0.935569
69	1	0	-2.179578	5.335357	-0.361606
70	1	0	-0.446205	5.231344	-0.697113
71	6	0	-2.291490	-2.821021	-1.983475
72	1	0	-1.568688	-2.157140	-2.473864
73	1	0	-2.912822	-3.273374	-2.762032
74	1	0	-1.716523	-3.623080	-1.506826
75	6	0	-6.738427	-0.820680	-0.736813
76	1	0	-7.366394	-0.790535	0.159576
77	1	0	-7.150623	-1.572285	-1.417862
78	1	0	-6.827650	0.155094	-1.231271
79	1	0	-1.440601	0.123777	-1.227110

25, PBE0/6-31+G(d):
Sum of electronic and thermal Free Energies= -1076.836527

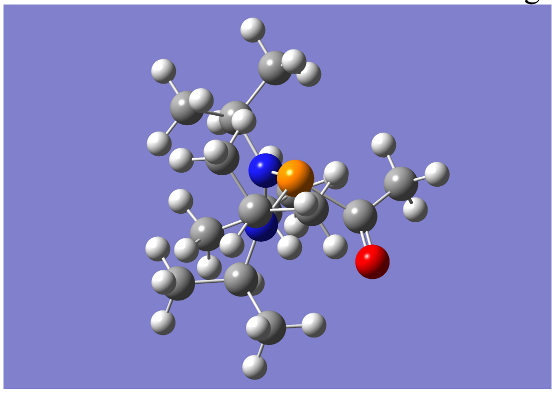

Center Number	Atomic Number	Atomic Type	Coordinates (Angstroms) X	Y	Z
1	15	0	0.029124	-0.143784	-0.824103
2	8	0	0.750947	-2.725571	-0.294019
3	7	0	-1.406904	0.253992	0.013771
4	7	0	1.427599	0.198803	0.075775
5	6	0	-0.107800	-2.013752	-0.780943
6	6	0	-1.940043	-0.456388	1.187515
7	1	0	-1.265526	-1.303990	1.359783
8	6	0	2.585993	0.753986	-0.652608
9	1	0	3.362609	0.903539	0.106498
10	6	0	1.840428	1.106511	2.342840
11	1	0	2.784500	1.609663	2.100285
12	1	0	1.875507	0.846577	3.407963
13	1	0	1.029318	1.824927	2.184891

14	6	0	-1.941392	0.398293	2.458095
15	1	0	-0.946243	0.796503	2.673477
16	1	0	-2.265603	-0.200312	3.317940
17	1	0	-2.633715	1.244317	2.371560
18	6	0	2.770649	-1.152381	1.713707
19	1	0	2.599641	-2.056262	1.124742
20	1	0	2.815817	-1.427001	2.774953
21	1	0	3.748442	-0.732576	1.447895
22	6	0	-2.173923	1.429936	-0.441977
23	1	0	-3.019269	1.518943	0.249887
24	6	0	2.305355	2.121616	-1.271227
25	1	0	1.556284	2.047403	-2.067745
26	1	0	3.221227	2.533615	-1.712489
27	1	0	1.938857	2.824489	-0.515646
28	6	0	1.638682	-0.147809	1.487684
29	1	0	0.715085	-0.632191	1.820869
30	6	0	-3.335305	-1.038894	0.940453
31	1	0	-4.089515	-0.253403	0.812780
32	1	0	-3.644806	-1.647934	1.798113
33	1	0	-3.350880	-1.672284	0.049112
34	6	0	3.145944	-0.219107	-1.691223
35	1	0	3.346933	-1.196964	-1.242858
36	1	0	4.077715	0.167273	-2.123433
37	1	0	2.431118	-0.362187	-2.510916
38	6	0	-1.381384	2.731517	-0.346946
39	1	0	-0.969922	2.867947	0.658738
40	1	0	-2.024557	3.589517	-0.578409
41	1	0	-0.549088	2.737594	-1.058722
42	6	0	-2.754160	1.236013	-1.842071
43	1	0	-1.952263	1.131045	-2.582048
44	1	0	-3.369719	2.097304	-2.129349
45	1	0	-3.380449	0.338024	-1.885009
46	6	0	-1.249183	-2.616562	-1.574249
47	1	0	-0.824297	-3.111271	-2.456127
48	1	0	-1.744189	-3.387733	-0.974314
49	1	0	-1.978645	-1.872223	-1.902398

26, PBE0/6-31+G(d):

Sum of electronic and thermal Free Energies= -612.934365

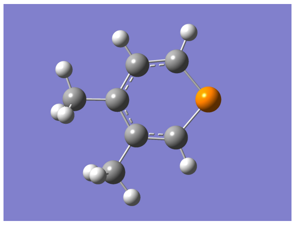

Center Number	Atomic Number	Atomic Type	Coordinates (Angstroms) X	Y	Z
1	6	0	-0.048986	-0.474837	0.033209
2	6	0	1.342330	-0.547406	0.089003
3	6	0	1.358228	2.099581	0.014919
4	6	0	-0.024784	1.974811	-0.034754
5	6	0	-0.724449	0.763321	-0.027563
6	1	0	1.781644	-1.544301	0.134467
7	1	0	1.777570	3.104549	0.002646
8	1	0	-0.617877	2.888673	-0.083235
9	15	0	2.471655	0.772603	0.095277
10	6	0	-0.850297	-1.751174	0.038279
11	1	0	-1.466966	-1.843910	-0.864733
12	1	0	-0.196713	-2.626520	0.088040
13	1	0	-1.534799	-1.794413	0.894928
14	6	0	-2.227145	0.793222	-0.085684
15	1	0	-2.610088	0.263526	-0.967532
16	1	0	-2.677021	0.309885	0.791088
17	1	0	-2.599676	1.820920	-0.126952

27 isomer A, PBE0/6-31+G(d):

Sum of electronic and thermal Free Energies= -1240.977494

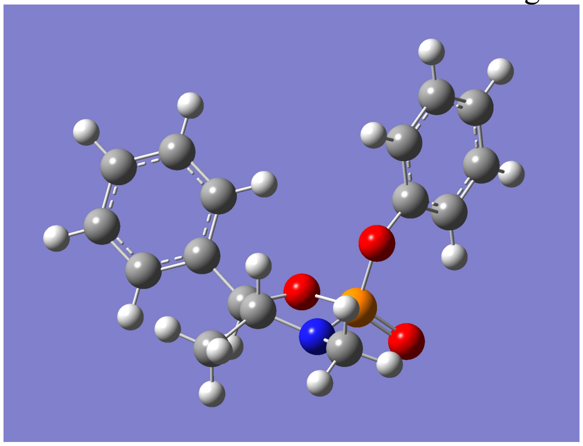

Center Number	Atomic Number	Atomic Type	Coordinates (Angstroms) X	Y	Z
1	6	0	-1.738228	0.616983	0.691159
2	6	0	-1.444685	1.708873	-0.363057
3	8	0	-0.477581	0.254756	1.291513
4	7	0	-0.308824	2.432326	0.209119
5	15	0	0.676102	1.339533	0.981704

6	8	0	1.518996	1.776116	2.109641
7	8	0	1.517468	0.682881	-0.255013
8	6	0	-2.648742	2.597021	-0.616345
9	1	0	-3.497354	1.988012	-0.946343
10	1	0	-2.445877	3.329350	-1.404081
11	1	0	-2.931744	3.135516	0.295221
12	6	0	-2.420251	-0.598935	0.129604
13	6	0	-3.794441	-0.771197	0.304161
14	6	0	-1.700467	-1.549214	-0.600723
15	6	0	-4.447770	-1.867711	-0.256783
16	1	0	-4.358423	-0.048221	0.890875
17	6	0	-2.350329	-2.648380	-1.153608
18	1	0	-0.625578	-1.435588	-0.717515
19	6	0	-3.726203	-2.808466	-0.987223
20	1	0	-5.517986	-1.991396	-0.111889
21	1	0	-1.779877	-3.386638	-1.711587
22	1	0	-4.231438	-3.669009	-1.418280
23	1	0	-1.147604	1.220901	-1.308662
24	1	0	-2.351470	1.057517	1.487793
25	6	0	0.269534	3.535775	-0.535035
26	1	0	0.573633	3.236656	-1.549367
27	1	0	-0.449871	4.357429	-0.605187
28	1	0	1.147738	3.906425	0.000705
29	6	0	2.476591	-0.310335	-0.171341
30	6	0	2.702535	-1.026332	-1.345553
31	6	0	3.201875	-0.584023	0.986382
32	6	0	3.662735	-2.033496	-1.361162
33	1	0	2.125884	-0.781048	-2.233187
34	6	0	4.155290	-1.601009	0.954442
35	1	0	3.030251	-0.002420	1.886363
36	6	0	4.391702	-2.328334	-0.209775
37	1	0	3.838742	-2.589941	-2.278361
38	1	0	4.722026	-1.818125	1.856346
39	1	0	5.139897	-3.116174	-0.221518

----

27 isomer B, PBE0/6-31+G(d):
Sum of electronic and thermal Free Energies= -1240.973470

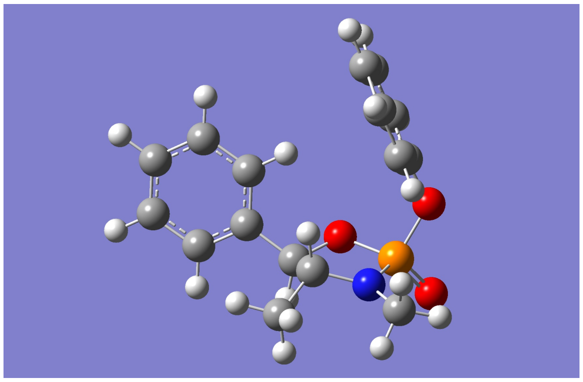

Center Number	Atomic Number	Atomic Type	Coordinates (Angstroms) X	Y	Z
1	15	0	-1.604330	-8.383055	-14.434989
2	8	0	-2.862145	-8.036454	-13.455354
3	8	0	-1.558733	-7.348179	-15.481901
4	8	0	-1.858446	-9.937408	-14.807375
5	7	0	-0.209894	-8.665695	-13.577563
6	6	0	-0.681883	-10.739640	-14.568010
7	6	0	0.052473	-10.087254	-13.378808
8	6	0	1.531213	-10.433210	-13.374023
9	6	0	0.527550	-7.639082	-12.879074
10	6	0	-2.001849	-12.537168	-13.382220
11	6	0	-2.312819	-13.876926	-13.171561
12	6	0	-1.683098	-14.871771	-13.920245
13	6	0	-0.746224	-14.519405	-14.888149
14	6	0	-0.442544	-13.176177	-15.105204
15	6	0	-1.060780	-12.176421	-14.352108
16	6	0	-4.451840	-9.641953	-12.694323
17	6	0	-4.957564	-10.444180	-11.674607
18	6	0	-4.329137	-10.484404	-10.429656
19	6	0	-3.192980	-9.708802	-10.204671
20	6	0	-2.682352	-8.897200	-11.216799
21	6	0	-3.312298	-8.876692	-12.458684
22	1	0	-0.042389	-10.656711	-15.457368
23	1	0	-0.402358	-10.437250	-12.436616
24	1	0	1.653231	-11.521010	-13.329989
25	1	0	2.024268	-10.061537	-14.279795
26	1	0	2.040814	-10.009742	-12.502511
27	1	0	0.173023	-6.658562	-13.208893
28	1	0	0.402086	-7.708473	-11.788009
29	1	0	1.597086	-7.698676	-13.109665
30	1	0	-2.504954	-11.767649	-12.802376
31	1	0	-3.050450	-14.145568	-12.419478
32	1	0	-1.927198	-15.917720	-13.752300

33	1	0	-0.257092	-15.287431	-15.481824
34	1	0	0.278979	-12.902248	-15.872650
35	1	0	-4.923354	-9.600567	-13.671455
36	1	0	-5.848201	-11.040582	-11.855606
37	1	0	-4.728290	-11.110303	-9.636035
38	1	0	-2.704747	-9.725197	-9.233521
39	1	0	-1.811874	-8.270095	-11.047982

----

27 isomer C, PBE0/6-31+G(d):
Sum of electronic and thermal Free Energies= -1240.973246

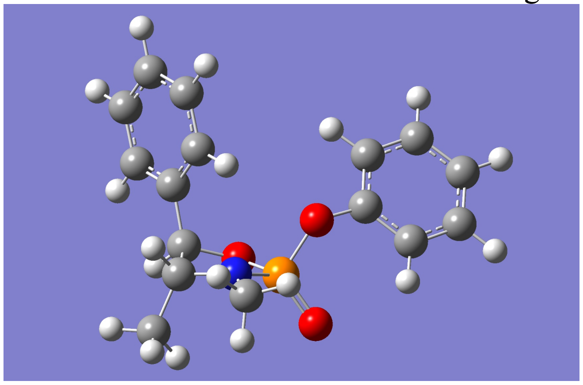

Center Number	Atomic Number	Atomic Type	Coordinates (Angstroms) X	Y	Z
1	6	0	-1.065836	0.283453	0.815238
2	6	0	-1.134250	1.287774	-0.363783
3	8	0	0.241405	0.439470	1.414547
4	7	0	0.248108	1.388074	-0.819597
5	15	0	1.322910	1.096664	0.413045
6	8	0	2.197492	-0.129427	-0.199718
7	6	0	-1.742456	2.622666	0.056833
8	1	0	-2.785012	2.483826	0.366161
9	1	0	-1.737961	3.340470	-0.770243
10	1	0	-1.184762	3.059880	0.893251
11	6	0	-1.340213	-1.154785	0.451025
12	6	0	-2.258265	-1.884125	1.209184
13	6	0	-0.707060	-1.780003	-0.629997
14	6	0	-2.545948	-3.212704	0.899348
15	1	0	-2.752597	-1.408871	2.054603
16	6	0	-0.991671	-3.106460	-0.939680
17	1	0	0.015997	-1.228006	-1.224341
18	6	0	-1.911374	-3.826978	-0.176650
19	1	0	-3.263117	-3.765715	1.500597
20	1	0	-0.492987	-3.581194	-1.781259
21	1	0	-2.131317	-4.862964	-0.422002

22	1	0	-1.736000	0.849178	-1.171685
23	1	0	-1.771562	0.588099	1.594028
24	6	0	0.581809	2.222874	-1.954923
25	1	0	-0.071278	1.968061	-2.797587
26	1	0	0.486954	3.295684	-1.740945
27	1	0	1.613251	2.025282	-2.260319
28	6	0	3.355528	-0.623993	0.386654
29	6	0	4.466656	0.183961	0.608912
30	6	0	3.379977	-1.984803	0.675951
31	6	0	5.620267	-0.393921	1.135306
32	1	0	4.411987	1.245788	0.395216
33	6	0	4.542612	-2.549050	1.195166
34	1	0	2.491001	-2.581403	0.492265
35	6	0	5.665596	-1.756317	1.426730
36	1	0	6.490644	0.231269	1.317686
37	1	0	4.566669	-3.611827	1.422317
38	1	0	6.571233	-2.197658	1.834273
39	8	0	2.160808	2.172700	0.981913

28, PBE0/6-31+G(d):
Sum of electronic and thermal Free Energies= -1240.978451

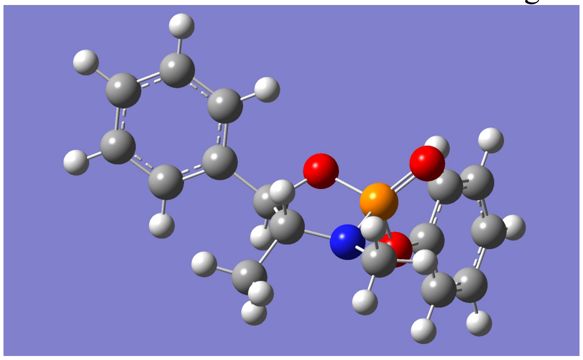

Center Number	Atomic Number	Atomic Type	Coordinates (Angstroms) X	Y	Z
1	6	0	1.369313	0.159074	-0.440532
2	6	0	1.502544	1.668570	-0.151251
3	8	0	0.399272	-0.338911	0.499582
4	7	0	0.146028	2.076531	0.210257
5	15	0	-0.714763	0.789842	0.817886
6	8	0	-1.863902	0.581980	-0.312986
7	8	0	-1.235145	0.811792	2.199309
8	6	0	-2.942651	-0.279209	-0.241107
9	6	0	-3.859359	-0.169064	-1.284839
10	6	0	-3.121882	-1.218477	0.771289
11	6	0	-4.965069	-1.012294	-1.319041

12	1	0	-3.690189	0.575567	-2.057324
13	6	0	-4.235257	-2.056257	0.722167
14	1	0	-2.421052	-1.280666	1.597038
15	6	0	-5.158454	-1.961670	-0.316160
16	1	0	-5.678160	-0.924607	-2.134879
17	1	0	-4.377630	-2.787978	1.513325
18	1	0	-6.022817	-2.619518	-0.342889
19	6	0	2.058605	2.440040	-1.334720
20	1	0	3.051080	2.059632	-1.600384
21	1	0	2.166956	3.503408	-1.098125
22	1	0	1.396435	2.346139	-2.202870
23	6	0	2.655766	-0.604601	-0.303172
24	6	0	3.381861	-0.958841	-1.441383
25	6	0	3.158535	-0.934469	0.958688
26	6	0	4.604415	-1.618406	-1.323998
27	1	0	2.986028	-0.725079	-2.428044
28	6	0	4.374633	-1.600841	1.076133
29	1	0	2.582533	-0.684837	1.846071
30	6	0	5.103208	-1.940048	-0.064123
31	1	0	5.159980	-1.890081	-2.217941
32	1	0	4.753651	-1.860376	2.061382
33	1	0	6.052396	-2.461322	0.030257
34	1	0	2.172344	1.801301	0.717168
35	1	0	0.960534	0.027878	-1.452303
36	6	0	-0.060783	3.415790	0.727732
37	1	0	0.637106	3.643785	1.546965
38	1	0	0.067728	4.157738	-0.067482
39	1	0	-1.078277	3.506883	1.115950

29, PBE0/6-31+G(d):
Sum of electronic and thermal Free Energies= -2859.887579

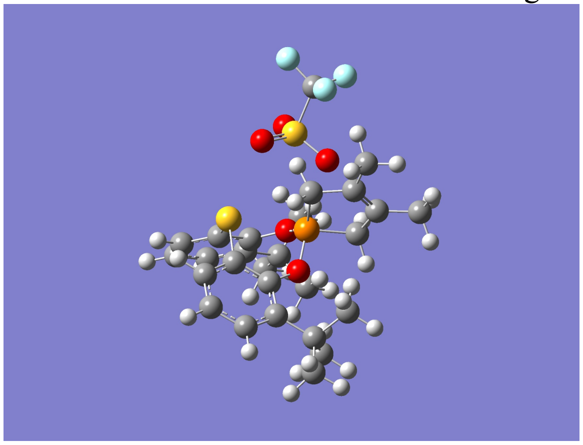

Center Number	Atomic Number	Atomic Type	Coordinates (Angstroms) X	Y	Z
1	8	0	-0.103785	1.142348	0.538573
2	8	0	2.013415	-0.309816	0.171858
3	16	0	0.106571	-0.050347	-2.150336
4	6	0	-0.090554	2.080742	-0.472206
5	6	0	0.028875	1.684107	-1.804142
6	6	0	0.008541	2.630412	-2.826140
7	1	0	0.075901	2.305455	-3.860107
8	6	0	-0.114581	3.969641	-2.494485
9	6	0	-0.219465	4.347308	-1.157034
10	1	0	-0.317969	5.403129	-0.933485
11	6	0	-0.210330	3.432349	-0.098312
12	6	0	2.595742	-0.528690	-1.060373
13	6	0	1.856422	-0.368688	-2.239248
14	6	0	2.469294	-0.564902	-3.475887
15	1	0	1.881992	-0.456434	-4.383119
16	6	0	3.814380	-0.897896	-3.523269
17	6	0	4.534770	-1.039967	-2.339986
18	1	0	5.584632	-1.300020	-2.409751
19	6	0	3.962491	-0.866491	-1.074088
20	6	0	-0.312510	3.892489	1.362498
21	6	0	-0.437905	5.418164	1.460411
22	1	0	0.430229	5.939337	1.038576
23	1	0	-0.504469	5.698458	2.517348
24	1	0	-1.344887	5.788905	0.969185
25	6	0	-1.553108	3.284152	2.038183
26	1	0	-2.468933	3.555055	1.503033
27	1	0	-1.630977	3.668909	3.062833
28	1	0	-1.524465	2.194517	2.086421
29	6	0	0.963938	3.494719	2.125858
30	1	0	1.106025	2.411814	2.158523
31	1	0	0.897753	3.851980	3.160681
32	1	0	1.853146	3.945567	1.668586
33	6	0	4.788990	-1.036816	0.208769
34	6	0	6.245786	-1.402392	-0.103161
35	1	0	6.329439	-2.353020	-0.642790
36	1	0	6.792490	-1.514834	0.839523
37	1	0	6.754358	-0.623749	-0.683541
38	6	0	4.211017	-2.176708	1.066642
39	1	0	4.229077	-3.124080	0.515076
40	1	0	3.182294	-1.987107	1.381053
41	1	0	4.820844	-2.303027	1.969583
42	6	0	4.813664	0.278805	1.007900

43	1	0	5.251325	1.087106	0.410529
44	1	0	5.431453	0.151861	1.905229
45	1	0	3.816958	0.592678	1.325049
46	6	0	0.592723	-0.611230	2.405414
47	6	0	-0.264185	-1.972603	0.208287
48	6	0	0.317211	-2.085881	2.580140
49	1	0	-0.167751	-0.001194	2.907470
50	1	0	1.584648	-0.307913	2.764299
51	6	0	-0.125831	-2.749854	1.496135
52	1	0	-1.294795	-1.838497	-0.141249
53	1	0	0.320876	-2.411607	-0.611446
54	15	0	0.449779	-0.356731	0.624208
55	6	0	-0.538300	-4.185312	1.419483
56	1	0	0.000650	-4.706969	0.617509
57	1	0	-0.360912	-4.725917	2.351905
58	1	0	-1.608209	-4.255894	1.184771
59	6	0	0.494935	-2.630552	3.961004
60	1	0	0.311158	-3.705418	4.021112
61	1	0	1.511636	-2.439548	4.329513
62	1	0	-0.194686	-2.133182	4.656148
63	1	0	4.307351	-1.049964	-4.479286
64	1	0	-0.139320	4.726378	-3.273463
65	16	0	-3.262526	-0.048460	0.657111
66	8	0	-2.212987	-0.325385	1.673433
67	8	0	-4.031284	1.177402	0.856585
68	8	0	-2.796313	-0.322598	-0.718025
69	6	0	-4.484912	-1.414233	0.966607
70	9	0	-4.983753	-1.348966	2.204144
71	9	0	-5.499986	-1.357474	0.102101
72	9	0	-3.892949	-2.615963	0.826734

----

anti-**30**, PBE0/6-31+G(d):
Sum of electronic and thermal Free Energies= -694.942735

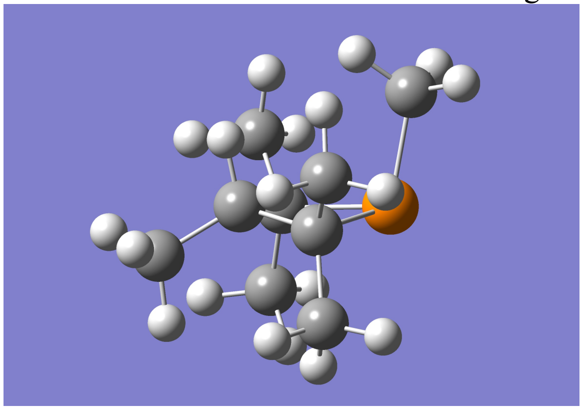

Center Number	Atomic Number	Atomic Type	Coordinates (Angstroms) X	Y	Z
1	6	0	-0.634364	1.055355	0.232469
2	6	0	0.786359	0.493048	-0.074743
3	6	0	-0.400642	2.550487	-0.138962
4	1	0	-0.752823	1.021279	1.325835
5	6	0	1.321942	-0.466198	0.978639
6	1	0	0.743934	-1.401626	0.968403
7	1	0	2.369409	-0.729712	0.783578
8	1	0	1.261241	-0.054539	1.991574
9	6	0	0.941863	-0.152247	-1.451694
10	1	0	1.996633	-0.386088	-1.640278
11	1	0	0.374663	-1.091619	-1.506723
12	1	0	0.603936	0.487844	-2.272093
13	6	0	-0.840083	2.946128	-1.548575
14	1	0	-1.935414	2.931307	-1.633971
15	1	0	-0.500999	3.964214	-1.775043
16	1	0	-0.437720	2.293363	-2.328980
17	6	0	-0.991016	3.542652	0.852888
18	1	0	-0.685045	4.570723	0.620253
19	1	0	-2.089417	3.512851	0.810314
20	1	0	-0.697546	3.329769	1.886073
21	6	0	-1.834593	0.342575	-0.375603
22	1	0	-1.882906	-0.699751	-0.036298
23	1	0	-2.771602	0.827975	-0.075330
24	1	0	-1.800085	0.334331	-1.470411
25	15	0	1.486516	2.267751	-0.105463
26	6	0	1.911182	2.570991	1.676379
27	1	0	2.894310	2.133293	1.882846
28	1	0	1.997073	3.651711	1.836050
29	1	0	1.195405	2.170295	2.402396

syn-**30** isomer A, PBE0/6-31+G(d):
Sum of electronic and thermal Free Energies= -694.942244

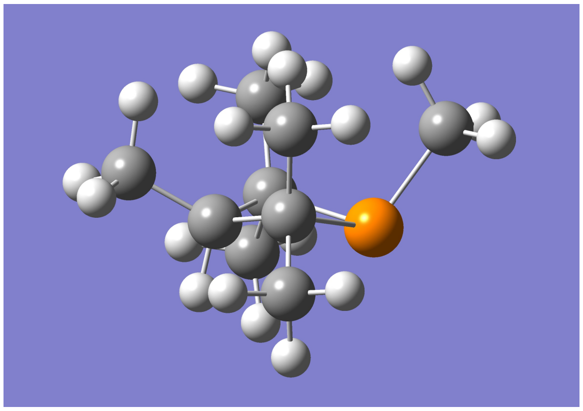

Center Number	Atomic Number	Atomic Type	Coordinates (Angstroms) X	Y	Z
1	6	0	-0.613860	1.066998	0.215126
2	6	0	0.834400	0.518560	0.011774
3	6	0	-0.357039	2.584430	-0.052855
4	1	0	-0.835329	0.972936	1.288962
5	6	0	1.257754	-0.492758	1.071699
6	1	0	0.662875	-1.414225	0.990427
7	1	0	2.313304	-0.770491	0.956835
8	1	0	1.128437	-0.093397	2.083976
9	6	0	1.115114	-0.057914	-1.371476
10	1	0	2.188615	-0.242857	-1.502176
11	1	0	0.601977	-1.022013	-1.494017
12	1	0	0.791185	0.587643	-2.192493
13	6	0	-0.682426	3.046005	-1.468848
14	1	0	-1.770797	3.074990	-1.618546
15	1	0	-0.302949	4.060567	-1.642499
16	1	0	-0.266502	2.401415	-2.247988
17	6	0	-1.046835	3.506840	0.946629
18	1	0	-0.756312	4.553086	0.786883
19	1	0	-2.140063	3.447900	0.842082
20	1	0	-0.790015	3.245675	1.979496
21	6	0	-1.746596	0.390034	-0.541631
22	1	0	-1.830457	-0.665524	-0.254884
23	1	0	-2.707354	0.868211	-0.314252
24	1	0	-1.604399	0.429197	-1.627062
25	15	0	1.479750	2.278441	0.335245
26	6	0	2.447705	2.792177	-1.160073
27	1	0	2.584036	3.879294	-1.129520
28	1	0	3.445637	2.342677	-1.101249
29	1	0	2.000877	2.524191	-2.122415

----

syn-**30** isomer B, PBE0/6-31+G(d):
Sum of electronic and thermal Free Energies= -694.938932

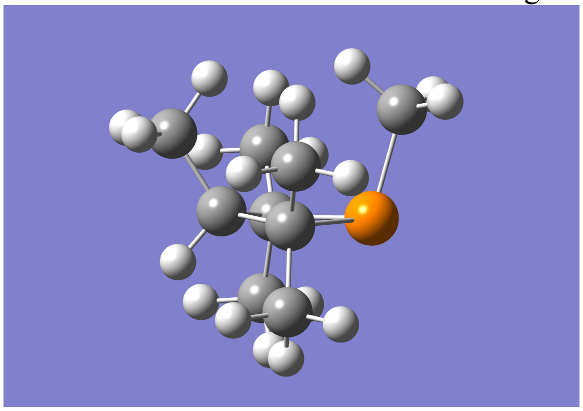

Center Number	Atomic Number	Atomic Type	Coordinates (Angstroms) X	Y	Z
1	6	0	-0.615538	1.067236	0.233297
2	6	0	0.832111	0.520312	-0.019833
3	6	0	-0.356187	2.580943	-0.084248
4	6	0	1.443902	-0.363936	1.058009
5	1	0	0.930732	-1.335045	1.099663
6	1	0	2.498195	-0.566337	0.829799
7	1	0	1.401772	0.073240	2.059614
8	6	0	0.938743	-0.204693	-1.361485
9	1	0	1.981988	-0.452725	-1.592244
10	1	0	0.364537	-1.142202	-1.333895
11	1	0	0.554400	0.394337	-2.194011
12	6	0	-0.895359	2.970761	-1.460921
13	1	0	-1.994650	2.941241	-1.459125
14	1	0	-0.583988	3.986966	-1.732174
15	1	0	-0.548202	2.300202	-2.254408
16	6	0	-0.844339	3.603706	0.932115
17	1	0	-0.483493	4.605295	0.665437
18	1	0	-1.942550	3.647601	0.939780
19	1	0	-0.516304	3.396372	1.954427
20	15	0	1.522911	2.288467	-0.180441
21	6	0	2.106786	2.674999	1.540015
22	1	0	3.100142	2.233369	1.677996
23	1	0	2.219119	3.760921	1.635475
24	1	0	1.457066	2.322637	2.347349
25	6	0	-1.212603	0.765921	1.602239
26	1	0	-2.209219	1.212786	1.696357
27	1	0	-1.323169	-0.315195	1.745346
28	1	0	-0.603769	1.145702	2.429209
29	1	0	-1.307141	0.644932	-0.513672

anti-**30[O]** isomer A, PBE0/6-31+G(d):
Sum of electronic and thermal Free Energies= -770.124628

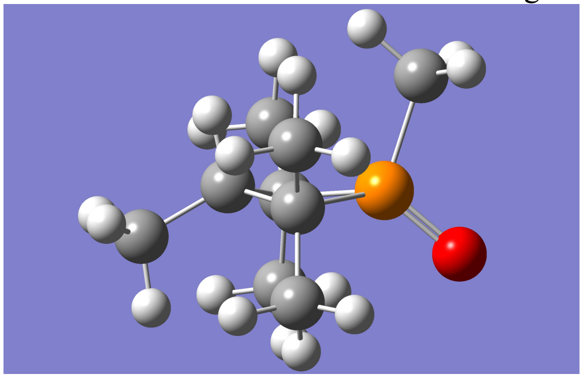

Center Number	Atomic Number	Atomic Type	Coordinates (Angstroms) X	Y	Z
1	6	0	-0.645752	1.048962	0.242762
2	6	0	0.789831	0.477342	-0.038461
3	6	0	-0.415570	2.562952	-0.102586
4	1	0	-0.803117	0.991034	1.330374
5	6	0	1.294559	-0.494718	1.020580
6	1	0	0.718628	-1.429543	0.980341
7	1	0	2.347294	-0.753476	0.854534
8	1	0	1.201006	-0.094382	2.036011
9	6	0	0.982819	-0.135746	-1.425662
10	1	0	2.042188	-0.359693	-1.590547
11	1	0	0.419236	-1.074203	-1.506455
12	1	0	0.669599	0.520774	-2.241066
13	6	0	-0.808439	2.972738	-1.522237
14	1	0	-1.900849	2.969144	-1.630579
15	1	0	-0.449449	3.986332	-1.730161
16	1	0	-0.388507	2.325291	-2.295877
17	6	0	-1.033237	3.534879	0.895128
18	1	0	-0.729180	4.567546	0.685385
19	1	0	-2.129377	3.498074	0.829335
20	1	0	-0.758517	3.305670	1.930698
21	6	0	-1.817914	0.350885	-0.428617
22	1	0	-1.880168	-0.697287	-0.111821
23	1	0	-2.765196	0.832345	-0.157309
24	1	0	-1.737109	0.364970	-1.520183
25	15	0	1.413359	2.230915	0.002029
26	6	0	1.976172	2.607964	1.697670
27	1	0	2.964236	2.159830	1.843911
28	1	0	2.073552	3.693785	1.797481
29	1	0	1.295842	2.236462	2.469628
30	8	0	2.357994	2.745844	-1.045452

anti-**30[O]** isomer B, PBE0/6-31+G(d):
Sum of electronic and thermal Free Energies= -770.120817

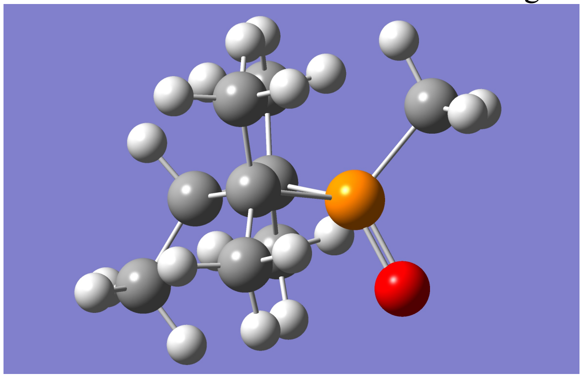

Center Number	Atomic Number	Atomic Type	Coordinates (Angstroms) X	Y	Z
1	6	0	-0.630481	1.037783	-0.468009
2	6	0	0.786752	0.473354	-0.049204
3	6	0	-0.419349	2.564756	-0.114738
4	15	0	1.402954	2.225067	0.009653
5	6	0	2.120309	2.684903	1.624577
6	1	0	3.113619	2.230530	1.701653
7	1	0	2.242695	3.772681	1.651190
8	1	0	1.511777	2.368772	2.476042
9	8	0	2.255350	2.680454	-1.142822
10	1	0	-1.398454	0.615865	0.196461
11	6	0	-1.043431	0.752836	-1.904877
12	1	0	-1.127950	-0.326203	-2.075652
13	1	0	-2.020888	1.197751	-2.122345
14	1	0	-0.327827	1.150018	-2.631743
15	6	0	0.736721	-0.250262	1.295430
16	1	0	0.205188	-1.204915	1.181112
17	1	0	1.743840	-0.475588	1.666950
18	1	0	0.212864	0.319567	2.070231
19	6	0	1.521707	-0.386343	-1.070761
20	1	0	2.514826	-0.653656	-0.690744
21	1	0	0.976644	-1.320706	-1.258158
22	1	0	1.670742	0.131631	-2.021465
23	6	0	-1.106894	2.946525	1.195352
24	1	0	-0.799358	3.943711	1.533076
25	1	0	-2.195366	2.968146	1.048092
26	1	0	-0.905311	2.239892	2.007541
27	6	0	-0.766959	3.581752	-1.195559
28	1	0	-1.840382	3.555768	-1.424079
29	1	0	-0.526639	4.593940	-0.849315
30	1	0	-0.205892	3.417628	-2.119110

syn-**30[O]** isomer A, PBE0/6-31+G(d):
Sum of electronic and thermal Free Energies= -770.123142

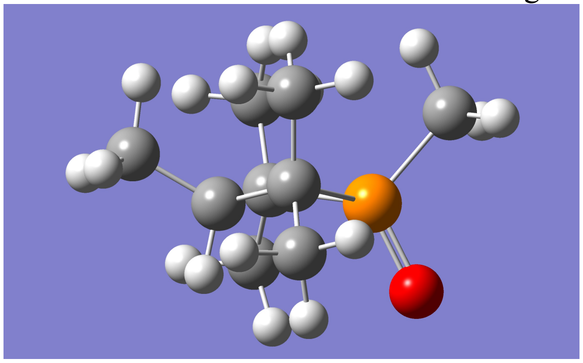

Center Number	Atomic Number	Atomic Type	Coordinates (Angstroms) X	Y	Z
1	6	0	-0.635789	1.053421	0.201385
2	6	0	0.820400	0.494314	0.007299
3	6	0	-0.384661	2.583426	-0.058436
4	1	0	-0.865407	0.954321	1.271522
5	6	0	1.258427	-0.466131	1.104985
6	1	0	0.612139	-1.354789	1.111021
7	1	0	2.289679	-0.804745	0.949317
8	1	0	1.211558	0.007086	2.090821
9	6	0	1.098044	-0.107284	-1.368120
10	1	0	2.169563	-0.304103	-1.492783
11	1	0	0.574376	-1.066257	-1.475455
12	1	0	0.779671	0.530306	-2.199001
13	6	0	-0.731728	3.058005	-1.467808
14	1	0	-1.821090	3.075235	-1.604029
15	1	0	-0.365326	4.078524	-1.631395
16	1	0	-0.316764	2.426245	-2.259956
17	6	0	-1.025781	3.494082	0.980559
18	1	0	-0.787261	4.546936	0.788190
19	1	0	-2.119238	3.388925	0.953639
20	1	0	-0.676580	3.255821	1.990177
21	6	0	-1.755395	0.383360	-0.578270
22	1	0	-1.842266	-0.674258	-0.301357
23	1	0	-2.719104	0.858524	-0.358633
24	1	0	-1.600305	0.431615	-1.661330
25	15	0	1.421971	2.241985	0.240080
26	8	0	1.965761	2.597701	1.596261
27	6	0	2.502257	2.824537	-1.111354
28	1	0	2.611332	3.910137	-1.018792
29	1	0	3.491133	2.372764	-0.981137
30	1	0	2.122673	2.583125	-2.107462

syn-**30[O]** isomer B, PBE0/6-31+G(d):
Sum of electronic and thermal Free Energies= -770.121206

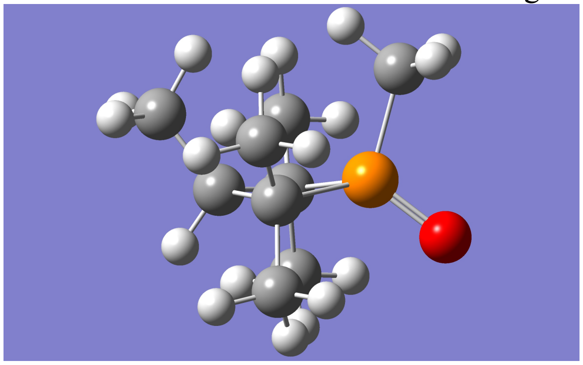

Center Number	Atomic Number	Atomic Type	Coordinates (Angstroms) X	Y	Z
1	6	0	0.000000	1.253904	-0.481302
2	6	0	1.206888	0.250556	-0.305790
3	6	0	-1.206889	0.250556	-0.305790
4	1	0	0.000000	1.633900	-1.512797
5	6	0	0.000000	-1.060832	2.103348
6	1	0	-0.887363	-1.604474	2.443421
7	1	0	0.887362	-1.604474	2.443422
8	1	0	0.000000	-0.061381	2.547362
9	15	0	0.000000	-1.037741	0.277094
10	6	0	2.320485	0.655554	0.653430
11	1	0	3.020350	-0.176815	0.794452
12	1	0	2.894974	1.496240	0.242229
13	1	0	1.958757	0.955530	1.641489
14	6	0	1.822713	-0.148171	-1.647923
15	1	0	2.356607	0.710326	-2.078035
16	1	0	2.536492	-0.969333	-1.520937
17	1	0	1.077552	-0.481916	-2.375177
18	6	0	0.000000	2.458900	0.449636
19	1	0	0.883609	3.082756	0.274180
20	1	0	-0.883609	3.082756	0.274180
21	1	0	0.000000	2.183746	1.510400
22	6	0	-1.822713	-0.148171	-1.647924
23	1	0	-2.536492	-0.969332	-1.520937
24	1	0	-2.356606	0.710326	-2.078035
25	1	0	-1.077552	-0.481916	-2.375178
26	6	0	-2.320485	0.655554	0.653430
27	1	0	-2.894974	1.496240	0.242229
28	1	0	-3.020351	-0.176815	0.794452
29	1	0	-1.958757	0.955530	1.641489
30	8	0	0.000000	-2.420108	-0.309549

anti-**31**, PBE0/6-31+G(d):
Sum of electronic and thermal Free Energies= -1286.723850

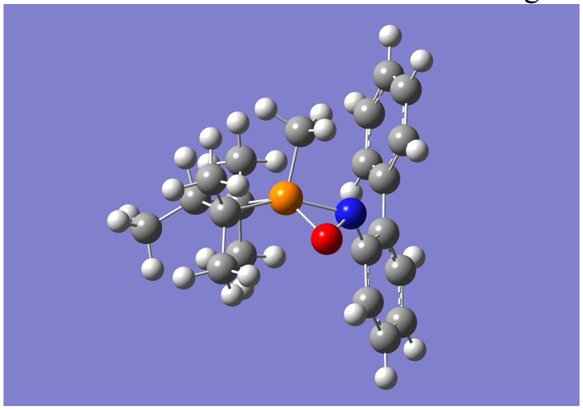

Center Number	Atomic Number	Atomic Type	Coordinates (Angstroms) X	Y	Z
1	6	0	-2.900995	-0.836946	1.051663
2	6	0	-3.241031	-0.262816	-0.360827
3	6	0	-1.375653	-0.484614	1.132714
4	15	0	-1.387657	-0.291963	-0.725083
5	6	0	-1.078577	-1.888049	-1.544809
6	1	0	-1.192149	-1.731984	-2.622802
7	1	0	-0.055568	-2.212961	-1.344209
8	1	0	-1.787059	-2.654730	-1.222323
9	8	0	-1.156498	1.020645	-1.659031
10	7	0	0.210921	0.468052	-1.275180
11	6	0	0.983873	1.453366	-0.672583
12	6	0	2.224244	1.079538	-0.086961
13	6	0	0.608333	2.806739	-0.673611
14	6	0	3.010035	2.079962	0.494654
15	6	0	1.422027	3.774834	-0.096541
16	1	0	-0.333553	3.081299	-1.135370
17	6	0	2.628231	3.419086	0.501459
18	1	0	3.966576	1.793782	0.926694
19	1	0	1.106593	4.815690	-0.117449
20	1	0	3.272074	4.170446	0.950175
21	6	0	2.713800	-0.320755	-0.064222
22	6	0	3.285544	-0.847264	1.104126
23	6	0	2.672881	-1.139991	-1.203401
24	6	0	3.798606	-2.142183	1.137352
25	1	0	3.313590	-0.235454	2.003317
26	6	0	3.188436	-2.432896	-1.171534
27	1	0	2.238441	-0.748874	-2.118553
28	6	0	3.751454	-2.943084	-0.001492
29	1	0	4.231137	-2.526544	2.058241
30	1	0	3.158693	-3.043936	-2.071091

31	1	0	4.151152	-3.953919	0.020675
32	1	0	-2.970341	-1.932898	0.980151
33	6	0	-3.776548	-0.402402	2.217021
34	1	0	-4.816505	-0.708674	2.051102
35	1	0	-3.443463	-0.867325	3.152915
36	1	0	-3.767566	0.682626	2.360747
37	6	0	-4.188874	-1.125780	-1.185953
38	1	0	-5.204754	-1.080453	-0.768463
39	1	0	-4.250298	-0.771230	-2.222244
40	1	0	-3.895542	-2.180668	-1.207131
41	6	0	-3.783705	1.168510	-0.344794
42	1	0	-3.852199	1.553313	-1.368302
43	1	0	-4.792252	1.189307	0.089287
44	1	0	-3.163745	1.870788	0.218615
45	6	0	-0.500612	-1.618967	1.654717
46	1	0	0.563866	-1.407420	1.508576
47	1	0	-0.670526	-1.745889	2.732869
48	1	0	-0.725584	-2.576000	1.171286
49	6	0	-1.046897	0.797368	1.898915
50	1	0	-1.300280	0.660599	2.958291
51	1	0	0.022716	1.016631	1.835490
52	1	0	-1.580496	1.680457	1.538902

----

syn-**31**, PBE0/6-31+G(d):
Sum of electronic and thermal Free Energies= -1286.722803

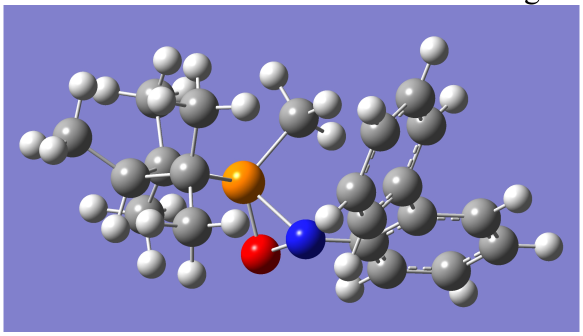

Center Number	Atomic Number	Atomic Type	Coordinates (Angstroms) X	Y	Z
1	6	0	3.144413	-0.736514	0.337428
2	6	0	0.472470	-0.704548	1.743398
3	15	0	1.270530	-0.513339	0.115345
4	6	0	3.642275	-0.658415	1.779872
5	1	0	3.354882	-1.557518	2.338485
6	1	0	4.738950	-0.601149	1.798981
7	1	0	3.267218	0.211476	2.330017
8	6	0	3.758512	-1.957778	-0.338627

9	1	0	4.853978	-1.866385	-0.368543
10	1	0	3.520739	-2.878233	0.208585
11	1	0	3.400220	-2.079459	-1.365488
12	8	0	0.927293	-1.608446	-1.029741
13	7	0	-0.290917	-0.696536	-0.907491
14	6	0	-1.400362	-1.446154	-0.528311
15	6	0	-2.559402	-0.754494	-0.086076
16	6	0	-1.424158	-2.849732	-0.581437
17	6	0	-3.677568	-1.499847	0.297318
18	6	0	-2.560608	-3.559666	-0.207634
19	1	0	-0.536055	-3.370617	-0.922151
20	6	0	-3.696396	-2.891811	0.242177
21	1	0	-4.564058	-0.961007	0.625938
22	1	0	-2.551954	-4.645755	-0.268497
23	1	0	-4.587300	-3.440533	0.534789
24	6	0	-2.620117	0.726090	-0.003540
25	6	0	-2.311460	1.536711	-1.105480
26	6	0	-3.050293	1.349289	1.176880
27	6	0	-2.437215	2.921029	-1.029133
28	1	0	-1.981845	1.067545	-2.028084
29	6	0	-3.169065	2.735740	1.256873
30	1	0	-3.288838	0.735121	2.042945
31	6	0	-2.863054	3.528247	0.152600
32	1	0	-2.207200	3.530459	-1.900382
33	1	0	-3.501990	3.196060	2.184287
34	1	0	-2.958854	4.609739	0.209838
35	6	0	1.877226	1.160221	-0.417629
36	6	0	3.333421	0.587384	-0.474384
37	1	0	3.506183	0.294935	-1.519797
38	6	0	1.375050	1.654253	-1.766335
39	1	0	0.339594	1.998081	-1.702011
40	1	0	2.000175	2.496774	-2.095307
41	1	0	1.422826	0.871658	-2.530026
42	6	0	1.624856	2.218482	0.657170
43	1	0	1.976884	1.925763	1.652298
44	1	0	2.136756	3.151668	0.389257
45	1	0	0.553111	2.437488	0.728715
46	6	0	4.459871	1.523525	-0.066541
47	1	0	5.432067	1.024705	-0.159139
48	1	0	4.483413	2.407528	-0.715549
49	1	0	4.361522	1.873124	0.966586
50	1	0	-0.336244	0.023929	1.848164
51	1	0	0.031455	-1.706552	1.781032
52	1	0	1.184118	-0.597503	2.563716

----

32 isomer A, PBE0/6-31+G(d):

Sum of electronic and thermal Free Energies= -881.622393

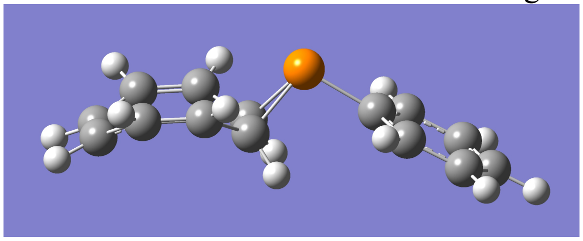

Center Number	Atomic Number	Atomic Type	Coordinates (Angstroms) X	Y	Z
1	6	0	0.989154	0.843832	0.565031
2	6	0	2.070438	0.602118	-0.408203
3	6	0	0.942759	3.983947	-0.726872
4	6	0	2.318703	1.134740	-1.622194
5	6	0	1.091176	3.344429	-2.016807
6	6	0	1.657749	2.171909	-2.385329
7	1	0	2.712084	-0.231490	-0.120963
8	1	0	0.306050	-0.006898	0.638633
9	1	0	3.126131	0.649641	-2.172975
10	1	0	1.715045	2.008385	-3.462203
11	6	0	0.365961	2.133956	0.970739
12	1	0	-0.679164	2.030575	1.275506
13	6	0	0.672278	3.498539	0.501989
14	1	0	0.551889	4.246448	1.286349
15	15	0	1.509011	1.416077	2.282096
16	1	0	0.764221	3.975967	-2.843824
17	1	0	0.990688	5.072577	-0.783384
18	6	0	0.309859	0.499782	3.344000
19	6	0	-0.658471	1.184210	4.089594
20	6	0	0.423326	-0.887010	3.506473
21	6	0	-1.506813	0.497921	4.956475
22	1	0	-0.753390	2.264189	3.991128
23	6	0	-0.424546	-1.575091	4.372105
24	1	0	1.179659	-1.436412	2.948559
25	6	0	-1.392907	-0.883952	5.098459
26	1	0	-2.257440	1.044137	5.522736
27	1	0	-0.327556	-2.652726	4.480306
28	1	0	-2.053755	-1.419593	5.775233

32 isomer B, PBE0/6-31+G(d):
Sum of electronic and thermal Free Energies= -881.621470

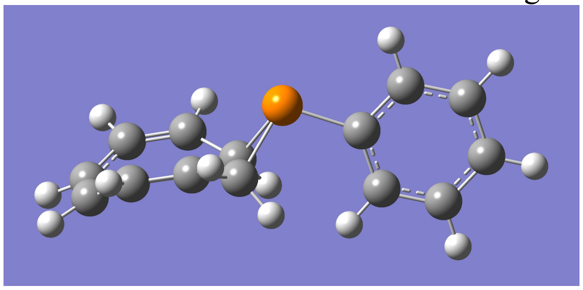

Center Number	Atomic Number	Atomic Type	Coordinates (Angstroms) X	Y	Z
1	6	0	-0.829831	-0.743742	0.306158
2	6	0	-1.865010	-1.683289	-0.159146
3	6	0	-3.212304	1.653807	-0.093224
4	6	0	-3.212509	-1.653366	-0.092133
5	6	0	-4.149667	0.677666	0.416648
6	6	0	-4.149733	-0.676748	0.417117
7	1	0	-1.434917	-2.608694	-0.543204
8	1	0	-0.221934	-1.180023	1.103644
9	1	0	-3.697770	-2.573617	-0.420633
10	1	0	-5.067529	-1.130816	0.792587
11	6	0	-0.829630	0.743900	0.304827
12	1	0	-0.221235	1.181237	1.101390
13	6	0	-1.864818	1.683278	-0.160699
14	1	0	-1.434574	2.608387	-0.545330
15	15	0	0.272415	-0.001206	-1.026091
16	1	0	-5.067400	1.132072	0.791854
17	1	0	-3.697353	2.574107	-0.421885
18	6	0	1.951173	-0.000465	-0.268406
19	6	0	3.012665	-0.000571	-1.184822
20	6	0	2.257249	0.000170	1.099445
21	6	0	4.337314	0.000075	-0.752244
22	1	0	2.798029	-0.001152	-2.252329
23	6	0	3.579860	0.000801	1.534187
24	1	0	1.465653	0.000173	1.846256
25	6	0	4.624766	0.000790	0.610245
26	1	0	5.143687	-0.000002	-1.481460
27	1	0	3.795584	0.001304	2.599994
28	1	0	5.656384	0.001288	0.952775

33, PBE0/6-31+G(d):
Sum of electronic and thermal Free Energies= -881.637881

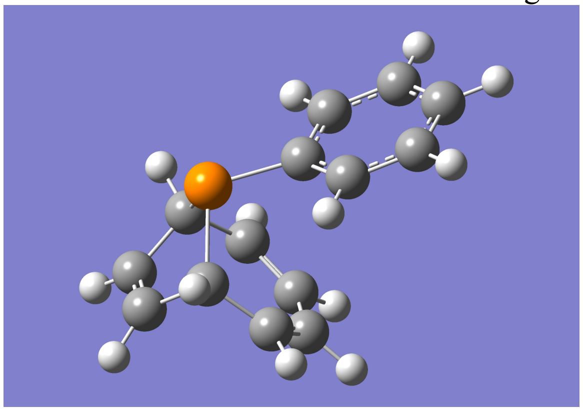

Center Number	Atomic Number	Atomic Type	Coordinates (Angstroms) X	Y	Z
1	6	0	0.141385	0.755507	-0.067749
2	6	0	1.420062	0.405274	-0.257823
3	6	0	0.776822	3.712313	-0.842291
4	6	0	2.446140	1.267308	0.442409
5	6	0	2.162202	3.331870	-1.050418
6	6	0	2.897776	2.330146	-0.515516
7	1	0	1.733950	-0.377191	-0.945744
8	1	0	-0.694089	0.287763	-0.584667
9	1	0	3.303919	0.674537	0.776127
10	1	0	3.940708	2.273897	-0.829672
11	6	0	-0.082905	1.959203	0.819273
12	1	0	-0.994163	1.850282	1.416094
13	6	0	-0.183097	3.175109	-0.054566
14	1	0	-1.153709	3.672410	-0.066517
15	15	0	1.428427	1.803786	1.936969
16	6	0	1.951934	3.517105	2.321312
17	6	0	1.012562	4.471423	2.732106
18	6	0	3.310499	3.851797	2.382675
19	6	0	1.417344	5.728441	3.174649
20	1	0	-0.049493	4.235281	2.705004
21	6	0	3.717771	5.108162	2.825312
22	1	0	4.062922	3.126646	2.078654
23	6	0	2.772093	6.052433	3.220829
24	1	0	0.671379	6.456837	3.483892
25	1	0	4.777534	5.349626	2.860326
26	1	0	3.088963	7.033341	3.566108
27	1	0	2.693657	3.978826	-1.747397
28	1	0	0.473721	4.589459	-1.412891

34, PBE0/6-31+G(d):
Sum of electronic and thermal Free Energies= -881.638639

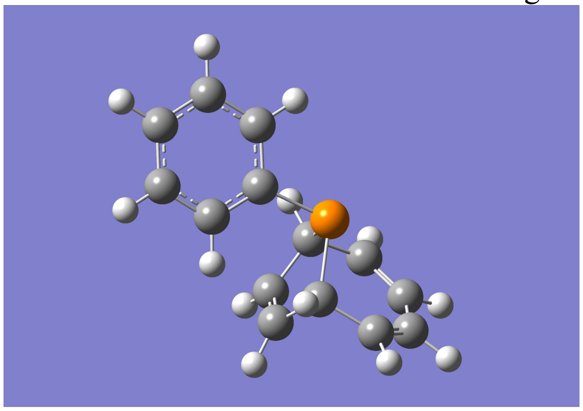

Center Number	Atomic Number	Atomic Type	Coordinates (Angstroms) X	Y	Z
1	6	0	1.083889	0.318916	1.684868
2	6	0	1.142387	1.434997	0.949186
3	6	0	3.412474	-0.940276	0.067277
4	6	0	1.096682	1.217188	-0.546201
5	6	0	3.470848	0.274843	-0.737758
6	6	0	2.528394	1.200999	-1.023797
7	1	0	1.341345	2.421971	1.364094
8	1	0	1.220822	0.300945	2.764631
9	1	0	0.535137	1.990514	-1.081514
10	1	0	2.839079	2.042123	-1.643159
11	6	0	0.992732	-0.964845	0.896272
12	1	0	0.341123	-1.711305	1.362242
13	6	0	2.396602	-1.496467	0.765069
14	1	0	2.619535	-2.414115	1.309313
15	15	0	0.304784	-0.467446	-0.791473
16	1	0	4.453553	0.461617	-1.169316
17	1	0	4.359858	-1.476066	0.112746
18	6	0	-1.458202	-0.153555	-0.349191
19	6	0	-2.336717	-1.222552	-0.581172
20	6	0	-1.991696	1.049204	0.130998
21	6	0	-3.699027	-1.106446	-0.315608
22	1	0	-1.948048	-2.155851	-0.986153
23	6	0	-3.357608	1.173816	0.379524
24	1	0	-1.341611	1.896789	0.326470
25	6	0	-4.214397	0.096264	0.163897
26	1	0	-4.359397	-1.950909	-0.497732
27	1	0	-3.752431	2.117474	0.748966
28	1	0	-5.278867	0.196001	0.361317

33[O], PBE0/6-31+G(d):
Sum of electronic and thermal Free Energies= -956.808962

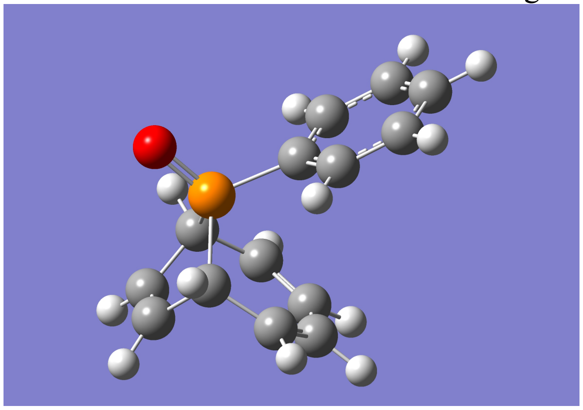

Center Number	Atomic Number	Atomic Type	Coordinates (Angstroms) X	Y	Z
1	6	0	0.140643	0.741392	-0.043384
2	6	0	1.419814	0.392426	-0.236991
3	6	0	0.758044	3.688901	-0.872322
4	6	0	2.449166	1.269118	0.444032
5	6	0	2.150971	3.308553	-1.083443
6	6	0	2.892439	2.315616	-0.547480
7	1	0	1.731354	-0.415125	-0.895106
8	1	0	-0.698722	0.247759	-0.527223
9	1	0	3.302501	0.693999	0.817855
10	1	0	3.920391	2.227365	-0.897728
11	6	0	-0.082217	1.959634	0.827411
12	1	0	-0.967739	1.859395	1.463364
13	6	0	-0.198318	3.159597	-0.079689
14	1	0	-1.183942	3.621776	-0.125603
15	15	0	1.443468	1.882976	1.870776
16	6	0	1.967333	3.552997	2.343747
17	6	0	1.020048	4.496412	2.753066
18	6	0	3.327094	3.869215	2.420064
19	6	0	1.426047	5.744152	3.218395
20	1	0	-0.040760	4.259474	2.710662
21	6	0	3.733594	5.116811	2.885609
22	1	0	4.075241	3.140264	2.116413
23	6	0	2.783068	6.056097	3.282140
24	1	0	0.683140	6.472609	3.533144
25	1	0	4.792680	5.355337	2.940656
26	1	0	3.100304	7.030267	3.645742
27	1	0	2.668088	3.937918	-1.806377
28	1	0	0.446792	4.544867	-1.469539
29	8	0	1.359099	0.928665	3.024648

34[O], PBE0/6-31+G(d):
Sum of electronic and thermal Free Energies=   -956.814336

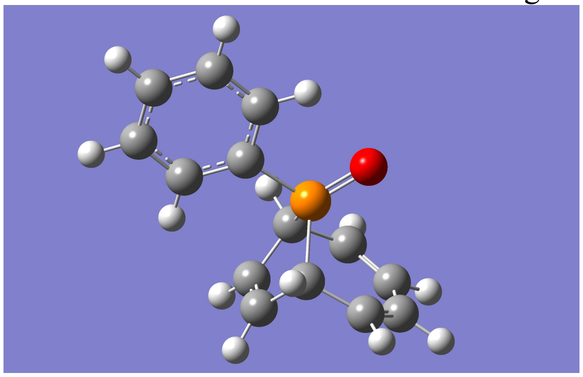

Center Number	Atomic Number	Atomic Type	Coordinates (Angstroms) X	Y	Z
1	6	0	1.214768	0.555217	1.697712
2	6	0	1.223535	1.566298	0.815239
3	6	0	3.427169	-0.952003	0.085255
4	6	0	1.102858	1.172458	-0.635242
5	6	0	3.436573	0.146096	-0.875734
6	6	0	2.489810	1.046807	-1.214697
7	1	0	1.388563	2.604351	1.096772
8	1	0	1.372631	0.691755	2.765771
9	1	0	0.501334	1.874811	-1.220931
10	1	0	2.754890	1.780309	-1.974499
11	6	0	1.084941	-0.828500	1.111330
12	1	0	0.471530	-1.496995	1.723918
13	6	0	2.468165	-1.399377	0.923273
14	1	0	2.719228	-2.251860	1.552373
15	15	0	0.279545	-0.486130	-0.521964
16	1	0	4.381108	0.244452	-1.408875
17	1	0	4.366514	-1.501942	0.120939
18	6	0	-1.499078	-0.211972	-0.222874
19	6	0	-2.351140	-0.859668	-1.126202
20	6	0	-2.051939	0.560837	0.807109
21	6	0	-3.733178	-0.732334	-1.005167
22	1	0	-1.912010	-1.464330	-1.915666
23	6	0	-3.434424	0.682627	0.924810
24	1	0	-1.407986	1.069353	1.517553
25	6	0	-4.277081	0.038600	0.019649
26	1	0	-4.384541	-1.239547	-1.712490
27	1	0	-3.854208	1.283165	1.728115
28	1	0	-5.355632	0.136862	0.115640
29	8	0	0.498455	-1.455935	-1.640945

Table S25. Coordinates of optimized structures for tricoordinate and tetracoordinate phosphorus compounds, ωB97x-D/6-31+G(d,p).

PH_3 ωB97x-D/6-31+G(d,p), chloroform IEFPCM:
Sum of electronic and thermal Free Energies= -343.118171 au

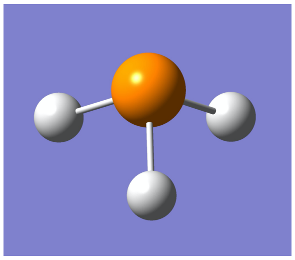

Center Number	Atomic Number	Atomic Type	Coordinates (Angstroms) X	Y	Z
1	15	0	0.921658	-0.120956	1.766892
2	1	0	1.672801	-1.321654	1.752193
3	1	0	1.672822	0.466651	2.814070
4	1	0	1.672822	0.492110	0.734414

$PMeH_2$ ωB97x-D/6-31+G(d,p), chloroform IEFPCM:
Sum of electronic and thermal Free Energies= -382.401528 au

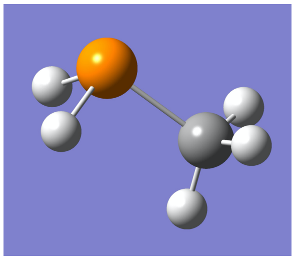

Center Number	Atomic Number	Atomic Type	Coordinates (Angstroms) X	Y	Z
1	15	0	0.661795	0.000023	-0.120044
2	1	0	0.937985	-1.039051	0.805323
3	1	0	0.937973	1.039023	0.805408
4	6	0	-1.193211	0.000006	0.025406
5	1	0	-1.587863	0.882980	-0.483542
6	1	0	-1.587853	-0.882930	-0.483617
7	1	0	-1.542405	-0.000040	1.059514

PMe_2H ωB97x-D/6-31+G(d,p), chloroform IEFPCM:
Sum of electronic and thermal Free Energies= -421.686898 au

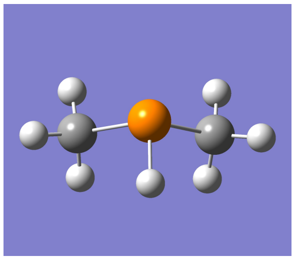

Center Number	Atomic Number	Atomic Type	Coordinates (Angstroms) X	Y	Z
1	15	0	0.000000	-0.655460	-0.104306
2	1	0	-0.000001	-1.093706	1.246125
3	6	0	1.424877	0.526060	0.021740
4	1	0	2.355997	-0.033538	0.145384
5	1	0	1.498029	1.095632	-0.909159
6	1	0	1.317359	1.226121	0.855205
7	6	0	-1.424876	0.526060	0.021738
8	1	0	-1.498027	1.095633	-0.909161
9	1	0	-2.355996	-0.033537	0.145380
10	1	0	-1.317359	1.226122	0.855204

PMe_3 ωB97x-D/6-31+G(d,p), chloroform IEFPCM:
Sum of electronic and thermal Free Energies= -460.974908 au

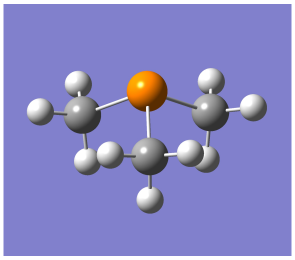

Center Number	Atomic Number	Atomic Type	Coordinates (Angstroms) X	Y	Z
1	15	0	-0.000044	0.000114	-0.598530
2	6	0	-0.991163	-1.297546	0.274108
3	1	0	-0.635821	-2.290365	-0.016914
4	1	0	-2.042096	-1.217193	-0.018598
5	6	0	-0.628182	1.507170	0.274307
6	1	0	-0.033731	2.377401	-0.018906
7	1	0	-0.580093	1.396838	1.363751
8	1	0	-0.919084	-1.201554	1.363599

9	1	0	-1.665959	1.695273	-0.015989
10	6	0	1.619286	-0.209577	0.274090
11	1	0	1.499964	-0.195299	1.363579
12	1	0	2.301390	0.594637	-0.016823
13	1	0	2.075240	-1.159842	-0.018744

PPh_3 ωB97x-D/6-31+G(d,p), chloroform IEFPCM:
Sum of electronic and thermal Free Energies= -1035.861929 au

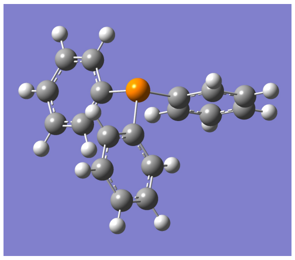

Center Number	Atomic Number	Atomic Type	Coordinates (Angstroms) X	Y	Z
1	15	0	-0.000783	-0.000972	-1.270943
2	6	0	-1.604467	-0.392528	-0.450724
3	6	0	-2.378075	-1.415343	-1.015206
4	6	0	-2.084171	0.263779	0.687880
5	6	0	-3.591901	-1.788783	-0.443530
6	1	0	-2.026885	-1.926645	-1.908190
7	6	0	-3.305490	-0.100478	1.253424
8	1	0	-1.503663	1.062742	1.139063
9	6	0	-4.059619	-1.129354	0.692710
10	1	0	-4.175938	-2.587329	-0.890455
11	1	0	-3.665129	0.419612	2.136043
12	1	0	-5.009432	-1.412984	1.135293
13	6	0	0.462094	1.584162	-0.451981
14	6	0	1.266155	1.673431	0.689478
15	6	0	-0.034935	2.764557	-1.020142
16	6	0	1.558636	2.914248	1.254234
17	1	0	1.666316	0.772252	1.144025
18	6	0	0.245963	4.003496	-0.449421
19	1	0	-0.650048	2.714588	-1.915296
20	6	0	1.046479	4.080802	0.689740
21	1	0	2.185352	2.967161	2.139261
22	1	0	-0.152213	4.907761	-0.899169
23	1	0	1.273639	5.045854	1.131929
24	6	0	1.142029	-1.194174	-0.453753
25	6	0	2.415733	-1.345784	-1.017715
26	6	0	0.815369	-1.943615	0.681560

27	6	0	3.348961	-2.208759	-0.448675
28	1	0	2.681685	-0.781339	-1.908132
29	6	0	1.744480	-2.817823	1.244393
30	1	0	-0.167293	-1.846194	1.132901
31	6	0	3.013631	-2.949284	0.684394
32	1	0	4.333408	-2.309564	-0.895027
33	1	0	1.475282	-3.393886	2.124462
34	1	0	3.736378	-3.628937	1.124995

Methoxyphospholane ωB97x-D/6-31+G(d,p), chloroform IEFPCM:
Sum of electronic and thermal Free Energies= -685.402801 au

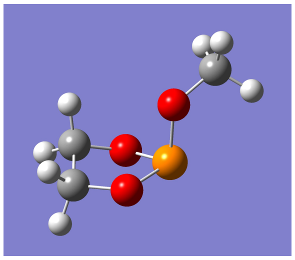

Center Number	Atomic Number	Atomic Type	Coordinates (Angstroms) X	Y	Z
1	8	0	-0.545765	1.059722	-0.672809
2	6	0	-1.461231	1.005980	0.432894
3	6	0	-1.952954	-0.441842	0.468227
4	1	0	-0.936199	1.277831	1.353634
5	1	0	-2.264826	1.717571	0.242582
6	1	0	-2.181774	-0.775490	1.481718
7	1	0	-2.824446	-0.589379	-0.175553
8	15	0	0.313202	-0.353472	-0.757845
9	8	0	-0.877974	-1.245700	-0.048606
10	6	0	2.632665	0.231459	0.362804
11	1	0	3.238666	-0.139162	1.190022
12	1	0	3.055216	-0.130791	-0.581520
13	1	0	2.642891	1.325480	0.367002
14	8	0	1.305234	-0.258304	0.562179

P(OMe)$_3$ isomer A ωB97x-D/6-31+G(d,p), chloroform IEFPCM:
Sum of electronic and thermal Free Energies= -686.585746 au

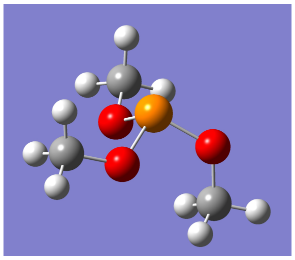

Center Number	Atomic Number	Atomic Type	Coordinates (Angstroms) X	Y	Z
1	15	0	-0.103921	-0.082646	-0.723489
2	6	0	1.588691	-1.901876	0.173885
3	1	0	1.627685	-2.677225	0.939747
4	1	0	1.472616	-2.373499	-0.808836
5	1	0	2.522227	-1.330177	0.190471
6	6	0	1.076117	1.913791	0.635273
7	1	0	1.861703	2.645381	0.446082
8	1	0	0.170960	2.419359	0.978276
9	1	0	1.408995	1.200164	1.393472
10	6	0	-2.516988	-0.415044	0.279687
11	1	0	-3.349114	0.185782	0.647279
12	1	0	-2.802590	-0.878301	-0.672167
13	1	0	-2.279537	-1.195813	1.008054
14	8	0	0.831021	1.247942	-0.611427
15	8	0	0.478174	-1.060694	0.483690
16	8	0	-1.409113	0.469349	0.113356

P(OMe)$_3$ isomer B ωB97x-D/6-31+G(d,p), chloroform IEFPCM:
Sum of electronic and thermal Free Energies= -686.583857 au

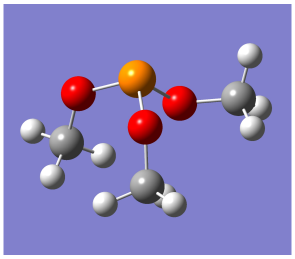

Center Number	Atomic Number	Atomic Type	Coordinates (Angstroms) X	Y	Z
1	6	0	2.281118	-0.690973	0.432317
2	8	0	0.871169	-0.893762	0.363258

3	15	0	0.072386	-0.249737	-0.942713
4	8	0	-1.419946	-0.773674	-0.541469
5	6	0	-1.978037	-0.835317	0.776513
6	8	0	0.111020	1.354359	-0.578522
7	1	0	2.658264	-1.343750	1.220006
8	1	0	2.768372	-0.951844	-0.514174
9	1	0	2.514382	0.349857	0.681478
10	1	0	-2.423713	0.127650	1.039326
11	1	0	-2.757639	-1.597395	0.751247
12	1	0	-1.219380	-1.111381	1.511403
13	6	0	-0.135387	1.911507	0.715506
14	1	0	-1.194289	2.165252	0.813709
15	1	0	0.461952	2.820999	0.795840
16	1	0	0.153186	1.216445	1.509358

$P(OMe)_3$ isomer C ωB97x-D/6-31+G(d,p), chloroform IEFPCM:
Sum of electronic and thermal Free Energies= -686.582561 au

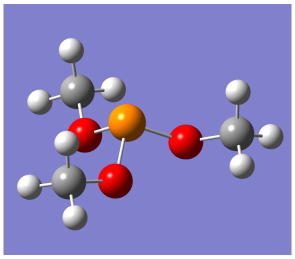

Center Number	Atomic Number	Atomic Type	Coordinates (Angstroms) X	Y	Z
1	15	0	0.001064	0.000808	0.421129
2	6	0	2.610853	0.055081	0.070333
3	1	0	3.361010	0.710756	-0.372249
4	1	0	2.685527	0.108743	1.162633
5	1	0	2.789095	-0.973521	-0.256707
6	6	0	-1.258286	-2.285235	0.072328
7	1	0	-1.067586	-3.263469	-0.369474
8	1	0	-1.250159	-2.376042	1.164719
9	1	0	-2.237140	-1.923259	-0.255678
10	6	0	-1.355442	2.230841	0.072046
11	1	0	-2.294038	2.554351	-0.378373
12	1	0	-1.449586	2.264836	1.163608
13	1	0	-0.550744	2.900657	-0.244983
14	8	0	-0.223719	-1.412986	-0.385860
15	8	0	1.337031	0.511452	-0.388062
16	8	0	-1.109837	0.900997	-0.388641

PCl_3 experimental geometry:

Center Number	Atomic Number	Atomic Type	Coordinates (Angstroms) X	Y	Z
1	15	0	0.000000	0.000000	0.734422
2	17	0	0.000000	1.808462	-0.216006
3	17	0	-1.566174	-0.904231	-0.216006
4	17	0	1.566174	-0.904231	-0.216006

H_3PO_4 ωB97x-D/6-31+G(d,p), water IEFPCM:
Sum of electronic and thermal Free Energies= -644.060644 au

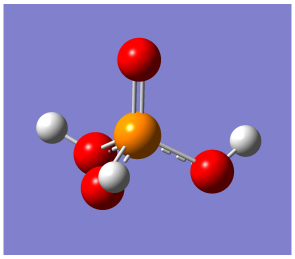

Center Number	Atomic Number	Atomic Type	Coordinates (Angstroms) X	Y	Z
1	15	0	-1.277694	1.092034	-0.159515
2	8	0	-1.127707	1.839422	1.243181
3	1	0	-0.275350	2.278991	1.358400
4	8	0	-0.710221	-0.361684	0.175921
5	1	0	-0.546536	-0.904548	-0.606055
6	8	0	-2.850241	0.826210	-0.232119
7	1	0	-3.349678	1.555157	-0.622413
8	8	0	-0.676938	1.775029	-1.328116

PH_4^+ BF_4^- ωB97x-D/6-31+G(d,p), chloroform IEFPCM:
Sum of electronic and thermal Free Energies= -768.052390

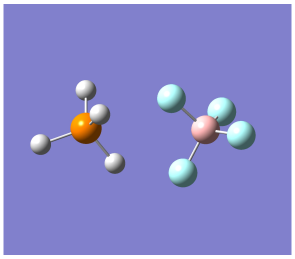

Center Number	Atomic Number	Atomic Type	Coordinates (Angstroms) X	Y	Z
1	15	0	-1.396101	0.990603	0.115250
2	1	0	-0.548080	-0.119621	0.104174
3	1	0	-1.141868	1.792820	1.221960
4	1	0	-1.251642	1.731880	-1.051767
5	1	0	-2.726017	0.574852	0.190744
6	5	0	2.119662	0.723050	-0.118487
7	9	0	1.108523	1.745066	-0.034069
8	9	0	3.021140	0.885746	0.930696
9	9	0	1.452031	-0.527274	-0.000238
10	9	0	2.750636	0.810940	-1.358259

$P(OPh)_4^+ PF_6^-$ ωB97x-D/6-31+G(d,p), chloroform IEFPCM:
Sum of electronic and thermal Free Energies= -2508.644577

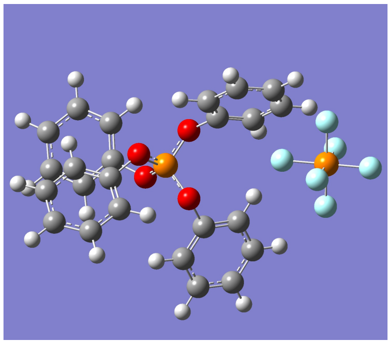

Center Number	Atomic Number	Atomic Type	Coordinates (Angstroms) X	Y	Z
1	15	0	-1.314896	0.372243	0.147938
2	8	0	-2.471285	1.010745	0.984455
3	8	0	-0.426065	1.552559	-0.335164
4	8	0	-0.562023	-0.633590	1.062146
5	8	0	-1.815299	-0.400023	-1.105630
6	6	0	-2.900121	-0.009576	-1.930738
7	6	0	-3.890403	-0.957973	-2.123530
8	6	0	-2.925302	1.247699	-2.510670
9	6	0	-4.971241	-0.618381	-2.932249
10	1	0	-3.815354	-1.927318	-1.644451
11	6	0	-4.021111	1.571434	-3.310341
12	1	0	-2.116714	1.953333	-2.354104
13	6	0	-5.040543	0.644998	-3.519435
14	1	0	-5.761009	-1.342879	-3.098233
15	1	0	-4.067516	2.550257	-3.774801

16	1	0	-5.886801	0.904875	-4.145957
17	6	0	-3.565414	0.248759	1.477294
18	6	0	-4.797451	0.459105	0.879489
19	6	0	-3.374377	-0.616678	2.542646
20	6	0	-5.890975	-0.251149	1.368236
21	1	0	-4.892712	1.155345	0.053457
22	6	0	-4.481114	-1.320688	3.015154
23	1	0	-2.395339	-0.735066	2.992470
24	6	0	-5.733320	-1.141307	2.430016
25	1	0	-6.865710	-0.106035	0.915504
26	1	0	-4.359130	-2.005750	3.847022
27	1	0	-6.589004	-1.691939	2.805467
28	6	0	0.111213	-1.808297	0.616842
29	6	0	1.438956	-1.713585	0.246496
30	6	0	-0.602052	-2.995301	0.630941
31	6	0	2.084326	-2.882858	-0.153261
32	1	0	1.969151	-0.768855	0.266850
33	6	0	0.061846	-4.153632	0.233765
34	1	0	-1.640730	-3.008732	0.943679
35	6	0	1.399138	-4.096343	-0.160045
36	1	0	3.122290	-2.825286	-0.461115
37	1	0	-0.469414	-5.099338	0.232545
38	1	0	1.908878	-5.001566	-0.472075
39	6	0	0.451460	2.283170	0.520583
40	6	0	-0.080075	3.125813	1.483900
41	6	0	1.807771	2.133233	0.296644
42	6	0	0.812920	3.843277	2.278248
43	1	0	-1.152462	3.223539	1.609367
44	6	0	2.682577	2.859315	1.102100
45	1	0	2.175914	1.477331	-0.484322
46	6	0	2.188005	3.708519	2.090598
47	1	0	0.427108	4.509918	3.041989
48	1	0	3.748846	2.748268	0.943069
49	1	0	2.875491	4.270125	2.713974
50	15	0	4.872282	0.030922	-1.279763
51	9	0	4.792032	1.658419	-1.119761
52	9	0	6.393496	0.040701	-0.691579
53	9	0	4.280781	-0.140186	0.242849
54	9	0	4.933545	-1.597213	-1.433354
55	9	0	5.441462	0.201857	-2.798458
56	9	0	3.331683	0.021654	-1.856622

O=P(OCH2)3P=O ωB97x-D/6-31+G(d,p), chloroform IEFPCM:
Sum of electronic and thermal Free Energies= -1176.592843

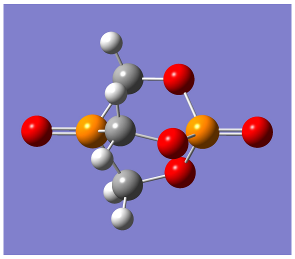

Center Number	Atomic Number	Atomic Type	Coordinates (Angstroms) X	Y	Z
1	6	0	-1.038323	0.471995	-1.348265
2	1	0	-1.418067	1.048455	-2.193315
3	6	0	-1.330248	-1.635034	0.506506
4	1	0	-0.610595	-2.342462	0.091674
5	6	0	-3.415911	0.229506	0.148297
6	1	0	-4.256540	-0.370389	0.499868
7	15	0	-1.117742	0.879370	1.250165
8	8	0	-0.542890	1.723881	2.299431
9	8	0	-0.580938	-0.638586	1.237024
10	8	0	-2.718401	0.717161	1.315929
11	8	0	-0.847550	1.393225	-0.251674
12	1	0	-3.789332	1.074104	-0.436595
13	1	0	-0.081571	0.016550	-1.616914
14	1	0	-2.007053	-2.158667	1.186832
15	15	0	-2.269081	-0.813939	-0.855792
16	8	0	-2.855084	-1.674725	-1.925179

$P(OMe)_4^+$ BF_4^- isomer A ωB97x-D/6-31+G(d,p), chloroform IEFPCM:
Sum of electronic and thermal Free Energies= -1226.032420

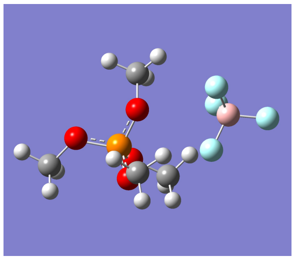

Center Number	Atomic Number	Atomic Type	Coordinates (Angstroms) X	Y	Z
1	15	0	-0.262751	0.683383	-0.456488
2	8	0	0.308142	1.773330	0.477708
3	8	0	0.061103	-0.690792	0.217527
4	8	0	-1.803642	0.681010	-0.662624
5	8	0	0.406226	0.852530	-1.848080

6	6	0	-0.133320	2.016782	1.841475
7	1	0	0.255896	1.225262	2.481585
8	1	0	0.285241	2.985662	2.097402
9	1	0	-1.221147	2.063497	1.871549
10	6	0	1.698915	1.489560	-2.068420
11	1	0	2.471090	0.943567	-1.526477
12	1	0	1.863326	1.423119	-3.140697
13	1	0	1.630031	2.527210	-1.747131
14	6	0	-0.392395	-1.957701	-0.324388
15	1	0	0.006525	-2.716305	0.344198
16	1	0	-1.482127	-1.983575	-0.327877
17	1	0	0.005925	-2.091524	-1.331008
18	6	0	-2.529005	1.660812	-1.469275
19	1	0	-2.092522	1.694894	-2.466605
20	1	0	-3.549739	1.288711	-1.502152
21	1	0	-2.468013	2.632500	-0.982946
22	9	0	0.644894	4.823083	0.304431
23	9	0	-1.578479	4.212396	0.263114
24	9	0	-0.264964	3.884656	-1.598878
25	9	0	-0.855637	6.021068	-0.970544
26	5	0	-0.507667	4.746431	-0.499489

$P(OMe)_4^+$ BF_4^- isomer B ωB97x-D/6-31+G(d,p), chloroform IEFPCM:
Sum of electronic and thermal Free Energies= -1226.026778

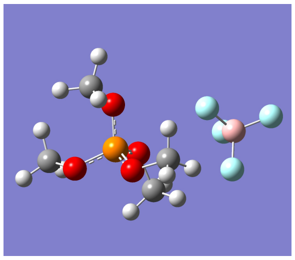

Center Number	Atomic Number	Atomic Type	Coordinates (Angstroms) X	Y	Z
1	15	0	-0.472168	0.511811	-0.046959
2	8	0	0.115229	0.946269	1.322095
3	8	0	-0.060053	-1.000416	-0.068697
4	8	0	-2.013666	0.707908	-0.162401
5	8	0	0.050613	1.275177	-1.288624
6	6	0	-0.151944	2.223701	1.983856
7	1	0	-1.219771	2.437097	1.955763
8	1	0	0.191932	2.078446	3.004518
9	1	0	0.402242	3.009027	1.473998
10	6	0	1.461759	1.575257	-1.501744
11	1	0	2.051416	0.660988	-1.418575

12	1	0	1.508902	1.980341	-2.508025
13	1	0	1.770866	2.332263	-0.782870
14	6	0	-0.270693	-1.817845	-1.250357
15	1	0	0.124990	-2.797335	-0.995149
16	1	0	-1.336560	-1.883947	-1.472353
17	1	0	0.272158	-1.395553	-2.096584
18	6	0	-2.944341	0.054300	0.740672
19	1	0	-2.650280	0.223924	1.777472
20	1	0	-3.906761	0.517569	0.541048
21	1	0	-2.979031	-1.012912	0.519575
22	9	0	-0.488514	6.044064	-0.710217
23	9	0	-1.256051	3.906605	-0.315349
24	9	0	0.987158	4.379988	-0.099439
25	9	0	0.014979	4.347603	-2.188936
26	5	0	-0.194153	4.678673	-0.838410

(*i*-PrO)$_2$P(O)H isomer A ωB97x-D/6-31+G(d,p), chloroform IEFPCM:
Sum of electronic and thermal Free Energies= -804.474215 au

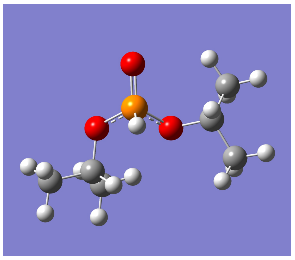

Center Number	Atomic Number	Atomic Type	Coordinates (Angstroms) X	Y	Z
1	15	0	-0.143774	0.265051	-0.965385
2	1	0	0.086071	0.245696	-2.351349
3	8	0	0.658753	1.284151	-0.248379
4	8	0	0.126676	-1.222357	-0.469674
5	8	0	-1.738754	0.380403	-0.851967
6	6	0	-2.417744	1.588432	-1.299005
7	1	0	-1.779538	2.092318	-2.037165
8	6	0	-0.528611	-2.365045	-1.094870
9	1	0	-0.955447	-2.038818	-2.051878
10	6	0	0.545428	-3.406845	-1.346265
11	1	0	1.002553	-3.715587	-0.401482
12	1	0	0.103693	-4.286727	-1.822760
13	1	0	1.325971	-3.010855	-2.000666
14	6	0	-1.640061	-2.845744	-0.179064
15	1	0	-1.227168	-3.149886	0.787343
16	1	0	-2.374979	-2.054120	-0.017768
17	1	0	-2.143279	-3.706592	-0.629228

18	6	0	-3.704660	1.141482	-1.966746
19	1	0	-4.257006	2.012736	-2.329948
20	1	0	-3.495497	0.483384	-2.813983
21	1	0	-4.334895	0.604600	-1.251132
22	6	0	-2.638331	2.500605	-0.104962
23	1	0	-3.261104	1.998747	0.641759
24	1	0	-1.685401	2.771946	0.354980
25	1	0	-3.146632	3.414687	-0.425786

(i-PrO)$_2$P(O)H isomer B ωB97x-D/6-31+G(d,p), chloroform IEFPCM:
Sum of electronic and thermal Free Energies= -804.476306 au

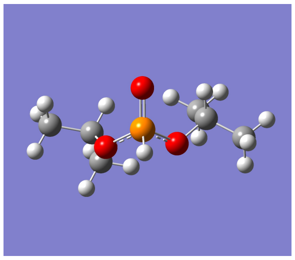

Center Number	Atomic Number	Atomic Type	Coordinates (Angstroms) X	Y	Z
1	15	0	-0.589159	0.151103	-2.833931
2	1	0	-1.157759	-0.524030	-3.913498
3	8	0	-0.084746	1.521446	-3.116746
4	8	0	0.491591	-0.941559	-2.399915
5	8	0	-1.718512	0.086620	-1.697709
6	6	0	-2.240765	1.264258	-1.020499
7	1	0	-1.938641	2.143611	-1.596743
8	6	0	1.356924	-0.712421	-1.248913
9	1	0	1.274733	0.341923	-0.960225
10	6	0	0.883918	-1.601523	-0.112401
11	1	0	0.957301	-2.654796	-0.399654
12	1	0	1.507855	-1.437413	0.771238
13	1	0	-0.153916	-1.379280	0.146457
14	6	0	2.778644	-0.998613	-1.693902
15	1	0	2.870252	-2.037328	-2.025931
16	1	0	3.065762	-0.338525	-2.515759
17	1	0	3.467842	-0.838441	-0.859710
18	6	0	-3.753052	1.136924	-1.009552
19	1	0	-4.194161	2.006891	-0.514435
20	1	0	-4.144857	1.082788	-2.028350
21	1	0	-4.058246	0.237045	-0.466645
22	6	0	-1.633443	1.326566	0.370347
23	1	0	-1.895154	0.431572	0.943486
24	1	0	-0.543909	1.405955	0.318588

25	1	0	-2.013247	2.203211	0.903223

(*i*-PrO)$_2$P(O)H isomer C ωB97x-D/6-31+G(d,p), chloroform IEFPCM:
Sum of electronic and thermal Free Energies= -804.475881

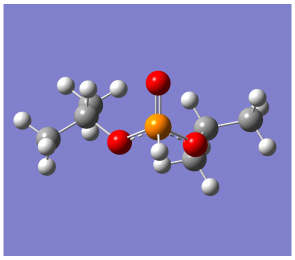

Center Number	Atomic Number	Atomic Type	Coordinates (Angstroms) X	Y	Z
1	15	0	-0.931447	-1.055572	-1.771111
2	1	0	-1.487317	-2.333467	-1.715408
3	8	0	-0.383605	-0.643594	-3.091004
4	8	0	0.141921	-1.042824	-0.579995
5	8	0	-2.171351	-0.221532	-1.209205
6	6	0	-2.078798	1.224257	-1.042903
7	1	0	-1.179614	1.574137	-1.563163
8	6	0	1.550500	-0.734552	-0.778116
9	1	0	1.768821	-0.832910	-1.845564
10	6	0	1.798343	0.696426	-0.332941
11	1	0	1.539737	0.819247	0.723492
12	1	0	2.854736	0.949127	-0.463191
13	1	0	1.205613	1.398496	-0.925997
14	6	0	2.342742	-1.755878	0.017114
15	1	0	2.100520	-1.684380	1.081958
16	1	0	2.122852	-2.769874	-0.326107
17	1	0	3.413748	-1.572094	-0.108053
18	6	0	-3.304174	1.829402	-1.701335
19	1	0	-3.278691	2.918512	-1.602770
20	1	0	-3.335956	1.575734	-2.763544
21	1	0	-4.214967	1.458155	-1.221528
22	6	0	-1.972029	1.527368	0.441135
23	1	0	-2.862920	1.169368	0.965969
24	1	0	-1.092119	1.044109	0.872061
25	1	0	-1.888269	2.607445	0.594143

(i-PrO)$_2$P(O)H isomer D ωB97x-D/6-31+G(d,p), chloroform IEFPCM:
Sum of electronic and thermal Free Energies= -804.474439 au

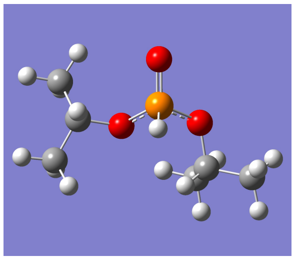

Center Number	Atomic Number	Atomic Type	Coordinates (Angstroms) X	Y	Z
1	15	0	-0.261929	-0.394790	-1.768950
2	1	0	-0.025668	-1.499339	-2.604663
3	8	0	0.041162	0.906968	-2.409727
4	8	0	0.549418	-0.754294	-0.433708
5	8	0	-1.775095	-0.536315	-1.298652
6	6	0	-2.278078	-1.769181	-0.704628
7	1	0	-1.558154	-2.571546	-0.910500
8	6	0	2.005137	-0.788363	-0.451744
9	1	0	2.336131	-0.957855	-1.484908
10	6	0	2.533883	0.546999	0.041691
11	1	0	2.195867	0.730109	1.066167
12	1	0	3.627827	0.539806	0.031072
13	1	0	2.182240	1.360424	-0.596999
14	6	0	2.423881	-1.963718	0.411419
15	1	0	2.069357	-1.824546	1.437316
16	1	0	2.013761	-2.898800	0.021388
17	1	0	3.514608	-2.042137	0.429022
18	6	0	-2.402650	-1.568083	0.795132
19	1	0	-2.782841	-2.482583	1.260037
20	1	0	-1.431411	-1.328967	1.233449
21	1	0	-3.099833	-0.752643	1.009515
22	6	0	-3.596661	-2.081818	-1.387094
23	1	0	-4.312395	-1.272072	-1.217143
24	1	0	-3.457700	-2.206986	-2.463802
25	1	0	-4.014731	-3.006613	-0.979232

(MeO)$_2$P(O)H isomer A ωB97x-D/6-31+G(d,p), chloroform IEFPCM:
Sum of electronic and thermal Free Energies= -647.335606

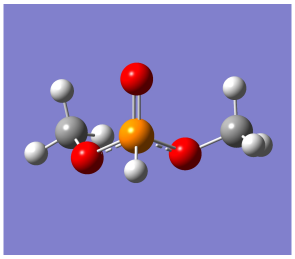

Center Number	Atomic Number	Atomic Type	Coordinates (Angstroms) X	Y	Z
1	15	0	0.006324	0.502474	0.399946
2	8	0	-1.433187	-0.158887	0.585010
3	8	0	0.945935	-0.790067	0.228368
4	8	0	0.168622	1.527221	-0.662635
5	6	0	-2.101425	-0.766620	-0.535347
6	1	0	-3.071307	-1.094765	-0.165898
7	1	0	-2.235918	-0.038360	-1.337921
8	1	0	-1.530438	-1.626041	-0.893973
9	6	0	2.282898	-0.650474	-0.278621
10	1	0	2.653456	-1.661643	-0.440033
11	1	0	2.279501	-0.097318	-1.219581
12	1	0	2.915972	-0.140484	0.452854
13	1	0	0.220703	0.960564	1.701602

$(MeO)_2P(O)H$ isomer B ωB97x-D/6-31+G(d,p), chloroform IEFPCM:
Sum of electronic and thermal Free Energies= -647.333608

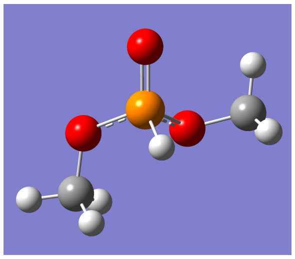

Center Number	Atomic Number	Atomic Type	Coordinates (Angstroms) X	Y	Z
1	15	0	-0.022658	-0.517756	0.224303
2	8	0	1.461140	-0.283224	-0.307063
3	8	0	-0.759229	0.826088	-0.253507
4	8	0	-0.618876	-1.797584	-0.220614
5	6	0	2.186907	0.909378	0.033482
6	1	0	3.206274	0.754001	-0.315348
7	1	0	1.744887	1.773119	-0.466495
8	1	0	2.191725	1.064455	1.116733

9	6	0	-2.164943	0.988328	-0.005596
10	1	0	-2.431239	1.971208	-0.390679
11	1	0	-2.733591	0.216071	-0.527491
12	1	0	-2.373865	0.947263	1.067758
13	1	0	0.057467	-0.378748	1.619993

$(MeO)_2P(O)H$ isomer C ωB97x-D/6-31+G(d,p), chloroform IEFPCM:
Sum of electronic and thermal Free Energies= -647.334502

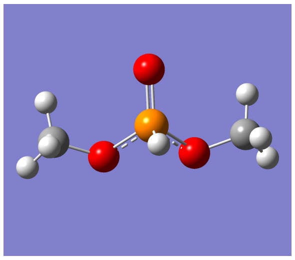

Center Number	Atomic Number	Atomic Type	Coordinates (Angstroms) X	Y	Z
1	15	0	-0.000022	0.202314	0.259950
2	8	0	-1.204094	-0.752279	-0.177799
3	8	0	1.204013	-0.752303	-0.177851
4	8	0	-0.000018	1.585985	-0.277185
5	6	0	-2.551551	-0.259736	-0.086854
6	1	0	-3.190794	-1.064021	-0.446619
7	1	0	-2.807106	-0.026037	0.950915
8	1	0	-2.677146	0.625857	-0.712926
9	6	0	2.551482	-0.259782	-0.086963
10	1	0	3.190697	-1.064078	-0.446754
11	1	0	2.677065	0.625809	-0.713042
12	1	0	2.807085	-0.026085	0.950794
13	1	0	0.000008	0.174226	1.663140

Ph_4P^+ Br^- ωB97x-D/6-31+G(d,p), chloroform IEFPCM:
Sum of electronic and thermal Free Energies= -3839.147643

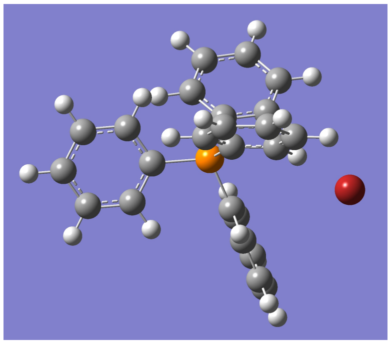

Center Number	Atomic Number	Atomic Type	Coordinates (Angstroms) X	Y	Z
1	15	0	0.782273	-0.003264	-0.024026
2	6	0	0.401540	-1.468688	-1.019092
3	6	0	-0.940594	-1.720481	-1.333733
4	6	0	1.402540	-2.340828	-1.461114
5	6	0	-1.266933	-2.823587	-2.114455
6	1	0	-1.744657	-1.096458	-0.945261
7	6	0	1.062978	-3.440760	-2.244446
8	1	0	2.441135	-2.183568	-1.194852
9	6	0	-0.268412	-3.678203	-2.578297
10	1	0	-2.308729	-3.014675	-2.348766
11	1	0	1.841745	-4.114276	-2.586087
12	1	0	-0.528740	-4.537130	-3.188424
13	6	0	0.413898	1.519374	-0.934094
14	6	0	-0.923115	1.783255	-1.260692
15	6	0	1.417539	2.426048	-1.292171
16	6	0	-1.240946	2.934808	-1.971752
17	1	0	-1.730263	1.130415	-0.930557
18	6	0	1.086642	3.574226	-2.007169
19	1	0	2.450884	2.257138	-1.012660
20	6	0	-0.238789	3.824733	-2.354718
21	1	0	-2.278774	3.135479	-2.215371
22	1	0	1.867313	4.274478	-2.284712
23	1	0	-0.492333	4.721131	-2.911466
24	6	0	2.530755	-0.022126	0.435419
25	6	0	2.888935	-0.068641	1.785960
26	6	0	3.527540	0.009644	-0.551482
27	6	0	4.234578	-0.083620	2.145693
28	1	0	2.125436	-0.092976	2.556371
29	6	0	4.867656	-0.005545	-0.184046
30	1	0	3.264795	0.047076	-1.604743
31	6	0	5.221584	-0.052136	1.164622
32	1	0	4.507383	-0.119585	3.194768
33	1	0	5.634577	0.019330	-0.950539
34	1	0	6.268633	-0.063699	1.448710
35	6	0	-0.228785	-0.038210	1.463029
36	6	0	-0.600599	1.162561	2.074522
37	6	0	-0.612536	-1.264201	2.014039
38	6	0	-1.359094	1.132685	3.238529
39	1	0	-0.317221	2.115706	1.639574
40	6	0	-1.373107	-1.283995	3.177042
41	1	0	-0.337844	-2.197355	1.532557
42	6	0	-1.747308	-0.088083	3.785965

43	1	0	-1.666856	2.063275	3.702665
44	1	0	-1.691776	-2.233191	3.593730
45	1	0	-2.357816	-0.107423	4.682768
46	35	0	-3.748085	0.003077	0.201755

Me_4P^+ Br^- ωB97x-D/6-31+G(d,p), chloroform IEFPCM:
Sum of electronic and thermal Free Energies= -3072.647950

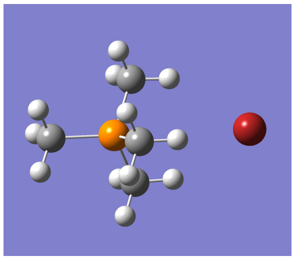

Center Number	Atomic Number	Atomic Type	Coordinates (Angstroms) X	Y	Z
1	15	0	1.500623	-0.001032	0.000480
2	6	0	3.308596	0.002347	0.005944
3	1	0	3.682258	-0.978617	-0.295871
4	1	0	3.676285	0.757800	-0.691965
5	1	0	3.672882	0.232989	1.009719
6	6	0	0.906831	1.617435	0.521953
7	1	0	1.265800	2.379884	-0.172805
8	1	0	-0.187505	1.583481	0.511989
9	1	0	1.268240	1.837822	1.528776
10	6	0	0.904209	-1.267577	1.133469
11	1	0	-0.190114	-1.230964	1.117314
12	1	0	1.251604	-2.249028	0.802589
13	1	0	1.274544	-1.065833	2.140752
14	6	0	0.907571	-0.352977	-1.663645
15	1	0	1.266818	-1.333826	-1.983244
16	1	0	-0.186978	-0.345732	-1.627222
17	1	0	1.266550	0.414415	-2.353391
18	35	0	-2.372869	-0.000798	0.014878

(*i*-PrO)$_2$P(O)Me isomer A ωB97x-D/6-31+G(d,p), chloroform IEFPCM:
Sum of electronic and thermal Free Energies= -843.771637

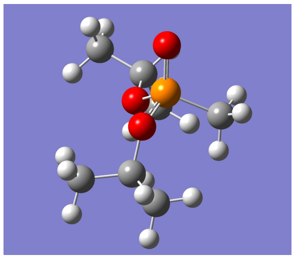

Center Number	Atomic Number	Atomic Type	Coordinates (Angstroms) X	Y	Z
1	15	0	-0.092075	0.253808	-1.158042
2	8	0	0.762026	1.277154	-0.501270
3	8	0	0.163360	-1.211282	-0.575122
4	8	0	-1.677185	0.415144	-0.923919
5	6	0	-2.348783	1.665401	-1.233384
6	1	0	-1.601331	2.389724	-1.578962
7	6	0	-0.650022	-2.362766	-0.931310
8	1	0	-1.390681	-2.052815	-1.677230
9	6	0	0.273940	-3.410263	-1.526278
10	1	0	1.033468	-3.704838	-0.796028
11	1	0	-0.300602	-4.297413	-1.808321
12	1	0	0.777308	-3.025173	-2.417288
13	6	0	-1.369886	-2.828361	0.321508
14	1	0	-0.645575	-3.111273	1.091221
15	1	0	-2.008403	-2.033000	0.712833
16	1	0	-1.992966	-3.698000	0.092213
17	6	0	-3.355378	1.393857	-2.338165
18	1	0	-3.883895	2.315507	-2.598394
19	1	0	-2.859510	1.015457	-3.236451
20	1	0	-4.090121	0.653775	-2.006395
21	6	0	-2.981168	2.177812	0.047917
22	1	0	-3.708241	1.452923	0.426981
23	1	0	-2.217717	2.343492	0.811523
24	1	0	-3.497976	3.123203	-0.141833
25	6	0	0.135606	0.164002	-2.946789
26	1	0	-0.143087	1.120304	-3.396676
27	1	0	1.188662	-0.030633	-3.160442
28	1	0	-0.479278	-0.627176	-3.382194

(*i*-PrO)$_2$P(O)Me isomer B ωB97x-D/6-31+G(d,p), chloroform IEFPCM:
Sum of electronic and thermal Free Energies= -843.774070

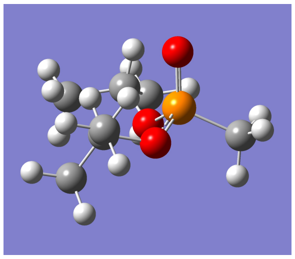

Center Number	Atomic Number	Atomic Type	Coordinates (Angstroms) X	Y	Z
1	15	0	0.206039	0.684115	-2.695676
2	8	0	0.725247	1.949944	-2.102588
3	8	0	1.034112	-0.641019	-2.330168
4	8	0	-1.297350	0.335359	-2.233038
5	6	0	-2.098739	1.218101	-1.405653
6	1	0	-1.512287	2.121369	-1.213264
7	6	0	1.249521	-0.993950	-0.936050
8	1	0	0.947689	-0.143351	-0.313287
9	6	0	0.389680	-2.203472	-0.611888
10	1	0	0.680612	-3.053429	-1.236942
11	1	0	0.519855	-2.483300	0.437931
12	1	0	-0.666032	-1.982626	-0.786866
13	6	0	2.736009	-1.240397	-0.755321
14	1	0	3.065396	-2.061030	-1.400293
15	1	0	3.307914	-0.343650	-1.005642
16	1	0	2.945808	-1.509969	0.283972
17	6	0	-3.354007	1.566944	-2.186210
18	1	0	-3.986939	2.236608	-1.596432
19	1	0	-3.098851	2.069662	-3.123038
20	1	0	-3.925604	0.662505	-2.417217
21	6	0	-2.383823	0.499904	-0.097864
22	1	0	-2.926788	-0.432462	-0.282979
23	1	0	-1.453767	0.265252	0.427569
24	1	0	-2.993992	1.134200	0.551955
25	6	0	0.177686	0.601694	-4.487792
26	1	0	1.194433	0.699063	-4.873029
27	1	0	-0.251151	-0.347043	-4.815095
28	1	0	-0.431605	1.426866	-4.862566

(*i*-PrO)$_2$P(O)Me isomer C ωB97x-D/6-31+G(d,p), chloroform IEFPCM:
Sum of electronic and thermal Free Energies= -843.772162

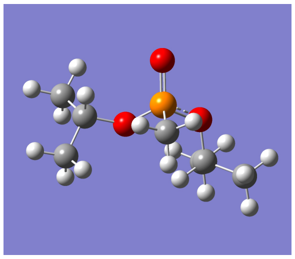

Center Number	Atomic Number	Atomic Type	Coordinates (Angstroms) X	Y	Z
1	15	0	-0.261310	-0.499243	-1.874080
2	8	0	0.018729	0.785559	-2.565997
3	8	0	0.567560	-0.754015	-0.517139
4	8	0	-1.765161	-0.584865	-1.342734
5	6	0	-2.250608	-1.672330	-0.508265
6	1	0	-1.429070	-2.377093	-0.337291
7	6	0	2.017841	-0.673384	-0.507367
8	1	0	2.361780	-0.394528	-1.510760
9	6	0	2.411446	0.416767	0.472867
10	1	0	2.047135	0.173032	1.475714
11	1	0	3.500718	0.510857	0.510898
12	1	0	1.987681	1.376343	0.167485
13	6	0	2.558991	-2.043995	-0.138640
14	1	0	2.201138	-2.337780	0.852979
15	1	0	2.240201	-2.798957	-0.862760
16	1	0	3.652517	-2.023004	-0.121136
17	6	0	-2.680005	-1.077872	0.820756
18	1	0	-3.053418	-1.867619	1.479451
19	1	0	-1.836032	-0.584392	1.308360
20	1	0	-3.477773	-0.344888	0.667911
21	6	0	-3.375856	-2.362525	-1.258319
22	1	0	-4.187978	-1.656892	-1.456938
23	1	0	-3.020655	-2.762789	-2.211902
24	1	0	-3.770836	-3.189263	-0.660654
25	6	0	0.023963	-1.953151	-2.905583
26	1	0	-0.582355	-1.870555	-3.809965
27	1	0	1.077854	-1.994074	-3.191844
28	1	0	-0.236268	-2.870636	-2.372338

Ph_3PO ωB97x-D/6-31+G(d,p), chloroform IEFPCM:
Sum of electronic and thermal Free Energies= -1111.098967

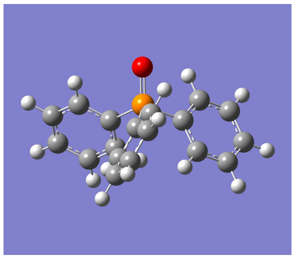

Center Number	Atomic Number	Atomic Type	Coordinates (Angstroms) X	Y	Z
1	15	0	-1.252477	1.375619	0.041148
2	8	0	-2.740347	1.429748	-0.184772
3	6	0	-0.301624	2.103782	-1.328493
4	6	0	-0.787850	1.919675	-2.627766
5	6	0	0.889904	2.807577	-1.129535
6	6	0	-0.083902	2.427018	-3.716150
7	1	0	-1.721162	1.386636	-2.782966
8	6	0	1.593131	3.313754	-2.221009
9	1	0	1.270863	2.967287	-0.125178
10	6	0	1.108067	3.122330	-3.513267
11	1	0	-0.466804	2.283217	-4.721351
12	1	0	2.516299	3.861225	-2.060250
13	1	0	1.655782	3.519159	-4.362266
14	6	0	-0.731106	2.268990	1.537808
15	6	0	-1.412340	3.447626	1.861802
16	6	0	0.324423	1.836000	2.345807
17	6	0	-1.035853	4.188405	2.978485
18	1	0	-2.241365	3.777173	1.242756
19	6	0	0.700012	2.579987	3.462710
20	1	0	0.853088	0.916978	2.110620
21	6	0	0.021769	3.755680	3.778300
22	1	0	-1.569564	5.100190	3.226553
23	1	0	1.518424	2.237717	4.087854
24	1	0	0.313574	4.332724	4.650161
25	6	0	-0.625760	-0.321418	0.235801
26	6	0	-1.430076	-1.235608	0.925780
27	6	0	0.621194	-0.726827	-0.249293
28	6	0	-0.987203	-2.538544	1.134264
29	1	0	-2.404735	-0.927086	1.291837
30	6	0	1.062705	-2.031761	-0.038228
31	1	0	1.249473	-0.029732	-0.795785
32	6	0	0.260422	-2.936585	0.654081
33	1	0	-1.616248	-3.243706	1.667902
34	1	0	2.030674	-2.341215	-0.419131
35	1	0	0.604673	-3.953264	0.815703

$(MeO)_2P(O)Me$ isomer A ωB97x-D/6-31+G(d,p), chloroform IEFPCM:
Sum of electronic and thermal Free Energies= -686.634325

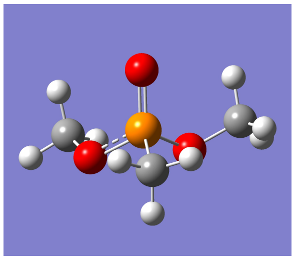

Center Number	Atomic Number	Atomic Type	Coordinates (Angstroms) X	Y	Z
1	15	0	0.016753	0.350772	0.166688
2	8	0	-0.099972	0.196361	1.643148
3	6	0	-0.411841	1.968102	-0.484305
4	1	0	-0.284305	1.986216	-1.567880
5	1	0	-1.453457	2.182780	-0.234289
6	1	0	0.226686	2.723956	-0.023122
7	8	0	1.474785	0.119844	-0.458462
8	8	0	-0.855110	-0.729396	-0.660899
9	6	0	2.203555	-1.072066	-0.125346
10	1	0	1.690553	-1.951689	-0.522135
11	1	0	3.181380	-0.972287	-0.594014
12	1	0	2.317247	-1.159249	0.957592
13	6	0	-2.119365	-1.173389	-0.151227
14	1	0	-2.435037	-1.996609	-0.791122
15	1	0	-2.018376	-1.517728	0.879942
16	1	0	-2.858790	-0.368489	-0.202660

$(MeO)_2P(O)Me$ isomer B ωB97x-D/6-31+G(d,p), chloroform IEFPCM:
Sum of electronic and thermal Free Energies= -686.631886

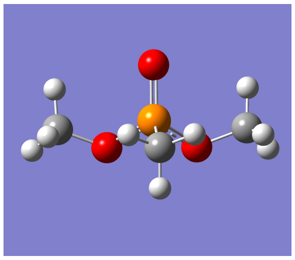

Center Number	Atomic Number	Atomic Type	Coordinates (Angstroms) X	Y	Z
1	15	0	0.019538	0.040588	0.170781
2	8	0	0.008431	-0.220567	1.636008
3	6	0	0.057571	1.793609	-0.250328

4	1	0	0.064610	1.922133	-1.334203
5	1	0	-0.819459	2.288518	0.173235
6	1	0	0.950848	2.251905	0.180414
7	8	0	1.213666	-0.648944	-0.653036
8	8	0	-1.195791	-0.598887	-0.662333
9	6	0	2.548338	-0.579314	-0.132014
10	1	0	2.584385	-0.976351	0.884756
11	1	0	3.164181	-1.189319	-0.791104
12	1	0	2.915211	0.451634	-0.143454
13	6	0	-2.530160	-0.475892	-0.150467
14	1	0	-3.165633	-1.059269	-0.815009
15	1	0	-2.589437	-0.872790	0.865259
16	1	0	-2.854746	0.569132	-0.162447

$(MeO)_2P(O)Me$ isomer C ωB97x-D/6-31+G(d,p), chloroform IEFPCM:
Sum of electronic and thermal Free Energies= -686.631522

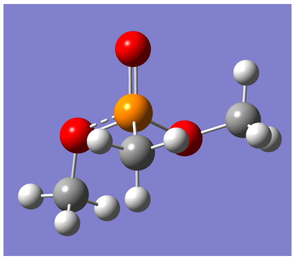

Center Number	Atomic Number	Atomic Type	Coordinates (Angstroms) X	Y	Z
1	15	0	0.029970	0.409115	-0.240900
2	8	0	0.646542	1.253310	-1.294734
3	6	0	-0.094901	1.211682	1.369190
4	1	0	-0.544401	0.547045	2.109995
5	1	0	0.904778	1.495532	1.706294
6	1	0	-0.699697	2.115359	1.269410
7	8	0	-1.439867	-0.078917	-0.646786
8	8	0	0.761096	-1.004149	0.027839
9	6	0	-2.188254	-1.016896	0.137555
10	1	0	-2.435557	-0.592538	1.114922
11	1	0	-3.107173	-1.209388	-0.414191
12	1	0	-1.627342	-1.945665	0.261292
13	6	0	2.182624	-1.051967	0.205379
14	1	0	2.449039	-2.106237	0.266900
15	1	0	2.690170	-0.589692	-0.644177
16	1	0	2.473625	-0.549408	1.132955

Me_3PO ωB97x-D/6-31+G(d,p), chloroform IEFPCM:
Sum of electronic and thermal Free Energies= -536.219087

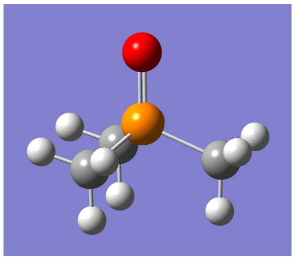

Center Number	Atomic Number	Atomic Type	Coordinates (Angstroms) X	Y	Z
1	15	0	-1.229472	1.331153	0.080856
2	6	0	-0.508904	2.150577	1.533592
3	1	0	-0.724337	3.221007	1.487399
4	1	0	-0.961870	1.739606	2.439265
5	1	0	0.573610	2.001569	1.571504
6	6	0	-0.328096	2.006525	-1.345029
7	1	0	-0.665596	1.504550	-2.255241
8	1	0	-0.541113	3.074816	-1.432755
9	1	0	0.749726	1.861333	-1.231252
10	6	0	-0.702624	-0.403638	0.200496
11	1	0	-1.149403	-0.855470	1.089652
12	1	0	-1.049333	-0.946803	-0.682199
13	1	0	0.386231	-0.478485	0.265945
14	8	0	-2.726723	1.498276	-0.020371

$EtOP(O)Me_2$ isomer A ωB97x-D/6-31+G(d,p), chloroform IEFPCM:
Sum of electronic and thermal Free Energies= -650.711626

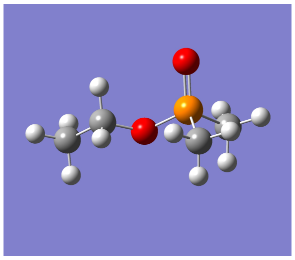

Center Number	Atomic Number	Atomic Type	Coordinates (Angstroms) X	Y	Z
1	15	0	-0.826611	-0.025495	0.169313
2	8	0	-1.009117	0.234758	1.633728
3	8	0	0.688944	-0.414589	-0.266350
4	6	0	1.786597	0.415631	0.156926
5	1	0	1.711338	0.586215	1.235187

6	1	0	1.721669	1.382694	-0.355971
7	6	0	3.077084	-0.292192	-0.195021
8	1	0	3.143821	-0.465547	-1.272491
9	1	0	3.928368	0.322804	0.110818
10	1	0	3.140888	-1.254039	0.320421
11	6	0	-1.719999	-1.455165	-0.469966
12	1	0	-1.411059	-2.343521	0.085176
13	1	0	-2.791217	-1.297314	-0.324255
14	1	0	-1.512574	-1.598594	-1.532589
15	6	0	-1.269257	1.400403	-0.857150
16	1	0	-1.077631	1.193714	-1.912705
17	1	0	-2.328835	1.626988	-0.715833
18	1	0	-0.685938	2.269771	-0.544116

$EtOP(O)Me_2$ isomer B ωB97x-D/6-31+G(d,p), chloroform IEFPCM:
Sum of electronic and thermal Free Energies= -650.707636

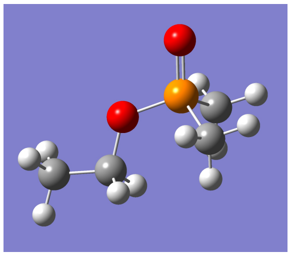

Center Number	Atomic Number	Atomic Type	Coordinates (Angstroms) X	Y	Z
1	15	0	-0.824081	0.012371	0.094574
2	8	0	-0.901930	0.270603	1.564241
3	8	0	0.720412	-0.216468	-0.347928
4	6	0	1.139107	-0.322301	-1.717119
5	1	0	1.000728	0.646032	-2.210916
6	1	0	0.526371	-1.068136	-2.237347
7	6	0	2.597131	-0.728665	-1.735589
8	1	0	2.733425	-1.699166	-1.251795
9	1	0	2.945161	-0.802078	-2.769947
10	1	0	3.208556	0.012252	-1.214334
11	6	0	-1.711562	-1.476565	-0.426888
12	1	0	-1.303938	-2.335590	0.110977
13	1	0	-2.767758	-1.364647	-0.169924
14	1	0	-1.626591	-1.646623	-1.503238
15	6	0	-1.439499	1.374887	-0.926273
16	1	0	-1.400507	1.135037	-1.991933
17	1	0	-2.476429	1.576856	-0.647170
18	1	0	-0.840450	2.267587	-0.730877

Table S26. Coordinates of optimized structures for 1a–34[O], ωB97x-D/6-31+G(d,p), IEFPCM solvation.

1a isomer A, ωB97x-D/6-31+G(d,p), benzene IEFPCM:
Sum of electronic and thermal Free Energies= -1309.900807

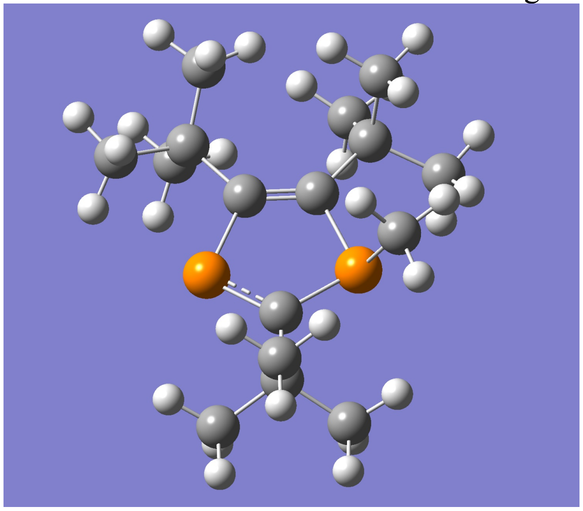

Center Number	Atomic Number	Atomic Type	Coordinates (Angstroms) X	Y	Z
1	6	0	-2.363275	4.068168	0.482844
2	6	0	-1.060448	4.316693	0.127036
3	6	0	-2.130296	6.709589	0.954073
4	15	0	-3.280992	5.501419	1.188552
5	15	0	-0.556168	6.053927	0.421863
6	6	0	-3.216252	2.764683	0.460480
7	6	0	-3.033509	1.926031	-0.818294
8	6	0	-2.945153	1.932263	1.730832
9	6	0	-4.730961	3.093820	0.475873
10	1	0	-3.266586	2.528873	-1.702322
11	1	0	-2.038213	1.511853	-0.946245
12	1	0	-3.730926	1.081905	-0.796858
13	1	0	-3.188556	2.518332	2.623423
14	1	0	-3.578235	1.037265	1.733276
15	1	0	-1.906755	1.615366	1.816298
16	1	0	-5.293169	2.160310	0.370176
17	1	0	-5.054993	3.564541	1.406515
18	1	0	-5.013145	3.749337	-0.355314
19	6	0	0.084650	3.392821	-0.385275
20	6	0	0.172867	2.042835	0.352986
21	6	0	-0.048721	3.186903	-1.909508
22	6	0	1.478181	4.026549	-0.143155
23	1	0	0.271949	2.206877	1.430654
24	1	0	-0.674534	1.384495	0.187674

25	1	0	1.065849	1.507422	0.012773
26	1	0	0.039823	4.141464	-2.436990
27	1	0	0.752364	2.530546	-2.268286
28	1	0	-1.003208	2.744847	-2.191421
29	1	0	2.242922	3.342212	-0.524943
30	1	0	1.625114	4.976972	-0.659832
31	1	0	1.669558	4.185334	0.921711
32	6	0	-2.367585	8.189677	1.268883
33	6	0	-3.404631	8.359580	2.393120
34	6	0	-1.066238	8.884376	1.708079
35	6	0	-2.910495	8.887671	0.002721
36	1	0	-4.371098	7.923952	2.121205
37	1	0	-3.063362	7.881320	3.316490
38	1	0	-3.562805	9.424846	2.593980
39	1	0	-0.302065	8.862272	0.924385
40	1	0	-1.268198	9.935208	1.941992
41	1	0	-0.647461	8.408323	2.599787
42	1	0	-3.082674	9.950555	0.207303
43	1	0	-2.211169	8.814873	-0.834492
44	1	0	-3.857970	8.437053	-0.307645
45	6	0	-0.348429	6.764137	-1.286700
46	1	0	-1.236520	6.577324	-1.895725
47	1	0	-0.187721	7.841282	-1.200652
48	1	0	0.525791	6.337121	-1.780811

1a isomer B, ωB97x-D/6-31+G(d,p), benzene IEFPCM:
Sum of electronic and thermal Free Energies= -1309.898638

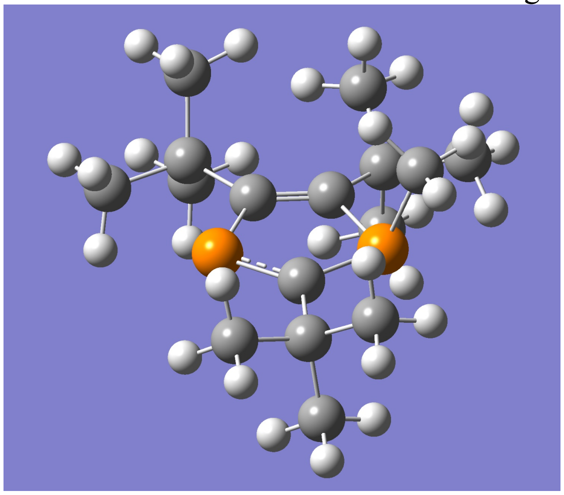

Center Number	Atomic Number	Atomic Type	Coordinates (Angstroms) X	Y	Z
1	6	0	-2.395109	4.037551	0.388938

2	6	0	-1.093568	4.282536	0.029558
3	6	0	-2.195509	6.701361	0.746612
4	15	0	-3.360862	5.505413	0.945888
5	15	0	-0.611150	6.054597	0.190909
6	6	0	-3.224312	2.717385	0.399055
7	6	0	-4.609842	2.903477	1.066125
8	6	0	-3.528626	2.300903	-1.055972
9	6	0	-2.568192	1.579620	1.204524
10	1	0	-4.528930	3.206230	2.114869
11	1	0	-5.233867	3.636588	0.548103
12	1	0	-5.139086	1.945221	1.035293
13	1	0	-2.633641	2.135932	-1.650811
14	1	0	-4.122260	1.379348	-1.065122
15	1	0	-4.114841	3.083102	-1.550181
16	1	0	-3.240046	0.714561	1.222020
17	1	0	-1.616556	1.239035	0.807236
18	1	0	-2.405739	1.896941	2.240089
19	6	0	0.104707	3.330335	-0.261897
20	6	0	1.291237	4.057344	-0.938959
21	6	0	0.638470	2.831472	1.101126
22	6	0	-0.188853	2.153279	-1.209903
23	1	0	1.063397	4.326852	-1.973472
24	1	0	1.613196	4.951310	-0.396990
25	1	0	2.146488	3.374899	-0.964863
26	1	0	-0.127609	2.332833	1.693628
27	1	0	1.467315	2.131111	0.944709
28	1	0	1.014590	3.676007	1.687937
29	1	0	0.745083	1.612976	-1.398279
30	1	0	-0.902374	1.430392	-0.825806
31	1	0	-0.556908	2.520995	-2.172978
32	6	0	-2.375214	8.145103	1.225084
33	6	0	-1.681803	9.155961	0.295939
34	6	0	-3.862197	8.523356	1.321808
35	6	0	-1.743730	8.263668	2.630429
36	1	0	-0.601639	8.982604	0.240415
37	1	0	-2.099084	9.118168	-0.715200
38	1	0	-1.829295	10.169903	0.682418
39	1	0	-4.398767	7.881852	2.027432
40	1	0	-3.958106	9.557214	1.670449
41	1	0	-4.354299	8.443101	0.347134
42	1	0	-1.847246	9.289917	3.001592
43	1	0	-2.239144	7.588093	3.334212
44	1	0	-0.678856	8.012697	2.606765
45	6	0	-0.599459	6.609328	-1.590722
46	1	0	0.291751	6.243110	-2.098522
47	1	0	-1.492788	6.247730	-2.106808

48	1	0	-0.580697	7.698240	-1.625158

2, ωB97x-D/6-31+G(d,p), benzene IEFPCM:
Sum of electronic and thermal Free Energies= -1422.687046

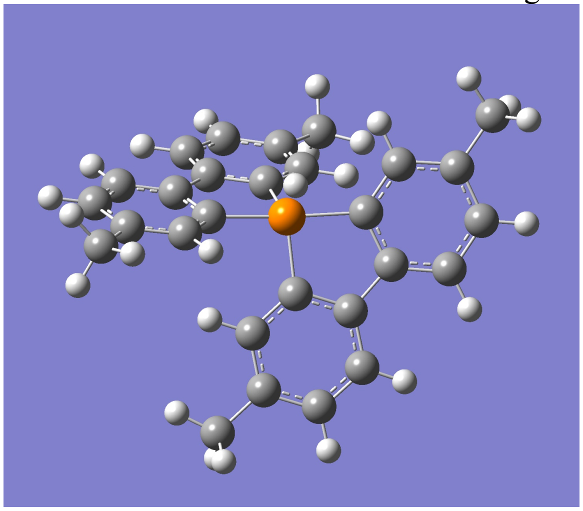

Center Number	Atomic Number	Atomic Type	Coordinates (Angstroms) X	Y	Z
1	6	0	-2.198075	-1.608852	-0.190169
2	6	0	-1.871422	-0.418333	-0.834445
3	6	0	-2.842216	0.261426	-1.563580
4	6	0	-4.156634	-0.210949	-1.628453
5	6	0	-4.468080	-1.403100	-0.958356
6	6	0	-3.501505	-2.107803	-0.248290
7	1	0	-2.583447	1.183275	-2.085756
8	1	0	-5.483303	-1.789425	-1.006618
9	1	0	-3.768667	-3.040654	0.240646
10	6	0	-1.033193	-2.229818	0.473007
11	6	0	-1.047814	-3.376252	1.267442
12	6	0	0.179645	-1.559696	0.257679
13	6	0	0.131105	-3.835437	1.845076
14	1	0	-1.976766	-3.909125	1.447136
15	6	0	1.351658	-2.022220	0.852069
16	6	0	1.345577	-3.167492	1.652498
17	1	0	0.108084	-4.728649	2.464735
18	1	0	2.284426	-1.490159	0.696879
19	6	0	1.033384	2.230277	0.472176
20	6	0	1.048185	3.376953	1.266266
21	6	0	-0.130599	3.836319	1.844015
22	6	0	-1.345116	3.168296	1.651943
23	6	0	-1.351388	2.022798	0.851851
24	6	0	-0.179506	1.560099	0.257320

25	1	0	1.977176	3.909897	1.445549
26	1	0	-0.107454	4.729757	2.463345
27	1	0	-2.284202	1.490719	0.697001
28	6	0	2.198130	1.609144	-0.191085
29	6	0	1.871243	0.418424	-0.835065
30	6	0	3.501404	2.108154	-0.249919
31	6	0	2.841677	-0.261349	-1.564459
32	6	0	4.467810	1.403283	-0.960261
33	1	0	3.768658	3.041318	0.238370
34	6	0	4.156204	0.210974	-1.629786
35	1	0	2.582675	-1.183108	-2.086702
36	1	0	5.482896	1.789841	-1.009241
37	15	0	-0.000075	0.000070	-0.736516
38	6	0	-2.612027	3.679353	2.291268
39	1	0	-2.866119	4.677076	1.918094
40	1	0	-3.455832	3.017078	2.082156
41	1	0	-2.502492	3.754141	3.377969
42	6	0	5.222785	-0.543685	-2.383682
43	1	0	4.783970	-1.293213	-3.047635
44	1	0	5.834014	0.131923	-2.989838
45	1	0	5.896353	-1.063674	-1.693305
46	6	0	2.612737	-3.678579	2.291307
47	1	0	2.869298	-4.674247	1.914314
48	1	0	3.455467	-3.013786	2.085871
49	1	0	2.502074	-3.758070	3.377546
50	6	0	-5.223481	0.541643	-2.384060
51	1	0	-4.786861	1.313542	-3.023314
52	1	0	-5.811268	-0.131066	-3.016243
53	1	0	-5.918850	1.033266	-1.694635
54	1	0	-0.000259	-0.000225	-2.172746

3, ωB97x-D/6-31+G(d,p), benzene IEFPCM:
Sum of electronic and thermal Free Energies= -875.071323

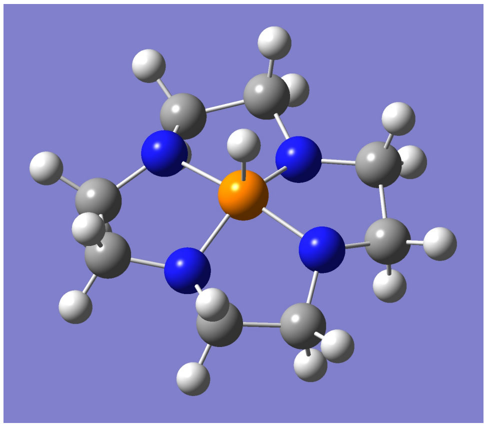

Center Number	Atomic Number	Atomic Type	Coordinates (Angstroms) X	Y	Z
1	6	0	2.907832	3.083927	-0.420038
2	6	0	3.260588	4.526239	-0.053133
3	6	0	1.885464	6.621735	0.183740
4	6	0	0.394158	6.914757	0.012033
5	1	0	3.692644	2.388829	-0.098564
6	1	0	3.753529	4.574219	0.932502
7	1	0	2.228400	6.896974	1.195389
8	1	0	2.487105	7.177940	-0.543118
9	1	0	0.164661	7.029183	-1.063043
10	6	0	-0.572337	1.988297	0.152007
11	6	0	0.862733	1.728768	-0.309192
12	6	0	-1.651482	5.560442	0.122217
13	6	0	-1.947117	4.083541	0.387954
14	1	0	-1.297673	1.506798	-0.512605
15	1	0	-0.734935	1.604101	1.173185
16	1	0	1.220778	0.751827	0.036987
17	1	0	-2.360231	6.208884	0.651017
18	1	0	-2.745585	3.713439	-0.264054
19	15	0	0.693891	4.282227	0.585454
20	1	0	0.818136	4.205702	1.988248
21	7	0	-0.698081	3.424301	0.093725
22	7	0	-0.291284	5.768794	0.569134
23	7	0	1.985653	5.201326	-0.047807
24	7	0	1.646552	2.815820	0.236198
25	1	0	0.898604	1.730327	-1.413828
26	1	0	-2.257410	3.924612	1.434517
27	1	0	-1.748362	5.764637	-0.959644
28	1	0	0.111504	7.846276	0.516819
29	1	0	3.934034	4.972227	-0.792887
30	1	0	2.809006	2.998701	-1.517557

4, ωB97x-D/6-31+G(d,p), benzene IEFPCM:
Sum of electronic and thermal Free Energies= -1114.140322

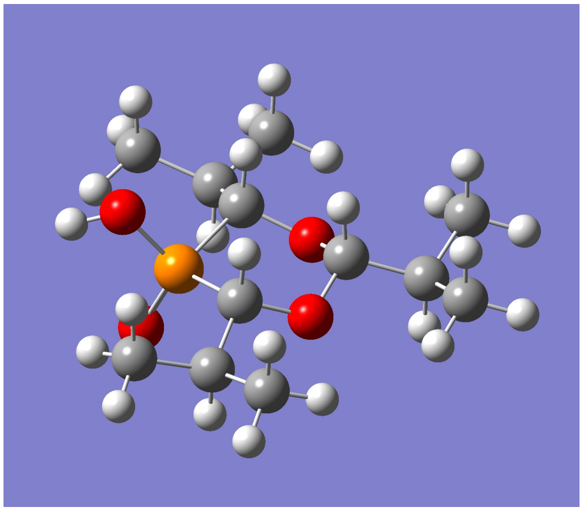

Center Number	Atomic Number	Atomic Type	Coordinates (Angstroms) X	Y	Z
1	6	0	-1.410914	-0.939244	-0.158961
2	6	0	-1.463237	1.561551	1.229771
3	6	0	-3.329316	0.154700	0.745288
4	1	0	-1.300587	-1.523159	0.772250
5	1	0	-1.354634	1.084195	2.220049
6	1	0	-3.086658	-0.366356	1.692288
7	15	0	-0.404167	0.597746	0.058310
8	8	0	-2.813249	1.461084	0.796279
9	8	0	-2.771009	-0.561852	-0.327278
10	8	0	0.037430	1.297400	-1.183947
11	8	0	0.819382	0.092706	1.012980
12	1	0	1.678441	0.317472	0.636598
13	6	0	-1.104660	3.046189	1.336881
14	6	0	0.357840	3.251824	1.742675
15	6	0	-2.046901	3.736595	2.327258
16	1	0	-1.255344	3.481666	0.342122
17	1	0	1.050090	2.908654	0.969225
18	1	0	0.549206	4.316771	1.902268
19	1	0	0.588987	2.724985	2.675465
20	1	0	-3.092523	3.605578	2.041185
21	1	0	-1.911846	3.329687	3.336924
22	1	0	-1.832399	4.808913	2.365570
23	6	0	-4.836956	0.233751	0.547638
24	6	0	-5.483368	1.036812	1.678384
25	6	0	-5.439130	-1.169495	0.450081
26	1	0	-4.996462	0.759109	-0.401967
27	1	0	-5.088683	2.054023	1.723631
28	1	0	-6.565701	1.092713	1.529228
29	1	0	-5.305090	0.556310	2.648303

30	1	0	-5.013601	-1.731114	-0.384178
31	1	0	-5.258684	-1.735667	1.372162
32	1	0	-6.521728	-1.105043	0.306295
33	6	0	-1.002681	-1.803306	-1.355268
34	6	0	-1.906052	-3.037405	-1.435284
35	6	0	0.472139	-2.211366	-1.288085
36	1	0	-1.155552	-1.193783	-2.253653
37	1	0	-2.959739	-2.757286	-1.495302
38	1	0	-1.656344	-3.629595	-2.320906
39	1	0	-1.768267	-3.675339	-0.553607
40	1	0	1.140782	-1.352712	-1.392921
41	1	0	0.701469	-2.718192	-0.343819
42	1	0	0.699782	-2.903103	-2.104099

5, ωB97x-D/6-31+G(d,p), chloroform IEFPCM:
Sum of electronic and thermal Free Energies= -2108.259391

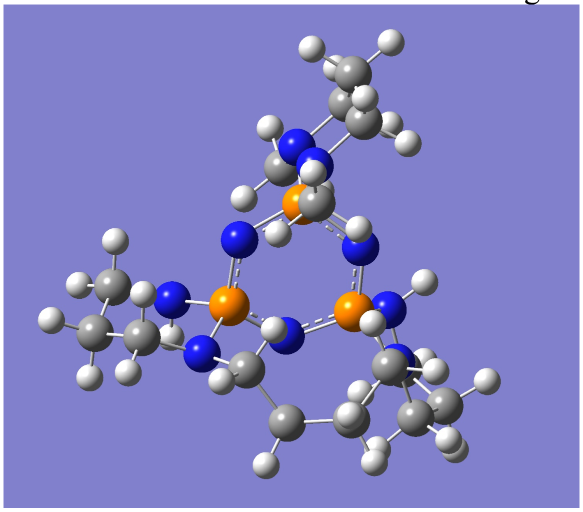

Center Number	Atomic Number	Atomic Type	Coordinates (Angstroms) X	Y	Z
1	15	0	9.555395	9.255480	5.939670
2	15	0	10.107716	8.114041	3.429278
3	15	0	7.491083	8.930131	4.035727
4	7	0	10.592635	8.759640	4.829797
5	7	0	8.535380	8.049253	3.168110
6	7	0	8.061571	9.580241	5.380113
7	7	0	9.491693	8.158481	7.226461
8	7	0	10.174088	10.621851	6.702229
9	7	0	10.742203	6.561999	3.330530
10	1	0	10.285029	6.043341	2.586133
11	7	0	10.872356	8.930661	2.150116
12	7	0	6.775901	10.154993	3.119048

13	7	0	6.193175	7.917806	4.393127
14	1	0	5.677146	8.307745	5.177071
15	6	0	9.046993	6.822604	6.830428
16	1	0	9.244305	6.129384	7.653470
17	1	0	9.608944	6.484548	5.956381
18	1	0	7.972688	6.790721	6.591725
19	6	0	8.813192	8.640738	8.433709
20	1	0	8.882429	7.848668	9.186853
21	1	0	7.740229	8.815508	8.241460
22	6	0	9.471053	9.908311	8.962351
23	1	0	10.500558	9.681434	9.257603
24	1	0	8.930726	10.248713	9.851515
25	6	0	9.482654	11.029583	7.931706
26	1	0	8.453831	11.361680	7.712353
27	1	0	10.022489	11.892349	8.335492
28	6	0	10.460899	11.746924	5.818110
29	1	0	9.547335	12.262688	5.483600
30	1	0	11.015533	11.396511	4.945961
31	1	0	11.087117	12.465521	6.355203
32	6	0	12.208871	6.498797	3.231750
33	1	0	12.500214	5.448403	3.151075
34	1	0	12.618813	6.880281	4.172884
35	6	0	12.753488	7.310150	2.054080
36	1	0	13.847474	7.253560	2.043772
37	1	0	12.388264	6.877778	1.114606
38	6	0	12.336876	8.779042	2.122253
39	1	0	12.803550	9.259859	2.998304
40	1	0	12.702760	9.298607	1.230748
41	6	0	10.394261	10.310015	1.966306
42	1	0	11.200511	10.885039	1.499783
43	1	0	10.217393	10.788411	2.941518
44	6	0	9.129430	10.380495	1.095856
45	1	0	9.421225	10.483631	0.044772
46	1	0	8.602117	9.428419	1.183679
47	6	0	8.176460	11.517123	1.471832
48	1	0	7.396954	11.587782	0.704022
49	1	0	8.703055	12.481035	1.453473
50	6	0	7.512413	11.393115	2.854340
51	1	0	8.252619	11.504560	3.650713
52	1	0	6.810243	12.230458	2.968862
53	6	0	5.789946	9.784156	2.098235
54	1	0	6.274552	9.319009	1.224241
55	1	0	5.322514	10.710997	1.747015
56	6	0	4.706754	8.849188	2.632302
57	1	0	4.044263	8.570515	1.805566
58	1	0	4.103118	9.380335	3.378274

59	6	0	5.302230	7.594418	3.270200
60	1	0	4.515937	6.929191	3.636991
61	1	0	5.884552	7.031480	2.532327

6, ωB97x-D/6-31+G(d,p), chloroform IEFPCM:
Sum of electronic and thermal Free Energies= -922.809824

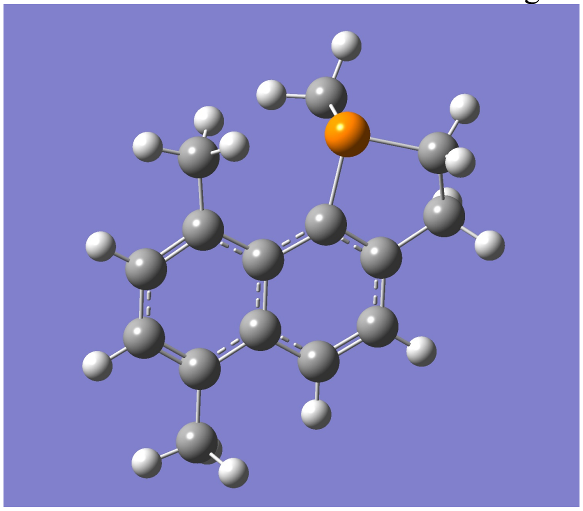

Center Number	Atomic Number	Atomic Type	Coordinates (Angstroms) X	Y	Z
1	6	0	-4.318009	-0.901203	-0.161930
2	6	0	-2.945840	-0.936099	-0.263997
3	6	0	-2.210192	0.297490	-0.142083
4	6	0	-2.956707	1.507808	0.073015
5	6	0	-4.387056	1.492690	0.166371
6	6	0	-5.037109	0.291590	0.050244
7	1	0	-4.873696	-1.830725	-0.251584
8	6	0	-0.777260	0.405506	-0.217805
9	6	0	-2.267675	2.744276	0.194375
10	1	0	-6.120771	0.251450	0.120182
11	6	0	-0.903635	2.814266	0.118221
12	6	0	-0.155281	1.638481	-0.086409
13	1	0	-2.836022	3.654327	0.349083
14	1	0	-0.394687	3.769428	0.214547
15	6	0	-2.305207	-2.279357	-0.514150
16	1	0	-1.756980	-2.303343	-1.458613
17	1	0	-1.602562	-2.561407	0.275290
18	1	0	-3.076902	-3.052415	-0.551901
19	6	0	-5.169907	2.762117	0.391052
20	1	0	-4.890325	3.250714	1.330743
21	1	0	-5.009970	3.485888	-0.415447
22	1	0	-6.239457	2.544090	0.434865

23	6	0	1.354091	1.652100	-0.127835
24	1	0	1.739664	1.729755	0.897189
25	1	0	1.727426	2.528872	-0.667085
26	6	0	1.805749	0.344118	-0.782705
27	1	0	2.780626	-0.001920	-0.427465
28	1	0	1.874450	0.465468	-1.868573
29	15	0	0.496692	-0.932173	-0.452935
30	6	0	0.854366	-1.318789	1.332864
31	1	0	1.750884	-1.943309	1.390087
32	1	0	1.004948	-0.416341	1.933156
33	1	0	0.021714	-1.882626	1.762398

7, ωB97x-D/6-31+G(d,p), chloroform IEFPCM:
Sum of electronic and thermal Free Energies= -2480.90609669

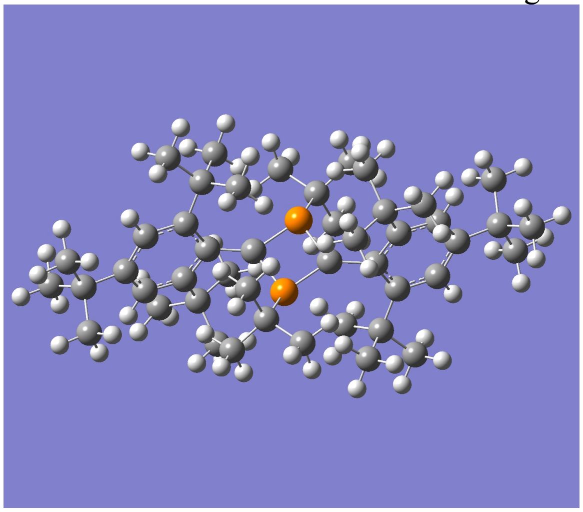

Center Number	Atomic Number	Atomic Type	Coordinates (Angstroms) X	Y	Z
1	6	0	1.155762	-0.005433	0.497184
2	6	0	2.620449	-0.135978	0.692239
3	6	0	3.370441	1.067462	0.870330
4	6	0	4.713728	1.077002	0.481510
5	1	0	5.252908	2.015555	0.514242
6	6	0	5.391446	-0.053178	0.048482
7	6	0	4.704084	-1.259888	0.145356
8	1	0	5.240490	-2.169664	-0.075467
9	6	0	3.351132	-1.352555	0.478862
10	6	0	2.844874	2.395913	1.492605
11	6	0	1.705015	2.177668	2.497180
12	1	0	2.022417	1.502715	3.299719
13	1	0	0.825574	1.753132	2.022487
14	1	0	1.427791	3.135935	2.950284

15	6	0	2.394609	3.402463	0.417773
16	1	0	1.499510	3.057506	-0.104535
17	1	0	3.185569	3.564294	-0.322469
18	1	0	2.163220	4.366787	0.885771
19	6	0	3.977878	3.072111	2.305559
20	1	0	3.552733	3.892529	2.892636
21	1	0	4.758020	3.508535	1.676253
22	1	0	4.448961	2.366416	2.997486
23	6	0	6.835756	0.055129	-0.453081
24	6	0	6.867970	0.996760	-1.673272
25	1	0	6.511519	1.999412	-1.417648
26	1	0	6.233850	0.609034	-2.477822
27	1	0	7.890640	1.090419	-2.055997
28	6	0	7.734768	0.628506	0.659441
29	1	0	7.719888	-0.020131	1.541632
30	1	0	7.410212	1.626570	0.969312
31	1	0	8.769973	0.707092	0.308417
32	6	0	7.408311	-1.303643	-0.880889
33	1	0	8.427661	-1.168636	-1.257148
34	1	0	6.815281	-1.759170	-1.681096
35	1	0	7.455931	-2.007438	-0.043067
36	6	0	2.767504	-2.781515	0.662783
37	6	0	3.861303	-3.869730	0.646648
38	1	0	4.635132	-3.690914	1.400261
39	1	0	4.340901	-3.971380	-0.332121
40	1	0	3.392296	-4.832428	0.875217
41	6	0	2.128687	-2.859456	2.059348
42	1	0	1.319967	-2.143916	2.167558
43	1	0	2.871249	-2.644108	2.835316
44	1	0	1.717368	-3.859236	2.239711
45	6	0	1.755279	-3.194856	-0.423625
46	1	0	1.302944	-4.155619	-0.151173
47	1	0	2.260344	-3.323969	-1.385564
48	1	0	0.950753	-2.474569	-0.552624
49	6	0	1.245790	0.536559	-2.593066
50	6	0	2.376028	1.580596	-2.625786
51	1	0	2.879870	1.523672	-3.598271
52	1	0	3.122790	1.394636	-1.847952
53	1	0	1.993592	2.598186	-2.501281
54	6	0	1.854883	-0.854288	-2.789599
55	1	0	1.095736	-1.638122	-2.806385
56	1	0	2.580102	-1.083903	-2.007055
57	1	0	2.378573	-0.872738	-3.754104
58	6	0	0.248511	0.829423	-3.723276
59	1	0	-0.656883	0.222410	-3.644005
60	1	0	0.730497	0.592220	-4.678989

61	1	0	-0.038908	1.883049	-3.755560
62	15	0	0.390606	0.728422	-0.909176
63	6	0	-1.155760	0.005391	-0.497202
64	6	0	-2.620443	0.135962	-0.692244
65	6	0	-3.370455	-1.067461	-0.870342
66	6	0	-4.713742	-1.076983	-0.481517
67	1	0	-5.252938	-2.015528	-0.514263
68	6	0	-5.391437	0.053202	-0.048470
69	6	0	-4.704052	1.259902	-0.145326
70	1	0	-5.240440	2.169684	0.075518
71	6	0	-3.351101	1.352551	-0.478840
72	6	0	-2.844926	-2.395914	-1.492643
73	6	0	-1.704953	-2.177725	-2.497101
74	1	0	-2.022188	-1.502662	-3.299611
75	1	0	-0.825496	-1.753351	-2.022287
76	1	0	-1.427814	-3.135992	-2.950260
77	6	0	-2.394832	-3.402549	-0.417819
78	1	0	-1.499762	-3.057670	0.104593
79	1	0	-3.185868	-3.564376	0.322342
80	1	0	-2.163452	-4.366861	-0.885845
81	6	0	-3.977919	-3.071966	-2.305732
82	1	0	-3.552816	-3.892443	-2.892757
83	1	0	-4.758201	-3.508273	-1.676518
84	1	0	-4.448819	-2.366206	-2.997717
85	6	0	-6.835747	-0.055085	0.453097
86	6	0	-6.867977	-0.996747	1.673265
87	1	0	-6.511551	-1.999401	1.417614
88	1	0	-6.233845	-0.609055	2.477822
89	1	0	-7.890648	-1.090392	2.055992
90	6	0	-7.734776	-0.628414	-0.659436
91	1	0	-7.719884	0.020246	-1.541611
92	1	0	-7.410242	-1.626477	-0.969334
93	1	0	-8.769981	-0.706987	-0.308410
94	6	0	-7.408270	1.303688	0.880942
95	1	0	-8.427621	1.168694	1.257203
96	1	0	-6.815226	1.759183	1.681157
97	1	0	-7.455880	2.007505	0.043138
98	6	0	-2.767448	2.781503	-0.662747
99	6	0	-3.861228	3.869737	-0.646570
100	1	0	-4.635076	3.690949	-1.400170
101	1	0	-4.340804	3.971378	0.332211
102	1	0	-3.392209	4.832431	-0.875132
103	6	0	-2.128661	2.859453	-2.059325
104	1	0	-1.319958	2.143897	-2.167562
105	1	0	-2.871244	2.644132	-2.835280
106	1	0	-1.717326	3.859227	-2.239681

107	6	0	-1.755190	3.194818	0.423641
108	1	0	-1.302872	4.155592	0.151199
109	1	0	-2.260224	3.323904	1.385600
110	1	0	-0.950650	2.474537	0.552595
111	6	0	-1.245800	-0.536605	2.593045
112	6	0	-2.375999	-1.580686	2.625781
113	1	0	-2.879839	-1.523773	3.598267
114	1	0	-3.122771	-1.394763	1.847948
115	1	0	-1.993522	-2.598262	2.501285
116	6	0	-1.854949	0.854220	2.789557
117	1	0	-1.095831	1.638081	2.806364
118	1	0	-2.580155	1.083810	2.006993
119	1	0	-2.378665	0.872654	3.754048
120	6	0	-0.248513	-0.829412	3.723261
121	1	0	0.656861	-0.222370	3.643971
122	1	0	-0.730504	-0.592200	4.678970
123	1	0	0.038940	-1.883028	3.755571
124	15	0	-0.390607	-0.728487	0.909159

8, ωB97x-D/6-31+G(d,p), chloroform IEFPCM:
Sum of electronic and thermal Free Energies= -1381.380631

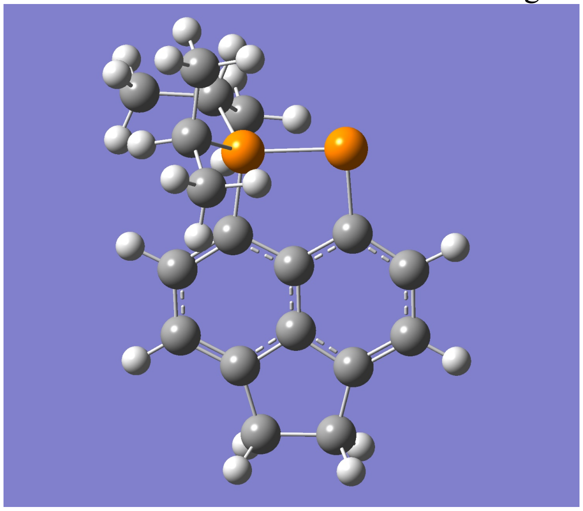

Center Number	Atomic Number	Atomic Type	Coordinates (Angstroms) X	Y	Z
1	15	0	8.561050	0.066980	12.004782
2	15	0	9.220157	-1.689717	10.950344
3	6	0	6.824096	0.128765	11.506610
4	6	0	5.815405	1.005250	11.868325
5	6	0	4.500332	0.872055	11.342143
6	6	0	4.225538	-0.150739	10.458247
7	6	0	5.256574	-1.039243	10.108525

8	6	0	4.853336	-2.050926	9.216065
9	6	0	5.802681	-2.969918	8.837530
10	6	0	7.134693	-2.878592	9.347608
11	6	0	7.540510	-1.883949	10.228709
12	6	0	6.549751	-0.931107	10.610424
13	6	0	2.967616	-0.578679	9.726885
14	6	0	3.383685	-1.843532	8.898468
15	6	0	8.645236	-0.005792	13.850014
16	6	0	7.807004	-1.170867	14.380576
17	6	0	10.102179	-0.092524	14.317427
18	6	0	9.411469	1.632439	11.486276
19	6	0	9.066078	2.843502	12.358483
20	6	0	9.119998	1.906785	10.007466
21	1	0	6.009858	1.809952	12.571537
22	1	0	3.734884	1.578389	11.649188
23	1	0	5.564215	-3.779750	8.152921
24	1	0	7.850730	-3.630523	9.025531
25	1	0	2.163446	-0.808436	10.432724
26	1	0	2.600742	0.222568	9.077912
27	1	0	2.785366	-2.714903	9.182432
28	1	0	3.223655	-1.683133	7.827663
29	1	0	8.203634	0.934662	14.202351
30	1	0	7.850592	-1.180043	15.474167
31	1	0	8.192823	-2.125954	14.011041
32	1	0	6.758741	-1.086356	14.080889
33	1	0	10.574857	-1.006322	13.941406
34	1	0	10.135592	-0.124063	15.410577
35	1	0	10.699684	0.763822	13.990624
36	1	0	10.479364	1.402932	11.594012
37	1	0	7.995899	3.071310	12.318513
38	1	0	9.599540	3.721145	11.980437
39	1	0	9.354034	2.708840	13.404161
40	1	0	9.739903	2.739435	9.661812
41	1	0	8.069695	2.180138	9.861856
42	1	0	9.338360	1.032800	9.387846

9, ωB97x-D/6-31+G(d,p), chloroform IEFPCM:
Sum of electronic and thermal Free Energies= -3370.242775

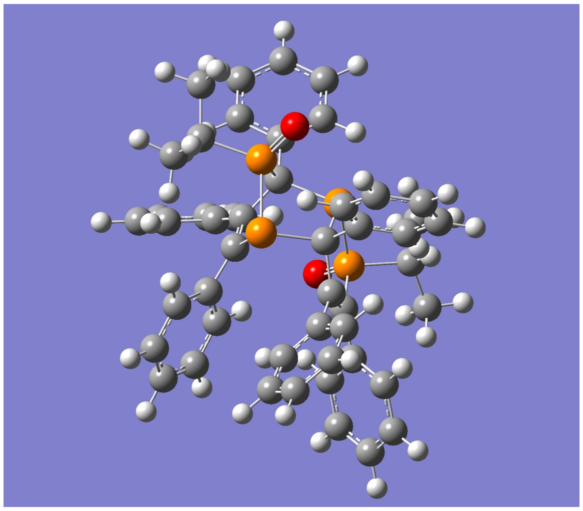

Center Number	Atomic Number	Atomic Type	Coordinates (Angstroms) X	Y	Z
1	15	0	1.017819	15.103574	4.158459
2	15	0	-0.248370	16.879914	3.751872
3	8	0	0.326485	18.094317	3.066329
4	6	0	-0.749337	17.157891	5.475929
5	6	0	-0.155552	16.397263	6.416298
6	6	0	0.832823	15.284103	6.038314
7	15	0	2.510541	15.789586	6.807369
8	15	0	3.638255	14.644068	5.292842
9	8	0	3.389449	13.165160	5.205250
10	6	0	2.836949	15.681662	3.929476
11	6	0	3.068413	17.091662	4.455177
12	6	0	2.838526	17.280152	5.783065
13	6	0	-1.682656	16.181213	2.832963
14	1	0	-2.030581	15.319138	3.417395
15	6	0	-2.820366	17.203548	2.706875
16	1	0	-3.620434	16.775941	2.094247
17	1	0	-2.468958	18.116692	2.216962
18	1	0	-3.244364	17.476784	3.675335
19	6	0	-1.220012	15.699420	1.451916
20	1	0	-2.070865	15.279166	0.907312
21	1	0	-0.453933	14.921910	1.521242
22	1	0	-0.817350	16.530717	0.865279
23	6	0	-1.756466	18.213503	5.759514
24	6	0	-2.903122	17.910249	6.504557
25	1	0	-3.040297	16.903683	6.887579
26	6	0	-3.862710	18.885260	6.754482
27	1	0	-4.745316	18.634755	7.334639
28	6	0	-3.691903	20.178573	6.260288
29	1	0	-4.440125	20.940228	6.456014

30	6	0	-2.558764	20.485478	5.510030
31	1	0	-2.420172	21.488025	5.117393
32	6	0	-1.598468	19.509109	5.255442
33	1	0	-0.725918	19.746741	4.657246
34	6	0	-0.365438	16.674426	7.875205
35	6	0	-0.912125	15.744814	8.768095
36	1	0	-1.228413	14.769250	8.420735
37	6	0	-1.082770	16.071878	10.111601
38	1	0	-1.516038	15.339967	10.786114
39	6	0	-0.705505	17.325443	10.587571
40	1	0	-0.835662	17.573918	11.636234
41	6	0	-0.167218	18.259459	9.704906
42	1	0	0.127144	19.243282	10.056536
43	6	0	-0.006530	17.938713	8.361481
44	1	0	0.399473	18.678329	7.680502
45	6	0	0.316320	13.941772	6.575730
46	6	0	-0.946088	13.501354	6.157012
47	1	0	-1.522047	14.101145	5.457213
48	6	0	-1.492028	12.319350	6.641717
49	1	0	-2.474472	12.003221	6.305532
50	6	0	-0.779329	11.546299	7.559158
51	1	0	-1.201869	10.623014	7.942250
52	6	0	0.479961	11.967642	7.972498
53	1	0	1.049569	11.373640	8.679973
54	6	0	1.022868	13.155690	7.483429
55	1	0	2.004701	13.458049	7.830730
56	6	0	5.430178	15.084872	5.434595
57	1	0	5.563770	16.064039	4.964746
58	6	0	6.270231	14.034521	4.697094
59	1	0	7.322784	14.332803	4.731410
60	1	0	5.978906	13.928017	3.649913
61	1	0	6.171707	13.056812	5.175928
62	6	0	5.847294	15.195015	6.907656
63	1	0	6.927749	15.359352	6.963083
64	1	0	5.620193	14.276519	7.458771
65	1	0	5.356225	16.030404	7.414552
66	6	0	3.396302	15.314533	2.558252
67	6	0	4.683288	15.726011	2.179263
68	1	0	5.275666	16.348890	2.837641
69	6	0	5.228303	15.365013	0.953034
70	1	0	6.225398	15.706972	0.694286
71	6	0	4.501867	14.576044	0.063557
72	1	0	4.924294	14.297556	-0.896523
73	6	0	3.229915	14.147853	0.426429
74	1	0	2.648101	13.525444	-0.245915
75	6	0	2.688756	14.510025	1.658892

76	1	0	1.703118	14.141338	1.916430
77	6	0	3.649397	18.159192	3.595073
78	6	0	4.787901	18.834263	4.055778
79	1	0	5.186352	18.601825	5.039042
80	6	0	5.416716	19.792827	3.267787
81	1	0	6.298812	20.303024	3.641906
82	6	0	4.913871	20.091988	2.002599
83	1	0	5.401204	20.839385	1.384142
84	6	0	3.783766	19.422696	1.535369
85	1	0	3.385485	19.648887	0.551209
86	6	0	3.160644	18.457551	2.320091
87	1	0	2.274631	17.951903	1.958643
88	6	0	2.777262	18.605721	6.441660
89	6	0	3.343780	18.829262	7.700787
90	1	0	3.892687	18.033216	8.196051
91	6	0	3.195670	20.059635	8.335943
92	1	0	3.640207	20.216557	9.313743
93	6	0	2.468181	21.079965	7.725725
94	1	0	2.341473	22.034491	8.226749
95	6	0	1.897082	20.864591	6.471021
96	1	0	1.318408	21.649024	5.993043
97	6	0	2.051026	19.638891	5.833048
98	1	0	1.590271	19.458835	4.865787

10, ωB97x-D/6-31+G(d,p), chloroform IEFPCM:
Sum of electronic and thermal Free Energies= -1949.797435

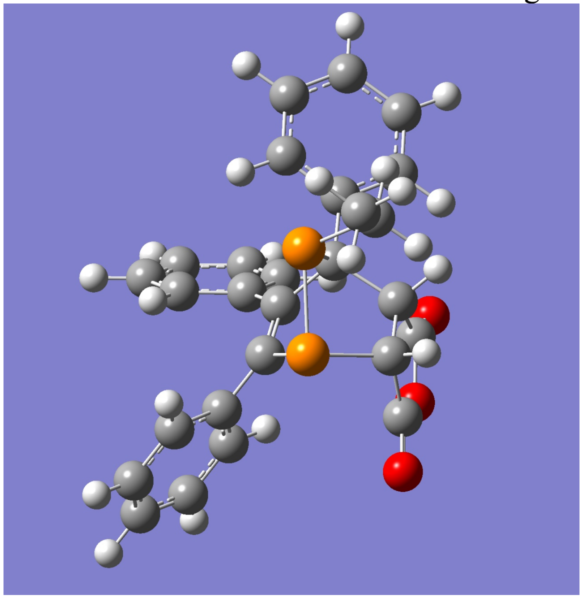

(mirror image of coordinates below shown for clarity)

Center Number	Atomic Number	Atomic Type	Coordinates (Angstroms) X	Y	Z
1	15	0	13.106363	8.802172	14.893267
2	15	0	13.086519	10.595895	13.607756
3	8	0	14.243924	10.113678	18.238176
4	8	0	14.485247	7.926516	17.809944
5	8	0	14.212293	12.343873	18.156229
6	6	0	13.096413	11.566910	15.248532
7	6	0	11.831468	10.981740	15.900208
8	6	0	11.746582	9.629230	15.850133
9	6	0	14.491718	9.537640	15.966921
10	6	0	14.416305	9.042829	17.383764
11	6	0	14.257058	11.307254	17.557066
12	6	0	14.346722	11.060618	16.060137
13	6	0	13.065481	13.058130	14.957798
14	6	0	14.046961	13.947614	15.399727
15	1	0	14.861032	13.610239	16.029275
16	6	0	13.981233	15.304441	15.079561
17	1	0	14.754678	15.973903	15.442589
18	6	0	12.930799	15.798040	14.314247
19	1	0	12.877923	16.854300	14.070599
20	6	0	11.942804	14.920699	13.866945
21	1	0	11.112999	15.288419	13.271801
22	6	0	12.013725	13.569960	14.182391
23	1	0	11.233067	12.903415	13.828744
24	6	0	10.775369	11.852044	16.481575
25	6	0	11.065946	12.892883	17.370164
26	1	0	12.090182	13.086235	17.667383
27	6	0	10.045601	13.690652	17.880614
28	1	0	10.288594	14.488939	18.574734
29	6	0	8.723360	13.472924	17.498756
30	1	0	7.930476	14.100383	17.893552
31	6	0	8.426293	12.449826	16.599492
32	1	0	7.401322	12.277264	16.286623
33	6	0	9.444317	11.647055	16.096047
34	1	0	9.209471	10.855088	15.391768
35	6	0	10.739339	8.791217	16.542092
36	6	0	10.454749	8.992241	17.898808
37	1	0	10.975542	9.772675	18.444820
38	6	0	9.512608	8.201371	18.548393
39	1	0	9.305781	8.368467	19.600711
40	6	0	8.840756	7.196764	17.852936
41	1	0	8.105142	6.580555	18.360154
42	6	0	9.125725	6.980807	16.505582

43	1	0	8.611342	6.197372	15.958010
44	6	0	10.074770	7.766957	15.857549
45	1	0	10.293095	7.591238	14.807625
46	6	0	14.812551	10.778131	12.946000
47	1	0	14.922057	11.841881	12.702781
48	1	0	15.581347	10.540978	13.687300
49	6	0	15.015723	9.924097	11.689967
50	1	0	14.919576	8.856791	11.913417
51	1	0	15.446714	9.214676	15.544344
52	1	0	15.243113	11.568258	15.695742
53	1	0	16.014656	10.089195	11.277024
54	1	0	14.285052	10.173704	10.915052

----

11 isomer A, ωB97x-D/6-31+G(d,p), toluene IEFPCM:
Sum of electronic and thermal Free Energies= -1091.405142

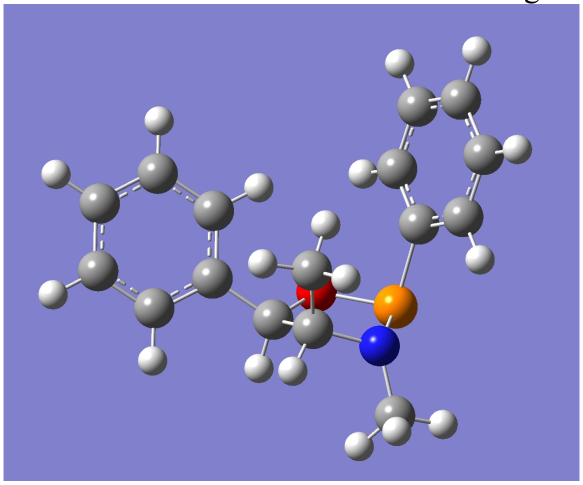

Center Number	Atomic Number	Atomic Type	Coordinates (Angstroms) X	Y	Z
1	6	0	0.450206	1.587308	0.722873
2	6	0	1.203333	1.077388	-0.545215
3	1	0	1.039174	2.401102	1.159789
4	1	0	1.702343	1.940704	-1.007559
5	15	0	-1.299615	1.288228	-1.177343
6	8	0	0.214640	0.606783	-1.466768
7	7	0	-0.804456	2.167653	0.208036
8	6	0	-0.804955	3.626926	0.098130
9	1	0	-1.727308	3.958363	-0.383410
10	1	0	-0.772190	4.065434	1.100074
11	1	0	0.045343	4.017897	-0.481325
12	6	0	2.235765	0.012955	-0.274999
13	6	0	1.913404	-1.344286	-0.315988

14	6	0	3.530842	0.394322	0.081631
15	6	0	2.870043	-2.304162	0.004394
16	1	0	0.909281	-1.640905	-0.599516
17	6	0	4.487706	-0.564246	0.408246
18	1	0	3.794574	1.449198	0.103007
19	6	0	4.158801	-1.918015	0.370606
20	1	0	2.608253	-3.357239	-0.032175
21	1	0	5.491025	-0.254028	0.683557
22	1	0	4.903699	-2.667589	0.618915
23	6	0	0.217786	0.533808	1.803302
24	1	0	-0.262386	-0.363540	1.408032
25	1	0	1.168563	0.237650	2.254924
26	1	0	-0.428136	0.952659	2.579077
27	6	0	-2.129232	-0.173794	-0.417679
28	6	0	-3.041967	-0.005461	0.627548
29	6	0	-1.934734	-1.450662	-0.953549
30	6	0	-3.726290	-1.100630	1.149716
31	1	0	-3.191899	0.982216	1.054967
32	6	0	-2.617276	-2.547210	-0.430162
33	1	0	-1.234626	-1.591778	-1.772625
34	6	0	-3.512968	-2.374425	0.623943
35	1	0	-4.421565	-0.961147	1.972014
36	1	0	-2.449270	-3.536343	-0.845364
37	1	0	-4.044021	-3.228421	1.032894

----

11 isomer B, ωB97x-D/6-31+G(d,p), toluene IEFPCM:
Sum of electronic and thermal Free Energies= -1091.404765

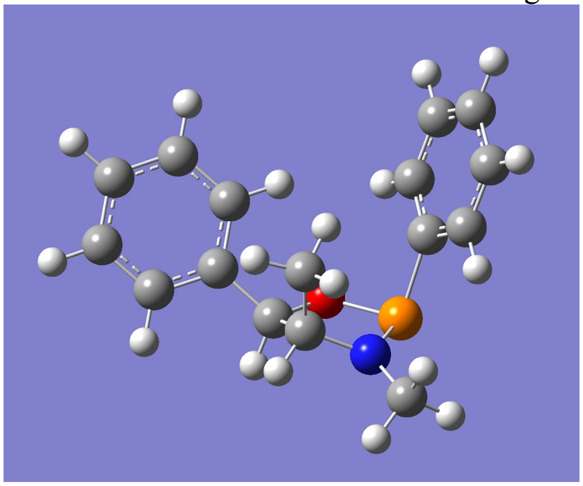

Center Number	Atomic Number	Atomic Type	Coordinates (Angstroms) X	Y	Z
1	6	0	0.367516	1.455296	0.952601

2	6	0	1.120228	1.067532	-0.351846
3	1	0	0.929613	2.268431	1.430846
4	1	0	1.617391	1.971826	-0.724892
5	15	0	-1.404093	1.245458	-0.977172
6	8	0	0.137777	0.692956	-1.329140
7	7	0	-0.873423	2.017424	0.435776
8	6	0	2.150926	-0.020265	-0.184113
9	6	0	1.824027	-1.369332	-0.328444
10	6	0	3.452552	0.328708	0.181729
11	6	0	2.782906	-2.353503	-0.102296
12	1	0	0.815057	-1.642397	-0.617966
13	6	0	4.411891	-0.654536	0.414645
14	1	0	3.719569	1.377968	0.283707
15	6	0	4.078459	-2.000192	0.272947
16	1	0	2.517225	-3.399748	-0.219071
17	1	0	5.420385	-0.369178	0.697773
18	1	0	4.825083	-2.768577	0.447838
19	6	0	0.179337	0.331233	1.973764
20	1	0	-0.277458	-0.553636	1.525132
21	1	0	1.140117	0.038933	2.407043
22	1	0	-0.471295	0.678036	2.782546
23	6	0	-2.138295	-0.365847	-0.410603
24	6	0	-3.041123	-0.384196	0.657208
25	6	0	-1.887463	-1.554742	-1.102752
26	6	0	-3.656457	-1.571461	1.048103
27	1	0	-3.251996	0.530633	1.206113
28	6	0	-2.500654	-2.744627	-0.713739
29	1	0	-1.198200	-1.554447	-1.943234
30	6	0	-3.384041	-2.755920	0.364604
31	1	0	-4.344436	-1.573851	1.888205
32	1	0	-2.288229	-3.663298	-1.252489
33	1	0	-3.860652	-3.682537	0.669126
34	6	0	-1.620100	2.986413	1.203848
35	1	0	-2.418728	3.405031	0.584372
36	1	0	-2.080241	2.555732	2.104763
37	1	0	-0.965185	3.809260	1.515981

----

11 isomer C, ωB97x-D/6-31+G(d,p), toluene IEFPCM:
Sum of electronic and thermal Free Energies= -1091.402038

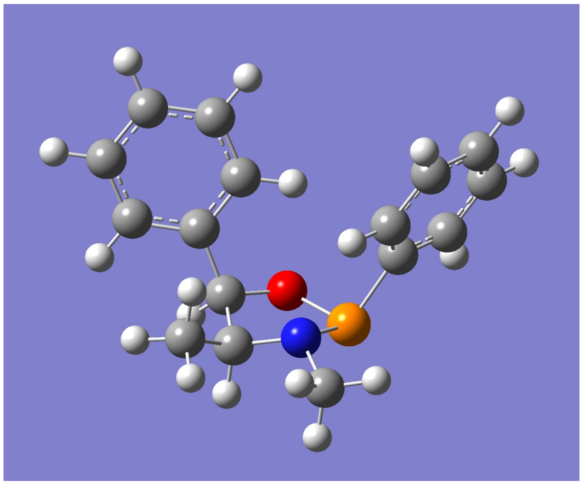

Center Number	Atomic Number	Atomic Type	Coordinates (Angstroms) X	Y	Z
1	6	0	0.326431	1.349640	0.351891
2	6	0	1.177553	0.123003	-0.077266
3	1	0	1.911721	0.464885	-0.815115
4	15	0	-1.338913	-0.407144	-0.580725
5	8	0	0.287511	-0.761292	-0.765489
6	7	0	-1.006854	0.807838	0.614886
7	6	0	1.935189	-0.581187	1.029852
8	6	0	3.222416	-0.140422	1.351597
9	6	0	1.395472	-1.651939	1.741568
10	6	0	3.941917	-0.730742	2.387150
11	1	0	3.670036	0.672908	0.785147
12	6	0	2.114183	-2.248863	2.775573
13	1	0	0.417138	-2.032795	1.477566
14	6	0	3.385889	-1.786822	3.107586
15	1	0	4.939569	-0.373614	2.623657
16	1	0	1.678130	-3.082411	3.317925
17	1	0	3.945601	-2.254098	3.911908
18	1	0	0.289393	2.032521	-0.518293
19	6	0	0.880724	2.127195	1.537318
20	1	0	0.877805	1.511721	2.440789
21	1	0	1.907763	2.445799	1.337236
22	1	0	0.288519	3.027941	1.718822
23	6	0	-2.056786	1.798851	0.810682
24	1	0	-3.025226	1.297788	0.895442
25	1	0	-1.890916	2.341893	1.745134
26	1	0	-2.114000	2.527492	-0.014657
27	6	0	-1.985058	-1.758627	0.497058
28	6	0	-2.459504	-2.912281	-0.137263
29	6	0	-2.074277	-1.661740	1.889790
30	6	0	-2.979079	-3.968483	0.608398

31	1	0	-2.420239	-2.988874	-1.221829
32	6	0	-2.611529	-2.707925	2.635554
33	1	0	-1.702784	-0.765424	2.378124
34	6	0	-3.057388	-3.864900	1.996392
35	1	0	-3.330722	-4.865266	0.107874
36	1	0	-2.677353	-2.624100	3.716189
37	1	0	-3.471465	-4.682139	2.579104

----

12 isomer A, ωB97x-D/6-31+G(d,p), toluene IEFPCM:
Sum of electronic and thermal Free Energies= -1091.407394

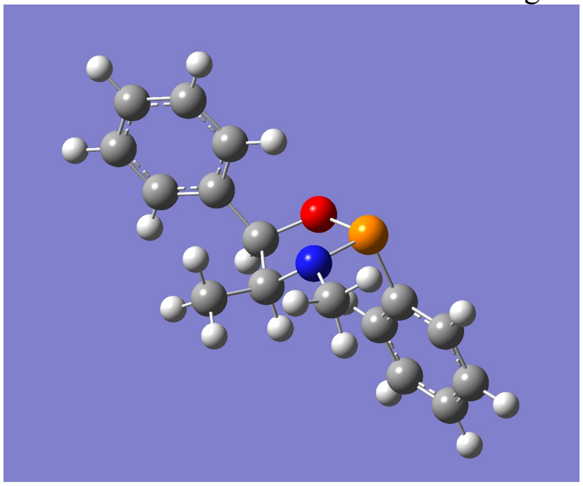

Center Number	Atomic Number	Atomic Type	Coordinates (Angstroms) X	Y	Z
1	6	0	-0.296756	0.516928	0.967290
2	6	0	-0.944828	-0.702323	0.258778
3	1	0	0.559639	0.143669	1.557559
4	1	0	-0.806262	-1.591361	0.884545
5	15	0	0.867817	0.294734	-1.333383
6	8	0	-0.213796	-0.912218	-0.962647
7	7	0	0.183270	1.339557	-0.143278
8	6	0	0.906374	2.550523	0.205876
9	1	0	1.270111	3.032978	-0.706188
10	1	0	0.239154	3.254957	0.709617
11	1	0	1.769465	2.354002	0.862670
12	6	0	2.394730	-0.350870	-0.500730
13	6	0	3.566298	0.402036	-0.642424
14	6	0	2.437948	-1.531012	0.248913
15	6	0	4.748790	0.004126	-0.021794
16	1	0	3.559760	1.307266	-1.247116
17	6	0	3.621744	-1.938030	0.861241
18	1	0	1.544360	-2.141301	0.339585

19	6	0	4.776857	-1.167831	0.732455
20	1	0	5.648609	0.600894	-0.135483
21	1	0	3.643898	-2.858274	1.437222
22	1	0	5.698687	-1.485588	1.209790
23	6	0	-2.418724	-0.549301	-0.042903
24	6	0	-2.866177	0.233008	-1.110428
25	6	0	-3.357958	-1.167667	0.783317
26	6	0	-4.229090	0.399158	-1.338570
27	1	0	-2.144119	0.711880	-1.763169
28	6	0	-4.723754	-0.999771	0.560220
29	1	0	-3.020643	-1.788688	1.609886
30	6	0	-5.162511	-0.213768	-0.502408
31	1	0	-4.564402	1.008166	-2.172495
32	1	0	-5.441974	-1.489301	1.210735
33	1	0	-6.225057	-0.084576	-0.683582
34	6	0	-1.230926	1.265308	1.908535
35	1	0	-2.039488	1.750270	1.355380
36	1	0	-1.674456	0.569999	2.627481
37	1	0	-0.682983	2.022022	2.476113

----

12 isomer B, ωB97x-D/6-31+G(d,p), toluene IEFPCM:
Sum of electronic and thermal Free Energies= -1091.406614

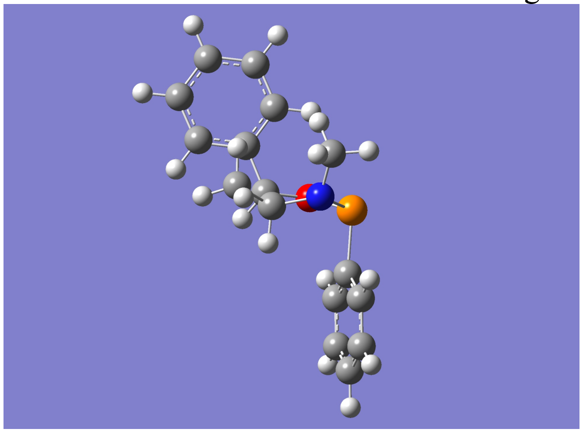

Center Number	Atomic Number	Atomic Type	Coordinates (Angstroms) X	Y	Z
1	6	0	-0.230167	0.621237	0.760283
2	6	0	-0.912492	-0.663670	0.162179
3	1	0	0.656162	0.262697	1.295718
4	1	0	-0.705643	-1.501674	0.838635
5	15	0	0.841456	0.264110	-1.519252
6	8	0	-0.263417	-0.929863	-1.085920
7	7	0	0.276041	1.403391	-0.381186
8	6	0	2.326267	-0.363871	-0.615025

9	6	0	3.348693	0.526757	-0.270119
10	6	0	2.490117	-1.725363	-0.345491
11	6	0	4.506660	0.066345	0.351582
12	1	0	3.231442	1.588560	-0.471514
13	6	0	3.650757	-2.187538	0.274185
14	1	0	1.699908	-2.421855	-0.610444
15	6	0	4.659909	-1.293043	0.624762
16	1	0	5.289409	0.767806	0.624043
17	1	0	3.764683	-3.246565	0.485266
18	1	0	5.563301	-1.652659	1.107696
19	6	0	-2.409154	-0.566969	-0.046954
20	6	0	-2.953690	-0.146641	-1.261314
21	6	0	-3.273003	-0.884587	1.004262
22	6	0	-4.332781	-0.018109	-1.412375
23	1	0	-2.293102	0.063975	-2.096115
24	6	0	-4.651940	-0.755699	0.857980
25	1	0	-2.864183	-1.237829	1.947909
26	6	0	-5.186636	-0.316406	-0.352030
27	1	0	-4.741089	0.308785	-2.363872
28	1	0	-5.308450	-1.006461	1.685523
29	1	0	-6.261198	-0.219222	-0.471258
30	6	0	-1.074060	1.415986	1.743013
31	1	0	-1.998149	1.788982	1.294931
32	1	0	-1.348642	0.781098	2.590010
33	1	0	-0.498630	2.261630	2.129000
34	6	0	-0.538496	2.513393	-0.881568
35	1	0	-0.551541	3.322487	-0.146412
36	1	0	-0.077442	2.900685	-1.792554
37	1	0	-1.575188	2.235067	-1.109512

13, ωB97x-D/6-31+G(d,p), chloroform IEFPCM:
Sum of electronic and thermal Free Energies= -1792.535955

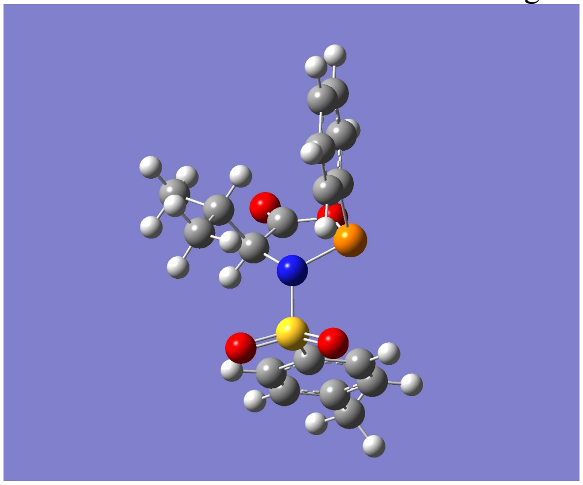

Center Number	Atomic Number	Atomic Type	Coordinates (Angstroms) X	Y	Z
1	15	0	0.698512	-1.375790	-0.371357
2	16	0	-0.961821	-0.241384	1.769407
3	7	0	0.278112	-0.024669	0.652335
4	8	0	0.330921	-0.481472	-1.773226
5	8	0	-0.089535	1.595017	-2.480132
6	8	0	-0.697385	-1.541723	2.370110
7	8	0	-1.002222	0.968321	2.577619
8	6	0	0.164810	0.850840	-1.570671
9	6	0	0.339195	1.240218	-0.098092
10	6	0	1.648225	2.043067	0.140949
11	6	0	1.952176	2.143972	1.634795
12	6	0	1.541635	3.434630	-0.488697
13	6	0	-5.962146	-0.519412	-1.654300
14	6	0	3.192598	-1.276618	0.854446
15	6	0	4.579156	-1.167978	0.896860
16	6	0	5.300366	-0.971076	-0.280984
17	6	0	4.633573	-0.888216	-1.501628
18	6	0	3.244636	-0.994921	-1.549045
19	6	0	2.520183	-1.174977	-0.367937
20	6	0	-3.200121	0.828904	0.602531
21	6	0	-4.334475	0.761458	-0.201165
22	6	0	-4.739790	-0.445024	-0.778354
23	6	0	-3.984641	-1.598130	-0.523920
24	6	0	-2.849530	-1.552636	0.273150
25	6	0	-2.463599	-0.330884	0.826931
26	1	0	-0.507932	1.875050	0.181498
27	1	0	2.470543	1.502363	-0.341166
28	1	0	2.871484	2.718703	1.780428
29	1	0	2.088961	1.158491	2.084850
30	1	0	1.139355	2.648888	2.166704
31	1	0	2.475948	3.980487	-0.330120
32	1	0	0.733801	4.008849	-0.020036
33	1	0	1.349304	3.390714	-1.562580
34	1	0	-6.465579	0.447313	-1.721516
35	1	0	-6.675242	-1.251646	-1.263705
36	1	0	-5.690823	-0.834104	-2.666836
37	1	0	2.634762	-1.417519	1.776781
38	1	0	5.095835	-1.232275	1.848909
39	1	0	6.381562	-0.883753	-0.246294
40	1	0	5.192595	-0.736118	-2.419238
41	1	0	2.729339	-0.916863	-2.501292
42	1	0	-2.902624	1.765440	1.061397

43	1	0	-4.913112	1.663155	-0.376486
44	1	0	-4.293438	-2.546225	-0.954271
45	1	0	-2.276063	-2.451360	0.472993

----

14 isomer A, ωB97x-D/6-31+G(d,p), chloroform IEFPCM:
Sum of electronic and thermal Free Energies= -1792.534008

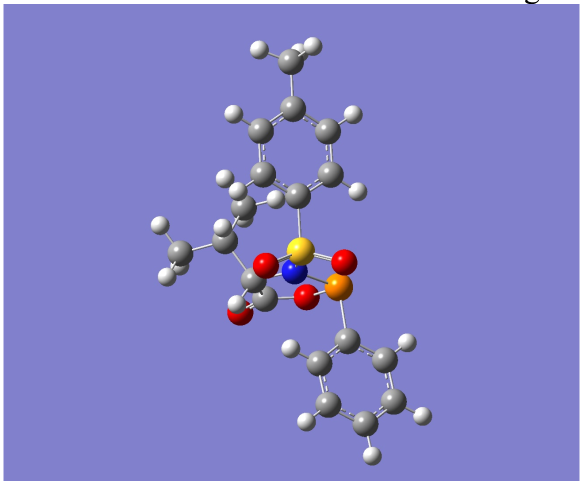

Center Number	Atomic Number	Atomic Type	Coordinates (Angstroms) X	Y	Z
1	15	0	-1.299004	-0.338061	-1.244643
2	16	0	0.469994	-1.013749	1.008128
3	7	0	-0.322936	0.144562	0.128333
4	8	0	-1.562245	1.314537	-1.585368
5	8	0	-1.588706	3.341159	-0.652465
6	8	0	0.448633	-0.610609	2.407844
7	8	0	-0.152705	-2.259928	0.566442
8	6	0	-1.241542	2.190616	-0.609081
9	6	0	-0.368950	1.568182	0.480618
10	6	0	1.023353	2.241744	0.551919
11	6	0	0.936320	3.645767	1.153740
12	6	0	1.741482	2.240083	-0.797488
13	6	0	6.202185	-0.969333	-0.978746
14	6	0	2.439455	-1.507062	-0.823370
15	6	0	3.749835	-1.476071	-1.277598
16	6	0	4.783922	-0.976643	-0.473277
17	6	0	4.470354	-0.509866	0.805868
18	6	0	3.160739	-0.529683	1.277904
19	6	0	2.156455	-1.021749	0.453411
20	1	0	-0.882610	1.709311	1.438813
21	1	0	1.600310	1.631500	1.253758
22	1	0	1.945771	4.039517	1.303593

23	1	0	0.431279	3.632173	2.124768
24	1	0	0.394693	4.330503	0.496783
25	1	0	2.771520	2.584693	-0.671147
26	1	0	1.249501	2.913767	-1.507987
27	1	0	1.777941	1.239415	-1.237895
28	1	0	6.607427	-1.986500	-0.993011
29	1	0	6.848757	-0.358014	-0.345408
30	1	0	6.252161	-0.582077	-2.000228
31	1	0	1.648068	-1.900876	-1.452873
32	1	0	3.976376	-1.847868	-2.272490
33	1	0	5.258275	-0.125804	1.446595
34	1	0	2.922923	-0.169264	2.272801
35	6	0	-2.913110	-0.735951	-0.481043
36	6	0	-3.856678	-1.344774	-1.315239
37	6	0	-3.242966	-0.466907	0.851828
38	6	0	-5.125661	-1.656923	-0.831338
39	1	0	-3.602987	-1.579760	-2.346240
40	6	0	-4.506856	-0.786472	1.336460
41	1	0	-2.510414	-0.027341	1.521808
42	6	0	-5.450109	-1.376231	0.493894
43	1	0	-5.854158	-2.125614	-1.484756
44	1	0	-4.755075	-0.580780	2.372582
45	1	0	-6.435163	-1.625473	0.875412

----

14 isomer B, ωB97x-D/6-31+G(d,p), chloroform IEFPCM:
Sum of electronic and thermal Free Energies= -1792.534340

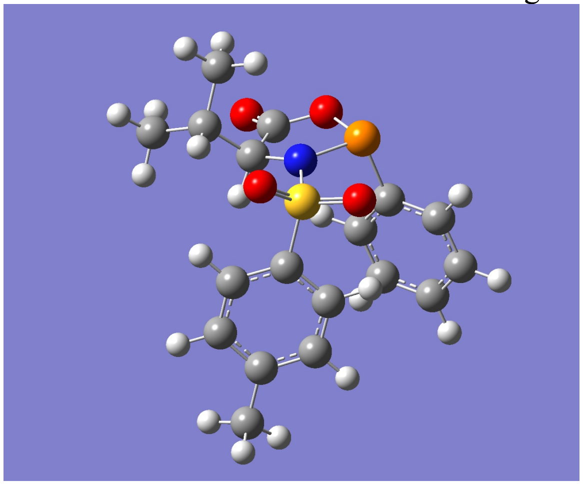

Center Number	Atomic Number	Atomic Type	Coordinates (Angstroms) X	Y	Z
1	15	0	1.344014	1.909340	-0.716079
2	16	0	0.363747	-0.658237	-1.795591

3	7	0	1.334723	0.139177	-0.710303
4	8	0	2.246372	1.863740	0.722268
5	8	0	2.819777	0.589885	2.460218
6	8	0	-0.058341	0.394653	-2.716894
7	8	0	1.063161	-1.833440	-2.295453
8	6	0	2.343903	0.676145	1.359383
9	6	0	1.820045	-0.488482	0.521858
10	6	0	2.907294	-1.564632	0.277054
11	6	0	3.182336	-2.377450	1.543217
12	6	0	4.179867	-0.960544	-0.316022
13	6	0	-4.351710	-2.554134	1.517221
14	6	0	-0.951249	-2.446704	-0.191490
15	6	0	-2.024648	-2.861036	0.587665
16	6	0	-3.180542	-2.078725	0.699038
17	6	0	-3.235280	-0.862513	0.010156
18	6	0	-2.168855	-0.425828	-0.766487
19	6	0	-1.032983	-1.225101	-0.859743
20	1	0	0.988798	-0.935859	1.086574
21	1	0	2.476628	-2.239521	-0.468408
22	1	0	3.865267	-3.198808	1.307847
23	1	0	2.259823	-2.811333	1.944392
24	1	0	3.638501	-1.764235	2.324372
25	1	0	4.873986	-1.756100	-0.599992
26	1	0	4.691781	-0.316795	0.408556
27	1	0	3.958942	-0.372522	-1.211829
28	1	0	-5.002258	-3.194360	0.911754
29	1	0	-4.952574	-1.715032	1.876011
30	1	0	-4.021924	-3.139381	2.379246
31	1	0	-0.067521	-3.068705	-0.288965
32	1	0	-1.965336	-3.809993	1.112282
33	1	0	-4.122547	-0.241054	0.085329
34	1	0	-2.218034	0.522247	-1.290601
35	6	0	-0.300883	2.334605	-0.029257
36	6	0	-1.138329	3.082496	-0.862807
37	6	0	-0.765870	1.927601	1.227373
38	6	0	-2.434447	3.399133	-0.458095
39	1	0	-0.784308	3.405147	-1.838403
40	6	0	-2.054554	2.251275	1.634699
41	1	0	-0.131401	1.358662	1.900057
42	6	0	-2.891599	2.981417	0.789281
43	1	0	-3.081604	3.972272	-1.113797
44	1	0	-2.409677	1.930213	2.608436
45	1	0	-3.899443	3.228274	1.107819

----

15 isomer A (cis, chair), ωB97x-D/6-31+G(d,p), benzene IEFPCM:
Sum of electronic and thermal Free Energies= -901.192958

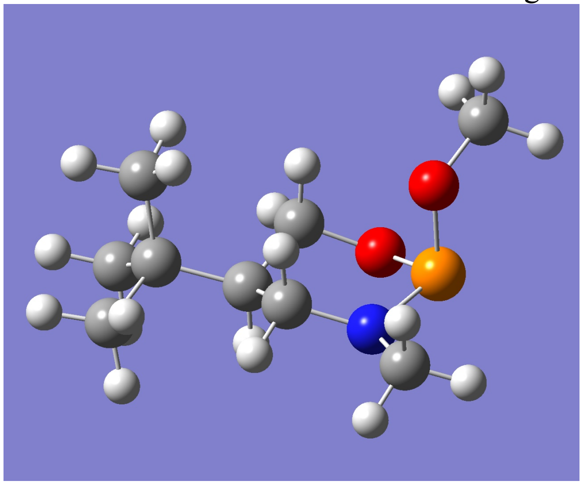

Center Number	Atomic Number	Atomic Type	Coordinates (Angstroms) X	Y	Z
1	6	0	0.238770	-1.208988	-0.534359
2	6	0	0.264796	1.165022	0.262930
3	6	0	1.068541	0.073329	-0.457724
4	1	0	-0.008219	-1.574389	0.469164
5	1	0	0.762969	-1.998914	-1.073885
6	1	0	0.804773	2.114665	0.201329
7	1	0	0.149379	0.922735	1.331839
8	1	0	1.214460	0.420067	-1.490292
9	6	0	2.486557	-0.161455	0.149140
10	6	0	2.428803	-0.888413	1.504063
11	1	0	3.433461	-0.947974	1.936542
12	1	0	1.790095	-0.364888	2.222764
13	1	0	2.056838	-1.912956	1.403337
14	6	0	3.325409	-1.002327	-0.830072
15	1	0	3.406566	-0.506532	-1.803734
16	1	0	4.338526	-1.138629	-0.436697
17	1	0	2.904260	-1.999048	-0.991468
18	6	0	3.206054	1.184692	0.341839
19	1	0	4.255352	1.012764	0.603945
20	1	0	3.183254	1.782489	-0.576730
21	1	0	2.762920	1.777496	1.147973
22	15	0	-2.072322	0.092740	-0.692249
23	8	0	-0.978781	-0.998298	-1.262454
24	7	0	-1.041414	1.391122	-0.359210
25	6	0	-1.691236	2.617753	0.081965
26	1	0	-1.069073	3.479949	-0.181990
27	1	0	-2.656390	2.729969	-0.421073

28	1	0	-1.864629	2.631495	1.168242
29	8	0	-2.299847	-0.475340	0.871746
30	6	0	-3.146987	-1.605426	1.031291
31	1	0	-2.631899	-2.526045	0.733151
32	1	0	-3.411672	-1.669434	2.088259
33	1	0	-4.065515	-1.505638	0.438623

----

15 isomer B (cis, twist 1), ωB97x-D/6-31+G(d,p), benzene IEFPCM:
Sum of electronic and thermal Free Energies= -901.185457

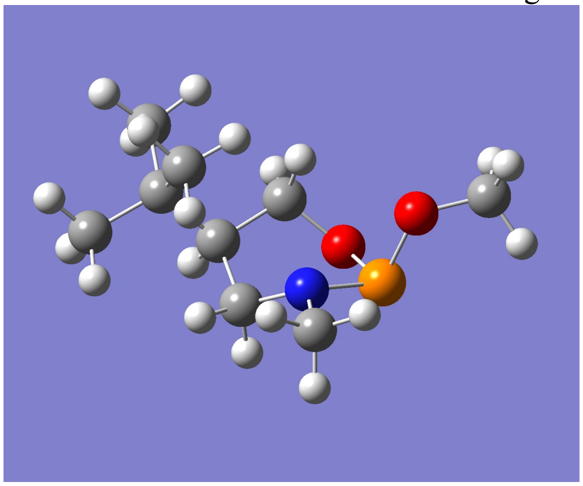

Center Number	Atomic Number	Atomic Type	Coordinates (Angstroms) X	Y	Z
1	6	0	-0.089778	-1.221938	-0.257159
2	6	0	0.146733	1.227258	0.226826
3	6	0	0.841593	-0.006954	-0.387900
4	1	0	0.038465	-1.718667	0.710026
5	1	0	0.093556	-1.951176	-1.047419
6	1	0	-0.386739	1.786035	-0.559045
7	1	0	0.890073	1.919523	0.632247
8	1	0	0.966997	0.195898	-1.459909
9	6	0	2.265691	-0.288880	0.177068
10	6	0	2.277979	-0.347392	1.713105
11	1	0	3.288536	-0.580323	2.066892
12	1	0	1.981993	0.608180	2.156937
13	1	0	1.600149	-1.112091	2.102710
14	6	0	2.785593	-1.625285	-0.382080
15	1	0	2.734022	-1.642653	-1.477104
16	1	0	3.831837	-1.773728	-0.094023
17	1	0	2.218616	-2.480261	-0.000009
18	6	0	3.223643	0.819589	-0.290264
19	1	0	4.235161	0.630839	0.085493

20	1	0	3.273079	0.860420	-1.384302
21	1	0	2.921909	1.808257	0.070571
22	15	0	-2.152016	-0.002156	0.851149
23	8	0	-1.463104	-0.828189	-0.396349
24	7	0	-0.746355	0.859402	1.328032
25	6	0	-0.954388	1.904723	2.318608
26	1	0	0.001993	2.151470	2.792636
27	1	0	-1.377915	2.828077	1.892017
28	1	0	-1.625738	1.544318	3.102446
29	8	0	-2.128814	-1.069224	2.125011
30	6	0	-3.297158	-1.856087	2.341418
31	1	0	-3.296313	-2.736621	1.690622
32	1	0	-3.278711	-2.180543	3.383154
33	1	0	-4.211584	-1.278467	2.161002

----

15 isomer C (cis, twist 2), ωB97x-D/6-31+G(d,p), benzene IEFPCM:
Sum of electronic and thermal Free Energies= -901.184267

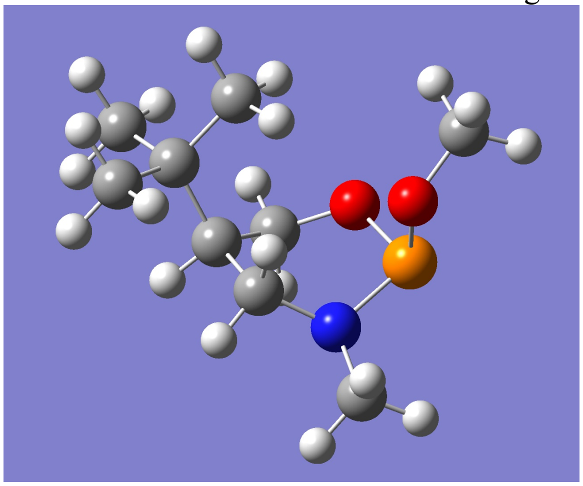

Center Number	Atomic Number	Atomic Type	Coordinates (Angstroms) X	Y	Z
1	6	0	0.767150	-1.180790	-1.666861
2	6	0	0.573664	1.272138	-1.072847
3	6	0	1.518995	0.041331	-1.126281
4	1	0	1.319508	-2.103189	-1.481477
5	1	0	0.646980	-1.076270	-2.750671
6	1	0	1.063057	2.130564	-1.542728
7	1	0	0.361959	1.557643	-0.034734
8	1	0	2.286298	0.251144	-1.884898
9	6	0	2.298274	-0.190974	0.202728
10	6	0	1.376545	-0.652921	1.341782
11	1	0	1.953151	-0.762405	2.267294

12	1	0	0.565224	0.057705	1.524823
13	1	0	0.915459	-1.617821	1.109833
14	6	0	3.390926	-1.252220	-0.014974
15	1	0	4.053837	-0.975579	-0.843194
16	1	0	4.005120	-1.348659	0.886772
17	1	0	2.975560	-2.241933	-0.227301
18	6	0	3.003253	1.116843	0.606642
19	1	0	3.631694	0.950376	1.488151
20	1	0	3.649591	1.480420	-0.201225
21	1	0	2.294825	1.911732	0.858235
22	15	0	-1.677751	-0.200738	-1.197883
23	8	0	-0.521676	-1.383558	-1.082147
24	7	0	-0.691521	1.044902	-1.782281
25	6	0	-1.370131	2.266106	-2.197043
26	1	0	-2.317819	2.016388	-2.682583
27	1	0	-1.581142	2.936511	-1.351067
28	1	0	-0.748210	2.804065	-2.920196
29	8	0	-1.785390	0.257052	0.406470
30	6	0	-2.449602	-0.622844	1.302604
31	1	0	-1.801908	-1.466950	1.563923
32	1	0	-2.682664	-0.055400	2.205364
33	1	0	-3.383648	-1.006718	0.871765

----

16 isomer A (trans, chair), ωB97x-D/6-31+G(d,p), benzene IEFPCM:
Sum of electronic and thermal Free Energies= -901.180788

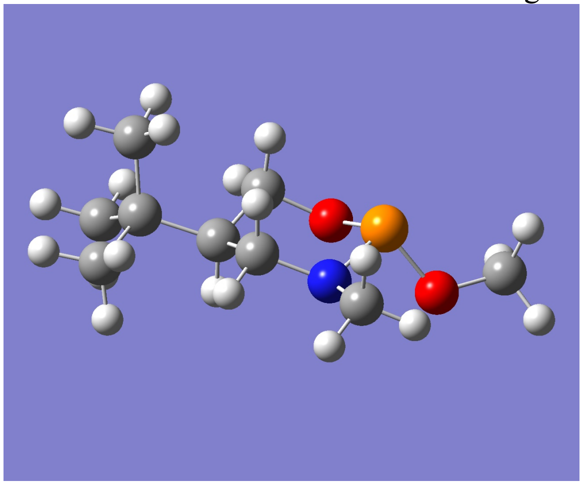

Center Number	Atomic Number	Atomic Type	Coordinates (Angstroms) X	Y	Z
1	6	0	0.473629	-1.320264	0.394290
2	6	0	0.450369	1.155962	0.065171
3	6	0	1.157766	-0.151048	-0.319223

4	1	0	0.548526	-1.205537	1.486263
5	1	0	0.916063	-2.280662	0.126215
6	1	0	0.860796	1.983827	-0.520192
7	1	0	0.643858	1.394488	1.128400
8	1	0	0.988149	-0.296179	-1.394401
9	6	0	2.701577	-0.119479	-0.097792
10	6	0	3.083043	-0.165265	1.392280
11	1	0	4.167709	-0.057518	1.501111
12	1	0	2.613220	0.643862	1.960847
13	1	0	2.802673	-1.115432	1.858144
14	6	0	3.338391	-1.327154	-0.809132
15	1	0	3.103046	-1.321560	-1.879095
16	1	0	4.428201	-1.294443	-0.704168
17	1	0	3.002090	-2.281577	-0.393235
18	6	0	3.296177	1.155499	-0.720540
19	1	0	4.389629	1.095161	-0.723134
20	1	0	2.964267	1.282742	-1.757391
21	1	0	3.022337	2.054736	-0.160131
22	15	0	-1.858825	-0.156470	0.531880
23	8	0	-0.900012	-1.405484	0.011194
24	7	0	-0.989417	1.127130	-0.221357
25	6	0	-1.606628	2.437718	-0.041697
26	1	0	-1.142151	3.148273	-0.733121
27	1	0	-2.670208	2.384508	-0.285477
28	1	0	-1.495750	2.832265	0.982244
29	6	0	-4.278154	-0.910378	-0.189106
30	1	0	-4.527309	-0.717149	0.860470
31	1	0	-5.071570	-0.516466	-0.825827
32	1	0	-4.189924	-1.989301	-0.347550
33	8	0	-3.071099	-0.252312	-0.571532

----

16 isomer B (trans, twist 1), ωB97x-D/6-31+G(d,p), benzene IEFPCM:
Sum of electronic and thermal Free Energies= -901.188816

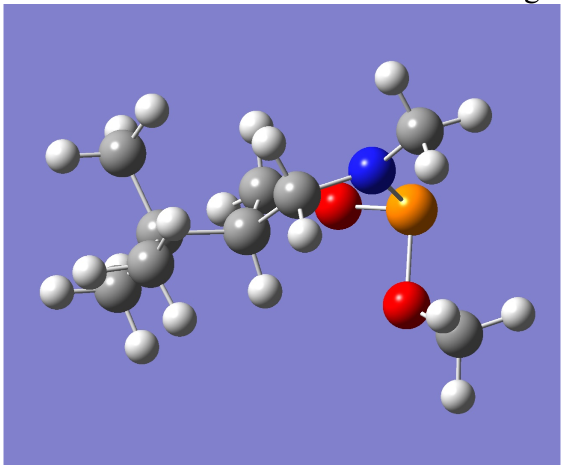

Center Number	Atomic Number	Atomic Type	Coordinates (Angstroms) X	Y	Z
1	6	0	0.696744	-1.652985	1.112959
2	6	0	0.395519	0.820017	1.010474
3	6	0	0.917525	-0.410656	0.248207
4	1	0	1.124438	-1.506538	2.112613
5	1	0	1.151683	-2.540305	0.669994
6	1	0	0.167945	1.634042	0.306402
7	1	0	1.170619	1.194004	1.695265
8	1	0	0.300511	-0.537716	-0.648963
9	6	0	2.389363	-0.251626	-0.236665
10	6	0	3.396674	-0.381296	0.918034
11	1	0	4.410651	-0.187205	0.552363
12	1	0	3.194944	0.333467	1.722854
13	1	0	3.391684	-1.387191	1.350441
14	6	0	2.697197	-1.330453	-1.289521
15	1	0	2.015410	-1.250457	-2.143119
16	1	0	3.720172	-1.212614	-1.662969
17	1	0	2.612992	-2.343438	-0.884624
18	6	0	2.566918	1.121713	-0.906270
19	1	0	3.560339	1.191293	-1.362054
20	1	0	1.822924	1.274296	-1.696478
21	1	0	2.477935	1.944455	-0.189812
22	15	0	-1.805013	-0.787280	1.469928
23	8	0	-0.692197	-1.976850	1.259692
24	7	0	-0.782962	0.512509	1.817363
25	6	0	-3.058952	0.457779	-0.463659
26	1	0	-2.577893	1.435962	-0.330313
27	1	0	-3.356526	0.350128	-1.508025
28	1	0	-3.958752	0.418398	0.165460
29	8	0	-2.160501	-0.599175	-0.166437
30	6	0	-1.264548	1.616149	2.627943
31	1	0	-0.493223	1.925538	3.344116
32	1	0	-1.531882	2.489403	2.014523
33	1	0	-2.149061	1.308160	3.191607

16 isomer C (trans, twist 2), ωB97x-D/6-31+G(d,p), benzene IEFPCM:
Sum of electronic and thermal Free Energies= -901.187796

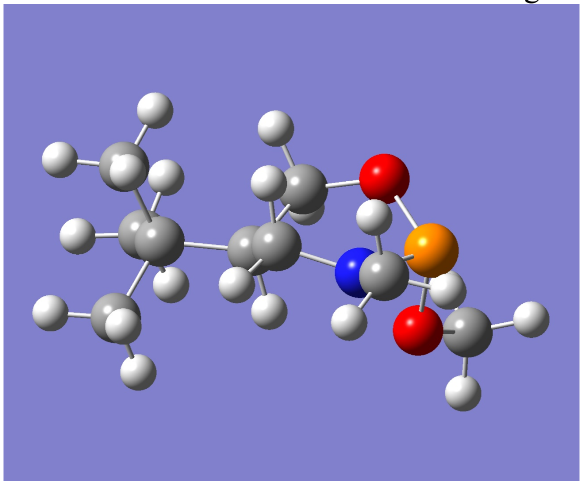

Center Number	Atomic Number	Atomic Type	Coordinates (Angstroms) X	Y	Z
1	6	0	0.061707	-1.249352	0.451075
2	6	0	0.336070	1.141569	-0.243857
3	6	0	0.843742	-0.288349	-0.458359
4	1	0	0.487093	-1.266623	1.459484
5	1	0	0.090085	-2.266469	0.048367
6	1	0	0.951049	1.852625	-0.806975
7	1	0	0.417721	1.415168	0.821750
8	1	0	0.610043	-0.553869	-1.495712
9	6	0	2.387113	-0.432231	-0.296517
10	6	0	2.897822	0.191257	1.012693
11	1	0	3.970618	-0.000326	1.122752
12	1	0	2.755282	1.276522	1.026985
13	1	0	2.397269	-0.226694	1.892370
14	6	0	2.771647	-1.922218	-0.326616
15	1	0	2.367621	-2.416160	-1.217760
16	1	0	3.861458	-2.027999	-0.349563
17	1	0	2.411081	-2.460479	0.555760
18	6	0	3.088349	0.255002	-1.480825
19	1	0	4.173784	0.132126	-1.399295
20	1	0	2.769875	-0.183144	-2.432964
21	1	0	2.884228	1.329294	-1.517981
22	15	0	-2.137712	0.025295	-0.468787
23	8	0	-1.317761	-0.887405	0.637320
24	7	0	-1.045896	1.295500	-0.690635
25	6	0	-2.546871	-2.043640	-2.037781
26	1	0	-3.615664	-1.904922	-1.829307
27	1	0	-2.425809	-2.353954	-3.076902

28	1	0	-2.157512	-2.829786	-1.380095
29	8	0	-1.820894	-0.831705	-1.872841
30	6	0	-1.548690	2.657303	-0.611088
31	1	0	-2.606447	2.685367	-0.884048
32	1	0	-1.440687	3.080238	0.399459
33	1	0	-1.002601	3.299157	-1.312193

----

17 isomer A (cis, chair), ωB97x-D/6-31+G(d,p), benzene IEFPCM:
Sum of electronic and thermal Free Energies= -920.578892

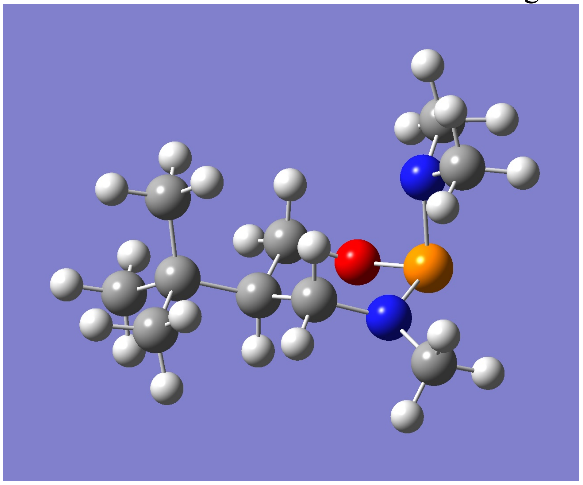

Center Number	Atomic Number	Atomic Type	Coordinates (Angstroms) X	Y	Z
1	6	0	0.467332	-1.158762	-0.768415
2	6	0	0.399094	1.127856	0.297213
3	6	0	1.267830	0.113579	-0.468455
4	1	0	0.171682	-1.656088	0.161005
5	1	0	1.047356	-1.856227	-1.374728
6	1	0	0.938741	2.075614	0.376918
7	1	0	0.201897	0.776226	1.322243
8	1	0	1.500180	0.579042	-1.436889
9	6	0	2.630717	-0.189257	0.228460
10	6	0	2.462711	-1.042787	1.497875
11	1	0	3.428449	-1.149643	2.004007
12	1	0	1.765362	-0.587283	2.208228
13	1	0	2.101469	-2.050068	1.268590
14	6	0	3.552862	-0.936001	-0.752321
15	1	0	3.702541	-0.356091	-1.669897
16	1	0	4.534454	-1.100935	-0.294935
17	1	0	3.157539	-1.916888	-1.032021
18	6	0	3.327755	1.128449	0.610248
19	1	0	4.351841	0.927406	0.942286

20	1	0	3.380628	1.811971	-0.245247
21	1	0	2.816553	1.643546	1.429463
22	15	0	-1.843634	0.143939	-0.918142
23	8	0	-0.709507	-0.885154	-1.541478
24	7	0	-0.847197	1.409774	-0.409832
25	6	0	-1.366342	2.759178	-0.297157
26	1	0	-0.643355	3.481545	-0.695262
27	1	0	-2.289820	2.848102	-0.876363
28	1	0	-1.590761	3.038587	0.743656
29	7	0	-2.121543	-0.704101	0.594232
30	6	0	-2.782681	-1.992752	0.424304
31	1	0	-2.749482	-2.545767	1.369675
32	1	0	-3.840364	-1.899138	0.121988
33	1	0	-2.260342	-2.584986	-0.331239
34	6	0	-2.763775	0.103848	1.620382
35	1	0	-2.789672	-0.460971	2.558677
36	1	0	-2.191275	1.017657	1.799482
37	1	0	-3.801839	0.387011	1.370319

----

17 isomer B (cis, twist 1), ωB97x-D/6-31+G(d,p), benzene IEFPCM:
Sum of electronic and thermal Free Energies= -920.578915

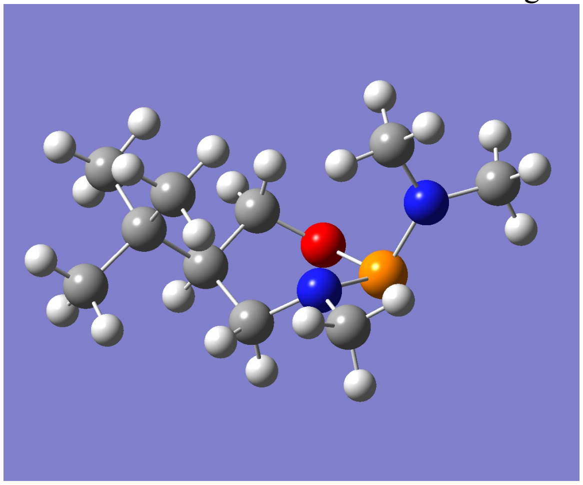

Center Number	Atomic Number	Atomic Type	Coordinates (Angstroms) X	Y	Z
1	6	0	0.038773	-1.385167	-0.202419
2	6	0	0.104572	1.077452	0.270955
3	6	0	0.873917	-0.104292	-0.357934
4	1	0	0.230870	-1.865212	0.763144
5	1	0	0.277370	-2.105881	-0.987074
6	1	0	-0.473544	1.604317	-0.506568
7	1	0	0.805157	1.815824	0.671730

8	1	0	0.954578	0.100410	-1.433925
9	6	0	2.331202	-0.272523	0.165636
10	6	0	2.391202	-0.351045	1.699619
11	1	0	3.431754	-0.454473	2.026882
12	1	0	1.979869	0.547643	2.169662
13	1	0	1.836487	-1.211241	2.086584
14	6	0	2.946454	-1.553336	-0.425569
15	1	0	2.871315	-1.558805	-1.519224
16	1	0	4.007627	-1.620165	-0.162306
17	1	0	2.459111	-2.457196	-0.046148
18	6	0	3.181387	0.918177	-0.308500
19	1	0	4.218533	0.801343	0.024341
20	1	0	3.184605	0.986285	-1.402221
21	1	0	2.818270	1.872114	0.087177
22	15	0	-2.149109	-0.243928	0.854342
23	8	0	-1.354844	-1.105515	-0.334821
24	7	0	-0.753147	0.645869	1.377226
25	6	0	-1.032518	1.695650	2.346264
26	1	0	-0.092331	2.031646	2.798189
27	1	0	-1.535158	2.571931	1.904955
28	1	0	-1.666965	1.301382	3.144624
29	7	0	-2.347302	-1.210884	2.229933
30	6	0	-1.277948	-1.782049	3.032443
31	1	0	-1.570430	-1.782971	4.089885
32	1	0	-1.058129	-2.819809	2.740296
33	1	0	-0.377252	-1.175936	2.921683
34	6	0	-3.631695	-1.869532	2.407031
35	1	0	-3.941215	-1.816893	3.458230
36	1	0	-4.392932	-1.373379	1.798979
37	1	0	-3.592174	-2.928579	2.113969

----

17 isomer C (cis, twist 2), ωB97x-D/6-31+G(d,p), benzene IEFPCM:
Sum of electronic and thermal Free Energies= -920.576045

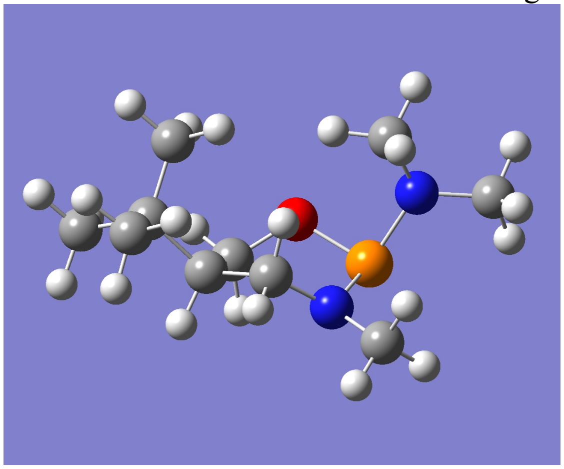

Center Number	Atomic Number	Atomic Type	Coordinates (Angstroms) X	Y	Z
1	6	0	0.721086	-1.371523	-1.088312
2	6	0	0.556122	1.101709	-0.655447
3	6	0	1.526794	-0.068279	-0.923191
4	1	0	1.285533	-2.244628	-0.754986
5	1	0	0.473016	-1.528597	-2.145483
6	1	0	0.999956	2.029396	-1.030979
7	1	0	0.408712	1.247461	0.424956
8	1	0	1.999315	0.128332	-1.895640
9	6	0	2.684429	-0.153207	0.113655
10	6	0	2.191580	-0.617198	1.493420
11	1	0	3.038798	-0.709734	2.181943
12	1	0	1.488804	0.096744	1.934164
13	1	0	1.691888	-1.589529	1.442247
14	6	0	3.751490	-1.138119	-0.394464
15	1	0	4.132326	-0.832228	-1.375598
16	1	0	4.598644	-1.171190	0.299245
17	1	0	3.367432	-2.159057	-0.485213
18	6	0	3.350372	1.226505	0.254540
19	1	0	4.233645	1.157310	0.898690
20	1	0	3.675657	1.608255	-0.720449
21	1	0	2.676697	1.964305	0.701584
22	15	0	-1.721393	-0.429415	-0.911390
23	8	0	-0.476095	-1.365719	-0.310757
24	7	0	-0.711755	0.903826	-1.350902
25	6	0	-1.433228	2.119924	-1.680460
26	1	0	-2.344392	1.869236	-2.234759
27	1	0	-1.728445	2.696763	-0.788362
28	1	0	-0.819002	2.764756	-2.318651
29	7	0	-2.485088	-0.126356	0.571857
30	6	0	-1.799284	0.279563	1.786251
31	1	0	-0.792799	-0.140470	1.797546
32	1	0	-1.738925	1.374616	1.881664
33	1	0	-2.336173	-0.108759	2.660153
34	6	0	-3.910168	0.155118	0.561895
35	1	0	-4.407735	-0.371689	1.385910
36	1	0	-4.118226	1.230353	0.669055
37	1	0	-4.351457	-0.186678	-0.378350

18 isomer A (trans, chair), ωB97x-D/6-31+G(d,p), benzene IEFPCM:
Sum of electronic and thermal Free Energies= -920.579964

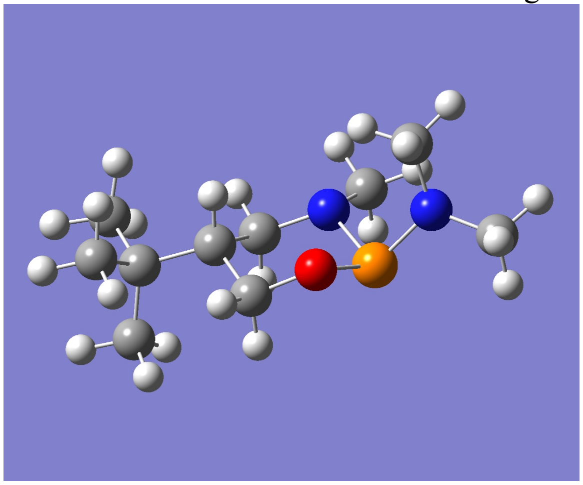

Center Number	Atomic Number	Atomic Type	Coordinates (Angstroms) X	Y	Z
1	6	0	-0.713684	-1.300839	-0.510958
2	6	0	-0.688875	1.163770	-0.124007
3	6	0	-1.368751	-0.155462	0.266564
4	1	0	-0.850433	-1.156113	-1.594019
5	1	0	-1.138776	-2.270823	-0.246067
6	1	0	-1.082668	1.978830	0.490705
7	1	0	-0.929024	1.416492	-1.175190
8	1	0	-1.139826	-0.324151	1.327873
9	6	0	-2.922454	-0.130995	0.133182
10	6	0	-3.387017	-0.142521	-1.333723
11	1	0	-4.477066	-0.042739	-1.378954
12	1	0	-2.955991	0.685362	-1.905908
13	1	0	-3.124411	-1.077440	-1.838940
14	6	0	-3.510372	-1.361214	0.848069
15	1	0	-3.216557	-1.379357	1.903494
16	1	0	-4.604614	-1.335999	0.803977
17	1	0	-3.189428	-2.301972	0.391065
18	6	0	-3.491021	1.122710	0.820215
19	1	0	-4.582207	1.052723	0.883095
20	1	0	-3.101845	1.227447	1.839672
21	1	0	-3.255678	2.037862	0.268247
22	15	0	1.606502	-0.109321	-0.777313
23	8	0	0.675617	-1.382713	-0.209162
24	7	0	0.760373	1.138031	0.100075
25	6	0	1.350656	2.460604	-0.072077
26	1	0	0.899685	3.151474	0.648424
27	1	0	2.424150	2.416237	0.128114

28	1	0	1.200540	2.875735	-1.083560
29	7	0	2.984962	-0.231665	0.168974
30	6	0	4.216713	-0.706095	-0.439435
31	1	0	4.166035	-0.592035	-1.525234
32	1	0	5.067800	-0.120518	-0.070807
33	1	0	4.403079	-1.764906	-0.209555
34	6	0	2.942512	-0.345212	1.618855
35	1	0	1.996101	0.054798	1.985887
36	1	0	3.039261	-1.391147	1.940360
37	1	0	3.764650	0.231503	2.060027

----

18 isomer B (trans, twist 1), ωB97x-D/6-31+G(d,p), benzene IEFPCM:
Sum of electronic and thermal Free Energies= -920.575476

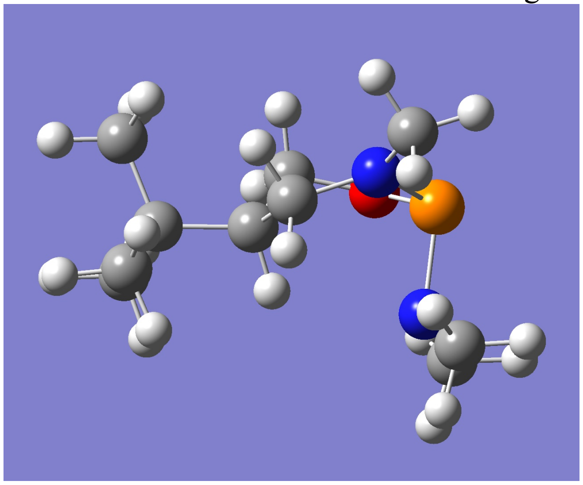

Center Number	Atomic Number	Atomic Type	Coordinates (Angstroms) X	Y	Z
1	6	0	0.759216	-1.619395	1.029998
2	6	0	0.517375	0.866364	0.910164
3	6	0	1.071813	-0.378295	0.189893
4	1	0	1.120482	-1.486260	2.057717
5	1	0	1.230334	-2.512837	0.615739
6	1	0	0.336815	1.674483	0.185272
7	1	0	1.258268	1.240675	1.631501
8	1	0	0.520498	-0.486977	-0.750111
9	6	0	2.579508	-0.249773	-0.179239
10	6	0	3.493785	-0.395208	1.048530
11	1	0	4.536539	-0.221438	0.761653
12	1	0	3.244337	0.325372	1.834602
13	1	0	3.435772	-1.399509	1.480909
14	6	0	2.947494	-1.336254	-1.204800
15	1	0	2.331073	-1.247891	-2.106153

16	1	0	3.996700	-1.235119	-1.503048
17	1	0	2.818941	-2.346798	-0.805339
18	6	0	2.833713	1.117788	-0.836317
19	1	0	3.860701	1.168402	-1.213774
20	1	0	2.155533	1.280310	-1.681846
21	1	0	2.703668	1.944870	-0.131304
22	15	0	-1.734391	-0.683042	1.235673
23	8	0	-0.641196	-1.921320	1.080825
24	7	0	-0.697902	0.576190	1.665843
25	6	0	-2.564294	-1.467096	-1.210386
26	1	0	-3.612597	-1.669544	-0.926768
27	1	0	-2.544441	-1.242378	-2.282596
28	1	0	-1.977312	-2.372562	-1.042296
29	6	0	-1.137340	1.632861	2.555046
30	1	0	-0.359446	1.855769	3.296459
31	1	0	-1.363561	2.562003	2.009615
32	1	0	-2.039702	1.321421	3.087305
33	7	0	-1.977999	-0.352637	-0.474428
34	6	0	-2.714118	0.885554	-0.696482
35	1	0	-3.763547	0.840320	-0.353851
36	1	0	-2.220566	1.717261	-0.185178
37	1	0	-2.722648	1.111674	-1.768161

----

18 isomer C (trans, twist 2), ωB97x-D/6-31+G(d,p), benzene IEFPCM:
Sum of electronic and thermal Free Energies= -920.578370

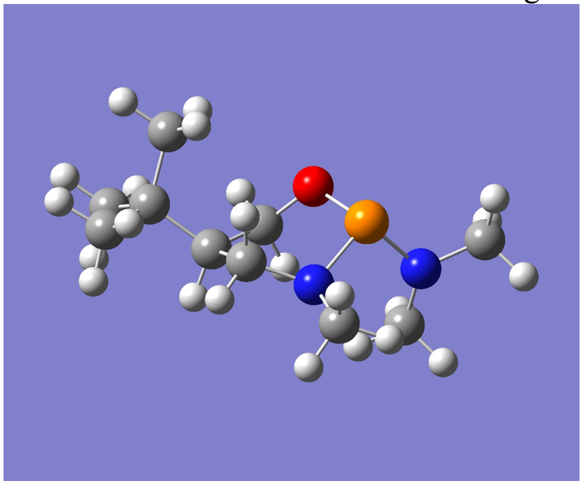

Center Number	Atomic Number	Atomic Type	Coordinates (Angstroms) X	Y	Z
1	6	0	0.776139	-1.425711	-0.767385
2	6	0	0.667852	1.079245	-0.799895
3	6	0	1.601775	-0.137584	-0.942403

4	1	0	1.378131	-2.245832	-0.370004
5	1	0	0.378337	-1.750314	-1.733740
6	1	0	1.065577	1.912662	-1.386214
7	1	0	0.634408	1.427579	0.247011
8	1	0	1.963940	-0.141918	-1.980500
9	6	0	2.864055	-0.051967	-0.036046
10	6	0	2.524351	-0.274062	1.446415
11	1	0	3.433472	-0.193667	2.052808
12	1	0	1.809548	0.466078	1.818274
13	1	0	2.091866	-1.264048	1.619411
14	6	0	3.881294	-1.116215	-0.481617
15	1	0	4.170653	-0.968775	-1.528557
16	1	0	4.788323	-1.054618	0.129538
17	1	0	3.489838	-2.133481	-0.379758
18	6	0	3.525441	1.327146	-0.202735
19	1	0	4.477601	1.358007	0.337908
20	1	0	3.732548	1.540582	-1.258227
21	1	0	2.899526	2.133705	0.191788
22	15	0	-1.577411	-0.249357	-0.233854
23	8	0	-0.296614	-1.243294	0.158353
24	7	0	-0.675155	0.782021	-1.305208
25	6	0	-3.755510	-1.678673	-0.911306
26	1	0	-4.024055	-1.306786	0.081127
27	1	0	-4.594122	-1.484472	-1.591409
28	1	0	-3.612684	-2.766952	-0.845340
29	6	0	-1.408401	1.965305	-1.737925
30	1	0	-2.392652	1.671274	-2.112354
31	1	0	-1.551007	2.705526	-0.933303
32	1	0	-0.866266	2.449046	-2.557160
33	7	0	-2.558644	-1.003804	-1.388318
34	6	0	-2.146824	-1.413537	-2.721288
35	1	0	-1.860387	-2.475367	-2.750266
36	1	0	-2.976209	-1.267571	-3.424528
37	1	0	-1.303828	-0.801688	-3.046604

19, ωB97x-D/6-31+G(d,p), chloroform IEFPCM:
Sum of electronic and thermal Free Energies= -2126.204849

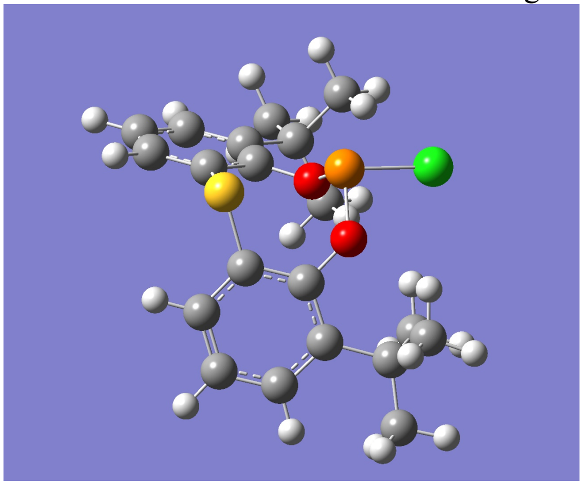

Center Number	Atomic Number	Atomic Type	Coordinates (Angstroms) X	Y	Z
1	15	0	0.035720	-0.722603	-1.829940
2	8	0	-0.866142	-0.489102	-0.453214
3	8	0	1.530824	-0.335645	-1.233441
4	16	0	-0.103417	2.121090	-1.707274
5	6	0	-1.814358	0.491376	-0.272149
6	6	0	-1.580093	1.783034	-0.762044
7	6	0	-2.508366	2.797167	-0.543020
8	1	0	-2.318314	3.792843	-0.929033
9	6	0	-3.660464	2.513242	0.176197
10	6	0	-3.867017	1.231593	0.679343
11	1	0	-4.770128	1.050617	1.248082
12	6	0	-2.961672	0.182349	0.480480
13	6	0	1.835022	0.688558	-0.365453
14	6	0	1.158319	1.912062	-0.463718
15	6	0	1.486039	2.971861	0.380238
16	1	0	0.951786	3.911823	0.292415
17	6	0	2.495983	2.804921	1.312839
18	6	0	3.166217	1.586110	1.395375
19	1	0	3.951549	1.492518	2.134257
20	6	0	2.863926	0.492198	0.578455
21	6	0	-3.214494	-1.222956	1.054272
22	6	0	-4.511748	-1.271184	1.878518
23	1	0	-4.482568	-0.586415	2.732550
24	1	0	-4.640421	-2.283672	2.272830
25	1	0	-5.395063	-1.041384	1.273685
26	6	0	-3.360323	-2.238629	-0.096247
27	1	0	-4.183197	-1.953299	-0.760224

28	1	0	-3.582476	-3.229758	0.313848
29	1	0	-2.447759	-2.321944	-0.686948
30	6	0	-2.059339	-1.635746	1.990552
31	1	0	-1.107422	-1.704295	1.463079
32	1	0	-2.277586	-2.616014	2.427767
33	1	0	-1.952742	-0.915784	2.809121
34	6	0	3.590733	-0.854006	0.745830
35	6	0	4.733418	-0.758766	1.771136
36	1	0	5.492385	-0.027157	1.474133
37	1	0	5.223945	-1.734346	1.840756
38	1	0	4.373136	-0.504733	2.773140
39	6	0	4.215111	-1.319361	-0.585432
40	1	0	4.909695	-0.564141	-0.969107
41	1	0	3.461626	-1.516378	-1.347329
42	1	0	4.779383	-2.242936	-0.417946
43	6	0	2.585849	-1.900189	1.267111
44	1	0	2.182760	-1.592958	2.238036
45	1	0	3.087873	-2.865504	1.395569
46	1	0	1.753476	-2.045438	0.578413
47	17	0	0.258520	-2.830382	-1.541062
48	1	0	-4.395751	3.289999	0.356687
49	1	0	2.769921	3.619139	1.975061

20, ωB97x-D/6-31+G(d,p), chloroform IEFPCM:
Sum of electronic and thermal Free Energies= -3749.424442

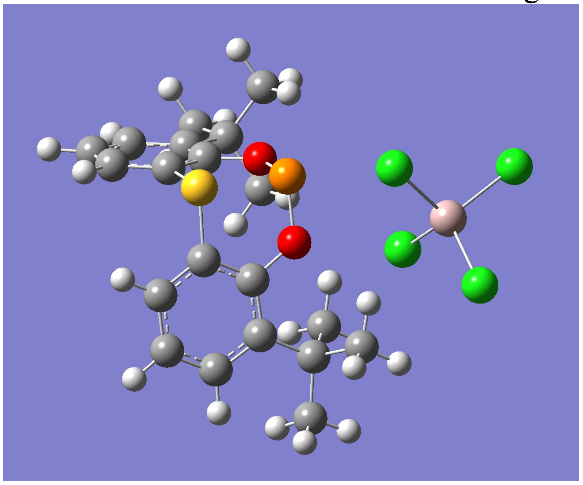

Center Number	Atomic Number	Atomic Type	Coordinates (Angstroms) X	Y	Z
1	15	0	-0.834332	0.061484	-2.008407
2	8	0	-1.075979	-1.189670	-0.992723
3	8	0	-0.512701	1.344171	-1.063089

4	16	0	-3.114968	0.566982	-1.872227
5	6	0	-2.267523	-1.522889	-0.388950
6	6	0	-3.400747	-0.810504	-0.777007
7	6	0	-4.663241	-1.126250	-0.289906
8	1	0	-5.541070	-0.573451	-0.603834
9	6	0	-4.752552	-2.170860	0.619406
10	6	0	-3.607849	-2.859754	1.020462
11	1	0	-3.727182	-3.660087	1.739784
12	6	0	-2.325828	-2.568983	0.537533
13	6	0	-1.452755	2.060067	-0.350472
14	6	0	-2.789998	1.866316	-0.692476
15	6	0	-3.807394	2.608452	-0.104953
16	1	0	-4.844791	2.459267	-0.381857
17	6	0	-3.443121	3.538000	0.859014
18	6	0	-2.104044	3.704714	1.211918
19	1	0	-1.870434	4.435538	1.975779
20	6	0	-1.058515	2.980624	0.625687
21	6	0	-1.067295	-3.319456	0.994769
22	6	0	-1.406401	-4.386706	2.045919
23	1	0	-1.855991	-3.950949	2.944256
24	1	0	-0.481917	-4.885088	2.350511
25	1	0	-2.079509	-5.155116	1.651293
26	6	0	-0.409600	-4.027430	-0.207236
27	1	0	-1.106772	-4.734115	-0.669751
28	1	0	0.465011	-4.587763	0.137524
29	1	0	-0.071014	-3.320089	-0.965674
30	6	0	-0.078413	-2.321227	1.630770
31	1	0	0.271699	-1.572140	0.918497
32	1	0	0.803191	-2.857912	1.993037
33	1	0	-0.538074	-1.808143	2.482208
34	6	0	0.413348	3.168333	1.016704
35	6	0	0.562768	4.191808	2.152087
36	1	0	0.212876	5.187277	1.859206
37	1	0	1.622487	4.278748	2.407331
38	1	0	0.030198	3.882160	3.057450
39	6	0	1.210626	3.685114	-0.197879
40	1	0	0.793218	4.630895	-0.559585
41	1	0	1.219424	2.966816	-1.019449
42	1	0	2.249632	3.856882	0.097317
43	6	0	0.994818	1.827482	1.508382
44	1	0	0.432401	1.450337	2.369071
45	1	0	2.034639	1.969209	1.816129
46	1	0	0.990056	1.062175	0.730125
47	17	0	2.176413	-0.864795	-1.533269
48	1	0	-5.718036	-2.450841	1.024949
49	1	0	-4.205366	4.135885	1.345401

50	13	0	3.910531	-0.478471	-0.264016
51	17	0	5.531872	-1.707813	-0.975827
52	17	0	4.430326	1.607889	-0.411875
53	17	0	3.438036	-0.988952	1.775297

21, ωB97x-D/6-31+G(d,p), dichloromethane IEFPCM:
Sum of electronic and thermal Free Energies= -3591.472942

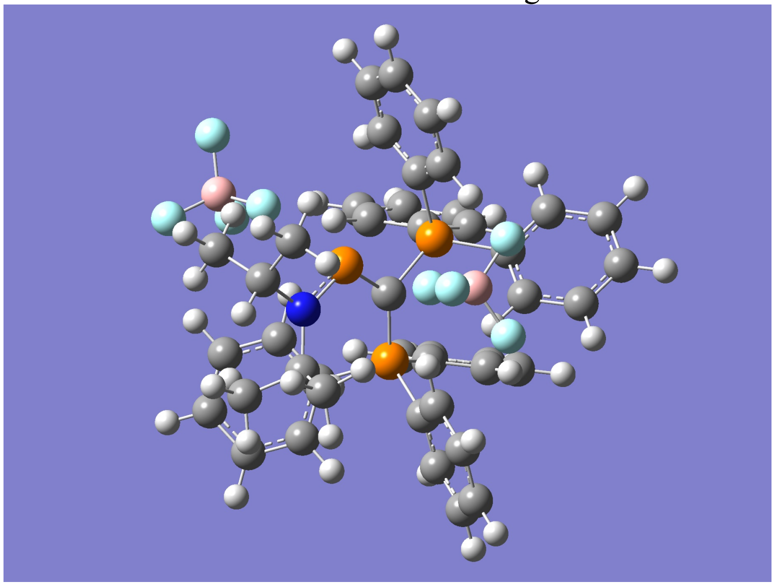

Center Number	Atomic Number	Atomic Type	Coordinates (Angstroms) X	Y	Z
1	6	0	8.623462	10.500053	7.658389
2	6	0	10.884611	7.015491	7.011350
3	1	0	11.353810	6.380700	7.762849
4	6	0	11.978571	7.542788	6.088819
5	1	0	12.496915	6.701179	5.620274
6	1	0	12.708002	8.136369	6.645609
7	1	0	11.562838	8.176699	5.300453
8	6	0	9.845279	6.160876	6.286053
9	1	0	9.395108	6.692143	5.442265
10	1	0	9.048055	5.854359	6.967569
11	1	0	10.337011	5.266140	5.894062
12	6	0	10.514194	8.076439	9.262471
13	1	0	10.039813	8.951360	9.695765
14	6	0	9.848148	6.841151	9.873427
15	1	0	8.807140	6.757223	9.554307
16	1	0	9.875015	6.926959	10.963418
17	1	0	10.369238	5.919558	9.599003
18	6	0	12.004191	8.158585	9.587527
19	1	0	12.541703	7.256851	9.281407

20	1	0	12.122776	8.263927	10.669211
21	1	0	12.467202	9.020945	9.104577
22	6	0	7.993094	12.973722	6.129498
23	6	0	7.137967	14.073795	6.234396
24	1	0	6.129137	13.964752	6.613282
25	6	0	7.597308	15.339570	5.883264
26	1	0	6.932336	16.191557	5.976549
27	6	0	8.904508	15.514922	5.435893
28	1	0	9.256804	16.505493	5.167130
29	6	0	9.763776	14.421681	5.349066
30	1	0	10.788757	14.538909	5.015829
31	6	0	9.312365	13.153994	5.696867
32	1	0	9.995716	12.314788	5.621085
33	6	0	7.383972	10.332310	4.972978
34	6	0	6.558306	9.199650	4.949942
35	1	0	5.981321	8.906868	5.821413
36	6	0	6.497327	8.417134	3.803605
37	1	0	5.865653	7.535484	3.794417
38	6	0	7.246477	8.762711	2.679152
39	1	0	7.198276	8.148374	1.785908
40	6	0	8.054794	9.896969	2.699047
41	1	0	8.635963	10.171537	1.825500
42	6	0	8.127486	10.685401	3.843832
43	1	0	8.767407	11.558812	3.842716
44	6	0	5.761297	11.272444	7.123630
45	6	0	4.761517	11.856407	6.331418
46	1	0	5.008116	12.333928	5.388053
47	6	0	3.435757	11.801374	6.741783
48	1	0	2.665936	12.257527	6.128847
49	6	0	3.095505	11.147075	7.927376
50	1	0	2.057642	11.100073	8.240090
51	6	0	4.081354	10.530817	8.690802
52	1	0	3.823729	9.983213	9.590335
53	6	0	5.411803	10.587681	8.285367
54	1	0	6.161241	10.067409	8.864358
55	6	0	8.431610	13.031896	9.260857
56	6	0	7.111330	13.293695	9.646653
57	1	0	6.433663	12.484180	9.894739
58	6	0	6.662962	14.606890	9.730530
59	1	0	5.639025	14.804703	10.028562
60	6	0	7.529422	15.659146	9.441873
61	1	0	7.178557	16.683566	9.510526
62	6	0	8.845675	15.399673	9.066156
63	1	0	9.521180	16.216788	8.838307
64	6	0	9.301150	14.089194	8.975238
65	1	0	10.327520	13.898764	8.681182

66	6	0	10.830425	11.385104	9.361952
67	6	0	11.617856	11.514669	8.214058
68	1	0	11.178151	11.489534	7.224589
69	6	0	12.997116	11.663222	8.330673
70	1	0	13.593220	11.753753	7.429630
71	6	0	13.591749	11.681595	9.589569
72	1	0	14.667149	11.793507	9.679729
73	6	0	12.808273	11.542407	10.734885
74	1	0	13.269971	11.540796	11.716329
75	6	0	11.429901	11.393974	10.626607
76	1	0	10.838836	11.269350	11.526670
77	6	0	8.282478	10.531518	10.651189
78	6	0	7.687915	9.271219	10.570395
79	1	0	7.637145	8.735459	9.627778
80	6	0	7.122627	8.695231	11.704360
81	1	0	6.649116	7.723809	11.620032
82	6	0	7.154638	9.371490	12.920403
83	1	0	6.713915	8.920830	13.803523
84	6	0	7.737658	10.636601	13.002798
85	1	0	7.750458	11.173641	13.945019
86	6	0	8.292834	11.222877	11.871885
87	1	0	8.712550	12.221766	11.941879
88	7	0	10.257589	8.138959	7.805942
89	15	0	9.295827	9.091550	6.894506
90	15	0	7.466766	11.283092	6.519296
91	15	0	9.025731	11.318507	9.193611
92	5	0	12.455584	11.351353	4.578539
93	5	0	5.838579	6.924951	8.086238
94	9	0	13.607879	10.657696	4.983776
95	9	0	12.485163	12.663268	5.101993
96	9	0	12.376652	11.396379	3.178108
97	9	0	11.303733	10.691062	5.089917
98	9	0	7.052043	7.636759	7.857792
99	9	0	5.315563	7.319093	9.331874
100	9	0	4.929589	7.236546	7.059444
101	9	0	6.112396	5.548135	8.092938

22, ωB97x-D/6-31+G(d,p), acetonitrile IEFPCM:
Sum of electronic and thermal Free Energies= -4591.551023

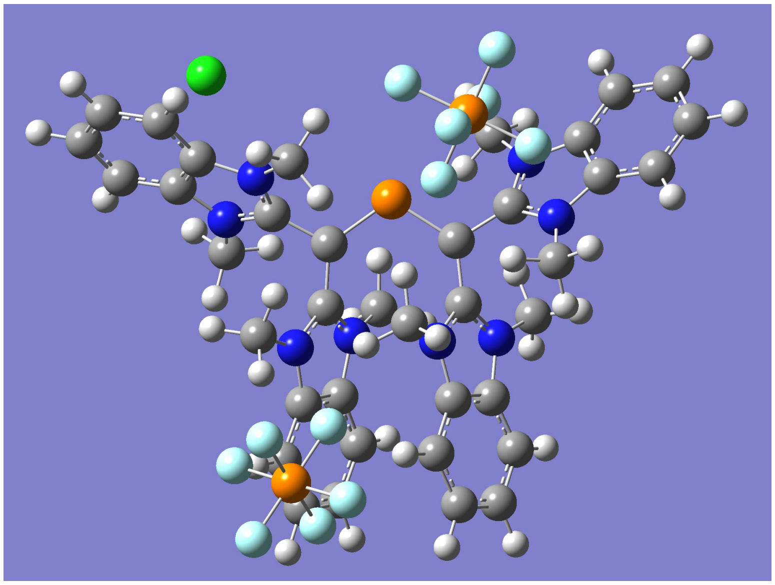

Center Number	Atomic Number	Atomic Type	Coordinates (Angstroms) X	Y	Z
1	6	0	1.650971	-1.026143	-0.862984
2	6	0	0.585622	-1.989080	-0.645891
3	6	0	-1.284259	-2.961886	0.071135
4	6	0	-2.501077	-3.265693	0.676951
5	1	0	-2.972691	-2.591682	1.382546
6	6	0	-3.089139	-4.462819	0.298846
7	1	0	-4.044026	-4.740182	0.731139
8	6	0	-2.483637	-5.323766	-0.636797
9	1	0	-2.982228	-6.249786	-0.900574
10	6	0	-1.271666	-5.015990	-1.236841
11	1	0	-0.811142	-5.679364	-1.959124
12	6	0	-0.687887	-3.806670	-0.863616
13	6	0	-0.568918	-0.900584	1.298361
14	1	0	0.231799	-0.167984	1.230783
15	1	0	-0.480976	-1.442029	2.242485
16	1	0	-1.540749	-0.413629	1.267405
17	6	0	1.406171	-3.721725	-2.262683
18	1	0	1.963157	-2.914595	-2.735561
19	1	0	0.845206	-4.258106	-3.028099
20	1	0	2.095927	-4.408507	-1.768724
21	6	0	3.002210	-1.529329	-0.665928
22	6	0	4.720932	-2.611587	0.249797
23	6	0	5.577266	-3.368523	1.047206
24	1	0	5.232480	-3.881927	1.936701
25	6	0	6.904229	-3.432834	0.647809
26	1	0	7.606723	-4.007627	1.241044
27	6	0	7.362229	-2.767605	-0.504902

28	1	0	8.409073	-2.840832	-0.777764
29	6	0	6.506884	-2.016057	-1.297416
30	1	0	6.863583	-1.495631	-2.178321
31	6	0	5.173914	-1.957673	-0.894465
32	6	0	2.532940	-2.694906	1.497602
33	1	0	1.970065	-1.823487	1.828660
34	1	0	3.181759	-3.001402	2.314387
35	1	0	1.870440	-3.522899	1.237543
36	6	0	4.135773	-0.607547	-2.725243
37	1	0	3.155868	-0.617264	-3.199611
38	1	0	4.838661	-1.129281	-3.374700
39	1	0	4.463085	0.421568	-2.573685
40	6	0	-0.103662	1.124475	-1.570383
41	6	0	-1.278315	0.292785	-1.772918
42	6	0	-2.644915	-1.313791	-2.489943
43	6	0	-3.226978	-2.415730	-3.112315
44	1	0	-2.700836	-2.994926	-3.861828
45	6	0	-4.513262	-2.739904	-2.708370
46	1	0	-5.006866	-3.596123	-3.154302
47	6	0	-5.191554	-1.990899	-1.727831
48	1	0	-6.192532	-2.288362	-1.435966
49	6	0	-4.611837	-0.890479	-1.115843
50	1	0	-5.127707	-0.328748	-0.346942
51	6	0	-3.317607	-0.571641	-1.520778
52	6	0	-0.492177	-1.061899	-3.739508
53	1	0	-1.025244	-0.913130	-4.680506
54	1	0	-0.171473	-2.100012	-3.672688
55	1	0	0.375467	-0.407621	-3.708134
56	6	0	-2.768950	1.441069	-0.110246
57	1	0	-1.862522	1.772119	0.393331
58	1	0	-3.442217	1.007793	0.628588
59	1	0	-3.252918	2.288492	-0.599201
60	6	0	-0.328367	2.560736	-1.682152
61	6	0	-0.375783	4.749011	-1.276960
62	6	0	-0.227326	6.031192	-0.749077
63	1	0	0.307669	6.212542	0.179961
64	6	0	-0.819196	7.061621	-1.464977
65	1	0	-0.735566	8.077112	-1.093459
66	6	0	-1.528827	6.824584	-2.658110
67	1	0	-1.976410	7.661529	-3.182764
68	6	0	-1.675115	5.546577	-3.177423
69	1	0	-2.230788	5.367719	-4.090229
70	6	0	-1.077807	4.511841	-2.458385
71	6	0	0.755056	3.335104	0.461013
72	1	0	0.490917	2.369359	0.891062
73	1	0	0.440792	4.128795	1.140594

74	1	0	1.834721	3.384122	0.324322
75	6	0	-1.529401	2.479249	-3.878440
76	1	0	-0.849936	1.677481	-4.163530
77	1	0	-1.559213	3.202921	-4.691364
78	1	0	-2.534543	2.089299	-3.707037
79	7	0	-0.453972	-1.855464	0.202422
80	7	0	0.469877	-3.168727	-1.291933
81	7	0	3.369963	-2.319191	0.365579
82	7	0	4.076426	-1.318434	-1.455242
83	7	0	-1.388974	-0.739009	-2.638005
84	7	0	-2.439161	0.418187	-1.097945
85	7	0	0.061886	3.514583	-0.812118
86	7	0	-1.016254	3.144621	-2.688527
87	15	0	1.522128	0.656328	-1.223352
88	9	0	2.387199	0.625794	1.909999
89	9	0	4.406761	0.664374	0.775110
90	9	0	3.626718	2.579622	1.806500
91	9	0	5.463831	1.467775	2.672531
92	9	0	4.225319	-0.485191	2.773177
93	9	0	3.443225	1.431124	3.805046
94	9	0	-5.505403	0.454118	1.788503
95	9	0	-4.728690	0.651387	3.957413
96	9	0	-6.641200	-0.580366	3.525260
97	9	0	-4.683642	-1.653000	4.156608
98	9	0	-3.555889	-0.616825	2.417961
99	9	0	-5.463078	-1.851351	1.989144
100	17	0	1.057514	6.447076	2.694249
101	15	0	3.931313	1.050400	2.294053
102	15	0	-5.103385	-0.601677	2.980228

23, ωB97x-D/6-31+G(d,p), benzene IEFPCM:
Sum of electronic and thermal Free Energies= -1266.746045

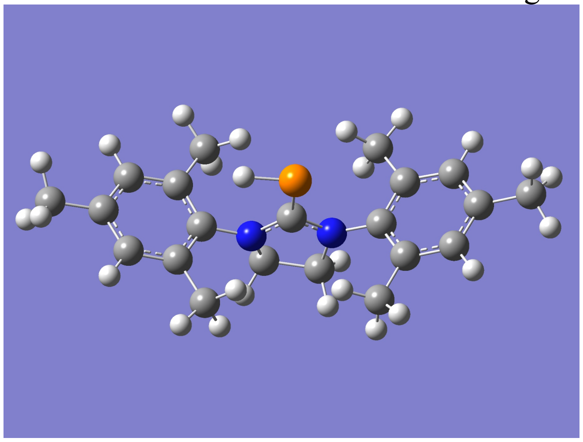

Center Number	Atomic Number	Atomic Type	Coordinates (Angstroms) X	Y	Z
1	15	0	7.808751	7.475026	12.037987
2	7	0	7.292684	4.904453	11.217244
3	6	0	7.884138	5.726695	12.131469
4	6	0	7.662141	3.505874	11.411423
5	1	0	8.430456	3.200964	10.688912
6	1	0	6.792239	2.855829	11.288922
7	6	0	6.608386	5.351548	10.050336
8	6	0	5.220468	5.531893	10.121226
9	6	0	4.547084	5.944043	8.972704
10	1	0	3.470065	6.090167	9.015464
11	6	0	5.223034	6.180155	7.773538
12	6	0	6.604837	5.991401	7.738520
13	1	0	7.145053	6.177619	6.812963
14	6	0	7.318399	5.579308	8.864992
15	6	0	4.490536	5.300507	11.417996
16	1	0	4.650064	4.283260	11.790699
17	1	0	3.416128	5.453980	11.293224
18	1	0	4.852128	5.990828	12.187472
19	6	0	4.478000	6.664004	6.554935
20	1	0	5.006752	6.402409	5.634487
21	1	0	4.370590	7.754052	6.577694
22	1	0	3.473210	6.234907	6.507022
23	6	0	8.812202	5.389291	8.806872
24	1	0	9.296587	5.818584	9.688633
25	1	0	9.228318	5.872357	7.919856
26	1	0	9.076768	4.326784	8.759599
27	1	0	8.522280	7.658015	13.252166
28	7	0	8.503176	4.930493	13.050861
29	6	0	8.201268	3.515047	12.843350
30	1	0	7.449723	3.168285	13.564384
31	1	0	9.102201	2.907880	12.962001
32	6	0	9.175206	5.377961	14.223349
33	6	0	10.559049	5.582241	14.161275
34	6	0	11.220498	5.994264	15.317765
35	1	0	12.294222	6.163748	15.280175
36	6	0	10.535524	6.204134	16.516193
37	6	0	9.154278	6.003111	16.539034
38	1	0	8.604963	6.181889	17.460718
39	6	0	8.454063	5.590641	15.405048
40	6	0	11.295128	5.388483	12.861797
41	1	0	11.166926	4.371223	12.477188
42	1	0	12.364491	5.573083	12.987514

43	1	0	10.912393	6.074481	12.098565
44	6	0	11.273837	6.620706	17.763457
45	1	0	10.638776	7.223550	18.418229
46	1	0	12.166209	7.203933	17.520819
47	1	0	11.597072	5.742299	18.333157
48	6	0	6.959582	5.402074	15.440225
49	1	0	6.482032	5.905034	14.593684
50	1	0	6.539677	5.809479	16.362701
51	1	0	6.689112	4.341417	15.393159

24, ωB97x-D/6-31+G(d,p), benzene IEFPCM:
Sum of electronic and thermal Free Energies= -2284.881287

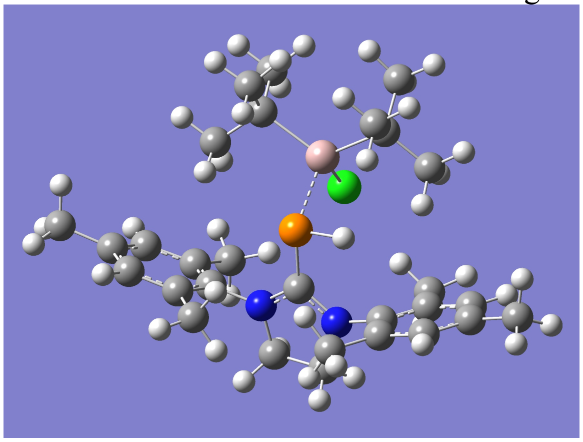

Center Number	Atomic Number	Atomic Type	Coordinates (Angstroms) X	Y	Z
1	15	0	10.617916	7.098156	4.507025
2	17	0	9.342318	10.698577	4.304605
3	13	0	11.212160	9.502458	4.175422
4	7	0	8.283206	7.047990	6.031473
5	7	0	7.908626	6.603295	3.914327
6	6	0	8.960332	7.157255	7.287548
7	6	0	9.648446	6.039899	7.788911
8	6	0	8.847333	6.934987	4.817732
9	6	0	8.837120	8.338138	8.030730
10	6	0	9.476662	8.404288	9.267984
11	1	0	9.407030	9.324906	9.842363
12	6	0	10.225553	7.341208	9.768582
13	6	0	9.695386	4.732055	7.043181
14	1	0	8.731844	4.496489	6.580664
15	1	0	9.956108	3.915602	7.720083
16	1	0	10.441368	4.765623	6.242637
17	6	0	10.288568	6.164494	9.020968
18	1	0	10.840478	5.313254	9.412442

19	6	0	8.107991	6.278904	2.535458
20	6	0	12.430825	10.010444	5.687469
21	6	0	6.827284	6.859411	5.977633
22	1	0	6.319137	7.804668	6.185947
23	1	0	6.523415	6.123364	6.725526
24	6	0	7.785100	7.232577	1.563134
25	6	0	11.903564	9.519442	2.282725
26	6	0	8.041948	9.512620	7.527796
27	1	0	8.130429	9.628003	6.445281
28	1	0	8.393160	10.438440	7.989209
29	1	0	6.980561	9.403404	7.780580
30	6	0	11.823392	9.606219	7.036468
31	1	0	10.861877	10.103103	7.206992
32	1	0	12.489516	9.891534	7.868862
33	1	0	11.655087	8.524894	7.114195
34	6	0	7.958883	6.883752	0.223739
35	1	0	7.727664	7.619412	-0.542644
36	6	0	10.967439	7.468288	11.073870
37	1	0	11.961281	7.897227	10.904932
38	1	0	11.103271	6.494915	11.552697
39	1	0	10.438773	8.124640	11.770298
40	6	0	6.598694	6.383048	4.539311
41	1	0	6.335284	5.321368	4.479788
42	1	0	5.830555	6.960077	4.021370
43	6	0	8.566729	4.998150	2.196293
44	6	0	8.436160	5.628727	-0.154112
45	6	0	13.790536	9.310889	5.531010
46	1	0	13.692438	8.217988	5.493124
47	1	0	14.450330	9.547566	6.382637
48	1	0	14.313409	9.630365	4.621475
49	6	0	7.281594	8.596368	1.948931
50	1	0	6.266673	8.545140	2.359063
51	1	0	7.254365	9.256225	1.079342
52	1	0	7.921090	9.061101	2.704240
53	6	0	10.767019	9.345737	1.265428
54	1	0	10.270382	8.373628	1.368365
55	1	0	11.156359	9.400992	0.234695
56	1	0	10.000031	10.121570	1.371060
57	6	0	8.727854	4.697624	0.844707
58	1	0	9.091371	3.711709	0.565171
59	6	0	12.657081	11.532244	5.702178
60	1	0	13.122872	11.893458	4.778004
61	1	0	13.323767	11.815835	6.534059
62	1	0	11.716868	12.080438	5.833849
63	6	0	12.946000	8.416572	2.032761
64	1	0	13.796766	8.480711	2.720412

65	1	0	13.349401	8.491872	1.009048
66	1	0	12.517777	7.410968	2.128582
67	6	0	12.574961	10.882928	2.036826
68	1	0	11.888448	11.719646	2.217181
69	1	0	12.921290	10.963143	0.992722
70	1	0	13.452665	11.027812	2.678205
71	6	0	8.906719	3.982904	3.256275
72	1	0	9.817015	4.268909	3.794419
73	1	0	9.074330	3.000581	2.809869
74	1	0	8.109013	3.883995	3.998977
75	6	0	8.660215	5.295922	-1.606914
76	1	0	7.953109	5.826480	-2.249924
77	1	0	8.555516	4.223406	-1.790961
78	1	0	9.670219	5.588567	-1.913963
79	1	0	10.490434	6.782398	3.135659

25, ωB97x-D/6-31+G(d,p), chloroform IEFPCM:
Sum of electronic and thermal Free Energies= -1077.694771

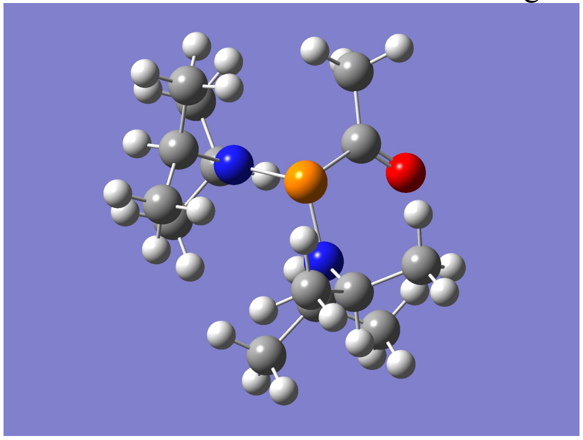

Center Number	Atomic Number	Atomic Type	Coordinates (Angstroms) X	Y	Z
1	15	0	0.037092	-0.158817	-0.852824
2	8	0	0.815849	-2.735699	-0.412235
3	7	0	-1.387385	0.233400	-0.010344
4	7	0	1.424831	0.187206	0.048111
5	6	0	-0.090270	-2.026541	-0.817497
6	6	0	-1.927524	-0.490322	1.155057
7	1	0	-1.280949	-1.362477	1.302522
8	6	0	2.586081	0.771614	-0.653450
9	1	0	3.345412	0.935102	0.116949
10	6	0	1.765386	1.119195	2.317597
11	1	0	2.710516	1.628841	2.100369
12	1	0	1.762252	0.869886	3.383994

13	1	0	0.951316	1.822007	2.118395
14	6	0	-1.888382	0.336669	2.446086
15	1	0	-0.879720	0.693763	2.660338
16	1	0	-2.226891	-0.270520	3.291803
17	1	0	-2.549260	1.207508	2.380097
18	6	0	2.756219	-1.131477	1.717723
19	1	0	2.616285	-2.029247	1.113270
20	1	0	2.777730	-1.412733	2.776025
21	1	0	3.729528	-0.689985	1.477186
22	6	0	-2.139645	1.431077	-0.438475
23	1	0	-2.964401	1.538618	0.271732
24	6	0	2.288219	2.140323	-1.268258
25	1	0	1.560692	2.058336	-2.082353
26	1	0	3.204955	2.575618	-1.679725
27	1	0	1.887385	2.821661	-0.512217
28	6	0	1.608130	-0.147262	1.467181
29	1	0	0.690088	-0.648892	1.783085
30	6	0	-3.346869	-1.019011	0.907393
31	1	0	-4.075177	-0.204351	0.837680
32	1	0	-3.653886	-1.661415	1.738636
33	1	0	-3.398999	-1.600042	-0.015375
34	6	0	3.187912	-0.191045	-1.681813
35	1	0	3.405921	-1.156941	-1.218740
36	1	0	4.114279	0.218534	-2.099155
37	1	0	2.489432	-0.359219	-2.509253
38	6	0	-1.311130	2.713521	-0.347399
39	1	0	-0.869740	2.817038	0.648387
40	1	0	-1.940605	3.587660	-0.544014
41	1	0	-0.500397	2.711497	-1.081839
42	6	0	-2.761553	1.254436	-1.825568
43	1	0	-1.983859	1.144113	-2.588540
44	1	0	-3.373713	2.124170	-2.086482
45	1	0	-3.396641	0.363759	-1.849645
46	6	0	-1.299634	-2.637679	-1.495235
47	1	0	-0.953995	-3.295401	-2.298159
48	1	0	-1.839089	-3.253668	-0.768915
49	1	0	-1.976803	-1.884045	-1.901607

26, ωB97x-D/6-31+G(d,p), chloroform IEFPCM:
Sum of electronic and thermal Free Energies= -613.312078

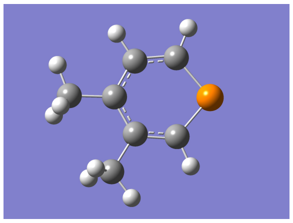

Center Number	Atomic Number	Atomic Type	Coordinates (Angstroms) X	Y	Z
1	6	0	-0.047963	-0.474897	0.033630
2	6	0	1.343657	-0.549086	0.089568
3	6	0	1.359542	2.101756	0.014697
4	6	0	-0.024617	1.976604	-0.034805
5	6	0	-0.722347	0.764198	-0.027402
6	1	0	1.783362	-1.544278	0.135072
7	1	0	1.777680	3.105645	0.002220
8	1	0	-0.615231	2.889753	-0.083107
9	15	0	2.465112	0.773275	0.095188
10	6	0	-0.853457	-1.753182	0.039069
11	1	0	-1.471632	-1.836531	-0.861355
12	1	0	-0.202363	-2.628342	0.084491
13	1	0	-1.532251	-1.790245	0.898028
14	6	0	-2.229164	0.791656	-0.086711
15	1	0	-2.602715	0.262100	-0.970046
16	1	0	-2.671622	0.303214	0.788384
17	1	0	-2.603364	1.816890	-0.125519

27 isomer A, ωB97x-D/6-31+G(d,p), benzene IEFPCM:
Sum of electronic and thermal Free Energies= -1241.879394

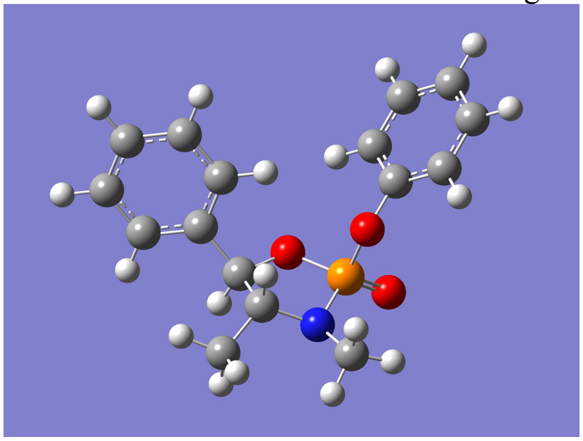

Center Number	Atomic Number	Atomic Type	Coordinates (Angstroms) X	Y	Z
1	6	0	-1.686903	0.740339	0.743030
2	6	0	-1.489265	1.745910	-0.419126
3	8	0	-0.382624	0.459431	1.300721
4	7	0	-0.274839	2.481391	-0.048252
5	15	0	0.756761	1.437595	0.718089
6	8	0	1.756991	1.937742	1.679212

7	8	0	1.422873	0.604146	-0.517125
8	6	0	-2.701859	2.645110	-0.598289
9	1	0	-3.593478	2.030795	-0.756341
10	1	0	-2.589610	3.293472	-1.470690
11	1	0	-2.852153	3.268778	0.288127
12	6	0	-2.354566	-0.538540	0.313070
13	6	0	-3.688539	-0.776752	0.640302
14	6	0	-1.656269	-1.478336	-0.451052
15	6	0	-4.325892	-1.937404	0.202061
16	1	0	-4.232607	-0.055729	1.244877
17	6	0	-2.288027	-2.640285	-0.880546
18	1	0	-0.614185	-1.301376	-0.702422
19	6	0	-3.626020	-2.871097	-0.557150
20	1	0	-5.364635	-2.113920	0.462616
21	1	0	-1.736456	-3.367701	-1.467697
22	1	0	-4.117919	-3.778471	-0.893057
23	1	0	-1.315226	1.185925	-1.352703
24	1	0	-2.265883	1.222462	1.537975
25	6	0	0.240070	3.472644	-0.983660
26	1	0	0.392505	3.045848	-1.984249
27	1	0	-0.456127	4.311489	-1.053827
28	1	0	1.192175	3.857486	-0.613061
29	6	0	2.392144	-0.372841	-0.347554
30	6	0	3.520681	-0.298549	-1.155771
31	6	0	2.211807	-1.422679	0.548721
32	6	0	4.489035	-1.294731	-1.062778
33	1	0	3.628352	0.534162	-1.842233
34	6	0	3.192692	-2.408285	0.637055
35	1	0	1.320740	-1.467928	1.166336
36	6	0	4.329913	-2.350501	-0.166077
37	1	0	5.372816	-1.240800	-1.690467
38	1	0	3.061151	-3.226119	1.338230
39	1	0	5.088630	-3.122606	-0.092857

----

27 isomer B, ωB97x-D/6-31+G(d,p), benzene IEFPCM:
Sum of electronic and thermal Free Energies=   -1241.875048

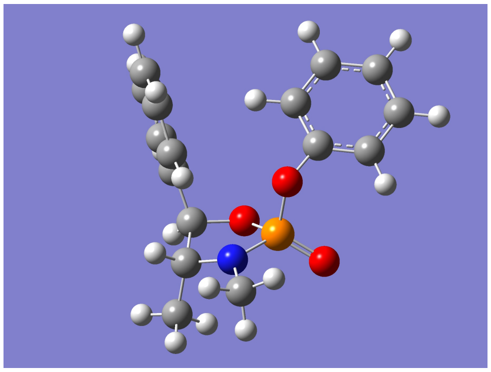

Center Number	Atomic Number	Atomic Type	Coordinates (Angstroms) X	Y	Z
1	6	0	-0.960721	0.401710	0.757167
2	6	0	-1.179649	1.403167	-0.401438
3	8	0	0.328112	0.744579	1.336040
4	7	0	0.170723	1.645427	-0.913985
5	15	0	1.332294	1.318691	0.213468
6	8	0	2.088738	0.025707	-0.415453
7	6	0	-1.884689	2.675990	0.064082
8	1	0	-2.894919	2.440121	0.412041
9	1	0	-1.973892	3.395486	-0.754078
10	1	0	-1.331477	3.148383	0.881838
11	6	0	-0.986715	-1.068068	0.394283
12	6	0	-1.273527	-1.993640	1.400674
13	6	0	-0.670810	-1.534264	-0.884522
14	6	0	-1.241632	-3.361192	1.139960
15	1	0	-1.512809	-1.640549	2.400278
16	6	0	-0.643837	-2.900972	-1.149811
17	1	0	-0.414163	-0.832627	-1.671556
18	6	0	-0.925985	-3.817694	-0.138557
19	1	0	-1.463720	-4.068053	1.933095
20	1	0	-0.390307	-3.249948	-2.145736
21	1	0	-0.900026	-4.882808	-0.346082
22	1	0	-1.777404	0.926179	-1.187647
23	1	0	-1.683388	0.589230	1.554179
24	6	0	0.378217	2.514558	-2.058974
25	1	0	-0.276005	2.198805	-2.877775
26	1	0	0.179344	3.567248	-1.827702
27	1	0	1.411279	2.427828	-2.401079
28	6	0	3.024952	-0.692856	0.320394
29	6	0	4.302703	-0.184353	0.524267
30	6	0	2.654982	-1.947809	0.792490
31	6	0	5.229132	-0.956800	1.221737
32	1	0	4.551640	0.803096	0.152994
33	6	0	3.592555	-2.710338	1.484820
34	1	0	1.647406	-2.309763	0.616185
35	6	0	4.879174	-2.218432	1.701065
36	1	0	6.228261	-0.567850	1.390344
37	1	0	3.312980	-3.690706	1.857521
38	1	0	5.606163	-2.815236	2.242413
39	8	0	2.274256	2.362722	0.670583

28, ωB97x-D/6-31+G(d,p), benzene IEFPCM:
Sum of electronic and thermal Free Energies= -1241.878821

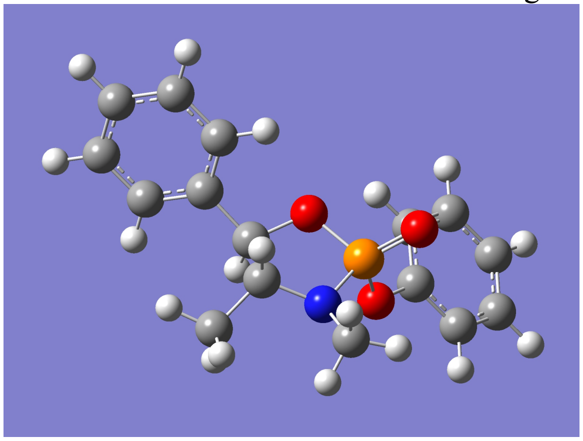

Center Number	Atomic Number	Atomic Type	Coordinates (Angstroms) X	Y	Z
1	6	0	1.393747	0.133008	-0.484580
2	6	0	1.512804	1.652715	-0.240925
3	8	0	0.354532	-0.329148	0.403325
4	7	0	0.133169	2.071789	0.024384
5	15	0	-0.761628	0.816358	0.627026
6	8	0	-1.875211	0.578995	-0.531680
7	8	0	-1.352876	0.871920	1.980185
8	6	0	-2.954739	-0.276286	-0.360358
9	6	0	-4.213787	0.216876	-0.681275
10	6	0	-2.777200	-1.592607	0.054140
11	6	0	-5.318431	-0.625182	-0.580814
12	1	0	-4.314494	1.247906	-1.002218
13	6	0	-3.891637	-2.421829	0.158492
14	1	0	-1.785125	-1.962657	0.291245
15	6	0	-5.162037	-1.944607	-0.158842
16	1	0	-6.304270	-0.244961	-0.828931
17	1	0	-3.761272	-3.447808	0.487429
18	1	0	-6.025166	-2.597007	-0.077342
19	6	0	2.138141	2.379315	-1.421134
20	1	0	3.135646	1.976240	-1.619828
21	1	0	2.248758	3.446237	-1.211779
22	1	0	1.519698	2.261152	-2.316175
23	6	0	2.668496	-0.621488	-0.223423
24	6	0	3.480573	-1.002025	-1.291780
25	6	0	3.075072	-0.902117	1.083298
26	6	0	4.695029	-1.645912	-1.059664
27	1	0	3.162314	-0.798299	-2.310953
28	6	0	4.282980	-1.552806	1.315012
29	1	0	2.435689	-0.622191	1.914933

30	6	0	5.097624	-1.922555	0.244445
31	1	0	5.319758	-1.938068	-1.897767
32	1	0	4.588851	-1.773808	2.332726
33	1	0	6.039559	-2.429960	0.427335
34	1	0	2.128898	1.819614	0.657975
35	1	0	1.058214	-0.038327	-1.515382
36	6	0	-0.110087	3.444686	0.441737
37	1	0	0.524581	3.721296	1.293729
38	1	0	0.083008	4.130948	-0.387042
39	1	0	-1.153619	3.558992	0.740122

29, ωB97x-D/6-31+G(d,p), chloroform IEFPCM:
Sum of electronic and thermal Free Energies= -2861.759296

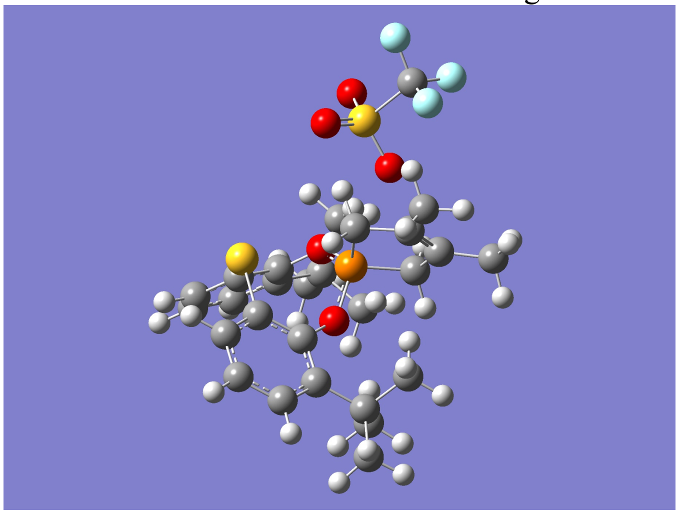

Center Number	Atomic Number	Atomic Type	Coordinates (Angstroms) X	Y	Z
1	8	0	-0.373423	1.182682	0.220760
2	8	0	1.897796	0.014829	-0.058454
3	16	0	0.164539	0.259397	-2.566556
4	6	0	-0.015672	2.221131	-0.636269
5	6	0	0.285084	1.933006	-1.969066
6	6	0	0.620441	2.967175	-2.840658
7	1	0	0.849384	2.740733	-3.876240
8	6	0	0.641236	4.269146	-2.366921
9	6	0	0.322415	4.532242	-1.037053
10	1	0	0.338019	5.562837	-0.707412
11	6	0	-0.012609	3.527854	-0.121969
12	6	0	2.519470	-0.414578	-1.229006
13	6	0	1.857429	-0.295286	-2.456538
14	6	0	2.507996	-0.693746	-3.622954
15	1	0	1.995721	-0.606796	-4.574811

16	6	0	3.802579	-1.185533	-3.548067
17	6	0	4.447521	-1.268206	-2.318239
18	1	0	5.462856	-1.641890	-2.301071
19	6	0	3.836836	-0.887067	-1.117287
20	6	0	-0.329902	3.850758	1.348766
21	6	0	-0.355348	5.368291	1.597929
22	1	0	0.613994	5.839669	1.405769
23	1	0	-0.601527	5.546313	2.648791
24	1	0	-1.117506	5.868395	0.991795
25	6	0	-1.712701	3.296575	1.747557
26	1	0	-2.491860	3.708324	1.097893
27	1	0	-1.939223	3.598712	2.775510
28	1	0	-1.769906	2.209175	1.697840
29	6	0	0.774045	3.260129	2.248182
30	1	0	0.839905	2.175239	2.164021
31	1	0	0.562943	3.495309	3.296484
32	1	0	1.751360	3.681918	1.991694
33	6	0	4.576096	-0.994151	0.229852
34	6	0	6.026158	-1.470657	0.038605
35	1	0	6.078668	-2.471508	-0.401272
36	1	0	6.511506	-1.519528	1.017741
37	1	0	6.606586	-0.781273	-0.582826
38	6	0	3.873263	-2.029316	1.130423
39	1	0	3.862641	-3.013494	0.650945
40	1	0	2.842615	-1.758542	1.358696
41	1	0	4.412283	-2.119581	2.079332
42	6	0	4.641286	0.381357	0.926479
43	1	0	5.142385	1.112831	0.284165
44	1	0	5.219679	0.292304	1.851860
45	1	0	3.655533	0.771342	1.181188
46	6	0	0.544076	-0.477868	2.232106
47	6	0	-0.306854	-1.756241	-0.027605
48	6	0	0.353229	-1.981439	2.320666
49	1	0	-0.247617	0.064102	2.758192
50	1	0	1.518941	-0.147091	2.605836
51	6	0	-0.073149	-2.606149	1.210086
52	1	0	-1.368662	-1.623059	-0.273158
53	1	0	0.208529	-2.152390	-0.907752
54	15	0	0.400018	-0.181308	0.467241
55	6	0	-0.349257	-4.068783	1.039202
56	1	0	0.314336	-4.494251	0.277516
57	1	0	-0.215197	-4.632667	1.962597
58	1	0	-1.377003	-4.218807	0.692995
59	6	0	0.649107	-2.596070	3.653968
60	1	0	0.482300	-3.673291	3.668324
61	1	0	1.691921	-2.407587	3.935064

62	1	0	0.018307	-2.142439	4.426369
63	1	0	4.319007	-1.495338	-4.450025
64	1	0	0.894502	5.087306	-3.032312
65	16	0	-3.628515	-0.395644	1.058532
66	8	0	-2.379423	-0.193845	1.814829
67	8	0	-4.630294	0.662844	1.207214
68	8	0	-3.422395	-0.893304	-0.312031
69	6	0	-4.395362	-1.852923	1.925450
70	9	0	-4.628179	-1.577688	3.215530
71	9	0	-5.559069	-2.194732	1.358609
72	9	0	-3.583745	-2.920402	1.874043

----

anti-**30**, ωB97x-D/6-31+G(d,p), toluene IEFPCM:
Sum of electronic and thermal Free Energies= -695.438255

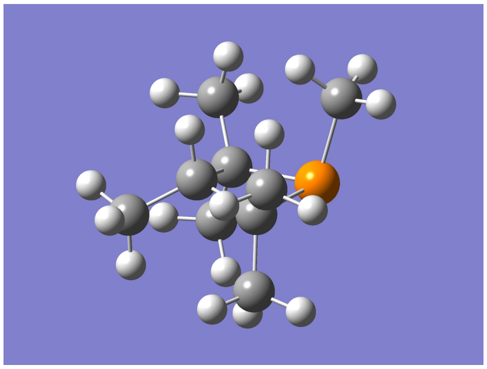

Center Number	Atomic Number	Atomic Type	Coordinates (Angstroms) X	Y	Z
1	6	0	-0.630691	1.058067	0.235324
2	6	0	0.792968	0.497920	-0.074376
3	6	0	-0.393750	2.554804	-0.138614
4	1	0	-0.753770	1.021165	1.326139
5	6	0	1.326506	-0.465236	0.982575
6	1	0	0.743713	-1.395373	0.966095
7	1	0	2.372895	-0.727907	0.789737
8	1	0	1.261215	-0.054191	1.993800
9	6	0	0.940807	-0.153846	-1.453632
10	1	0	1.993139	-0.388210	-1.646557
11	1	0	0.372632	-1.091116	-1.498417
12	1	0	0.594371	0.482737	-2.271694
13	6	0	-0.845953	2.943351	-1.550543
14	1	0	-1.940090	2.916502	-1.624483
15	1	0	-0.517332	3.962081	-1.782369
16	1	0	-0.445504	2.288001	-2.328128
17	6	0	-0.988325	3.547101	0.857040
18	1	0	-0.686556	4.574457	0.623419

19	1	0	-2.084455	3.506457	0.813148
20	1	0	-0.692279	3.333215	1.887782
21	6	0	-1.830059	0.346796	-0.381427
22	1	0	-1.879786	-0.692963	-0.040166
23	1	0	-2.763777	0.838993	-0.088245
24	1	0	-1.784798	0.338514	-1.474605
25	15	0	1.487237	2.269548	-0.102730
26	6	0	1.911109	2.569323	1.677838
27	1	0	2.885308	2.117655	1.886855
28	1	0	2.002534	3.647382	1.839658
29	1	0	1.182904	2.170941	2.390103

----

syn-**30** isomer A, ωB97x-D/6-31+G(d,p), toluene IEFPCM:
Sum of electronic and thermal Free Energies= -695.437383

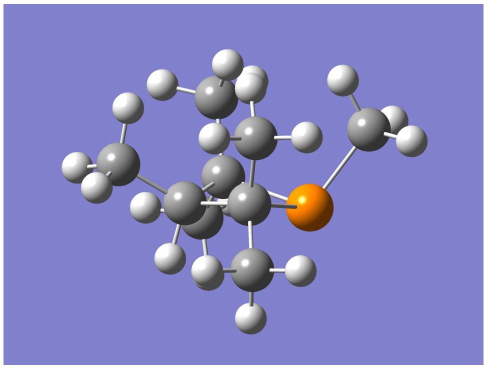

Center Number	Atomic Number	Atomic Type	Coordinates (Angstroms) X	Y	Z
1	6	0	-0.611614	1.068405	0.219093
2	6	0	0.840202	0.522562	0.016635
3	6	0	-0.350884	2.587495	-0.048090
4	1	0	-0.840651	0.969834	1.289081
5	6	0	1.259784	-0.495389	1.076931
6	1	0	0.655114	-1.408056	0.992073
7	1	0	2.311505	-0.779935	0.957865
8	1	0	1.132598	-0.096836	2.088438
9	6	0	1.113546	-0.056964	-1.371814
10	1	0	2.185196	-0.237245	-1.509384
11	1	0	0.598652	-1.018865	-1.485092
12	1	0	0.779080	0.588152	-2.187182
13	6	0	-0.678281	3.048561	-1.468940
14	1	0	-1.765350	3.078403	-1.612578
15	1	0	-0.293904	4.059333	-1.643581
16	1	0	-0.266361	2.399303	-2.244673
17	6	0	-1.049501	3.508918	0.951689
18	1	0	-0.765048	4.555108	0.790884

19	1	0	-2.139656	3.438333	0.840568
20	1	0	-0.795907	3.247338	1.983895
21	6	0	-1.741378	0.392519	-0.548468
22	1	0	-1.816873	-0.664144	-0.270138
23	1	0	-2.701049	0.867214	-0.317337
24	1	0	-1.594786	0.443810	-1.631461
25	15	0	1.482248	2.279726	0.331501
26	6	0	2.445985	2.788751	-1.165195
27	1	0	2.562279	3.876648	-1.149573
28	1	0	3.446613	2.350949	-1.099156
29	1	0	1.999170	2.498160	-2.118842

----

syn-**30** isomer B, ωB97x-D/6-31+G(d,p), toluene IEFPCM:
Sum of electronic and thermal Free Energies= -695.434446

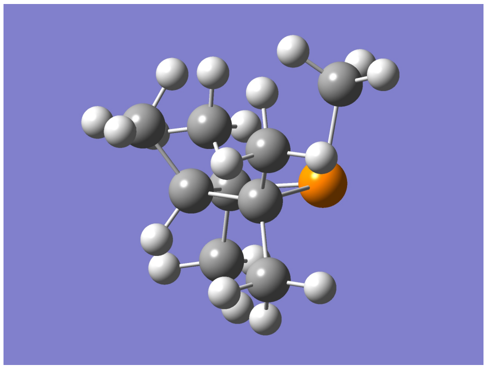

Center Number	Atomic Number	Atomic Type	Coordinates (Angstroms) X	Y	Z
1	6	0	-0.611693	1.069227	0.236381
2	6	0	0.838061	0.523482	-0.024175
3	6	0	-0.350079	2.583643	-0.088559
4	6	0	1.450601	-0.369189	1.052530
5	1	0	0.931069	-1.334806	1.088654
6	1	0	2.502784	-0.570761	0.821950
7	1	0	1.408203	0.065788	2.053344
8	6	0	0.934660	-0.202512	-1.370912
9	1	0	1.974027	-0.457755	-1.604605
10	1	0	0.353089	-1.132920	-1.336573
11	1	0	0.548565	0.398682	-2.199384
12	6	0	-0.892766	2.966604	-1.470155
13	1	0	-1.989873	2.929291	-1.464434
14	1	0	-0.586958	3.982434	-1.743332
15	1	0	-0.542844	2.293782	-2.258641
16	6	0	-0.844403	3.610203	0.927862
17	1	0	-0.484141	4.609935	0.660145
18	1	0	-1.940913	3.646019	0.931665

19	1	0	-0.517597	3.402675	1.949036
20	15	0	1.524378	2.288550	-0.170545
21	6	0	2.092130	2.669904	1.553751
22	1	0	3.081929	2.227540	1.700084
23	1	0	2.194991	3.754371	1.654729
24	1	0	1.429986	2.308459	2.344553
25	6	0	-1.198921	0.773967	1.614362
26	1	0	-2.191431	1.224832	1.711603
27	1	0	-1.307924	-0.305184	1.760463
28	1	0	-0.581163	1.156912	2.431666
29	1	0	-1.307874	0.644640	-0.501795

----

anti-**30[O]**, ωB97x-D/6-31+G(d,p), toluene IEFPCM:
Sum of electronic and thermal Free Energies= -770.679104

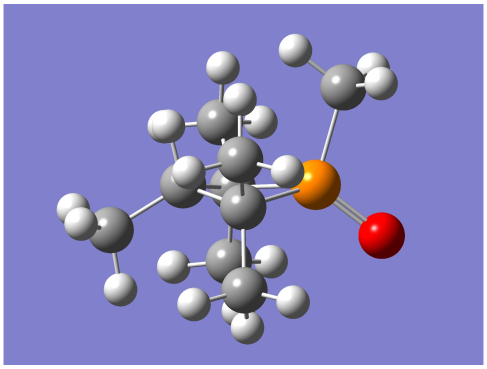

Center Number	Atomic Number	Atomic Type	Coordinates (Angstroms) X	Y	Z
1	6	0	-0.539348	1.101062	-0.095574
2	6	0	0.937313	0.560162	-0.114248
3	6	0	-0.265573	2.647789	-0.176689
4	15	0	1.485737	2.280048	0.315196
5	6	0	1.610171	2.405881	2.128312
6	1	0	2.536930	1.923162	2.448182
7	1	0	1.649358	3.463056	2.402343
8	1	0	0.765121	1.933920	2.634751
9	8	0	2.648598	2.928348	-0.386918
10	1	0	-0.943221	0.898187	0.905374
11	6	0	-1.509087	0.511788	-1.111288
12	1	0	-1.617211	-0.566809	-0.957428
13	1	0	-2.499568	0.965893	-1.004302
14	1	0	-1.174625	0.673021	-2.139728
15	6	0	1.205580	-0.556856	0.892524
16	1	0	0.669948	-1.464465	0.589008
17	1	0	2.271974	-0.801076	0.938487
18	1	0	0.871583	-0.298524	1.901679

19	6	0	1.450146	0.136706	-1.494966
20	1	0	2.522681	-0.071666	-1.446429
21	1	0	0.939012	-0.776901	-1.818563
22	1	0	1.304600	0.896010	-2.265560
23	6	0	-1.121863	3.483969	0.772504
24	1	0	-0.798142	4.529957	0.780306
25	1	0	-2.167730	3.462618	0.443102
26	1	0	-1.092139	3.110860	1.800393
27	6	0	-0.340079	3.241357	-1.587717
28	1	0	-1.378236	3.240027	-1.938330
29	1	0	0.014604	4.275865	-1.576011
30	1	0	0.264270	2.702271	-2.319632

----

syn-**30[O]** isomer A, ωB97x-D/6-31+G(d,p), toluene IEFPCM:
Sum of electronic and thermal Free Energies= -770.677702

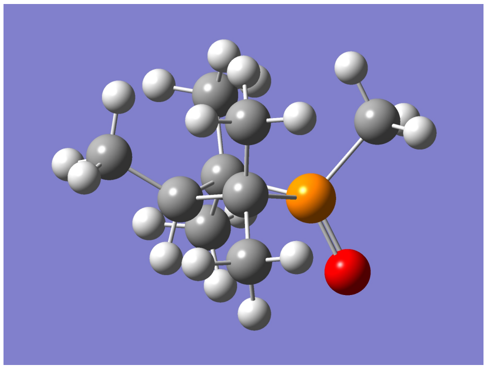

Center Number	Atomic Number	Atomic Type	Coordinates (Angstroms) X	Y	Z
1	6	0	-0.000384	1.224138	-0.489904
2	6	0	1.205244	0.293614	-0.092623
3	6	0	-1.205434	0.292859	-0.092645
4	1	0	-0.000395	1.287408	-1.585377
5	6	0	2.293282	0.213180	-1.159993
6	1	0	2.744178	1.202508	-1.305399
7	1	0	3.087968	-0.479385	-0.864011
8	1	0	1.889930	-0.128712	-2.116636
9	6	0	1.824895	0.602422	1.273351
10	1	0	2.523438	-0.188681	1.564500
11	1	0	2.386808	1.541811	1.223622
12	1	0	1.086752	0.706654	2.073084
13	6	0	-1.825273	0.601311	1.273324
14	1	0	-2.387774	1.540346	1.223561
15	1	0	-2.523317	-0.190220	1.564509
16	1	0	-1.087189	0.706044	2.073046
17	6	0	-2.293420	0.211756	-1.160016

18	1	0	-3.087772	-0.481157	-0.863954
19	1	0	-2.744799	1.200843	-1.305565
20	1	0	-1.889885	-0.130079	-2.116601
21	6	0	-0.000826	2.645199	0.056711
22	1	0	0.883026	3.189416	-0.291751
23	1	0	-0.885058	3.188846	-0.291676
24	1	0	-0.000788	2.671259	1.150105
25	15	0	0.000353	-1.120017	-0.095473
26	8	0	0.000685	-2.020021	-1.304581
27	6	0	0.000603	-2.061558	1.463632
28	1	0	-0.886847	-2.699331	1.481175
29	1	0	0.888583	-2.698587	1.481364
30	1	0	0.000238	-1.416095	2.343722

----

syn-**30[O]** isomer B, ωB97x-D/6-31+G(d,p), toluene IEFPCM:
Sum of electronic and thermal Free Energies= -770.676788

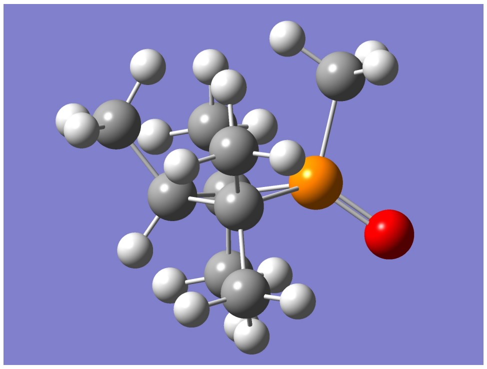

Center Number	Atomic Number	Atomic Type	Coordinates (Angstroms) X	Y	Z
1	6	0	0.000000	1.260582	-0.454076
2	6	0	1.207329	0.248042	-0.307457
3	6	0	-1.207329	0.248042	-0.307457
4	1	0	0.000000	1.672687	-1.470799
5	6	0	0.000000	-1.057196	2.087666
6	1	0	-0.889073	-1.592855	2.429592
7	1	0	0.889073	-1.592855	2.429592
8	1	0	0.000000	-0.051781	2.514186
9	15	0	0.000000	-1.034752	0.265901
10	6	0	2.328858	0.635551	0.656057
11	1	0	3.027664	-0.198625	0.776493
12	1	0	2.894405	1.484765	0.255591
13	1	0	1.969857	0.916251	1.648984
14	6	0	1.816332	-0.117912	-1.665954
15	1	0	2.334744	0.755945	-2.078151
16	1	0	2.540374	-0.931038	-1.559962

17	1	0	1.068196	-0.440231	-2.393384
18	6	0	0.000000	2.436678	0.517676
19	1	0	0.883668	3.062067	0.360570
20	1	0	-0.883668	3.062067	0.360570
21	1	0	0.000000	2.125031	1.566967
22	6	0	-1.816332	-0.117912	-1.665954
23	1	0	-2.540374	-0.931038	-1.559962
24	1	0	-2.334744	0.755945	-2.078151
25	1	0	-1.068196	-0.440231	-2.393384
26	6	0	-2.328858	0.635551	0.656057
27	1	0	-2.894405	1.484765	0.255590
28	1	0	-3.027664	-0.198625	0.776493
29	1	0	-1.969857	0.916251	1.648984
30	8	0	0.000000	-2.420721	-0.322210

----

anti-**31**, ωB97x-D/6-31+G(d,p), toluene IEFPCM:
Sum of electronic and thermal Free Energies= -1287.726518

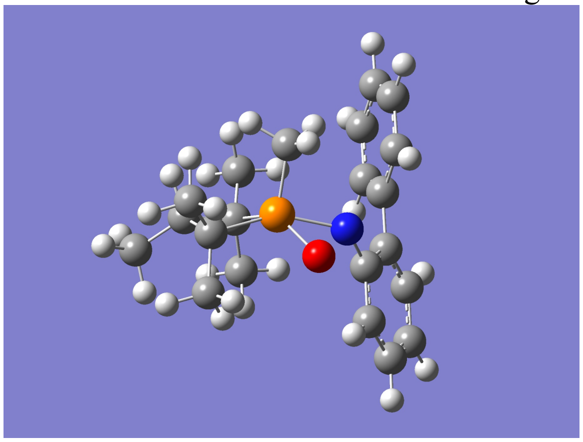

Center Number	Atomic Number	Atomic Type	Coordinates (Angstroms) X	Y	Z
1	6	0	-2.783144	-0.870265	1.061581
2	6	0	-3.190389	-0.168031	-0.273929
3	6	0	-1.240440	-0.580427	1.061220
4	15	0	-1.373916	-0.223395	-0.765400
5	6	0	-1.165217	-1.762213	-1.709711
6	1	0	-1.389460	-1.548535	-2.757731
7	1	0	-0.133201	-2.103865	-1.622871
8	1	0	-1.845584	-2.535788	-1.350342
9	8	0	-1.181065	1.140922	-1.637732
10	7	0	0.190949	0.564766	-1.338164
11	6	0	0.983002	1.505718	-0.679135
12	6	0	2.212470	1.082372	-0.112449
13	6	0	0.622757	2.859346	-0.604658

14	6	0	3.024173	2.039920	0.501740
15	6	0	1.454821	3.785254	0.014658
16	1	0	-0.322066	3.168888	-1.033526
17	6	0	2.664130	3.383175	0.574262
18	1	0	3.974171	1.719377	0.920462
19	1	0	1.151921	4.827488	0.056129
20	1	0	3.322783	4.100945	1.051847
21	6	0	2.640057	-0.342771	-0.105924
22	6	0	3.183844	-0.899883	1.060009
23	6	0	2.526615	-1.161103	-1.238243
24	6	0	3.594265	-2.230255	1.099173
25	1	0	3.259333	-0.290786	1.956336
26	6	0	2.933994	-2.491694	-1.199529
27	1	0	2.109262	-0.748496	-2.149971
28	6	0	3.465781	-3.034976	-0.030456
29	1	0	4.002675	-2.640117	2.017984
30	1	0	2.839315	-3.106367	-2.089981
31	1	0	3.778138	-4.074249	-0.001691
32	1	0	-2.906431	-1.951460	0.912834
33	6	0	-3.558678	-0.492561	2.317381
34	1	0	-4.619261	-0.736170	2.196383
35	1	0	-3.185607	-1.047426	3.184525
36	1	0	-3.482845	0.574708	2.542557
37	6	0	-4.218201	-0.934025	-1.105746
38	1	0	-5.197459	-0.904821	-0.611425
39	1	0	-4.337156	-0.485225	-2.097775
40	1	0	-3.952243	-1.986472	-1.239493
41	6	0	-3.679800	1.275534	-0.096646
42	1	0	-3.802272	1.750062	-1.074629
43	1	0	-4.652751	1.285482	0.408410
44	1	0	-2.996606	1.901259	0.482005
45	6	0	-0.385826	-1.796172	1.419708
46	1	0	0.672015	-1.617844	1.211579
47	1	0	-0.486688	-2.004841	2.491823
48	1	0	-0.694319	-2.694430	0.876977
49	6	0	-0.805820	0.614483	1.917440
50	1	0	-1.026244	0.403644	2.969750
51	1	0	0.270725	0.775592	1.824398
52	1	0	-1.299906	1.551074	1.650967

----

syn-**31**, ωB97x-D/6-31+G(d,p), toluene IEFPCM:
Sum of electronic and thermal Free Energies= -1287.724659

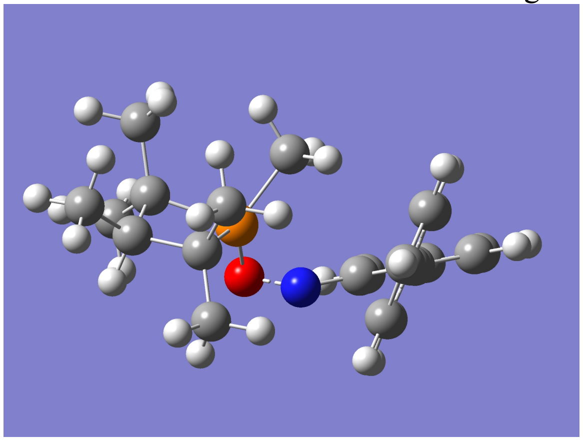

Center Number	Atomic Number	Atomic Type	Coordinates (Angstroms) X	Y	Z
1	6	0	-3.144113	-0.463927	-1.011130
2	6	0	-0.958369	0.091277	0.981992
3	15	0	-1.296184	-0.091575	-0.799778
4	6	0	-3.892664	-0.825482	0.275321
5	1	0	-4.011524	0.055188	0.915289
6	1	0	-4.895963	-1.193934	0.031565
7	1	0	-3.398799	-1.605744	0.862501
8	6	0	-3.953255	0.574617	-1.788227
9	1	0	-4.925260	0.153781	-2.076455
10	1	0	-4.142619	1.465064	-1.179153
11	1	0	-3.437941	0.893515	-2.698105
12	8	0	-1.079301	1.220131	-1.731060
13	7	0	0.298221	0.655850	-1.411642
14	6	0	1.050422	1.588248	-0.697234
15	6	0	2.194379	1.117900	-0.006925
16	6	0	0.730936	2.952277	-0.644622
17	6	0	2.969139	2.028941	0.711148
18	6	0	1.530498	3.839506	0.070337
19	1	0	-0.152479	3.302040	-1.165054
20	6	0	2.653821	3.386296	0.756379
21	1	0	3.845009	1.659137	1.238025
22	1	0	1.266381	4.892894	0.091480
23	1	0	3.278411	4.074953	1.315687
24	6	0	2.558085	-0.326350	-0.000874
25	6	0	2.806990	-1.019916	-1.190969
26	6	0	2.681143	-1.015729	1.211611
27	6	0	3.168821	-2.363768	-1.168064
28	1	0	2.708947	-0.497015	-2.136904
29	6	0	3.036082	-2.363613	1.236527

30	1	0	2.487945	-0.489352	2.142748
31	6	0	3.280411	-3.042777	0.045002
32	1	0	3.361098	-2.884243	-2.101614
33	1	0	3.120015	-2.881551	2.187145
34	1	0	3.556941	-4.092422	0.060403
35	6	0	-1.210061	-1.788688	-1.536861
36	6	0	-2.736909	-1.704838	-1.876966
37	1	0	-2.803142	-1.396359	-2.928140
38	6	0	-0.337673	-1.951302	-2.776914
39	1	0	0.721280	-1.962922	-2.515283
40	1	0	-0.589064	-2.902657	-3.262846
41	1	0	-0.501356	-1.143308	-3.495126
42	6	0	-0.818542	-2.822414	-0.474384
43	1	0	-1.435922	-2.769122	0.427761
44	1	0	-0.918399	-3.833330	-0.884691
45	1	0	0.227332	-2.680905	-0.180088
46	6	0	-3.549662	-2.984335	-1.725870
47	1	0	-4.595340	-2.810075	-1.999640
48	1	0	-3.158952	-3.764835	-2.387293
49	1	0	-3.530986	-3.373410	-0.703823
50	1	0	-0.008987	-0.390198	1.223741
51	1	0	-0.870742	1.159104	1.199298
52	1	0	-1.755499	-0.332353	1.590573

----

32 isomer A, ωB97x-D/6-31+G(d,p), $CHCl_3$ IEFPCM:
Sum of electronic and thermal Free Energies= -882.232307

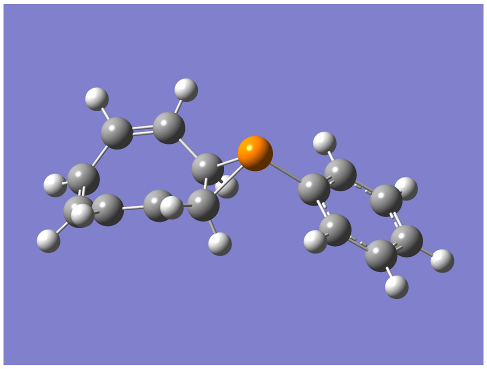

Center Number	Atomic Number	Atomic Type	Coordinates (Angstroms) X	Y	Z
1	6	0	-0.844952	-0.785229	0.028037
2	6	0	-1.965864	-1.604685	-0.508771
3	6	0	-3.188442	1.617970	-0.020004
4	6	0	-3.262499	-1.567644	-0.165760
5	6	0	-3.925108	0.673295	0.818599
6	6	0	-3.956487	-0.670286	0.756978

7	1	0	-1.658879	-2.408304	-1.175680
8	1	0	-0.323272	-1.280068	0.848555
9	1	0	-3.891608	-2.358055	-0.574590
10	1	0	-4.666863	-1.169560	1.414955
11	6	0	-0.810065	0.710571	0.095241
12	1	0	-0.265190	1.105159	0.954141
13	6	0	-1.890958	1.626206	-0.361863
14	1	0	-1.546191	2.472390	-0.953417
15	15	0	0.335844	-0.008685	-1.197824
16	1	0	-4.612722	1.142835	1.521124
17	1	0	-3.779612	2.470662	-0.353388
18	6	0	1.925130	-0.084733	-0.264861
19	6	0	2.607960	1.091963	0.066395
20	6	0	2.512680	-1.315794	0.050510
21	6	0	3.840675	1.040002	0.714602
22	1	0	2.171188	2.057641	-0.175738
23	6	0	3.745620	-1.369568	0.698397
24	1	0	2.001634	-2.240887	-0.204160
25	6	0	4.411707	-0.191431	1.032751
26	1	0	4.354489	1.961292	0.971429
27	1	0	4.185241	-2.331887	0.942265
28	1	0	5.371766	-0.232727	1.537640

----

32 isomer B, ωB97x-D/6-31+G(d,p), $CHCl_3$ IEFPCM:
Sum of electronic and thermal Free Energies= -882.231109

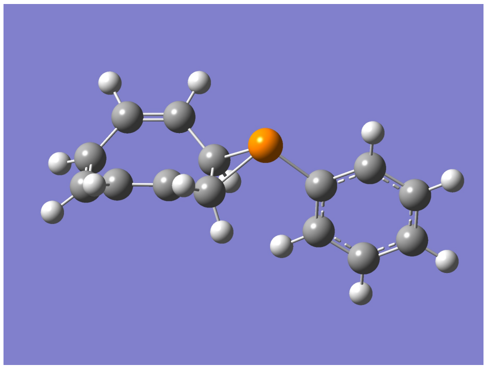

Center Number	Atomic Number	Atomic Type	Coordinates (Angstroms) X	Y	Z
1	6	0	-0.843562	-0.748580	0.100470
2	6	0	-1.935717	-1.621165	-0.411829
3	6	0	-3.239905	1.595879	-0.105328
4	6	0	-3.239955	-1.596361	-0.098028
5	6	0	-3.975240	0.674412	0.759551
6	6	0	-3.975243	-0.670938	0.762651
7	1	0	-1.595982	-2.451433	-1.028107

8	1	0	-0.320721	-1.200809	0.944988
9	1	0	-3.841369	-2.421631	-0.478579
10	1	0	-4.690258	-1.154138	1.427578
11	6	0	-0.843535	0.748922	0.097117
12	1	0	-0.320630	1.204900	0.939577
13	6	0	-1.935642	1.619238	-0.419134
14	1	0	-1.595840	2.446725	-1.039106
15	15	0	0.329289	-0.002669	-1.148620
16	1	0	-4.690256	1.160674	1.422239
17	1	0	-3.841271	2.419444	-0.489626
18	6	0	1.957251	-0.000712	-0.281719
19	6	0	2.155845	0.001678	1.105417
20	6	0	3.084663	-0.001845	-1.114178
21	6	0	3.441258	0.002842	1.641206
22	1	0	1.311319	0.002670	1.789189
23	6	0	4.372510	-0.000648	-0.580250
24	1	0	2.956787	-0.003683	-2.194049
25	6	0	4.554047	0.001673	0.800471
26	1	0	3.573274	0.004656	2.718821
27	1	0	5.231295	-0.001548	-1.244230
28	1	0	5.554863	0.002575	1.220565

33, ωB97x-D/6-31+G(d,p), $CHCl_3$ IEFPCM:
Sum of electronic and thermal Free Energies= -882.251616

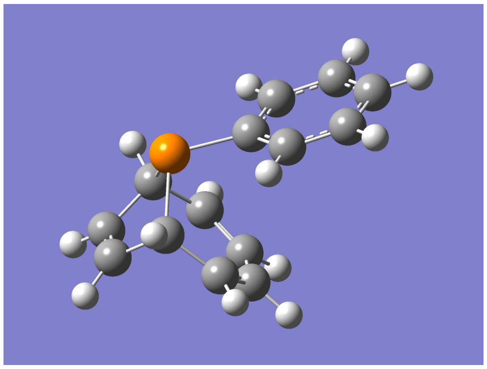

Center Number	Atomic Number	Atomic Type	Coordinates (Angstroms) X	Y	Z
1	6	0	0.142128	0.750040	-0.076704
2	6	0	1.419703	0.404682	-0.268805
3	6	0	0.775235	3.734204	-0.809625
4	6	0	2.445227	1.265765	0.444153
5	6	0	2.171352	3.358059	-1.016807
6	6	0	2.902015	2.350582	-0.496189
7	1	0	1.736403	-0.375882	-0.954354
8	1	0	-0.692499	0.280770	-0.589273

9	1	0	3.303608	0.673692	0.769328
10	1	0	3.946191	2.300149	-0.799694
11	6	0	-0.083618	1.950291	0.823419
12	1	0	-0.995313	1.837739	1.414314
13	6	0	-0.183065	3.183611	-0.036137
14	1	0	-1.154066	3.675741	-0.040900
15	15	0	1.424203	1.785273	1.937974
16	6	0	1.949058	3.496985	2.335320
17	6	0	1.009100	4.459476	2.722032
18	6	0	3.307985	3.828712	2.390925
19	6	0	1.414914	5.725710	3.137876
20	1	0	-0.052325	4.226664	2.693031
21	6	0	3.716322	5.094247	2.806206
22	1	0	4.059341	3.098545	2.101029
23	6	0	2.770450	6.048075	3.178777
24	1	0	0.670614	6.461005	3.428178
25	1	0	4.774603	5.335147	2.836833
26	1	0	3.087687	7.034961	3.501071
27	1	0	2.703929	4.019004	-1.697385
28	1	0	0.475126	4.618339	-1.368105

34, ωB97x-D/6-31+G(d,p), $CHCl_3$ IEFPCM:
Sum of electronic and thermal Free Energies= -882.251431

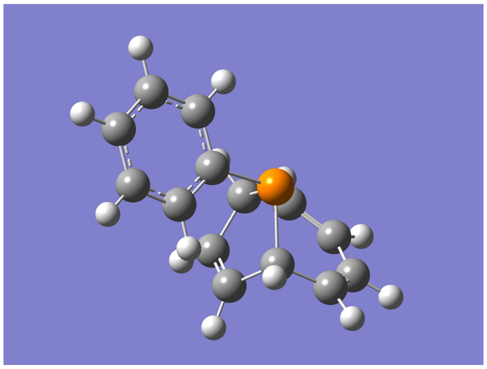

Center Number	Atomic Number	Atomic Type	Coordinates (Angstroms) X	Y	Z
1	6	0	-0.501752	0.870877	-0.140500
2	6	0	0.708097	0.330377	-0.305513
3	6	0	0.511515	3.638712	-1.181574
4	6	0	1.847434	1.108705	0.325057
5	6	0	1.844041	3.047828	-1.355779
6	6	0	2.438334	2.001853	-0.746362
7	1	0	0.905070	-0.536528	-0.930111
8	1	0	-1.403160	0.491129	-0.613202
9	1	0	2.631802	0.466044	0.732649

10	1	0	3.444646	1.749893	-1.074222
11	6	0	-0.524798	2.168958	0.640665
12	1	0	-1.398376	2.255771	1.291598
13	6	0	-0.502553	3.304251	-0.358018
14	1	0	-1.412510	3.896285	-0.431786
15	15	0	1.052496	2.142339	1.671440
16	1	0	2.439928	3.545509	-2.117885
17	1	0	0.323472	4.482385	-1.842055
18	6	0	0.569649	0.880201	2.928234
19	6	0	-0.137373	1.358142	4.041005
20	6	0	0.884236	-0.482523	2.876967
21	6	0	-0.539879	0.498694	5.060423
22	1	0	-0.374907	2.417232	4.114602
23	6	0	0.496640	-1.342267	3.903965
24	1	0	1.426520	-0.891704	2.031275
25	6	0	-0.220643	-0.856734	4.995261
26	1	0	-1.092984	0.889579	5.908946
27	1	0	0.751970	-2.396029	3.845880
28	1	0	-0.524776	-1.528769	5.791696

33[O], ωB97x-D/6-31+G(d,p), $CHCl_3$ IEFPCM:
Sum of electronic and thermal Free Energies= -957.485407

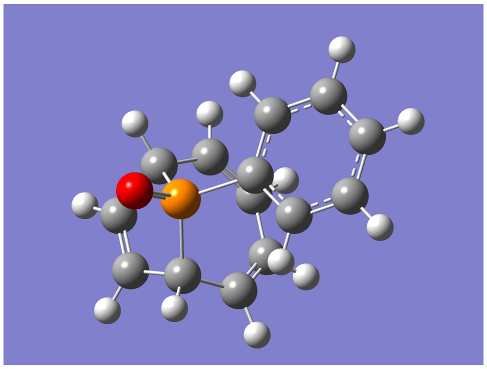

Center Number	Atomic Number	Atomic Type	Coordinates (Angstroms) X	Y	Z
1	6	0	0.140899	0.733142	-0.048431
2	6	0	1.418766	0.385352	-0.237613
3	6	0	0.761801	3.705211	-0.854510
4	6	0	2.448344	1.266807	0.449101
5	6	0	2.163840	3.323579	-1.062265
6	6	0	2.899439	2.328346	-0.531673
7	1	0	1.734625	-0.422737	-0.889323
8	1	0	-0.697031	0.239126	-0.529358
9	1	0	3.302549	0.695875	0.820242
10	1	0	3.931086	2.245610	-0.864786

11	6	0	-0.084934	1.956338	0.824326
12	1	0	-0.972301	1.859159	1.453861
13	6	0	-0.195570	3.170716	-0.072972
14	1	0	-1.177714	3.635888	-0.107444
15	15	0	1.436492	1.880992	1.862446
16	6	0	1.962163	3.542162	2.352369
17	6	0	1.014708	4.485992	2.759463
18	6	0	3.322469	3.859318	2.408715
19	6	0	1.423586	5.738436	3.209326
20	1	0	-0.045431	4.250929	2.722545
21	6	0	3.730474	5.111982	2.858723
22	1	0	4.068648	3.133846	2.096593
23	6	0	2.781128	6.052164	3.256689
24	1	0	0.683731	6.468352	3.521396
25	1	0	4.787567	5.354082	2.897575
26	1	0	3.099577	7.029304	3.605916
27	1	0	2.684653	3.962299	-1.771728
28	1	0	0.457563	4.568373	-1.441804
29	8	0	1.344813	0.919828	3.019330

34[O], ωB97x-D/6-31+G(d,p), $CHCl_3$ IEFPCM:
Sum of electronic and thermal Free Energies= -957.486822

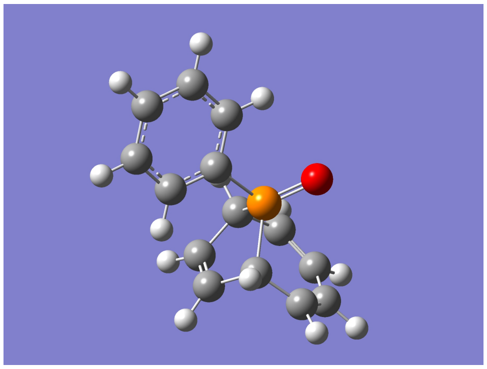

Center Number	Atomic Number	Atomic Type	Coordinates (Angstroms) X	Y	Z
1	6	0	1.148996	0.293054	1.742144
2	6	0	1.181924	1.422943	1.024332
3	6	0	3.435279	-0.960577	-0.024645
4	6	0	1.116395	1.252286	-0.479417
5	6	0	3.464422	0.277334	-0.814078
6	6	0	2.522471	1.213216	-1.038496
7	1	0	1.301932	2.409375	1.461908
8	1	0	1.240335	0.267412	2.823400
9	1	0	0.535638	2.038756	-0.967433
10	1	0	2.799533	2.054757	-1.668100

11	6	0	1.061354	-0.988957	0.943747
12	1	0	0.437183	-1.743897	1.427210
13	6	0	2.455878	-1.526157	0.706822
14	1	0	2.690147	-2.461315	1.209137
15	15	0	0.289384	-0.391279	-0.626221
16	1	0	4.422342	0.456608	-1.296753
17	1	0	4.376281	-1.505365	-0.044533
18	6	0	-1.487293	-0.149752	-0.301812
19	6	0	-2.344119	-1.131844	-0.813069
20	6	0	-2.029718	0.939418	0.390328
21	6	0	-3.720250	-1.034578	-0.622673
22	1	0	-1.928391	-1.963432	-1.373360
23	6	0	-3.406906	1.037280	0.573343
24	1	0	-1.386234	1.714716	0.791270
25	6	0	-4.253184	0.049778	0.071487
26	1	0	-4.374917	-1.801683	-1.023615
27	1	0	-3.818295	1.888046	1.107065
28	1	0	-5.326046	0.129204	0.216063
29	8	0	0.516257	-1.211941	-1.863878
